# Partisanship, health behavior, and policy attitudes in the early stages of the COVID-19 pandemic

**DOI:** 10.1371/journal.pone.0249596

**Published:** 2021-04-07

**Authors:** Shana Kushner Gadarian, Sara Wallace Goodman, Thomas B. Pepinsky

**Affiliations:** 1 Department of Political Science, Syracuse University, Syracuse, NY, United States of America; 2 Department of Political Science, University of California, Irvine, CA, United States of America; 3 Department of Government, Cornell University, Ithaca, NY, United States of America; Vanderbilt University, UNITED STATES

## Abstract

**Objective:**

To study the U.S. public’s health behaviors, attitudes, and policy opinions about COVID-19 in the earliest weeks of the national health crisis (March 20–23, 2020).

**Method:**

We designed and fielded an original representative survey of 3,000 American adults between March 20–23, 2020 to collect data on a battery of 38 health-related behaviors, government policy preferences on COVID-19 response and worries about the pandemic. We test for partisan differences COVID-19 related policy attitudes and behaviors, measured in three different ways: party affiliation, intended 2020 Presidential vote, and self-placed ideological positioning. Our multivariate approach adjusts for a wide range of individual demographic and geographic characteristics that might confound the relationship between partisanship and health behaviors, attitudes, and preferences.

**Results:**

We find that partisanship—measured as party identification, support for President Trump, or left-right ideological positioning—explains differences in Americans across a wide range of health behaviors and policy preferences. We find no consistent evidence that controlling for individual news consumption, the local policy environment, and local pandemic-related deaths erases the observed partisan differences in health behaviors, beliefs, and attitudes. In further analyses, we use a LASSO regression approach to select predictors, and find that a partisanship indicator is the most commonly selected predictor across the 38 dependent variables that we study.

**Conclusion:**

Our analysis of individual self-reported behavior, attitudes, and policy preferences in response to COVID-19 reveals that partisanship played a central role in shaping individual responses in the earliest months of the COVID-19 pandemic. These results indicate that partisan differences in responding to a national public health emergency were entrenched from the earliest days of the pandemic.

## Introduction

The coronavirus (COVID-19) pandemic has affected nearly every aspect of economic, social and political life in the United States. This public health emergency emerged in a media environment saturated with misinformation, rancorous partisan infighting, and messaging from the President that undermined health experts and undercut national unity [1]. As early as January, the administration downplayed its severity by comparing it to the seasonal flu, asserted that it was under control, and even described COVID-19 in racial terms (“Chinese” or “Wuhan” virus). Administrative missteps created further uncertainty, revealing severe mismanagement from the federal government and the Centers from the Disease Control (CDC), most prominently in the lack of nationwide test availability and reversing recommendations on face masking. And, differences in mitigation strategies among state governors exposed the deeply political nature of public health responses [2].

In this conflicting and noisy informational environment, how do individuals interpret a novel public health threat such as COVID-19? Americans, on average, pay little attention to political news [3–5], but in times of high threat, news consumption increases as people seek out steps to mitigate and avoid threats to their physical health and safety [6]. In a public health crisis, mass media provide health information in a very fast-moving environment [7] and mixed or poor health communication can increase risks to public health [8]. When individuals are worried about the risk of diseases and outbreaks directly, they put their trust in medical experts more than political leaders and will undertake behavioral changes to lower their risk as well as support policies framed as increasing their safety [9]. Self-interest does not always motivate political attitudes or decision-making, but in the face of salient threats that are explicitly connected to public policy, people will use their own experiences and interests to guide their attitudes and behaviors [10].

The COVID-19 pandemic is an example of a policy area where we may expect that mediated or direct personal experience with the pandemic would drive respondent to follow steps to keep themselves, their loved ones, and their communities safe in the early stages given its salience and immediate and dramatic policy changes at the state and local level. However, the messages Americans received from the mass media about the coronavirus pandemic varied by media source and what political leaders they trusted. President Donald Trump and conservative media publicly disagreed with public health experts about how serious the coronavirus pandemic and what types of policies can effectively manage it [11], making partisanship a potential component of public responses to the pandemic.

Partisanship is among the most powerful forces in American political life, and is particularly meaningful in contexts with conflicting and shifting information. Americans use partisan identification as a guide to help choose political candidates during elections [12], form attitudes [13] and process information [14]. More generally, this happens because partisanship operates as a social identity [15] that is increasingly tied to other important identities [16,17] and even personality type [18]. Finally, increasing partisan polarization on the elite level [19] and the rise of ideologically aligned media [20] combine to make partisans not only prefer their own party members over those in the opposite party [21] but also actively dislike members of the other party [22].

Partisan polarization also affects the public’s evaluations of the president’s performance in health crises as well as their own health behaviors. During the Obama administration, Republicans reported more concern over Ebola than Democrats [23] and Republicans in the public were less likely to get the H1N1 vaccine, particularly if they paid close attention to right wing media sources [24]. The public generally takes cues on what issues to be concerned about from the leaders of their own party [25], and also can use the president’s position to benchmark their own policy preferences [26].

A number of recent behavioral studies use aggregate data to establish correlations between partisanship and social distancing practices during COVID-19 [27–30]. Our individual-level analysis that avoids problems of ecological inference, and contains the rich individual covariates that can isolate partisanship from other factors correlated with it. Although other survey-based analyses have studied individual health behavior in response to COVID-19, ours is unique in two ways: it captures *early evidence* across the *widest range of behaviors and attitudes* available at the onset of the pandemic. This is in contrast to work that focuses on partisan affect at a later point in the pandemic [31] or that focuses more narrowly on self-reported social distancing only [32]. Relative to existing individual-level survey research on partisanship and health behavior in the early stages of the US pandemic [28,33], our analysis covers a much wider array of policy responses and attitudes.

This manuscript reports results from a novel survey in the first phase of widespread school closures and shelter-in-place policymaking (March 20–23, 2020). This was very early in the pandemic, a week after the World Health Organization declared COVID-19 a pandemic at a moment in which Americans were first experiencing lockdown measures, and in which there was great uncertainty about public health recommendations such as mask wearing. We find early, consistent partisan differences among Americans not only in terms of their desired public health and public policy responses, but also on self-reported health behavior, like hand-washing and social distancing practices. In contrast to existing literature on rally-around-the-flag effects in response to national disasters and health crises [34–36], we find that partisan differences emerged early on in the COVID-19 crisis in the United States.

Differing from the voluminous body of survey analyses by public opinion research firms such as Pew and Gallup, ours is an IRB-approved analysis of the partisan politics of COVID-19 that adjusts flexibly for a wide range of demographic and geographic differences, using a saturated indicator variable approach that allows for nonlinear relationships between plausible confounders, health behaviors, and attitudes. By employing this flexible approach with controls, we show that these partisan differences cannot be attributed to a wide range of demographic or geographic factors that are correlated with individual partisanship, nor are they significantly altered when accounting for differences in news consumption, local or state caseloads, or local or state policy responses. In further analysis, we use a LASSO regression approach to select the best predictors of COVID-related attitudes, beliefs, and behaviors. This procedure selects partisanship more often than any other predictor across our 38 outcome variables, further establishing that partisanship is of central importance for predicting COVID-related attitudes, beliefs, and behaviors.

Our findings put the subsequent course of the pandemic into sharp relief, as early divergence on COVID messaging—both in terms of seriousness of the pandemic and the substance of national politics, where Democrats emphasized health while Republicans focused on China and business [1]—and subsequent message-taking created a deep polarization at the individual level, in terms of behaviors and attitudes. Effective policy response that encourages broad pro-social behavior must attend not only to the source of the message, but also must address the deeply rooted individual partisan differences in beliefs and behavior that are unlikely to be moved by bipartisan messaging or trusted authorities alone [37].

## Data and methods

### Data

We fielded a large, nationally-representative survey of American adults (preregistered; *N =* 3000) between March 20 and March 23—one week after Trump declared a national emergency and contemporaneous to several state shutdown orders—to evaluate the relationship between partisan and political affiliations and health behaviors and policy preferences. Our sample was collected by YouGov (information on human subject participation is available in the Supporting Information). Summary demographic statistics from the sample can be found in S1 Table in S1 File. Sample size was determined by budgetary considerations. We do not exclude any respondents from our analysis, nor do we drop any respondents for missing data purposes.

Our research complies with all relevant ethical regulations in the United States, and was overseen by the Institutional Review Board for Human Participant Research at Cornell University (Protocol 2003009479), the Institutional Review Board at the Office of Research Integrity and Protections at Syracuse University (Protocol 20–099), and the University of California, Irvine (granted self-exemption with confirmation from the Office of Research, March 6, 2020). No deception was used. We obtained voluntary and informed consent from participants using an IRB-approved consent protocol. Consent was obtained from the respondents when they clicked “Yes” after having read this consent protocol. Informed consent was obtained from all respondents prior to the onset of the survey: participants were aware that they were taking part in a research study and had to affirmatively consent to proceed. No children participated in this study.

We collected information on four kinds of outcome variables: *health behaviors* (e.g., hand washing, self-quarantining), *health attitudes* (e.g., understanding of the scale of the threat, level of worry about COVID-19), *health policy views* (e.g., should public events be cancelled, should costs be waived for COVID-19 related treatment), and *public policy views* (e.g., should elections be delayed, should interest rates be lowered, should air travel be suspended), totaling 38 items altogether (we present a full list of dependent variables in S2 Table in S1 File). We also collected information on demographic covariates (e.g., age, gender, race, level of education, income, location) as well as three measures of political orientations and affiliations: partisan affiliation (Democrat, Republican, or Other, calculated from the Pew Research Center standard “PID3” variable), as well as intended 2020 Presidential vote choice (for Trump, for the Democrat, or for a third party or other) and ideological positioning (conservative, liberal, and moderate or non-ideological).

We find that there is wide variation in the health behaviors that our respondents reported (Table 1).

**Table 1:** Health behaviors.

	Mean	Standard Deviation
*Wash Hands*	0.855	0.352
*Bought Sanitizer*	0.407	0.491
*Visited the Doctor*	0.047	0.212
*Changed Travel Plans*	0.329	0.470
*Avoid Contact w/Others*	0.660	0.474
*Avoided Gatherings*	0.772	0.419
*Sought Info on COVID-19*	0.546	0.498
*Self-Quarantined*	0.357	0.479

We find that already by March 20–23, 85% of our respondents reported washing their hands more frequently than before, and 77% of our respondents were avoiding gatherings. Far fewer Americans reported self-quarantining (36%) or purchasing hand sanitizer (41%). These pictures in aggregate are encouraging, but do not capture the differences among Americans with respect to health behavior.

### Method

To explore those differences, we model the relationship between political variables and outcome variables using a flexible covariate adjusted logistic regression approach (with ordinal logistic regressions for ordinal dependent variables), comparing respondents in a standard between-subjects analysis. All analyses were conducted using Stata 15.1. Our baseline model takes the following form:

logit(P(Y=1))=α+βPartisanship+γX+ϵ

Where *Y* is each of our independent variables and *Partisanship* is a measure of partisanship. For ordered dependent variables, we replace the logit specification with an ordered logistic regression specification. We include as covariates **X** a full range of dummy variables for gender, four age categories, race (white versus nonwhite), marital status (married versus other), seventeen income levels, six education levels, a nine-level measure of county urban/rural status matched to respondent ZIP codes, and state of residence. This broad array of indicator variables allows for factors such as age, education, and rural status to have nonlinear relationships with outcome variables. Results using ordinary least squares regression—or representing age, education, urban-rural, and income as continuous variables—are substantively identical. Given the large number of dependent variables, we implement a strict Bonferroni correction, correcting for nine comparisons each for health views and health behaviors (nominal *α* = 0.05→0.0056) and for twenty comparisons for the policy outcomes (nominal *α* = 0.05→0.0025).

Last, we note that because our research design does not model the assignment of partisan identity, our statistical correlations should not be interpreted as causal relationships. They can only be given a causal interpretation with further assumptions. Specifically, the partisan differences that we identify below would have a causal interpretation if we assume unconfoundedness conditional on observed covariates [38] and that the logistic regression model is the correct functional form. We do not make these assumptions, so our results should be interpreted as covariate-adjusted partisan differences.

We also recognize survey results such as these are inherently vulnerable to social desirability bias in self-reported behavior, specifically partisan bias [39,40]. As such, Democrats may over-report pro-social behavior to signal their identity, whereas Republicans may report the opposite to do the same. Our survey design does not allow us to this rule out, though behavioral studies of COVID-19 [27–30,41] are reassuring external evidence that partisan differences in survey responses have analogues in real world behavior.

## Results

**Fig 1:**
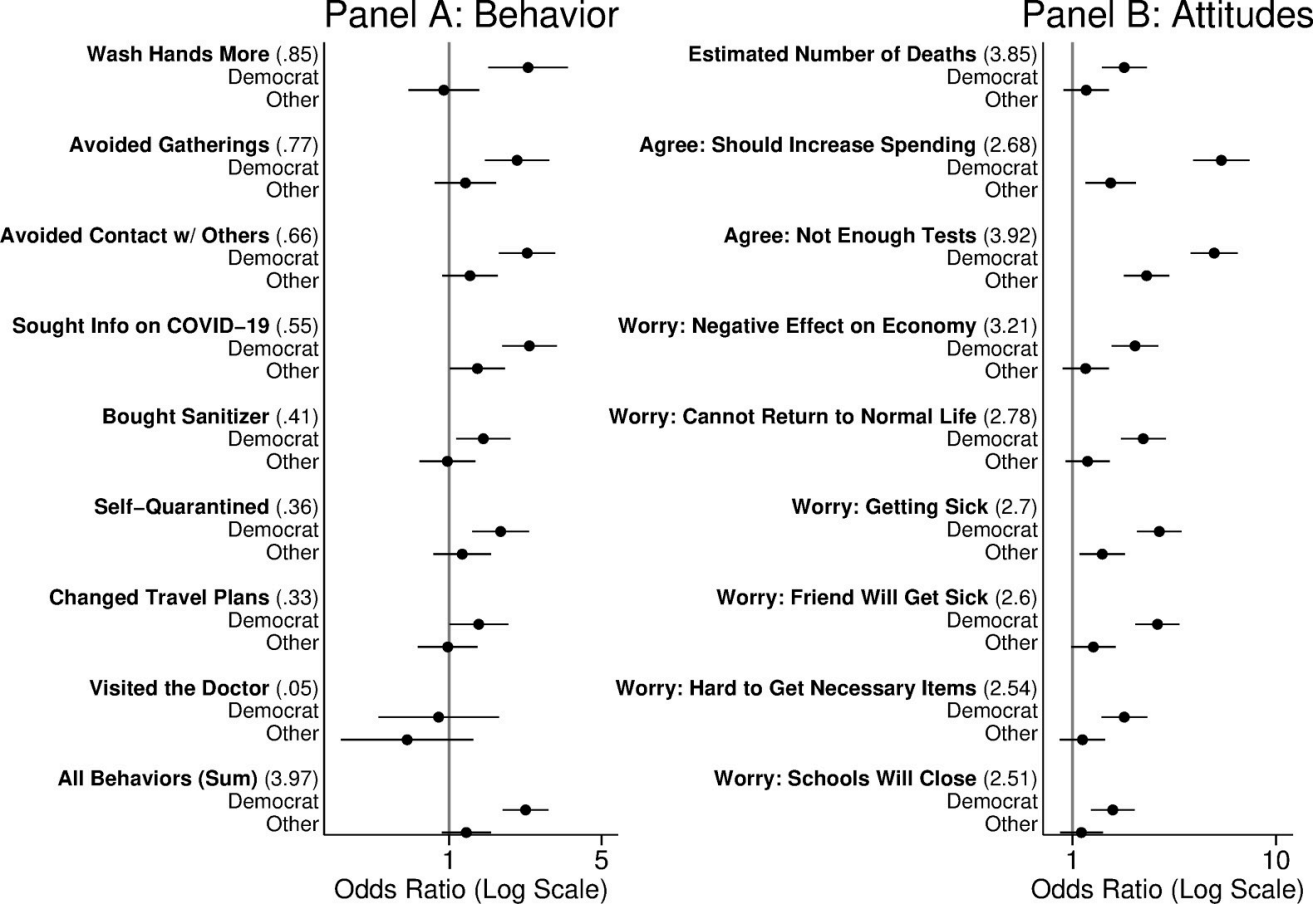
Partisanship and health behaviors and attitudes. Estimates are odds-ratios comparing Democrats and Others (unaffiliated or identifying with a third party) to self-identified Republicans. Odds ratios greater than 1 imply the respondent is more likely report a behavior or to express a view. 95% confidence intervals are adjusted for nine comparisons using a Bonferroni correction. For each dependent variable, the number in parentheses is the proportion saying yes (Panel A) or the mean response on a five-point scale (Panel B).

We present here results for the trichotomous partisan affiliation variable (analyses are substantively identical using intended vote choice or ideological positioning, see S1-S4 Figs in S1 File). Our results for the first two collections of outcome variables are in Fig 1 (full regression results are available in S3 and S4 Tables in S1 File).

We find strong evidence in Panel A that relative to Republicans, Democrats were already significantly more likely to report having adopted several health behaviors in response to COVID-19. These behaviors collectively reflect a practice of “social distancing” and aligned with CDC recommendations for preventing the spread of COVID-19. In Panel B, we also find strong evidence that relative to Republicans, Democrats were more worried about the pandemic. Democrats believed that the death toll was higher, that spending on public health responses should be increased, and were more likely to report an array of worries about the consequences of COVID-19 for their lives.

To illustrate the substantive differences in health behaviors between Democrats and Republicans, we calculate the adjusted risk differences between the two for each of our health behavior outcomes. These differences appear in Table 2.

**Table 2:** Adjusted risk differences, health behaviors. This table reports the adjusted risk differences for eight health behaviors. These are the differences between Republicans and Democrats from the statistical models in Fig 1 (S3 Table in S1 File), adjusting for all other covariates.

	Adjusted Risk Difference, Democrats–Republicans	Standard Error	p-value	95% Confidence Interval	
*Wash Hands*	0.088	0.017	0.000	0.056	0.120
*Bought Sanitizer*	0.083	0.023	0.000	0.037	0.129
*Visited the Doctor*	-0.005	0.011	0.632	-0.027	0.017
*Changed Travel Plans*	0.063	0.022	0.005	0.020	0.107
*Avoid Contact w/Others*	0.173	0.022	0.000	0.129	0.216
*Avoided Gatherings*	0.116	0.020	0.000	0.076	0.155
*Sought Info on COVID-19*	0.193	0.023	0.000	0.148	0.238
*Self-Quarantined*	0.118	0.023	0.000	0.073	0.162

The results reveal a stark partisan divide in health attitudes (Fig 1) and behaviors (Table 2) on COVID-19.

Substantively, how large are these differences? In Table 3 we benchmark the partisan differences that we have uncovered relative to differences across two highly salient demographic differences that may also affect COVID-19 behaviors: income and education. Specifically, we estimate the adjusted risk differences for the eight health behaviors previously analyzed, comparing first Americans with a family income of $50,000/year and those earning $150,000/year (Panel A), and second Americans with a college degree and those who only possess a high school diploma (Panel B).

**Table 3:** Adjusted risk differences for income and education. This table reports the adjusted risk differences for eight health behaviors. These are the differences between Americans earning $150,000 per year and those earning $50,000 per year, from the statistical models in Fig 1 (S3 Table in S1 File), adjusting for all other covariates.

**Panel A**					
	**Adjusted Risk Difference, 150,000/year– 50,000/year**	**Standard Error**	**p-value**	**95% Confidence Interval**	
*Wash Hands*	0.048	0.034	0.153	-0.018	0.115
*Bought Sanitizer*	0.064	0.057	0.259	-0.047	0.175
*Visited the Doctor*	-0.012	0.024	0.614	-0.059	0.035
*Changed Travel Plans*	0.149	0.053	0.005	0.045	0.252
*Avoid Contact w/Others*	0.078	0.054	0.149	-0.028	0.184
*Avoided Gatherings*	0.014	0.048	0.767	-0.080	0.108
*Sought Info on COVID-19*	0.002	0.058	0.971	-0.111	0.115
*Self-Quarantined*	0.024	0.054	0.660	-0.082	0.129
**Panel B**					
	**Adjusted Risk Difference, College Graduate–High School Graduate**	**Standard Error**	**p-value**	**95% Confidence Interval**	
*Wash Hands*	-0.048	0.022	0.031	-0.091	-0.004
*Bought Sanitizer*	0.036	0.032	0.266	-0.027	0.100
*Visited the Doctor*	-0.023	0.015	0.112	-0.052	0.005
*Changed Travel Plans*	-0.078	0.031	0.011	-0.138	-0.018
*Avoid Contact w/Others*	-0.030	0.031	0.333	-0.090	0.031
*Avoided Gatherings*	-0.038	0.027	0.152	-0.090	0.014
*Sought Info on COVID-19*	-0.029	0.032	0.362	-0.093	0.034
*Self-Quarantined*	-0.036	0.031	0.248	-0.097	0.025

These results illustrate that the partisan differences in behavior that we have identified are almost always substantively larger than those associated with salient differences in education and income. They are also almost always estimated with far more precision. The exception to both of the preceding statements is the result for changing travel plans: here, the partisan difference is small and imprecisely estimated, but the difference according to income is large and statistically significant at the *p* < .01 level.

**Fig 2:**
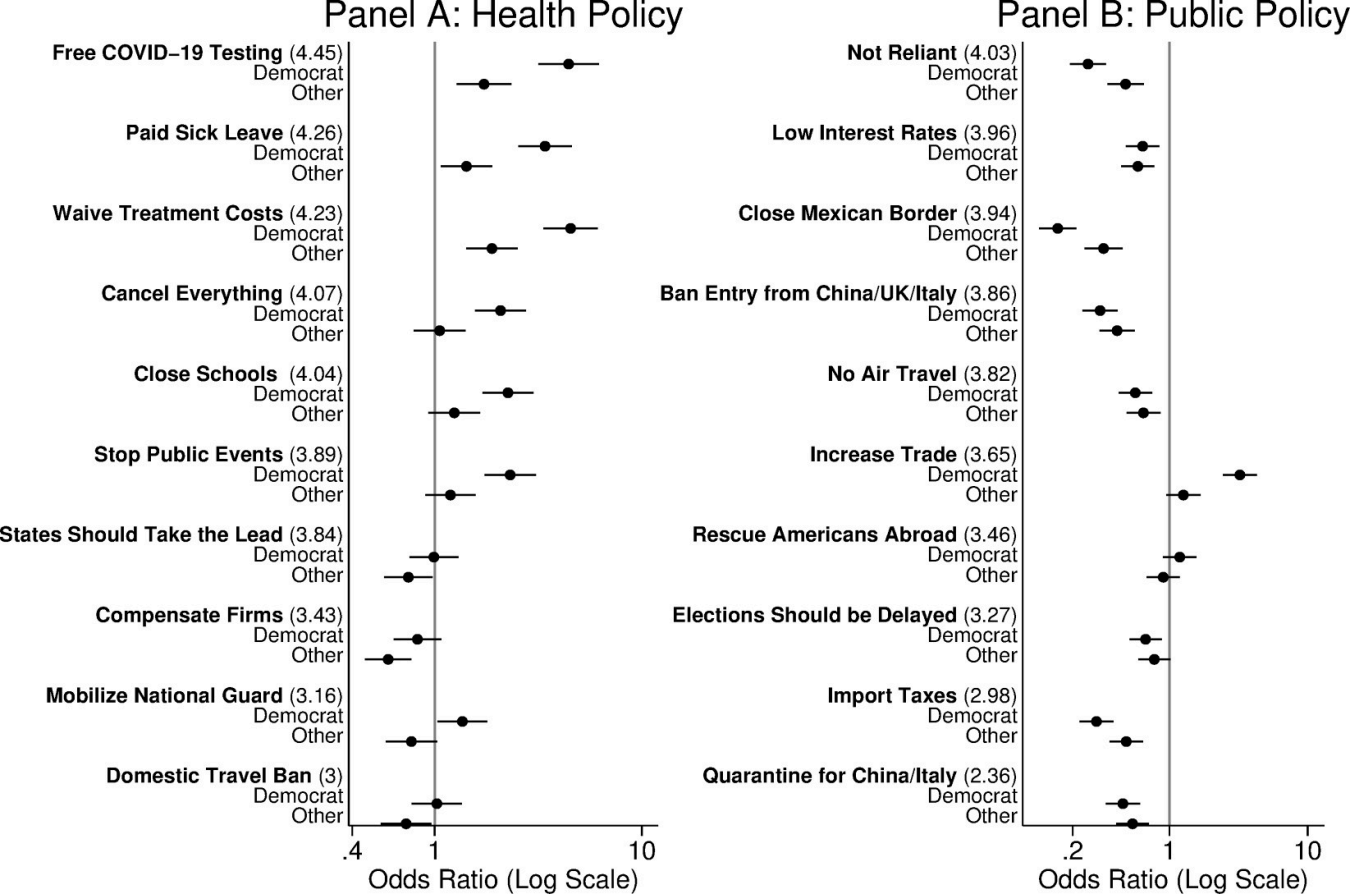
Partisanship and policy preferences. Odds ratios greater than 1 imply the respondent is more likely to support a policy. 95% confidence intervals are adjusted for twenty comparisons using a Bonferroni correction. For each dependent variable, the number in parentheses is the mean response on a five-point scale.

In Fig 2, we show that partisan differences in behaviors and attitudes also spill over into policy preferences (full regression results are available in S5 and S6 Tables in S1 File).

In Panel A, we observe strong partisan differences in public health responses such as socializing the costs of diagnosis and treatment. We also observe that Democrats were already in March much more likely to support some measures that support social distancing (such as cancelling public events and covering workers with paid sick leave). There were no partisan differences in support for firms, restrictions on domestic travel, or support for state governments taking the lead in the public health response.

We observe further partisan differences in trade and immigration policy responses that were framed as responding to the pandemic. Democrats were more likely to support free trade and to oppose import taxes in response to COVID-19; they were also less likely to support expansionary macroeconomic policy to support economic growth. Democrats were far less supportive than Republicans of policies designed to halt the spread of COVID-19 through restrictions on international travel or movement. Finally, Democrats were less likely to support delaying elections until the COVID-19 threat has passed.

One possible explanation for these results is that if Democrats tend to live in urban or cosmopolitan locations that are themselves more affected by COVID-19 in this first phase of spread, then these correlations are simply reflecting objective conditions of the pandemic. Our fixed effects by state and urban-rural locality, however, minimize this particular inferential threat. Furthermore, we estimate multilevel regression models (S7-S18 Tables and S5-S8 Figs in S1 File) with ZIP code-level random intercepts [42] that control for county-level COVID-19 diagnoses and county-level COVID-19 deaths as of March 23 (available from the *New York Times* COVID-19 tracking project at https://github.com/nytimes/covid-19-data/blob/master/us-counties.csv) along with state fixed effects. Our findings about partisanship remain unchanged, and objective country-level indicators of COVID-19 severity on March 23, 2020 are never correlated with any outcome variable.

To confirm the predictive importance of partisanship, we adopt a regularized regression approach to select what variables among the political and demographic predictors above best predict the outcomes above. Specifically, for each of the 38 outcomes, we use (linear) lasso regression [43] to select from among the 87 predictors we included in the analysis above, and check to see whether partisanship is among them [we allow the penalization to be data-driven, following methods in 44]. This approach selects our variable capturing Democrats in 31 out of 38 regressions (see S19-S22 Tables in S1 File). The next most commonly selected variable is a dummy for respondents who have completed a High School education only, selected in only 17 regressions. These findings comprise powerful evidence that in addition to being a good predictor of health behaviors and attitudes, partisanship is the most consistently related to health behaviors and attitudes among the predictors we have included.

### Explaining partisan differences

What is driving these differences in attitudes and behaviors across partisan identifiers? In additional analyses, we investigate both the robustness of our main findings to additional factors that might confound the relationship between partisan identity and coronavirus-related behaviors and attitudes. First, it could be that differences in partisanship are attributable to selective exposure by way of differences in news consumption [45]. If Republicans are more likely to view Fox News or other news sources that downplayed the severity of the crisis and undermined public messaging on health behaviors, then this would confound our estimate of the observed differences across parties. Unlike other control variables like age, gender, and place of residence, however, news consumption might itself be affected by partisanship, meaning that we must adopt special care in investigating how media consumption, partisanship, and health behaviors. If we found, for example, that controlling for new consumption eliminated partisan differences in health behaviors, we might infer that news consumption is the mechanism through which partisanship affiliation shapes health behaviors,

To investigate the role of news sources in explaining partisan differences, we create a measure of right-wing news consumption from respondents’ self-reported news sources (see the Supporting Information for further explanation and coding procedure). We then proceed in two ways. First, we treat right-wing news consumption as a confounder, and control for it in our regressions (see S23-S26 Tables in S1 File). We find no evidence that adjusting for right-wing news sources explains partisan differences in health behaviors.

Next, we check whether right-wing news consumption moderates the partisan differences that we have identified by interacting the partisanship variables with the right-wing news variable. We find no general pattern in the results that suggests that the broad partisan differences in behaviors, attitudes, or preferences are larger among those who consume right-wing news (see S27-S30 Tables and S9-S12 Figs in S1 File), a result that differs with recent behavioral studies on the effect of Fox News viewership on social distancing behavior over time [41].

Finally, we explore whether the local policymaking environment might explain our results. Although policy environments are unlikely to be themselves driven by individual partisanship, it is possible that those municipalities where we observe many Democrats are also those that implement the most aggressive policy responses.

We using data on the local policymaking environment in American municipalities [46] to control for this possibility. These data code urban areas according to whether or not they have implemented six policies: testing facilities, shelter in place orders, social distancing, closing businesses, limiting business hours, and limiting the size of gatherings. We create an additive index of COVID-19 response policies from these six indicators, and then include it as a control variable. Because the local policymaking data only include residents of urban areas, we lose approximately 33% of our respondents, but our results are nevertheless revealing (see S31-S34 Tables in S1 File). We find that accounting for the number of COVID policies implemented in each locality does not eliminate any of the partisan differences that we identified in the main analysis.

Finally, we investigate the robustness of our estimated partisan differences to different choices of control variables. We find that the size and statistical significance of these estimated partisan differences generally does not depend on whether we control or not for individual demographics, geography, or the combination of the two (see S13 and S14 Figs in S1 File).

## Discussion

Our results collectively describe a sharp and wide-reaching political divide as an early reaction to COVID-19: already by mid-March, Republicans were less likely than Democrats to report responding with CDC-recommended behavior, and were less concerned about the pandemic, yet were more likely to support policies that restrict trade and movement across borders as a response to it. Democrats, by contrast, responded by changing their personal health behaviors, and supported policies that socialize the costs of testing and treatment. Thus, in the first month of COVID-19 in the US partisanship was the best predictor of differences in behaviors, attitudes, and preferences than anything else that we measure.

Our analysis does not allow us to explain the mechanisms that link partisan identity to public health responses. Although we note that partisan elites politicized COVID-19 from the very onset of the pandemic in early 2020, it is also conceivable that Americans’ partisan affiliations capture more fundamental dispositions towards public health messaging and pandemic response. However, recalling that Republicans reported *more* concern over Ebola than Democrats under a Democratic presidential administration [23], we find it unlikely that partisan differences in behavior towards COVID-19 simply reflect fundamental partisan dispositions towards public health and pandemic response. Further research may be able to adjudicate among potential explanations for the partisan differences that we have uncovered.

Because an effective public health response to a rapidly-moving influenza pandemic such as COVID-19 requires consistent participation and collective action across communities [47,48] in addition to broader public health interventions [49], our findings have disturbing implications for health crisis management. An effective strategy for mitigating the damage of COVID-19 requires citizens to practice social distancing measures and state and local governments to participate in them regardless of political leanings or partisan affiliations. Aggregate behavioral data shows that partisanship predicts social distancing practices (27, 28, 30), and that partisan differences in social distancing, in turn, predict subsequent infection rates (29). Our findings are the strongest evidence available that partisanship had already shaped the course of COVID-19 at the individual level at the pandemic’s onset, which in turn helps to account for the tragic timeline of the American experience of the COVID-19 pandemic. An effective COVID-19 public health campaign would have exhibited bipartisan solidarity through a common message, endorsed across parties and by political elites of all persuasions, to slow the pandemic and ease the strain on health services (“flatten the curve”). Facing such rooted partisan disagreement about the pandemic, and with the Trump administration unwilling to take decisive bipartisan action, the arc of the pandemic was determined early on.

An effective public health response must confront the deeply rooted partisan politics of the crisis. Such a response must be pitched at the federal level: our analysis both of local policy environments and of local case loads reveals that although local context matters, it does not erase systematic partisan differences across Americans writ large. Without a federal response that confronts the partisan politics of the crisis, messaging that attempts to sidestep partisanship—using clergy, celebrities, health experts, etc.—will be undermined by this deepest cleavage.

## Supporting information

S1 File (DOCX)
