# Supplementary material for: Partisanship, health behavior, and policy attitudes in the early stages of the COVID-19 pandemic
Source: PLoS One. 2021 Apr 7;16(4):e0249596. doi: 10.1371/journal.pone.0249596 (PMC8026027; doi:10.1371/journal.pone.0249596)
Supplement: S1 File — (DOCX) [file pone.0249596.s001.docx]

Supplementary Materials:

Partisanship, Health Behavior, and Policy Attitudes

in the Early Stages of the COVID-19 Pandemic

# Human Subject Participation and Informed Consent

Our research complies with all relevant ethical regulations in the United States, and was overseen by the Institutional Review Board for Human Participant Research at Cornell University (Protocol 2003009479), the Institutional Review Board at the Office of Research Integrity and Protections at Syracuse University (Protocol 20-099), and the University of California, Irvine (granted self-exemption with confirmation from the Office of Research, March 6, 2020). No deception was used. We obtained voluntary and informed consent from participants using an IRB-approved consent protocol. Consent was obtained from the respondents when they clicked “Yes” after having read this consent protocol. Informed consent was obtained from all respondents prior to the onset of the survey: participants were aware that they were taking part in a research study and had to affirmatively consent to proceed. No children participated in this study.

Informed consent was obtained from all respondents prior to the onset of the survey, and respondents were not compensated by us for participation in our study (though they were compensated by YouGov, which maintains an internal point system for its 3 million person panel that participants can use to redeem awards, which include cash and gift cards. We do not determine the internal point amount, and amounts may vary across waves in order to induce participation).

Participants were aware that they were taking part in a research study and had to affirmatively consent to proceed. Participants received the following consent document as their first screen:

You are invited to participate in a survey on your views about current public health issues conducted by YouGov in conjunction with researchers from Universities A, B, and C.

The goal of this survey is to obtain opinions on current events. You will be asked a number of questions on current events. Your participation is voluntary. You are free not to answer any question or to withdraw from the study at any time. This poll should take approximately 10 minutes to complete.

To be eligible to participate in this study, you must be an adult (aged 18 and over).

A report of the results of this study will be provided to you upon request. In order to analyze responses to our questionnaire, your answers will be recorded. *No identifying information about you will be made public and any views you express will be kept completely confidential.*

Findings from this study will be reported in scholarly journals, at academic seminars, and at research association meetings. The data will be stored at a secured location and retained indefinitely. There are no foreseeable risks in participating. A benefit from participating in this study is that it may increase awareness of current events.

Should you have questions regarding the research project, please contact Shana Kushner Gadarian [sgadaria@maxwell.syr.edu], Sara Wallace Goodman [s.goodman@uci.edu], or Thomas B. Pepinsky [pepinsky@cornell.edu].

By clicking ‘yes’ you are confirming your willingness to participate in this study and verifying your eligibility to do so.

S1 Table. Summary Statistics.

| Variable | N | Mean | St. Dev. | Minimum | Maximum |
| --- | --- | --- | --- | --- | --- |
| *Age* | 3,000 | 49.8 | 16.9 | 19 | 92 |
| *Education Level* | 3,000 | 3.5 | 1.5 | 1 | 6 |
| *Income Level* | 3,000 | 7.8 | 4.8 | 1 | 17 |
|  |  |  |  |  |  |
| *Race* | White | 2,171 | 72.37 |  |  |
|  | Black | 308 | 1.27 |  |  |
|  | Hispanic | 322 | 1.73 |  |  |
|  | Asian | 70 | 2.33 |  |  |
|  | Native American | 35 | 1.17 |  |  |
|  | Mixed | 50 | 1.67 |  |  |
|  | Other | 43 | 1.43 |  |  |
|  | Middle Eastern | 1 | .03 |  |  |
|  |  |  |  |  |  |
| *Marital Status* | Married | 1,443 | 48.1 |  |  |
|  | Separated | 48 | 1.6 |  |  |
|  | Divorced | 331 | 11.03 |  |  |
|  | Widowed | 183 | 6.1 |  |  |
|  | Never married | 843 | 28.2 |  |  |
|  | Domestic/civil partnership | 149 | 4.97 |  |  |
|  |  |  |  |  |  |
| Gender | Female | 1,596 | 53.2 |  |  |
|  | Male | 1,404 | 46.8 |  |  |

S2 Table. List of Dependent Variables.

| **Variable Name** | **Description** | **Distribution** |
| --- | --- | --- |
| washhands | Washing hands more often | 2564 1 selected  436 0 not selected |
| boughtsanitizer | Bought hand sanitizer | 1221 1 selected  1779 0 not selected |
| visitdoctor | Gone to the doctor | 141 1 selected  2859 0 not selected |
| changedtravel | Altered travel plans | 988 1 selected  2012 0 not selected |
| avoidcontact | Avoid physical contact | 1980 1 selected  1020 0 not selected |
| avoidgatherings | Avoid large gatherings | 2317 1 selected  683 0 not selected |
| soughtinfo | Looked for information about coronavirus in the media | 1638 1 selected  1362 0 not selected |
| selfquarantine | Self-quarantined | 1070 1 selected  1930 0 not selected |
| changehealth | Washhands + boughtsanitizer + visitdoctor + changedtravel + avoidcontact + avoidgatherings + soughtinfo + selfquarantine |  |
| numberdeaths | Estimate of Coronavirus deaths in the US | 29 0 None  269 1 1-25  832 2 25-100  1198 3 100-200  348 4 200-500  324 5 More than 500 |
| spendingincrease | *Reverse coding of* Should federal spending on preventing infectious diseases  increase or decrease | 2110 1 Increasing spending  810 2 No change  78 3 Decreasing spending  2 . skipped |
| notenoughtests | *Reverse coding of* We have enough coronavirus tests in my state | 1369 1 Strongly disagree  414 2 Somewhat disagree  914 3 Neither agree nor disagree  171 4 Somewhat agree  117 5 Strongly agree  15 . skipped |
| getsick | Worried: You will get sick from coronavirus | 391 1 Not at all worried  1017 2 Somewhat worried  689 3 Worried  901 4 Very worried  2 . skipped |
| negativeeconomy | Negative impact on economy | 147 1 Not at all worried  583 2 Somewhat worried  767 3 Worried  1501 4 Very worried  2 . skipped |
| friendsick | Friend gets sick | 418 1 Not at all worried  1098 2 Somewhat worried  763 3 Worried  720 4 Very worried  1 . skipped |
| cannotreturn | Cannot return to way things were | 360 1 Not at all worried  910 2 Somewhat worried  764 3 Worried  965 4 Very worried  1 . skipped |
| schoolsclosed | Schools will be closed | 654 1 Not at all worried  951 2 Somewhat worried  614 3 Worried  780 4 Very worried  1 . skipped |
| hardgetitems | Hard to get items you need | 482 1 Not at all worried  1126 2 Somewhat worried  669 3 Worried  720 4 Very worried  3 . skipped |
| increasetrade | Increase trade | 136 1 Strongly disagree  229 2 Somewhat disagree  929 3 Neither agree nor disagree  925 4 Somewhat agree  765 5 Strongly agree  16 . skipped |
| importtaxes | Increase taxes on foreign imports | 418 1 Strongly disagree  481 2 Somewhat disagree  1166 3 Neither agree nor disagree  580 4 Somewhat agree  345 5 Strongly agree  10 . skipped |
| lowinterest | Keep interest rates low | 84 1 Strongly disagree  176 2 Somewhat disagree  669 3 Neither agree nor disagree  912 4 Somewhat agree  1145 5 Strongly agree  14 . skipped |
| notreliant | Not be reliant on other countries | 112 1 Strongly disagree  213 2 Somewhat disagree  514 3 Neither agree nor disagree  786 4 Somewhat agree  1363 5 Strongly agree  12 . skipped |
| noairtravel | Halt all international air travel | 170 1 Strongly disagree  335 2 Somewhat disagree  541 3 Neither agree nor disagree  774 4 Somewhat agree  1174 5 Strongly agree  6 . skipped |
| banentry | Ban the entry of citizens of China/Italy/Great Britain | 171 1 Strongly disagree  246 2 Somewhat disagree  626 3 Neither agree nor disagree  733 4 Somewhat agree  1215 5 Strongly agree  9 . skipped |
| entrymexico | Impose entry restrictions at the US-Mexico border | 182 1 Strongly disagree  235 2 Somewhat disagree  532 3 Neither agree nor disagree  661 4 Somewhat agree  1380 5 Strongly agree  10 . skipped |
| quarantine | Quarantine Chinese/Italians in the United States | 1198 1 Strongly disagree  481 2 Somewhat disagree  689 3 Neither agree nor disagree  274 4 Somewhat agree  342 5 Strongly agree  16 . skipped |
| rescueUS | Rescue US citizens from abroad | 206 1 Strongly disagree  373 2 Somewhat disagree  929 3 Neither agree nor disagree  794 4 Somewhat agree  686 5 Strongly agree  12 . skipped |
| freetesting | Make all testing for COVID-19 free for all Americans | 66 1 Strongly disagree  86 2 Somewhat disagree  315 3 Neither agree nor disagree  480 4 Somewhat agree  2041 5 Strongly agree  12 . skipped |
| waivecosts | The government should waive insurance costs and hospital fees  for treating COVID-19 | 74 1 Strongly disagree  175 2 Somewhat disagree  439 3 Neither agree nor disagree  607 4 Somewhat agree  1697 5 Strongly agree  8 . skipped |
| closeschools | Close all schools in order to contain the spread of coronavirus | 111 1 Strongly disagree  201 2 Somewhat disagree  485 3 Neither agree nor disagree  855 4 Somewhat agree  1332 5 Strongly agree  16 . skipped |
| canceleverything | Ban public events | 106 1 Strongly disagree  200 2 Somewhat disagree  460 3 Neither agree nor disagree  846 4 Somewhat agree  1379 5 Strongly agree  9 . skipped |
| paidleave | Grant paid leave to anyone diagnosed with coronavirus | 89 1 Strongly disagree  129 2 Somewhat disagree  420 3 Neither agree nor disagree  640 4 Somewhat agree  1713 5 Strongly agree  9 . skipped |
| compensatefirms | Provide compensation to American companies | 263 1 Strongly disagree  410 2 Somewhat disagree  707 3 Neither agree nor disagree  984 4 Somewhat agree  626 5 Strongly agree  10 . skipped |
| nationalguard | Mobilize the National Guard to enforce quarantines | 428 1 Strongly disagree  468 2 Somewhat disagree  866 3 Neither agree nor disagree  670 4 Somewhat agree  560 5 Strongly agree  8 . skipped |
| stopevents | Sporting and other public events should continue to take place | 1413 1 Strongly disagree  578 2 Somewhat disagree  476 3 Neither agree nor disagree  298 4 Somewhat agree  228 5 Strongly agree  7 . skipped |
| travelban | Domestic travel ban | 583 1 Strongly disagree  542 2 Somewhat disagree  713 3 Neither agree nor disagree  599 4 Somewhat agree  551 5 Strongly agree  12 . skipped |
| stateslead | State governments should lead | 105 1 Strongly disagree  261 2 Somewhat disagree  605 3 Neither agree nor disagree  1065 4 Somewhat agree  955 5 Strongly agree  9 . skipped |
| delayelections | Elections should be delayed | 594 1 Strongly disagree  329 2 Somewhat disagree  564 3 Neither agree nor disagree  700 4 Somewhat agree  805 5 Strongly agree  8 . skipped |

S3 Table. Regression Results, Health Behaviors.

|  | (1) | (2) | (3) | (4) | (5) | (6) | (7) | (8) | (9) |
| --- | --- | --- | --- | --- | --- | --- | --- | --- | --- |
|  | washhands | boughtsanitizer | visitdoctor | changedtravel | avoidcontact | avoidgatherings | soughtinfo | selfquarantine | changehealth |
| Democrat | .834 | .362 | -.111 | .313 | .825 | .718 | .848 | .544 | .808 |
|  | .151 | .103 | .229 | .112 | .107 | .122 | .104 | .107 | .088 |
|  | [.538, 1.130] | [.161, .563] | [-.560, .338] | [.094, .532] | [.615, 1.035] | [.478, .958] | [.645, 1.051] | [.334, .754] | [.636, .979] |
|  | (<.001) | (<.001) | (.628) | (.005) | (<.001) | (<.001) | (<.001) | (<.001) | (<.001) |
| Other | -.055 | -.019 | -.443 | -.014 | .221 | .172 | .299 | .138 | .181 |
|  | .135 | .106 | .252 | .114 | .105 | .117 | .104 | .110 | .093 |
|  | [-.320, .209] | [-.226, .189] | [-.938, .052] | [-.237, .209] | [.014, .427] | [-.057, .401] | [.095, .503] | [-.077, .354] | [-.002, .364] |
|  | (.681) | (.861) | (.079) | (.903) | (.036) | (.140) | (.004) | (.208) | (.052) |
| 35- | .047 | .222 | .018 | -.239 | -.001 | -.054 | -.034 | -.194 | -.073 |
|  | .157 | .116 | .257 | .122 | .118 | .130 | .118 | .118 | .103 |
|  | [-.261, .356] | [-.006, .450] | [-.485, .521] | [-.479, -.000] | [-.232, .230] | [-.309, .201] | [-.267, .198] | [-.425, .037] | [-.274, .128] |
|  | (.764) | (.056) | (.945) | (.050) | (.995) | (.677) | (.771) | (.099) | (.476) |
| 50- | .196 | .226 | -.166 | -.321 | .075 | .313 | -.155 | -.337 | -.035 |
|  | .153 | .110 | .263 | .114 | .112 | .128 | .110 | .112 | .094 |
|  | [-.104, .496] | [.010, .441] | [-.681, .350] | [-.545, -.097] | [-.144, .294] | [.062, .565] | [-.371, .061] | [-.556, -.118] | [-.219, .150] |
|  | (.201) | (.040) | (.529) | (.005) | (.502) | (.015) | (.160) | (.003) | (.712) |
| 70- | .545 | .300 | -.291 | -.112 | .512 | .981 | -.316 | .281 | .291 |
|  | .209 | .138 | .338 | .146 | .150 | .183 | .138 | .138 | .113 |
|  | [.136, .954] | [.029, .570] | [-.953, .371] | [-.398, .174] | [.218, .805] | [.622, 1.340] | [-.586, -.046] | [.010, .552] | [.069, .512] |
|  | (.009) | (.030) | (.388) | (.443) | (.001) | (<.001) | (.022) | (.042) | (.010) |
| gender | .346 | .070 | -.073 | -.066 | .033 | .260 | .161 | .277 | .166 |
|  | .111 | .079 | .183 | .085 | .083 | .093 | .080 | .082 | .067 |
|  | [.129, .563] | [-.085, .225] | [-.431, .286] | [-.233, .100] | [-.129, .194] | [.077, .443] | [.004, .317] | [.115, .439] | [.034, .298] |
|  | (.002) | (.374) | (.692) | (.434) | (.692) | (.005) | (.045) | (.001) | (.014) |
| white | -.076 | -.358 | -.649 | -.172 | .212 | .297 | .365 | .011 | .026 |
|  | .135 | .093 | .209 | .102 | .097 | .110 | .096 | .098 | .089 |
|  | [-.341, .190] | [-.541, -.175] | [-1.058, -.240] | [-.371, .028] | [.021, .403] | [.081, .512] | [.176, .554] | [-.182, .204] | [-.148, .200] |
|  | (.576) | (<.001) | (.002) | (.091) | (.029) | (.007) | (<.001) | (.910) | (.774) |
| married | -.044 | .202 | .345 | .215 | .157 | -.030 | .094 | .045 | .157 |
|  | .121 | .087 | .214 | .093 | .091 | .103 | .088 | .090 | .073 |
|  | [-.281, .193] | [.032, .372] | [-.073, .764] | [.033, .397] | [-.021, .335] | [-.232, .172] | [-.078, .265] | [-.131, .221] | [.014, .301] |
|  | (.714) | (.020) | (.106) | (.020) | (.083) | (.769) | (.285) | (.619) | (.031) |
| High school graduate | -.131 | .024 | -.009 | .575 | -.560 | -.227 | .012 | -.003 | -.111 |
|  | .291 | .231 | .492 | .313 | .245 | .256 | .231 | .243 | .215 |
|  | [-.702, .439] | [-.428, .476] | [-.974, .955] | [-.039, 1.188] | [-1.040, -.081] | [-.729, .274] | [-.441, .465] | [-.478, .473] | [-.532, .310] |
|  | (.652) | (.917) | (.985) | (.066) | (.022) | (.374) | (.957) | (.992) | (.606) |
| Some college | .170 | -.044 | -.441 | 1.038 | -.281 | .128 | .591 | .078 | .255 |
|  | .298 | .235 | .517 | .314 | .249 | .262 | .235 | .247 | .217 |
|  | [-.415, .754] | [-.504, .417] | [-1.454, .573] | [.423, 1.652] | [-.769, .207] | [-.385, .642] | [.131, 1.051] | [-.407, .562] | [-.171, .680] |
|  | (.569) | (.852) | (.394) | (.001) | (.260) | (.624) | (.012) | (.753) | (.241) |
| 2-year | .117 | .032 | -.179 | .812 | -.322 | .084 | .546 | -.034 | .165 |
|  | .316 | .248 | .542 | .326 | .260 | .276 | .247 | .261 | .228 |
|  | [-.502, .737] | [-.454, .517] | [-1.243, .884] | [.173, 1.451] | [-.832, .187] | [-.458, .625] | [.062, 1.030] | [-.545, .477] | [-.282, .613] |
|  | (.710) | (.898) | (.741) | (.013) | (.215) | (.762) | (.027) | (.896) | (.469) |
| 4-year | .580 | -.128 | .333 | 1.185 | -.180 | .329 | .674 | .136 | .379 |
|  | .318 | .240 | .518 | .317 | .254 | .271 | .240 | .251 | .218 |
|  | [-.042, 1.203] | [-.599, .342] | [-.682, 1.348] | [.564, 1.806] | [-.678, .319] | [-.201, .860] | [.204, 1.144] | [-.357, .628] | [-.048, .806] |
|  | (.068) | (.593) | (.520) | (<.001) | (.480) | (.224) | (.005) | (.589) | (.082) |
| Post-grad | .511 | -.086 | .493 | 1.628 | .171 | .590 | 1.035 | .312 | .696 |
|  | .344 | .252 | .563 | .324 | .273 | .297 | .256 | .262 | .226 |
|  | [-.163, 1.185] | [-.580, .408] | [-.611, 1.597] | [.992, 2.263] | [-.364, .706] | [.007, 1.172] | [.532, 1.537] | [-.201, .825] | [.253, 1.139] |
|  | (.138) | (.733) | (.381) | (<.001) | (.531) | (.047) | (<.001) | (.234) | (.002) |
| income=2 | -.112 | -.170 | -1.323 | -.041 | -.024 | -.148 | .094 | -.272 | -.139 |
|  | .294 | .224 | .495 | .260 | .230 | .245 | .225 | .228 | .215 |
|  | [-.687, .464] | [-.608, .268] | [-2.292, -.353] | [-.551, .469] | [-.474, .426] | [-.629, .332] | [-.348, .535] | [-.720, .176] | [-.561, .284] |
|  | (.704) | (.447) | (.007) | (.875) | (.916) | (.545) | (.678) | (.234) | (.520) |
| income=3 | -.255 | -.228 | -.808 | -.133 | -.182 | .007 | .219 | -.570 | -.211 |
|  | .280 | .218 | .406 | .253 | .222 | .240 | .216 | .222 | .201 |
|  | [-.805, .294] | [-.654, .199] | [-1.604, -.011] | [-.629, .364] | [-.617, .253] | [-.464, .477] | [-.203, .642] | [-1.005, -.135] | [-.604, .182] |
|  | (.362) | (.296) | (.047) | (.601) | (.412) | (.977) | (.309) | (.010) | (.292) |
| income=4 | .136 | .266 | -.926 | .011 | .158 | .337 | .451 | -.566 | .134 |
|  | .298 | .219 | .426 | .257 | .230 | .253 | .220 | .228 | .206 |
|  | [-.448, .719] | [-.163, .695] | [-1.761, -.091] | [-.492, .515] | [-.294, .609] | [-.158, .832] | [.019, .883] | [-1.012, -.119] | [-.269, .537] |
|  | (.648) | (.225) | (.030) | (.965) | (.494) | (.182) | (.041) | (.013) | (.514) |
| income=5 | .270 | -.082 | -.896 | -.231 | -.078 | .247 | .530 | -.578 | -.001 |
|  | .304 | .227 | .448 | .264 | .232 | .255 | .228 | .231 | .216 |
|  | [-.327, .866] | [-.527, .363] | [-1.773, -.018] | [-.749, .287] | [-.533, .377] | [-.252, .746] | [.083, .978] | [-1.032, -.125] | [-.424, .422] |
|  | (.376) | (.717) | (.045) | (.383) | (.737) | (.331) | (.020) | (.012) | (.996) |
| income=6 | .350 | .020 | -.771 | -.038 | .224 | .463 | .309 | -.521 | .076 |
|  | .319 | .229 | .434 | .260 | .241 | .266 | .230 | .235 | .207 |
|  | [-.276, .976] | [-.429, .469] | [-1.622, .081] | [-.548, .471] | [-.248, .697] | [-.059, .985] | [-.142, .760] | [-.981, -.060] | [-.329, .481] |
|  | (.273) | (.930) | (.076) | (.883) | (.352) | (.082) | (.180) | (.027) | (.714) |
| income=7 | .195 | -.038 | -.714 | .550 | -.019 | .407 | .501 | -.679 | .164 |
|  | .336 | .244 | .423 | .269 | .254 | .279 | .245 | .256 | .227 |
|  | [-.463, .854] | [-.516, .441] | [-1.542, .115] | [.022, 1.078] | [-.517, .479] | [-.140, .955] | [.022, .981] | [-1.180, -.177] | [-.280, .608] |
|  | (.562) | (.878) | (.091) | (.041) | (.941) | (.145) | (.041) | (.008) | (.469) |
| income=8 | -.050 | -.354 | -1.482 | .292 | -.162 | .225 | .038 | -.356 | -.096 |
|  | .322 | .241 | .524 | .263 | .243 | .269 | .237 | .241 | .217 |
|  | [-.682, .581] | [-.825, .118] | [-2.508, -.455] | [-.222, .807] | [-.639, .315] | [-.302, .753] | [-.426, .502] | [-.829, .117] | [-.522, .330] |
|  | (.876) | (.142) | (.005) | (.266) | (.506) | (.402) | (.873) | (.140) | (.658) |
| income=9 | .720 | .242 | -1.326 | .271 | .063 | .233 | .482 | -.679 | .168 |
|  | .353 | .233 | .496 | .261 | .249 | .270 | .239 | .242 | .211 |
|  | [.028, 1.412] | [-.215, .698] | [-2.299, -.353] | [-.240, .782] | [-.425, .551] | [-.297, .763] | [.014, .951] | [-1.154, -.204] | [-.247, .582] |
|  | (.041) | (.299) | (.008) | (.299) | (.799) | (.388) | (.044) | (.005) | (.428) |
| income=10 | .270 | -.124 | -1.363 | .412 | .257 | .409 | .123 | -.647 | .058 |
|  | .341 | .246 | .496 | .268 | .256 | .280 | .247 | .253 | .223 |
|  | [-.399, .939] | [-.607, .359] | [-2.334, -.392] | [-.114, .938] | [-.245, .760] | [-.140, .957] | [-.360, .607] | [-1.143, -.151] | [-.379, .495] |
|  | (.429) | (.616) | (.006) | (.125) | (.316) | (.144) | (.617) | (.011) | (.794) |
| income=11 | .123 | .187 | -1.476 | .513 | .039 | .238 | .297 | -.495 | .235 |
|  | .347 | .250 | .517 | .272 | .259 | .284 | .251 | .252 | .230 |
|  | [-.557, .802] | [-.302, .676] | [-2.490, -.462] | [-.020, 1.045] | [-.469, .546] | [-.319, .795] | [-.195, .788] | [-.989, -.001] | [-.217, .686] |
|  | (.723) | (.453) | (.004) | (.059) | (.881) | (.403) | (.237) | (.050) | (.308) |
| income=12 | .823 | .195 | -1.163 | .505 | .301 | .336 | .540 | -.468 | .420 |
|  | .454 | .276 | .570 | .295 | .302 | .329 | .290 | .282 | .239 |
|  | [-.067, 1.713] | [-.345, .735] | [-2.281, -.045] | [-.073, 1.083] | [-.290, .892] | [-.309, .981] | [-.028, 1.108] | [-1.020, .084] | [-.048, .889] |
|  | (.070) | (.480) | (.041) | (.087) | (.318) | (.307) | (.062) | (.097) | (.079) |
| income=13 | .499 | .045 | -16.872 | .972 | .354 | .859 | 1.192 | -.276 | .655 |
|  | .601 | .388 | .422 | .406 | .429 | .543 | .441 | .387 | .412 |
|  | [-.679, 1.676] | [-.716, .806] | [-17.699, -16.044] | [.176, 1.767] | [-.487, 1.194] | [-.204, 1.923] | [.327, 2.057] | [-1.035, .482] | [-.152, 1.462] |
|  | (.407) | (.908) | (<.001) | (.017) | (.410) | (.113) | (.007) | (.475) | (.112) |
| income=14 | -.162 | .347 | -.670 | 1.428 | .577 | .704 | .295 | -.164 | .808 |
|  | .635 | .397 | .666 | .438 | .497 | .564 | .414 | .400 | .341 |
|  | [-1.406, 1.082] | [-.432, 1.126] | [-1.974, .635] | [.570, 2.287] | [-.397, 1.551] | [-.400, 1.809] | [-.517, 1.107] | [-.947, .620] | [.140, 1.476] |
|  | (.798) | (.383) | (.314) | (.001) | (.245) | (.211) | (.476) | (.682) | (.018) |
| income=15 | 14.846 | .753 | .476 | 1.182 | 15.104 | 13.397 | 14.721 | .915 | 1.793 |
|  | .706 | 1.256 | 1.216 | 1.489 | .791 | .808 | .840 | 1.635 | 1.926 |
|  | [13.463,16.229] | [-1.709, 3.215] | [-1.907, 2.859] | [-1.737, 4.100] | [13.554,16.655] | [11.814,14.981] | [13.075,16.368] | [-2.291, 4.120] | [-1.982, 5.568] |
|  | (<.001) | (.549) | (.695) | (.427) | (<.001) | (<.001) | (<.001) | (.576) | (.352) |
| income=16 | 15.091 | 1.741 | -.795 | .854 | 1.005 | .556 | .869 | -.499 | 1.037 |
|  | .429 | .661 | 1.186 | .535 | .839 | .832 | .673 | .569 | .384 |
|  | [14.249,15.932] | [.445, 3.037] | [-3.119, 1.530] | [-.195, 1.903] | [-.639, 2.650] | [-1.075, 2.188] | [-.450, 2.188] | [-1.614, .616] | [.284, 1.790] |
|  | (<.001) | (.008) | (.503) | (.110) | (.231) | (.504) | (.197) | (.381) | (.007) |
| income=17 | -.051 | -.044 | -1.802 | .096 | -.065 | .316 | .136 | -.443 | -.046 |
|  | .279 | .210 | .463 | .239 | .215 | .236 | .210 | .212 | .199 |
|  | [-.598, .496] | [-.455, .367] | [-2.710, -.894] | [-.372, .564] | [-.486, .356] | [-.146, .779] | [-.275, .547] | [-.860, -.027] | [-.436, .345] |
|  | (.854) | (.835) | (<.001) | (.687) | (.762) | (.180) | (.516) | (.037) | (.818) |
| Rural Code=2 | -.146 | -.137 | -.350 | .010 | .174 | .115 | -.027 | -.062 | .005 |
|  | .148 | .105 | .246 | .113 | .109 | .126 | .104 | .108 | .090 |
|  | [-.436, .143] | [-.344, .069] | [-.831, .132] | [-.210, .231] | [-.040, .388] | [-.133, .362] | [-.231, .177] | [-.274, .149] | [-.172, .182] |
|  | (.322) | (.193) | (.154) | (.926) | (.111) | (.364) | (.792) | (.562) | (.955) |
| Rural Code=3 | -.081 | -.136 | -.367 | .032 | .184 | -.151 | .002 | -.201 | -.075 |
|  | .199 | .144 | .383 | .152 | .148 | .165 | .140 | .151 | .118 |
|  | [-.470, .309] | [-.418, .146] | [-1.117, .383] | [-.265, .329] | [-.107, .475] | [-.474, .172] | [-.272, .277] | [-.496, .094] | [-.306, .157] |
|  | (.684) | (.345) | (.337) | (.834) | (.215) | (.359) | (.987) | (.182) | (.528) |
| Rural Code=4 | -.598 | .087 | .050 | -.243 | -.157 | -.010 | -.329 | -.576 | -.385 |
|  | .258 | .197 | .385 | .225 | .208 | .236 | .191 | .225 | .160 |
|  | [-1.104, -.092] | [-.299, .472] | [-.705, .804] | [-.684, .198] | [-.565, .251] | [-.473, .453] | [-.704, .046] | [-1.016, -.136] | [-.699, -.071] |
|  | (.020) | (.660) | (.897) | (.281) | (.452) | (.967) | (.086) | (.010) | (.016) |
| Rural Code=5 | -.346 | .007 | -15.674 | .654 | .363 | .440 | -.300 | -.605 | -.016 |
|  | .397 | .303 | .416 | .336 | .328 | .411 | .302 | .334 | .236 |
|  | [-1.124, .431] | [-.588, .601] | [-16.489, -14.859] | [-.005, 1.313] | [-.281, 1.006] | [-.366, 1.246] | [-.890, .291] | [-1.259, .049] | [-.478, .447] |
|  | (.383) | (.982) | (<.001) | (.052) | (.269) | (.284) | (.321) | (.070) | (.947) |
| Rural Code=6 | -.409 | .028 | -.987 | -.026 | .014 | -.248 | -.087 | -.033 | -.113 |
|  | .253 | .195 | .624 | .220 | .200 | .218 | .202 | .206 | .189 |
|  | [-.905, .087] | [-.353, .410] | [-2.210, .236] | [-.456, .405] | [-.378, .406] | [-.676, .180] | [-.482, .308] | [-.437, .371] | [-.484, .257] |
|  | (.106) | (.884) | (.114) | (.908) | (.945) | (.256) | (.667) | (.874) | (.549) |
| Rural Code=7 | -.762 | -.011 | .132 | .042 | -.360 | -.154 | -.122 | -.415 | -.322 |
|  | .337 | .274 | .561 | .305 | .273 | .296 | .300 | .314 | .228 |
|  | [-1.422, -.102] | [-.548, .527] | [-.968, 1.232] | [-.556, .641] | [-.894, .174] | [-.735, .427] | [-.709, .465] | [-1.031, .201] | [-.769, .125] |
|  | (.024) | (.969) | (.814) | (.890) | (.186) | (.603) | (.684) | (.187) | (.158) |
| Rural Code=8 | -.780 | -.110 | -15.512 | -1.268 | -.744 | -.518 | -.736 | -.955 | -.857 |
|  | .578 | .538 | .469 | .832 | .530 | .594 | .538 | .710 | .530 |
|  | [-1.913, .352] | [-1.164, .943] | [-16.432, -14.592] | [-2.898, .361] | [-1.783, .296] | [-1.682, .645] | [-1.790, .318] | [-2.348, .437] | [-1.896, .183] |
|  | (.177) | (.837) | (<.001) | (.127) | (.161) | (.383) | (.171) | (.179) | (.106) |
| Rural Code=9 | -.894 | -.493 | -.055 | -.239 | -.111 | -.077 | .273 | -.056 | -.153 |
|  | .555 | .479 | .983 | .620 | .440 | .511 | .462 | .456 | .437 |
|  | [-1.982, .193] | [-1.433, .446] | [-1.982, 1.871] | [-1.454, .975] | [-.973, .751] | [-1.079, .926] | [-.632, 1.178] | [-.949, .837] | [-1.010, .703] |
|  | (.107) | (.303) | (.955) | (.699) | (.801) | (.881) | (.554) | (.902) | (.726) |
| Alaska | 15.935 | .669 | -16.296 | 1.395 | -.051 | .980 | -.149 | .961 | .802 |
|  | .606 | .787 | .845 | .759 | .836 | 1.013 | .953 | .842 | .712 |
|  | [14.748,17.123] | [-.872, 2.211] | [-17.953, -14.640] | [-.092, 2.882] | [-1.689, 1.587] | [-1.006, 2.965] | [-2.017, 1.719] | [-.689, 2.611] | [-.594, 2.199] |
|  | (<.001) | (.395) | (<.001) | (.066) | (.951) | (.333) | (.876) | (.254) | (.260) |
| Arizona | .321 | -.835 | -.655 | -.082 | .084 | .055 | -.546 | .507 | -.226 |
|  | .482 | .408 | .831 | .444 | .410 | .414 | .427 | .449 | .371 |
|  | [-.623, 1.266] | [-1.635, -.034] | [-2.283, .973] | [-.952, .789] | [-.720, .888] | [-.757, .867] | [-1.384, .292] | [-.373, 1.386] | [-.954, .502] |
|  | (.505) | (.041) | (.430) | (.854) | (.838) | (.894) | (.202) | (.259) | (.543) |
| Arkansas | .837 | -.285 | -.514 | -.056 | .074 | -.052 | -.221 | .419 | .038 |
|  | .590 | .446 | .961 | .499 | .454 | .474 | .474 | .504 | .401 |
|  | [-.320, 1.993] | [-1.159, .590] | [-2.398, 1.370] | [-1.034, .922] | [-.816, .964] | [-.980, .876] | [-1.149, .708] | [-.570, 1.408] | [-.748, .823] |
|  | (.156) | (.523) | (.593) | (.911) | (.870) | (.912) | (.641) | (.407) | (.925) |
| California | .601 | -.106 | -.708 | .326 | .297 | .609 | -.210 | .852 | .352 |
|  | .426 | .345 | .677 | .378 | .358 | .363 | .382 | .399 | .324 |
|  | [-.233, 1.435] | [-.782, .570] | [-2.035, .619] | [-.415, 1.067] | [-.404, .999] | [-.103, 1.320] | [-.959, .540] | [.069, 1.634] | [-.283, .987] |
|  | (.158) | (.759) | (.295) | (.388) | (.406) | (.093) | (.583) | (.033) | (.277) |
| Colorado | 1.940 | .016 | -1.251 | .855 | .731 | .915 | .244 | .725 | .844 |
|  | .833 | .457 | 1.186 | .473 | .473 | .523 | .503 | .497 | .416 |
|  | [.307, 3.573] | [-.879, .911] | [-3.576, 1.074] | [-.073, 1.783] | [-.196, 1.658] | [-.111, 1.940] | [-.741, 1.230] | [-.249, 1.699] | [.028, 1.659] |
|  | (.020) | (.972) | (.292) | (.071) | (.122) | (.080) | (.627) | (.144) | (.043) |
| Connecticut | -.041 | -.086 | -16.164 | .209 | .971 | .759 | -.165 | 1.150 | .360 |
|  | .612 | .486 | .667 | .528 | .546 | .583 | .506 | .540 | .402 |
|  | [-1.240, 1.159] | [-1.039, .867] | [-17.472, -14.856] | [-.827, 1.244] | [-.100, 2.042] | [-.383, 1.902] | [-1.157, .826] | [.091, 2.208] | [-.428, 1.147] |
|  | (.947) | (.859) | (<.001) | (.693) | (.076) | (.193) | (.744) | (.033) | (.371) |
| Delaware | 15.333 | .943 | -16.119 | -.170 | 15.129 | .689 | .364 | 1.197 | 1.000 |
|  | .494 | .721 | .707 | .706 | .444 | .866 | .641 | .686 | .467 |
|  | [14.364,16.302] | [-.471, 2.357] | [-17.504, -14.734] | [-1.555, 1.214] | [14.260,15.999] | [-1.009, 2.387] | [-.892, 1.620] | [-.148, 2.541] | [.085, 1.914] |
|  | (<.001) | (.191) | (<.001) | (.809) | (<.001) | (.426) | (.570) | (.081) | (.032) |
| District of Columbia | .339 | -.845 | -16.408 | 1.452 | .897 | 1.222 | -.595 | 1.451 | .719 |
|  | 1.097 | .843 | .798 | .731 | .854 | 1.051 | .814 | .784 | .576 |
|  | [-1.811, 2.488] | [-2.498, .808] | [-17.973, -14.844] | [.020, 2.884] | [-.777, 2.572] | [-.838, 3.282] | [-2.191, 1.001] | [-.085, 2.986] | [-.409, 1.848] |
|  | (.757) | (.316) | (<.001) | (.047) | (.294) | (.245) | (.465) | (.064) | (.212) |
| Florida | .670 | .121 | -.809 | .029 | .427 | .413 | -.293 | .640 | .281 |
|  | .434 | .348 | .702 | .384 | .361 | .366 | .386 | .405 | .325 |
|  | [-.181, 1.520] | [-.561, .802] | [-2.186, .567] | [-.725, .782] | [-.281, 1.136] | [-.303, 1.130] | [-1.050, .464] | [-.153, 1.434] | [-.357, .919] |
|  | (.123) | (.729) | (.249) | (.940) | (.237) | (.258) | (.448) | (.114) | (.387) |
| Georgia | .383 | -.105 | -.797 | .730 | .400 | .616 | -.426 | .519 | .341 |
|  | .471 | .382 | .792 | .405 | .393 | .404 | .414 | .436 | .355 |
|  | [-.539, 1.306] | [-.854, .645] | [-2.349, .755] | [-.064, 1.525] | [-.370, 1.169] | [-.176, 1.407] | [-1.237, .384] | [-.335, 1.373] | [-.355, 1.037] |
|  | (.415) | (.784) | (.314) | (.072) | (.309) | (.128) | (.303) | (.234) | (.337) |
| Hawaii | .921 | .109 | .252 | -2.100 | -.385 | 1.451 | -.101 | .407 | -.157 |
|  | 1.200 | .706 | 1.321 | 1.107 | .682 | 1.028 | .732 | .742 | .495 |
|  | [-1.431, 3.273] | [-1.275, 1.493] | [-2.336, 2.841] | [-4.269, .069] | [-1.723, .953] | [-.563, 3.465] | [-1.536, 1.333] | [-1.047, 1.861] | [-1.128, .813] |
|  | (.443) | (.877) | (.848) | (.058) | (.573) | (.158) | (.890) | (.583) | (.751) |
| Idaho | -.496 | -.376 | -15.796 | .441 | -.374 | .037 | -1.326 | -.325 | -.646 |
|  | .620 | .586 | .684 | .571 | .584 | .584 | .620 | .718 | .664 |
|  | [-1.712, .721] | [-1.524, .772] | [-17.137, -14.456] | [-.679, 1.560] | [-1.519, .770] | [-1.108, 1.182] | [-2.541, -.111] | [-1.731, 1.082] | [-1.947, .655] |
|  | (.424) | (.521) | (<.001) | (.440) | (.522) | (.949) | (.032) | (.651) | (.330) |
| Illinois | .131 | -.607 | -.989 | .534 | .127 | .264 | -.404 | .907 | .121 |
|  | .453 | .383 | .818 | .407 | .388 | .394 | .411 | .425 | .350 |
|  | [-.758, 1.019] | [-1.358, .144] | [-2.593, .615] | [-.263, 1.332] | [-.633, .887] | [-.509, 1.037] | [-1.209, .402] | [.074, 1.739] | [-.564, .807] |
|  | (.773) | (.113) | (.227) | (.189) | (.743) | (.503) | (.326) | (.033) | (.729) |
| Indiana | .264 | -.555 | -.839 | .504 | .550 | .960 | -.615 | .864 | .254 |
|  | .489 | .434 | .952 | .449 | .434 | .476 | .444 | .457 | .379 |
|  | [-.695, 1.223] | [-1.406, .296] | [-2.705, 1.027] | [-.375, 1.383] | [-.301, 1.401] | [.026, 1.894] | [-1.486, .256] | [-.032, 1.760] | [-.488, .997] |
|  | (.589) | (.201) | (.378) | (.261) | (.205) | (.044) | (.166) | (.059) | (.502) |
| Iowa | 1.586 | .044 | .147 | .152 | -.250 | .525 | -.242 | -.357 | .046 |
|  | .837 | .490 | 1.040 | .567 | .493 | .566 | .522 | .606 | .412 |
|  | [-.054, 3.227] | [-.916, 1.005] | [-1.891, 2.185] | [-.960, 1.263] | [-1.216, .716] | [-.584, 1.634] | [-1.264, .780] | [-1.545, .831] | [-.762, .853] |
|  | (.058) | (.928) | (.888) | (.789) | (.611) | (.354) | (.642) | (.556) | (.912) |
| Kansas | 1.763 | -.753 | -16.290 | -.104 | .399 | .986 | -.288 | .481 | .074 |
|  | 1.079 | .659 | .681 | .676 | .594 | .723 | .609 | .649 | .478 |
|  | [-.351, 3.877] | [-2.046, .539] | [-17.624, -14.956] | [-1.429, 1.222] | [-.765, 1.564] | [-.431, 2.403] | [-1.482, .907] | [-.791, 1.753] | [-.863, 1.011] |
|  | (.102) | (.253) | (<.001) | (.878) | (.501) | (.173) | (.637) | (.459) | (.877) |
| Kentucky | 1.046 | -.491 | -.654 | -1.281 | .598 | .309 | -.244 | -.049 | -.063 |
|  | .634 | .460 | .937 | .660 | .484 | .497 | .485 | .535 | .396 |
|  | [-.196, 2.288] | [-1.393, .410] | [-2.491, 1.183] | [-2.574, .012] | [-.349, 1.546] | [-.664, 1.282] | [-1.194, .706] | [-1.098, .999] | [-.840, .714] |
|  | (.099) | (.285) | (.485) | (.052) | (.216) | (.533) | (.615) | (.927) | (.874) |
| Louisiana | .806 | -.429 | -1.625 | -.530 | .680 | .890 | -.180 | .504 | .097 |
|  | .611 | .462 | 1.159 | .544 | .479 | .516 | .480 | .511 | .366 |
|  | [-.391, 2.004] | [-1.334, .476] | [-3.897, .646] | [-1.596, .537] | [-.258, 1.618] | [-.122, 1.901] | [-1.120, .760] | [-.498, 1.506] | [-.621, .816] |
|  | (.187) | (.353) | (.161) | (.330) | (.155) | (.085) | (.707) | (.324) | (.790) |
| Maine | .993 | -1.058 | -15.973 | .872 | 1.219 | 1.339 | 1.641 | 1.054 | .917 |
|  | .819 | .733 | .716 | .689 | .722 | .776 | .840 | .674 | .522 |
|  | [-.613, 2.599] | [-2.495, .380] | [-17.376, -14.569] | [-.479, 2.224] | [-.195, 2.633] | [-.182, 2.860] | [-.005, 3.287] | [-.267, 2.375] | [-.105, 1.940] |
|  | (.226) | (.149) | (<.001) | (.206) | (.091) | (.085) | (.051) | (.118) | (.079) |
| Maryland | 1.039 | -.128 | -1.565 | .049 | .428 | .947 | -.134 | -.108 | .150 |
|  | .718 | .453 | 1.229 | .477 | .474 | .554 | .475 | .551 | .403 |
|  | [-.368, 2.445] | [-1.016, .760] | [-3.974, .843] | [-.887, .984] | [-.502, 1.357] | [-.139, 2.032] | [-1.066, .798] | [-1.187, .971] | [-.640, .940] |
|  | (.148) | (.777) | (.203) | (.919) | (.367) | (.087) | (.778) | (.844) | (.710) |
| Massachusetts | .488 | -.411 | -16.010 | .528 | .796 | 1.049 | -.818 | .815 | .223 |
|  | .532 | .437 | .662 | .448 | .451 | .492 | .454 | .469 | .367 |
|  | [-.554, 1.530] | [-1.267, .445] | [-17.308, -14.712] | [-.351, 1.407] | [-.087, 1.680] | [.085, 2.014] | [-1.708, .073] | [-.104, 1.735] | [-.496, .942] |
|  | (.359) | (.347) | (<.001) | (.239) | (.077) | (.033) | (.072) | (.082) | (.543) |
| Michigan | .940 | -.217 | -.249 | -.288 | .513 | .480 | -.518 | .429 | .068 |
|  | .490 | .375 | .734 | .417 | .389 | .402 | .405 | .434 | .337 |
|  | [-.020, 1.899] | [-.951, .518] | [-1.687, 1.189] | [-1.106, .530] | [-.249, 1.275] | [-.307, 1.268] | [-1.311, .275] | [-.422, 1.279] | [-.592, .729] |
|  | (.055) | (.563) | (.735) | (.490) | (.187) | (.232) | (.200) | (.323) | (.839) |
| Minnesota | .974 | -.528 | -1.517 | .368 | .488 | .528 | -.159 | .654 | .291 |
|  | .642 | .465 | 1.204 | .480 | .477 | .502 | .497 | .500 | .394 |
|  | [-.284, 2.232] | [-1.439, .383] | [-3.878, .843] | [-.572, 1.308] | [-.447, 1.424] | [-.456, 1.512] | [-1.133, .816] | [-.326, 1.633] | [-.482, 1.064] |
|  | (.129) | (.256) | (.208) | (.443) | (.306) | (.293) | (.750) | (.191) | (.460) |
| Mississippi | .645 | -.319 | -.295 | -.628 | .644 | .365 | .170 | 1.123 | .370 |
|  | .647 | .506 | .963 | .614 | .529 | .541 | .523 | .555 | .459 |
|  | [-.623, 1.913] | [-1.311, .674] | [-2.182, 1.592] | [-1.831, .575] | [-.393, 1.681] | [-.695, 1.424] | [-.855, 1.194] | [.035, 2.211] | [-.530, 1.270] |
|  | (.319) | (.529) | (.759) | (.306) | (.224) | (.500) | (.746) | (.043) | (.420) |
| Missouri | .084 | -.570 | -.553 | .014 | .154 | .839 | -.401 | .196 | -.090 |
|  | .476 | .420 | .872 | .451 | .416 | .456 | .437 | .467 | .355 |
|  | [-.849, 1.018] | [-1.393, .252] | [-2.262, 1.155] | [-.869, .898] | [-.662, .970] | [-.055, 1.733] | [-1.257, .455] | [-.718, 1.111] | [-.785, .604] |
|  | (.860) | (.174) | (.525) | (.975) | (.712) | (.066) | (.358) | (.674) | (.799) |
| Montana | 1.085 | -.343 | -15.490 | .398 | .341 | .656 | -.789 | .768 | .269 |
|  | .823 | .685 | .761 | .675 | .629 | .718 | .668 | .681 | .551 |
|  | [-.528, 2.698] | [-1.685, .999] | [-16.982, -13.998] | [-.926, 1.722] | [-.891, 1.574] | [-.751, 2.062] | [-2.099, .520] | [-.567, 2.102] | [-.811, 1.348] |
|  | (.188) | (.616) | (<.001) | (.556) | (.587) | (.361) | (.238) | (.260) | (.626) |
| Nebraska | .759 | -.118 | -16.023 | -.091 | .533 | .628 | -.126 | .081 | .145 |
|  | .865 | .648 | .729 | .824 | .751 | .746 | .718 | .768 | .693 |
|  | [-.937, 2.454] | [-1.388, 1.153] | [-17.452, -14.594] | [-1.706, 1.524] | [-.940, 2.005] | [-.833, 2.089] | [-1.532, 1.281] | [-1.424, 1.586] | [-1.213, 1.504] |
|  | (.380) | (.856) | (<.001) | (.912) | (.478) | (.400) | (.861) | (.916) | (.834) |
| Nevada | .505 | -.727 | -.781 | -.259 | 1.117 | .791 | -.174 | 1.103 | .269 |
|  | .601 | .458 | .966 | .512 | .510 | .507 | .485 | .490 | .410 |
|  | [-.673, 1.682] | [-1.625, .171] | [-2.673, 1.111] | [-1.264, .745] | [.118, 2.117] | [-.203, 1.786] | [-1.126, .777] | [.142, 2.064] | [-.535, 1.072] |
|  | (.401) | (.113) | (.419) | (.613) | (.028) | (.119) | (.719) | (.025) | (.512) |
| New Hampshire | 1.147 | -.877 | -.413 | -.403 | .110 | .328 | -.217 | -.061 | -.220 |
|  | .830 | .592 | 1.207 | .631 | .535 | .600 | .553 | .657 | .408 |
|  | [-.479, 2.773] | [-2.038, .283] | [-2.779, 1.953] | [-1.639, .833] | [-.940, 1.159] | [-.847, 1.504] | [-1.301, .867] | [-1.348, 1.225] | [-1.020, .580] |
|  | (.167) | (.138) | (.732) | (.523) | (.837) | (.584) | (.694) | (.925) | (.590) |
| New Jersey | 1.026 | .155 | -.660 | .284 | .616 | .798 | -.217 | .291 | .394 |
|  | .574 | .408 | .850 | .441 | .432 | .458 | .436 | .466 | .364 |
|  | [-.098, 2.150] | [-.645, .954] | [-2.325, 1.005] | [-.580, 1.148] | [-.230, 1.463] | [-.099, 1.695] | [-1.073, .638] | [-.623, 1.205] | [-.319, 1.107] |
|  | (.074) | (.704) | (.437) | (.520) | (.154) | (.081) | (.618) | (.533) | (.279) |
| New Mexico | .848 | -.536 | -1.018 | .246 | .568 | -.219 | .275 | .665 | .115 |
|  | .680 | .510 | 1.082 | .546 | .554 | .529 | .542 | .552 | .448 |
|  | [-.484, 2.180] | [-1.535, .464] | [-3.139, 1.104] | [-.825, 1.316] | [-.519, 1.654] | [-1.256, .818] | [-.788, 1.338] | [-.416, 1.746] | [-.763, .993] |
|  | (.212) | (.294) | (.347) | (.653) | (.306) | (.679) | (.612) | (.228) | (.797) |
| New York | .107 | .118 | -.405 | .255 | .417 | .771 | -.180 | .510 | .353 |
|  | .431 | .355 | .686 | .391 | .372 | .386 | .396 | .414 | .338 |
|  | [-.738, .952] | [-.578, .814] | [-1.750, .939] | [-.512, 1.022] | [-.313, 1.147] | [.014, 1.528] | [-.957, .596] | [-.301, 1.322] | [-.309, 1.015] |
|  | (.804) | (.740) | (.554) | (.514) | (.263) | (.046) | (.649) | (.217) | (.296) |
| North Carolina | .689 | .070 | -.387 | .282 | .484 | .684 | .023 | .286 | .351 |
|  | .491 | .385 | .787 | .421 | .400 | .427 | .425 | .449 | .366 |
|  | [-.273, 1.652] | [-.684, .825] | [-1.929, 1.155] | [-.543, 1.107] | [-.300, 1.268] | [-.153, 1.521] | [-.810, .856] | [-.595, 1.166] | [-.366, 1.067] |
|  | (.161) | (.855) | (.623) | (.503) | (.227) | (.109) | (.957) | (.525) | (.337) |
| North Dakota | .656 | 1.445 | -15.847 | -.171 | .036 | 1.308 | -.087 | .705 | .688 |
|  | 1.045 | .921 | .838 | .939 | .790 | 1.071 | .746 | .733 | .686 |
|  | [-1.392, 2.704] | [-.361, 3.251] | [-17.489, -14.205] | [-2.012, 1.669] | [-1.513, 1.585] | [-.791, 3.408] | [-1.548, 1.375] | [-.732, 2.142] | [-.656, 2.032] |
|  | (.530) | (.117) | (<.001) | (.855) | (.964) | (.222) | (.908) | (.336) | (.316) |
| Ohio | .778 | -.159 | -.659 | .274 | .434 | .814 | -.052 | .705 | .405 |
|  | .484 | .374 | .769 | .414 | .390 | .414 | .414 | .425 | .353 |
|  | [-.171, 1.727] | [-.893, .574] | [-2.167, .849] | [-.537, 1.085] | [-.330, 1.199] | [.002, 1.626] | [-.864, .760] | [-.128, 1.537] | [-.286, 1.097] |
|  | (.108) | (.670) | (.392) | (.508) | (.265) | (.049) | (.900) | (.097) | (.251) |
| Oklahoma | .388 | .340 | .362 | .409 | .674 | .555 | -.340 | .549 | .203 |
|  | .609 | .525 | .841 | .547 | .565 | .574 | .524 | .561 | .458 |
|  | [-.806, 1.582] | [-.688, 1.369] | [-1.285, 2.010] | [-.664, 1.482] | [-.434, 1.782] | [-.570, 1.679] | [-1.367, .687] | [-.550, 1.649] | [-.694, 1.100] |
|  | (.525) | (.517) | (.667) | (.455) | (.233) | (.334) | (.516) | (.328) | (.657) |
| Oregon | .074 | -.570 | -1.737 | .311 | .301 | .617 | -.193 | .711 | .170 |
|  | .521 | .426 | 1.219 | .448 | .428 | .474 | .456 | .464 | .389 |
|  | [-.948, 1.095] | [-1.406, .265] | [-4.125, .652] | [-.566, 1.189] | [-.538, 1.141] | [-.312, 1.545] | [-1.086, .700] | [-.198, 1.619] | [-.592, .931] |
|  | (.888) | (.181) | (.154) | (.486) | (.482) | (.193) | (.672) | (.125) | (.662) |
| Pennsylvania | .878 | -.496 | -.873 | .188 | .472 | .840 | -.370 | .527 | .174 |
|  | .446 | .357 | .730 | .388 | .369 | .380 | .390 | .411 | .325 |
|  | [.003, 1.753] | [-1.195, .204] | [-2.304, .558] | [-.572, .948] | [-.251, 1.195] | [.095, 1.586] | [-1.134, .394] | [-.279, 1.333] | [-.463, .811] |
|  | (.049) | (.165) | (.232) | (.628) | (.200) | (.027) | (.343) | (.200) | (.592) |
| Rhode Island | .786 | 1.317 | .538 | .759 | .959 | .546 | -.416 | .641 | .895 |
|  | 1.126 | .840 | 1.322 | .873 | .883 | .862 | .833 | .805 | 1.052 |
|  | [-1.420, 2.992] | [-.330, 2.965] | [-2.054, 3.130] | [-.953, 2.470] | [-.772, 2.690] | [-1.145, 2.236] | [-2.048, 1.216] | [-.936, 2.218] | [-1.167, 2.956] |
|  | (.485) | (.117) | (.684) | (.385) | (.278) | (.527) | (.617) | (.425) | (.395) |
| South Carolina | .683 | .038 | -1.507 | .196 | .891 | 1.090 | .146 | .657 | .609 |
|  | .564 | .434 | 1.218 | .489 | .477 | .522 | .476 | .481 | .410 |
|  | [-.422, 1.789] | [-.813, .888] | [-3.894, .881] | [-.763, 1.155] | [-.045, 1.827] | [.067, 2.113] | [-.788, 1.080] | [-.285, 1.599] | [-.195, 1.413] |
|  | (.226) | (.931) | (.216) | (.689) | (.062) | (.037) | (.759) | (.172) | (.138) |
| South Dakota | .085 | -1.862 | -15.683 | -.677 | -.583 | .520 | -.884 | -13.730 | -.881 |
|  | .885 | 1.100 | .798 | 1.013 | .748 | .836 | .817 | .536 | .620 |
|  | [-1.649, 1.819] | [-4.018, .293] | [-17.247, -14.119] | [-2.662, 1.308] | [-2.049, .884] | [-1.118, 2.158] | [-2.485, .717] | [-14.781, -12.680] | [-2.097, .335] |
|  | (.923) | (.090) | (<.001) | (.504) | (.436) | (.534) | (.279) | (<.001) | (.155) |
| Tennessee | 1.304 | .381 | -.674 | .230 | .476 | 1.383 | .345 | .651 | .834 |
|  | .641 | .423 | .959 | .471 | .444 | .552 | .470 | .480 | .399 |
|  | [.049, 2.560] | [-.448, 1.211] | [-2.554, 1.207] | [-.693, 1.154] | [-.394, 1.347] | [.302, 2.464] | [-.576, 1.267] | [-.290, 1.592] | [.052, 1.616] |
|  | (.042) | (.367) | (.483) | (.625) | (.284) | (.012) | (.462) | (.175) | (.037) |
| Texas | .334 | -.064 | -.525 | .201 | .079 | .211 | -.627 | .037 | -.100 |
|  | .429 | .353 | .686 | .391 | .363 | .369 | .391 | .416 | .335 |
|  | [-.507, 1.176] | [-.755, .628] | [-1.870, .819] | [-.565, .967] | [-.632, .790] | [-.511, .933] | [-1.394, .139] | [-.779, .853] | [-.757, .557] |
|  | (.436) | (.857) | (.444) | (.607) | (.828) | (.567) | (.109) | (.929) | (.766) |
| Utah | .441 | -.668 | -15.860 | .741 | .164 | .566 | -.183 | .545 | .171 |
|  | .619 | .545 | .694 | .517 | .508 | .548 | .517 | .551 | .440 |
|  | [-.771, 1.654] | [-1.736, .400] | [-17.219, -14.500] | [-.272, 1.753] | [-.831, 1.160] | [-.508, 1.640] | [-1.197, .831] | [-.535, 1.624] | [-.692, 1.034] |
|  | (.475) | (.221) | (<.001) | (.152) | (.746) | (.302) | (.723) | (.323) | (.698) |
| Vermont | 15.815 | -.598 | -15.986 | 1.033 | .897 | -.101 | .244 | -.891 | .243 |
|  | .540 | .756 | .760 | .778 | .916 | .854 | .687 | 1.144 | .530 |
|  | [14.756,16.874] | [-2.079, .884] | [-17.475, -14.497] | [-.491, 2.557] | [-.899, 2.693] | [-1.775, 1.573] | [-1.102, 1.590] | [-3.133, 1.350] | [-.795, 1.281] |
|  | (<.001) | (.429) | (<.001) | (.184) | (.328) | (.906) | (.722) | (.436) | (.646) |
| Virginia | .472 | .111 | -.752 | .228 | .211 | .760 | -.154 | .718 | .373 |
|  | .475 | .378 | .765 | .411 | .390 | .422 | .416 | .432 | .357 |
|  | [-.458, 1.403] | [-.630, .852] | [-2.251, .747] | [-.578, 1.034] | [-.553, .976] | [-.066, 1.587] | [-.970, .662] | [-.130, 1.565] | [-.327, 1.073] |
|  | (.320) | (.769) | (.326) | (.579) | (.588) | (.071) | (.712) | (.097) | (.296) |
| Washington | .887 | -.365 | -.026 | .312 | .718 | 1.062 | -.131 | .537 | .434 |
|  | .531 | .408 | .775 | .437 | .426 | .473 | .435 | .458 | .366 |
|  | [-.154, 1.929] | [-1.164, .434] | [-1.545, 1.494] | [-.546, 1.169] | [-.116, 1.552] | [.135, 1.989] | [-.983, .722] | [-.360, 1.435] | [-.283, 1.151] |
|  | (.095) | (.370) | (.973) | (.476) | (.092) | (.025) | (.764) | (.240) | (.235) |
| West Virginia | .046 | -.163 | -.371 | .321 | -.141 | .201 | -.658 | -.131 | -.235 |
|  | .636 | .539 | 1.254 | .595 | .532 | .585 | .530 | .617 | .489 |
|  | [-1.201, 1.294] | [-1.221, .894] | [-2.830, 2.088] | [-.846, 1.488] | [-1.184, .903] | [-.947, 1.348] | [-1.697, .381] | [-1.341, 1.078] | [-1.193, .723] |
|  | (.942) | (.762) | (.768) | (.590) | (.792) | (.732) | (.214) | (.832) | (.631) |
| Wisconsin | .521 | -.332 | -.285 | .507 | .379 | .705 | -.462 | .143 | .151 |
|  | .529 | .419 | .873 | .459 | .434 | .459 | .465 | .489 | .351 |
|  | [-.515, 1.557] | [-1.153, .488] | [-1.997, 1.426] | [-.392, 1.406] | [-.472, 1.231] | [-.194, 1.604] | [-1.374, .450] | [-.816, 1.103] | [-.537, .838] |
|  | (.324) | (.427) | (.744) | (.269) | (.383) | (.124) | (.321) | (.769) | (.668) |
| Wyoming | 16.128 | 1.041 | 1.764 | .223 | .636 | .330 | .674 | 2.219 | 1.266 |
|  | .761 | 1.420 | 1.156 | 1.100 | 1.173 | 1.158 | 1.192 | 1.336 | 1.697 |
|  | [14.637,17.619] | [-1.743, 3.824] | [-.501, 4.029] | [-1.932, 2.378] | [-1.663, 2.936] | [-1.939, 2.600] | [-1.663, 3.010] | [-.401, 4.838] | [-2.060, 4.592] |
|  | (<.001) | (.464) | (.127) | (.839) | (.588) | (.775) | (.572) | (.097) | (.456) |
| cut1 | -.216 | .379 | .478 | 1.885 | .260 | .818 | 1.129 | 1.246 | -1.633 |
|  | .530 | .434 | .744 | .500 | .453 | .471 | .457 | .474 | .413 |
|  | [-1.255, .822] | [-.472, 1.229] | [-.979, 1.936] | [.905, 2.864] | [-.627, 1.147] | [-.105, 1.741] | [.233, 2.025] | [.317, 2.174] | [-2.443, -.824] |
|  | (.683) | (.383) | (.520) | (<.001) | (.566) | (.082) | (.014) | (.009) | (<.001) |
| cut2 |  |  |  |  |  |  |  |  | -.844 |
|  |  |  |  |  |  |  |  |  | .409 |
|  |  |  |  |  |  |  |  |  | [-1.645, -.042] |
|  |  |  |  |  |  |  |  |  | (.039) |
| cut3 |  |  |  |  |  |  |  |  | -.201 |
|  |  |  |  |  |  |  |  |  | .407 |
|  |  |  |  |  |  |  |  |  | [-.999, .596] |
|  |  |  |  |  |  |  |  |  | (.621) |
| cut4 |  |  |  |  |  |  |  |  | .597 |
|  |  |  |  |  |  |  |  |  | .408 |
|  |  |  |  |  |  |  |  |  | [-.202, 1.396] |
|  |  |  |  |  |  |  |  |  | (.143) |
| cut5 |  |  |  |  |  |  |  |  | 1.468 |
|  |  |  |  |  |  |  |  |  | .408 |
|  |  |  |  |  |  |  |  |  | [.668, 2.269] |
|  |  |  |  |  |  |  |  |  | (<.001) |
| cut6 |  |  |  |  |  |  |  |  | 2.518 |
|  |  |  |  |  |  |  |  |  | .409 |
|  |  |  |  |  |  |  |  |  | [1.715, 3.320] |
|  |  |  |  |  |  |  |  |  | (<.001) |
| cut7 |  |  |  |  |  |  |  |  | 3.954 |
|  |  |  |  |  |  |  |  |  | .416 |
|  |  |  |  |  |  |  |  |  | [3.138, 4.770] |
|  |  |  |  |  |  |  |  |  | (<.001) |
| cut8 |  |  |  |  |  |  |  |  | 6.255 |
|  |  |  |  |  |  |  |  |  | .474 |
|  |  |  |  |  |  |  |  |  | [5.326,7.184] |
|  |  |  |  |  |  |  |  |  | (<.001) |
| N | 2982 | 2982 | 2982 | 2982 | 2982 | 2982 | 2982 | 2982 | 2982 |
| Pseudo-R^2^ | .0848 | .0473 | .104 | .0806 | .0564 | .0695 | .0724 | .0544 | .0305 |
| Model χ^2^ | 8081.5 | 171.0 | 20181.7 | 267.0 | 3103.0 | 497.0 | 562.5 | 1942.4 | 386.2 |
| d.f. | 87 | 87 | 87 | 87 | 87 | 87 | 87 | 87 | 87 |
| p-value | <.001 | <.001 | <.001 | <.001 | <.001 | <.001 | <.001 | <.001 | <.001 |

S4 Table. Regression Results, Health Views.

|  | (1) | (2) | (3) | (4) | (5) | (6) | (7) | (8) | (9) |
| --- | --- | --- | --- | --- | --- | --- | --- | --- | --- |
|  | numberdeaths | spendingincrease | notenoughtests | getsick | negativeeconomy | friendsick | cannotreturn | schoolsclosed | hardgetitems |
| Democrat | .586 | 1.684 | 1.604 | .983 | .708 | .962 | .801 | .456 | .587 |
|  | .091 | .114 | .095 | .090 | .095 | .090 | .091 | .089 | .093 |
|  | [.407, .766] | [1.461, 1.908] | [1.417, 1.791] | [.806, 1.160] | [.522, .895] | [.786, 1.138] | [.622, .980] | [.281, .631] | [.405, .768] |
|  | (<.001) | (<.001) | (<.001) | (<.001) | (<.001) | (<.001) | (<.001) | (<.001) | (<.001) |
| Other | .156 | .433 | .839 | .337 | .149 | .237 | .171 | .101 | .113 |
|  | .092 | .102 | .091 | .092 | .094 | .091 | .090 | .087 | .092 |
|  | [-.024, .335] | [.233, .632] | [.659, 1.018] | [.157, .517] | [-.035, .333] | [.059, .414] | [-.005, .347] | [-.070, .271] | [-.068, .295] |
|  | (.089) | (<.001) | (<.001) | (<.001) | (.113) | (.009) | (.057) | (.247) | (.219) |
| 35- | -.036 | .195 | .149 | .109 | -.125 | .126 | -.249 | .061 | .013 |
|  | .105 | .131 | .112 | .104 | .109 | .106 | .103 | .100 | .102 |
|  | [-.242, .169] | [-.061, .451] | [-.070, .369] | [-.094, .313] | [-.339, .090] | [-.082, .334] | [-.451, -.048] | [-.134, .257] | [-.187, .213] |
|  | (.730) | (.136) | (.183) | (.293) | (.255) | (.234) | (.015) | (.538) | (.898) |
| 50- | .310 | .108 | .221 | -.077 | .096 | -.032 | -.388 | -.173 | -.374 |
|  | .100 | .120 | .105 | .097 | .102 | .096 | .096 | .097 | .096 |
|  | [.114, .506] | [-.127, .342] | [.015, .428] | [-.267, .114] | [-.104, .297] | [-.221, .157] | [-.576, -.200] | [-.363, .017] | [-.564, -.185] |
|  | (.002) | (.369) | (.035) | (.429) | (.347) | (.740) | (<.001) | (.075) | (<.001) |
| 70- | .580 | .313 | .183 | .012 | -.103 | -.006 | -.513 | -.908 | -.789 |
|  | .121 | .151 | .121 | .117 | .124 | .116 | .117 | .117 | .120 |
|  | [.342, .818] | [.017, .608] | [-.055, .420] | [-.217, .240] | [-.347, .140] | [-.234, .222] | [-.742, -.284] | [-1.136, -.679] | [-1.025, -.553] |
|  | (<.001) | (.038) | (.132) | (.920) | (.404) | (.959) | (<.001) | (<.001) | (<.001) |
| gender | .079 | -.118 | -.007 | .269 | -.145 | .255 | .038 | .084 | .255 |
|  | .069 | .086 | .073 | .070 | .074 | .070 | .071 | .071 | .070 |
|  | [-.056, .215] | [-.286, .050] | [-.149, .136] | [.133, .406] | [-.289, .000] | [.118, .392] | [-.101, .177] | [-.055, .223] | [.118, .392] |
|  | (.252) | (.170) | (.925) | (<.001) | (.050) | (<.001) | (.596) | (.235) | (<.001) |
| white | -.049 | .304 | .459 | .040 | .203 | .013 | .244 | -.119 | .077 |
|  | .086 | .104 | .091 | .090 | .090 | .087 | .087 | .085 | .088 |
|  | [-.218, .120] | [.100, .507] | [.280, .638] | [-.137, .217] | [.027, .379] | [-.157, .184] | [.073, .415] | [-.284, .047] | [-.096, .251] |
|  | (.567) | (.003) | (<.001) | (.656) | (.024) | (.880) | (.005) | (.161) | (.382) |
| married | -.003 | -.050 | -.183 | .182 | .158 | .168 | .035 | .124 | .196 |
|  | .077 | .096 | .083 | .076 | .080 | .077 | .076 | .076 | .077 |
|  | [-.154, .149] | [-.237, .137] | [-.345, -.021] | [.033, .330] | [.001, .315] | [.016, .319] | [-.114, .185] | [-.026, .273] | [.046, .347] |
|  | (.973) | (.601) | (.027) | (.017) | (.048) | (.030) | (.642) | (.105) | (.011) |
| High school graduate | -.001 | -.346 | .006 | -.106 | -.210 | -.187 | -.111 | .037 | -.430 |
|  | .268 | .277 | .203 | .246 | .206 | .210 | .224 | .229 | .223 |
|  | [-.526, .524] | [-.889, .196] | [-.392, .403] | [-.588, .377] | [-.614, .194] | [-.598, .225] | [-.549, .327] | [-.411, .486] | [-.866, .006] |
|  | (.996) | (.211) | (.978) | (.668) | (.309) | (.374) | (.620) | (.870) | (.053) |
| Some college | -.063 | -.038 | .442 | -.024 | -.012 | -.153 | -.098 | -.019 | -.618 |
|  | .267 | .284 | .208 | .250 | .212 | .214 | .229 | .233 | .226 |
|  | [-.586, .460] | [-.593, .518] | [.033, .850] | [-.515, .467] | [-.427, .403] | [-.573, .267] | [-.547, .352] | [-.475, .437] | [-1.061, -.176] |
|  | (.813) | (.894) | (.034) | (.924) | (.955) | (.474) | (.669) | (.935) | (.006) |
| 2-year | .016 | -.005 | .312 | -.016 | .018 | -.129 | -.143 | -.020 | -.678 |
|  | .277 | .295 | .221 | .255 | .220 | .224 | .239 | .242 | .237 |
|  | [-.527, .560] | [-.584, .574] | [-.121, .744] | [-.517, .484] | [-.413, .450] | [-.567, .309] | [-.611, .325] | [-.495, .454] | [-1.143, -.213] |
|  | (.953) | (.987) | (.158) | (.950) | (.934) | (.564) | (.550) | (.934) | (.004) |
| 4-year | -.059 | .050 | .332 | -.078 | .057 | -.085 | -.063 | -.096 | -.603 |
|  | .269 | .288 | .213 | .252 | .217 | .217 | .229 | .235 | .229 |
|  | [-.586, .468] | [-.515, .614] | [-.085, .749] | [-.572, .416] | [-.367, .481] | [-.511, .341] | [-.511, .386] | [-.557, .365] | [-1.052, -.154] |
|  | (.826) | (.863) | (.119) | (.757) | (.793) | (.695) | (.783) | (.683) | (.008) |
| Post-grad | -.020 | .145 | .482 | .103 | .222 | .039 | .019 | .001 | -.800 |
|  | .279 | .300 | .231 | .262 | .228 | .224 | .238 | .247 | .240 |
|  | [-.567, .526] | [-.443, .733] | [.029, .935] | [-.410, .617] | [-.224, .669] | [-.400, .478] | [-.448, .486] | [-.483, .484] | [-1.271, -.330] |
|  | (.942) | (.629) | (.037) | (.694) | (.329) | (.863) | (.935) | (.997) | (.001) |
| income=2 | .129 | -.199 | .606 | -.178 | .007 | -.032 | -.332 | -.257 | -.271 |
|  | .243 | .240 | .205 | .212 | .206 | .213 | .215 | .198 | .230 |
|  | [-.348, .606] | [-.669, .271] | [.205, 1.007] | [-.594, .237] | [-.397, .411] | [-.449, .385] | [-.755, .090] | [-.645, .132] | [-.722, .181] |
|  | (.597) | (.406) | (.003) | (.401) | (.972) | (.879) | (.123) | (.195) | (.240) |
| income=3 | .294 | .017 | .529 | -.228 | .209 | -.078 | -.263 | -.112 | -.339 |
|  | .233 | .237 | .197 | .207 | .203 | .212 | .207 | .203 | .226 |
|  | [-.163, .751] | [-.447, .482] | [.142, .917] | [-.633, .177] | [-.190, .607] | [-.493, .338] | [-.669, .143] | [-.510, .286] | [-.782, .104] |
|  | (.207) | (.942) | (.007) | (.270) | (.305) | (.714) | (.204) | (.581) | (.134) |
| income=4 | .233 | .162 | .525 | -.025 | .357 | .141 | -.193 | -.112 | -.264 |
|  | .234 | .246 | .207 | .207 | .212 | .208 | .211 | .199 | .226 |
|  | [-.226, .692] | [-.321, .644] | [.119, .930] | [-.430, .380] | [-.058, .772] | [-.267, .550] | [-.606, .220] | [-.502, .279] | [-.707, .179] |
|  | (.320) | (.511) | (.011) | (.904) | (.092) | (.498) | (.359) | (.575) | (.243) |
| income=5 | .346 | .125 | .614 | -.174 | .264 | -.051 | -.354 | -.087 | -.423 |
|  | .236 | .249 | .215 | .213 | .213 | .210 | .210 | .194 | .225 |
|  | [-.115, .808] | [-.363, .613] | [.193, 1.034] | [-.592, .244] | [-.153, .682] | [-.464, .361] | [-.766, .057] | [-.466, .293] | [-.864, .018] |
|  | (.141) | (.616) | (.004) | (.414) | (.215) | (.808) | (.092) | (.655) | (.060) |
| income=6 | .395 | .070 | .617 | -.031 | .269 | -.040 | -.227 | .144 | -.319 |
|  | .241 | .257 | .213 | .214 | .218 | .214 | .218 | .204 | .233 |
|  | [-.077, .868] | [-.434, .573] | [.200, 1.034] | [-.450, .388] | [-.158, .697] | [-.458, .379] | [-.655, .201] | [-.255, .543] | [-.776, .137] |
|  | (.101) | (.786) | (.004) | (.886) | (.217) | (.853) | (.299) | (.480) | (.170) |
| income=7 | .205 | .032 | .631 | -.227 | .115 | -.093 | -.415 | -.177 | -.595 |
|  | .246 | .268 | .220 | .219 | .225 | .220 | .228 | .228 | .245 |
|  | [-.278, .687] | [-.494, .557] | [.200, 1.061] | [-.657, .202] | [-.326, .556] | [-.524, .339] | [-.862, .031] | [-.623, .269] | [-1.076, -.114] |
|  | (.406) | (.906) | (.004) | (.299) | (.608) | (.674) | (.068) | (.436) | (.015) |
| income=8 | .220 | .087 | .537 | -.552 | .022 | -.461 | -.534 | -.330 | -.760 |
|  | .243 | .264 | .224 | .222 | .221 | .217 | .222 | .215 | .239 |
|  | [-.257, .698] | [-.431, .604] | [.098, .976] | [-.987, -.117] | [-.411, .456] | [-.886, -.036] | [-.970, -.098] | [-.751, .092] | [-1.230, -.291] |
|  | (.365) | (.743) | (.017) | (.013) | (.919) | (.034) | (.016) | (.125) | (.002) |
| income=9 | .612 | .098 | .776 | -.397 | .273 | -.190 | -.203 | -.182 | -.635 |
|  | .240 | .259 | .223 | .214 | .220 | .215 | .218 | .212 | .228 |
|  | [.141, 1.083] | [-.410, .606] | [.338, 1.213] | [-.815, .022] | [-.159, .705] | [-.611, .231] | [-.630, .224] | [-.597, .233] | [-1.082, -.189] |
|  | (.011) | (.705) | (.001) | (.063) | (.215) | (.376) | (.351) | (.390) | (.005) |
| income=10 | .463 | .025 | .846 | -.381 | .414 | -.187 | -.254 | -.212 | -.735 |
|  | .238 | .268 | .234 | .216 | .235 | .221 | .223 | .212 | .245 |
|  | [-.003, .929] | [-.501, .551] | [.387, 1.305] | [-.804, .042] | [-.047, .875] | [-.620, .247] | [-.692, .184] | [-.627, .203] | [-1.215, -.256] |
|  | (.052) | (.925) | (<.001) | (.078) | (.078) | (.399) | (.256) | (.317) | (.003) |
| income=11 | .702 | .030 | .770 | -.202 | .850 | -.047 | .065 | -.187 | -.717 |
|  | .245 | .273 | .238 | .232 | .254 | .235 | .235 | .219 | .248 |
|  | [.223, 1.182] | [-.506, .565] | [.303, 1.238] | [-.657, .252] | [.352, 1.349] | [-.507, .413] | [-.395, .525] | [-.617, .242] | [-1.203, -.230] |
|  | (.004) | (.913) | (.001) | (.383) | (.001) | (.841) | (.782) | (.393) | (.004) |
| income=12 | .435 | .209 | .735 | -.311 | .468 | -.031 | -.015 | -.375 | -.904 |
|  | .259 | .308 | .292 | .249 | .264 | .280 | .265 | .261 | .288 |
|  | [-.074, .943] | [-.394, .812] | [.164, 1.307] | [-.799, .177] | [-.048, .985] | [-.580, .517] | [-.535, .504] | [-.887, .137] | [-1.468, -.340] |
|  | (.094) | (.497) | (.012) | (.212) | (.076) | (.911) | (.954) | (.151) | (.002) |
| income=13 | .413 | .466 | 1.030 | -.532 | .101 | -.301 | -.185 | -.767 | -1.238 |
|  | .371 | .465 | .366 | .296 | .366 | .313 | .325 | .390 | .355 |
|  | [-.313, 1.140] | [-.445, 1.377] | [.313, 1.747] | [-1.111, .048] | [-.617, .818] | [-.914, .312] | [-.823, .452] | [-1.530, -.004] | [-1.935, -.541] |
|  | (.265) | (.316) | (.005) | (.072) | (.783) | (.335) | (.569) | (.049) | (<.001) |
| income=14 | .631 | -.434 | .786 | -.479 | .262 | -.168 | -.322 | -.141 | -.907 |
|  | .323 | .419 | .323 | .413 | .422 | .420 | .435 | .312 | .368 |
|  | [-.003, 1.265] | [-1.255, .386] | [.153, 1.418] | [-1.289, .331] | [-.564, 1.089] | [-.990, .654] | [-1.175, .531] | [-.753, .471] | [-1.628, -.185] |
|  | (.051) | (.300) | (.015) | (.247) | (.534) | (.689) | (.460) | (.652) | (.014) |
| income=15 | .451 | 14.105 | 14.516 | .276 | 12.696 | -.007 | .248 | -.246 | -2.023 |
|  | 1.658 | .833 | .806 | 1.065 | .706 | 1.436 | .887 | 1.067 | 1.003 |
|  | [-2.798, 3.701] | [12.473,15.737] | [12.937,16.095] | [-1.811, 2.362] | [11.311,14.080] | [-2.822, 2.808] | [-1.490, 1.986] | [-2.336, 1.844] | [-3.988, -.057] |
|  | (.785) | (<.001) | (<.001) | (.796) | (<.001) | (.996) | (.780) | (.818) | (.044) |
| income=16 | .933 | .185 | 1.732 | -.606 | .409 | -.161 | .361 | -.068 | -.930 |
|  | .407 | .649 | .673 | .624 | .627 | .732 | .578 | .530 | .480 |
|  | [.135, 1.731] | [-1.088, 1.458] | [.412, 3.052] | [-1.829, .617] | [-.821, 1.639] | [-1.597, 1.274] | [-.772, 1.494] | [-1.107, .970] | [-1.871, .012] |
|  | (.022) | (.776) | (.010) | (.332) | (.515) | (.826) | (.532) | (.898) | (.053) |
| income=17 | .515 | -.062 | .562 | -.469 | .075 | -.284 | -.478 | -.454 | -.840 |
|  | .226 | .230 | .192 | .201 | .203 | .203 | .205 | .192 | .219 |
|  | [.073, .958] | [-.512, .388] | [.187, .938] | [-.863, -.074] | [-.322, .472] | [-.681, .114] | [-.879, -.077] | [-.831, -.077] | [-1.268, -.412] |
|  | (.022) | (.789) | (.003) | (.020) | (.710) | (.162) | (.020) | (.018) | (<.001) |
| Rural Code=2 | .015 | -.078 | -.121 | -.011 | -.110 | -.122 | -.094 | -.163 | -.016 |
|  | .093 | .114 | .096 | .096 | .096 | .093 | .093 | .091 | .096 |
|  | [-.166, .197] | [-.301, .145] | [-.308, .066] | [-.198, .177] | [-.297, .078] | [-.304, .060] | [-.275, .087] | [-.342, .016] | [-.205, .173] |
|  | (.870) | (.492) | (.206) | (.910) | (.252) | (.188) | (.310) | (.074) | (.869) |
| Rural Code=3 | -.004 | -.132 | -.209 | .034 | -.042 | .086 | .017 | .036 | .124 |
|  | .127 | .151 | .125 | .129 | .131 | .125 | .122 | .126 | .119 |
|  | [-.253, .245] | [-.429, .164] | [-.454, .037] | [-.218, .286] | [-.299, .215] | [-.159, .331] | [-.222, .255] | [-.211, .284] | [-.110, .357] |
|  | (.973) | (.382) | (.096) | (.792) | (.749) | (.492) | (.891) | (.774) | (.299) |
| Rural Code=4 | -.115 | .053 | .122 | -.250 | -.453 | -.326 | -.280 | -.160 | -.076 |
|  | .174 | .229 | .186 | .159 | .170 | .174 | .169 | .181 | .169 |
|  | [-.457, .226] | [-.395, .502] | [-.242, .486] | [-.562, .063] | [-.786, -.120] | [-.668, .016] | [-.612, .052] | [-.516, .196] | [-.407, .256] |
|  | (.509) | (.816) | (.512) | (.117) | (.008) | (.061) | (.099) | (.378) | (.654) |
| Rural Code=5 | -.220 | -.099 | -.057 | -.271 | .044 | .079 | -.203 | -.016 | .003 |
|  | .297 | .306 | .248 | .241 | .293 | .243 | .233 | .285 | .248 |
|  | [-.803, .363] | [-.698, .501] | [-.543, .430] | [-.742, .201] | [-.531, .619] | [-.396, .555] | [-.659, .253] | [-.574, .542] | [-.483, .489] |
|  | (.460) | (.747) | (.819) | (.260) | (.881) | (.744) | (.384) | (.955) | (.991) |
| Rural Code=6 | .252 | -.099 | -.096 | .006 | .010 | -.153 | -.002 | -.009 | .202 |
|  | .195 | .212 | .180 | .183 | .168 | .184 | .182 | .182 | .188 |
|  | [-.130, .635] | [-.513, .316] | [-.449, .258] | [-.352, .363] | [-.319, .339] | [-.514, .208] | [-.358, .355] | [-.365, .347] | [-.167, .571] |
|  | (.196) | (.641) | (.596) | (.976) | (.953) | (.407) | (.992) | (.961) | (.283) |
| Rural Code=7 | .067 | -.259 | .031 | .096 | -.223 | .124 | .062 | -.078 | .097 |
|  | .246 | .273 | .251 | .239 | .255 | .252 | .251 | .261 | .224 |
|  | [-.416, .550] | [-.794, .276] | [-.460, .522] | [-.372, .565] | [-.724, .277] | [-.371, .618] | [-.429, .554] | [-.590, .433] | [-.342, .535] |
|  | (.786) | (.342) | (.901) | (.686) | (.382) | (.624) | (.803) | (.764) | (.666) |
| Rural Code=8 | .892 | -.230 | -.384 | -.736 | -.031 | -.337 | -.226 | -.837 | .121 |
|  | .440 | .508 | .416 | .434 | .407 | .417 | .358 | .480 | .301 |
|  | [.030, 1.754] | [-1.226, .767] | [-1.198, .431] | [-1.586, .115] | [-.828, .767] | [-1.155, .481] | [-.928, .476] | [-1.778, .105] | [-.469, .712] |
|  | (.043) | (.652) | (.356) | (.090) | (.940) | (.420) | (.529) | (.082) | (.687) |
| Rural Code=9 | -.059 | -.281 | -.006 | .430 | -.219 | .442 | -.050 | -.111 | -.447 |
|  | .324 | .462 | .347 | .377 | .365 | .355 | .335 | .364 | .461 |
|  | [-.695, .577] | [-1.187, .624] | [-.685, .674] | [-.308, 1.169] | [-.934, .496] | [-.254, 1.138] | [-.707, .607] | [-.824, .603] | [-1.350, .456] |
|  | (.856) | (.543) | (.987) | (.253) | (.548) | (.213) | (.881) | (.761) | (.332) |
| Alaska | .417 | .220 | .551 | .043 | -.386 | -.825 | -.914 | .199 | -.402 |
|  | .713 | 1.130 | .686 | .569 | .637 | .476 | .654 | .676 | .596 |
|  | [-.981, 1.815] | [-1.994, 2.435] | [-.793, 1.894] | [-1.074, 1.159] | [-1.634, .863] | [-1.759, .108] | [-2.196, .367] | [-1.126, 1.524] | [-1.571, .767] |
|  | (.559) | (.845) | (.422) | (.940) | (.545) | (.083) | (.162) | (.768) | (.501) |
| Arizona | -.250 | .298 | .198 | -.035 | -.206 | -.418 | -.305 | -.277 | .022 |
|  | .371 | .436 | .382 | .372 | .360 | .346 | .370 | .328 | .337 |
|  | [-.977, .478] | [-.556, 1.152] | [-.550, .946] | [-.764, .693] | [-.911, .500] | [-1.097, .260] | [-1.030, .419] | [-.919, .365] | [-.639, .683] |
|  | (.501) | (.494) | (.603) | (.924) | (.568) | (.227) | (.409) | (.398) | (.948) |
| Arkansas | .175 | .271 | -.053 | .266 | -.465 | -.072 | .056 | .090 | .438 |
|  | .412 | .507 | .430 | .387 | .412 | .364 | .422 | .366 | .384 |
|  | [-.633, .983] | [-.723, 1.264] | [-.895, .790] | [-.493, 1.026] | [-1.272, .342] | [-.786, .642] | [-.771, .883] | [-.626, .807] | [-.315, 1.190] |
|  | (.671) | (.593) | (.903) | (.492) | (.259) | (.843) | (.894) | (.805) | (.254) |
| California | -.048 | .507 | .060 | .374 | -.283 | -.098 | -.081 | .030 | .213 |
|  | .338 | .391 | .343 | .326 | .299 | .300 | .329 | .291 | .297 |
|  | [-.711, .615] | [-.260, 1.273] | [-.612, .733] | [-.264, 1.013] | [-.869, .302] | [-.686, .491] | [-.725, .563] | [-.541, .601] | [-.369, .795] |
|  | (.887) | (.195) | (.860) | (.250) | (.343) | (.745) | (.806) | (.917) | (.473) |
| Colorado | .436 | .034 | -.099 | .312 | -.746 | -.303 | -.414 | -.081 | -.330 |
|  | .420 | .498 | .443 | .403 | .383 | .365 | .410 | .342 | .389 |
|  | [-.387, 1.258] | [-.942, 1.010] | [-.967, .770] | [-.478, 1.101] | [-1.497, .005] | [-1.019, .413] | [-1.218, .390] | [-.751, .590] | [-1.092, .432] |
|  | (.300) | (.945) | (.824) | (.439) | (.052) | (.407) | (.313) | (.813) | (.396) |
| Connecticut | .052 | 1.822 | .514 | .438 | -.037 | -.084 | .238 | .024 | .009 |
|  | .433 | .743 | .425 | .428 | .439 | .357 | .490 | .420 | .417 |
|  | [-.797, .902] | [.366, 3.278] | [-.319, 1.347] | [-.402, 1.277] | [-.898, .825] | [-.785, .616] | [-.722, 1.198] | [-.799, .847] | [-.809, .827] |
|  | (.904) | (.014) | (.227) | (.307) | (.933) | (.813) | (.627) | (.955) | (.983) |
| Delaware | .685 | -.322 | -.624 | .236 | -.328 | -.783 | -.373 | -.001 | -.614 |
|  | .498 | .687 | .519 | .503 | .496 | .517 | .464 | .533 | .522 |
|  | [-.290, 1.660] | [-1.670, 1.025] | [-1.641, .394] | [-.750, 1.222] | [-1.300, .644] | [-1.796, .230] | [-1.283, .538] | [-1.045, 1.043] | [-1.637, .410] |
|  | (.169) | (.639) | (.230) | (.639) | (.508) | (.130) | (.422) | (.999) | (.240) |
| District of Columbia | .320 | .364 | .302 | .623 | -.371 | -.180 | .042 | -.320 | .630 |
|  | 1.033 | .776 | .704 | .498 | .675 | .347 | .671 | .433 | .539 |
|  | [-1.705, 2.345] | [-1.157, 1.886] | [-1.078, 1.682] | [-.352, 1.598] | [-1.693, .951] | [-.861, .501] | [-1.273, 1.357] | [-1.169, .530] | [-.426, 1.686] |
|  | (.757) | (.639) | (.668) | (.210) | (.582) | (.605) | (.950) | (.461) | (.243) |
| Florida | .301 | .297 | .167 | .252 | -.390 | -.313 | -.061 | .019 | .135 |
|  | .339 | .386 | .344 | .327 | .306 | .299 | .330 | .304 | .303 |
|  | [-.362, .965] | [-.461, 1.054] | [-.506, .841] | [-.388, .892] | [-.990, .210] | [-.899, .274] | [-.709, .586] | [-.577, .614] | [-.459, .728] |
|  | (.374) | (.442) | (.626) | (.440) | (.203) | (.296) | (.853) | (.951) | (.657) |
| Georgia | .058 | .310 | .221 | -.012 | -.634 | -.393 | .071 | -.268 | -.015 |
|  | .395 | .422 | .368 | .366 | .341 | .339 | .361 | .326 | .331 |
|  | [-.716, .832] | [-.516, 1.136] | [-.501, .942] | [-.730, .705] | [-1.302, .035] | [-1.057, .270] | [-.637, .779] | [-.907, .371] | [-.664, .634] |
|  | (.883) | (.462) | (.549) | (.974) | (.063) | (.246) | (.844) | (.411) | (.963) |
| Hawaii | .025 | 14.342 | 1.228 | .013 | .258 | -.750 | .245 | -.172 | .584 |
|  | .636 | .506 | .809 | .657 | .643 | .499 | .590 | .669 | .700 |
|  | [-1.222, 1.272] | [13.350,15.334] | [-.357, 2.813] | [-1.274, 1.300] | [-1.002, 1.518] | [-1.729, .229] | [-.912, 1.402] | [-1.483, 1.139] | [-.788, 1.956] |
|  | (.969) | (<.001) | (.129) | (.984) | (.688) | (.133) | (.678) | (.797) | (.404) |
| Idaho | .113 | -.249 | -.337 | -.736 | -.421 | -1.050 | -.605 | -.332 | -.127 |
|  | .506 | .667 | .599 | .618 | .478 | .574 | .442 | .502 | .478 |
|  | [-.878, 1.105] | [-1.556, 1.057] | [-1.511, .837] | [-1.946, .474] | [-1.357, .516] | [-2.175, .074] | [-1.471, .261] | [-1.315, .651] | [-1.063, .810] |
|  | (.823) | (.708) | (.574) | (.233) | (.379) | (.067) | (.171) | (.508) | (.791) |
| Illinois | .102 | .495 | -.026 | .637 | -.505 | -.259 | -.121 | -.069 | .085 |
|  | .361 | .426 | .368 | .347 | .343 | .332 | .358 | .325 | .319 |
|  | [-.606, .810] | [-.340, 1.331] | [-.747, .694] | [-.044, 1.317] | [-1.176, .167] | [-.910, .392] | [-.823, .582] | [-.706, .569] | [-.540, .710] |
|  | (.778) | (.245) | (.943) | (.067) | (.141) | (.436) | (.736) | (.833) | (.790) |
| Indiana | -.311 | .295 | .550 | .569 | -.428 | -.084 | -.044 | -.103 | -.042 |
|  | .377 | .424 | .404 | .374 | .354 | .363 | .385 | .370 | .352 |
|  | [-1.050, .429] | [-.537, 1.127] | [-.243, 1.342] | [-.164, 1.302] | [-1.122, .266] | [-.796, .628] | [-.799, .711] | [-.827, .622] | [-.732, .649] |
|  | (.410) | (.487) | (.174) | (.128) | (.226) | (.817) | (.910) | (.781) | (.906) |
| Iowa | -.012 | -.150 | -.108 | .850 | -.092 | -.115 | -.149 | .113 | -.068 |
|  | .487 | .500 | .465 | .435 | .415 | .420 | .414 | .456 | .414 |
|  | [-.966, .942] | [-1.130, .831] | [-1.020, .803] | [-.003, 1.703] | [-.905, .722] | [-.939, .709] | [-.961, .663] | [-.782, 1.007] | [-.880, .743] |
|  | (.980) | (.765) | (.816) | (.051) | (.825) | (.784) | (.719) | (.805) | (.869) |
| Kansas | .266 | .815 | .252 | .392 | .719 | -.215 | .270 | .563 | .069 |
|  | .487 | .728 | .556 | .598 | .675 | .570 | .557 | .532 | .472 |
|  | [-.688, 1.221] | [-.612, 2.241] | [-.837, 1.342] | [-.781, 1.565] | [-.603, 2.042] | [-1.331, .902] | [-.822, 1.363] | [-.479, 1.606] | [-.856, .994] |
|  | (.584) | (.263) | (.650) | (.512) | (.286) | (.706) | (.628) | (.290) | (.884) |
| Kentucky | -.384 | -.052 | -.107 | .278 | .154 | -.411 | -.092 | -.121 | -.108 |
|  | .416 | .478 | .404 | .443 | .407 | .374 | .412 | .412 | .360 |
|  | [-1.199, .431] | [-.989, .885] | [-.899, .684] | [-.590, 1.146] | [-.644, .951] | [-1.143, .322] | [-.899, .715] | [-.928, .686] | [-.814, .597] |
|  | (.355) | (.913) | (.791) | (.531) | (.706) | (.272) | (.823) | (.769) | (.764) |
| Louisiana | .732 | .239 | -.019 | -.079 | -.260 | -.568 | .059 | .011 | .151 |
|  | .495 | .510 | .431 | .496 | .447 | .449 | .439 | .399 | .412 |
|  | [-.238, 1.703] | [-.761, 1.238] | [-.864, .826] | [-1.052, .894] | [-1.135, .615] | [-1.449, .312] | [-.801, .919] | [-.771, .792] | [-.657, .958] |
|  | (.139) | (.640) | (.965) | (.873) | (.561) | (.206) | (.893) | (.979) | (.715) |
| Maine | -.477 | .567 | -.679 | .260 | -.395 | -.353 | -.638 | -.774 | -1.596 |
|  | .489 | .721 | .657 | .585 | .500 | .468 | .629 | .536 | .590 |
|  | [-1.435, .481] | [-.847, 1.981] | [-1.967, .609] | [-.887, 1.407] | [-1.375, .585] | [-1.270, .565] | [-1.871, .596] | [-1.824, .276] | [-2.752, -.440] |
|  | (.329) | (.432) | (.301) | (.657) | (.429) | (.451) | (.311) | (.148) | (.007) |
| Maryland | -.150 | .769 | -.162 | .223 | -.255 | .042 | -.149 | .063 | -.132 |
|  | .389 | .555 | .460 | .410 | .393 | .395 | .403 | .395 | .415 |
|  | [-.913, .613] | [-.319, 1.857] | [-1.064, .740] | [-.580, 1.026] | [-1.024, .515] | [-.732, .815] | [-.940, .642] | [-.711, .838] | [-.946, .682] |
|  | (.700) | (.166) | (.724) | (.586) | (.517) | (.916) | (.712) | (.873) | (.751) |
| Massachusetts | .033 | .257 | .096 | .737 | -.267 | .346 | .274 | .195 | .327 |
|  | .416 | .447 | .403 | .402 | .389 | .363 | .386 | .394 | .344 |
|  | [-.782, .848] | [-.620, 1.134] | [-.693, .886] | [-.050, 1.525] | [-1.028, .495] | [-.366, 1.057] | [-.482, 1.030] | [-.577, .966] | [-.347, 1.002] |
|  | (.936) | (.566) | (.811) | (.067) | (.493) | (.341) | (.477) | (.621) | (.341) |
| Michigan | .037 | .487 | .129 | .406 | -.301 | -.283 | -.111 | -.180 | -.141 |
|  | .353 | .419 | .366 | .346 | .321 | .324 | .349 | .323 | .328 |
|  | [-.656, .730] | [-.333, 1.308] | [-.589, .847] | [-.271, 1.083] | [-.930, .327] | [-.918, .353] | [-.794, .573] | [-.813, .453] | [-.783, .502] |
|  | (.916) | (.245) | (.725) | (.240) | (.348) | (.383) | (.751) | (.578) | (.668) |
| Minnesota | .064 | .147 | 1.116 | .284 | -.202 | -.318 | .009 | -.071 | -.199 |
|  | .409 | .472 | .477 | .396 | .394 | .379 | .409 | .369 | .367 |
|  | [-.737, .865] | [-.778, 1.071] | [.180, 2.052] | [-.492, 1.060] | [-.975, .571] | [-1.060, .425] | [-.793, .810] | [-.794, .652] | [-.918, .520] |
|  | (.876) | (.756) | (.019) | (.473) | (.609) | (.402) | (.983) | (.846) | (.588) |
| Mississippi | .600 | -.227 | -.111 | .423 | -.603 | -.378 | -.316 | -.763 | -.071 |
|  | .458 | .519 | .455 | .476 | .468 | .398 | .476 | .509 | .437 |
|  | [-.299, 1.498] | [-1.244, .791] | [-1.004, .781] | [-.511, 1.356] | [-1.520, .314] | [-1.159, .403] | [-1.249, .617] | [-1.762, .235] | [-.927, .785] |
|  | (.191) | (.662) | (.807) | (.375) | (.197) | (.343) | (.507) | (.134) | (.871) |
| Missouri | -.283 | .237 | -.138 | -.005 | -.540 | -.356 | -.198 | -.117 | -.158 |
|  | .393 | .440 | .381 | .369 | .336 | .348 | .355 | .335 | .322 |
|  | [-1.054, .488] | [-.625, 1.098] | [-.886, .609] | [-.728, .718] | [-1.200, .119] | [-1.037, .326] | [-.895, .498] | [-.773, .539] | [-.789, .473] |
|  | (.472) | (.591) | (.717) | (.990) | (.108) | (.306) | (.577) | (.726) | (.623) |
| Montana | -.284 | .407 | .036 | .822 | .454 | -.490 | .454 | -.318 | .028 |
|  | .603 | .720 | .695 | .473 | .541 | .603 | .798 | .618 | .490 |
|  | [-1.465, .898] | [-1.004, 1.819] | [-1.327, 1.398] | [-.105, 1.749] | [-.605, 1.514] | [-1.673, .693] | [-1.110, 2.019] | [-1.530, .893] | [-.932, .989] |
|  | (.638) | (.571) | (.959) | (.082) | (.401) | (.417) | (.569) | (.607) | (.954) |
| Nebraska | .050 | -.158 | .474 | .544 | -.098 | -.393 | -.137 | .471 | .032 |
|  | .502 | .786 | .481 | .780 | .635 | .533 | .625 | .580 | .692 |
|  | [-.934, 1.035] | [-1.699, 1.382] | [-.470, 1.417] | [-.984, 2.072] | [-1.343, 1.146] | [-1.438, .653] | [-1.362, 1.087] | [-.665, 1.607] | [-1.324, 1.388] |
|  | (.921) | (.840) | (.325) | (.485) | (.877) | (.462) | (.826) | (.416) | (.963) |
| Nevada | .237 | .117 | .316 | .485 | .069 | .173 | -.130 | -.199 | .094 |
|  | .397 | .468 | .442 | .404 | .440 | .382 | .447 | .408 | .392 |
|  | [-.542, 1.016] | [-.801, 1.034] | [-.550, 1.183] | [-.307, 1.276] | [-.793, .931] | [-.576, .923] | [-1.007, .747] | [-.999, .601] | [-.676, .863] |
|  | (.551) | (.803) | (.474) | (.230) | (.875) | (.651) | (.772) | (.626) | (.812) |
| New Hampshire | .491 | -.013 | .030 | .098 | -.869 | -.247 | -.625 | -.424 | .079 |
|  | .512 | .634 | .469 | .505 | .471 | .416 | .470 | .552 | .463 |
|  | [-.513, 1.494] | [-1.255, 1.229] | [-.888, .949] | [-.892, 1.089] | [-1.792, .054] | [-1.062, .567] | [-1.547, .296] | [-1.506, .659] | [-.828, .986] |
|  | (.338) | (.984) | (.949) | (.846) | (.065) | (.552) | (.184) | (.443) | (.864) |
| New Jersey | .491 | .364 | -.303 | .612 | -.509 | .098 | .200 | .054 | .420 |
|  | .393 | .451 | .392 | .384 | .381 | .375 | .399 | .331 | .396 |
|  | [-.279, 1.261] | [-.521, 1.248] | [-1.071, .465] | [-.141, 1.365] | [-1.255, .238] | [-.637, .834] | [-.581, .981] | [-.596, .703] | [-.355, 1.196] |
|  | (.211) | (.420) | (.440) | (.111) | (.182) | (.793) | (.616) | (.872) | (.288) |
| New Mexico | .428 | .295 | -.406 | .274 | -.002 | -.381 | -.163 | .365 | -.136 |
|  | .450 | .505 | .509 | .491 | .435 | .426 | .414 | .471 | .423 |
|  | [-.454, 1.310] | [-.694, 1.284] | [-1.403, .591] | [-.688, 1.236] | [-.855, .850] | [-1.215, .454] | [-.974, .648] | [-.557, 1.287] | [-.965, .694] |
|  | (.342) | (.559) | (.425) | (.577) | (.996) | (.371) | (.693) | (.438) | (.749) |
| New York | .347 | .319 | -.248 | .656 | -.248 | .110 | -.053 | .009 | .271 |
|  | .355 | .396 | .357 | .331 | .311 | .311 | .328 | .307 | .299 |
|  | [-.349, 1.042] | [-.459, 1.096] | [-.948, .451] | [.007, 1.305] | [-.859, .362] | [-.499, .720] | [-.696, .591] | [-.592, .610] | [-.314, .857] |
|  | (.328) | (.422) | (.486) | (.048) | (.425) | (.723) | (.872) | (.977) | (.364) |
| North Carolina | -.309 | .406 | .029 | .451 | -.096 | -.222 | .017 | .272 | .149 |
|  | .396 | .425 | .366 | .369 | .344 | .344 | .360 | .341 | .350 |
|  | [-1.086, .468] | [-.428, 1.240] | [-.689, .747] | [-.273, 1.175] | [-.769, .578] | [-.896, .452] | [-.688, .722] | [-.396, .941] | [-.537, .836] |
|  | (.436) | (.340) | (.938) | (.222) | (.780) | (.519) | (.962) | (.425) | (.670) |
| North Dakota | -.013 | .681 | .589 | .158 | -.589 | -.826 | .341 | -.677 | -.454 |
|  | .582 | .914 | 1.002 | .621 | .542 | .492 | .645 | .849 | .787 |
|  | [-1.154, 1.128] | [-1.111, 2.473] | [-1.374, 2.552] | [-1.059, 1.375] | [-1.652, .473] | [-1.791, .139] | [-.924, 1.605] | [-2.341, .988] | [-1.997, 1.089] |
|  | (.983) | (.456) | (.556) | (.799) | (.277) | (.093) | (.598) | (.426) | (.564) |
| Ohio | .030 | .333 | .010 | .295 | -.179 | -.169 | -.109 | -.414 | -.236 |
|  | .358 | .414 | .360 | .354 | .323 | .329 | .349 | .319 | .319 |
|  | [-.673, .732] | [-.478, 1.144] | [-.695, .716] | [-.399, .989] | [-.811, .454] | [-.814, .475] | [-.793, .576] | [-1.040, .211] | [-.861, .390] |
|  | (.934) | (.421) | (.977) | (.404) | (.579) | (.606) | (.755) | (.194) | (.460) |
| Oklahoma | -.101 | .381 | .568 | -.004 | -.166 | -.162 | -.086 | -.188 | -.325 |
|  | .550 | .602 | .491 | .470 | .506 | .427 | .465 | .426 | .499 |
|  | [-1.179, .977] | [-.799, 1.561] | [-.394, 1.530] | [-.926, .918] | [-1.158, .827] | [-.999, .675] | [-.996, .825] | [-1.023, .646] | [-1.304, .653] |
|  | (.855) | (.527) | (.247) | (.993) | (.743) | (.704) | (.854) | (.658) | (.515) |
| Oregon | -.271 | .453 | .757 | .271 | .265 | -.311 | .184 | -.111 | .183 |
|  | .373 | .454 | .393 | .381 | .370 | .350 | .381 | .351 | .372 |
|  | [-1.001, .460] | [-.438, 1.343] | [-.014, 1.528] | [-.476, 1.018] | [-.459, .989] | [-.997, .375] | [-.563, .931] | [-.799, .577] | [-.545, .912] |
|  | (.468) | (.319) | (.054) | (.477) | (.473) | (.375) | (.629) | (.751) | (.622) |
| Pennsylvania | .032 | .456 | .150 | .283 | -.421 | -.358 | -.105 | -.053 | -.065 |
|  | .340 | .397 | .337 | .332 | .313 | .304 | .335 | .300 | .306 |
|  | [-.635, .698] | [-.321, 1.234] | [-.511, .812] | [-.368, .934] | [-1.034, .191] | [-.955, .238] | [-.761, .551] | [-.642, .536] | [-.664, .535] |
|  | (.925) | (.250) | (.656) | (.394) | (.178) | (.239) | (.754) | (.860) | (.832) |
| Rhode Island | .168 | .631 | -.362 | .906 | -.234 | .070 | .166 | .043 | -.035 |
|  | .678 | .845 | .617 | .827 | .655 | .863 | .655 | .594 | .631 |
|  | [-1.161, 1.496] | [-1.025, 2.288] | [-1.571, .846] | [-.714, 2.527] | [-1.517, 1.049] | [-1.622, 1.762] | [-1.118, 1.450] | [-1.123, 1.208] | [-1.271, 1.202] |
|  | (.805) | (.455) | (.557) | (.273) | (.721) | (.936) | (.800) | (.943) | (.956) |
| South Carolina | .104 | .201 | -.023 | .110 | -.023 | -.259 | .213 | .042 | -.099 |
|  | .431 | .470 | .415 | .384 | .363 | .378 | .404 | .380 | .371 |
|  | [-.741, .949] | [-.721, 1.123] | [-.837, .790] | [-.642, .863] | [-.735, .690] | [-.999, .481] | [-.579, 1.005] | [-.704, .787] | [-.826, .628] |
|  | (.809) | (.669) | (.955) | (.773) | (.950) | (.493) | (.598) | (.912) | (.790) |
| South Dakota | .444 | .316 | .359 | -1.132 | -.144 | -1.389 | -.291 | -.036 | -.735 |
|  | .507 | .741 | .539 | .554 | .568 | .627 | .619 | .660 | .573 |
|  | [-.550, 1.438] | [-1.136, 1.767] | [-.697, 1.415] | [-2.218, -.047] | [-1.258, .970] | [-2.618, -.159] | [-1.504, .922] | [-1.330, 1.258] | [-1.858, .387] |
|  | (.382) | (.670) | (.505) | (.041) | (.800) | (.027) | (.639) | (.957) | (.199) |
| Tennessee | .072 | .543 | .187 | .682 | -.187 | .283 | .247 | -.326 | .060 |
|  | .416 | .479 | .377 | .406 | .356 | .369 | .381 | .366 | .383 |
|  | [-.742, .887] | [-.396, 1.482] | [-.551, .925] | [-.115, 1.479] | [-.885, .510] | [-.440, 1.005] | [-.501, .994] | [-1.043, .391] | [-.692, .811] |
|  | (.862) | (.257) | (.619) | (.093) | (.599) | (.443) | (.518) | (.373) | (.876) |
| Texas | -.404 | .421 | .049 | .167 | -.330 | -.530 | -.086 | .033 | .121 |
|  | .348 | .394 | .349 | .340 | .310 | .318 | .337 | .304 | .309 |
|  | [-1.086, .279] | [-.351, 1.194] | [-.635, .732] | [-.499, .833] | [-.937, .277] | [-1.153, .093] | [-.746, .574] | [-.561, .628] | [-.485, .726] |
|  | (.246) | (.285) | (.889) | (.624) | (.287) | (.096) | (.798) | (.912) | (.696) |
| Utah | -.094 | .379 | .858 | .718 | -.319 | -.221 | -.330 | .372 | .159 |
|  | .493 | .629 | .461 | .507 | .406 | .470 | .467 | .541 | .468 |
|  | [-1.060, .872] | [-.855, 1.612] | [-.046, 1.763] | [-.277, 1.712] | [-1.115, .478] | [-1.142, .700] | [-1.244, .585] | [-.689, 1.432] | [-.759, 1.077] |
|  | (.848) | (.547) | (.063) | (.157) | (.433) | (.639) | (.480) | (.492) | (.734) |
| Vermont | .994 | .240 | -.154 | -.071 | -.490 | -.865 | -.304 | .216 | -1.373 |
|  | .519 | .764 | .659 | .517 | .555 | .543 | .506 | .494 | .567 |
|  | [-.023, 2.010] | [-1.256, 1.736] | [-1.446, 1.137] | [-1.085, .942] | [-1.577, .597] | [-1.929, .199] | [-1.296, .688] | [-.751, 1.184] | [-2.483, -.262] |
|  | (.055) | (.753) | (.815) | (.890) | (.377) | (.111) | (.548) | (.661) | (.015) |
| Virginia | .208 | .512 | .205 | .220 | -.377 | -.352 | -.336 | -.424 | .004 |
|  | .354 | .418 | .362 | .356 | .328 | .326 | .357 | .331 | .334 |
|  | [-.485, .901] | [-.307, 1.331] | [-.505, .915] | [-.478, .918] | [-1.021, .266] | [-.992, .287] | [-1.035, .363] | [-1.073, .224] | [-.651, .659] |
|  | (.557) | (.220) | (.571) | (.537) | (.251) | (.280) | (.346) | (.200) | (.991) |
| Washington | .561 | .460 | .463 | .515 | -.492 | -.013 | .019 | .012 | -.066 |
|  | .381 | .464 | .375 | .369 | .342 | .340 | .363 | .331 | .333 |
|  | [-.187, 1.308] | [-.449, 1.369] | [-.272, 1.197] | [-.208, 1.238] | [-1.163, .179] | [-.679, .653] | [-.692, .729] | [-.637, .661] | [-.718, .586] |
|  | (.142) | (.321) | (.217) | (.163) | (.151) | (.970) | (.959) | (.971) | (.842) |
| West Virginia | -.363 | .607 | .331 | .415 | -.450 | .050 | -.016 | -.102 | .241 |
|  | .503 | .593 | .440 | .494 | .422 | .525 | .395 | .488 | .423 |
|  | [-1.349, .622] | [-.556, 1.770] | [-.532, 1.194] | [-.553, 1.384] | [-1.278, .378] | [-.979, 1.079] | [-.789, .758] | [-1.059, .855] | [-.588, 1.070] |
|  | (.470) | (.306) | (.452) | (.401) | (.286) | (.924) | (.968) | (.834) | (.569) |
| Wisconsin | .176 | .379 | -.065 | .188 | -.620 | -.751 | -.319 | -.401 | -.383 |
|  | .414 | .468 | .381 | .377 | .379 | .365 | .374 | .340 | .351 |
|  | [-.635, .988] | [-.539, 1.296] | [-.812, .682] | [-.549, .926] | [-1.363, .123] | [-1.466, -.037] | [-1.052, .413] | [-1.068, .266] | [-1.071, .305] |
|  | (.670) | (.419) | (.865) | (.617) | (.102) | (.039) | (.393) | (.239) | (.275) |
| Wyoming | 1.063 | .538 | -1.011 | -1.477 | .015 | -.524 | -.018 | .235 | .123 |
|  | .487 | 1.121 | 1.589 | 2.356 | .643 | 1.998 | 1.079 | 1.562 | 1.847 |
|  | [.109, 2.017] | [-1.659, 2.735] | [-4.125, 2.104] | [-6.094, 3.139] | [-1.246, 1.275] | [-4.440, 3.391] | [-2.132, 2.096] | [-2.826, 3.296] | [-3.497, 3.742] |
|  | (.029) | (.631) | (.525) | (.531) | (.982) | (.793) | (.987) | (.880) | (.947) |
| cut1 | -3.756 | -2.657 | -1.253 | -.960 | -2.903 | -1.470 | -2.205 | -1.548 | -2.272 |
|  | .488 | .499 | .429 | .439 | .388 | .380 | .419 | .398 | .398 |
|  | [-4.713, -2.799] | [-3.635, -1.679] | [-2.093, -.413] | [-1.820, -.100] | [-3.664, -2.142] | [-2.216, -.724] | [-3.027, -1.383] | [-2.328, -.768] | [-3.051, -1.493] |
|  | (<.001) | (<.001) | (.003) | (.029) | (<.001) | (<.001) | (<.001) | (<.001) | (<.001) |
| cut2 | -1.316 | .238 | -.261 | .913 | -1.022 | .471 | -.452 | -.056 | -.356 |
|  | .450 | .486 | .420 | .438 | .375 | .378 | .413 | .396 | .393 |
|  | [-2.198, -.435] | [-.714, 1.190] | [-1.084, .563] | [.054, 1.771] | [-1.758, -.287] | [-.269, 1.211] | [-1.261, .357] | [-.834, .721] | [-1.128, .415] |
|  | (.003) | (.624) | (.535) | (.037) | (.006) | (.212) | (.274) | (.887) | (.365) |
| cut3 | .440 |  | 1.763 | 1.944 | .169 | 1.681 | .662 | .903 | .718 |
|  | .447 |  | .420 | .439 | .375 | .378 | .412 | .397 | .392 |
|  | [-.436, 1.316] |  | [.939, 2.586] | [1.084, 2.804] | [-.566, .904] | [.941, 2.422] | [-.146, 1.470] | [.126, 1.680] | [-.050, 1.487] |
|  | (.325) |  | (<.001) | (<.001) | (.652) | (<.001) | (.108) | (.023) | (.067) |
| cut4 | 2.280 |  | 2.409 |  |  |  |  |  |  |
|  | .450 |  | .421 |  |  |  |  |  |  |
|  | [1.397, 3.162] |  | [1.583, 3.234] |  |  |  |  |  |  |
|  | (<.001) |  | (<.001) |  |  |  |  |  |  |
| cut5 | 3.176 |  |  |  |  |  |  |  |  |
|  | .454 |  |  |  |  |  |  |  |  |
|  | [2.287, 4.066] |  |  |  |  |  |  |  |  |
|  | (<.001) |  |  |  |  |  |  |  |  |
| N | 2982 | 2980 | 2968 | 2980 | 2980 | 2981 | 2981 | 2981 | 2979 |
| Pseudo-R^2^ | .0224 | .0824 | .0605 | .0330 | .0290 | .0328 | .0255 | .0265 | .0353 |
| Model χ^2^ | 21.8 | 2421.9 | 739.5 | 276.3 | 538.5 | 275.2 | 209.8 | 22.3 | 27.2 |
| d.f. | 87 | 87 | 87 | 87 | 87 | 87 | 87 | 87 | 87 |
| p-value | <.001 | <.001 | <.001 | <.001 | <.001 | <.001 | <.001 | <.001 | <.001 |

S5 Table. Regression Results, Health Policies.

|  | (1) | (2) | (3) | (4) | (5) | (6) | (7) | (8) | (9) | (10) |
| --- | --- | --- | --- | --- | --- | --- | --- | --- | --- | --- |
|  | freetesting | waivecosts | closeschools | canceleverything | paidleave | compensatefirms | nationalguard | stopevents | travelban | stateslead |
| Democrat | 1.490 | 1.512 | .814 | .732 | 1.227 | -.191 | .308 | .839 | .024 | -.009 |
|  | .111 | .100 | .094 | .094 | .098 | .087 | .091 | .095 | .092 | .090 |
|  | [1.273, 1.706] | [1.316, 1.707] | [.631, .998] | [.548, .916] | [1.035, 1.419] | [-.362, -.021] | [.129, .486] | [.654, 1.025] | [-.156, .205] | [-.185, .167] |
|  | (<.001) | (<.001) | (<.001) | (<.001) | (<.001) | (.028) | (.001) | (<.001) | (.790) | (.921) |
| Other | .548 | .637 | .218 | .056 | .353 | -.517 | -.259 | .175 | -.318 | -.293 |
|  | .101 | .095 | .095 | .095 | .095 | .085 | .094 | .092 | .093 | .089 |
|  | [.350, .746] | [.451, .823] | [.032, .405] | [-.131, .242] | [.168, .539] | [-.683, -.352] | [-.443, -.075] | [-.006, .355] | [-.500, -.136] | [-.468, -.118] |
|  | (<.001) | (<.001) | (.022) | (.557) | (<.001) | (<.001) | (.006) | (.058) | (.001) | (.001) |
| 35- | .343 | .357 | .201 | .253 | .295 | .126 | .056 | .122 | -.004 | .358 |
|  | .118 | .112 | .106 | .104 | .118 | .108 | .097 | .104 | .099 | .101 |
|  | [.111, .575] | [.138, .576] | [-.008, .410] | [.050, .456] | [.064, .527] | [-.085, .337] | [-.135, .246] | [-.081, .325] | [-.197, .190] | [.159, .556] |
|  | (.004) | (.001) | (.059) | (.015) | (.012) | (.241) | (.566) | (.237) | (.972) | (<.001) |
| 50- | .405 | .233 | .215 | .318 | .114 | .068 | -.082 | .309 | -.215 | .380 |
|  | .116 | .104 | .100 | .101 | .107 | .100 | .091 | .097 | .093 | .094 |
|  | [.178, .633] | [.029, .437] | [.019, .410] | [.120, .516] | [-.095, .324] | [-.127, .264] | [-.261, .096] | [.118, .500] | [-.398, -.031] | [.195, .564] |
|  | (<.001) | (.025) | (.031) | (.002) | (.285) | (.494) | (.365) | (.002) | (.022) | (<.001) |
| 70- | .511 | .004 | .210 | .617 | -.090 | -.088 | .339 | .430 | -.363 | .592 |
|  | .143 | .127 | .122 | .125 | .128 | .116 | .116 | .124 | .121 | .118 |
|  | [.231, .792] | [-.244, .252] | [-.028, .449] | [.372, .863] | [-.342, .162] | [-.316, .139] | [.112, .567] | [.187, .673] | [-.599, -.126] | [.362, .823] |
|  | (<.001) | (.976) | (.084) | (<.001) | (.483) | (.446) | (.003) | (.001) | (.003) | (<.001) |
| gender | .289 | .104 | .327 | .418 | .265 | .465 | .258 | .299 | .725 | .150 |
|  | .083 | .076 | .072 | .072 | .075 | .070 | .071 | .072 | .070 | .070 |
|  | [.127, .451] | [-.046, .253] | [.187, .468] | [.277, .560] | [.118, .413] | [.328, .602] | [.120, .397] | [.158, .440] | [.588, .862] | [.012, .288] |
|  | (<.001) | (.174) | (<.001) | (<.001) | (<.001) | (<.001) | (<.001) | (<.001) | (<.001) | (.033) |
| white | .296 | .327 | -.001 | .018 | .290 | -.099 | -.268 | .411 | -.375 | -.074 |
|  | .101 | .093 | .088 | .086 | .093 | .087 | .084 | .088 | .081 | .086 |
|  | [.099, .494] | [.144, .510] | [-.174, .172] | [-.149, .186] | [.107, .473] | [-.270, .071] | [-.432, -.103] | [.239, .584] | [-.534, -.216] | [-.243, .095] |
|  | (.003) | (<.001) | (.994) | (.831) | (.002) | (.253) | (.001) | (<.001) | (<.001) | (.390) |
| married | -.111 | -.017 | -.008 | .083 | -.062 | -.008 | .186 | -.046 | .242 | -.013 |
|  | .092 | .083 | .077 | .079 | .084 | .078 | .075 | .079 | .076 | .076 |
|  | [-.290, .069] | [-.179, .146] | [-.158, .143] | [-.072, .237] | [-.226, .102] | [-.160, .144] | [.040, .333] | [-.202, .110] | [.094, .391] | [-.162, .136] |
|  | (.227) | (.842) | (.921) | (.293) | (.458) | (.920) | (.013) | (.564) | (.001) | (.860) |
| High school graduate | -.474 | -.389 | -.320 | -.260 | -.586 | -.450 | -.201 | .133 | .024 | -.435 |
|  | .282 | .242 | .241 | .253 | .261 | .207 | .211 | .227 | .224 | .216 |
|  | [-1.027, .080] | [-.864, .085] | [-.793, .153] | [-.756, .236] | [-1.097, -.076] | [-.856, -.043] | [-.614, .213] | [-.312, .578] | [-.416, .463] | [-.857, -.013] |
|  | (.093) | (.108) | (.185) | (.305) | (.024) | (.030) | (.341) | (.558) | (.916) | (.043) |
| Some college | -.228 | -.153 | -.357 | -.206 | -.315 | -.647 | -.541 | .490 | -.482 | -.422 |
|  | .286 | .248 | .246 | .257 | .267 | .213 | .215 | .231 | .227 | .220 |
|  | [-.789, .334] | [-.639, .333] | [-.839, .124] | [-.709, .296] | [-.838, .207] | [-1.064, -.230] | [-.963, -.120] | [.038, .942] | [-.927, -.037] | [-.854, .010] |
|  | (.427) | (.537) | (.146) | (.421) | (.237) | (.002) | (.012) | (.033) | (.034) | (.056) |
| 2-year | -.243 | -.228 | -.373 | -.194 | -.458 | -.417 | -.452 | .433 | -.274 | -.454 |
|  | .300 | .258 | .257 | .267 | .274 | .222 | .226 | .240 | .235 | .230 |
|  | [-.830, .345] | [-.734, .278] | [-.877, .131] | [-.717, .328] | [-.994, .079] | [-.851, .017] | [-.894, -.010] | [-.038, .905] | [-.735, .187] | [-.906, -.003] |
|  | (.418) | (.377) | (.147) | (.466) | (.095) | (.060) | (.045) | (.071) | (.244) | (.048) |
| 4-year | -.586 | -.476 | -.553 | -.342 | -.585 | -.678 | -.552 | .447 | -.548 | -.481 |
|  | .289 | .249 | .246 | .260 | .267 | .213 | .220 | .235 | .229 | .224 |
|  | [-1.153, -.019] | [-.964, .013] | [-1.035, -.071] | [-.852, .168] | [-1.108, -.062] | [-1.096, -.260] | [-.983, -.122] | [-.014, .909] | [-.997, -.098] | [-.919, -.043] |
|  | (.043) | (.057) | (.025) | (.189) | (.028) | (.001) | (.012) | (.057) | (.017) | (.031) |
| Post-grad | -.477 | -.424 | -.637 | -.272 | -.506 | -.806 | -.505 | .689 | -.822 | -.684 |
|  | .299 | .261 | .257 | .267 | .279 | .225 | .233 | .251 | .242 | .235 |
|  | [-1.062, .108] | [-.936, .088] | [-1.140, -.133] | [-.796, .252] | [-1.053, .041] | [-1.247, -.365] | [-.961, -.049] | [.196, 1.182] | [-1.296, -.348] | [-1.144, -.223] |
|  | (.110) | (.105) | (.013) | (.309) | (.070) | (<.001) | (.030) | (.006) | (.001) | (.004) |
| income=2 | .229 | .035 | .095 | .051 | .127 | -.163 | .072 | .004 | -.068 | .068 |
|  | .249 | .238 | .206 | .222 | .224 | .222 | .191 | .197 | .195 | .206 |
|  | [-.259, .717] | [-.431, .501] | [-.308, .499] | [-.383, .486] | [-.311, .566] | [-.598, .271] | [-.302, .446] | [-.382, .391] | [-.450, .314] | [-.335, .471] |
|  | (.358) | (.883) | (.644) | (.816) | (.569) | (.462) | (.705) | (.982) | (.727) | (.741) |
| income=3 | .387 | .093 | .234 | .054 | .366 | -.185 | -.016 | -.095 | -.141 | .379 |
|  | .242 | .228 | .204 | .214 | .217 | .213 | .191 | .190 | .194 | .204 |
|  | [-.087, .861] | [-.353, .540] | [-.166, .634] | [-.366, .473] | [-.059, .791] | [-.604, .233] | [-.390, .358] | [-.468, .277] | [-.521, .238] | [-.021, .779] |
|  | (.110) | (.682) | (.252) | (.802) | (.091) | (.386) | (.934) | (.616) | (.465) | (.063) |
| income=4 | .486 | .280 | .259 | .203 | .267 | -.283 | .135 | .289 | -.109 | .268 |
|  | .251 | .237 | .211 | .219 | .222 | .219 | .186 | .202 | .196 | .210 |
|  | [-.006, .978] | [-.184, .744] | [-.156, .673] | [-.226, .631] | [-.169, .703] | [-.711, .146] | [-.230, .501] | [-.106, .684] | [-.494, .275] | [-.144, .680] |
|  | (.053) | (.237) | (.221) | (.354) | (.230) | (.197) | (.468) | (.152) | (.578) | (.202) |
| income=5 | .356 | .114 | -.026 | -.025 | .177 | -.527 | -.228 | .016 | -.561 | .143 |
|  | .248 | .233 | .219 | .223 | .224 | .221 | .192 | .199 | .202 | .205 |
|  | [-.130, .843] | [-.343, .572] | [-.456, .404] | [-.462, .413] | [-.262, .616] | [-.960, -.094] | [-.605, .149] | [-.374, .406] | [-.957, -.166] | [-.260, .545] |
|  | (.151) | (.624) | (.905) | (.912) | (.429) | (.017) | (.236) | (.934) | (.005) | (.488) |
| income=6 | .378 | .105 | .271 | .207 | .400 | -.253 | .140 | .192 | -.239 | .238 |
|  | .253 | .241 | .216 | .227 | .233 | .228 | .197 | .207 | .204 | .211 |
|  | [-.118, .873] | [-.367, .577] | [-.152, .695] | [-.237, .651] | [-.056, .856] | [-.700, .194] | [-.246, .526] | [-.215, .598] | [-.639, .160] | [-.176, .652] |
|  | (.136) | (.662) | (.209) | (.361) | (.086) | (.268) | (.477) | (.355) | (.241) | (.261) |
| income=7 | .516 | -.134 | .392 | .264 | .308 | -.038 | .042 | .396 | -.164 | -.019 |
|  | .267 | .245 | .221 | .230 | .235 | .230 | .200 | .217 | .215 | .221 |
|  | [-.007, 1.039] | [-.614, .346] | [-.042, .826] | [-.185, .714] | [-.153, .769] | [-.489, .414] | [-.350, .433] | [-.028, .821] | [-.585, .258] | [-.452, .414] |
|  | (.053) | (.584) | (.077) | (.249) | (.191) | (.870) | (.835) | (.067) | (.446) | (.932) |
| income=8 | .328 | -.021 | .118 | .027 | .415 | -.142 | -.209 | .024 | -.542 | .089 |
|  | .247 | .244 | .216 | .223 | .232 | .227 | .213 | .200 | .214 | .211 |
|  | [-.157, .813] | [-.500, .458] | [-.305, .541] | [-.410, .465] | [-.038, .869] | [-.588, .303] | [-.626, .209] | [-.369, .417] | [-.962, -.122] | [-.325, .503] |
|  | (.185) | (.931) | (.584) | (.903) | (.073) | (.531) | (.327) | (.904) | (.011) | (.674) |
| income=9 | -.039 | -.365 | .161 | -.047 | .099 | -.322 | -.351 | .253 | -.622 | .097 |
|  | .250 | .240 | .219 | .224 | .230 | .220 | .196 | .205 | .207 | .218 |
|  | [-.529, .451] | [-.836, .106] | [-.268, .591] | [-.486, .392] | [-.351, .549] | [-.752, .108] | [-.736, .034] | [-.149, .655] | [-1.028, -.216] | [-.330, .523] |
|  | (.876) | (.129) | (.461) | (.832) | (.666) | (.143) | (.074) | (.217) | (.003) | (.657) |
| income=10 | .378 | -.156 | -.007 | .177 | .298 | -.168 | -.136 | .270 | -.710 | .126 |
|  | .258 | .242 | .222 | .234 | .236 | .232 | .206 | .225 | .213 | .226 |
|  | [-.128, .883] | [-.629, .318] | [-.443, .428] | [-.282, .637] | [-.165, .761] | [-.622, .286] | [-.539, .268] | [-.170, .710] | [-1.128, -.292] | [-.317, .569] |
|  | (.143) | (.520) | (.973) | (.449) | (.207) | (.468) | (.510) | (.229) | (.001) | (.578) |
| income=11 | .392 | -.218 | .383 | .422 | .145 | -.179 | -.182 | .392 | -.575 | .161 |
|  | .267 | .259 | .234 | .241 | .249 | .245 | .214 | .219 | .231 | .234 |
|  | [-.132, .915] | [-.726, .290] | [-.075, .840] | [-.050, .894] | [-.342, .632] | [-.660, .302] | [-.601, .238] | [-.038, .822] | [-1.027, -.122] | [-.297, .619] |
|  | (.143) | (.400) | (.101) | (.080) | (.559) | (.466) | (.396) | (.074) | (.013) | (.490) |
| income=12 | .114 | -.401 | .175 | .194 | .071 | -.216 | -.434 | .617 | -.553 | .121 |
|  | .288 | .276 | .253 | .271 | .269 | .266 | .247 | .276 | .243 | .250 |
|  | [-.450, .679] | [-.941, .140] | [-.321, .670] | [-.337, .725] | [-.456, .598] | [-.738, .306] | [-.918, .050] | [.076, 1.159] | [-1.029, -.078] | [-.369, .610] |
|  | (.692) | (.147) | (.489) | (.474) | (.792) | (.418) | (.079) | (.025) | (.023) | (.629) |
| income=13 | .960 | .165 | .217 | .620 | .326 | -.642 | -.134 | .508 | -.525 | -.006 |
|  | .425 | .363 | .349 | .390 | .351 | .381 | .371 | .351 | .367 | .329 |
|  | [.127, 1.792] | [-.548, .877] | [-.467, .901] | [-.145, 1.384] | [-.363, 1.014] | [-1.389, .105] | [-.861, .593] | [-.181, 1.197] | [-1.244, .193] | [-.652, .639] |
|  | (.024) | (.650) | (.535) | (.112) | (.354) | (.092) | (.718) | (.148) | (.152) | (.985) |
| income=14 | -.207 | -.506 | -.384 | -.042 | -.183 | -.445 | -.727 | .379 | -.497 | -.494 |
|  | .455 | .412 | .342 | .338 | .407 | .333 | .436 | .368 | .374 | .376 |
|  | [-1.100, .685] | [-1.315, .302] | [-1.056, .287] | [-.704, .620] | [-.981, .615] | [-1.097, .207] | [-1.581, .128] | [-.341, 1.100] | [-1.230, .237] | [-1.231, .244] |
|  | (.649) | (.220) | (.262) | (.901) | (.652) | (.181) | (.096) | (.302) | (.185) | (.190) |
| income=15 | 15.716 | 13.458 | -.022 | .643 | 1.148 | -.013 | 2.277 | 1.328 | -1.374 | 15.036 |
|  | .841 | .859 | 1.145 | 1.925 | .514 | .596 | 1.083 | .564 | 1.095 | .637 |
|  | [14.068,17.363] | [11.774,15.142] | [-2.267, 2.222] | [-3.130, 4.415] | [.141, 2.155] | [-1.180, 1.155] | [.155, 4.399] | [.223, 2.433] | [-3.521, .773] | [13.788,16.285] |
|  | (<.001) | (<.001) | (.984) | (.738) | (.025) | (.983) | (.035) | (.019) | (.210) | (<.001) |
| income=16 | .735 | .637 | .706 | .927 | 1.116 | -.121 | .172 | -.119 | -.368 | .446 |
|  | .670 | .570 | .527 | .646 | .600 | .511 | .556 | .612 | .546 | .634 |
|  | [-.579, 2.048] | [-.481, 1.754] | [-.326, 1.738] | [-.339, 2.193] | [-.061, 2.292] | [-1.122, .880] | [-.918, 1.262] | [-1.318, 1.080] | [-1.438, .701] | [-.797, 1.689] |
|  | (.273) | (.264) | (.180) | (.151) | (.063) | (.813) | (.757) | (.846) | (.500) | (.482) |
| income=17 | .097 | -.129 | -.013 | -.066 | -.002 | -.329 | -.471 | .235 | -.636 | .143 |
|  | .231 | .224 | .195 | .206 | .211 | .208 | .181 | .179 | .187 | .196 |
|  | [-.356, .550] | [-.567, .310] | [-.395, .370] | [-.470, .337] | [-.415, .411] | [-.736, .078] | [-.825, -.117] | [-.115, .586] | [-1.002, -.270] | [-.242, .527] |
|  | (.676) | (.565) | (.949) | (.747) | (.993) | (.113) | (.009) | (.188) | (.001) | (.467) |
| Rural Code=2 | -.165 | -.116 | -.187 | -.134 | -.070 | -.160 | -.193 | -.002 | -.287 | -.050 |
|  | .108 | .101 | .095 | .096 | .098 | .093 | .092 | .094 | .090 | .093 |
|  | [-.377, .047] | [-.314, .083] | [-.373, -.002] | [-.322, .054] | [-.263, .123] | [-.342, .022] | [-.374, -.012] | [-.185, .182] | [-.464, -.110] | [-.231, .132] |
|  | (.127) | (.253) | (.048) | (.161) | (.478) | (.085) | (.037) | (.986) | (.001) | (.591) |
| Rural Code=3 | -.198 | -.334 | -.282 | -.085 | -.049 | -.017 | -.064 | .031 | .016 | -.127 |
|  | .143 | .131 | .129 | .127 | .135 | .128 | .119 | .127 | .122 | .123 |
|  | [-.479, .082] | [-.591, -.076] | [-.533, -.030] | [-.334, .165] | [-.313, .215] | [-.268, .234] | [-.298, .169] | [-.217, .280] | [-.223, .256] | [-.368, .114] |
|  | (.165) | (.011) | (.028) | (.506) | (.716) | (.895) | (.588) | (.804) | (.894) | (.301) |
| Rural Code=4 | -.032 | .009 | -.407 | -.068 | .164 | -.044 | -.220 | -.284 | -.469 | .096 |
|  | .238 | .204 | .196 | .198 | .204 | .188 | .170 | .184 | .196 | .169 |
|  | [-.497, .434] | [-.390, .409] | [-.792, -.022] | [-.457, .320] | [-.236, .564] | [-.413, .324] | [-.553, .112] | [-.645, .077] | [-.853, -.085] | [-.236, .428] |
|  | (.894) | (.963) | (.038) | (.731) | (.421) | (.813) | (.194) | (.122) | (.017) | (.569) |
| Rural Code=5 | .044 | -.141 | .143 | .214 | .253 | -.142 | .026 | .448 | .034 | -.226 |
|  | .356 | .363 | .280 | .269 | .314 | .260 | .263 | .276 | .264 | .254 |
|  | [-.655, .742] | [-.853, .570] | [-.405, .691] | [-.314, .742] | [-.362, .869] | [-.652, .368] | [-.489, .541] | [-.093, .989] | [-.484, .552] | [-.723, .271] |
|  | (.902) | (.697) | (.609) | (.428) | (.420) | (.586) | (.921) | (.104) | (.899) | (.373) |
| Rural Code=6 | -.024 | -.123 | -.219 | -.220 | -.034 | -.025 | .020 | -.125 | .152 | -.346 |
|  | .215 | .180 | .177 | .196 | .181 | .155 | .197 | .172 | .168 | .192 |
|  | [-.445, .397] | [-.475, .230] | [-.566, .129] | [-.605, .164] | [-.389, .321] | [-.328, .278] | [-.366, .407] | [-.462, .212] | [-.177, .481] | [-.722, .030] |
|  | (.911) | (.495) | (.218) | (.262) | (.852) | (.871) | (.917) | (.466) | (.365) | (.072) |
| Rural Code=7 | -.571 | -.218 | -.635 | -.324 | -.370 | -.241 | -.166 | -.292 | -.070 | -.365 |
|  | .253 | .255 | .256 | .270 | .261 | .221 | .232 | .254 | .249 | .266 |
|  | [-1.067, -.076] | [-.718, .282] | [-1.137, -.134] | [-.853, .204] | [-.882, .142] | [-.674, .193] | [-.620, .289] | [-.790, .206] | [-.559, .418] | [-.886, .155] |
|  | (.024) | (.393) | (.013) | (.229) | (.156) | (.276) | (.475) | (.251) | (.779) | (.169) |
| Rural Code=8 | -.046 | -.391 | -.292 | -.761 | -.144 | .186 | -1.116 | -.163 | .139 | .835 |
|  | .610 | .531 | .635 | .430 | .552 | .413 | .411 | .407 | .358 | .486 |
|  | [-1.241, 1.148] | [-1.432, .650] | [-1.536, .952] | [-1.605, .082] | [-1.227, .938] | [-.624, .996] | [-1.921, -.312] | [-.962, .635] | [-.561, .840] | [-.118, 1.787] |
|  | (.939) | (.462) | (.645) | (.077) | (.794) | (.653) | (.007) | (.689) | (.697) | (.086) |
| Rural Code=9 | -.129 | .051 | .180 | .004 | .268 | -.633 | .015 | -.005 | -.403 | -.177 |
|  | .455 | .423 | .399 | .417 | .429 | .391 | .329 | .338 | .353 | .269 |
|  | [-1.020, .763] | [-.777, .880] | [-.602, .962] | [-.814, .822] | [-.574, 1.109] | [-1.401, .134] | [-.630, .661] | [-.667, .657] | [-1.095, .290] | [-.704, .349] |
|  | (.777) | (.903) | (.652) | (.992) | (.533) | (.106) | (.963) | (.988) | (.255) | (.509) |
| Alaska | -.287 | .030 | .435 | .344 | -.283 | .528 | .336 | .269 | .948 | -.099 |
|  | .686 | 1.020 | .730 | .732 | .652 | .631 | .740 | .643 | 1.130 | .844 |
|  | [-1.630, 1.057] | [-1.968, 2.029] | [-.996, 1.866] | [-1.091, 1.778] | [-1.561, .995] | [-.709, 1.764] | [-1.115, 1.787] | [-.991, 1.529] | [-1.266, 3.162] | [-1.754, 1.555] |
|  | (.676) | (.976) | (.552) | (.639) | (.664) | (.403) | (.650) | (.675) | (.401) | (.906) |
| Arizona | -.397 | -.125 | .083 | -.185 | -.119 | -.416 | -.493 | .118 | -.752 | -.556 |
|  | .444 | .389 | .376 | .380 | .362 | .354 | .315 | .453 | .402 | .395 |
|  | [-1.266, .473] | [-.887, .638] | [-.655, .820] | [-.931, .560] | [-.829, .590] | [-1.109, .278] | [-1.110, .124] | [-.770, 1.005] | [-1.540, .036] | [-1.331, .219] |
|  | (.371) | (.749) | (.826) | (.626) | (.742) | (.240) | (.118) | (.795) | (.061) | (.160) |
| Arkansas | -.566 | -.310 | -.115 | -.310 | -.243 | -.403 | <.001 | .077 | -.426 | -.332 |
|  | .435 | .409 | .414 | .389 | .404 | .434 | .371 | .466 | .402 | .445 |
|  | [-1.418, .286] | [-1.110, .491] | [-.926, .696] | [-1.072, .452] | [-1.035, .549] | [-1.255, .448] | [-.727, .728] | [-.836, .991] | [-1.213, .362] | [-1.204, .540] |
|  | (.193) | (.448) | (.781) | (.426) | (.548) | (.353) | (.999) | (.868) | (.290) | (.455) |
| California | -.098 | .310 | .159 | -.028 | .023 | -.325 | .206 | .400 | -.228 | -.417 |
|  | .373 | .335 | .315 | .331 | .314 | .338 | .274 | .413 | .366 | .353 |
|  | [-.830, .633] | [-.347, .968] | [-.458, .775] | [-.677, .621] | [-.592, .638] | [-.988, .338] | [-.331, .743] | [-.408, 1.209] | [-.947, .490] | [-1.108, .275] |
|  | (.792) | (.355) | (.613) | (.932) | (.941) | (.337) | (.452) | (.332) | (.533) | (.238) |
| Colorado | -.141 | -.578 | -.122 | -.012 | -.307 | -.300 | .091 | .424 | -.366 | -.392 |
|  | .515 | .424 | .426 | .459 | .431 | .415 | .404 | .493 | .465 | .424 |
|  | [-1.151, .870] | [-1.410, .253] | [-.957, .714] | [-.911, .888] | [-1.152, .538] | [-1.114, .514] | [-.700, .883] | [-.541, 1.390] | [-1.277, .544] | [-1.223, .439] |
|  | (.785) | (.173) | (.775) | (.980) | (.476) | (.470) | (.821) | (.389) | (.431) | (.355) |
| Connecticut | .899 | .502 | .836 | .904 | .033 | .256 | .327 | .337 | .097 | .285 |
|  | .677 | .431 | .398 | .449 | .437 | .451 | .387 | .562 | .446 | .477 |
|  | [-.428, 2.227] | [-.343, 1.346] | [.057, 1.616] | [.023, 1.785] | [-.822, .889] | [-.629, 1.141] | [-.432, 1.087] | [-.765, 1.440] | [-.777, .971] | [-.649, 1.219] |
|  | (.184) | (.244) | (.035) | (.044) | (.939) | (.570) | (.399) | (.548) | (.828) | (.550) |
| Delaware | -.870 | .181 | .056 | .252 | -.721 | -.906 | .340 | -.147 | -.306 | -.694 |
|  | .572 | .546 | .546 | .621 | .568 | .734 | .550 | .689 | .673 | .626 |
|  | [-1.991, .251] | [-.888, 1.250] | [-1.015, 1.127] | [-.966, 1.469] | [-1.834, .393] | [-2.345, .533] | [-.738, 1.417] | [-1.498, 1.203] | [-1.625, 1.013] | [-1.922, .533] |
|  | (.128) | (.740) | (.918) | (.686) | (.205) | (.217) | (.537) | (.831) | (.650) | (.267) |
| District of Columbia | -1.191 | -.246 | .367 | -.489 | -.646 | -.063 | .669 | .949 | -.539 | -.007 |
|  | .872 | .638 | .976 | .651 | 1.105 | .450 | .723 | .789 | .635 | .835 |
|  | [-2.901, .519] | [-1.495, 1.004] | [-1.546, 2.280] | [-1.765, .787] | [-2.813, 1.520] | [-.944, .818] | [-.748, 2.086] | [-.597, 2.495] | [-1.784, .705] | [-1.643, 1.629] |
|  | (.172) | (.700) | (.707) | (.452) | (.559) | (.888) | (.355) | (.229) | (.396) | (.994) |
| Florida | -.336 | -.087 | .325 | -.169 | -.211 | -.211 | .338 | .343 | -.490 | -.168 |
|  | .368 | .333 | .319 | .327 | .314 | .337 | .266 | .408 | .363 | .354 |
|  | [-1.057, .386] | [-.740, .565] | [-.300, .949] | [-.811, .472] | [-.826, .403] | [-.871, .449] | [-.183, .859] | [-.457, 1.143] | [-1.202, .222] | [-.862, .527] |
|  | (.362) | (.793) | (.308) | (.605) | (.501) | (.531) | (.203) | (.401) | (.178) | (.636) |
| Georgia | -.457 | -.484 | .047 | -.390 | -.175 | -.515 | .282 | -.018 | -.603 | -.384 |
|  | .404 | .361 | .345 | .358 | .350 | .345 | .294 | .425 | .385 | .375 |
|  | [-1.249, .335] | [-1.190, .223] | [-.628, .722] | [-1.091, .312] | [-.860, .511] | [-1.192, .162] | [-.294, .858] | [-.850, .815] | [-1.357, .151] | [-1.119, .351] |
|  | (.258) | (.180) | (.892) | (.276) | (.618) | (.136) | (.337) | (.967) | (.117) | (.306) |
| Hawaii | 1.169 | 2.153 | 1.183 | .704 | .030 | -.269 | .658 | .234 | 1.195 | -.146 |
|  | 1.114 | .900 | .614 | .695 | .694 | .659 | .549 | .807 | .533 | .534 |
|  | [-1.014, 3.351] | [.389, 3.916] | [-.021, 2.387] | [-.658, 2.065] | [-1.330, 1.391] | [-1.560, 1.022] | [-.417, 1.734] | [-1.347, 1.815] | [.149, 2.240] | [-1.193, .900] |
|  | (.294) | (.017) | (.054) | (.311) | (.965) | (.683) | (.230) | (.772) | (.025) | (.784) |
| Idaho | -.577 | -.482 | .232 | -.562 | -.882 | -.581 | -.655 | .388 | -.594 | -.557 |
|  | .672 | .613 | .495 | .511 | .536 | .436 | .394 | .618 | .531 | .546 |
|  | [-1.895, .741] | [-1.684, .720] | [-.738, 1.203] | [-1.564, .439] | [-1.933, .168] | [-1.435, .273] | [-1.427, .118] | [-.824, 1.600] | [-1.635, .447] | [-1.628, .513] |
|  | (.391) | (.432) | (.639) | (.271) | (.100) | (.183) | (.097) | (.530) | (.264) | (.307) |
| Illinois | -.293 | -.095 | .246 | .087 | .145 | -.308 | .284 | .471 | -.390 | -.263 |
|  | .390 | .363 | .345 | .357 | .365 | .359 | .295 | .441 | .383 | .371 |
|  | [-1.058, .472] | [-.806, .617] | [-.430, .922] | [-.612, .786] | [-.570, .860] | [-1.012, .395] | [-.294, .861] | [-.395, 1.336] | [-1.142, .361] | [-.990, .463] |
|  | (.453) | (.795) | (.476) | (.807) | (.692) | (.391) | (.336) | (.286) | (.309) | (.477) |
| Indiana | .095 | .611 | .993 | .443 | .226 | -.292 | .193 | 1.015 | -.530 | -.429 |
|  | .438 | .403 | .400 | .386 | .415 | .389 | .341 | .466 | .411 | .418 |
|  | [-.764, .953] | [-.179, 1.401] | [.209, 1.777] | [-.314, 1.200] | [-.587, 1.040] | [-1.053, .470] | [-.476, .863] | [.101, 1.928] | [-1.335, .275] | [-1.248, .390] |
|  | (.829) | (.129) | (.013) | (.251) | (.585) | (.453) | (.571) | (.029) | (.197) | (.305) |
| Iowa | -.413 | -.316 | .104 | -.006 | -.299 | .098 | .491 | .799 | -.616 | -.172 |
|  | .509 | .432 | .439 | .420 | .433 | .404 | .410 | .525 | .411 | .479 |
|  | [-1.410, .584] | [-1.163, .531] | [-.756, .964] | [-.830, .818] | [-1.147, .550] | [-.693, .889] | [-.313, 1.295] | [-.230, 1.828] | [-1.422, .191] | [-1.110, .766] |
|  | (.416) | (.465) | (.813) | (.989) | (.491) | (.808) | (.231) | (.128) | (.135) | (.719) |
| Kansas | -.081 | .604 | -.103 | .468 | .725 | -.279 | .238 | .833 | -.449 | -.430 |
|  | .653 | .736 | .451 | .520 | .774 | .560 | .560 | .607 | .513 | .616 |
|  | [-1.361, 1.199] | [-.840, 2.047] | [-.987, .782] | [-.552, 1.488] | [-.792, 2.242] | [-1.378, .819] | [-.859, 1.335] | [-.357, 2.023] | [-1.454, .557] | [-1.638, .778] |
|  | (.901) | (.412) | (.820) | (.368) | (.349) | (.618) | (.670) | (.170) | (.382) | (.486) |
| Kentucky | -.846 | -.133 | .226 | -.481 | -.131 | .146 | -.230 | .375 | -.272 | -.297 |
|  | .448 | .422 | .411 | .401 | .398 | .409 | .334 | .494 | .452 | .421 |
|  | [-1.725, .033] | [-.961, .694] | [-.580, 1.031] | [-1.267, .306] | [-.911, .649] | [-.655, .946] | [-.885, .426] | [-.593, 1.342] | [-1.158, .615] | [-1.122, .527] |
|  | (.059) | (.752) | (.583) | (.231) | (.743) | (.721) | (.492) | (.448) | (.548) | (.480) |
| Louisiana | -.147 | .620 | .307 | -.195 | -.184 | -.201 | .219 | .204 | -.511 | -.351 |
|  | .542 | .505 | .476 | .462 | .443 | .469 | .353 | .529 | .473 | .445 |
|  | [-1.209, .914] | [-.369, 1.610] | [-.626, 1.240] | [-1.101, .710] | [-1.053, .685] | [-1.120, .719] | [-.473, .910] | [-.833, 1.240] | [-1.440, .417] | [-1.222, .521] |
|  | (.785) | (.219) | (.519) | (.672) | (.678) | (.669) | (.535) | (.700) | (.280) | (.430) |
| Maine | -.095 | .079 | 1.583 | 1.392 | .432 | -.194 | -.505 | .602 | -.358 | .397 |
|  | .640 | .538 | .564 | .845 | .650 | .611 | .492 | .706 | .541 | .548 |
|  | [-1.351, 1.160] | [-.975, 1.133] | [.478, 2.687] | [-.264, 3.048] | [-.841, 1.705] | [-1.392, 1.004] | [-1.469, .458] | [-.781, 1.986] | [-1.419, .702] | [-.676, 1.471] |
|  | (.881) | (.883) | (.005) | (.099) | (.506) | (.750) | (.304) | (.394) | (.508) | (.468) |
| Maryland | -.354 | .010 | -.001 | -.324 | .321 | -.384 | .516 | .049 | -.509 | -.187 |
|  | .468 | .412 | .416 | .413 | .443 | .405 | .387 | .469 | .464 | .434 |
|  | [-1.270, .563] | [-.798, .817] | [-.816, .814] | [-1.133, .486] | [-.547, 1.189] | [-1.178, .411] | [-.243, 1.276] | [-.870, .968] | [-1.419, .401] | [-1.038, .664] |
|  | (.449) | (.981) | (.998) | (.433) | (.468) | (.344) | (.183) | (.916) | (.273) | (.666) |
| Massachusetts | -.095 | .292 | -.015 | .076 | .003 | -.150 | .142 | .604 | -.274 | -.614 |
|  | .448 | .422 | .373 | .408 | .373 | .398 | .309 | .454 | .398 | .412 |
|  | [-.973, .784] | [-.535, 1.120] | [-.747, .716] | [-.724, .876] | [-.729, .734] | [-.930, .631] | [-.464, .749] | [-.285, 1.493] | [-1.053, .505] | [-1.422, .195] |
|  | (.832) | (.489) | (.967) | (.852) | (.994) | (.707) | (.646) | (.183) | (.491) | (.137) |
| Michigan | -.241 | .120 | .493 | -.232 | -.261 | -.288 | .069 | .560 | -.472 | -.214 |
|  | .401 | .357 | .343 | .349 | .347 | .358 | .311 | .433 | .393 | .378 |
|  | [-1.027, .546] | [-.580, .819] | [-.180, 1.166] | [-.917, .452] | [-.940, .418] | [-.990, .414] | [-.542, .679] | [-.288, 1.408] | [-1.242, .299] | [-.954, .527] |
|  | (.548) | (.737) | (.151) | (.506) | (.451) | (.421) | (.825) | (.195) | (.230) | (.572) |
| Minnesota | -.736 | -.220 | .446 | -.104 | -.535 | -.286 | .070 | .290 | -.081 | -.008 |
|  | .472 | .417 | .453 | .397 | .364 | .389 | .370 | .500 | .440 | .443 |
|  | [-1.662, .190] | [-1.038, .598] | [-.442, 1.334] | [-.882, .673] | [-1.248, .178] | [-1.047, .476] | [-.655, .794] | [-.691, 1.270] | [-.944, .782] | [-.876, .861] |
|  | (.119) | (.598) | (.325) | (.793) | (.142) | (.462) | (.851) | (.563) | (.854) | (.986) |
| Mississippi | -.698 | -.498 | .308 | -.103 | -.262 | .073 | .375 | .079 | -.301 | -.137 |
|  | .524 | .467 | .449 | .438 | .430 | .428 | .390 | .512 | .459 | .440 |
|  | [-1.725, .330] | [-1.412, .417] | [-.573, 1.188] | [-.961, .755] | [-1.105, .581] | [-.765, .911] | [-.390, 1.139] | [-.924, 1.083] | [-1.200, .598] | [-.999, .725] |
|  | (.183) | (.286) | (.493) | (.814) | (.543) | (.865) | (.337) | (.877) | (.512) | (.756) |
| Missouri | -.750 | -.270 | -.094 | -.433 | -.140 | -.446 | .131 | .163 | -.470 | -.246 |
|  | .416 | .371 | .353 | .391 | .355 | .386 | .313 | .452 | .400 | .411 |
|  | [-1.566, .065] | [-.998, .457] | [-.785, .597] | [-1.199, .332] | [-.837, .556] | [-1.203, .310] | [-.482, .744] | [-.723, 1.049] | [-1.254, .314] | [-1.052, .560] |
|  | (.071) | (.466) | (.790) | (.267) | (.693) | (.248) | (.676) | (.718) | (.240) | (.550) |
| Montana | -1.345 | -1.108 | -.244 | -.983 | -.236 | -1.147 | -.524 | -.532 | -1.238 | -.893 |
|  | .648 | .707 | .571 | .643 | .557 | .480 | .688 | .608 | .634 | .644 |
|  | [-2.615, -.075] | [-2.494, .278] | [-1.363, .876] | [-2.244, .278] | [-1.328, .856] | [-2.088, -.205] | [-1.871, .824] | [-1.725, .660] | [-2.479, .004] | [-2.156, .370] |
|  | (.038) | (.117) | (.670) | (.127) | (.672) | (.017) | (.446) | (.382) | (.051) | (.166) |
| Nebraska | -.514 | .403 | -.654 | -.389 | -.110 | .019 | .652 | -.201 | -.472 | -.299 |
|  | .609 | .622 | .551 | .574 | .650 | .651 | .734 | .610 | .684 | .559 |
|  | [-1.708, .679] | [-.817, 1.623] | [-1.733, .425] | [-1.514, .735] | [-1.383, 1.163] | [-1.257, 1.295] | [-.786, 2.091] | [-1.396, .994] | [-1.812, .868] | [-1.394, .796] |
|  | (.398) | (.517) | (.235) | (.497) | (.865) | (.977) | (.374) | (.741) | (.490) | (.593) |
| Nevada | .003 | .312 | .346 | -.055 | -.171 | -.525 | -.292 | .645 | -.249 | -.182 |
|  | .513 | .423 | .404 | .408 | .449 | .418 | .337 | .494 | .448 | .414 |
|  | [-1.002, 1.008] | [-.517, 1.140] | [-.446, 1.137] | [-.855, .746] | [-1.052, .710] | [-1.344, .294] | [-.953, .369] | [-.322, 1.612] | [-1.127, .628] | [-.993, .629] |
|  | (.995) | (.461) | (.392) | (.893) | (.704) | (.209) | (.386) | (.191) | (.578) | (.661) |
| New Hampshire | .162 | .771 | .094 | -.215 | .264 | -.375 | .591 | -.083 | -.364 | .094 |
|  | .540 | .569 | .500 | .515 | .556 | .455 | .522 | .545 | .493 | .581 |
|  | [-.897, 1.222] | [-.346, 1.887] | [-.886, 1.074] | [-1.224, .794] | [-.826, 1.354] | [-1.267, .517] | [-.431, 1.614] | [-1.151, .985] | [-1.331, .602] | [-1.046, 1.233] |
|  | (.764) | (.176) | (.851) | (.676) | (.635) | (.410) | (.257) | (.879) | (.460) | (.872) |
| New Jersey | -.112 | .524 | .436 | -.087 | .078 | .098 | .589 | .207 | -.247 | -.092 |
|  | .453 | .407 | .378 | .374 | .374 | .382 | .321 | .434 | .403 | .408 |
|  | [-1.001, .777] | [-.273, 1.321] | [-.304, 1.176] | [-.820, .646] | [-.655, .810] | [-.651, .846] | [-.040, 1.219] | [-.644, 1.057] | [-1.036, .542] | [-.892, .708] |
|  | (.805) | (.198) | (.249) | (.816) | (.836) | (.798) | (.066) | (.634) | (.539) | (.822) |
| New Mexico | .317 | .034 | .512 | -.210 | -.298 | -.523 | -.745 | .374 | -.432 | -.739 |
|  | .563 | .581 | .497 | .483 | .446 | .455 | .532 | .555 | .488 | .510 |
|  | [-.787, 1.421] | [-1.106, 1.173] | [-.462, 1.487] | [-1.156, .736] | [-1.171, .576] | [-1.415, .370] | [-1.788, .299] | [-.714, 1.462] | [-1.389, .525] | [-1.738, .260] |
|  | (.574) | (.954) | (.303) | (.663) | (.504) | (.251) | (.162) | (.500) | (.376) | (.147) |
| New York | -.561 | .090 | .313 | -.119 | -.129 | -.232 | .320 | .045 | -.015 | -.123 |
|  | .377 | .339 | .329 | .343 | .326 | .340 | .276 | .420 | .374 | .366 |
|  | [-1.300, .178] | [-.573, .754] | [-.331, .957] | [-.792, .554] | [-.767, .509] | [-.900, .435] | [-.221, .860] | [-.778, .868] | [-.748, .717] | [-.840, .593] |
|  | (.137) | (.790) | (.341) | (.729) | (.692) | (.495) | (.246) | (.914) | (.967) | (.736) |
| North Carolina | -.271 | .231 | .227 | .148 | -.077 | -.560 | .386 | .064 | -.058 | -.501 |
|  | .414 | .363 | .356 | .364 | .369 | .368 | .311 | .441 | .401 | .394 |
|  | [-1.084, .541] | [-.481, .943] | [-.471, .924] | [-.565, .862] | [-.800, .646] | [-1.281, .161] | [-.224, .997] | [-.799, .928] | [-.845, .728] | [-1.273, .271] |
|  | (.512) | (.525) | (.524) | (.684) | (.835) | (.128) | (.215) | (.884) | (.884) | (.203) |
| North Dakota | -.209 | .824 | -.635 | -.741 | .249 | .004 | .654 | -.436 | -.752 | -.884 |
|  | .890 | .619 | .740 | .530 | .716 | .797 | .461 | .562 | .598 | .571 |
|  | [-1.953, 1.536] | [-.389, 2.037] | [-2.086, .816] | [-1.781, .299] | [-1.155, 1.653] | [-1.558, 1.566] | [-.249, 1.557] | [-1.538, .667] | [-1.924, .421] | [-2.004, .236] |
|  | (.815) | (.183) | (.391) | (.162) | (.728) | (.996) | (.156) | (.439) | (.209) | (.122) |
| Ohio | -.409 | -.005 | .422 | .080 | -.115 | -.073 | -.047 | .511 | -.116 | -.022 |
|  | .401 | .354 | .347 | .366 | .341 | .366 | .287 | .436 | .381 | .374 |
|  | [-1.194, .377] | [-.700, .689] | [-.259, 1.102] | [-.636, .796] | [-.785, .554] | [-.790, .644] | [-.611, .516] | [-.344, 1.367] | [-.862, .630] | [-.755, .710] |
|  | (.308) | (.988) | (.225) | (.827) | (.735) | (.842) | (.869) | (.242) | (.760) | (.952) |
| Oklahoma | -.686 | .029 | .480 | .199 | -.262 | .501 | .615 | -.006 | -.055 | -.490 |
|  | .483 | .482 | .521 | .457 | .451 | .507 | .411 | .539 | .516 | .495 |
|  | [-1.633, .261] | [-.916, .975] | [-.541, 1.501] | [-.696, 1.094] | [-1.146, .623] | [-.493, 1.496] | [-.191, 1.421] | [-1.063, 1.050] | [-1.066, .956] | [-1.460, .480] |
|  | (.155) | (.951) | (.356) | (.662) | (.562) | (.323) | (.135) | (.991) | (.915) | (.323) |
| Oregon | .213 | .381 | -.403 | -.273 | .001 | -.438 | -.087 | .285 | -.917 | -.770 |
|  | .471 | .406 | .369 | .371 | .385 | .402 | .339 | .455 | .419 | .405 |
|  | [-.710, 1.137] | [-.415, 1.177] | [-1.126, .319] | [-1.000, .453] | [-.754, .756] | [-1.226, .351] | [-.750, .577] | [-.607, 1.177] | [-1.739, -.095] | [-1.564, .025] |
|  | (.651) | (.348) | (.274) | (.461) | (.999) | (.277) | (.798) | (.532) | (.029) | (.058) |
| Pennsylvania | -.274 | -.044 | .238 | -.016 | -.021 | -.231 | .019 | .403 | -.336 | -.152 |
|  | .377 | .336 | .325 | .334 | .318 | .343 | .265 | .413 | .373 | .356 |
|  | [-1.013, .466] | [-.703, .615] | [-.398, .875] | [-.670, .638] | [-.645, .603] | [-.903, .440] | [-.500, .538] | [-.407, 1.213] | [-1.067, .395] | [-.850, .547] |
|  | (.469) | (.896) | (.463) | (.961) | (.948) | (.500) | (.943) | (.329) | (.368) | (.670) |
| Rhode Island | 14.906 | .795 | .694 | .071 | .659 | 1.610 | .035 | -.242 | .281 | -.142 |
|  | .552 | 1.016 | .860 | .766 | .858 | .751 | .795 | .859 | 1.141 | .681 |
|  | [13.824,15.988] | [-1.196, 2.787] | [-.992, 2.379] | [-1.431, 1.572] | [-1.022, 2.340] | [.139, 3.082] | [-1.523, 1.593] | [-1.925, 1.441] | [-1.956, 2.518] | [-1.476, 1.192] |
|  | (<.001) | (.434) | (.420) | (.927) | (.442) | (.032) | (.965) | (.778) | (.806) | (.835) |
| South Carolina | .010 | -.030 | .526 | .265 | -.356 | -.185 | .046 | .489 | -.107 | -.101 |
|  | .498 | .448 | .384 | .391 | .442 | .416 | .377 | .459 | .444 | .425 |
|  | [-.967, .986] | [-.908, .848] | [-.227, 1.280] | [-.502, 1.032] | [-1.223, .510] | [-1.000, .630] | [-.693, .785] | [-.411, 1.389] | [-.978, .765] | [-.934, .731] |
|  | (.985) | (.947) | (.171) | (.498) | (.420) | (.656) | (.902) | (.287) | (.811) | (.811) |
| South Dakota | -.336 | -.321 | -.767 | -.499 | -.344 | -.186 | .281 | -.585 | -.062 | -.338 |
|  | .934 | .612 | .510 | .663 | .785 | .618 | .499 | .544 | .651 | .399 |
|  | [-2.168, 1.495] | [-1.519, .878] | [-1.767, .233] | [-1.798, .800] | [-1.883, 1.195] | [-1.397, 1.025] | [-.697, 1.259] | [-1.651, .481] | [-1.337, 1.213] | [-1.121, .444] |
|  | (.719) | (.600) | (.133) | (.451) | (.661) | (.763) | (.573) | (.282) | (.925) | (.396) |
| Tennessee | .010 | .501 | .178 | -.436 | -.192 | -.329 | -.121 | .491 | -.382 | -.394 |
|  | .479 | .420 | .410 | .434 | .380 | .380 | .348 | .494 | .466 | .428 |
|  | [-.929, .949] | [-.323, 1.325] | [-.626, .981] | [-1.287, .415] | [-.937, .553] | [-1.074, .417] | [-.803, .560] | [-.477, 1.460] | [-1.295, .531] | [-1.232, .444] |
|  | (.984) | (.233) | (.664) | (.315) | (.614) | (.388) | (.728) | (.320) | (.412) | (.357) |
| Texas | -.447 | -.037 | .038 | -.149 | -.402 | -.383 | .002 | .116 | -.343 | -.250 |
|  | .374 | .342 | .324 | .339 | .322 | .344 | .275 | .418 | .378 | .365 |
|  | [-1.179, .285] | [-.707, .633] | [-.598, .674] | [-.813, .516] | [-1.034, .229] | [-1.057, .292] | [-.537, .541] | [-.703, .935] | [-1.083, .398] | [-.965, .466] |
|  | (.231) | (.915) | (.906) | (.661) | (.211) | (.266) | (.994) | (.782) | (.365) | (.494) |
| Utah | -.354 | .065 | -.155 | .084 | -.100 | -.560 | .357 | .648 | -.377 | -.575 |
|  | .526 | .506 | .417 | .517 | .444 | .572 | .433 | .500 | .437 | .482 |
|  | [-1.385, .677] | [-.926, 1.056] | [-.972, .663] | [-.929, 1.097] | [-.970, .770] | [-1.682, .561] | [-.492, 1.207] | [-.331, 1.628] | [-1.234, .480] | [-1.519, .369] |
|  | (.501) | (.898) | (.711) | (.871) | (.822) | (.327) | (.410) | (.195) | (.389) | (.232) |
| Vermont | -.243 | -.042 | .515 | .499 | -.684 | -.495 | .397 | 1.032 | .033 | -.244 |
|  | .694 | .647 | .668 | .624 | .718 | .522 | .767 | .827 | .726 | .679 |
|  | [-1.604, 1.118] | [-1.309, 1.226] | [-.794, 1.823] | [-.723, 1.722] | [-2.091, .724] | [-1.518, .527] | [-1.106, 1.899] | [-.589, 2.652] | [-1.389, 1.455] | [-1.575, 1.088] |
|  | (.727) | (.949) | (.441) | (.423) | (.341) | (.342) | (.605) | (.212) | (.964) | (.720) |
| Virginia | -.172 | -.030 | .057 | -.248 | -.164 | -.510 | .131 | .419 | -.329 | -.100 |
|  | .414 | .368 | .344 | .356 | .347 | .363 | .295 | .440 | .386 | .380 |
|  | [-.984, .640] | [-.751, .691] | [-.617, .730] | [-.945, .450] | [-.844, .516] | [-1.222, .202] | [-.448, .710] | [-.444, 1.282] | [-1.085, .428] | [-.845, .644] |
|  | (.678) | (.935) | (.869) | (.487) | (.637) | (.160) | (.657) | (.342) | (.394) | (.792) |
| Washington | -.158 | .172 | -.146 | -.340 | .095 | -.498 | -.277 | .308 | -.413 | -.591 |
|  | .424 | .396 | .350 | .354 | .364 | .361 | .288 | .427 | .393 | .392 |
|  | [-.989, .673] | [-.605, .948] | [-.832, .539] | [-1.034, .354] | [-.619, .808] | [-1.205, .209] | [-.841, .286] | [-.528, 1.145] | [-1.183, .358] | [-1.358, .177] |
|  | (.709) | (.665) | (.675) | (.336) | (.794) | (.167) | (.335) | (.470) | (.294) | (.131) |
| West Virginia | -1.123 | -.104 | .545 | -.066 | -.289 | -.493 | .155 | .476 | .063 | -.013 |
|  | .555 | .491 | .484 | .490 | .485 | .427 | .468 | .502 | .513 | .531 |
|  | [-2.210, -.035] | [-1.066, .859] | [-.403, 1.493] | [-1.025, .893] | [-1.240, .662] | [-1.329, .343] | [-.762, 1.073] | [-.509, 1.460] | [-.943, 1.070] | [-1.054, 1.028] |
|  | (.043) | (.833) | (.260) | (.893) | (.551) | (.248) | (.740) | (.344) | (.902) | (.980) |
| Wisconsin | -.697 | -.118 | .189 | -.209 | -.620 | -.605 | .045 | .295 | -.096 | -.381 |
|  | .442 | .387 | .415 | .400 | .370 | .369 | .319 | .444 | .445 | .392 |
|  | [-1.563, .169] | [-.876, .639] | [-.625, 1.003] | [-.993, .575] | [-1.345, .105] | [-1.328, .118] | [-.580, .669] | [-.576, 1.166] | [-.967, .775] | [-1.150, .388] |
|  | (.115) | (.760) | (.649) | (.601) | (.094) | (.101) | (.889) | (.507) | (.829) | (.332) |
| Wyoming | 15.567 | 1.405 | 1.017 | .509 | -.180 | -.478 | 2.199 | -.644 | .665 | -.722 |
|  | .734 | .959 | 1.673 | 1.832 | 1.122 | 1.108 | 1.187 | 1.521 | .851 | 1.139 |
|  | [14.129,17.005] | [-.475, 3.285] | [-2.262, 4.295] | [-3.083, 4.100] | [-2.379, 2.019] | [-2.650, 1.694] | [-.128, 4.526] | [-3.624, 2.337] | [-1.003, 2.333] | [-2.955, 1.510] |
|  | (<.001) | (.143) | (.543) | (.781) | (.872) | (.666) | (.064) | (.672) | (.434) | (.526) |
| cut1 | -2.851 | -2.948 | -2.510 | -2.457 | -2.787 | -3.149 | -2.010 | -.452 | -1.976 | -3.629 |
|  | .483 | .439 | .431 | .434 | .438 | .424 | .360 | .498 | .433 | .429 |
|  | [-3.798, -1.903] | [-3.809, -2.086] | [-3.355, -1.666] | [-3.307, -1.606] | [-3.645, -1.929] | [-3.980, -2.318] | [-2.715, -1.305] | [-1.429, .524] | [-2.825, -1.127] | [-4.470, -2.788] |
|  | (<.001) | (<.001) | (<.001) | (<.001) | (<.001) | (<.001) | (<.001) | (.364) | (<.001) | (<.001) |
| cut2 | -1.973 | -1.632 | -1.378 | -1.311 | -1.839 | -2.012 | -1.018 | .517 | -.978 | -2.274 |
|  | .474 | .429 | .423 | .429 | .428 | .420 | .359 | .497 | .432 | .422 |
|  | [-2.903, -1.044] | [-2.474, -.791] | [-2.207, -.548] | [-2.152, -.470] | [-2.677, -1.000] | [-2.834, -1.189] | [-1.721, -.315] | [-.458, 1.492] | [-1.825, -.131] | [-3.102, -1.446] |
|  | (<.001) | (<.001) | (.001) | (.002) | (<.001) | (<.001) | (.005) | (.298) | (.024) | (<.001) |
| cut3 | -.658 | -.351 | -.183 | -.157 | -.544 | -.886 | .277 | 1.436 | .101 | -1.007 |
|  | .478 | .430 | .422 | .429 | .424 | .421 | .359 | .498 | .433 | .420 |
|  | [-1.594, .278] | [-1.193, .492] | [-1.011, .645] | [-.997, .684] | [-1.376, .288] | [-1.711, -.062] | [-.427, .982] | [.461, 2.412] | [-.747, .950] | [-1.832, -.183] |
|  | (.168) | (.415) | (.664) | (.715) | (.200) | (.035) | (.441) | (.004) | (.815) | (.017) |
| cut4 | .347 | .691 | 1.120 | 1.146 | .549 | .661 | 1.446 | 2.305 | 1.201 | .533 |
|  | .476 | .429 | .423 | .429 | .423 | .421 | .362 | .499 | .433 | .419 |
|  | [-.586, 1.280] | [-.149, 1.532] | [.292, 1.949] | [.305, 1.986] | [-.281, 1.379] | [-.165, 1.487] | [.737, 2.155] | [1.327, 3.284] | [.351, 2.050] | [-.289, 1.356] |
|  | (.466) | (.107) | (.008) | (.008) | (.195) | (.117) | (<.001) | (<.001) | (.006) | (.203) |
| N | 2970 | 2974 | 2967 | 2973 | 2973 | 2973 | 2974 | 2975 | 2970 | 2973 |
| Pseudo-R^2^ | .0586 | .0562 | .0305 | .0316 | .0432 | .0216 | .0290 | .0348 | .0433 | .0170 |
| Model χ^2^ | 2456.6 | 618.0 | 239.8 | 237.5 | 296.8 | 201.4 | 281.3 | 283.0 | 406.4 | 75.8 |
| d.f. | 87 | 87 | 87 | 87 | 87 | 87 | 87 | 87 | 87 | 87 |
| p-value | <.001 | <.001 | <.001 | <.001 | <.001 | <.001 | <.001 | <.001 | <.001 | <.001 |

S6 Table. Regression Results, Public Policies.

|  | (1) | (2) | (3) | (4) | (5) | (6) | (7) | (8) | (9) | (10) |
| --- | --- | --- | --- | --- | --- | --- | --- | --- | --- | --- |
|  | increasetrade | importtaxes | lowinterest | notreliant | noairtravel | banentry | entrymexico | quarantine | rescueUS | delayelections |
| Democrat | 1.174 | -1.215 | -.445 | -1.355 | -.568 | -1.153 | -1.859 | -.774 | .172 | -.396 |
|  | .094 | .093 | .092 | .099 | .092 | .097 | .102 | .094 | .091 | .088 |
|  | [.990, 1.358] | [-1.396, -1.033] | [-.625, -.265] | [-1.549, -1.162] | [-.748, -.387] | [-1.342, -.963] | [-2.059, -1.659] | [-.959, -.589] | [-.007, .351] | [-.569, -.224] |
|  | (<.001) | (<.001) | (<.001) | (<.001) | (<.001) | (<.001) | (<.001) | (<.001) | (.060) | (<.001) |
| Other | .233 | -.718 | -.526 | -.730 | -.433 | -.871 | -1.096 | -.614 | -.102 | -.250 |
|  | .094 | .092 | .091 | .100 | .093 | .096 | .104 | .090 | .090 | .089 |
|  | [.049, .417] | [-.898, -.538] | [-.705, -.346] | [-.925, -.534] | [-.615, -.251] | [-1.059, -.684] | [-1.300, -.892] | [-.790, -.437] | [-.279, .075] | [-.424, -.077] |
|  | (.013) | (<.001) | (<.001) | (<.001) | (<.001) | (<.001) | (<.001) | (<.001) | (.258) | (.005) |
| 35- | -.143 | .280 | .337 | .492 | .347 | .257 | .342 | .172 | .074 | -.127 |
|  | .100 | .101 | .103 | .101 | .101 | .099 | .102 | .104 | .098 | .098 |
|  | [-.338, .052] | [.082, .477] | [.135, .539] | [.294, .690] | [.149, .546] | [.064, .451] | [.143, .541] | [-.032, .376] | [-.118, .266] | [-.320, .066] |
|  | (.152) | (.005) | (.001) | (<.001) | (.001) | (.009) | (.001) | (.098) | (.450) | (.196) |
| 50- | .014 | .251 | .438 | .843 | .327 | .506 | .771 | -.006 | .211 | -.453 |
|  | .095 | .095 | .096 | .098 | .096 | .094 | .099 | .101 | .093 | .094 |
|  | [-.173, .201] | [.065, .436] | [.251, .626] | [.651, 1.035] | [.138, .515] | [.321, .691] | [.576, .965] | [-.203, .191] | [.028, .393] | [-.636, -.270] |
|  | (.881) | (.008) | (<.001) | (<.001) | (.001) | (<.001) | (<.001) | (.953) | (.024) | (<.001) |
| 70- | .179 | .059 | .463 | .855 | .158 | .423 | .851 | -.244 | .457 | -.304 |
|  | .121 | .114 | .119 | .129 | .117 | .124 | .127 | .121 | .118 | .114 |
|  | [-.059, .417] | [-.164, .282] | [.230, .695] | [.603, 1.108] | [-.072, .387] | [.179, .667] | [.601, 1.100] | [-.482, -.006] | [.226, .688] | [-.528, -.080] |
|  | (.140) | (.605) | (<.001) | (<.001) | (.178) | (.001) | (<.001) | (.044) | (<.001) | (.008) |
| gender | -.323 | .272 | .147 | -.164 | .392 | .156 | .006 | .180 | -.106 | .551 |
|  | .071 | .071 | .071 | .073 | .070 | .071 | .074 | .071 | .070 | .069 |
|  | [-.463, -.183] | [.132, .411] | [.008, .286] | [-.308, -.020] | [.254, .530] | [.016, .296] | [-.140, .152] | [.040, .320] | [-.243, .031] | [.416, .687] |
|  | (<.001) | (<.001) | (.038) | (.025) | (<.001) | (.029) | (.936) | (.012) | (.131) | (<.001) |
| white | -.046 | -.168 | -.175 | -.173 | -.165 | -.057 | -.091 | -.422 | .067 | -.108 |
|  | .087 | .087 | .086 | .087 | .083 | .085 | .087 | .086 | .086 | .082 |
|  | [-.216, .124] | [-.338, .002] | [-.343, -.006] | [-.344, -.002] | [-.328, -.002] | [-.224, .110] | [-.262, .079] | [-.591, -.253] | [-.102, .236] | [-.268, .052] |
|  | (.596) | (.053) | (.042) | (.048) | (.048) | (.507) | (.293) | (<.001) | (.440) | (.187) |
| married | -.055 | .185 | -.012 | .004 | .134 | .037 | .017 | .079 | -.064 | -.076 |
|  | .078 | .077 | .078 | .081 | .075 | .079 | .082 | .079 | .078 | .075 |
|  | [-.209, .098] | [.034, .336] | [-.165, .140] | [-.155, .164] | [-.013, .280] | [-.119, .192] | [-.144, .179] | [-.077, .234] | [-.216, .088] | [-.223, .071] |
|  | (.480) | (.016) | (.872) | (.957) | (.073) | (.645) | (.833) | (.320) | (.409) | (.313) |
| High school graduate | -.580 | -.224 | -.161 | -.288 | -.001 | -.687 | -.386 | -.549 | .040 | -.035 |
|  | .211 | .198 | .235 | .225 | .213 | .236 | .224 | .192 | .204 | .206 |
|  | [-.994, -.166] | [-.612, .164] | [-.621, .299] | [-.729, .154] | [-.418, .416] | [-1.150, -.223] | [-.825, .052] | [-.925, -.173] | [-.360, .440] | [-.439, .369] |
|  | (.006) | (.258) | (.493) | (.201) | (.996) | (.004) | (.084) | (.004) | (.845) | (.865) |
| Some college | -.488 | -.499 | -.183 | -.295 | -.361 | -.945 | -.607 | -1.193 | .137 | -.254 |
|  | .217 | .205 | .239 | .230 | .215 | .239 | .228 | .196 | .210 | .211 |
|  | [-.913, -.064] | [-.901, -.098] | [-.652, .285] | [-.746, .155] | [-.782, .060] | [-1.414, -.477] | [-1.054, -.160] | [-1.578, -.808] | [-.274, .548] | [-.668, .160] |
|  | (.024) | (.015) | (.444) | (.199) | (.093) | (<.001) | (.008) | (<.001) | (.512) | (.229) |
| 2-year | -.461 | -.230 | -.152 | -.206 | -.221 | -.724 | -.475 | -1.085 | -.032 | -.141 |
|  | .224 | .210 | .248 | .238 | .225 | .248 | .235 | .207 | .215 | .220 |
|  | [-.899, -.022] | [-.642, .182] | [-.638, .334] | [-.673, .262] | [-.663, .220] | [-1.209, -.239] | [-.936, -.014] | [-1.490, -.679] | [-.455, .390] | [-.571, .290] |
|  | (.040) | (.273) | (.539) | (.389) | (.326) | (.003) | (.043) | (<.001) | (.881) | (.523) |
| 4-year | -.161 | -.706 | -.441 | -.670 | -.656 | -1.282 | -.856 | -1.307 | .029 | -.289 |
|  | .220 | .208 | .240 | .233 | .220 | .244 | .232 | .205 | .211 | .214 |
|  | [-.593, .271] | [-1.113, -.299] | [-.910, .029] | [-1.126, -.214] | [-1.088, -.225] | [-1.760, -.803] | [-1.310, -.402] | [-1.708, -.906] | [-.384, .443] | [-.709, .131] |
|  | (.465) | (.001) | (.066) | (.004) | (.003) | (<.001) | (<.001) | (<.001) | (.890) | (.177) |
| Post-grad | -.029 | -.872 | -.471 | -.728 | -.940 | -1.265 | -1.066 | -1.695 | -.048 | -.538 |
|  | .233 | .223 | .250 | .245 | .230 | .253 | .244 | .225 | .224 | .229 |
|  | [-.486, .427] | [-1.308, -.435] | [-.960, .019] | [-1.208, -.248] | [-1.390, -.489] | [-1.762, -.768] | [-1.544, -.589] | [-2.137, -1.253] | [-.488, .392] | [-.988, -.089] |
|  | (.900) | (<.001) | (.059) | (.003) | (<.001) | (<.001) | (<.001) | (<.001) | (.830) | (.019) |
| income=2 | -.294 | .190 | .178 | .090 | .253 | .236 | .266 | .011 | .027 | .152 |
|  | .196 | .193 | .216 | .211 | .202 | .193 | .212 | .177 | .209 | .215 |
|  | [-.678, .089] | [-.187, .568] | [-.246, .601] | [-.324, .504] | [-.142, .648] | [-.143, .615] | [-.150, .682] | [-.336, .357] | [-.382, .436] | [-.269, .573] |
|  | (.132) | (.323) | (.411) | (.669) | (.210) | (.222) | (.211) | (.952) | (.899) | (.480) |
| income=3 | -.135 | .127 | .228 | .246 | .135 | .239 | .311 | -.283 | .205 | .252 |
|  | .189 | .188 | .216 | .205 | .194 | .195 | .209 | .174 | .201 | .212 |
|  | [-.506, .237] | [-.241, .495] | [-.196, .651] | [-.156, .648] | [-.245, .516] | [-.142, .621] | [-.100, .721] | [-.624, .059] | [-.190, .599] | [-.164, .667] |
|  | (.477) | (.498) | (.292) | (.230) | (.485) | (.219) | (.138) | (.105) | (.310) | (.235) |
| income=4 | -.076 | .095 | .352 | .178 | .105 | .002 | .167 | -.302 | .015 | .094 |
|  | .193 | .195 | .217 | .207 | .197 | .200 | .208 | .185 | .205 | .212 |
|  | [-.454, .303] | [-.287, .477] | [-.073, .777] | [-.229, .584] | [-.282, .492] | [-.390, .393] | [-.239, .574] | [-.665, .061] | [-.387, .416] | [-.320, .509] |
|  | (.694) | (.626) | (.104) | (.392) | (.596) | (.994) | (.420) | (.103) | (.942) | (.656) |
| income=5 | -.158 | .004 | .116 | .414 | .070 | .157 | .384 | -.537 | .167 | -.023 |
|  | .196 | .191 | .222 | .215 | .200 | .203 | .218 | .181 | .206 | .213 |
|  | [-.542, .226] | [-.370, .378] | [-.319, .551] | [-.006, .835] | [-.322, .462] | [-.240, .554] | [-.043, .810] | [-.892, -.182] | [-.238, .572] | [-.441, .395] |
|  | (.421) | (.984) | (.601) | (.053) | (.726) | (.438) | (.078) | (.003) | (.418) | (.915) |
| income=6 | -.173 | -.045 | .177 | .580 | .162 | .228 | .355 | -.386 | .104 | .182 |
|  | .193 | .203 | .221 | .219 | .205 | .200 | .214 | .190 | .203 | .222 |
|  | [-.552, .206] | [-.443, .353] | [-.256, .610] | [.151, 1.009] | [-.240, .565] | [-.163, .619] | [-.065, .775] | [-.760, -.013] | [-.294, .503] | [-.254, .617] |
|  | (.372) | (.825) | (.423) | (.008) | (.429) | (.252) | (.097) | (.042) | (.608) | (.414) |
| income=7 | -.268 | -.046 | .173 | .351 | .341 | .483 | .255 | -.379 | -.132 | .022 |
|  | .214 | .206 | .228 | .235 | .205 | .214 | .234 | .211 | .227 | .225 |
|  | [-.687, .150] | [-.450, .358] | [-.274, .619] | [-.109, .811] | [-.061, .743] | [.064, .903] | [-.203, .714] | [-.792, .035] | [-.577, .313] | [-.419, .463] |
|  | (.209) | (.822) | (.449) | (.135) | (.097) | (.024) | (.275) | (.073) | (.561) | (.922) |
| income=8 | -.341 | -.064 | .284 | .368 | .023 | .113 | .286 | -.449 | .012 | -.023 |
|  | .212 | .215 | .225 | .226 | .209 | .209 | .218 | .200 | .217 | .223 |
|  | [-.756, .074] | [-.485, .357] | [-.157, .725] | [-.075, .810] | [-.387, .432] | [-.298, .523] | [-.142, .714] | [-.841, -.057] | [-.412, .437] | [-.460, .414] |
|  | (.108) | (.766) | (.207) | (.104) | (.914) | (.591) | (.190) | (.025) | (.954) | (.918) |
| income=9 | .078 | -.276 | .213 | .184 | -.277 | .157 | .087 | -.616 | .019 | -.262 |
|  | .209 | .202 | .227 | .224 | .207 | .213 | .229 | .196 | .223 | .224 |
|  | [-.331, .487] | [-.672, .120] | [-.231, .658] | [-.256, .623] | [-.682, .128] | [-.260, .574] | [-.363, .536] | [-.999, -.232] | [-.418, .457] | [-.701, .177] |
|  | (.708) | (.172) | (.346) | (.412) | (.180) | (.461) | (.706) | (.002) | (.931) | (.242) |
| income=10 | .002 | -.307 | .203 | .166 | -.015 | -.057 | .278 | -.713 | .015 | -.275 |
|  | .214 | .212 | .233 | .232 | .227 | .214 | .235 | .218 | .229 | .235 |
|  | [-.417, .421] | [-.721, .108] | [-.254, .660] | [-.290, .622] | [-.460, .430] | [-.477, .363] | [-.182, .737] | [-1.140, -.286] | [-.434, .464] | [-.736, .186] |
|  | (.992) | (.147) | (.384) | (.475) | (.949) | (.789) | (.237) | (.001) | (.948) | (.243) |
| income=11 | .039 | -.395 | .297 | -.066 | -.206 | .054 | .014 | -.895 | -.073 | -.001 |
|  | .222 | .234 | .242 | .240 | .243 | .237 | .237 | .223 | .227 | .244 |
|  | [-.396, .473] | [-.852, .063] | [-.177, .770] | [-.536, .404] | [-.683, .270] | [-.411, .519] | [-.450, .478] | [-1.332, -.459] | [-.519, .372] | [-.480, .478] |
|  | (.862) | (.091) | (.219) | (.783) | (.396) | (.820) | (.952) | (<.001) | (.747) | (.996) |
| income=12 | -.098 | -.351 | .161 | -.053 | -.134 | .081 | .432 | -1.009 | -.022 | -.262 |
|  | .256 | .274 | .267 | .275 | .257 | .254 | .278 | .255 | .254 | .258 |
|  | [-.600, .404] | [-.889, .187] | [-.361, .684] | [-.593, .487] | [-.638, .370] | [-.418, .579] | [-.113, .978] | [-1.510, -.509] | [-.519, .475] | [-.768, .244] |
|  | (.702) | (.201) | (.545) | (.847) | (.602) | (.751) | (.120) | (<.001) | (.930) | (.310) |
| income=13 | .183 | -.676 | -.310 | -.192 | -.353 | .075 | .069 | -1.151 | -.259 | -.431 |
|  | .293 | .331 | .380 | .310 | .375 | .323 | .344 | .357 | .321 | .371 |
|  | [-.390, .757] | [-1.325, -.026] | [-1.056, .436] | [-.800, .416] | [-1.087, .382] | [-.558, .708] | [-.605, .743] | [-1.851, -.451] | [-.889, .371] | [-1.159, .296] |
|  | (.531) | (.041) | (.415) | (.536) | (.347) | (.817) | (.841) | (.001) | (.420) | (.245) |
| income=14 | -.181 | -.201 | .099 | .215 | -.044 | .198 | .314 | -.950 | -.474 | .092 |
|  | .404 | .376 | .294 | .348 | .395 | .339 | .342 | .358 | .376 | .391 |
|  | [-.974, .612] | [-.938, .536] | [-.478, .676] | [-.467, .896] | [-.818, .730] | [-.467, .862] | [-.356, .984] | [-1.652, -.247] | [-1.211, .263] | [-.675, .858] |
|  | (.654) | (.593) | (.737) | (.537) | (.912) | (.560) | (.359) | (.008) | (.208) | (.815) |
| income=15 | .824 | -.350 | -.735 | .982 | .808 | .910 | -.357 | -.533 | 1.883 | .654 |
|  | 2.047 | 1.738 | .925 | 1.332 | .574 | .426 | 1.594 | 1.889 | 1.093 | 1.058 |
|  | [-3.188, 4.837] | [-3.757, 3.058] | [-2.548, 1.078] | [-1.629, 3.594] | [-.317, 1.933] | [.075, 1.745] | [-3.482, 2.768] | [-4.235, 3.169] | [-.259, 4.025] | [-1.418, 2.727] |
|  | (.687) | (.841) | (.427) | (.461) | (.159) | (.033) | (.823) | (.778) | (.085) | (.536) |
| income=16 | -.117 | .024 | .614 | .053 | .107 | .471 | 1.037 | -.931 | -.070 | -.053 |
|  | .494 | .577 | .552 | .560 | .434 | .410 | .489 | .600 | .882 | .444 |
|  | [-1.084, .850] | [-1.106, 1.154] | [-.469, 1.696] | [-1.045, 1.150] | [-.744, .959] | [-.333, 1.275] | [.080, 1.995] | [-2.107, .245] | [-1.798, 1.658] | [-.924, .818] |
|  | (.812) | (.966) | (.267) | (.925) | (.805) | (.251) | (.034) | (.121) | (.937) | (.905) |
| income=17 | -.411 | -.002 | -.080 | .240 | -.105 | .157 | .287 | -.410 | -.144 | -.125 |
|  | .183 | .180 | .207 | .197 | .185 | .185 | .198 | .173 | .191 | .200 |
|  | [-.771, -.052] | [-.355, .351] | [-.486, .326] | [-.147, .626] | [-.468, .259] | [-.206, .520] | [-.101, .676] | [-.749, -.071] | [-.518, .231] | [-.518, .268] |
|  | (.025) | (.991) | (.699) | (.224) | (.572) | (.396) | (.147) | (.018) | (.452) | (.532) |
| Rural Code=2 | -.195 | -.077 | -.117 | .123 | -.102 | -.092 | -.141 | -.025 | -.180 | -.204 |
|  | .094 | .093 | .094 | .096 | .094 | .093 | .100 | .092 | .092 | .093 |
|  | [-.379, -.011] | [-.260, .106] | [-.300, .067] | [-.065, .311] | [-.287, .082] | [-.275, .090] | [-.337, .055] | [-.206, .156] | [-.360, .001] | [-.387, -.021] |
|  | (.037) | (.412) | (.213) | (.199) | (.277) | (.320) | (.159) | (.787) | (.051) | (.029) |
| Rural Code=3 | -.162 | -.071 | -.029 | .081 | .009 | .153 | -.009 | .105 | -.176 | .002 |
|  | .124 | .121 | .125 | .125 | .125 | .130 | .126 | .121 | .125 | .118 |
|  | [-.406, .082] | [-.309, .166] | [-.274, .217] | [-.165, .326] | [-.236, .255] | [-.102, .409] | [-.255, .238] | [-.131, .342] | [-.421, .068] | [-.230, .234] |
|  | (.193) | (.556) | (.820) | (.519) | (.940) | (.239) | (.946) | (.382) | (.158) | (.985) |
| Rural Code=4 | -.332 | .099 | .136 | .260 | .149 | .084 | .489 | .237 | .109 | -.214 |
|  | .177 | .169 | .183 | .190 | .184 | .175 | .184 | .151 | .176 | .161 |
|  | [-.679, .015] | [-.232, .431] | [-.222, .495] | [-.112, .631] | [-.213, .510] | [-.258, .427] | [.129, .849] | [-.059, .534] | [-.235, .453] | [-.529, .101] |
|  | (.061) | (.558) | (.456) | (.171) | (.421) | (.629) | (.008) | (.117) | (.535) | (.183) |
| Rural Code=5 | -.168 | -.117 | -.288 | -.220 | .158 | .032 | .185 | -.048 | -.511 | .281 |
|  | .231 | .210 | .261 | .251 | .269 | .303 | .317 | .297 | .322 | .278 |
|  | [-.621, .285] | [-.529, .295] | [-.799, .223] | [-.713, .273] | [-.369, .685] | [-.562, .626] | [-.437, .807] | [-.629, .533] | [-1.142, .121] | [-.264, .827] |
|  | (.468) | (.579) | (.269) | (.381) | (.557) | (.916) | (.560) | (.872) | (.113) | (.312) |
| Rural Code=6 | -.173 | .184 | .141 | .215 | .114 | .171 | .205 | .275 | -.093 | -.023 |
|  | .200 | .187 | .173 | .191 | .174 | .182 | .182 | .170 | .170 | .173 |
|  | [-.565, .219] | [-.183, .552] | [-.198, .480] | [-.159, .590] | [-.227, .455] | [-.187, .528] | [-.152, .562] | [-.058, .607] | [-.427, .241] | [-.362, .316] |
|  | (.387) | (.326) | (.414) | (.260) | (.512) | (.349) | (.260) | (.105) | (.585) | (.894) |
| Rural Code=7 | -.159 | .012 | -.461 | .239 | -.026 | .488 | -.013 | .100 | .091 | -.155 |
|  | .211 | .250 | .249 | .272 | .249 | .272 | .302 | .242 | .218 | .226 |
|  | [-.573, .255] | [-.477, .502] | [-.949, .028] | [-.293, .771] | [-.513, .461] | [-.045, 1.021] | [-.605, .579] | [-.374, .574] | [-.337, .519] | [-.597, .287] |
|  | (.452) | (.961) | (.065) | (.379) | (.916) | (.073) | (.967) | (.680) | (.676) | (.492) |
| Rural Code=8 | -1.016 | -.133 | -.299 | .717 | .214 | .056 | .408 | -.100 | .523 | .003 |
|  | .443 | .284 | .428 | .539 | .511 | .596 | .548 | .481 | .484 | .432 |
|  | [-1.885, -.148] | [-.690, .425] | [-1.139, .541] | [-.340, 1.773] | [-.788, 1.216] | [-1.113, 1.225] | [-.666, 1.482] | [-1.042, .842] | [-.427, 1.472] | [-.843, .850] |
|  | (.022) | (.640) | (.485) | (.184) | (.675) | (.925) | (.456) | (.835) | (.281) | (.994) |
| Rural Code=9 | -.082 | -.233 | -.370 | -.104 | -.140 | -.169 | -.732 | -.303 | -.567 | .288 |
|  | .341 | .363 | .315 | .457 | .367 | .378 | .378 | .330 | .252 | .302 |
|  | [-.749, .586] | [-.944, .478] | [-.987, .248] | [-1.001, .792] | [-.859, .580] | [-.909, .572] | [-1.472, .008] | [-.950, .343] | [-1.060, -.073] | [-.305, .880] |
|  | (.810) | (.520) | (.241) | (.819) | (.704) | (.655) | (.053) | (.358) | (.024) | (.341) |
| Alaska | -.242 | 1.212 | .465 | .029 | .224 | -.821 | .050 | -.038 | -.221 | -.421 |
|  | .688 | .624 | .717 | .647 | .980 | .838 | .709 | .786 | .530 | .906 |
|  | [-1.590, 1.106] | [-.012, 2.436] | [-.941, 1.871] | [-1.238, 1.297] | [-1.696, 2.144] | [-2.465, .822] | [-1.339, 1.440] | [-1.579, 1.503] | [-1.261, .818] | [-2.197, 1.355] |
|  | (.725) | (.052) | (.517) | (.964) | (.819) | (.327) | (.943) | (.962) | (.676) | (.642) |
| Arizona | .016 | -.255 | -.737 | -.490 | -.677 | -.094 | -.588 | -.414 | -.110 | -.985 |
|  | .363 | .375 | .391 | .405 | .439 | .407 | .360 | .363 | .384 | .372 |
|  | [-.695, .727] | [-.990, .481] | [-1.504, .030] | [-1.285, .304] | [-1.538, .184] | [-.893, .704] | [-1.293, .117] | [-1.126, .298] | [-.862, .642] | [-1.715, -.255] |
|  | (.965) | (.497) | (.060) | (.226) | (.123) | (.817) | (.102) | (.254) | (.774) | (.008) |
| Arkansas | .125 | .315 | -.006 | -.028 | -.028 | .205 | .176 | .130 | -.048 | .104 |
|  | .410 | .410 | .451 | .436 | .495 | .447 | .416 | .385 | .412 | .450 |
|  | [-.679, .929] | [-.487, 1.118] | [-.890, .878] | [-.884, .827] | [-.998, .943] | [-.670, 1.080] | [-.638, .991] | [-.626, .885] | [-.857, .760] | [-.778, .986] |
|  | (.761) | (.441) | (.990) | (.949) | (.956) | (.646) | (.671) | (.736) | (.907) | (.817) |
| California | -.058 | .026 | -.479 | .006 | -.364 | -.024 | -.033 | -.135 | .011 | -.578 |
|  | .307 | .349 | .348 | .366 | .401 | .346 | .310 | .331 | .339 | .339 |
|  | [-.660, .545] | [-.659, .710] | [-1.161, .202] | [-.712, .724] | [-1.150, .422] | [-.703, .654] | [-.641, .575] | [-.784, .513] | [-.653, .676] | [-1.242, .087] |
|  | (.851) | (.942) | (.168) | (.988) | (.364) | (.944) | (.915) | (.682) | (.973) | (.088) |
| Colorado | -.678 | .302 | -.296 | -.108 | -.560 | .143 | -.050 | -.387 | -.477 | -.351 |
|  | .429 | .399 | .391 | .455 | .484 | .425 | .401 | .391 | .414 | .394 |
|  | [-1.518, .162] | [-.481, 1.084] | [-1.063, .471] | [-1.000, .785] | [-1.509, .388] | [-.691, .976] | [-.836, .735] | [-1.154, .379] | [-1.289, .335] | [-1.123, .421] |
|  | (.114) | (.450) | (.449) | (.813) | (.247) | (.737) | (.900) | (.322) | (.249) | (.373) |
| Connecticut | .734 | .377 | -.433 | -.470 | -.279 | .554 | .185 | .183 | .358 | -.296 |
|  | .428 | .504 | .480 | .453 | .522 | .440 | .407 | .437 | .449 | .447 |
|  | [-.104, 1.573] | [-.611, 1.364] | [-1.374, .508] | [-1.358, .418] | [-1.303, .744] | [-.309, 1.418] | [-.613, .983] | [-.674, 1.039] | [-.521, 1.238] | [-1.171, .580] |
|  | (.086) | (.455) | (.367) | (.300) | (.593) | (.208) | (.649) | (.676) | (.425) | (.508) |
| Delaware | .170 | .225 | .377 | -.378 | .022 | -.443 | .109 | -.098 | .456 | -1.132 |
|  | .572 | .487 | .719 | .447 | .593 | .485 | .500 | .585 | .711 | .589 |
|  | [-.951, 1.290] | [-.730, 1.179] | [-1.033, 1.786] | [-1.254, .497] | [-1.141, 1.185] | [-1.393, .506] | [-.871, 1.089] | [-1.246, 1.049] | [-.938, 1.849] | [-2.287, .023] |
|  | (.767) | (.645) | (.600) | (.397) | (.970) | (.360) | (.827) | (.866) | (.522) | (.055) |
| District of Columbia | -.522 | .076 | .042 | -.649 | -.694 | .558 | .823 | -.434 | -.012 | .896 |
|  | .414 | .618 | .559 | .612 | .678 | .648 | .971 | .645 | .668 | .965 |
|  | [-1.333, .288] | [-1.135, 1.287] | [-1.054, 1.138] | [-1.848, .549] | [-2.022, .635] | [-.712, 1.828] | [-1.079, 2.726] | [-1.699, .831] | [-1.321, 1.297] | [-.996, 2.788] |
|  | (.206) | (.902) | (.940) | (.288) | (.306) | (.389) | (.396) | (.501) | (.986) | (.353) |
| Florida | -.115 | .144 | -.176 | -.249 | -.520 | .080 | -.244 | .024 | .038 | -.668 |
|  | .306 | .351 | .350 | .367 | .401 | .348 | .310 | .331 | .345 | .334 |
|  | [-.715, .484] | [-.544, .831] | [-.863, .510] | [-.968, .470] | [-1.307, .266] | [-.602, .762] | [-.852, .365] | [-.624, .672] | [-.638, .713] | [-1.323, -.013] |
|  | (.706) | (.682) | (.615) | (.498) | (.195) | (.817) | (.432) | (.941) | (.913) | (.045) |
| Georgia | -.290 | .179 | -.428 | -.232 | -.762 | .026 | -.154 | .004 | -.238 | -.335 |
|  | .330 | .365 | .378 | .395 | .416 | .360 | .325 | .353 | .368 | .358 |
|  | [-.938, .358] | [-.536, .894] | [-1.168, .312] | [-1.006, .542] | [-1.578, .054] | [-.681, .732] | [-.792, .483] | [-.689, .696] | [-.959, .483] | [-1.037, .367] |
|  | (.380) | (.623) | (.257) | (.556) | (.067) | (.944) | (.635) | (.991) | (.518) | (.349) |
| Hawaii | .800 | .120 | -.050 | -.918 | -.116 | .566 | -.637 | .047 | -.319 | -.254 |
|  | .687 | .554 | .576 | 1.003 | .841 | .527 | .569 | .624 | .651 | .625 |
|  | [-.548, 2.147] | [-.965, 1.206] | [-1.178, 1.079] | [-2.883, 1.048] | [-1.765, 1.534] | [-.466, 1.598] | [-1.753, .479] | [-1.177, 1.270] | [-1.595, .957] | [-1.479, .971] |
|  | (.245) | (.828) | (.931) | (.360) | (.891) | (.282) | (.263) | (.940) | (.624) | (.684) |
| Idaho | -.419 | -.040 | -.209 | -.280 | -1.014 | -.592 | -.761 | .186 | -.748 | -.036 |
|  | .424 | .610 | .512 | .515 | .503 | .538 | .447 | .490 | .491 | .526 |
|  | [-1.250, .412] | [-1.236, 1.157] | [-1.212, .795] | [-1.290, .730] | [-2.000, -.029] | [-1.647, .463] | [-1.636, .114] | [-.773, 1.146] | [-1.711, .215] | [-1.067, .995] |
|  | (.323) | (.948) | (.684) | (.586) | (.044) | (.271) | (.088) | (.704) | (.128) | (.946) |
| Illinois | -.326 | .293 | -.315 | .239 | -.245 | .234 | .105 | .219 | -.241 | -.124 |
|  | .337 | .371 | .374 | .388 | .424 | .373 | .341 | .365 | .368 | .361 |
|  | [-.985, .334] | [-.434, 1.021] | [-1.048, .418] | [-.521, .999] | [-1.076, .587] | [-.497, .965] | [-.565, .774] | [-.496, .934] | [-.962, .480] | [-.831, .582] |
|  | (.333) | (.429) | (.400) | (.538) | (.565) | (.531) | (.759) | (.549) | (.512) | (.730) |
| Indiana | -.027 | -.038 | .040 | -.030 | -.374 | .058 | -.051 | -.278 | .010 | -.202 |
|  | .368 | .403 | .406 | .417 | .446 | .407 | .402 | .389 | .419 | .397 |
|  | [-.748, .695] | [-.828, .751] | [-.757, .836] | [-.847, .787] | [-1.247, .500] | [-.740, .857] | [-.839, .736] | [-1.041, .485] | [-.812, .831] | [-.981, .577] |
|  | (.942) | (.925) | (.922) | (.943) | (.402) | (.886) | (.898) | (.475) | (.981) | (.612) |
| Iowa | -.212 | .539 | -.421 | .059 | -.781 | .528 | .156 | .101 | -.326 | -.163 |
|  | .413 | .447 | .470 | .504 | .502 | .505 | .492 | .437 | .456 | .465 |
|  | [-1.020, .597] | [-.336, 1.414] | [-1.342, .501] | [-.928, 1.046] | [-1.766, .203] | [-.461, 1.517] | [-.808, 1.119] | [-.755, .958] | [-1.218, .567] | [-1.073, .748] |
|  | (.608) | (.227) | (.371) | (.907) | (.120) | (.295) | (.752) | (.816) | (.475) | (.726) |
| Kansas | -.810 | -.501 | -.332 | .187 | -.272 | .375 | -.267 | -.599 | -.658 | -.882 |
|  | .597 | .525 | .527 | .485 | .580 | .498 | .512 | .488 | .747 | .594 |
|  | [-1.980, .360] | [-1.529, .528] | [-1.365, .701] | [-.764, 1.137] | [-1.409, .864] | [-.602, 1.351] | [-1.271, .737] | [-1.556, .358] | [-2.122, .807] | [-2.046, .283] |
|  | (.175) | (.340) | (.529) | (.700) | (.639) | (.452) | (.602) | (.220) | (.379) | (.138) |
| Kentucky | -.230 | .512 | -.060 | .363 | -.149 | .307 | .669 | .017 | .255 | -.350 |
|  | .415 | .413 | .441 | .519 | .514 | .424 | .459 | .394 | .431 | .409 |
|  | [-1.043, .583] | [-.298, 1.321] | [-.923, .804] | [-.654, 1.381] | [-1.158, .859] | [-.525, 1.138] | [-.232, 1.569] | [-.757, .790] | [-.590, 1.099] | [-1.151, .452] |
|  | (.580) | (.216) | (.893) | (.484) | (.772) | (.470) | (.146) | (.966) | (.554) | (.393) |
| Louisiana | -.377 | .574 | -.143 | -.497 | -.381 | .670 | .137 | .178 | -.095 | .011 |
|  | .459 | .445 | .469 | .447 | .509 | .429 | .422 | .381 | .437 | .490 |
|  | [-1.276, .523] | [-.299, 1.447] | [-1.063, .776] | [-1.373, .379] | [-1.380, .617] | [-.170, 1.511] | [-.689, .964] | [-.568, .924] | [-.952, .762] | [-.949, .971] |
|  | (.412) | (.198) | (.760) | (.266) | (.454) | (.118) | (.745) | (.640) | (.829) | (.982) |
| Maine | -.661 | .417 | .259 | .114 | .620 | .307 | 1.341 | -.174 | -.729 | -.630 |
|  | .615 | .583 | .609 | .507 | .718 | .494 | .726 | .687 | .642 | .557 |
|  | [-1.866, .543] | [-.727, 1.560] | [-.935, 1.454] | [-.879, 1.108] | [-.788, 2.027] | [-.661, 1.274] | [-.081, 2.763] | [-1.519, 1.172] | [-1.988, .530] | [-1.721, .462] |
|  | (.282) | (.475) | (.670) | (.821) | (.388) | (.534) | (.065) | (.800) | (.257) | (.258) |
| Maryland | .188 | -.153 | -.916 | -.551 | -.484 | .063 | -.235 | -.344 | -.145 | -.285 |
|  | .384 | .453 | .446 | .474 | .474 | .482 | .432 | .447 | .415 | .432 |
|  | [-.564, .940] | [-1.040, .735] | [-1.790, -.042] | [-1.481, .378] | [-1.412, .445] | [-.881, 1.007] | [-1.083, .612] | [-1.220, .532] | [-.960, .669] | [-1.132, .563] |
|  | (.624) | (.736) | (.040) | (.245) | (.307) | (.896) | (.586) | (.442) | (.726) | (.510) |
| Massachusetts | -.174 | -.209 | -.092 | -.426 | -.660 | .148 | -.058 | -.607 | .001 | -.025 |
|  | .369 | .391 | .390 | .427 | .454 | .420 | .366 | .381 | .385 | .393 |
|  | [-.898, .550] | [-.975, .558] | [-.856, .672] | [-1.263, .411] | [-1.550, .230] | [-.675, .971] | [-.775, .659] | [-1.355, .140] | [-.754, .755] | [-.795, .746] |
|  | (.638) | (.594) | (.813) | (.318) | (.146) | (.725) | (.873) | (.111) | (.999) | (.950) |
| Michigan | -.194 | .312 | -.192 | -.165 | -.550 | .140 | .038 | -.165 | -.304 | -.572 |
|  | .347 | .370 | .372 | .392 | .425 | .365 | .342 | .345 | .358 | .359 |
|  | [-.873, .486] | [-.413, 1.036] | [-.920, .537] | [-.934, .604] | [-1.383, .282] | [-.575, .856] | [-.632, .708] | [-.840, .511] | [-1.005, .397] | [-1.275, .130] |
|  | (.576) | (.399) | (.606) | (.674) | (.195) | (.701) | (.912) | (.633) | (.396) | (.110) |
| Minnesota | -.448 | .291 | -.943 | .081 | -.510 | -.064 | -.015 | -.529 | -.558 | -.931 |
|  | .412 | .469 | .443 | .425 | .491 | .452 | .431 | .405 | .435 | .404 |
|  | [-1.255, .358] | [-.629, 1.211] | [-1.812, -.075] | [-.752, .915] | [-1.472, .453] | [-.950, .821] | [-.860, .830] | [-1.322, .265] | [-1.411, .295] | [-1.724, -.138] |
|  | (.276) | (.535) | (.033) | (.848) | (.299) | (.887) | (.972) | (.192) | (.200) | (.021) |
| Mississippi | -.179 | .303 | -1.054 | .002 | -.656 | -.396 | .369 | -.077 | -.568 | -.058 |
|  | .412 | .435 | .488 | .500 | .502 | .480 | .464 | .434 | .426 | .477 |
|  | [-.988, .629] | [-.550, 1.156] | [-2.011, -.097] | [-.978, .981] | [-1.639, .327] | [-1.338, .545] | [-.541, 1.279] | [-.927, .773] | [-1.403, .268] | [-.993, .877] |
|  | (.664) | (.486) | (.031) | (.997) | (.191) | (.409) | (.427) | (.858) | (.183) | (.904) |
| Missouri | -.428 | .629 | -.236 | .165 | -.419 | .419 | .052 | .008 | -.232 | -.067 |
|  | .361 | .410 | .404 | .414 | .444 | .392 | .362 | .363 | .379 | .385 |
|  | [-1.137, .280] | [-.175, 1.434] | [-1.028, .555] | [-.647, .977] | [-1.290, .452] | [-.351, 1.188] | [-.658, .762] | [-.703, .720] | [-.975, .511] | [-.820, .687] |
|  | (.236) | (.125) | (.559) | (.690) | (.346) | (.286) | (.886) | (.982) | (.541) | (.863) |
| Montana | .254 | -.453 | -.805 | -.235 | -1.834 | -1.376 | -1.387 | -.553 | -.939 | -1.643 |
|  | .466 | .667 | .504 | .529 | .661 | .590 | .572 | .590 | .567 | .626 |
|  | [-.659, 1.167] | [-1.761, .855] | [-1.794, .183] | [-1.272, .803] | [-3.130, -.538] | [-2.532, -.220] | [-2.509, -.266] | [-1.708, .603] | [-2.049, .172] | [-2.870, -.416] |
|  | (.585) | (.497) | (.110) | (.658) | (.006) | (.020) | (.015) | (.348) | (.097) | (.009) |
| Nebraska | -.181 | .356 | -.128 | .855 | -.337 | -.439 | -.296 | -.408 | -.130 | -.355 |
|  | .763 | .616 | .575 | .736 | .691 | .661 | .524 | .706 | .566 | .682 |
|  | [-1.676, 1.314] | [-.851, 1.563] | [-1.255, .999] | [-.588, 2.299] | [-1.692, 1.018] | [-1.735, .856] | [-1.323, .730] | [-1.792, .976] | [-1.240, .980] | [-1.692, .982] |
|  | (.812) | (.563) | (.824) | (.245) | (.626) | (.506) | (.572) | (.564) | (.819) | (.602) |
| Nevada | -.204 | -.007 | -.048 | -.073 | -.354 | .050 | -.085 | -.417 | -.410 | -.697 |
|  | .381 | .412 | .442 | .453 | .482 | .433 | .415 | .415 | .419 | .406 |
|  | [-.952, .543] | [-.816, .801] | [-.914, .817] | [-.961, .814] | [-1.298, .590] | [-.799, .899] | [-.898, .727] | [-1.231, .397] | [-1.232, .412] | [-1.493, .099] |
|  | (.592) | (.986) | (.913) | (.872) | (.462) | (.908) | (.837) | (.316) | (.328) | (.086) |
| New Hampshire | -.168 | -.130 | -.373 | .189 | -.397 | .173 | .185 | .105 | .817 | -.882 |
|  | .505 | .511 | .648 | .495 | .489 | .471 | .481 | .520 | .504 | .566 |
|  | [-1.158, .823] | [-1.131, .872] | [-1.642, .896] | [-.781, 1.159] | [-1.356, .562] | [-.751, 1.096] | [-.758, 1.129] | [-.915, 1.125] | [-.171, 1.804] | [-1.991, .227] |
|  | (.740) | (.800) | (.565) | (.703) | (.418) | (.714) | (.700) | (.840) | (.105) | (.119) |
| New Jersey | -.345 | -.172 | -.150 | -.458 | -.209 | .127 | -.063 | -.232 | .021 | -.406 |
|  | .390 | .454 | .381 | .426 | .457 | .421 | .384 | .397 | .373 | .404 |
|  | [-1.109, .418] | [-1.061, .718] | [-.898, .597] | [-1.292, .376] | [-1.105, .687] | [-.699, .953] | [-.816, .690] | [-1.010, .546] | [-.710, .752] | [-1.197, .385] |
|  | (.376) | (.705) | (.693) | (.282) | (.648) | (.763) | (.869) | (.559) | (.955) | (.315) |
| New Mexico | -.288 | 1.124 | -.061 | .405 | -.181 | .248 | .651 | .183 | -.075 | -.409 |
|  | .499 | .503 | .487 | .536 | .531 | .467 | .483 | .453 | .547 | .466 |
|  | [-1.267, .691] | [.139, 2.110] | [-1.016, .893] | [-.645, 1.455] | [-1.222, .860] | [-.667, 1.163] | [-.295, 1.597] | [-.704, 1.070] | [-1.147, .996] | [-1.322, .505] |
|  | (.565) | (.025) | (.900) | (.449) | (.733) | (.595) | (.178) | (.686) | (.891) | (.381) |
| New York | -.255 | .459 | -.417 | .009 | -.170 | .293 | .266 | .218 | .041 | -.305 |
|  | .322 | .355 | .358 | .372 | .411 | .353 | .327 | .339 | .350 | .342 |
|  | [-.886, .376] | [-.237, 1.155] | [-1.119, .285] | [-.719, .738] | [-.975, .635] | [-.398, .984] | [-.375, .907] | [-.448, .883] | [-.645, .728] | [-.975, .364] |
|  | (.428) | (.196) | (.244) | (.980) | (.679) | (.406) | (.416) | (.521) | (.906) | (.371) |
| North Carolina | -.169 | .099 | -.339 | -.163 | .165 | .284 | .135 | .071 | -.305 | -.238 |
|  | .350 | .377 | .392 | .413 | .427 | .378 | .350 | .355 | .384 | .382 |
|  | [-.855, .518] | [-.639, .838] | [-1.107, .430] | [-.972, .646] | [-.671, 1.001] | [-.458, 1.025] | [-.551, .821] | [-.626, .767] | [-1.057, .447] | [-.986, .511] |
|  | (.630) | (.792) | (.388) | (.692) | (.699) | (.454) | (.699) | (.842) | (.427) | (.533) |
| North Dakota | .351 | .013 | -1.021 | -.701 | -1.052 | -1.185 | -.956 | .111 | -.262 | -1.924 |
|  | .510 | .681 | .688 | .811 | .850 | .578 | .704 | .507 | .643 | .702 |
|  | [-.648, 1.349] | [-1.322, 1.348] | [-2.370, .328] | [-2.291, .889] | [-2.719, .615] | [-2.319, -.051] | [-2.336, .423] | [-.883, 1.105] | [-1.523, .998] | [-3.301, -.548] |
|  | (.492) | (.984) | (.138) | (.387) | (.216) | (.040) | (.174) | (.826) | (.683) | (.006) |
| Ohio | -.107 | .419 | -.352 | .133 | -.202 | .354 | .020 | -.505 | -.286 | .099 |
|  | .333 | .365 | .374 | .393 | .422 | .376 | .336 | .363 | .368 | .358 |
|  | [-.759, .545] | [-.296, 1.134] | [-1.085, .382] | [-.636, .903] | [-1.029, .626] | [-.383, 1.090] | [-.638, .678] | [-1.217, .207] | [-1.007, .434] | [-.603, .801] |
|  | (.748) | (.250) | (.347) | (.735) | (.633) | (.347) | (.952) | (.165) | (.436) | (.783) |
| Oklahoma | -.411 | .001 | -1.022 | -.158 | -.099 | -.466 | -.341 | .068 | -.295 | -.819 |
|  | .509 | .487 | .525 | .545 | .556 | .511 | .506 | .452 | .538 | .473 |
|  | [-1.408, .585] | [-.954, .956] | [-2.051, .008] | [-1.227, .910] | [-1.189, .991] | [-1.467, .535] | [-1.333, .652] | [-.819, .954] | [-1.348, .759] | [-1.747, .109] |
|  | (.419) | (.998) | (.052) | (.772) | (.859) | (.362) | (.501) | (.881) | (.583) | (.084) |
| Oregon | -.374 | .044 | -.438 | .168 | -1.303 | .044 | -.501 | -.877 | .468 | -.522 |
|  | .377 | .407 | .415 | .425 | .462 | .419 | .360 | .385 | .399 | .382 |
|  | [-1.113, .364] | [-.753, .842] | [-1.251, .375] | [-.665, 1.002] | [-2.209, -.398] | [-.776, .865] | [-1.207, .204] | [-1.631, -.123] | [-.313, 1.250] | [-1.270, .226] |
|  | (.320) | (.914) | (.291) | (.692) | (.005) | (.916) | (.164) | (.023) | (.240) | (.172) |
| Pennsylvania | -.239 | .174 | -.268 | .086 | -.041 | .240 | .367 | -.376 | -.134 | -.168 |
|  | .304 | .350 | .357 | .373 | .409 | .358 | .320 | .333 | .345 | .336 |
|  | [-.835, .357] | [-.511, .859] | [-.969, .432] | [-.645, .816] | [-.842, .760] | [-.462, .942] | [-.260, .993] | [-1.030, .277] | [-.811, .542] | [-.826, .490] |
|  | (.432) | (.619) | (.453) | (.818) | (.920) | (.503) | (.251) | (.259) | (.697) | (.617) |
| Rhode Island | .259 | 1.560 | -.028 | .296 | .192 | .317 | .444 | .567 | -.291 | -.208 |
|  | 1.074 | .819 | .536 | 1.006 | .699 | .758 | .841 | .703 | .801 | .805 |
|  | [-1.845, 2.364] | [-.045, 3.165] | [-1.079, 1.022] | [-1.676, 2.268] | [-1.179, 1.562] | [-1.168, 1.802] | [-1.203, 2.092] | [-.810, 1.945] | [-1.862, 1.279] | [-1.786, 1.370] |
|  | (.809) | (.057) | (.958) | (.769) | (.784) | (.676) | (.597) | (.419) | (.716) | (.796) |
| South Carolina | -.148 | -.243 | -.238 | .022 | .040 | .359 | .415 | -.647 | .021 | -.259 |
|  | .422 | .418 | .431 | .480 | .500 | .422 | .454 | .424 | .458 | .411 |
|  | [-.975, .680] | [-1.062, .575] | [-1.084, .607] | [-.919, .962] | [-.941, 1.021] | [-.467, 1.185] | [-.476, 1.305] | [-1.478, .183] | [-.877, .918] | [-1.064, .546] |
|  | (.727) | (.560) | (.581) | (.964) | (.936) | (.395) | (.361) | (.127) | (.964) | (.529) |
| South Dakota | -.130 | .350 | -.770 | .083 | -.336 | .209 | -.504 | -1.121 | -.295 | -.447 |
|  | .561 | .602 | .554 | .580 | .663 | .699 | .649 | .698 | .572 | .449 |
|  | [-1.230, .970] | [-.831, 1.531] | [-1.856, .316] | [-1.054, 1.220] | [-1.636, .965] | [-1.161, 1.579] | [-1.777, .769] | [-2.489, .248] | [-1.416, .826] | [-1.327, .432] |
|  | (.817) | (.561) | (.165) | (.886) | (.613) | (.765) | (.438) | (.108) | (.606) | (.319) |
| Tennessee | -.292 | -.303 | -.505 | -.499 | -.870 | -.303 | -.005 | -.156 | -.008 | -.592 |
|  | .378 | .406 | .415 | .437 | .456 | .414 | .407 | .441 | .429 | .415 |
|  | [-1.033, .449] | [-1.098, .492] | [-1.318, .308] | [-1.355, .357] | [-1.764, .024] | [-1.115, .508] | [-.803, .794] | [-1.020, .708] | [-.849, .832] | [-1.405, .221] |
|  | (.440) | (.455) | (.223) | (.253) | (.057) | (.464) | (.991) | (.724) | (.985) | (.154) |
| Texas | -.167 | .135 | -.588 | -.244 | -.414 | .041 | .055 | -.121 | -.247 | -.597 |
|  | .317 | .355 | .353 | .373 | .410 | .357 | .320 | .335 | .351 | .349 |
|  | [-.788, .453] | [-.561, .832] | [-1.280, .105] | [-.974, .486] | [-1.218, .390] | [-.659, .741] | [-.572, .682] | [-.777, .535] | [-.935, .441] | [-1.281, .086] |
|  | (.597) | (.703) | (.096) | (.513) | (.313) | (.909) | (.864) | (.718) | (.481) | (.087) |
| Utah | .122 | -.390 | -.740 | -.651 | -.634 | -.193 | -.094 | -.975 | .149 | -1.296 |
|  | .448 | .476 | .506 | .455 | .483 | .411 | .455 | .521 | .506 | .541 |
|  | [-.757, 1.000] | [-1.323, .542] | [-1.731, .251] | [-1.542, .241] | [-1.581, .313] | [-.998, .612] | [-.985, .797] | [-1.997, .047] | [-.842, 1.141] | [-2.357, -.234] |
|  | (.786) | (.412) | (.143) | (.153) | (.190) | (.638) | (.836) | (.061) | (.768) | (.017) |
| Vermont | .403 | -.168 | -.131 | .059 | -.717 | .486 | -.252 | -1.242 | -.604 | -1.728 |
|  | .464 | .678 | .579 | .650 | .590 | .808 | .638 | .643 | .752 | .636 |
|  | [-.507, 1.312] | [-1.498, 1.162] | [-1.265, 1.004] | [-1.215, 1.333] | [-1.873, .439] | [-1.098, 2.071] | [-1.502, .999] | [-2.502, .018] | [-2.078, .869] | [-2.975, -.481] |
|  | (.385) | (.805) | (.822) | (.927) | (.224) | (.547) | (.693) | (.053) | (.421) | (.007) |
| Virginia | -.097 | .209 | -.377 | -.117 | -.586 | .209 | -.020 | -.452 | .055 | -.667 |
|  | .334 | .374 | .374 | .411 | .428 | .379 | .337 | .368 | .376 | .370 |
|  | [-.751, .557] | [-.524, .941] | [-1.111, .356] | [-.922, .688] | [-1.424, .252] | [-.534, .953] | [-.681, .640] | [-1.173, .269] | [-.682, .793] | [-1.393, .059] |
|  | (.772) | (.577) | (.313) | (.776) | (.170) | (.581) | (.952) | (.219) | (.883) | (.072) |
| Washington | -.251 | -.299 | -.191 | -.376 | -1.214 | -.294 | -.755 | -.509 | -.398 | -1.131 |
|  | .368 | .397 | .384 | .415 | .438 | .392 | .347 | .364 | .399 | .371 |
|  | [-.972, .470] | [-1.076, .478] | [-.943, .561] | [-1.190, .438] | [-2.072, -.357] | [-1.062, .475] | [-1.436, -.075] | [-1.222, .205] | [-1.180, .385] | [-1.858, -.404] |
|  | (.495) | (.451) | (.619) | (.365) | (.006) | (.454) | (.030) | (.163) | (.319) | (.002) |
| West Virginia | -.201 | -.002 | -.312 | -.711 | .053 | .527 | .347 | .090 | .022 | -.594 |
|  | .501 | .439 | .490 | .507 | .563 | .571 | .556 | .451 | .465 | .488 |
|  | [-1.183, .782] | [-.863, .859] | [-1.273, .649] | [-1.705, .282] | [-1.051, 1.156] | [-.592, 1.646] | [-.744, 1.437] | [-.794, .974] | [-.889, .932] | [-1.550, .362] |
|  | (.689) | (.996) | (.525) | (.161) | (.926) | (.356) | (.533) | (.841) | (.963) | (.223) |
| Wisconsin | .036 | -.042 | -.592 | -.106 | -.411 | -.032 | -.134 | -.270 | -.597 | -.486 |
|  | .354 | .379 | .398 | .431 | .449 | .379 | .363 | .373 | .380 | .367 |
|  | [-.658, .730] | [-.785, .700] | [-1.372, .188] | [-.950, .739] | [-1.291, .470] | [-.775, .711] | [-.845, .578] | [-1.002, .461] | [-1.340, .147] | [-1.205, .233] |
|  | (.918) | (.911) | (.137) | (.806) | (.361) | (.933) | (.712) | (.469) | (.116) | (.185) |
| Wyoming | -.876 | 1.543 | .529 | -.545 | .853 | .097 | 13.873 | 1.644 | 1.870 | -.532 |
|  | 2.573 | .854 | 1.701 | 1.638 | .923 | 2.436 | .749 | 1.965 | 1.263 | 1.373 |
|  | [-5.919, 4.167] | [-.130, 3.216] | [-2.804, 3.862] | [-3.756, 2.666] | [-.956, 2.662] | [-4.676, 4.871] | [12.405,15.341] | [-2.208, 5.496] | [-.605, 4.346] | [-3.223, 2.159] |
|  | (.734) | (.071) | (.756) | (.739) | (.356) | (.968) | (<.001) | (.403) | (.139) | (.698) |
| cut1 | -4.048 | -2.542 | -4.075 | -4.336 | -3.320 | -3.865 | -4.137 | -2.577 | -2.750 | -1.960 |
|  | .404 | .449 | .452 | .452 | .466 | .443 | .403 | .403 | .443 | .430 |
|  | [-4.840, -3.255] | [-3.422, -1.662] | [-4.961, -3.189] | [-5.221, -3.451] | [-4.233, -2.407] | [-4.733, -2.997] | [-4.926, -3.348] | [-3.367, -1.787] | [-3.619, -1.881] | [-2.803, -1.118] |
|  | (<.001) | (<.001) | (<.001) | (<.001) | (<.001) | (<.001) | (<.001) | (<.001) | (<.001) | (<.001) |
| cut2 | -2.949 | -1.474 | -2.870 | -3.147 | -2.050 | -2.837 | -3.164 | -1.823 | -1.558 | -1.324 |
|  | .401 | .448 | .439 | .444 | .460 | .435 | .398 | .402 | .438 | .429 |
|  | [-3.736, -2.162] | [-2.351, -.597] | [-3.730, -2.011] | [-4.018, -2.276] | [-2.952, -1.147] | [-3.690, -1.985] | [-3.945, -2.383] | [-2.611, -1.035] | [-2.417, -.698] | [-2.164, -.484] |
|  | (<.001) | (.001) | (<.001) | (<.001) | (<.001) | (<.001) | (<.001) | (<.001) | (<.001) | (.002) |
| cut3 | -1.120 | .348 | -1.262 | -1.891 | -1.000 | -1.558 | -1.974 | -.599 | -.074 | -.468 |
|  | .398 | .446 | .438 | .443 | .460 | .433 | .399 | .402 | .438 | .429 |
|  | [-1.900, -.340] | [-.527, 1.223] | [-2.121, -.403] | [-2.759, -1.023] | [-1.901, -.099] | [-2.407, -.709] | [-2.755, -1.192] | [-1.387, .189] | [-.933, .785] | [-1.308, .372] |
|  | (.005) | (.436) | (.004) | (<.001) | (.030) | (<.001) | (<.001) | (.136) | (.866) | (.275) |
| cut4 | .355 | 1.668 | .067 | -.629 | .151 | -.452 | -.873 | .144 | 1.155 | .608 |
|  | .398 | .449 | .438 | .441 | .460 | .432 | .398 | .404 | .440 | .429 |
|  | [-.425, 1.135] | [.788, 2.547] | [-.790, .925] | [-1.494, .235] | [-.750, 1.051] | [-1.299, .395] | [-1.653, -.093] | [-.649, .937] | [.292, 2.017] | [-.233, 1.448] |
|  | (.372) | (<.001) | (.878) | (.154) | (.743) | (.295) | (.028) | (.722) | (.009) | (.157) |
| N | 2967 | 2973 | 2969 | 2970 | 2976 | 2973 | 2972 | 2967 | 2970 | 2975 |
| Pseudo-R^2^ | .0453 | .0456 | .0215 | .0596 | .0399 | .0450 | .0829 | .0555 | .0145 | .0319 |
| Model χ^2^ | 399.1 | 416.1 | 176.4 | 443.2 | 333.2 | 358.8 | 1074.8 | 476.4 | 131.8 | 303.4 |
| d.f. | 87 | 87 | 87 | 87 | 87 | 87 | 87 | 87 | 87 | 87 |
| p-value | <.001 | <.001 | <.001 | <.001 | <.001 | <.001 | <.001 | <.001 | <.001 | <.001 |

# Different Measures of Partisanship

**S1 Fig. Behaviors and Attitudes, by Trump Support.** Estimates are odds-ratios comparing those who will vote for the Democratic candidate and others who will vote for another candidate or not vote at all to those who intend to vote for Trump. Odds ratios greater than 1 imply the respondent is more likely report a behavior or to express a view. 95% confidence intervals are adjusted for nine comparisons using a Bonferroni correction. For each dependent variable, the number in parentheses is the proportion saying yes or the mean response on a five-point scale.

**S2 Fig. Behaviors and Attitudes, by Ideology.** Estimates are odds-ratios comparing moderates and liberals to conservatives. Odds ratios greater than 1 imply the respondent is more likely report a behavior or to express a view. 95% confidence intervals are adjusted for nine comparisons using a Bonferroni correction. For each dependent variable, the number in parentheses is the proportion saying yes or the mean response on a five-point scale.

**S3 Fig. Policies, by Trump Support.** Odds ratios greater than 1 imply the respondent is more likely to support a policy. 95% confidence intervals are adjusted for twenty comparisons using a Bonferroni correction. For each dependent variable, the number in parentheses is the mean response on a five-point scale.

**S4 Fig. Policies, by Ideology.** Odds ratios greater than 1 imply the respondent is more likely to support a policy. 95% confidence intervals are adjusted for twenty comparisons using a Bonferroni correction. For each dependent variable, the number in parentheses is the mean response on a five-point scale.

# Multilevel Models

In this section we present results from multilevel models that differ from the main specifications in the text as follows: in addition to state fixed effects, we include random effects by ZIP code and include county-level deaths from COVID-19 as of March 23 and positive diagnoses of COVID-19 as of March 23 as predictors. Each of these county-level predictors was matched to respondent’s ZIP code following the same procedure through which we matched urban-rural codes to respondent ZIP codes. We estimate linear multilevel models throughout.

Our main conclusions are two: *first*, county-level predictors for cases and deaths are never significantly correlated with any dependent variable, and *second*, their inclusion never changes our inferences about the partisanship variables.

S7 Table. Regression Results, Health Behavior, Multilevel Model.

|  | (1) | (2) | (3) | (4) | (5) | (6) | (7) | (8) | (9) |
| --- | --- | --- | --- | --- | --- | --- | --- | --- | --- |
|  | washhands | boughtsanitizer | visitdoctor | changedtravel | avoidcontact | avoidgatherings | soughtinfo | selfquarantine | changehealth |
| main |  |  |  |  |  |  |  |  |  |
| Democrat | .084 | .084 | -.006 | .064 | .170 | .109 | .192 | .119 | .815 |
|  | .017 | .023 | .010 | .022 | .022 | .020 | .023 | .023 | .088 |
|  | [.052, .117] | [.038, .129] | [-.026, .014] | [.022, .107] | [.126, .214] | [.070, .147] | [.147, .237] | [.074, .163] | [.642, .987] |
|  | (<.001) | (<.001) | (.538) | (.003) | (<.001) | (<.001) | (<.001) | (<.001) | (<.001) |
| Other | -.011 | -.004 | -.020 | -.003 | .049 | .024 | .070 | .029 | .133 |
|  | .017 | .024 | .010 | .022 | .023 | .020 | .023 | .023 | .089 |
|  | [-.044, .022] | [-.050, .042] | [-.040, .000] | [-.046, .040] | [.004, .093] | [-.015, .063] | [.024, .116] | [-.016, .074] | [-.042, .308] |
|  | (.526) | (.866) | (.053) | (.894) | (.032) | (.232) | (.003) | (.205) | (.135) |
| 35- | .007 | .051 | .001 | -.049 | .001 | -.011 | -.008 | -.043 | -.053 |
|  | .019 | .026 | .011 | .025 | .025 | .022 | .026 | .025 | .099 |
|  | [-.030, .044] | [-<.001, .102] | [-.021, .024] | [-.097, -.001] | [-.048, .050] | [-.055, .032] | [-.059, .043] | [-.093, .006] | [-.246, .141] |
|  | (.707) | (.052) | (.918) | (.047) | (.975) | (.609) | (.753) | (.088) | (.593) |
| 50- | .024 | .051 | -.008 | -.066 | .017 | .052 | -.034 | -.072 | -.037 |
|  | .018 | .025 | .011 | .023 | .024 | .021 | .025 | .024 | .093 |
|  | [-.010, .059] | [.003, .099] | [-.029, .013] | [-.112, -.021] | [-.029, .064] | [.011, .093] | [-.082, .014] | [-.119, -.025] | [-.219, .146] |
|  | (.169) | (.039) | (.470) | (.004) | (.470) | (.013) | (.167) | (.003) | (.693) |
| 70- | .060 | .067 | -.012 | -.024 | .103 | .140 | -.070 | .063 | .326 |
|  | .022 | .031 | .014 | .029 | .030 | .027 | .031 | .030 | .118 |
|  | [.016, .104] | [.006, .128] | [-.038, .015] | [-.081, .033] | [.044, .162] | [.088, .192] | [-.131, -.009] | [.004, .123] | [.095, .558] |
|  | (.007) | (.031) | (.393) | (.413) | (.001) | (<.001) | (.024) | (.037) | (.006) |
| gender | .041 | .016 | -.002 | -.012 | .007 | .042 | .035 | .060 | .186 |
|  | .013 | .018 | .008 | .017 | .017 | .015 | .018 | .017 | .068 |
|  | [.016, .066] | [-.019, .051] | [-.018, .013] | [-.045, .021] | [-.027, .041] | [.013, .072] | [.000, .071] | [.025, .094] | [.053, .319] |
|  | (.001) | (.371) | (.758) | (.466) | (.702) | (.005) | (.048) | (.001) | (.006) |
| white | -.011 | -.084 | -.031 | -.030 | .043 | .050 | .080 | .003 | .019 |
|  | .015 | .021 | .009 | .020 | .021 | .018 | .021 | .021 | .081 |
|  | [-.041, .019] | [-.126, -.042] | [-.050, -.013] | [-.070, .009] | [.002, .083] | [.014, .086] | [.038, .122] | [-.038, .044] | [-.140, .178] |
|  | (.472) | (<.001) | (.001) | (.132) | (.038) | (.006) | (<.001) | (.899) | (.816) |
| married | -.002 | .046 | .014 | .043 | .032 | -.004 | .021 | .011 | .161 |
|  | .014 | .020 | .009 | .019 | .019 | .017 | .020 | .019 | .075 |
|  | [-.030, .025] | [.007, .084] | [-.002, .031] | [.007, .080] | [-.005, .069] | [-.037, .028] | [-.018, .059] | [-.026, .049] | [.015, .307] |
|  | (.865) | (.021) | (.094) | (.019) | (.093) | (.793) | (.288) | (.550) | (.031) |
| High school graduate | -.015 | .006 | -.001 | .081 | -.121 | -.038 | .002 | .002 | -.084 |
|  | .038 | .053 | .023 | .049 | .051 | .045 | .052 | .051 | .199 |
|  | [-.088, .059] | [-.098, .109] | [-.047, .044] | [-.016, .178] | [-.220, -.021] | [-.125, .050] | [-.101, .105] | [-.099, .102] | [-.474, .306] |
|  | (.700) | (.916) | (.952) | (.101) | (.017) | (.399) | (.974) | (.971) | (.674) |
| Some college | .025 | -.010 | -.017 | .168 | -.060 | .024 | .138 | .017 | .286 |
|  | .038 | .054 | .024 | .050 | .052 | .046 | .054 | .052 | .203 |
|  | [-.051, .100] | [-.115, .096] | [-.063, .029] | [.069, .266] | [-.161, .041] | [-.065, .114] | [.033, .243] | [-.086, .119] | [-.112, .684] |
|  | (.521) | (.856) | (.477) | (.001) | (.246) | (.597) | (.010) | (.749) | (.159) |
| 2-year | .019 | .007 | -.006 | .123 | -.067 | .018 | .127 | -.007 | .217 |
|  | .040 | .056 | .025 | .053 | .054 | .048 | .056 | .055 | .213 |
|  | [-.060, .099] | [-.103, .118] | [-.055, .042] | [.019, .227] | [-.173, .039] | [-.076, .112] | [.017, .238] | [-.114, .101] | [-.201, .635] |
|  | (.632) | (.898) | (.794) | (.020) | (.217) | (.701) | (.023) | (.904) | (.309) |
| 4-year | .067 | -.030 | .014 | .201 | -.036 | .056 | .158 | .029 | .461 |
|  | .039 | .055 | .024 | .051 | .053 | .047 | .055 | .053 | .207 |
|  | [-.010, .144] | [-.137, .078] | [-.033, .061] | [.100, .302] | [-.140, .067] | [-.035, .147] | [.051, .265] | [-.076, .133] | [.055, .867] |
|  | (.087) | (.590) | (.561) | (<.001) | (.489) | (.228) | (.004) | (.593) | (.026) |
| Post-grad | .053 | -.019 | .019 | .304 | .020 | .083 | .229 | .069 | .760 |
|  | .041 | .058 | .025 | .054 | .055 | .049 | .057 | .056 | .218 |
|  | [-.028, .134] | [-.132, .094] | [-.031, .068] | [.198, .410] | [-.088, .129] | [-.013, .179] | [.116, .341] | [-.041, .179] | [.334, 1.187] |
|  | (.201) | (.738) | (.461) | (<.001) | (.714) | (.090) | (<.001) | (.218) | (<.001) |
| income=2 | -.014 | -.038 | -.080 | -.010 | -.001 | -.023 | .023 | -.064 | -.205 |
|  | .036 | .051 | .022 | .048 | .049 | .043 | .051 | .049 | .192 |
|  | [-.085, .057] | [-.138, .061] | [-.123, -.036] | [-.103, .083] | [-.097, .095] | [-.107, .062] | [-.076, .122] | [-.161, .033] | [-.581, .171] |
|  | (.702) | (.450) | (<.001) | (.834) | (.980) | (.602) | (.650) | (.195) | (.286) |
| income=3 | -.033 | -.050 | -.061 | -.024 | -.036 | .009 | .051 | -.127 | -.271 |
|  | .035 | .049 | .022 | .046 | .047 | .042 | .049 | .048 | .186 |
|  | [-.102, .036] | [-.146, .047] | [-.104, -.019] | [-.115, .066] | [-.129, .056] | [-.073, .091] | [-.045, .147] | [-.221, -.033] | [-.635, .093] |
|  | (.345) | (.311) | (.004) | (.597) | (.441) | (.826) | (.302) | (.008) | (.145) |
| income=4 | .019 | .062 | -.067 | -.003 | .037 | .067 | .105 | -.126 | .093 |
|  | .036 | .050 | .022 | .047 | .048 | .043 | .050 | .049 | .190 |
|  | [-.052, .090] | [-.037, .161] | [-.111, -.024] | [-.096, .089] | [-.058, .132] | [-.017, .151] | [.007, .204] | [-.222, -.030] | [-.280, .466] |
|  | (.595) | (.220) | (.002) | (.946) | (.447) | (.119) | (.036) | (.010) | (.624) |
| income=5 | .033 | -.018 | -.065 | -.047 | -.013 | .052 | .123 | -.129 | -.064 |
|  | .037 | .051 | .023 | .048 | .049 | .044 | .051 | .050 | .194 |
|  | [-.039, .106] | [-.119, .083] | [-.109, -.021] | [-.142, .047] | [-.110, .084] | [-.034, .137] | [.022, .223] | [-.227, -.031] | [-.445, .317] |
|  | (.364) | (.726) | (.004) | (.328) | (.793) | (.237) | (.017) | (.010) | (.742) |
| income=6 | .041 | .004 | -.059 | -.015 | .047 | .082 | .073 | -.118 | .058 |
|  | .037 | .052 | .023 | .049 | .050 | .044 | .052 | .051 | .196 |
|  | [-.032, .114] | [-.098, .106] | [-.103, -.014] | [-.111, .080] | [-.051, .145] | [-.004, .169] | [-.029, .174] | [-.217, -.018] | [-.327, .443] |
|  | (.271) | (.937) | (.010) | (.755) | (.344) | (.063) | (.161) | (.020) | (.767) |
| income=7 | .025 | -.009 | -.052 | .110 | <.001 | .079 | .115 | -.148 | .119 |
|  | .040 | .056 | .024 | .052 | .054 | .047 | .055 | .054 | .210 |
|  | [-.053, .103] | [-.118, .100] | [-.100, -.004] | [.008, .212] | [-.105, .105] | [-.014, .171] | [.007, .224] | [-.255, -.042] | [-.293, .532] |
|  | (.526) | (.870) | (.033) | (.035) | (.999) | (.097) | (.038) | (.006) | (.570) |
| income=8 | -.004 | -.079 | -.088 | .056 | -.029 | .048 | .011 | -.080 | -.165 |
|  | .039 | .054 | .024 | .051 | .052 | .046 | .054 | .053 | .205 |
|  | [-.080, .073] | [-.185, .028] | [-.134, -.041] | [-.044, .156] | [-.131, .073] | [-.043, .138] | [-.095, .117] | [-.184, .023] | [-.567, .236] |
|  | (.926) | (.147) | (<.001) | (.271) | (.577) | (.302) | (.840) | (.127) | (.420) |
| income=9 | .073 | .056 | -.082 | .053 | .016 | .049 | .110 | -.149 | .126 |
|  | .038 | .054 | .023 | .050 | .052 | .046 | .053 | .052 | .203 |
|  | [-.002, .148] | [-.049, .161] | [-.128, -.036] | [-.046, .151] | [-.085, .117] | [-.040, .138] | [.005, .215] | [-.252, -.047] | [-.271, .524] |
|  | (.058) | (.295) | (<.001) | (.295) | (.755) | (.283) | (.040) | (.004) | (.533) |
| income=10 | .030 | -.029 | -.085 | .087 | .054 | .074 | .030 | -.142 | .020 |
|  | .040 | .056 | .024 | .052 | .054 | .047 | .056 | .054 | .211 |
|  | [-.048, .109] | [-.139, .080] | [-.133, -.037] | [-.016, .189] | [-.051, .159] | [-.019, .167] | [-.079, .139] | [-.248, -.035] | [-.393, .433] |
|  | (.449) | (.600) | (.001) | (.097) | (.316) | (.118) | (.591) | (.009) | (.925) |
| income=11 | .016 | .044 | -.088 | .109 | .012 | .050 | .071 | -.112 | .103 |
|  | .041 | .057 | .025 | .054 | .055 | .049 | .057 | .056 | .216 |
|  | [-.064, .097] | [-.068, .156] | [-.137, -.039] | [.003, .214] | [-.096, .120] | [-.046, .145] | [-.041, .183] | [-.221, -.003] | [-.321, .527] |
|  | (.694) | (.442) | (<.001) | (.043) | (.822) | (.306) | (.213) | (.044) | (.633) |
| income=12 | .072 | .047 | -.074 | .107 | .062 | .068 | .123 | -.107 | .299 |
|  | .045 | .063 | .028 | .059 | .061 | .054 | .063 | .061 | .238 |
|  | [-.016, .160] | [-.077, .170] | [-.128, -.020] | [-.009, .223] | [-.056, .181] | [-.037, .173] | [.000, .246] | [-.227, .013] | [-.168, .765] |
|  | (.110) | (.460) | (.007) | (.069) | (.304) | (.204) | (.050) | (.080) | (.210) |
| income=13 | .052 | .008 | -.130 | .214 | .067 | .124 | .235 | -.065 | .505 |
|  | .062 | .087 | .038 | .082 | .084 | .074 | .087 | .085 | .329 |
|  | [-.070, .174] | [-.163, .179] | [-.204, -.055] | [.053, .374] | [-.097, .231] | [-.021, .269] | [.065, .405] | [-.231, .101] | [-.140, 1.150] |
|  | (.406) | (.928) | (.001) | (.009) | (.426) | (.094) | (.007) | (.442) | (.125) |
| income=14 | -.010 | .083 | -.030 | .315 | .101 | .101 | .072 | -.033 | .601 |
|  | .066 | .092 | .040 | .087 | .089 | .078 | .092 | .090 | .349 |
|  | [-.139, .120] | [-.097, .264] | [-.109, .049] | [.146, .485] | [-.073, .275] | [-.052, .255] | [-.108, .253] | [-.209, .143] | [-.082, 1.284] |
|  | (.881) | (.366) | (.458) | (<.001) | (.255) | (.196) | (.431) | (.714) | (.085) |
| income=15 | .111 | .181 | .176 | .270 | .313 | .217 | .444 | .186 | 1.906 |
|  | .200 | .280 | .122 | .262 | .269 | .238 | .278 | .272 | 1.056 |
|  | [-.282, .503] | [-.367, .729] | [-.064, .416] | [-.243, .784] | [-.213, .840] | [-.248, .683] | [-.102, .990] | [-.347, .718] | [-.163, 3.975] |
|  | (.580) | (.518) | (.151) | (.302) | (.243) | (.360) | (.111) | (.494) | (.071) |
| income=16 | .132 | .381 | -.058 | .188 | .156 | .081 | .196 | -.114 | .959 |
|  | .094 | .131 | .058 | .123 | .126 | .112 | .131 | .128 | .496 |
|  | [-.052, .316] | [.123, .638] | [-.171, .054] | [-.054, .429] | [-.092, .403] | [-.138, .300] | [-.061, .452] | [-.364, .136] | [-.014, 1.931] |
|  | (.160) | (.004) | (.310) | (.128) | (.217) | (.470) | (.135) | (.372) | (.053) |
| income=17 | -.004 | -.010 | -.096 | .014 | -.010 | .061 | .032 | -.101 | -.114 |
|  | .034 | .048 | .021 | .045 | .046 | .041 | .048 | .046 | .180 |
|  | [-.071, .063] | [-.104, .083] | [-.137, -.055] | [-.074, .101] | [-.100, .080] | [-.018, .141] | [-.061, .125] | [-.192, -.010] | [-.467, .240] |
|  | (.899) | (.831) | (<.001) | (.758) | (.822) | (.131) | (.497) | (.030) | (.529) |
| Rural Code=2 | -.018 | -.031 | -.013 | -<.001 | .036 | .018 | -.006 | -.015 | -.029 |
|  | .017 | .024 | .010 | .022 | .023 | .020 | .024 | .023 | .090 |
|  | [-.051, .015] | [-.078, .016] | [-.034, .007] | [-.045, .044] | [-.009, .081] | [-.022, .057] | [-.052, .041] | [-.061, .031] | [-.206, .147] |
|  | (.290) | (.194) | (.211) | (.982) | (.119) | (.379) | (.814) | (.521) | (.744) |
| Rural Code=3 | -.009 | -.031 | -.014 | .009 | .036 | -.026 | -.002 | -.042 | -.080 |
|  | .023 | .033 | .014 | .031 | .031 | .028 | .032 | .032 | .123 |
|  | [-.054, .037] | [-.094, .033] | [-.042, .014] | [-.051, .069] | [-.025, .097] | [-.080, .028] | [-.065, .062] | [-.105, .020] | [-.321, .160] |
|  | (.701) | (.347) | (.327) | (.776) | (.244) | (.341) | (.961) | (.180) | (.512) |
| Rural Code=4 | -.081 | .019 | .004 | -.047 | -.035 | <.001 | -.072 | -.119 | -.332 |
|  | .032 | .045 | .020 | .043 | .043 | .038 | .045 | .044 | .171 |
|  | [-.145, -.018] | [-.069, .108] | [-.035, .042] | [-.130, .036] | [-.120, .050] | [-.075, .076] | [-.161, .016] | [-.206, -.033] | [-.667, .003] |
|  | (.012) | (.668) | (.857) | (.268) | (.416) | (.992) | (.108) | (.007) | (.052) |
| Rural Code=5 | -.037 | -<.001 | -.059 | .124 | .079 | .071 | -.067 | -.130 | -.019 |
|  | .050 | .069 | .030 | .065 | .067 | .059 | .069 | .068 | .262 |
|  | [-.135, .060] | [-.136, .136] | [-.119, .000] | [-.004, .252] | [-.051, .210] | [-.044, .186] | [-.202, .068] | [-.262, .003] | [-.532, .494] |
|  | (.450) | (.995) | (.050) | (.057) | (.234) | (.227) | (.331) | (.055) | (.943) |
| Rural Code=6 | -.052 | .007 | -.032 | -.009 | .001 | -.043 | -.020 | -.008 | -.156 |
|  | .032 | .045 | .020 | .042 | .043 | .038 | .045 | .044 | .170 |
|  | [-.115, .011] | [-.081, .095] | [-.071, .006] | [-.091, .074] | [-.084, .086] | [-.117, .032] | [-.107, .068] | [-.094, .078] | [-.489, .177] |
|  | (.103) | (.877) | (.102) | (.841) | (.980) | (.265) | (.663) | (.857) | (.359) |
| Rural Code=7 | -.101 | -.003 | -.001 | .010 | -.082 | -.028 | -.023 | -.084 | -.316 |
|  | .044 | .062 | .027 | .058 | .059 | .053 | .062 | .060 | .234 |
|  | [-.188, -.014] | [-.124, .119] | [-.054, .053] | [-.104, .124] | [-.199, .034] | [-.132, .075] | [-.144, .097] | [-.202, .034] | [-.774, .143] |
|  | (.023) | (.963) | (.985) | (.868) | (.166) | (.588) | (.704) | (.163) | (.177) |
| Rural Code=8 | -.120 | -.026 | -.057 | -.149 | -.177 | -.108 | -.159 | -.174 | -.970 |
|  | .083 | .116 | .051 | .109 | .112 | .099 | .116 | .113 | .439 |
|  | [-.283, .043] | [-.254, .202] | [-.156, .043] | [-.363, .064] | [-.396, .042] | [-.302, .086] | [-.386, .068] | [-.396, .048] | [-1.831, -.110] |
|  | (.150) | (.822) | (.265) | (.171) | (.114) | (.274) | (.169) | (.124) | (.027) |
| Rural Code=9 | -.129 | -.098 | -.003 | -.029 | -.026 | -.010 | .064 | -.013 | -.245 |
|  | .071 | .099 | .043 | .093 | .095 | .084 | .098 | .096 | .372 |
|  | [-.268, .009] | [-.291, .096] | [-.088, .081] | [-.210, .153] | [-.212, .159] | [-.174, .154] | [-.128, .256] | [-.201, .175] | [-.974, .485] |
|  | (.067) | (.323) | (.936) | (.756) | (.781) | (.906) | (.515) | (.892) | (.511) |
| Alaska | .252 | .160 | -.090 | .301 | -.009 | .168 | -.032 | .205 | .955 |
|  | .142 | .198 | .087 | .185 | .190 | .168 | .197 | .192 | .747 |
|  | [-.025, .530] | [-.228, .547] | [-.260, .079] | [-.063, .664] | [-.381, .364] | [-.161, .497] | [-.418, .354] | [-.172, .582] | [-.509, 2.419] |
|  | (.075) | (.419) | (.297) | (.105) | (.963) | (.317) | (.871) | (.287) | (.201) |
| Arizona | .050 | -.184 | -.046 | -.018 | .017 | .022 | -.122 | .108 | -.174 |
|  | .067 | .094 | .041 | .089 | .090 | .080 | .094 | .092 | .356 |
|  | [-.082, .182] | [-.368, .001] | [-.127, .034] | [-.192, .155] | [-.160, .194] | [-.135, .179] | [-.306, .061] | [-.072, .288] | [-.871, .523] |
|  | (.460) | (.052) | (.260) | (.836) | (.849) | (.784) | (.192) | (.240) | (.625) |
| Arkansas | .114 | -.066 | -.042 | -.012 | .017 | .002 | -.049 | .086 | .051 |
|  | .076 | .106 | .046 | .099 | .102 | .090 | .105 | .103 | .399 |
|  | [-.034, .263] | [-.273, .142] | [-.133, .049] | [-.207, .183] | [-.182, .217] | [-.174, .178] | [-.255, .157] | [-.116, .288] | [-.732, .833] |
|  | (.131) | (.533) | (.367) | (.902) | (.863) | (.983) | (.639) | (.402) | (.899) |
| California | .081 | -.024 | -.046 | .066 | .063 | .118 | -.046 | .186 | .397 |
|  | .059 | .083 | .036 | .078 | .080 | .070 | .082 | .081 | .313 |
|  | [-.035, .198] | [-.187, .139] | [-.118, .025] | [-.087, .219] | [-.093, .219] | [-.020, .256] | [-.208, .115] | [.027, .344] | [-.217, 1.010] |
|  | (.170) | (.773) | (.200) | (.396) | (.430) | (.093) | (.575) | (.022) | (.205) |
| Colorado | .188 | .004 | -.064 | .182 | .155 | .169 | .056 | .158 | .847 |
|  | .076 | .106 | .047 | .100 | .102 | .090 | .106 | .104 | .402 |
|  | [.039, .337] | [-.204, .213] | [-.155, .027] | [-.014, .378] | [-.045, .355] | [-.008, .346] | [-.151, .263] | [-.045, .362] | [.060, 1.634] |
|  | (.013) | (.968) | (.171) | (.069) | (.128) | (.061) | (.595) | (.127) | (.035) |
| Connecticut | -.003 | -.021 | -.094 | .039 | .193 | .144 | -.037 | .259 | .483 |
|  | .083 | .116 | .051 | .109 | .111 | .098 | .115 | .113 | .436 |
|  | [-.165, .159] | [-.248, .206] | [-.193, .005] | [-.174, .253] | [-.025, .410] | [-.048, .336] | [-.262, .188] | [.038, .480] | [-.373, 1.338] |
|  | (.973) | (.857) | (.063) | (.716) | (.082) | (.142) | (.749) | (.021) | (.269) |
| Delaware | .176 | .213 | -.088 | -.029 | .346 | .137 | .074 | .270 | 1.094 |
|  | .107 | .150 | .065 | .141 | .143 | .127 | .149 | .146 | .565 |
|  | [-.033, .385] | [-.080, .506] | [-.217, .040] | [-.305, .247] | [.065, .627] | [-.111, .385] | [-.217, .365] | [-.016, .556] | [-.012, 2.200] |
|  | (.099) | (.155) | (.177) | (.835) | (.016) | (.279) | (.617) | (.064) | (.053) |
| District of Columbia | .053 | -.185 | -.107 | .331 | .183 | .195 | -.134 | .331 | .666 |
|  | .133 | .186 | .082 | .175 | .179 | .158 | .186 | .181 | .704 |
|  | [-.209, .314] | [-.550, .181] | [-.267, .053] | [-.012, .674] | [-.168, .534] | [-.116, .505] | [-.498, .229] | [-.024, .687] | [-.714, 2.046] |
|  | (.692) | (.322) | (.189) | (.058) | (.307) | (.219) | (.469) | (.068) | (.344) |
| Florida | .090 | .030 | -.051 | .008 | .091 | .085 | -.065 | .136 | .321 |
|  | .060 | .084 | .037 | .079 | .081 | .071 | .083 | .082 | .317 |
|  | [-.028, .207] | [-.134, .195] | [-.123, .020] | [-.146, .162] | [-.067, .248] | [-.054, .224] | [-.228, .099] | [-.024, .296] | [-.300, .941] |
|  | (.134) | (.718) | (.161) | (.918) | (.260) | (.232) | (.438) | (.095) | (.311) |
| Georgia | .054 | -.025 | -.053 | .151 | .086 | .120 | -.096 | .106 | .344 |
|  | .065 | .091 | .040 | .085 | .087 | .077 | .090 | .088 | .343 |
|  | [-.073, .181] | [-.203, .154] | [-.131, .025] | [-.016, .318] | [-.085, .257] | [-.031, .271] | [-.273, .082] | [-.067, .280] | [-.328, 1.016] |
|  | (.406) | (.787) | (.184) | (.076) | (.326) | (.120) | (.290) | (.229) | (.316) |
| Hawaii | .124 | .027 | -.003 | -.309 | -.081 | .206 | -.018 | .084 | .030 |
|  | .115 | .161 | .070 | .152 | .154 | .136 | .160 | .157 | .607 |
|  | [-.101, .349] | [-.289, .343] | [-.141, .136] | [-.606, -.012] | [-.384, .221] | [-.061, .474] | [-.331, .295] | [-.223, .392] | [-1.160, 1.220] |
|  | (.280) | (.866) | (.972) | (.042) | (.599) | (.130) | (.911) | (.591) | (.961) |
| Idaho | -.112 | -.088 | -.085 | .089 | -.092 | .005 | -.295 | -.037 | -.618 |
|  | .096 | .135 | .059 | .127 | .129 | .114 | .134 | .132 | .509 |
|  | [-.301, .076] | [-.353, .176] | [-.201, .030] | [-.160, .338] | [-.345, .161] | [-.219, .229] | [-.558, -.033] | [-.295, .221] | [-1.615, .379] |
|  | (.243) | (.512) | (.148) | (.483) | (.475) | (.967) | (.027) | (.779) | (.224) |
| Illinois | .023 | -.138 | -.058 | .108 | .026 | .058 | -.089 | .197 | .129 |
|  | .064 | .089 | .039 | .084 | .086 | .076 | .089 | .087 | .338 |
|  | [-.102, .148] | [-.313, .038] | [-.134, .019] | [-.057, .273] | [-.142, .195] | [-.091, .206] | [-.263, .086] | [.026, .368] | [-.533, .790] |
|  | (.718) | (.124) | (.140) | (.199) | (.759) | (.448) | (.318) | (.024) | (.703) |
| Indiana | .037 | -.127 | -.053 | .097 | .117 | .176 | -.138 | .184 | .293 |
|  | .071 | .099 | .043 | .093 | .095 | .084 | .098 | .096 | .373 |
|  | [-.101, .176] | [-.321, .067] | [-.137, .032] | [-.085, .279] | [-.069, .303] | [.011, .340] | [-.331, .055] | [-.005, .372] | [-.438, 1.025] |
|  | (.599) | (.200) | (.225) | (.298) | (.219) | (.036) | (.161) | (.056) | (.432) |
| Iowa | .176 | .009 | -.010 | .029 | -.062 | .105 | -.053 | -.054 | .140 |
|  | .084 | .118 | .051 | .110 | .113 | .100 | .117 | .114 | .444 |
|  | [.011, .341] | [-.221, .239] | [-.110, .091] | [-.187, .245] | [-.283, .159] | [-.091, .300] | [-.283, .176] | [-.278, .170] | [-.730, 1.009] |
|  | (.037) | (.938) | (.853) | (.795) | (.583) | (.294) | (.649) | (.638) | (.753) |
| Kansas | .193 | -.164 | -.099 | -.012 | .082 | .180 | -.064 | .099 | .216 |
|  | .102 | .142 | .062 | .134 | .137 | .121 | .142 | .139 | .537 |
|  | [-.006, .393] | [-.443, .115] | [-.221, .023] | [-.274, .250] | [-.185, .350] | [-.057, .416] | [-.341, .214] | [-.172, .371] | [-.836, 1.269] |
|  | (.057) | (.249) | (.113) | (.931) | (.547) | (.136) | (.653) | (.473) | (.687) |
| Kentucky | .139 | -.113 | -.047 | -.161 | .130 | .067 | -.052 | .003 | -.033 |
|  | .077 | .108 | .047 | .101 | .103 | .091 | .107 | .105 | .406 |
|  | [-.012, .290] | [-.324, .098] | [-.140, .045] | [-.359, .037] | [-.073, .332] | [-.112, .246] | [-.262, .158] | [-.203, .208] | [-.829, .763] |
|  | (.071) | (.294) | (.316) | (.111) | (.209) | (.464) | (.625) | (.979) | (.935) |
| Louisiana | .106 | -.099 | -.078 | -.081 | .146 | .165 | -.039 | .106 | .227 |
|  | .077 | .108 | .047 | .102 | .104 | .092 | .107 | .105 | .408 |
|  | [-.045, .257] | [-.310, .113] | [-.171, .014] | [-.280, .118] | [-.058, .349] | [-.015, .344] | [-.250, .171] | [-.100, .313] | [-.572, 1.026] |
|  | (.169) | (.362) | (.097) | (.426) | (.160) | (.072) | (.713) | (.312) | (.578) |
| Maine | .122 | -.221 | -.083 | .170 | .234 | .226 | .272 | .231 | .951 |
|  | .107 | .150 | .066 | .141 | .144 | .127 | .149 | .146 | .565 |
|  | [-.088, .331] | [-.514, .073] | [-.211, .045] | [-.106, .446] | [-.048, .515] | [-.023, .474] | [-.019, .564] | [-.055, .517] | [-.157, 2.058] |
|  | (.254) | (.141) | (.205) | (.227) | (.104) | (.075) | (.067) | (.114) | (.093) |
| Maryland | .120 | -.030 | -.077 | .008 | .091 | .173 | -.030 | -.015 | .241 |
|  | .076 | .107 | .047 | .100 | .103 | .091 | .106 | .104 | .404 |
|  | [-.030, .270] | [-.239, .180] | [-.169, .015] | [-.189, .205] | [-.110, .293] | [-.004, .351] | [-.238, .179] | [-.219, .189] | [-.550, 1.032] |
|  | (.117) | (.782) | (.100) | (.937) | (.373) | (.056) | (.779) | (.888) | (.550) |
| Massachusetts | .070 | -.095 | -.083 | .108 | .162 | .184 | -.185 | .177 | .335 |
|  | .072 | .100 | .044 | .094 | .096 | .085 | .100 | .098 | .379 |
|  | [-.071, .211] | [-.292, .102] | [-.169, .003] | [-.077, .293] | [-.027, .351] | [.017, .350] | [-.381, .010] | [-.015, .368] | [-.408, 1.078] |
|  | (.329) | (.343) | (.058) | (.251) | (.093) | (.031) | (.064) | (.071) | (.377) |
| Michigan | .119 | -.052 | -.026 | -.052 | .109 | .096 | -.116 | .087 | .165 |
|  | .064 | .090 | .039 | .084 | .086 | .076 | .089 | .087 | .338 |
|  | [-.006, .245] | [-.227, .124] | [-.103, .051] | [-.217, .112] | [-.060, .277] | [-.053, .245] | [-.291, .059] | [-.084, .258] | [-.498, .827] |
|  | (.062) | (.565) | (.505) | (.534) | (.206) | (.205) | (.193) | (.317) | (.626) |
| Minnesota | .119 | -.120 | -.073 | .076 | .104 | .107 | -.035 | .136 | .314 |
|  | .076 | .106 | .046 | .099 | .101 | .090 | .105 | .103 | .399 |
|  | [-.029, .267] | [-.327, .088] | [-.163, .018] | [-.119, .270] | [-.095, .303] | [-.069, .282] | [-.241, .171] | [-.066, .338] | [-.468, 1.096] |
|  | (.114) | (.258) | (.117) | (.446) | (.305) | (.235) | (.737) | (.186) | (.432) |
| Mississippi | .086 | -.076 | -.024 | -.105 | .138 | .075 | .043 | .241 | .378 |
|  | .086 | .120 | .052 | .112 | .115 | .102 | .119 | .116 | .451 |
|  | [-.082, .254] | [-.310, .158] | [-.127, .079] | [-.325, .115] | [-.087, .363] | [-.124, .273] | [-.190, .277] | [.013, .469] | [-.507, 1.263] |
|  | (.315) | (.525) | (.646) | (.348) | (.229) | (.463) | (.715) | (.039) | (.403) |
| Missouri | .015 | -.129 | -.040 | -.001 | .033 | .151 | -.088 | .042 | -.017 |
|  | .069 | .097 | .042 | .091 | .093 | .082 | .096 | .094 | .366 |
|  | [-.120, .151] | [-.319, .061] | [-.123, .043] | [-.179, .178] | [-.149, .215] | [-.010, .312] | [-.277, .100] | [-.143, .227] | [-.734, .700] |
|  | (.824) | (.182) | (.343) | (.995) | (.724) | (.065) | (.358) | (.655) | (.963) |
| Montana | .149 | -.079 | -.064 | .084 | .070 | .130 | -.184 | .153 | .262 |
|  | .107 | .149 | .065 | .141 | .143 | .127 | .148 | .146 | .564 |
|  | [-.060, .358] | [-.372, .214] | [-.192, .065] | [-.191, .360] | [-.210, .351] | [-.119, .378] | [-.475, .107] | [-.132, .439] | [-.843, 1.367] |
|  | (.162) | (.599) | (.331) | (.548) | (.623) | (.306) | (.216) | (.292) | (.642) |
| Nebraska | .098 | -.030 | -.089 | -.007 | .113 | .124 | -.031 | .025 | .202 |
|  | .110 | .154 | .067 | .144 | .148 | .131 | .153 | .149 | .580 |
|  | [-.118, .314] | [-.332, .271] | [-.221, .042] | [-.289, .276] | [-.177, .402] | [-.132, .380] | [-.331, .269] | [-.268, .318] | [-.936, 1.339] |
|  | (.373) | (.844) | (.183) | (.962) | (.446) | (.343) | (.841) | (.865) | (.728) |
| Nevada | .066 | -.163 | -.053 | -.044 | .215 | .140 | -.041 | .246 | .368 |
|  | .075 | .106 | .046 | .099 | .101 | .089 | .105 | .103 | .398 |
|  | [-.082, .214] | [-.369, .044] | [-.143, .038] | [-.239, .151] | [.016, .413] | [-.035, .315] | [-.246, .164] | [.044, .447] | [-.413, 1.148] |
|  | (.381) | (.124) | (.254) | (.658) | (.034) | (.117) | (.696) | (.017) | (.356) |
| New Hampshire | .147 | -.192 | -.037 | -.082 | .025 | .070 | -.047 | -.005 | -.122 |
|  | .090 | .127 | .055 | .119 | .121 | .107 | .126 | .123 | .478 |
|  | [-.030, .324] | [-.440, .056] | [-.145, .072] | [-.315, .152] | [-.213, .262] | [-.140, .281] | [-.293, .199] | [-.247, .237] | [-1.058, .814] |
|  | (.104) | (.130) | (.506) | (.492) | (.839) | (.512) | (.709) | (.968) | (.798) |
| New Jersey | .121 | .038 | -.043 | .059 | .128 | .146 | -.048 | .060 | .461 |
|  | .070 | .098 | .043 | .092 | .094 | .083 | .097 | .095 | .368 |
|  | [-.016, .258] | [-.153, .229] | [-.127, .041] | [-.120, .239] | [-.056, .311] | [-.016, .308] | [-.238, .142] | [-.126, .246] | [-.261, 1.183] |
|  | (.083) | (.696) | (.315) | (.516) | (.173) | (.078) | (.622) | (.529) | (.211) |
| New Mexico | .113 | -.122 | -.056 | .048 | .124 | -.047 | .061 | .141 | .259 |
|  | .083 | .117 | .051 | .110 | .112 | .099 | .116 | .114 | .440 |
|  | [-.050, .276] | [-.351, .107] | [-.156, .044] | [-.167, .263] | [-.095, .343] | [-.240, .147] | [-.166, .289] | [-.082, .363] | [-.603, 1.121] |
|  | (.175) | (.295) | (.268) | (.660) | (.267) | (.636) | (.596) | (.215) | (.556) |
| New York | .025 | .029 | -.030 | .051 | .092 | .147 | -.040 | .105 | .381 |
|  | .061 | .086 | .037 | .080 | .082 | .073 | .085 | .083 | .323 |
|  | [-.095, .144] | [-.138, .197] | [-.103, .043] | [-.107, .208] | [-.069, .253] | [.005, .289] | [-.207, .127] | [-.058, .268] | [-.252, 1.013] |
|  | (.688) | (.731) | (.422) | (.527) | (.263) | (.042) | (.639) | (.206) | (.238) |
| North Carolina | .096 | .016 | -.035 | .056 | .106 | .135 | .008 | .058 | .440 |
|  | .066 | .092 | .040 | .087 | .089 | .078 | .092 | .090 | .348 |
|  | [-.033, .225] | [-.165, .197] | [-.114, .044] | [-.114, .226] | [-.067, .280] | [-.018, .289] | [-.172, .188] | [-.118, .234] | [-.242, 1.123] |
|  | (.145) | (.860) | (.386) | (.516) | (.229) | (.084) | (.930) | (.518) | (.206) |
| North Dakota | .094 | .325 | -.084 | -.050 | .010 | .211 | -.015 | .152 | .642 |
|  | .134 | .188 | .082 | .176 | .180 | .159 | .186 | .183 | .708 |
|  | [-.168, .357] | [-.043, .692] | [-.245, .077] | [-.396, .295] | [-.342, .363] | [-.100, .523] | [-.380, .350] | [-.206, .510] | [-.745, 2.030] |
|  | (.481) | (.084) | (.304) | (.775) | (.954) | (.183) | (.937) | (.406) | (.364) |
| Ohio | .106 | -.038 | -.046 | .050 | .095 | .153 | -.010 | .150 | .461 |
|  | .064 | .090 | .039 | .084 | .086 | .076 | .089 | .087 | .339 |
|  | [-.020, .232] | [-.214, .138] | [-.122, .031] | [-.115, .215] | [-.074, .263] | [.004, .303] | [-.185, .165] | [-.021, .321] | [-.203, 1.125] |
|  | (.098) | (.669) | (.246) | (.552) | (.272) | (.044) | (.909) | (.086) | (.173) |
| Oklahoma | .045 | .082 | .020 | .082 | .146 | .108 | -.078 | .115 | .519 |
|  | .086 | .120 | .053 | .113 | .115 | .102 | .120 | .117 | .454 |
|  | [-.124, .213] | [-.154, .318] | [-.083, .123] | [-.140, .303] | [-.080, .372] | [-.092, .308] | [-.312, .157] | [-.114, .345] | [-.371, 1.409] |
|  | (.602) | (.496) | (.707) | (.470) | (.206) | (.290) | (.515) | (.325) | (.253) |
| Oregon | .017 | -.129 | -.072 | .064 | .067 | .124 | -.041 | .150 | .181 |
|  | .071 | .099 | .043 | .094 | .095 | .084 | .099 | .097 | .375 |
|  | [-.122, .156] | [-.324, .066] | [-.157, .013] | [-.119, .248] | [-.120, .254] | [-.041, .289] | [-.234, .153] | [-.039, .340] | [-.554, .916] |
|  | (.810) | (.193) | (.099) | (.492) | (.482) | (.142) | (.679) | (.120) | (.629) |
| Pennsylvania | .113 | -.113 | -.056 | .035 | .101 | .155 | -.082 | .109 | .261 |
|  | .061 | .085 | .037 | .080 | .082 | .072 | .085 | .083 | .321 |
|  | [-.006, .232] | [-.280, .053] | [-.129, .017] | [-.121, .191] | [-.059, .261] | [.014, .297] | [-.247, .084] | [-.053, .271] | [-.367, .890] |
|  | (.063) | (.182) | (.134) | (.661) | (.217) | (.031) | (.334) | (.186) | (.415) |
| Rhode Island | .098 | .300 | .034 | .153 | .195 | .104 | -.092 | .134 | .926 |
|  | .133 | .186 | .081 | .175 | .179 | .158 | .185 | .181 | .703 |
|  | [-.163, .359] | [-.065, .665] | [-.126, .194] | [-.189, .495] | [-.155, .546] | [-.205, .414] | [-.455, .271] | [-.221, .489] | [-.451, 2.304] |
|  | (.460) | (.107) | (.677) | (.382) | (.275) | (.509) | (.620) | (.460) | (.187) |
| South Carolina | .099 | .008 | -.073 | .041 | .187 | .195 | .035 | .142 | .631 |
|  | .074 | .104 | .046 | .098 | .100 | .088 | .104 | .102 | .393 |
|  | [-.046, .245] | [-.197, .212] | [-.162, .016] | [-.151, .233] | [-.009, .383] | [.022, .368] | [-.168, .237] | [-.057, .341] | [-.140, 1.401] |
|  | (.182) | (.943) | (.109) | (.674) | (.061) | (.027) | (.739) | (.163) | (.109) |
| South Dakota | -.022 | -.315 | -.089 | -.105 | -.139 | .093 | -.199 | -.183 | -.958 |
|  | .123 | .172 | .075 | .162 | .165 | .146 | .171 | .168 | .651 |
|  | [-.263, .220] | [-.653, .023] | [-.237, .059] | [-.422, .213] | [-.463, .186] | [-.194, .379] | [-.535, .137] | [-.512, .147] | [-2.233, .318] |
|  | (.861) | (.068) | (.238) | (.518) | (.402) | (.526) | (.246) | (.277) | (.141) |
| Tennessee | .156 | .090 | -.047 | .045 | .099 | .226 | .075 | .136 | .779 |
|  | .073 | .102 | .045 | .096 | .098 | .086 | .101 | .099 | .384 |
|  | [.014, .299] | [-.110, .290] | [-.134, .040] | [-.143, .232] | [-.093, .290] | [.057, .395] | [-.124, .273] | [-.059, .330] | [.026, 1.532] |
|  | (.032) | (.377) | (.292) | (.638) | (.313) | (.009) | (.460) | (.171) | (.043) |
| Texas | .053 | -.014 | -.035 | .038 | .015 | .047 | -.142 | .008 | -.029 |
|  | .061 | .085 | .037 | .080 | .082 | .072 | .085 | .083 | .321 |
|  | [-.066, .173] | [-.181, .153] | [-.108, .038] | [-.118, .194] | [-.145, .175] | [-.094, .189] | [-.307, .024] | [-.154, .170] | [-.658, .600] |
|  | (.379) | (.870) | (.344) | (.635) | (.856) | (.514) | (.094) | (.920) | (.927) |
| Utah | .065 | -.143 | -.076 | .151 | .036 | .115 | -.037 | .114 | .222 |
|  | .084 | .118 | .052 | .111 | .113 | .100 | .117 | .115 | .445 |
|  | [-.100, .230] | [-.375, .088] | [-.177, .025] | [-.067, .369] | [-.186, .257] | [-.081, .311] | [-.266, .193] | [-.111, .340] | [-.650, 1.095] |
|  | (.441) | (.225) | (.142) | (.174) | (.752) | (.252) | (.754) | (.321) | (.617) |
| Vermont | .237 | -.136 | -.081 | .218 | .180 | .009 | .053 | -.105 | .376 |
|  | .122 | .170 | .075 | .160 | .164 | .145 | .170 | .166 | .643 |
|  | [-.002, .476] | [-.470, .198] | [-.227, .065] | [-.095, .531] | [-.141, .500] | [-.274, .293] | [-.279, .385] | [-.430, .220] | [-.884, 1.637] |
|  | (.052) | (.425) | (.278) | (.173) | (.273) | (.948) | (.754) | (.526) | (.558) |
| Virginia | .072 | .028 | -.049 | .043 | .049 | .149 | -.030 | .152 | .414 |
|  | .065 | .091 | .040 | .086 | .087 | .077 | .091 | .089 | .344 |
|  | [-.055, .200] | [-.150, .207] | [-.127, .029] | [-.125, .211] | [-.122, .221] | [-.003, .300] | [-.207, .148] | [-.022, .326] | [-.260, 1.089] |
|  | (.266) | (.756) | (.218) | (.619) | (.574) | (.055) | (.743) | (.087) | (.229) |
| Washington | .114 | -.085 | -.016 | .064 | .149 | .184 | -.027 | .113 | .494 |
|  | .068 | .096 | .042 | .090 | .092 | .081 | .095 | .093 | .361 |
|  | [-.020, .248] | [-.273, .102] | [-.098, .066] | [-.112, .240] | [-.031, .328] | [.026, .343] | [-.213, .160] | [-.070, .296] | [-.213, 1.202] |
|  | (.096) | (.373) | (.710) | (.476) | (.105) | (.023) | (.778) | (.225) | (.171) |
| West Virginia | -.010 | -.038 | -.036 | .058 | -.034 | .039 | -.150 | -.013 | -.182 |
|  | .087 | .122 | .053 | .114 | .117 | .103 | .121 | .118 | .459 |
|  | [-.180, .161] | [-.276, .201] | [-.140, .069] | [-.165, .282] | [-.263, .195] | [-.163, .241] | [-.387, .087] | [-.245, .219] | [-1.082, .718] |
|  | (.913) | (.758) | (.502) | (.609) | (.769) | (.705) | (.216) | (.915) | (.692) |
| Wisconsin | .072 | -.078 | -.031 | .096 | .082 | .135 | -.104 | .033 | .203 |
|  | .071 | .099 | .043 | .093 | .095 | .084 | .098 | .096 | .373 |
|  | [-.067, .210] | [-.272, .116] | [-.116, .053] | [-.086, .278] | [-.104, .268] | [-.030, .299] | [-.297, .089] | [-.156, .221] | [-.528, .935] |
|  | (.308) | (.429) | (.468) | (.303) | (.388) | (.108) | (.290) | (.733) | (.586) |
| Wyoming | .272 | .251 | .238 | .035 | .145 | .068 | .156 | .488 | 1.659 |
|  | .205 | .289 | .126 | .273 | .276 | .244 | .285 | .283 | 1.088 |
|  | [-.130, .675] | [-.316, .818] | [-.009, .486] | [-.501, .571] | [-.395, .685] | [-.409, .545] | [-.404, .715] | [-.066, 1.041] | [-.473, 3.791] |
|  | (.184) | (.385) | (.059) | (.898) | (.599) | (.780) | (.586) | (.084) | (.127) |
| N | 2982 | 2982 | 2982 | 2982 | 2982 | 2982 | 2982 | 2982 | 2982 |
| Pseudo-R^2^ |  |  |  |  |  |  |  |  |  |
| Model χ^2^ | 208.7 | 196.6 | 114.7 | 32.4 | 214.2 | 226.9 | 31.8 | 213.9 | 383.5 |
| d.f. | 87 | 87 | 87 | 87 | 87 | 87 | 87 | 87 | 87 |
| p-value | <.001 | <.001 | <.001 | <.001 | <.001 | <.001 | <.001 | <.001 | <.001 |

S8 Table. Health Behaviors, Multilevel Models, Controlling for Deaths.

|  | (1) | (2) | (3) | (4) | (5) | (6) | (7) | (8) | (9) |
| --- | --- | --- | --- | --- | --- | --- | --- | --- | --- |
|  | washhands | boughtsanitizer | visitdoctor | changedtravel | avoidcontact | avoidgatherings | soughtinfo | selfquarantine | changehealth |
| main |  |  |  |  |  |  |  |  |  |
| Democrat | .084 | .083 | -.006 | .065 | .170 | .108 | .193 | .119 | .814 |
|  | .017 | .023 | .010 | .022 | .022 | .020 | .023 | .023 | .088 |
|  | [.051, .117] | [.037, .128] | [-.026, .014] | [.022, .107] | [.126, .213] | [.069, .147] | [.147, .238] | [.074, .163] | [.641, .986] |
|  | (<.001) | (<.001) | (.537) | (.003) | (<.001) | (<.001) | (<.001) | (<.001) | (<.001) |
| Other | -.010 | -.003 | -.020 | -.003 | .049 | .024 | .069 | .029 | .135 |
|  | .017 | .024 | .010 | .022 | .023 | .020 | .023 | .023 | .089 |
|  | [-.043, .023] | [-.049, .044] | [-.040, .000] | [-.047, .040] | [.004, .093] | [-.015, .064] | [.023, .115] | [-.016, .074] | [-.040, .310] |
|  | (.552) | (.910) | (.053) | (.884) | (.032) | (.223) | (.003) | (.202) | (.130) |
| Local COVID-19 Deaths | <.001 | <.001 | <.001 | -<.001 | <.001 | <.001 | -<.001 | <.001 | <.001 |
|  | <.001 | <.001 | <.001 | <.001 | <.001 | <.001 | <.001 | <.001 | <.001 |
|  | [-<.001, .000] | [.000, .000] | [-<.001, .000] | [-<.001, .000] | [-<.001, .000] | [-<.001, .000] | [-<.001, .000] | [-<.001, .000] | [-<.001, .000] |
|  | (.078) | (.012) | (.882) | (.553) | (.693) | (.325) | (.147) | (.723) | (.366) |
| 35- | .007 | .051 | .001 | -.049 | .001 | -.011 | -.008 | -.043 | -.052 |
|  | .019 | .026 | .011 | .025 | .025 | .022 | .026 | .025 | .099 |
|  | [-.029, .044] | [.000, .103] | [-.021, .024] | [-.097, -.001] | [-.048, .050] | [-.055, .032] | [-.059, .043] | [-.093, .007] | [-.246, .141] |
|  | (.699) | (.049) | (.917) | (.046) | (.973) | (.613) | (.746) | (.089) | (.597) |
| 50- | .025 | .052 | -.008 | -.067 | .017 | .053 | -.035 | -.072 | -.035 |
|  | .018 | .025 | .011 | .023 | .024 | .021 | .025 | .024 | .093 |
|  | [-.010, .060] | [.004, .101] | [-.029, .013] | [-.112, -.021] | [-.029, .064] | [.012, .094] | [-.083, .013] | [-.119, -.025] | [-.218, .148] |
|  | (.158) | (.034) | (.472) | (.004) | (.465) | (.012) | (.158) | (.003) | (.707) |
| 70- | .061 | .069 | -.012 | -.024 | .103 | .141 | -.071 | .064 | .329 |
|  | .022 | .031 | .014 | .029 | .030 | .027 | .031 | .030 | .118 |
|  | [.017, .105] | [.008, .130] | [-.038, .015] | [-.082, .033] | [.044, .162] | [.089, .193] | [-.132, -.010] | [.004, .123] | [.097, .560] |
|  | (.007) | (.028) | (.395) | (.406) | (.001) | (<.001) | (.023) | (.036) | (.005) |
| gender | .041 | .016 | -.002 | -.012 | .007 | .042 | .036 | .060 | .186 |
|  | .013 | .018 | .008 | .017 | .017 | .015 | .018 | .017 | .068 |
|  | [.016, .066] | [-.019, .051] | [-.018, .013] | [-.045, .021] | [-.027, .040] | [.012, .072] | [.000, .071] | [.025, .094] | [.053, .319] |
|  | (.001) | (.374) | (.758) | (.467) | (.703) | (.005) | (.047) | (.001) | (.006) |
| white | -.010 | -.082 | -.031 | -.031 | .043 | .051 | .079 | .003 | .022 |
|  | .015 | .021 | .009 | .020 | .021 | .018 | .021 | .021 | .081 |
|  | [-.040, .020] | [-.124, -.039] | [-.050, -.013] | [-.070, .009] | [.003, .084] | [.015, .086] | [.037, .121] | [-.038, .044] | [-.137, .181] |
|  | (.516) | (<.001) | (.001) | (.126) | (.036) | (.006) | (<.001) | (.888) | (.788) |
| married | -.002 | .046 | .014 | .043 | .032 | -.004 | .021 | .012 | .162 |
|  | .014 | .020 | .009 | .019 | .019 | .017 | .020 | .019 | .075 |
|  | [-.030, .026] | [.007, .085] | [-.002, .031] | [.007, .080] | [-.005, .069] | [-.037, .029] | [-.018, .059] | [-.026, .049] | [.016, .308] |
|  | (.882) | (.019) | (.094) | (.020) | (.092) | (.802) | (.296) | (.547) | (.030) |
| High school graduate | -.013 | .008 | -.001 | .081 | -.120 | -.037 | <.001 | .002 | -.080 |
|  | .038 | .053 | .023 | .049 | .051 | .045 | .052 | .051 | .199 |
|  | [-.087, .061] | [-.095, .111] | [-.047, .044] | [-.016, .177] | [-.219, -.021] | [-.125, .051] | [-.103, .103] | [-.098, .103] | [-.470, .310] |
|  | (.726) | (.877) | (.955) | (.103) | (.018) | (.410) | (.997) | (.966) | (.687) |
| Some college | .026 | -.006 | -.017 | .167 | -.059 | .025 | .136 | .017 | .291 |
|  | .038 | .054 | .024 | .050 | .052 | .046 | .054 | .052 | .203 |
|  | [-.049, .102] | [-.112, .099] | [-.063, .029] | [.068, .266] | [-.161, .042] | [-.064, .115] | [.031, .241] | [-.085, .120] | [-.107, .688] |
|  | (.491) | (.907) | (.480) | (.001) | (.250) | (.579) | (.011) | (.742) | (.152) |
| 2-year | .021 | .010 | -.006 | .122 | -.066 | .019 | .126 | -.006 | .221 |
|  | .040 | .056 | .025 | .053 | .054 | .048 | .056 | .055 | .213 |
|  | [-.058, .100] | [-.100, .121] | [-.055, .042] | [.019, .226] | [-.173, .040] | [-.075, .113] | [.015, .236] | [-.114, .101] | [-.197, .639] |
|  | (.604) | (.855) | (.796) | (.021) | (.221) | (.685) | (.026) | (.910) | (.300) |
| 4-year | .069 | -.027 | .014 | .200 | -.036 | .057 | .156 | .029 | .465 |
|  | .039 | .055 | .024 | .051 | .053 | .047 | .055 | .053 | .207 |
|  | [-.008, .146] | [-.134, .081] | [-.033, .061] | [.100, .301] | [-.139, .067] | [-.034, .148] | [.049, .263] | [-.076, .133] | [.059, .870] |
|  | (.080) | (.624) | (.559) | (<.001) | (.494) | (.220) | (.004) | (.588) | (.025) |
| Post-grad | .055 | -.015 | .019 | .303 | .021 | .084 | .226 | .070 | .766 |
|  | .041 | .058 | .025 | .054 | .055 | .049 | .057 | .056 | .218 |
|  | [-.026, .136] | [-.128, .098] | [-.031, .068] | [.197, .409] | [-.088, .130] | [-.011, .180] | [.114, .339] | [-.040, .179] | [.340, 1.193] |
|  | (.184) | (.793) | (.459) | (<.001) | (.705) | (.084) | (<.001) | (.214) | (<.001) |
| income=2 | -.013 | -.036 | -.080 | -.010 | -.001 | -.022 | .022 | -.064 | -.202 |
|  | .036 | .051 | .022 | .048 | .049 | .043 | .051 | .049 | .192 |
|  | [-.084, .058] | [-.136, .063] | [-.123, -.036] | [-.104, .083] | [-.097, .095] | [-.107, .063] | [-.077, .121] | [-.161, .033] | [-.578, .174] |
|  | (.723) | (.476) | (<.001) | (.827) | (.985) | (.613) | (.667) | (.197) | (.292) |
| income=3 | -.033 | -.049 | -.061 | -.025 | -.036 | .010 | .050 | -.127 | -.270 |
|  | .035 | .049 | .022 | .046 | .047 | .042 | .049 | .048 | .186 |
|  | [-.102, .036] | [-.145, .048] | [-.104, -.019] | [-.115, .066] | [-.129, .056] | [-.072, .091] | [-.046, .146] | [-.220, -.033] | [-.634, .095] |
|  | (.352) | (.321) | (.004) | (.593) | (.443) | (.820) | (.307) | (.008) | (.147) |
| income=4 | .022 | .067 | -.067 | -.004 | .038 | .068 | .103 | -.126 | .100 |
|  | .036 | .050 | .022 | .047 | .048 | .043 | .050 | .049 | .190 |
|  | [-.049, .092] | [-.032, .165] | [-.111, -.024] | [-.097, .088] | [-.057, .133] | [-.016, .152] | [.004, .201] | [-.222, -.030] | [-.273, .473] |
|  | (.550) | (.186) | (.002) | (.928) | (.439) | (.111) | (.041) | (.010) | (.600) |
| income=5 | .035 | -.016 | -.065 | -.048 | -.013 | .053 | .121 | -.129 | -.061 |
|  | .037 | .051 | .023 | .048 | .049 | .044 | .051 | .050 | .194 |
|  | [-.038, .107] | [-.116, .085] | [-.109, -.021] | [-.142, .047] | [-.110, .084] | [-.033, .138] | [.021, .222] | [-.227, -.031] | [-.442, .320] |
|  | (.348) | (.761) | (.004) | (.322) | (.798) | (.230) | (.018) | (.010) | (.755) |
| income=6 | .043 | .007 | -.059 | -.016 | .048 | .083 | .071 | -.117 | .063 |
|  | .037 | .052 | .023 | .049 | .050 | .044 | .052 | .051 | .197 |
|  | [-.030, .116] | [-.095, .109] | [-.103, -.014] | [-.111, .080] | [-.050, .146] | [-.003, .170] | [-.031, .172] | [-.216, -.018] | [-.323, .448] |
|  | (.253) | (.889) | (.010) | (.744) | (.339) | (.059) | (.172) | (.021) | (.750) |
| income=7 | .028 | -.004 | -.052 | .109 | .001 | .080 | .113 | -.148 | .126 |
|  | .040 | .056 | .024 | .052 | .054 | .047 | .055 | .054 | .210 |
|  | [-.051, .106] | [-.113, .105] | [-.100, -.004] | [.006, .211] | [-.104, .106] | [-.013, .173] | [.004, .221] | [-.254, -.042] | [-.287, .538] |
|  | (.488) | (.938) | (.034) | (.037) | (.989) | (.091) | (.042) | (.006) | (.549) |
| income=8 | -.002 | -.075 | -.088 | .055 | -.029 | .049 | .009 | -.080 | -.160 |
|  | .039 | .054 | .024 | .051 | .052 | .046 | .054 | .053 | .205 |
|  | [-.078, .075] | [-.181, .032] | [-.134, -.041] | [-.045, .155] | [-.131, .074] | [-.041, .139] | [-.097, .115] | [-.183, .023] | [-.561, .242] |
|  | (.967) | (.169) | (<.001) | (.279) | (.585) | (.288) | (.873) | (.130) | (.436) |
| income=9 | .072 | .054 | -.082 | .053 | .016 | .048 | .111 | -.150 | .124 |
|  | .038 | .054 | .023 | .050 | .052 | .046 | .053 | .052 | .203 |
|  | [-.003, .147] | [-.051, .160] | [-.128, -.036] | [-.046, .152] | [-.085, .117] | [-.041, .138] | [.006, .216] | [-.252, -.047] | [-.273, .521] |
|  | (.061) | (.309) | (<.001) | (.291) | (.759) | (.289) | (.038) | (.004) | (.541) |
| income=10 | .030 | -.030 | -.085 | .087 | .054 | .074 | .030 | -.142 | .019 |
|  | .040 | .056 | .024 | .052 | .054 | .047 | .056 | .054 | .211 |
|  | [-.048, .108] | [-.139, .079] | [-.133, -.037] | [-.016, .189] | [-.052, .159] | [-.019, .167] | [-.079, .139] | [-.248, -.036] | [-.394, .431] |
|  | (.456) | (.590) | (.001) | (.096) | (.317) | (.120) | (.585) | (.009) | (.930) |
| income=11 | .017 | .046 | -.088 | .108 | .013 | .050 | .070 | -.112 | .105 |
|  | .041 | .057 | .025 | .054 | .055 | .049 | .057 | .056 | .216 |
|  | [-.064, .097] | [-.067, .158] | [-.137, -.039] | [.003, .214] | [-.095, .121] | [-.045, .146] | [-.042, .182] | [-.221, -.003] | [-.319, .529] |
|  | (.682) | (.427) | (<.001) | (.044) | (.819) | (.301) | (.218) | (.045) | (.627) |
| income=12 | .073 | .048 | -.074 | .107 | .062 | .068 | .122 | -.107 | .300 |
|  | .045 | .063 | .028 | .059 | .061 | .054 | .063 | .061 | .238 |
|  | [-.016, .161] | [-.076, .171] | [-.128, -.020] | [-.009, .223] | [-.056, .181] | [-.037, .173] | [-.001, .245] | [-.227, .013] | [-.166, .767] |
|  | (.107) | (.448) | (.007) | (.070) | (.303) | (.202) | (.051) | (.080) | (.207) |
| income=13 | .051 | .006 | -.130 | .214 | .066 | .123 | .236 | -.065 | .502 |
|  | .062 | .087 | .038 | .082 | .084 | .074 | .087 | .085 | .329 |
|  | [-.071, .173] | [-.165, .177] | [-.204, -.055] | [.054, .374] | [-.098, .231] | [-.022, .269] | [.066, .406] | [-.232, .101] | [-.143, 1.148] |
|  | (.415) | (.944) | (.001) | (.009) | (.428) | (.095) | (.007) | (.441) | (.127) |
| income=14 | -.013 | .078 | -.030 | .317 | .100 | .099 | .076 | -.034 | .593 |
|  | .066 | .092 | .040 | .087 | .089 | .078 | .092 | .090 | .349 |
|  | [-.142, .117] | [-.103, .258] | [-.109, .049] | [.147, .486] | [-.074, .274] | [-.054, .253] | [-.104, .256] | [-.210, .142] | [-.091, 1.276] |
|  | (.845) | (.400) | (.456) | (<.001) | (.259) | (.205) | (.409) | (.708) | (.089) |
| income=15 | .118 | .196 | .176 | .267 | .316 | .223 | .435 | .188 | 1.927 |
|  | .200 | .279 | .122 | .262 | .269 | .238 | .278 | .272 | 1.056 |
|  | [-.274, .511] | [-.351, .743] | [-.064, .416] | [-.246, .780] | [-.211, .843] | [-.243, .688] | [-.110, .981] | [-.345, .721] | [-.143, 3.997] |
|  | (.554) | (.483) | (.150) | (.308) | (.240) | (.349) | (.118) | (.489) | (.068) |
| income=16 | .133 | .382 | -.058 | .187 | .156 | .081 | .195 | -.114 | .960 |
|  | .094 | .131 | .058 | .123 | .126 | .112 | .131 | .128 | .496 |
|  | [-.052, .317] | [.125, .639] | [-.171, .054] | [-.054, .429] | [-.092, .404] | [-.138, .300] | [-.061, .452] | [-.364, .137] | [-.013, 1.932] |
|  | (.158) | (.004) | (.310) | (.128) | (.217) | (.468) | (.136) | (.373) | (.053) |
| income=17 | -.004 | -.010 | -.096 | .014 | -.010 | .061 | .032 | -.101 | -.113 |
|  | .034 | .048 | .021 | .045 | .046 | .041 | .048 | .046 | .180 |
|  | [-.071, .063] | [-.103, .084] | [-.137, -.055] | [-.074, .101] | [-.100, .080] | [-.018, .141] | [-.061, .125] | [-.192, -.010] | [-.466, .240] |
|  | (.902) | (.835) | (<.001) | (.759) | (.823) | (.130) | (.498) | (.030) | (.530) |
| Rural Code=2 | -.010 | -.015 | -.013 | -.004 | .038 | .023 | -.015 | -.013 | -.008 |
|  | .018 | .025 | .011 | .023 | .024 | .021 | .024 | .024 | .093 |
|  | [-.045, .024] | [-.064, .033] | [-.034, .009] | [-.050, .042] | [-.008, .084] | [-.018, .064] | [-.063, .033] | [-.060, .034] | [-.190, .175] |
|  | (.565) | (.536) | (.241) | (.862) | (.107) | (.271) | (.550) | (.596) | (.933) |
| Rural Code=3 | -<.001 | -.013 | -.013 | .005 | .039 | -.020 | -.012 | -.040 | -.056 |
|  | .024 | .033 | .015 | .031 | .032 | .028 | .033 | .032 | .125 |
|  | [-.046, .046] | [-.078, .052] | [-.042, .015] | [-.057, .066] | [-.023, .101] | [-.076, .035] | [-.076, .053] | [-.104, .024] | [-.302, .190] |
|  | (1.000) | (.700) | (.354) | (.880) | (.221) | (.471) | (.721) | (.217) | (.654) |
| Rural Code=4 | -.072 | .038 | .004 | -.051 | -.032 | .007 | -.083 | -.117 | -.307 |
|  | .033 | .046 | .020 | .043 | .044 | .039 | .046 | .045 | .173 |
|  | [-.136, -.008] | [-.052, .128] | [-.035, .043] | [-.136, .033] | [-.119, .054] | [-.070, .083] | [-.173, .006] | [-.204, -.029] | [-.646, .033] |
|  | (.028) | (.404) | (.840) | (.234) | (.461) | (.864) | (.068) | (.009) | (.077) |
| Rural Code=5 | -.027 | .020 | -.059 | .119 | .082 | .078 | -.079 | -.127 | .010 |
|  | .050 | .070 | .031 | .066 | .067 | .059 | .069 | .068 | .264 |
|  | [-.125, .071] | [-.116, .157] | [-.119, .001] | [-.009, .248] | [-.049, .214] | [-.038, .194] | [-.215, .057] | [-.260, .007] | [-.507, .526] |
|  | (.588) | (.771) | (.054) | (.069) | (.219) | (.188) | (.255) | (.063) | (.971) |
| Rural Code=6 | -.044 | .024 | -.032 | -.012 | .004 | -.037 | -.029 | -.006 | -.133 |
|  | .033 | .045 | .020 | .043 | .044 | .039 | .045 | .044 | .172 |
|  | [-.108, .020] | [-.065, .113] | [-.071, .007] | [-.096, .072] | [-.082, .089] | [-.113, .039] | [-.118, .059] | [-.092, .081] | [-.469, .204] |
|  | (.176) | (.598) | (.111) | (.774) | (.933) | (.339) | (.518) | (.901) | (.439) |
| Rural Code=7 | -.091 | .017 | <.001 | .005 | -.079 | -.022 | -.035 | -.081 | -.288 |
|  | .045 | .062 | .027 | .059 | .060 | .053 | .062 | .061 | .236 |
|  | [-.178, -.004] | [-.105, .140] | [-.054, .054] | [-.110, .120] | [-.197, .038] | [-.126, .082] | [-.157, .087] | [-.201, .038] | [-.750, .174] |
|  | (.041) | (.783) | (1.000) | (.929) | (.186) | (.681) | (.574) | (.181) | (.222) |
| Rural Code=8 | -.111 | -.008 | -.056 | -.153 | -.174 | -.102 | -.170 | -.172 | -.945 |
|  | .083 | .116 | .051 | .109 | .112 | .099 | .116 | .113 | .440 |
|  | [-.274, .053] | [-.236, .221] | [-.156, .044] | [-.368, .061] | [-.393, .045] | [-.296, .092] | [-.397, .058] | [-.394, .051] | [-1.807, -.083] |
|  | (.184) | (.948) | (.271) | (.160) | (.120) | (.303) | (.143) | (.130) | (.032) |
| Rural Code=9 | -.119 | -.078 | -.003 | -.033 | -.023 | -.003 | .053 | -.010 | -.217 |
|  | .071 | .099 | .043 | .093 | .095 | .084 | .098 | .096 | .374 |
|  | [-.258, .019] | [-.271, .116] | [-.088, .082] | [-.215, .149] | [-.210, .163] | [-.168, .161] | [-.140, .245] | [-.199, .178] | [-.950, .515] |
|  | (.091) | (.432) | (.946) | (.721) | (.807) | (.969) | (.594) | (.915) | (.561) |
| Alaska | .252 | .159 | -.090 | .301 | -.009 | .168 | -.031 | .205 | .953 |
|  | .141 | .198 | .087 | .185 | .190 | .168 | .197 | .192 | .747 |
|  | [-.026, .529] | [-.229, .546] | [-.260, .079] | [-.062, .665] | [-.381, .364] | [-.161, .497] | [-.417, .354] | [-.172, .582] | [-.510, 2.417] |
|  | (.075) | (.422) | (.297) | (.105) | (.962) | (.318) | (.874) | (.287) | (.202) |
| Arizona | .051 | -.180 | -.046 | -.019 | .018 | .023 | -.124 | .108 | -.169 |
|  | .067 | .094 | .041 | .089 | .090 | .080 | .094 | .092 | .356 |
|  | [-.081, .183] | [-.365, .004] | [-.127, .034] | [-.193, .155] | [-.159, .195] | [-.134, .180] | [-.308, .059] | [-.072, .288] | [-.866, .528] |
|  | (.446) | (.056) | (.261) | (.829) | (.845) | (.774) | (.185) | (.238) | (.634) |
| Arkansas | .114 | -.067 | -.042 | -.012 | .017 | .001 | -.049 | .086 | .049 |
|  | .076 | .106 | .046 | .099 | .102 | .090 | .105 | .103 | .399 |
|  | [-.034, .262] | [-.274, .140] | [-.133, .049] | [-.207, .183] | [-.182, .216] | [-.174, .177] | [-.255, .157] | [-.116, .288] | [-.734, .832] |
|  | (.133) | (.526) | (.367) | (.904) | (.865) | (.987) | (.644) | (.402) | (.902) |
| California | .073 | -.040 | -.047 | .070 | .060 | .113 | -.037 | .183 | .375 |
|  | .059 | .083 | .036 | .078 | .080 | .071 | .083 | .081 | .314 |
|  | [-.043, .190] | [-.202, .123] | [-.118, .024] | [-.083, .223] | [-.096, .217] | [-.025, .251] | [-.199, .125] | [.025, .342] | [-.240, .990] |
|  | (.216) | (.634) | (.198) | (.373) | (.449) | (.110) | (.652) | (.023) | (.232) |
| Colorado | .190 | .007 | -.064 | .181 | .156 | .170 | .054 | .159 | .851 |
|  | .076 | .106 | .047 | .100 | .102 | .090 | .106 | .104 | .402 |
|  | [.041, .339] | [-.201, .216] | [-.155, .028] | [-.015, .377] | [-.044, .356] | [-.007, .347] | [-.153, .262] | [-.044, .362] | [.064, 1.638] |
|  | (.012) | (.944) | (.171) | (.070) | (.127) | (.060) | (.607) | (.126) | (.034) |
| Connecticut | -.019 | -.053 | -.095 | .047 | .188 | .133 | -.018 | .255 | .439 |
|  | .083 | .116 | .051 | .109 | .112 | .099 | .116 | .113 | .439 |
|  | [-.182, .144] | [-.281, .175] | [-.195, .005] | [-.168, .261] | [-.031, .407] | [-.060, .327] | [-.245, .208] | [.033, .477] | [-.422, 1.299] |
|  | (.820) | (.648) | (.063) | (.670) | (.092) | (.177) | (.874) | (.025) | (.317) |
| Delaware | .176 | .212 | -.088 | -.029 | .346 | .137 | .075 | .270 | 1.093 |
|  | .107 | .150 | .065 | .141 | .143 | .127 | .148 | .146 | .564 |
|  | [-.034, .385] | [-.081, .505] | [-.217, .040] | [-.305, .247] | [.065, .627] | [-.111, .385] | [-.216, .366] | [-.016, .556] | [-.013, 2.199] |
|  | (.100) | (.156) | (.177) | (.837) | (.016) | (.280) | (.615) | (.064) | (.053) |
| District of Columbia | .053 | -.185 | -.107 | .331 | .183 | .195 | -.134 | .331 | .665 |
|  | .133 | .186 | .082 | .175 | .179 | .158 | .186 | .181 | .704 |
|  | [-.209, .314] | [-.550, .180] | [-.267, .053] | [-.012, .674] | [-.168, .534] | [-.116, .505] | [-.498, .230] | [-.024, .687] | [-.714, 2.045] |
|  | (.693) | (.320) | (.189) | (.058) | (.307) | (.219) | (.470) | (.068) | (.345) |
| Florida | .093 | .036 | -.051 | .007 | .091 | .087 | -.068 | .137 | .328 |
|  | .060 | .084 | .037 | .079 | .081 | .071 | .083 | .082 | .317 |
|  | [-.025, .210] | [-.128, .200] | [-.123, .021] | [-.148, .161] | [-.066, .249] | [-.053, .226] | [-.231, .095] | [-.023, .297] | [-.292, .949] |
|  | (.122) | (.667) | (.162) | (.930) | (.256) | (.222) | (.415) | (.093) | (.300) |
| Georgia | .057 | -.018 | -.053 | .150 | .087 | .122 | -.099 | .107 | .353 |
|  | .065 | .091 | .040 | .085 | .087 | .077 | .090 | .088 | .343 |
|  | [-.070, .185] | [-.196, .160] | [-.131, .025] | [-.017, .317] | [-.084, .258] | [-.029, .273] | [-.276, .078] | [-.066, .281] | [-.320, 1.025] |
|  | (.378) | (.843) | (.185) | (.079) | (.321) | (.114) | (.272) | (.225) | (.304) |
| Hawaii | .124 | .026 | -.003 | -.308 | -.081 | .206 | -.017 | .084 | .028 |
|  | .115 | .161 | .070 | .152 | .154 | .136 | .160 | .157 | .607 |
|  | [-.102, .349] | [-.289, .341] | [-.141, .136] | [-.606, -.011] | [-.384, .221] | [-.061, .473] | [-.330, .296] | [-.223, .392] | [-1.162, 1.218] |
|  | (.282) | (.872) | (.971) | (.042) | (.598) | (.131) | (.914) | (.591) | (.963) |
| Idaho | -.113 | -.090 | -.085 | .089 | -.092 | .004 | -.294 | -.037 | -.620 |
|  | .096 | .135 | .059 | .127 | .129 | .114 | .134 | .132 | .509 |
|  | [-.301, .075] | [-.354, .174] | [-.201, .030] | [-.160, .338] | [-.346, .161] | [-.220, .228] | [-.557, -.032] | [-.295, .221] | [-1.617, .376] |
|  | (.240) | (.504) | (.148) | (.481) | (.474) | (.971) | (.028) | (.778) | (.223) |
| Illinois | -.001 | -.185 | -.059 | .118 | .019 | .042 | -.062 | .191 | .064 |
|  | .065 | .091 | .040 | .086 | .088 | .078 | .091 | .089 | .345 |
|  | [-.129, .127] | [-.364, -.006] | [-.137, .019] | [-.050, .287] | [-.153, .191] | [-.110, .194] | [-.240, .116] | [.016, .365] | [-.612, .741] |
|  | (.992) | (.043) | (.140) | (.168) | (.827) | (.589) | (.498) | (.032) | (.852) |
| Indiana | .037 | -.126 | -.053 | .096 | .117 | .176 | -.138 | .184 | .294 |
|  | .071 | .099 | .043 | .093 | .095 | .084 | .098 | .096 | .373 |
|  | [-.101, .176] | [-.320, .068] | [-.137, .032] | [-.086, .279] | [-.069, .303] | [.012, .341] | [-.331, .055] | [-.005, .373] | [-.437, 1.026] |
|  | (.596) | (.202) | (.225) | (.299) | (.219) | (.036) | (.160) | (.056) | (.430) |
| Iowa | .175 | .008 | -.010 | .029 | -.062 | .104 | -.052 | -.054 | .137 |
|  | .084 | .117 | .051 | .110 | .113 | .100 | .117 | .114 | .444 |
|  | [.010, .340] | [-.223, .238] | [-.110, .091] | [-.187, .245] | [-.284, .159] | [-.091, .300] | [-.282, .177] | [-.278, .170] | [-.732, 1.007] |
|  | (.037) | (.949) | (.852) | (.793) | (.581) | (.297) | (.654) | (.636) | (.757) |
| Kansas | .196 | -.160 | -.099 | -.013 | .083 | .181 | -.066 | .100 | .222 |
|  | .102 | .142 | .062 | .134 | .137 | .121 | .141 | .139 | .537 |
|  | [-.004, .395] | [-.438, .119] | [-.221, .023] | [-.275, .249] | [-.185, .351] | [-.055, .418] | [-.343, .211] | [-.172, .371] | [-.830, 1.275] |
|  | (.054) | (.262) | (.113) | (.925) | (.543) | (.133) | (.640) | (.471) | (.679) |
| Kentucky | .140 | -.111 | -.047 | -.161 | .130 | .067 | -.053 | .003 | -.031 |
|  | .077 | .108 | .047 | .101 | .103 | .091 | .107 | .105 | .406 |
|  | [-.011, .290] | [-.322, .099] | [-.140, .045] | [-.360, .037] | [-.072, .333] | [-.112, .246] | [-.263, .157] | [-.202, .208] | [-.827, .765] |
|  | (.069) | (.300) | (.316) | (.110) | (.208) | (.460) | (.620) | (.978) | (.939) |
| Louisiana | .104 | -.102 | -.079 | -.080 | .145 | .164 | -.038 | .106 | .222 |
|  | .077 | .108 | .047 | .102 | .104 | .092 | .107 | .105 | .408 |
|  | [-.047, .256] | [-.313, .110] | [-.171, .014] | [-.279, .119] | [-.058, .348] | [-.016, .343] | [-.248, .173] | [-.100, .312] | [-.577, 1.021] |
|  | (.175) | (.347) | (.097) | (.430) | (.161) | (.074) | (.726) | (.314) | (.585) |
| Maine | .122 | -.222 | -.083 | .170 | .234 | .225 | .273 | .231 | .949 |
|  | .107 | .150 | .066 | .141 | .144 | .127 | .149 | .146 | .565 |
|  | [-.088, .331] | [-.515, .072] | [-.211, .045] | [-.106, .446] | [-.048, .515] | [-.023, .474] | [-.019, .564] | [-.055, .517] | [-.158, 2.057] |
|  | (.255) | (.139) | (.205) | (.227) | (.104) | (.076) | (.067) | (.114) | (.093) |
| Maryland | .123 | -.023 | -.077 | .007 | .092 | .176 | -.034 | -.014 | .250 |
|  | .076 | .107 | .047 | .100 | .103 | .091 | .106 | .104 | .404 |
|  | [-.027, .273] | [-.233, .186] | [-.168, .015] | [-.190, .203] | [-.109, .294] | [-.002, .353] | [-.242, .175] | [-.218, .190] | [-.541, 1.041] |
|  | (.107) | (.829) | (.101) | (.948) | (.368) | (.053) | (.752) | (.895) | (.536) |
| Massachusetts | .058 | -.120 | -.084 | .114 | .158 | .175 | -.171 | .173 | .302 |
|  | .072 | .101 | .044 | .095 | .097 | .086 | .100 | .098 | .381 |
|  | [-.083, .199] | [-.317, .078] | [-.171, .003] | [-.072, .300] | [-.032, .348] | [.008, .343] | [-.368, .025] | [-.019, .366] | [-.445, 1.048] |
|  | (.423) | (.235) | (.057) | (.230) | (.102) | (.040) | (.088) | (.078) | (.428) |
| Michigan | .106 | -.078 | -.027 | -.046 | .105 | .087 | -.101 | .083 | .128 |
|  | .064 | .090 | .039 | .085 | .087 | .077 | .090 | .088 | .340 |
|  | [-.020, .232] | [-.255, .098] | [-.104, .051] | [-.212, .120] | [-.065, .274] | [-.063, .237] | [-.276, .075] | [-.089, .256] | [-.539, .795] |
|  | (.100) | (.384) | (.497) | (.584) | (.226) | (.253) | (.262) | (.342) | (.706) |
| Minnesota | .122 | -.115 | -.072 | .075 | .105 | .108 | -.038 | .137 | .320 |
|  | .076 | .106 | .046 | .099 | .101 | .090 | .105 | .103 | .399 |
|  | [-.026, .270] | [-.322, .092] | [-.163, .018] | [-.120, .269] | [-.094, .304] | [-.068, .284] | [-.244, .168] | [-.065, .338] | [-.462, 1.102] |
|  | (.107) | (.275) | (.117) | (.452) | (.302) | (.228) | (.719) | (.184) | (.423) |
| Mississippi | .086 | -.077 | -.024 | -.105 | .138 | .074 | .044 | .241 | .377 |
|  | .085 | .119 | .052 | .112 | .115 | .102 | .119 | .116 | .451 |
|  | [-.082, .253] | [-.311, .157] | [-.127, .079] | [-.325, .115] | [-.087, .363] | [-.125, .273] | [-.189, .277] | [.013, .469] | [-.508, 1.261] |
|  | (.317) | (.519) | (.646) | (.349) | (.229) | (.465) | (.712) | (.039) | (.404) |
| Missouri | .018 | -.125 | -.040 | -.001 | .033 | .153 | -.091 | .043 | -.011 |
|  | .069 | .097 | .042 | .091 | .093 | .082 | .096 | .094 | .366 |
|  | [-.118, .153] | [-.315, .065] | [-.123, .043] | [-.180, .177] | [-.149, .216] | [-.008, .314] | [-.280, .098] | [-.142, .228] | [-.728, .705] |
|  | (.800) | (.197) | (.344) | (.987) | (.719) | (.063) | (.345) | (.651) | (.975) |
| Montana | .147 | -.083 | -.064 | .085 | .070 | .128 | -.181 | .153 | .256 |
|  | .107 | .149 | .065 | .141 | .143 | .127 | .148 | .146 | .564 |
|  | [-.062, .356] | [-.376, .210] | [-.192, .064] | [-.190, .361] | [-.211, .351] | [-.120, .376] | [-.472, .110] | [-.132, .438] | [-.849, 1.361] |
|  | (.168) | (.578) | (.330) | (.544) | (.626) | (.312) | (.222) | (.294) | (.650) |
| Nebraska | .096 | -.034 | -.090 | -.006 | .112 | .123 | -.029 | .025 | .197 |
|  | .110 | .154 | .067 | .144 | .148 | .131 | .153 | .149 | .580 |
|  | [-.119, .312] | [-.334, .267] | [-.221, .042] | [-.289, .276] | [-.177, .402] | [-.133, .378] | [-.329, .271] | [-.268, .318] | [-.940, 1.334] |
|  | (.380) | (.827) | (.183) | (.966) | (.448) | (.348) | (.850) | (.867) | (.734) |
| Nevada | .066 | -.162 | -.053 | -.044 | .215 | .140 | -.041 | .246 | .368 |
|  | .075 | .105 | .046 | .099 | .101 | .089 | .105 | .103 | .398 |
|  | [-.081, .214] | [-.369, .045] | [-.143, .038] | [-.239, .151] | [.016, .413] | [-.035, .316] | [-.247, .164] | [.044, .447] | [-.412, 1.148] |
|  | (.379) | (.125) | (.254) | (.657) | (.034) | (.116) | (.694) | (.017) | (.355) |
| New Hampshire | .148 | -.190 | -.037 | -.082 | .025 | .071 | -.048 | -.005 | -.119 |
|  | .090 | .127 | .055 | .119 | .121 | .107 | .126 | .123 | .478 |
|  | [-.029, .325] | [-.438, .058] | [-.145, .072] | [-.316, .151] | [-.213, .263] | [-.139, .281] | [-.295, .198] | [-.246, .237] | [-1.055, .817] |
|  | (.101) | (.134) | (.507) | (.490) | (.837) | (.507) | (.701) | (.970) | (.803) |
| New Jersey | .112 | .020 | -.043 | .064 | .125 | .140 | -.038 | .057 | .436 |
|  | .070 | .098 | .043 | .092 | .094 | .083 | .097 | .095 | .369 |
|  | [-.025, .249] | [-.171, .212] | [-.127, .041] | [-.116, .243] | [-.059, .309] | [-.023, .303] | [-.228, .153] | [-.129, .244] | [-.287, 1.160] |
|  | (.109) | (.836) | (.311) | (.489) | (.184) | (.092) | (.700) | (.548) | (.237) |
| New Mexico | .113 | -.123 | -.056 | .048 | .124 | -.047 | .062 | .141 | .258 |
|  | .083 | .116 | .051 | .110 | .112 | .099 | .116 | .114 | .440 |
|  | [-.051, .276] | [-.351, .106] | [-.156, .043] | [-.166, .263] | [-.095, .343] | [-.241, .147] | [-.165, .289] | [-.082, .363] | [-.604, 1.120] |
|  | (.176) | (.292) | (.268) | (.658) | (.268) | (.635) | (.593) | (.216) | (.558) |
| New York | .020 | .019 | -.030 | .053 | .090 | .144 | -.034 | .104 | .367 |
|  | .061 | .086 | .037 | .080 | .082 | .073 | .085 | .083 | .323 |
|  | [-.100, .139] | [-.148, .187] | [-.104, .043] | [-.104, .211] | [-.071, .251] | [.002, .286] | [-.201, .133] | [-.060, .267] | [-.266, 1.000] |
|  | (.749) | (.822) | (.418) | (.509) | (.272) | (.048) | (.688) | (.213) | (.256) |
| North Carolina | .098 | .020 | -.035 | .055 | .107 | .137 | .006 | .059 | .446 |
|  | .066 | .092 | .040 | .087 | .089 | .078 | .092 | .090 | .348 |
|  | [-.031, .228] | [-.160, .201] | [-.114, .044] | [-.115, .225] | [-.067, .281] | [-.017, .290] | [-.174, .185] | [-.117, .235] | [-.237, 1.129] |
|  | (.136) | (.824) | (.387) | (.523) | (.227) | (.080) | (.951) | (.514) | (.200) |
| North Dakota | .093 | .322 | -.084 | -.050 | .010 | .211 | -.013 | .152 | .640 |
|  | .134 | .187 | .082 | .176 | .180 | .159 | .186 | .183 | .708 |
|  | [-.169, .356] | [-.045, .690] | [-.245, .076] | [-.396, .296] | [-.342, .363] | [-.101, .522] | [-.378, .352] | [-.207, .510] | [-.747, 2.026] |
|  | (.486) | (.085) | (.304) | (.777) | (.955) | (.185) | (.942) | (.407) | (.366) |
| Ohio | .107 | -.036 | -.045 | .050 | .095 | .154 | -.012 | .151 | .464 |
|  | .064 | .090 | .039 | .084 | .086 | .076 | .089 | .087 | .339 |
|  | [-.018, .233] | [-.212, .140] | [-.122, .031] | [-.116, .215] | [-.074, .264] | [.005, .303] | [-.186, .163] | [-.021, .322] | [-.200, 1.128] |
|  | (.094) | (.688) | (.247) | (.556) | (.270) | (.043) | (.897) | (.085) | (.171) |
| Oklahoma | .046 | .084 | .020 | .081 | .146 | .109 | -.079 | .115 | .522 |
|  | .086 | .120 | .053 | .113 | .116 | .102 | .120 | .117 | .454 |
|  | [-.122, .215] | [-.151, .320] | [-.083, .123] | [-.140, .302] | [-.080, .373] | [-.091, .309] | [-.314, .155] | [-.114, .345] | [-.367, 1.412] |
|  | (.591) | (.482) | (.706) | (.473) | (.205) | (.287) | (.507) | (.324) | (.250) |
| Oregon | .020 | -.123 | -.072 | .063 | .068 | .126 | -.045 | .151 | .190 |
|  | .071 | .099 | .043 | .094 | .095 | .084 | .099 | .097 | .375 |
|  | [-.119, .159] | [-.317, .072] | [-.157, .014] | [-.121, .246] | [-.119, .255] | [-.039, .291] | [-.238, .149] | [-.039, .341] | [-.545, .925] |
|  | (.773) | (.217) | (.100) | (.502) | (.475) | (.134) | (.651) | (.118) | (.612) |
| Pennsylvania | .111 | -.117 | -.056 | .036 | .100 | .154 | -.079 | .109 | .256 |
|  | .061 | .085 | .037 | .080 | .082 | .072 | .085 | .083 | .321 |
|  | [-.008, .230] | [-.284, .049] | [-.129, .017] | [-.121, .192] | [-.060, .260] | [.013, .295] | [-.245, .086] | [-.053, .271] | [-.373, .885] |
|  | (.068) | (.167) | (.134) | (.653) | (.220) | (.033) | (.348) | (.189) | (.425) |
| Rhode Island | .104 | .312 | .034 | .150 | .197 | .108 | -.099 | .136 | .943 |
|  | .133 | .186 | .081 | .175 | .179 | .158 | .185 | .181 | .703 |
|  | [-.157, .366] | [-.052, .677] | [-.125, .194] | [-.192, .492] | [-.153, .548] | [-.201, .418] | [-.462, .264] | [-.219, .490] | [-.435, 2.321] |
|  | (.433) | (.093) | (.674) | (.391) | (.270) | (.493) | (.593) | (.454) | (.180) |
| South Carolina | .100 | .009 | -.073 | .041 | .187 | .196 | .034 | .142 | .633 |
|  | .074 | .104 | .046 | .098 | .100 | .088 | .103 | .102 | .393 |
|  | [-.046, .246] | [-.195, .213] | [-.162, .016] | [-.151, .233] | [-.009, .383] | [.023, .369] | [-.169, .236] | [-.057, .341] | [-.138, 1.403] |
|  | (.178) | (.931) | (.109) | (.676) | (.061) | (.027) | (.745) | (.163) | (.107) |
| South Dakota | -.023 | -.317 | -.089 | -.104 | -.139 | .092 | -.198 | -.183 | -.961 |
|  | .123 | .172 | .075 | .162 | .165 | .146 | .171 | .168 | .651 |
|  | [-.264, .219] | [-.655, .020] | [-.237, .059] | [-.422, .213] | [-.463, .185] | [-.195, .379] | [-.533, .138] | [-.512, .146] | [-2.236, .314] |
|  | (.854) | (.065) | (.237) | (.520) | (.401) | (.529) | (.249) | (.276) | (.140) |
| Tennessee | .158 | .093 | -.047 | .044 | .099 | .227 | .073 | .136 | .783 |
|  | .073 | .102 | .045 | .096 | .098 | .086 | .101 | .099 | .384 |
|  | [.015, .300] | [-.106, .292] | [-.134, .040] | [-.143, .232] | [-.093, .290] | [.058, .396] | [-.125, .271] | [-.058, .330] | [.030, 1.536] |
|  | (.030) | (.361) | (.293) | (.643) | (.311) | (.009) | (.470) | (.170) | (.042) |
| Texas | .057 | -.007 | -.035 | .036 | .016 | .049 | -.146 | .009 | -.020 |
|  | .061 | .085 | .037 | .080 | .082 | .072 | .085 | .083 | .321 |
|  | [-.062, .176] | [-.173, .160] | [-.108, .038] | [-.120, .193] | [-.144, .176] | [-.092, .191] | [-.311, .020] | [-.153, .172] | [-.649, .610] |
|  | (.348) | (.935) | (.347) | (.649) | (.846) | (.493) | (.085) | (.910) | (.951) |
| Utah | .067 | -.140 | -.076 | .150 | .036 | .116 | -.039 | .115 | .227 |
|  | .084 | .118 | .052 | .111 | .113 | .100 | .117 | .115 | .445 |
|  | [-.098, .232] | [-.371, .091] | [-.177, .025] | [-.067, .368] | [-.185, .258] | [-.080, .312] | [-.268, .191] | [-.111, .340] | [-.646, 1.100] |
|  | (.429) | (.236) | (.142) | (.176) | (.748) | (.247) | (.741) | (.319) | (.610) |
| Vermont | .235 | -.140 | -.081 | .219 | .179 | .008 | .056 | -.106 | .370 |
|  | .122 | .170 | .075 | .160 | .164 | .145 | .170 | .166 | .643 |
|  | [-.004, .474] | [-.474, .193] | [-.227, .065] | [-.094, .532] | [-.142, .500] | [-.276, .291] | [-.277, .388] | [-.430, .219] | [-.890, 1.631] |
|  | (.054) | (.410) | (.277) | (.171) | (.274) | (.956) | (.743) | (.523) | (.565) |
| Virginia | .077 | .037 | -.049 | .041 | .051 | .152 | -.035 | .153 | .427 |
|  | .065 | .091 | .040 | .086 | .088 | .077 | .091 | .089 | .344 |
|  | [-.051, .205] | [-.141, .216] | [-.127, .029] | [-.128, .209] | [-.121, .222] | [.000, .303] | [-.213, .143] | [-.021, .328] | [-.248, 1.101] |
|  | (.237) | (.681) | (.221) | (.636) | (.563) | (.050) | (.699) | (.084) | (.215) |
| Washington | .114 | -.085 | -.016 | .064 | .149 | .184 | -.027 | .113 | .494 |
|  | .068 | .096 | .042 | .090 | .092 | .081 | .095 | .093 | .361 |
|  | [-.020, .248] | [-.272, .102] | [-.098, .066] | [-.112, .240] | [-.031, .328] | [.026, .343] | [-.213, .159] | [-.070, .296] | [-.213, 1.201] |
|  | (.096) | (.373) | (.710) | (.476) | (.105) | (.023) | (.778) | (.225) | (.171) |
| West Virginia | -.010 | -.038 | -.036 | .059 | -.034 | .039 | -.149 | -.013 | -.183 |
|  | .087 | .122 | .053 | .114 | .117 | .103 | .121 | .118 | .459 |
|  | [-.180, .161] | [-.276, .200] | [-.140, .069] | [-.165, .282] | [-.263, .195] | [-.163, .241] | [-.386, .088] | [-.245, .219] | [-1.083, .717] |
|  | (.910) | (.754) | (.502) | (.608) | (.769) | (.707) | (.217) | (.915) | (.690) |
| Wisconsin | .073 | -.077 | -.031 | .095 | .082 | .135 | -.105 | .033 | .206 |
|  | .071 | .099 | .043 | .093 | .095 | .084 | .098 | .096 | .373 |
|  | [-.066, .211] | [-.270, .117] | [-.116, .053] | [-.087, .277] | [-.104, .268] | [-.029, .300] | [-.298, .088] | [-.156, .222] | [-.525, .937] |
|  | (.303) | (.439) | (.469) | (.305) | (.387) | (.107) | (.286) | (.731) | (.581) |
| Wyoming | .271 | .249 | .238 | .036 | .144 | .067 | .157 | .487 | 1.655 |
|  | .205 | .289 | .126 | .273 | .276 | .244 | .285 | .283 | 1.088 |
|  | [-.131, .673] | [-.318, .815] | [-.010, .486] | [-.500, .572] | [-.396, .684] | [-.410, .544] | [-.402, .716] | [-.066, 1.041] | [-.476, 3.787] |
|  | (.186) | (.389) | (.059) | (.896) | (.600) | (.783) | (.582) | (.085) | (.128) |
| N | 2982 | 2982 | 2982 | 2982 | 2982 | 2982 | 2982 | 2982 | 2982 |
| Pseudo-R^2^ |  |  |  |  |  |  |  |  |  |
| Model χ^2^ | 212.0 | 203.3 | 114.7 | 32.7 | 214.3 | 228.0 | 313.1 | 214.1 | 384.5 |
| d.f. | 88 | 88 | 88 | 88 | 88 | 88 | 88 | 88 | 88 |
| p-value | <.001 | <.001 | <.001 | <.001 | <.001 | <.001 | <.001 | <.001 | <.001 |

S9 Table. Regression Results, Health Behavior, Multilevel Models, Controlling for Positive Diagnoses.

|  | (1) | (2) | (3) | (4) | (5) | (6) | (7) | (8) | (9) |
| --- | --- | --- | --- | --- | --- | --- | --- | --- | --- |
|  | washhands | boughtsanitizer | visitdoctor | changedtravel | avoidcontact | avoidgatherings | soughtinfo | selfquarantine | changehealth |
| main |  |  |  |  |  |  |  |  |  |
| Democrat | .084 | .083 | -.006 | .065 | .170 | .108 | .193 | .119 | .814 |
|  | .017 | .023 | .010 | .022 | .022 | .020 | .023 | .023 | .088 |
|  | [.051, .116] | [.037, .128] | [-.026, .014] | [.022, .108] | [.126, .213] | [.069, .147] | [.147, .238] | [.075, .163] | [.641, .986] |
|  | (<.001) | (<.001) | (.537) | (.003) | (<.001) | (<.001) | (<.001) | (<.001) | (<.001) |
| Other | -.010 | -.003 | -.020 | -.003 | .049 | .024 | .069 | .029 | .134 |
|  | .017 | .024 | .010 | .022 | .023 | .020 | .023 | .023 | .089 |
|  | [-.043, .023] | [-.049, .043] | [-.040, .000] | [-.047, .040] | [.004, .093] | [-.015, .064] | [.023, .115] | [-.016, .074] | [-.041, .309] |
|  | (.546) | (.899) | (.053) | (.883) | (.032) | (.226) | (.003) | (.206) | (.133) |
| Local COVID-19 Cases | <.001 | <.001 | <.001 | -<.001 | <.001 | <.001 | -<.001 | -<.001 | <.001 |
|  | <.001 | <.001 | <.001 | <.001 | <.001 | <.001 | <.001 | <.001 | <.001 |
|  | [-<.001, .000] | [.000, .000] | [-<.001, .000] | [-<.001, .000] | [-<.001, .000] | [-<.001, .000] | [-<.001, .000] | [-<.001, .000] | [-<.001, .000] |
|  | (.127) | (.026) | (.951) | (.456) | (.763) | (.455) | (.117) | (.829) | (.631) |
| 35- | .007 | .052 | .001 | -.049 | .001 | -.011 | -.009 | -.043 | -.052 |
|  | .019 | .026 | .011 | .025 | .025 | .022 | .026 | .025 | .099 |
|  | [-.029, .044] | [.001, .103] | [-.021, .024] | [-.097, -.001] | [-.048, .050] | [-.055, .032] | [-.060, .042] | [-.093, .006] | [-.246, .142] |
|  | (.689) | (.047) | (.917) | (.046) | (.971) | (.617) | (.734) | (.088) | (.599) |
| 50- | .025 | .052 | -.008 | -.067 | .017 | .053 | -.035 | -.073 | -.036 |
|  | .018 | .025 | .011 | .023 | .024 | .021 | .025 | .024 | .093 |
|  | [-.010, .059] | [.004, .101] | [-.029, .013] | [-.112, -.021] | [-.029, .064] | [.012, .094] | [-.083, .013] | [-.120, -.025] | [-.219, .147] |
|  | (.159) | (.034) | (.471) | (.004) | (.466) | (.012) | (.157) | (.003) | (.701) |
| 70- | .061 | .068 | -.012 | -.024 | .103 | .140 | -.071 | .063 | .327 |
|  | .022 | .031 | .014 | .029 | .030 | .027 | .031 | .030 | .118 |
|  | [.017, .104] | [.007, .129] | [-.038, .015] | [-.082, .033] | [.044, .162] | [.088, .193] | [-.132, -.010] | [.004, .123] | [.096, .559] |
|  | (.007) | (.029) | (.394) | (.407) | (.001) | (<.001) | (.023) | (.037) | (.006) |
| gender | .041 | .016 | -.002 | -.012 | .007 | .042 | .036 | .060 | .186 |
|  | .013 | .018 | .008 | .017 | .017 | .015 | .018 | .017 | .068 |
|  | [.016, .066] | [-.020, .051] | [-.018, .013] | [-.045, .021] | [-.027, .040] | [.012, .072] | [.001, .071] | [.025, .094] | [.052, .319] |
|  | (.002) | (.382) | (.758) | (.470) | (.704) | (.006) | (.046) | (.001) | (.006) |
| white | -.010 | -.082 | -.031 | -.031 | .043 | .051 | .079 | .002 | .021 |
|  | .015 | .021 | .009 | .020 | .021 | .018 | .021 | .021 | .081 |
|  | [-.040, .020] | [-.124, -.039] | [-.050, -.013] | [-.071, .009] | [.003, .084] | [.015, .086] | [.037, .121] | [-.039, .043] | [-.139, .180] |
|  | (.515) | (<.001) | (.001) | (.124) | (.037) | (.006) | (<.001) | (.906) | (.799) |
| married | -.002 | .046 | .014 | .043 | .032 | -.004 | .021 | .011 | .161 |
|  | .014 | .020 | .009 | .019 | .019 | .017 | .020 | .019 | .075 |
|  | [-.030, .026] | [.007, .085] | [-.002, .031] | [.007, .080] | [-.005, .069] | [-.037, .029] | [-.018, .059] | [-.026, .049] | [.015, .308] |
|  | (.876) | (.020) | (.094) | (.020) | (.093) | (.798) | (.294) | (.551) | (.031) |
| High school graduate | -.014 | .006 | -.001 | .081 | -.121 | -.038 | .001 | .002 | -.083 |
|  | .038 | .053 | .023 | .049 | .051 | .045 | .052 | .051 | .199 |
|  | [-.088, .060] | [-.097, .109] | [-.047, .044] | [-.016, .178] | [-.220, -.021] | [-.125, .050] | [-.102, .104] | [-.099, .102] | [-.473, .307] |
|  | (.707) | (.905) | (.953) | (.102) | (.017) | (.402) | (.983) | (.972) | (.676) |
| Some college | .026 | -.008 | -.017 | .167 | -.060 | .025 | .136 | .017 | .287 |
|  | .038 | .054 | .024 | .050 | .052 | .046 | .053 | .052 | .203 |
|  | [-.050, .101] | [-.113, .097] | [-.063, .029] | [.068, .266] | [-.161, .042] | [-.065, .114] | [.032, .241] | [-.086, .119] | [-.110, .685] |
|  | (.505) | (.883) | (.478) | (.001) | (.248) | (.589) | (.011) | (.752) | (.157) |
| 2-year | .020 | .008 | -.006 | .123 | -.067 | .019 | .126 | -.007 | .218 |
|  | .040 | .056 | .025 | .053 | .054 | .048 | .056 | .055 | .213 |
|  | [-.059, .099] | [-.102, .119] | [-.055, .042] | [.019, .226] | [-.173, .040] | [-.075, .113] | [.016, .237] | [-.114, .101] | [-.200, .636] |
|  | (.620) | (.881) | (.794) | (.020) | (.219) | (.695) | (.024) | (.902) | (.307) |
| 4-year | .068 | -.029 | .014 | .201 | -.036 | .056 | .158 | .029 | .461 |
|  | .039 | .055 | .024 | .051 | .053 | .047 | .055 | .053 | .207 |
|  | [-.009, .144] | [-.137, .078] | [-.033, .061] | [.100, .302] | [-.140, .067] | [-.035, .148] | [.051, .265] | [-.076, .133] | [.055, .867] |
|  | (.085) | (.595) | (.560) | (<.001) | (.490) | (.226) | (.004) | (.593) | (.026) |
| Post-grad | .054 | -.017 | .019 | .304 | .021 | .084 | .227 | .069 | .762 |
|  | .041 | .058 | .025 | .054 | .055 | .049 | .057 | .056 | .218 |
|  | [-.027, .135] | [-.130, .096] | [-.031, .068] | [.198, .409] | [-.088, .129] | [-.012, .180] | [.115, .340] | [-.041, .179] | [.336, 1.189] |
|  | (.192) | (.766) | (.461) | (<.001) | (.710) | (.087) | (<.001) | (.219) | (<.001) |
| income=2 | -.013 | -.036 | -.080 | -.011 | -.001 | -.022 | .021 | -.064 | -.203 |
|  | .036 | .051 | .022 | .048 | .049 | .043 | .051 | .049 | .192 |
|  | [-.084, .058] | [-.136, .063] | [-.123, -.036] | [-.104, .083] | [-.097, .095] | [-.107, .063] | [-.078, .121] | [-.161, .033] | [-.579, .173] |
|  | (.723) | (.477) | (<.001) | (.823) | (.985) | (.611) | (.671) | (.193) | (.290) |
| income=3 | -.032 | -.048 | -.061 | -.025 | -.036 | .010 | .049 | -.127 | -.269 |
|  | .035 | .049 | .022 | .046 | .047 | .042 | .049 | .048 | .186 |
|  | [-.101, .037] | [-.144, .049] | [-.104, -.019] | [-.115, .065] | [-.129, .057] | [-.072, .092] | [-.047, .145] | [-.221, -.033] | [-.634, .095] |
|  | (.360) | (.333) | (.004) | (.586) | (.445) | (.815) | (.317) | (.008) | (.148) |
| income=4 | .022 | .067 | -.067 | -.005 | .037 | .068 | .102 | -.127 | .097 |
|  | .036 | .050 | .022 | .047 | .048 | .043 | .050 | .049 | .190 |
|  | [-.049, .092] | [-.032, .166] | [-.111, -.024] | [-.097, .088] | [-.058, .132] | [-.016, .152] | [.003, .200] | [-.223, -.031] | [-.276, .471] |
|  | (.550) | (.185) | (.002) | (.920) | (.440) | (.112) | (.042) | (.010) | (.609) |
| income=5 | .035 | -.015 | -.065 | -.048 | -.013 | .053 | .121 | -.130 | -.062 |
|  | .037 | .051 | .023 | .048 | .050 | .044 | .051 | .050 | .194 |
|  | [-.037, .107] | [-.116, .086] | [-.109, -.021] | [-.143, .046] | [-.110, .084] | [-.033, .138] | [.020, .221] | [-.228, -.031] | [-.443, .320] |
|  | (.345) | (.770) | (.004) | (.319) | (.799) | (.230) | (.019) | (.010) | (.751) |
| income=6 | .042 | .007 | -.059 | -.016 | .048 | .083 | .071 | -.118 | .061 |
|  | .037 | .052 | .023 | .049 | .050 | .044 | .052 | .051 | .197 |
|  | [-.031, .115] | [-.095, .109] | [-.103, -.014] | [-.112, .079] | [-.050, .146] | [-.004, .170] | [-.031, .172] | [-.217, -.019] | [-.325, .446] |
|  | (.255) | (.893) | (.010) | (.741) | (.340) | (.060) | (.173) | (.020) | (.758) |
| income=7 | .028 | -.004 | -.052 | .108 | .001 | .080 | .112 | -.149 | .124 |
|  | .040 | .056 | .024 | .052 | .054 | .047 | .055 | .054 | .211 |
|  | [-.050, .106] | [-.113, .105] | [-.100, -.004] | [.006, .211] | [-.104, .106] | [-.013, .173] | [.003, .221] | [-.255, -.043] | [-.289, .536] |
|  | (.487) | (.942) | (.034) | (.038) | (.990) | (.091) | (.044) | (.006) | (.557) |
| income=8 | -.002 | -.075 | -.088 | .055 | -.029 | .049 | .009 | -.081 | -.162 |
|  | .039 | .054 | .024 | .051 | .052 | .046 | .054 | .053 | .205 |
|  | [-.078, .074] | [-.181, .031] | [-.134, -.041] | [-.045, .155] | [-.131, .074] | [-.042, .139] | [-.097, .114] | [-.184, .023] | [-.564, .239] |
|  | (.960) | (.166) | (<.001) | (.280) | (.583) | (.292) | (.875) | (.126) | (.428) |
| income=9 | .072 | .055 | -.082 | .053 | .016 | .049 | .110 | -.149 | .126 |
|  | .038 | .054 | .023 | .050 | .052 | .046 | .053 | .052 | .203 |
|  | [-.003, .148] | [-.050, .160] | [-.128, -.036] | [-.046, .151] | [-.085, .117] | [-.041, .138] | [.006, .215] | [-.252, -.047] | [-.272, .523] |
|  | (.059) | (.302) | (<.001) | (.293) | (.756) | (.285) | (.039) | (.004) | (.535) |
| income=10 | .030 | -.030 | -.085 | .087 | .054 | .074 | .030 | -.142 | .019 |
|  | .040 | .056 | .024 | .052 | .054 | .047 | .056 | .054 | .211 |
|  | [-.048, .108] | [-.139, .080] | [-.133, -.037] | [-.016, .189] | [-.051, .159] | [-.019, .167] | [-.079, .139] | [-.248, -.035] | [-.394, .432] |
|  | (.452) | (.596) | (.001) | (.097) | (.317) | (.119) | (.588) | (.009) | (.926) |
| income=11 | .017 | .045 | -.088 | .108 | .013 | .050 | .070 | -.112 | .104 |
|  | .041 | .057 | .025 | .054 | .055 | .049 | .057 | .056 | .216 |
|  | [-.064, .097] | [-.067, .157] | [-.137, -.039] | [.003, .214] | [-.095, .120] | [-.045, .146] | [-.041, .182] | [-.221, -.003] | [-.320, .528] |
|  | (.686) | (.430) | (<.001) | (.044) | (.820) | (.303) | (.217) | (.044) | (.630) |
| income=12 | .073 | .048 | -.074 | .107 | .062 | .068 | .122 | -.108 | .300 |
|  | .045 | .063 | .028 | .059 | .061 | .054 | .063 | .061 | .238 |
|  | [-.016, .161] | [-.075, .172] | [-.128, -.020] | [-.009, .223] | [-.056, .181] | [-.037, .173] | [-.001, .245] | [-.228, .013] | [-.166, .766] |
|  | (.106) | (.444) | (.007) | (.071) | (.302) | (.201) | (.052) | (.079) | (.208) |
| income=13 | .050 | .005 | -.130 | .214 | .066 | .123 | .237 | -.065 | .503 |
|  | .062 | .087 | .038 | .082 | .084 | .074 | .087 | .085 | .329 |
|  | [-.072, .173] | [-.166, .176] | [-.204, -.055] | [.054, .374] | [-.098, .231] | [-.022, .268] | [.067, .407] | [-.231, .101] | [-.143, 1.148] |
|  | (.419) | (.953) | (.001) | (.009) | (.428) | (.096) | (.006) | (.444) | (.127) |
| income=14 | -.011 | .081 | -.030 | .316 | .101 | .100 | .075 | -.033 | .598 |
|  | .066 | .092 | .040 | .087 | .089 | .078 | .092 | .090 | .349 |
|  | [-.141, .118] | [-.100, .261] | [-.109, .049] | [.147, .486] | [-.073, .275] | [-.053, .254] | [-.106, .255] | [-.209, .143] | [-.085, 1.282] |
|  | (.864) | (.383) | (.458) | (<.001) | (.256) | (.200) | (.417) | (.717) | (.086) |
| income=15 | .119 | .197 | .176 | .265 | .316 | .222 | .433 | .184 | 1.919 |
|  | .200 | .279 | .122 | .262 | .269 | .238 | .278 | .272 | 1.056 |
|  | [-.274, .511] | [-.351, .744] | [-.064, .416] | [-.248, .779] | [-.211, .843] | [-.244, .688] | [-.113, .978] | [-.348, .717] | [-.151, 3.989] |
|  | (.553) | (.481) | (.150) | (.311) | (.240) | (.350) | (.120) | (.498) | (.069) |
| income=16 | .133 | .382 | -.058 | .187 | .156 | .081 | .195 | -.114 | .960 |
|  | .094 | .131 | .058 | .123 | .126 | .112 | .131 | .128 | .496 |
|  | [-.052, .317] | [.125, .639] | [-.171, .054] | [-.054, .429] | [-.092, .403] | [-.138, .300] | [-.061, .451] | [-.364, .136] | [-.013, 1.932] |
|  | (.158) | (.004) | (.310) | (.128) | (.217) | (.468) | (.136) | (.372) | (.053) |
| income=17 | -.004 | -.009 | -.096 | .013 | -.010 | .062 | .031 | -.101 | -.112 |
|  | .034 | .048 | .021 | .045 | .046 | .041 | .048 | .046 | .180 |
|  | [-.071, .063] | [-.102, .085] | [-.137, -.055] | [-.074, .101] | [-.100, .080] | [-.018, .141] | [-.062, .124] | [-.192, -.010] | [-.466, .241] |
|  | (.915) | (.854) | (<.001) | (.766) | (.826) | (.128) | (.510) | (.029) | (.533) |
| Rural Code=2 | -.011 | -.017 | -.013 | -.005 | .037 | .022 | -.015 | -.016 | -.018 |
|  | .018 | .025 | .011 | .023 | .024 | .021 | .024 | .024 | .093 |
|  | [-.046, .023] | [-.066, .031] | [-.034, .008] | [-.050, .041] | [-.009, .084] | [-.019, .063] | [-.063, .033] | [-.063, .031] | [-.200, .164] |
|  | (.516) | (.478) | (.231) | (.836) | (.112) | (.300) | (.538) | (.500) | (.844) |
| Rural Code=3 | -.002 | -.015 | -.014 | .004 | .038 | -.022 | -.012 | -.044 | -.068 |
|  | .024 | .033 | .015 | .031 | .032 | .028 | .033 | .032 | .125 |
|  | [-.048, .045] | [-.080, .050] | [-.042, .015] | [-.057, .065] | [-.024, .101] | [-.077, .033] | [-.077, .052] | [-.107, .020] | [-.313, .177] |
|  | (.949) | (.643) | (.344) | (.901) | (.229) | (.436) | (.712) | (.175) | (.587) |
| Rural Code=4 | -.074 | .035 | .004 | -.052 | -.033 | .005 | -.083 | -.121 | -.319 |
|  | .033 | .046 | .020 | .043 | .044 | .039 | .046 | .045 | .173 |
|  | [-.138, -.009] | [-.055, .125] | [-.036, .043] | [-.136, .032] | [-.119, .053] | [-.071, .081] | [-.173, .006] | [-.208, -.033] | [-.658, .019] |
|  | (.024) | (.443) | (.852) | (.226) | (.449) | (.901) | (.067) | (.007) | (.065) |
| Rural Code=5 | -.029 | .018 | -.059 | .118 | .082 | .076 | -.080 | -.131 | -.004 |
|  | .050 | .070 | .031 | .066 | .067 | .059 | .069 | .068 | .264 |
|  | [-.126, .069] | [-.119, .154] | [-.119, .001] | [-.010, .247] | [-.050, .213] | [-.040, .192] | [-.216, .056] | [-.265, .002] | [-.521, .513] |
|  | (.566) | (.802) | (.053) | (.071) | (.224) | (.199) | (.251) | (.054) | (.988) |
| Rural Code=6 | -.045 | .022 | -.032 | -.013 | .003 | -.038 | -.030 | -.009 | -.144 |
|  | .033 | .046 | .020 | .043 | .044 | .039 | .045 | .044 | .172 |
|  | [-.109, .019] | [-.067, .111] | [-.071, .007] | [-.097, .071] | [-.083, .089] | [-.114, .037] | [-.119, .059] | [-.096, .078] | [-.480, .193] |
|  | (.165) | (.628) | (.108) | (.757) | (.944) | (.321) | (.507) | (.834) | (.403) |
| Rural Code=7 | -.093 | .013 | -<.001 | .005 | -.080 | -.024 | -.035 | -.086 | -.302 |
|  | .045 | .062 | .027 | .059 | .060 | .053 | .062 | .061 | .235 |
|  | [-.180, -.006] | [-.109, .136] | [-.054, .053] | [-.110, .119] | [-.198, .037] | [-.128, .080] | [-.156, .087] | [-.205, .033] | [-.764, .159] |
|  | (.037) | (.830) | (.991) | (.938) | (.180) | (.652) | (.576) | (.159) | (.199) |
| Rural Code=8 | -.112 | -.009 | -.056 | -.155 | -.175 | -.103 | -.171 | -.176 | -.957 |
|  | .083 | .116 | .051 | .109 | .112 | .099 | .116 | .113 | .440 |
|  | [-.275, .052] | [-.238, .219] | [-.156, .043] | [-.369, .060] | [-.394, .045] | [-.297, .091] | [-.398, .057] | [-.398, .046] | [-1.819, -.094] |
|  | (.180) | (.935) | (.268) | (.157) | (.119) | (.297) | (.141) | (.121) | (.030) |
| Rural Code=9 | -.122 | -.082 | -.003 | -.034 | -.024 | -.005 | .053 | -.015 | -.231 |
|  | .071 | .099 | .043 | .093 | .095 | .084 | .098 | .096 | .373 |
|  | [-.260, .017] | [-.275, .112] | [-.088, .082] | [-.216, .148] | [-.210, .162] | [-.170, .159] | [-.140, .246] | [-.203, .174] | [-.963, .500] |
|  | (.086) | (.409) | (.940) | (.716) | (.799) | (.949) | (.592) | (.880) | (.535) |
| Alaska | .251 | .158 | -.090 | .301 | -.009 | .168 | -.031 | .205 | .953 |
|  | .141 | .198 | .087 | .185 | .190 | .168 | .197 | .192 | .747 |
|  | [-.026, .529] | [-.229, .545] | [-.260, .079] | [-.062, .665] | [-.381, .363] | [-.162, .497] | [-.416, .355] | [-.172, .582] | [-.510, 2.417] |
|  | (.076) | (.424) | (.297) | (.104) | (.962) | (.318) | (.876) | (.287) | (.202) |
| Arizona | .049 | -.185 | -.046 | -.018 | .017 | .022 | -.121 | .108 | -.175 |
|  | .067 | .094 | .041 | .089 | .090 | .080 | .094 | .092 | .356 |
|  | [-.083, .181] | [-.369, -<.001] | [-.127, .034] | [-.192, .156] | [-.160, .194] | [-.135, .178] | [-.305, .062] | [-.072, .288] | [-.872, .522] |
|  | (.466) | (.050) | (.260) | (.839) | (.851) | (.788) | (.195) | (.239) | (.623) |
| Arkansas | .113 | -.068 | -.042 | -.011 | .017 | .001 | -.048 | .087 | .048 |
|  | .076 | .106 | .046 | .099 | .102 | .090 | .105 | .103 | .399 |
|  | [-.035, .261] | [-.276, .139] | [-.133, .049] | [-.206, .183] | [-.182, .216] | [-.175, .177] | [-.254, .159] | [-.115, .289] | [-.734, .831] |
|  | (.135) | (.518) | (.367) | (.909) | (.866) | (.990) | (.651) | (.400) | (.903) |
| California | .073 | -.041 | -.047 | .072 | .061 | .113 | -.034 | .187 | .382 |
|  | .060 | .083 | .036 | .078 | .080 | .071 | .083 | .081 | .314 |
|  | [-.044, .189] | [-.205, .122] | [-.118, .025] | [-.082, .225] | [-.096, .217] | [-.025, .252] | [-.196, .128] | [.028, .346] | [-.234, .999] |
|  | (.221) | (.619) | (.200) | (.360) | (.449) | (.109) | (.681) | (.021) | (.224) |
| Colorado | .189 | .006 | -.064 | .181 | .156 | .169 | .055 | .158 | .848 |
|  | .076 | .106 | .047 | .100 | .102 | .090 | .106 | .104 | .402 |
|  | [.040, .338] | [-.202, .215] | [-.155, .027] | [-.015, .377] | [-.044, .356] | [-.007, .346] | [-.152, .262] | [-.045, .361] | [.061, 1.636] |
|  | (.013) | (.955) | (.171) | (.070) | (.127) | (.060) | (.603) | (.127) | (.035) |
| Connecticut | -.011 | -.037 | -.094 | .045 | .191 | .139 | -.025 | .261 | .469 |
|  | .083 | .116 | .051 | .109 | .111 | .098 | .115 | .113 | .437 |
|  | [-.173, .151] | [-.264, .190] | [-.194, .005] | [-.169, .258] | [-.027, .408] | [-.053, .332] | [-.251, .200] | [.039, .482] | [-.388, 1.327] |
|  | (.897) | (.748) | (.063) | (.682) | (.087) | (.156) | (.825) | (.021) | (.283) |
| Delaware | .174 | .209 | -.088 | -.028 | .345 | .136 | .077 | .271 | 1.091 |
|  | .107 | .150 | .065 | .141 | .143 | .127 | .148 | .146 | .565 |
|  | [-.035, .383] | [-.084, .502] | [-.217, .040] | [-.304, .248] | [.064, .626] | [-.112, .384] | [-.214, .368] | [-.015, .557] | [-.016, 2.197] |
|  | (.103) | (.163) | (.177) | (.843) | (.016) | (.283) | (.603) | (.064) | (.053) |
| District of Columbia | .052 | -.187 | -.107 | .331 | .183 | .194 | -.133 | .332 | .664 |
|  | .133 | .186 | .082 | .175 | .179 | .158 | .186 | .181 | .704 |
|  | [-.209, .313] | [-.552, .179] | [-.267, .053] | [-.011, .674] | [-.168, .534] | [-.116, .505] | [-.497, .231] | [-.024, .687] | [-.715, 2.044] |
|  | (.697) | (.317) | (.189) | (.058) | (.308) | (.220) | (.473) | (.068) | (.345) |
| Florida | .091 | .032 | -.051 | .008 | .091 | .085 | -.066 | .136 | .322 |
|  | .060 | .084 | .037 | .079 | .081 | .071 | .083 | .082 | .317 |
|  | [-.027, .208] | [-.133, .196] | [-.123, .021] | [-.147, .162] | [-.067, .249] | [-.054, .225] | [-.229, .098] | [-.024, .296] | [-.299, .942] |
|  | (.131) | (.706) | (.161) | (.921) | (.260) | (.230) | (.431) | (.096) | (.310) |
| Georgia | .056 | -.021 | -.053 | .150 | .086 | .121 | -.098 | .106 | .347 |
|  | .065 | .091 | .040 | .085 | .087 | .077 | .090 | .088 | .343 |
|  | [-.072, .183] | [-.199, .157] | [-.131, .025] | [-.017, .317] | [-.085, .257] | [-.030, .272] | [-.275, .079] | [-.067, .279] | [-.325, 1.019] |
|  | (.391) | (.817) | (.184) | (.078) | (.324) | (.117) | (.278) | (.230) | (.312) |
| Hawaii | .123 | .025 | -.003 | -.308 | -.082 | .206 | -.016 | .085 | .028 |
|  | .115 | .161 | .070 | .152 | .154 | .136 | .160 | .157 | .607 |
|  | [-.102, .348] | [-.291, .340] | [-.141, .136] | [-.605, -.011] | [-.384, .221] | [-.061, .473] | [-.329, .297] | [-.223, .392] | [-1.162, 1.218] |
|  | (.284) | (.878) | (.971) | (.042) | (.597) | (.131) | (.919) | (.590) | (.963) |
| Idaho | -.114 | -.092 | -.085 | .090 | -.093 | .004 | -.293 | -.037 | -.621 |
|  | .096 | .135 | .059 | .127 | .129 | .114 | .134 | .132 | .509 |
|  | [-.302, .075] | [-.356, .172] | [-.201, .030] | [-.159, .339] | [-.346, .161] | [-.220, .228] | [-.555, -.031] | [-.294, .221] | [-1.618, .376] |
|  | (.236) | (.495) | (.148) | (.478) | (.473) | (.974) | (.029) | (.781) | (.222) |
| Illinois | -.003 | -.190 | -.058 | .124 | .019 | .043 | -.052 | .202 | .086 |
|  | .066 | .092 | .040 | .087 | .089 | .078 | .092 | .090 | .349 |
|  | [-.132, .127] | [-.371, -.009] | [-.138, .021] | [-.046, .294] | [-.155, .193] | [-.111, .196] | [-.232, .128] | [.026, .378] | [-.598, .770] |
|  | (.969) | (.040) | (.149) | (.153) | (.826) | (.586) | (.571) | (.025) | (.805) |
| Indiana | .037 | -.127 | -.053 | .097 | .117 | .176 | -.138 | .184 | .293 |
|  | .071 | .099 | .043 | .093 | .095 | .084 | .098 | .096 | .373 |
|  | [-.102, .176] | [-.321, .067] | [-.137, .032] | [-.085, .279] | [-.070, .303] | [.011, .340] | [-.330, .055] | [-.005, .372] | [-.439, 1.025] |
|  | (.600) | (.199) | (.225) | (.298) | (.219) | (.036) | (.162) | (.056) | (.432) |
| Iowa | .174 | .006 | -.010 | .030 | -.062 | .104 | -.051 | -.054 | .137 |
|  | .084 | .117 | .051 | .110 | .113 | .100 | .117 | .114 | .444 |
|  | [.010, .339] | [-.224, .236] | [-.110, .091] | [-.186, .246] | [-.284, .159] | [-.092, .299] | [-.280, .178] | [-.278, .171] | [-.733, 1.007] |
|  | (.038) | (.959) | (.852) | (.788) | (.580) | (.299) | (.662) | (.640) | (.757) |
| Kansas | .195 | -.161 | -.099 | -.012 | .083 | .181 | -.065 | .099 | .218 |
|  | .102 | .142 | .062 | .134 | .137 | .121 | .141 | .139 | .537 |
|  | [-.005, .394] | [-.440, .117] | [-.221, .023] | [-.274, .249] | [-.185, .350] | [-.056, .417] | [-.343, .212] | [-.172, .371] | [-.834, 1.271] |
|  | (.056) | (.256) | (.113) | (.926) | (.545) | (.135) | (.644) | (.475) | (.684) |
| Kentucky | .139 | -.113 | -.047 | -.161 | .130 | .067 | -.052 | .003 | -.033 |
|  | .077 | .108 | .047 | .101 | .103 | .091 | .107 | .105 | .406 |
|  | [-.012, .290] | [-.323, .098] | [-.140, .045] | [-.359, .037] | [-.073, .332] | [-.112, .246] | [-.262, .157] | [-.203, .208] | [-.829, .763] |
|  | (.070) | (.295) | (.316) | (.111) | (.209) | (.463) | (.624) | (.979) | (.936) |
| Louisiana | .104 | -.102 | -.078 | -.080 | .145 | .164 | -.037 | .107 | .224 |
|  | .077 | .108 | .047 | .102 | .104 | .092 | .107 | .105 | .408 |
|  | [-.047, .256] | [-.313, .110] | [-.171, .014] | [-.279, .119] | [-.058, .348] | [-.016, .343] | [-.247, .173] | [-.100, .313] | [-.575, 1.023] |
|  | (.176) | (.346) | (.097) | (.432) | (.161) | (.074) | (.729) | (.311) | (.583) |
| Maine | .121 | -.223 | -.083 | .171 | .233 | .225 | .274 | .231 | .949 |
|  | .107 | .150 | .066 | .141 | .144 | .127 | .149 | .146 | .565 |
|  | [-.088, .330] | [-.516, .071] | [-.211, .045] | [-.106, .447] | [-.048, .515] | [-.024, .474] | [-.018, .565] | [-.055, .517] | [-.158, 2.056] |
|  | (.258) | (.137) | (.205) | (.226) | (.104) | (.076) | (.066) | (.113) | (.093) |
| Maryland | .121 | -.026 | -.077 | .007 | .092 | .174 | -.032 | -.015 | .244 |
|  | .076 | .107 | .047 | .100 | .103 | .091 | .106 | .104 | .404 |
|  | [-.028, .271] | [-.236, .183] | [-.169, .015] | [-.190, .204] | [-.109, .293] | [-.003, .352] | [-.241, .176] | [-.219, .189] | [-.547, 1.035] |
|  | (.112) | (.806) | (.100) | (.945) | (.371) | (.055) | (.761) | (.886) | (.546) |
| Massachusetts | .061 | -.115 | -.084 | .114 | .159 | .178 | -.172 | .178 | .319 |
|  | .072 | .101 | .044 | .095 | .097 | .085 | .100 | .098 | .380 |
|  | [-.080, .202] | [-.312, .083] | [-.170, .003] | [-.071, .300] | [-.030, .349] | [.011, .346] | [-.368, .025] | [-.014, .371] | [-.426, 1.065] |
|  | (.400) | (.256) | (.058) | (.227) | (.099) | (.037) | (.086) | (.069) | (.401) |
| Michigan | .114 | -.062 | -.026 | -.049 | .107 | .093 | -.109 | .088 | .156 |
|  | .064 | .090 | .039 | .084 | .086 | .076 | .089 | .087 | .338 |
|  | [-.011, .240] | [-.238, .114] | [-.103, .051] | [-.214, .116] | [-.061, .276] | [-.056, .243] | [-.283, .066] | [-.083, .259] | [-.507, .820] |
|  | (.075) | (.489) | (.503) | (.561) | (.212) | (.220) | (.223) | (.312) | (.644) |
| Minnesota | .122 | -.114 | -.073 | .074 | .105 | .108 | -.039 | .136 | .318 |
|  | .076 | .106 | .046 | .099 | .101 | .090 | .105 | .103 | .399 |
|  | [-.026, .270] | [-.321, .093] | [-.163, .018] | [-.121, .268] | [-.094, .304] | [-.068, .284] | [-.245, .167] | [-.066, .337] | [-.464, 1.100] |
|  | (.106) | (.281) | (.117) | (.457) | (.301) | (.228) | (.709) | (.188) | (.425) |
| Mississippi | .085 | -.078 | -.024 | -.105 | .138 | .074 | .045 | .241 | .376 |
|  | .086 | .120 | .052 | .112 | .115 | .102 | .119 | .116 | .451 |
|  | [-.083, .252] | [-.313, .156] | [-.127, .079] | [-.325, .115] | [-.087, .363] | [-.125, .273] | [-.188, .278] | [.013, .469] | [-.509, 1.261] |
|  | (.321) | (.512) | (.646) | (.351) | (.230) | (.467) | (.705) | (.038) | (.405) |
| Missouri | .017 | -.127 | -.040 | -.001 | .033 | .152 | -.090 | .042 | -.015 |
|  | .069 | .097 | .042 | .091 | .093 | .082 | .096 | .094 | .366 |
|  | [-.119, .152] | [-.317, .063] | [-.123, .043] | [-.180, .177] | [-.149, .215] | [-.009, .313] | [-.279, .099] | [-.143, .227] | [-.732, .702] |
|  | (.811) | (.190) | (.343) | (.989) | (.722) | (.064) | (.349) | (.657) | (.967) |
| Montana | .147 | -.084 | -.064 | .086 | .070 | .128 | -.180 | .154 | .258 |
|  | .107 | .149 | .065 | .141 | .143 | .127 | .148 | .146 | .564 |
|  | [-.062, .356] | [-.376, .209] | [-.192, .065] | [-.190, .361] | [-.211, .351] | [-.120, .376] | [-.471, .111] | [-.131, .439] | [-.847, 1.363] |
|  | (.169) | (.576) | (.330) | (.541) | (.626) | (.312) | (.224) | (.290) | (.647) |
| Nebraska | .095 | -.036 | -.090 | -.005 | .112 | .122 | -.027 | .026 | .197 |
|  | .110 | .154 | .067 | .144 | .148 | .131 | .153 | .150 | .580 |
|  | [-.120, .311] | [-.337, .265] | [-.221, .042] | [-.288, .277] | [-.178, .401] | [-.134, .378] | [-.327, .273] | [-.267, .319] | [-.940, 1.335] |
|  | (.386) | (.815) | (.183) | (.972) | (.449) | (.350) | (.861) | (.862) | (.734) |
| Nevada | .065 | -.164 | -.053 | -.044 | .214 | .140 | -.040 | .246 | .366 |
|  | .075 | .105 | .046 | .099 | .101 | .089 | .105 | .103 | .398 |
|  | [-.082, .213] | [-.371, .043] | [-.143, .038] | [-.238, .151] | [.016, .413] | [-.035, .315] | [-.245, .165] | [.044, .447] | [-.414, 1.147] |
|  | (.386) | (.120) | (.254) | (.661) | (.034) | (.118) | (.703) | (.017) | (.357) |
| New Hampshire | .147 | -.192 | -.037 | -.082 | .025 | .070 | -.047 | -.005 | -.122 |
|  | .090 | .127 | .055 | .119 | .121 | .107 | .126 | .123 | .478 |
|  | [-.030, .324] | [-.440, .056] | [-.145, .072] | [-.315, .152] | [-.213, .262] | [-.140, .281] | [-.293, .199] | [-.247, .237] | [-1.058, .814] |
|  | (.104) | (.130) | (.506) | (.492) | (.839) | (.512) | (.708) | (.968) | (.798) |
| New Jersey | .115 | .026 | -.043 | .063 | .126 | .142 | -.039 | .061 | .451 |
|  | .070 | .098 | .043 | .092 | .094 | .083 | .097 | .095 | .369 |
|  | [-.022, .252] | [-.165, .217] | [-.127, .041] | [-.116, .243] | [-.058, .310] | [-.020, .305] | [-.230, .151] | [-.125, .247] | [-.272, 1.174] |
|  | (.099) | (.789) | (.314) | (.490) | (.179) | (.086) | (.685) | (.522) | (.221) |
| New Mexico | .112 | -.124 | -.056 | .049 | .124 | -.047 | .063 | .141 | .257 |
|  | .083 | .117 | .051 | .110 | .112 | .099 | .116 | .114 | .440 |
|  | [-.051, .275] | [-.352, .104] | [-.156, .043] | [-.166, .264] | [-.095, .343] | [-.241, .146] | [-.164, .290] | [-.082, .363] | [-.605, 1.119] |
|  | (.179) | (.287) | (.268) | (.655) | (.268) | (.632) | (.586) | (.215) | (.559) |
| New York | .018 | .017 | -.030 | .055 | .090 | .144 | -.031 | .106 | .371 |
|  | .061 | .086 | .037 | .080 | .082 | .073 | .085 | .083 | .323 |
|  | [-.102, .139] | [-.151, .185] | [-.104, .043] | [-.103, .213] | [-.071, .251] | [.001, .286] | [-.198, .136] | [-.057, .270] | [-.263, 1.004] |
|  | (.763) | (.844) | (.421) | (.495) | (.273) | (.048) | (.714) | (.202) | (.252) |
| North Carolina | .097 | .018 | -.035 | .056 | .107 | .136 | .007 | .058 | .442 |
|  | .066 | .092 | .040 | .087 | .089 | .078 | .092 | .090 | .348 |
|  | [-.032, .226] | [-.163, .199] | [-.114, .044] | [-.114, .226] | [-.067, .280] | [-.017, .289] | [-.173, .187] | [-.118, .234] | [-.241, 1.124] |
|  | (.141) | (.844) | (.386) | (.520) | (.228) | (.082) | (.941) | (.519) | (.205) |
| North Dakota | .093 | .321 | -.084 | -.049 | .010 | .211 | -.012 | .152 | .640 |
|  | .134 | .188 | .082 | .176 | .180 | .159 | .186 | .183 | .708 |
|  | [-.170, .355] | [-.046, .689] | [-.245, .077] | [-.395, .297] | [-.342, .362] | [-.101, .522] | [-.377, .353] | [-.206, .510] | [-.747, 2.027] |
|  | (.488) | (.087) | (.304) | (.780) | (.956) | (.185) | (.947) | (.405) | (.366) |
| Ohio | .107 | -.037 | -.046 | .050 | .095 | .154 | -.011 | .150 | .462 |
|  | .064 | .090 | .039 | .084 | .086 | .076 | .089 | .087 | .339 |
|  | [-.019, .232] | [-.213, .138] | [-.122, .031] | [-.115, .215] | [-.074, .264] | [.005, .303] | [-.186, .164] | [-.021, .321] | [-.202, 1.126] |
|  | (.096) | (.678) | (.246) | (.555) | (.271) | (.043) | (.903) | (.086) | (.173) |
| Oklahoma | .045 | .083 | .020 | .081 | .146 | .108 | -.078 | .115 | .519 |
|  | .086 | .120 | .053 | .113 | .115 | .102 | .120 | .117 | .454 |
|  | [-.123, .214] | [-.153, .318] | [-.083, .123] | [-.140, .303] | [-.080, .372] | [-.092, .308] | [-.313, .156] | [-.114, .344] | [-.370, 1.409] |
|  | (.599) | (.492) | (.707) | (.471) | (.206) | (.290) | (.513) | (.325) | (.253) |
| Oregon | .019 | -.125 | -.072 | .063 | .068 | .125 | -.044 | .150 | .185 |
|  | .071 | .099 | .043 | .094 | .095 | .084 | .099 | .097 | .375 |
|  | [-.120, .158] | [-.320, .070] | [-.157, .013] | [-.120, .246] | [-.119, .254] | [-.040, .290] | [-.237, .149] | [-.040, .340] | [-.550, .920] |
|  | (.787) | (.209) | (.099) | (.501) | (.478) | (.138) | (.656) | (.121) | (.622) |
| Pennsylvania | .110 | -.119 | -.056 | .037 | .100 | .154 | -.078 | .110 | .257 |
|  | .061 | .085 | .037 | .080 | .082 | .072 | .085 | .083 | .321 |
|  | [-.009, .229] | [-.285, .048] | [-.129, .017] | [-.120, .193] | [-.060, .260] | [.012, .295] | [-.244, .088] | [-.052, .272] | [-.372, .886] |
|  | (.069) | (.163) | (.134) | (.646) | (.220) | (.033) | (.357) | (.184) | (.423) |
| Rhode Island | .100 | .303 | .034 | .152 | .196 | .105 | -.094 | .134 | .929 |
|  | .133 | .186 | .081 | .175 | .179 | .158 | .185 | .181 | .703 |
|  | [-.161, .361] | [-.062, .667] | [-.126, .194] | [-.190, .494] | [-.155, .546] | [-.205, .415] | [-.457, .269] | [-.221, .488] | [-.449, 2.306] |
|  | (.453) | (.103) | (.677) | (.385) | (.274) | (.506) | (.611) | (.461) | (.186) |
| South Carolina | .099 | .007 | -.073 | .041 | .187 | .195 | .035 | .142 | .631 |
|  | .074 | .104 | .046 | .098 | .100 | .088 | .103 | .102 | .393 |
|  | [-.047, .245] | [-.197, .211] | [-.162, .016] | [-.151, .233] | [-.009, .383] | [.022, .368] | [-.168, .238] | [-.057, .341] | [-.140, 1.401] |
|  | (.182) | (.944) | (.109) | (.673) | (.061) | (.027) | (.737) | (.163) | (.109) |
| South Dakota | -.023 | -.319 | -.089 | -.104 | -.139 | .092 | -.196 | -.182 | -.961 |
|  | .123 | .172 | .075 | .162 | .165 | .146 | .171 | .168 | .651 |
|  | [-.265, .218] | [-.657, .019] | [-.237, .059] | [-.421, .214] | [-.463, .185] | [-.195, .378] | [-.532, .139] | [-.511, .147] | [-2.236, .315] |
|  | (.849) | (.064) | (.237) | (.523) | (.401) | (.531) | (.252) | (.278) | (.140) |
| Tennessee | .157 | .091 | -.047 | .045 | .099 | .226 | .074 | .136 | .779 |
|  | .073 | .102 | .045 | .096 | .098 | .086 | .101 | .099 | .384 |
|  | [.014, .299] | [-.109, .290] | [-.134, .040] | [-.143, .232] | [-.093, .290] | [.057, .396] | [-.124, .273] | [-.059, .330] | [.026, 1.533] |
|  | (.031) | (.373) | (.292) | (.640) | (.313) | (.009) | (.462) | (.171) | (.042) |
| Texas | .054 | -.013 | -.035 | .038 | .015 | .047 | -.142 | .008 | -.028 |
|  | .061 | .085 | .037 | .080 | .082 | .072 | .085 | .083 | .321 |
|  | [-.065, .173] | [-.179, .154] | [-.108, .038] | [-.119, .194] | [-.145, .175] | [-.094, .189] | [-.308, .023] | [-.154, .170] | [-.657, .601] |
|  | (.373) | (.881) | (.344) | (.638) | (.854) | (.510) | (.092) | (.921) | (.930) |
| Utah | .064 | -.144 | -.076 | .151 | .036 | .114 | -.036 | .114 | .222 |
|  | .084 | .118 | .052 | .111 | .113 | .100 | .117 | .115 | .445 |
|  | [-.101, .229] | [-.376, .087] | [-.177, .025] | [-.066, .369] | [-.186, .257] | [-.082, .310] | [-.266, .194] | [-.111, .340] | [-.651, 1.094] |
|  | (.444) | (.222) | (.142) | (.173) | (.753) | (.253) | (.758) | (.320) | (.619) |
| Vermont | .235 | -.140 | -.081 | .219 | .179 | .008 | .056 | -.105 | .373 |
|  | .122 | .170 | .075 | .160 | .164 | .145 | .170 | .166 | .643 |
|  | [-.004, .474] | [-.474, .193] | [-.227, .065] | [-.094, .532] | [-.142, .500] | [-.275, .292] | [-.276, .389] | [-.429, .220] | [-.888, 1.633] |
|  | (.054) | (.409) | (.277) | (.170) | (.274) | (.956) | (.739) | (.528) | (.562) |
| Virginia | .075 | .035 | -.049 | .041 | .050 | .150 | -.034 | .152 | .419 |
|  | .065 | .091 | .040 | .086 | .087 | .077 | .091 | .089 | .344 |
|  | [-.052, .203] | [-.144, .213] | [-.127, .029] | [-.127, .209] | [-.121, .221] | [-.001, .302] | [-.212, .144] | [-.023, .326] | [-.255, 1.094] |
|  | (.247) | (.705) | (.219) | (.635) | (.568) | (.052) | (.707) | (.088) | (.223) |
| Washington | .114 | -.085 | -.016 | .064 | .149 | .184 | -.027 | .113 | .494 |
|  | .068 | .096 | .042 | .090 | .092 | .081 | .095 | .093 | .361 |
|  | [-.020, .247] | [-.273, .102] | [-.098, .066] | [-.112, .240] | [-.031, .328] | [.025, .343] | [-.213, .159] | [-.070, .296] | [-.213, 1.202] |
|  | (.096) | (.373) | (.710) | (.476) | (.105) | (.023) | (.778) | (.225) | (.171) |
| West Virginia | -.010 | -.039 | -.036 | .059 | -.035 | .038 | -.148 | -.012 | -.184 |
|  | .087 | .122 | .053 | .114 | .117 | .103 | .121 | .118 | .459 |
|  | [-.181, .160] | [-.278, .199] | [-.140, .069] | [-.165, .283] | [-.263, .194] | [-.164, .241] | [-.385, .089] | [-.244, .220] | [-1.084, .716] |
|  | (.904) | (.745) | (.502) | (.605) | (.768) | (.709) | (.220) | (.917) | (.689) |
| Wisconsin | .072 | -.078 | -.031 | .096 | .082 | .135 | -.104 | .033 | .203 |
|  | .071 | .099 | .043 | .093 | .095 | .084 | .098 | .096 | .373 |
|  | [-.066, .210] | [-.272, .115] | [-.116, .053] | [-.086, .278] | [-.104, .268] | [-.030, .299] | [-.297, .089] | [-.156, .221] | [-.528, .935] |
|  | (.308) | (.429) | (.468) | (.303) | (.388) | (.108) | (.290) | (.733) | (.585) |
| Wyoming | .271 | .248 | .238 | .036 | .144 | .067 | .158 | .488 | 1.656 |
|  | .205 | .289 | .126 | .273 | .276 | .244 | .285 | .283 | 1.088 |
|  | [-.131, .673] | [-.319, .814] | [-.010, .486] | [-.500, .572] | [-.396, .684] | [-.410, .544] | [-.401, .717] | [-.066, 1.042] | [-.476, 3.788] |
|  | (.187) | (.392) | (.059) | (.895) | (.601) | (.784) | (.579) | (.084) | (.128) |
| N | 2982 | 2982 | 2982 | 2982 | 2982 | 2982 | 2982 | 2982 | 2982 |
| Pseudo-R^2^ |  |  |  |  |  |  |  |  |  |
| Model χ^2^ | 211.2 | 201.9 | 114.7 | 32.9 | 214.3 | 227.5 | 313.5 | 214.0 | 383.8 |
| d.f. | 88 | 88 | 88 | 88 | 88 | 88 | 88 | 88 | 88 |
| p-value | <.001 | <.001 | <.001 | <.001 | <.001 | <.001 | <.001 | <.001 | <.001 |

**S5 Fig. Health Behaviors, Multilevel Models.** This figure compares key coefficients of interest from S7-S9 Tables.

S10 Table. Regression Results, Health Attitudes, Multilevel Models.

|  | (1) | (2) | (3) | (4) | (5) | (6) | (7) | (8) |
| --- | --- | --- | --- | --- | --- | --- | --- | --- |
|  | numberdeaths | spendingincrease | notenoughtests | getsick | friendsick | cannotreturn | schoolsclosed | hardgetitems |
| main |  |  |  |  |  |  |  |  |
| Democrat | .358 | .331 | .866 | .545 | .512 | .439 | .271 | .309 |
|  | .053 | .024 | .053 | .048 | .047 | .049 | .052 | .048 |
|  | [.254, .461] | [.284, .379] | [.763, .970] | [.450, .641] | [.420, .604] | [.344, .535] | [.169, .372] | [.215, .403] |
|  | (<.001) | (<.001) | (<.001) | (<.001) | (<.001) | (<.001) | (<.001) | (<.001) |
| Other | .098 | .099 | .506 | .190 | .130 | .094 | .064 | .056 |
|  | .054 | .025 | .054 | .049 | .047 | .049 | .052 | .049 |
|  | [-.007, .203] | [.051, .147] | [.401, .611] | [.094, .287] | [.037, .223] | [-.002, .191] | [-.039, .166] | [-.039, .151] |
|  | (.066) | (<.001) | (<.001) | (<.001) | (.006) | (.056) | (.226) | (.250) |
| 35- | -.037 | .041 | .071 | .062 | .068 | -.138 | .037 | .013 |
|  | .059 | .027 | .059 | .054 | .053 | .055 | .058 | .054 |
|  | [-.153, .080] | [-.013, .094] | [-.046, .187] | [-.045, .168] | [-.035, .171] | [-.245, -.031] | [-.077, .151] | [-.093, .118] |
|  | (.537) | (.134) | (.234) | (.257) | (.195) | (.012) | (.521) | (.813) |
| 50- | .154 | .031 | .136 | -.051 | -.017 | -.216 | -.103 | -.191 |
|  | .056 | .026 | .056 | .051 | .050 | .052 | .055 | .051 |
|  | [.044, .264] | [-.020, .081] | [.026, .246] | [-.151, .050] | [-.115, .080] | [-.317, -.115] | [-.210, .005] | [-.291, -.092] |
|  | (.006) | (.235) | (.015) | (.325) | (.729) | (<.001) | (.062) | (<.001) |
| 70- | .336 | .075 | .127 | -.004 | -.009 | -.286 | -.543 | -.408 |
|  | .071 | .033 | .071 | .065 | .063 | .065 | .069 | .064 |
|  | [.197, .475] | [.012, .139] | [-.012, .265] | [-.131, .124] | [-.132, .114] | [-.414, -.158] | [-.679, -.407] | [-.534, -.282] |
|  | (<.001) | (.020) | (.074) | (.953) | (.886) | (<.001) | (<.001) | (<.001) |
| gender | .047 | -.026 | .010 | .145 | .135 | .029 | .047 | .144 |
|  | .041 | .019 | .041 | .037 | .036 | .038 | .040 | .037 |
|  | [-.033, .127] | [-.063, .010] | [-.070, .090] | [.072, .218] | [.064, .206] | [-.045, .103] | [-.032, .125] | [.072, .217] |
|  | (.247) | (.160) | (.802) | (<.001) | (<.001) | (.441) | (.243) | (<.001) |
| white | -.040 | .069 | .245 | .030 | .006 | .134 | -.076 | .027 |
|  | .049 | .022 | .049 | .045 | .043 | .045 | .048 | .044 |
|  | [-.136, .056] | [.025, .113] | [.150, .341] | [-.058, .117] | [-.079, .091] | [.046, .222] | [-.170, .017] | [-.060, .113] |
|  | (.413) | (.002) | (<.001) | (.505) | (.889) | (.003) | (.111) | (.547) |
| married | .006 | -.015 | -.120 | .102 | .082 | .021 | .068 | .101 |
|  | .045 | .021 | .045 | .041 | .040 | .041 | .044 | .041 |
|  | [-.082, .094] | [-.055, .025] | [-.207, -.032] | [.021, .182] | [.004, .160] | [-.060, .102] | [-.018, .154] | [.022, .181] |
|  | (.893) | (.467) | (.008) | (.014) | (.040) | (.609) | (.121) | (.013) |
| High school graduate | -.040 | -.057 | -.012 | -.067 | -.120 | -.074 | .014 | -.218 |
|  | .120 | .055 | .120 | .110 | .106 | .110 | .117 | .108 |
|  | [-.275, .194] | [-.165, .050] | [-.247, .224] | [-.282, .148] | [-.327, .088] | [-.290, .141] | [-.215, .244] | [-.431, -.006] |
|  | (.737) | (.296) | (.921) | (.543) | (.259) | (.499) | (.903) | (.044) |
| Some college | -.076 | .005 | .235 | -.021 | -.102 | -.070 | -.019 | -.317 |
|  | .122 | .056 | .122 | .112 | .108 | .112 | .119 | .110 |
|  | [-.315, .163] | [-.105, .115] | [-.005, .475] | [-.240, .199] | [-.314, .110] | [-.290, .150] | [-.253, .215] | [-.533, -.100] |
|  | (.534) | (.928) | (.055) | (.854) | (.345) | (.534) | (.875) | (.004) |
| 2-year | -.010 | .019 | .157 | -.015 | -.089 | -.106 | -.025 | -.350 |
|  | .128 | .059 | .129 | .118 | .114 | .118 | .126 | .116 |
|  | [-.261, .242] | [-.096, .135] | [-.095, .409] | [-.246, .215] | [-.312, .133] | [-.337, .125] | [-.271, .221] | [-.578, -.123] |
|  | (.940) | (.740) | (.222) | (.895) | (.432) | (.368) | (.841) | (.003) |
| 4-year | -.057 | .027 | .154 | -.053 | -.062 | -.046 | -.059 | -.316 |
|  | .125 | .057 | .125 | .114 | .110 | .114 | .122 | .113 |
|  | [-.301, .187] | [-.085, .138] | [-.090, .399] | [-.277, .170] | [-.278, .154] | [-.270, .178] | [-.298, .180] | [-.537, -.095] |
|  | (.647) | (.642) | (.216) | (.640) | (.576) | (.687) | (.627) | (.005) |
| Post-grad | -.054 | .046 | .209 | .050 | .008 | -.003 | .003 | -.412 |
|  | .131 | .060 | .131 | .120 | .116 | .120 | .128 | .118 |
|  | [-.310, .203] | [-.071, .163] | [-.048, .466] | [-.185, .286] | [-.219, .235] | [-.238, .233] | [-.248, .254] | [-.644, -.180] |
|  | (.682) | (.443) | (.112) | (.674) | (.947) | (.982) | (.980) | (<.001) |
| income=2 | .064 | -.041 | .362 | -.074 | -.001 | -.161 | -.154 | -.115 |
|  | .115 | .053 | .116 | .106 | .102 | .106 | .113 | .104 |
|  | [-.163, .290] | [-.144, .063] | [.135, .589] | [-.281, .134] | [-.201, .199] | [-.369, .047] | [-.376, .067] | [-.320, .090] |
|  | (.582) | (.443) | (.002) | (.486) | (.994) | (.129) | (.173) | (.270) |
| income=3 | .171 | -.005 | .306 | -.094 | -.013 | -.112 | -.042 | -.149 |
|  | .112 | .051 | .113 | .103 | .099 | .103 | .109 | .101 |
|  | [-.048, .390] | [-.105, .096] | [.086, .527] | [-.295, .107] | [-.207, .181] | [-.313, .090] | [-.257, .172] | [-.347, .049] |
|  | (.126) | (.926) | (.007) | (.358) | (.898) | (.278) | (.700) | (.141) |
| income=4 | .121 | .025 | .290 | .010 | .090 | -.087 | -.047 | -.122 |
|  | .114 | .052 | .115 | .105 | .101 | .105 | .112 | .104 |
|  | [-.103, .345] | [-.078, .127] | [.065, .516] | [-.196, .216] | [-.109, .288] | [-.293, .120] | [-.267, .173] | [-.325, .081] |
|  | (.290) | (.638) | (.012) | (.923) | (.376) | (.411) | (.675) | (.238) |
| income=5 | .179 | .028 | .366 | -.061 | .007 | -.161 | -.035 | -.201 |
|  | .117 | .054 | .118 | .107 | .103 | .107 | .114 | .106 |
|  | [-.050, .408] | [-.077, .133] | [.136, .597] | [-.271, .149] | [-.196, .210] | [-.372, .049] | [-.259, .189] | [-.408, .006] |
|  | (.126) | (.602) | (.002) | (.571) | (.945) | (.133) | (.759) | (.058) |
| income=6 | .216 | -.004 | .366 | .011 | .006 | -.092 | .087 | -.150 |
|  | .118 | .054 | .119 | .108 | .105 | .109 | .116 | .107 |
|  | [-.016, .447] | [-.110, .102] | [.134, .599] | [-.201, .224] | [-.199, .211] | [-.305, .121] | [-.140, .314] | [-.359, .060] |
|  | (.068) | (.938) | (.002) | (.916) | (.956) | (.398) | (.452) | (.161) |
| income=7 | .080 | .001 | .395 | -.077 | -.022 | -.183 | -.099 | -.288 |
|  | .127 | .058 | .127 | .116 | .112 | .116 | .124 | .114 |
|  | [-.168, .328] | [-.112, .115] | [.146, .645] | [-.304, .150] | [-.241, .197] | [-.411, .045] | [-.341, .144] | [-.512, -.063] |
|  | (.525) | (.980) | (.002) | (.506) | (.845) | (.116) | (.426) | (.012) |
| income=8 | .116 | .010 | .332 | -.278 | -.231 | -.269 | -.180 | -.374 |
|  | .123 | .056 | .124 | .113 | .109 | .113 | .121 | .111 |
|  | [-.126, .357] | [-.101, .120] | [.089, .575] | [-.499, -.056] | [-.445, -.017] | [-.491, -.047] | [-.416, .056] | [-.593, -.156] |
|  | (.348) | (.863) | (.007) | (.014) | (.034) | (.017) | (.136) | (.001) |
| income=9 | .350 | .009 | .464 | -.187 | -.067 | -.081 | -.108 | -.309 |
|  | .122 | .056 | .122 | .112 | .108 | .112 | .119 | .110 |
|  | [.111, .589] | [-.100, .118] | [.224, .704] | [-.406, .032] | [-.279, .144] | [-.301, .139] | [-.342, .126] | [-.525, -.093] |
|  | (.004) | (.873) | (<.001) | (.094) | (.532) | (.470) | (.365) | (.005) |
| income=10 | .221 | .004 | .489 | -.180 | -.078 | -.101 | -.108 | -.375 |
|  | .127 | .058 | .127 | .116 | .112 | .116 | .124 | .115 |
|  | [-.028, .469] | [-.109, .118] | [.240, .738] | [-.407, .048] | [-.298, .141] | [-.329, .127] | [-.352, .135] | [-.600, -.150] |
|  | (.082) | (.938) | (<.001) | (.122) | (.484) | (.385) | (.382) | (.001) |
| income=11 | .369 | .001 | .467 | -.076 | -.002 | .073 | -.099 | -.355 |
|  | .130 | .060 | .131 | .119 | .115 | .120 | .127 | .118 |
|  | [.114, .624] | [-.116, .118] | [.212, .723] | [-.310, .158] | [-.228, .223] | [-.161, .307] | [-.349, .151] | [-.586, -.124] |
|  | (.005) | (.988) | (<.001) | (.526) | (.983) | (.542) | (.437) | (.003) |
| income=12 | .234 | .034 | .364 | -.140 | .002 | .022 | -.183 | -.420 |
|  | .143 | .066 | .143 | .131 | .127 | .132 | .140 | .130 |
|  | [-.046, .514] | [-.095, .163] | [.083, .645] | [-.397, .117] | [-.246, .250] | [-.236, .280] | [-.457, .092] | [-.674, -.166] |
|  | (.102) | (.605) | (.011) | (.287) | (.988) | (.865) | (.192) | (.001) |
| income=13 | .212 | .063 | .643 | -.260 | -.134 | -.083 | -.420 | -.649 |
|  | .198 | .091 | .198 | .181 | .175 | .182 | .194 | .179 |
|  | [-.176, .600] | [-.115, .241] | [.255, 1.031] | [-.616, .095] | [-.478, .210] | [-.440, .273] | [-.799, -.040] | [-1.000, -.298] |
|  | (.284) | (.489) | (.001) | (.152) | (.445) | (.647) | (.030) | (<.001) |
| income=14 | .305 | -.078 | .518 | -.235 | -.077 | -.191 | -.071 | -.442 |
|  | .210 | .096 | .209 | .192 | .186 | .193 | .205 | .190 |
|  | [-.106, .716] | [-.266, .111] | [.107, .928] | [-.611, .142] | [-.441, .287] | [-.569, .187] | [-.474, .331] | [-.814, -.071] |
|  | (.145) | (.419) | (.013) | (.222) | (.679) | (.321) | (.728) | (.020) |
| income=15 | .269 | .234 | 1.394 | .429 | .221 | .369 | -.069 | -1.156 |
|  | .635 | .290 | .633 | .582 | .562 | .584 | .622 | .574 |
|  | [-.975, 1.514] | [-.335, .803] | [.154, 2.635] | [-.711, 1.570] | [-.880, 1.322] | [-.775, 1.513] | [-1.287, 1.149] | [-2.282, -.031] |
|  | (.672) | (.420) | (.028) | (.461) | (.694) | (.527) | (.911) | (.044) |
| income=16 | .529 | .045 | .868 | -.302 | -.095 | .167 | -.037 | -.489 |
|  | .298 | .137 | .298 | .274 | .264 | .274 | .292 | .270 |
|  | [-.056, 1.114] | [-.223, .312] | [.284, 1.451] | [-.838, .234] | [-.613, .423] | [-.370, .705] | [-.610, .535] | [-1.018, .040] |
|  | (.076) | (.743) | (.004) | (.269) | (.719) | (.542) | (.899) | (.070) |
| income=17 | .271 | -.033 | .342 | -.236 | -.133 | -.229 | -.263 | -.422 |
|  | .108 | .050 | .109 | .099 | .096 | .100 | .106 | .098 |
|  | [.059, .484] | [-.130, .065] | [.128, .556] | [-.430, -.041] | [-.321, .055] | [-.424, -.034] | [-.471, -.055] | [-.614, -.229] |
|  | (.012) | (.509) | (.002) | (.018) | (.167) | (.022) | (.013) | (<.001) |
| Rural Code=2 | .006 | -.016 | -.053 | -.006 | -.063 | -.040 | -.100 | -.013 |
|  | .054 | .025 | .054 | .050 | .048 | .050 | .053 | .049 |
|  | [-.100, .112] | [-.065, .033] | [-.159, .054] | [-.103, .091] | [-.158, .031] | [-.138, .057] | [-.203, .004] | [-.110, .083] |
|  | (.915) | (.524) | (.332) | (.907) | (.189) | (.417) | (.060) | (.784) |
| Rural Code=3 | -.009 | -.028 | -.105 | .021 | .048 | .018 | .022 | .051 |
|  | .074 | .034 | .074 | .067 | .066 | .068 | .072 | .067 |
|  | [-.153, .135] | [-.095, .039] | [-.249, .040] | [-.112, .153] | [-.080, .177] | [-.115, .150] | [-.119, .163] | [-.080, .183] |
|  | (.903) | (.407) | (.157) | (.760) | (.464) | (.794) | (.761) | (.444) |
| Rural Code=4 | -.083 | .013 | .067 | -.139 | -.176 | -.141 | -.094 | -.048 |
|  | .103 | .047 | .103 | .094 | .091 | .094 | .100 | .093 |
|  | [-.284, .119] | [-.080, .105] | [-.135, .268] | [-.324, .046] | [-.354, .003] | [-.326, .044] | [-.291, .103] | [-.231, .135] |
|  | (.422) | (.790) | (.517) | (.140) | (.054) | (.135) | (.350) | (.606) |
| Rural Code=5 | -.171 | .001 | -.003 | -.143 | .046 | -.085 | .003 | -.010 |
|  | .157 | .072 | .157 | .144 | .140 | .145 | .154 | .143 |
|  | [-.479, .138] | [-.141, .143] | [-.311, .306] | [-.426, .140] | [-.227, .320] | [-.368, .199] | [-.298, .305] | [-.290, .270] |
|  | (.278) | (.988) | (.987) | (.321) | (.739) | (.558) | (.983) | (.945) |
| Rural Code=6 | .124 | -.012 | -.046 | .004 | -.070 | -.002 | .010 | .104 |
|  | .102 | .047 | .102 | .094 | .091 | .094 | .100 | .093 |
|  | [-.076, .324] | [-.105, .080] | [-.246, .154] | [-.179, .187] | [-.248, .108] | [-.186, .182] | [-.186, .206] | [-.077, .286] |
|  | (.224) | (.792) | (.651) | (.967) | (.440) | (.983) | (.922) | (.260) |
| Rural Code=7 | .052 | -.044 | .067 | .061 | .057 | .043 | -.034 | .068 |
|  | .141 | .065 | .141 | .129 | .125 | .129 | .138 | .128 |
|  | [-.224, .328] | [-.171, .083] | [-.209, .342] | [-.191, .314] | [-.188, .301] | [-.210, .296] | [-.304, .236] | [-.182, .318] |
|  | (.712) | (.496) | (.635) | (.635) | (.650) | (.738) | (.805) | (.594) |
| Rural Code=8 | .497 | -.022 | -.202 | -.424 | -.215 | -.089 | -.408 | .089 |
|  | .264 | .121 | .270 | .242 | .234 | .243 | .258 | .239 |
|  | [-.021, 1.014] | [-.260, .215] | [-.732, .327] | [-.899, .050] | [-.673, .244] | [-.565, .387] | [-.915, .098] | [-.380, .557] |
|  | (.060) | (.853) | (.454) | (.080) | (.359) | (.714) | (.114) | (.710) |
| Rural Code=9 | -.125 | -.058 | -.003 | .221 | .221 | -.038 | -.082 | -.187 |
|  | .224 | .103 | .224 | .205 | .199 | .206 | .219 | .203 |
|  | [-.564, .314] | [-.260, .143] | [-.441, .435] | [-.181, .623] | [-.168, .610] | [-.441, .366] | [-.511, .347] | [-.585, .210] |
|  | (.576) | (.571) | (.989) | (.281) | (.266) | (.855) | (.708) | (.356) |
| Alaska | .255 | -.022 | .343 | -.011 | -.464 | -.508 | .041 | -.269 |
|  | .449 | .206 | .449 | .412 | .398 | .413 | .440 | .407 |
|  | [-.625, 1.135] | [-.426, .381] | [-.536, 1.222] | [-.817, .796] | [-1.244, .315] | [-1.317, .301] | [-.820, .902] | [-1.066, .528] |
|  | (.571) | (.915) | (.445) | (.979) | (.243) | (.218) | (.926) | (.508) |
| Arizona | -.205 | .064 | .148 | -.033 | -.204 | -.128 | -.175 | .032 |
|  | .214 | .099 | .216 | .196 | .190 | .196 | .209 | .194 |
|  | [-.624, .215] | [-.129, .257] | [-.275, .571] | [-.416, .351] | [-.577, .168] | [-.513, .256] | [-.585, .234] | [-.348, .412] |
|  | (.339) | (.517) | (.493) | (.868) | (.282) | (.513) | (.401) | (.869) |
| Arkansas | .130 | .046 | -.004 | .112 | -.023 | .036 | .022 | .272 |
|  | .240 | .111 | .241 | .220 | .213 | .221 | .235 | .218 |
|  | [-.340, .601] | [-.171, .262] | [-.477, .469] | [-.319, .543] | [-.441, .395] | [-.396, .469] | [-.438, .483] | [-.155, .699] |
|  | (.587) | (.680) | (.987) | (.612) | (.913) | (.869) | (.925) | (.212) |
| California | -.007 | .090 | .005 | .189 | -.044 | -.019 | .010 | .143 |
|  | .188 | .087 | .190 | .172 | .167 | .173 | .184 | .171 |
|  | [-.376, .362] | [-.080, .259] | [-.367, .377] | [-.149, .527] | [-.371, .284] | [-.358, .320] | [-.351, .371] | [-.192, .477] |
|  | (.970) | (.299) | (.980) | (.274) | (.793) | (.912) | (.956) | (.403) |
| Colorado | .207 | .010 | -.025 | .170 | -.133 | -.202 | -.062 | -.150 |
|  | .241 | .111 | .244 | .221 | .215 | .222 | .236 | .219 |
|  | [-.266, .680] | [-.208, .229] | [-.503, .454] | [-.263, .603] | [-.554, .288] | [-.637, .232] | [-.524, .401] | [-.579, .280] |
|  | (.391) | (.926) | (.920) | (.442) | (.536) | (.362) | (.793) | (.495) |
| Connecticut | .021 | .273 | .271 | .238 | -.010 | .128 | .013 | .029 |
|  | .262 | .121 | .264 | .240 | .233 | .241 | .257 | .238 |
|  | [-.493, .536] | [.036, .510] | [-.246, .787] | [-.233, .709] | [-.467, .447] | [-.345, .600] | [-.490, .515] | [-.438, .496] |
|  | (.935) | (.024) | (.304) | (.322) | (.965) | (.596) | (.961) | (.903) |
| Delaware | .344 | -.044 | -.271 | .159 | -.385 | -.156 | .007 | -.312 |
|  | .339 | .157 | .341 | .310 | .302 | .311 | .332 | .308 |
|  | [-.321, 1.009] | [-.351, .264] | [-.938, .397] | [-.450, .767] | [-.977, .207] | [-.767, .454] | [-.642, .657] | [-.917, .292] |
|  | (.311) | (.781) | (.427) | (.609) | (.202) | (.616) | (.982) | (.311) |
| District of Columbia | .161 | .087 | .189 | .343 | -.088 | .029 | -.220 | .395 |
|  | .423 | .194 | .447 | .388 | .375 | .389 | .414 | .383 |
|  | [-.669, .991] | [-.293, .468] | [-.688, 1.066] | [-.417, 1.104] | [-.823, .647] | [-.733, .792] | [-1.032, .593] | [-.356, 1.146] |
|  | (.704) | (.653) | (.673) | (.376) | (.814) | (.940) | (.596) | (.303) |
| Florida | .171 | .068 | .075 | .121 | -.156 | -.004 | .016 | .107 |
|  | .190 | .087 | .192 | .174 | .169 | .175 | .186 | .173 |
|  | [-.202, .544] | [-.103, .240] | [-.302, .451] | [-.221, .463] | [-.487, .175] | [-.347, .339] | [-.349, .381] | [-.231, .446] |
|  | (.368) | (.435) | (.697) | (.488) | (.356) | (.981) | (.931) | (.534) |
| Georgia | .045 | .065 | .168 | -.021 | -.179 | .067 | -.161 | .005 |
|  | .206 | .095 | .208 | .189 | .183 | .189 | .202 | .187 |
|  | [-.359, .449] | [-.120, .251] | [-.239, .575] | [-.392, .349] | [-.537, .179] | [-.304, .438] | [-.556, .234] | [-.361, .372] |
|  | (.828) | (.491) | (.419) | (.910) | (.328) | (.724) | (.425) | (.977) |
| Hawaii | .024 | .301 | .553 | -.009 | -.406 | .132 | -.128 | .385 |
|  | .365 | .169 | .366 | .334 | .325 | .335 | .357 | .332 |
|  | [-.692, .739] | [-.030, .631] | [-.165, 1.271] | [-.663, .646] | [-1.043, .231] | [-.524, .789] | [-.827, .572] | [-.266, 1.035] |
|  | (.948) | (.074) | (.131) | (.979) | (.211) | (.693) | (.721) | (.247) |
| Idaho | .130 | -.114 | -.167 | -.364 | -.511 | -.341 | -.189 | -.019 |
|  | .306 | .141 | .307 | .280 | .272 | .281 | .299 | .278 |
|  | [-.470, .729] | [-.391, .163] | [-.770, .435] | [-.912, .185] | [-1.045, .023] | [-.891, .209] | [-.775, .396] | [-.564, .526] |
|  | (.672) | (.421) | (.586) | (.194) | (.061) | (.224) | (.527) | (.945) |
| Illinois | .042 | .107 | .025 | .320 | -.137 | -.046 | -.059 | .081 |
|  | .203 | .093 | .205 | .186 | .180 | .186 | .199 | .184 |
|  | [-.356, .440] | [-.076, .290] | [-.376, .426] | [-.044, .685] | [-.490, .217] | [-.411, .320] | [-.448, .331] | [-.280, .442] |
|  | (.836) | (.254) | (.902) | (.085) | (.448) | (.807) | (.768) | (.660) |
| Indiana | -.182 | .071 | .333 | .301 | -.035 | -.018 | -.063 | .037 |
|  | .225 | .103 | .226 | .206 | .199 | .206 | .220 | .204 |
|  | [-.622, .258] | [-.131, .274] | [-.110, .775] | [-.102, .704] | [-.426, .355] | [-.423, .386] | [-.494, .367] | [-.362, .436] |
|  | (.419) | (.489) | (.140) | (.143) | (.860) | (.929) | (.773) | (.857) |
| Iowa | .071 | -.033 | -.091 | .429 | -.069 | -.051 | .061 | -.019 |
|  | .267 | .122 | .267 | .245 | .236 | .245 | .261 | .242 |
|  | [-.452, .594] | [-.273, .207] | [-.615, .433] | [-.051, .908] | [-.532, .395] | [-.532, .429] | [-.451, .572] | [-.492, .455] |
|  | (.790) | (.788) | (.733) | (.080) | (.772) | (.835) | (.817) | (.938) |
| Kansas | .087 | .128 | .174 | .244 | -.033 | .170 | .324 | .070 |
|  | .323 | .148 | .323 | .296 | .287 | .297 | .316 | .293 |
|  | [-.546, .720] | [-.163, .419] | [-.460, .808] | [-.336, .824] | [-.595, .529] | [-.411, .752] | [-.295, .943] | [-.504, .644] |
|  | (.788) | (.389) | (.590) | (.410) | (.908) | (.566) | (.305) | (.811) |
| Kentucky | -.218 | -.037 | -.019 | .116 | -.219 | -.008 | -.103 | -.058 |
|  | .244 | .112 | .247 | .224 | .217 | .224 | .239 | .222 |
|  | [-.696, .261] | [-.257, .183] | [-.503, .464] | [-.323, .554] | [-.644, .206] | [-.447, .432] | [-.571, .365] | [-.492, .376] |
|  | (.373) | (.743) | (.938) | (.605) | (.313) | (.973) | (.666) | (.794) |
| Louisiana | .477 | .055 | .011 | -.048 | -.292 | .038 | -.001 | .102 |
|  | .245 | .113 | .247 | .224 | .218 | .225 | .240 | .223 |
|  | [-.004, .957] | [-.166, .277] | [-.472, .494] | [-.488, .392] | [-.719, .135] | [-.403, .479] | [-.471, .468] | [-.335, .538] |
|  | (.052) | (.624) | (.963) | (.830) | (.180) | (.866) | (.996) | (.648) |
| Maine | -.316 | .123 | -.388 | .167 | -.155 | -.337 | -.512 | -.784 |
|  | .340 | .157 | .341 | .311 | .302 | .312 | .332 | .309 |
|  | [-.982, .350] | [-.184, .431] | [-1.056, .280] | [-.442, .777] | [-.747, .438] | [-.948, .274] | [-1.162, .139] | [-1.388, -.179] |
|  | (.353) | (.431) | (.255) | (.590) | (.609) | (.280) | (.123) | (.011) |
| Maryland | -.084 | .138 | -.088 | .108 | .056 | -.047 | .043 | -.033 |
|  | .243 | .112 | .244 | .222 | .215 | .223 | .237 | .220 |
|  | [-.560, .391] | [-.081, .357] | [-.565, .390] | [-.328, .543] | [-.366, .479] | [-.483, .390] | [-.422, .508] | [-.465, .398] |
|  | (.729) | (.216) | (.719) | (.628) | (.793) | (.835) | (.857) | (.879) |
| Massachusetts | -.002 | .069 | .094 | .383 | .188 | .150 | .095 | .221 |
|  | .228 | .105 | .229 | .209 | .202 | .209 | .223 | .207 |
|  | [-.449, .444] | [-.137, .275] | [-.355, .543] | [-.026, .792] | [-.209, .584] | [-.260, .560] | [-.342, .532] | [-.184, .626] |
|  | (.992) | (.510) | (.681) | (.067) | (.354) | (.474) | (.670) | (.285) |
| Michigan | -.014 | .093 | .056 | .202 | -.149 | -.035 | -.120 | -.046 |
|  | .203 | .093 | .205 | .186 | .180 | .187 | .199 | .184 |
|  | [-.412, .385] | [-.090, .276] | [-.347, .458] | [-.163, .567] | [-.502, .205] | [-.401, .331] | [-.509, .270] | [-.407, .315] |
|  | (.947) | (.319) | (.787) | (.279) | (.409) | (.849) | (.547) | (.804) |
| Minnesota | .070 | .041 | .522 | .132 | -.187 | .021 | -.024 | -.076 |
|  | .240 | .110 | .241 | .220 | .213 | .220 | .235 | .217 |
|  | [-.400, .540] | [-.175, .257] | [.050, .994] | [-.298, .563] | [-.604, .230] | [-.411, .452] | [-.484, .435] | [-.502, .351] |
|  | (.770) | (.708) | (.030) | (.547) | (.379) | (.926) | (.917) | (.728) |
| Mississippi | .410 | -.073 | -.030 | .249 | -.192 | -.145 | -.406 | -.020 |
|  | .272 | .125 | .272 | .249 | .241 | .249 | .266 | .246 |
|  | [-.122, .942] | [-.317, .171] | [-.564, .503] | [-.239, .736] | [-.663, .280] | [-.634, .344] | [-.926, .115] | [-.502, .462] |
|  | (.131) | (.559) | (.912) | (.317) | (.426) | (.560) | (.127) | (.935) |
| Missouri | -.123 | .046 | -.058 | -.029 | -.181 | -.087 | -.082 | -.051 |
|  | .220 | .101 | .221 | .201 | .195 | .202 | .215 | .199 |
|  | [-.554, .308] | [-.152, .244] | [-.492, .375] | [-.424, .366] | [-.563, .201] | [-.483, .309] | [-.503, .340] | [-.442, .340] |
|  | (.575) | (.650) | (.792) | (.886) | (.354) | (.667) | (.704) | (.798) |
| Montana | -.207 | .056 | -.078 | .455 | -.237 | .210 | -.146 | .060 |
|  | .339 | .156 | .340 | .310 | .301 | .311 | .331 | .308 |
|  | [-.871, .458] | [-.251, .362] | [-.745, .588] | [-.154, 1.063] | [-.828, .353] | [-.400, .820] | [-.795, .504] | [-.543, .664] |
|  | (.542) | (.722) | (.818) | (.143) | (.431) | (.500) | (.660) | (.845) |
| Nebraska | .069 | -.086 | .308 | .271 | -.209 | -.054 | .322 | .091 |
|  | .349 | .165 | .349 | .320 | .309 | .321 | .342 | .316 |
|  | [-.615, .753] | [-.409, .237] | [-.376, .992] | [-.356, .898] | [-.815, .397] | [-.682, .575] | [-.348, .991] | [-.529, .710] |
|  | (.844) | (.604) | (.377) | (.397) | (.499) | (.867) | (.346) | (.775) |
| Nevada | .097 | .033 | .203 | .258 | .111 | -.034 | -.102 | .079 |
|  | .239 | .111 | .241 | .219 | .213 | .220 | .234 | .217 |
|  | [-.372, .566] | [-.185, .250] | [-.269, .675] | [-.172, .687] | [-.306, .528] | [-.464, .397] | [-.560, .357] | [-.347, .505] |
|  | (.686) | (.767) | (.400) | (.239) | (.602) | (.878) | (.663) | (.717) |
| New Hampshire | .292 | -.029 | .069 | .083 | -.110 | -.320 | -.181 | .076 |
|  | .287 | .133 | .288 | .263 | .255 | .264 | .281 | .261 |
|  | [-.271, .855] | [-.289, .231] | [-.496, .634] | [-.432, .598] | [-.611, .391] | [-.837, .196] | [-.731, .369] | [-.436, .587] |
|  | (.309) | (.827) | (.811) | (.751) | (.666) | (.225) | (.518) | (.772) |
| New Jersey | .304 | .090 | -.174 | .299 | .055 | .135 | .032 | .254 |
|  | .221 | .102 | .223 | .203 | .196 | .204 | .217 | .201 |
|  | [-.130, .739] | [-.110, .289] | [-.612, .263] | [-.098, .697] | [-.329, .440] | [-.264, .534] | [-.393, .456] | [-.139, .647] |
|  | (.169) | (.378) | (.434) | (.140) | (.778) | (.507) | (.884) | (.206) |
| New Mexico | .264 | .074 | -.197 | .119 | -.187 | -.079 | .169 | -.021 |
|  | .264 | .122 | .266 | .242 | .235 | .243 | .259 | .240 |
|  | [-.255, .782] | [-.164, .313] | [-.718, .324] | [-.356, .594] | [-.648, .274] | [-.555, .397] | [-.338, .676] | [-.492, .449] |
|  | (.319) | (.542) | (.459) | (.623) | (.426) | (.745) | (.513) | (.930) |
| New York | .219 | .071 | -.151 | .352 | .056 | .002 | -.005 | .195 |
|  | .194 | .089 | .196 | .178 | .172 | .178 | .190 | .176 |
|  | [-.162, .599] | [-.104, .245] | [-.534, .233] | [.003, .700] | [-.281, .394] | [-.348, .351] | [-.377, .367] | [-.150, .540] |
|  | (.260) | (.428) | (.441) | (.048) | (.744) | (.992) | (.980) | (.267) |
| North Carolina | -.139 | .104 | .050 | .240 | -.086 | .036 | .148 | .124 |
|  | .209 | .096 | .211 | .192 | .186 | .192 | .205 | .190 |
|  | [-.549, .272] | [-.085, .293] | [-.363, .464] | [-.136, .616] | [-.450, .278] | [-.341, .413] | [-.254, .549] | [-.248, .496] |
|  | (.508) | (.280) | (.811) | (.211) | (.644) | (.852) | (.470) | (.513) |
| North Dakota | .096 | .151 | .174 | .062 | -.402 | .288 | -.345 | -.165 |
|  | .425 | .196 | .427 | .389 | .378 | .391 | .416 | .386 |
|  | [-.738, .930] | [-.233, .536] | [-.662, 1.010] | [-.701, .825] | [-1.143, .340] | [-.478, 1.053] | [-1.160, .470] | [-.922, .593] |
|  | (.821) | (.441) | (.684) | (.873) | (.288) | (.461) | (.407) | (.670) |
| Ohio | .018 | .083 | .045 | .154 | -.074 | -.035 | -.250 | -.077 |
|  | .204 | .094 | .205 | .187 | .181 | .187 | .199 | .185 |
|  | [-.381, .417] | [-.101, .266] | [-.357, .447] | [-.212, .519] | [-.428, .280] | [-.402, .332] | [-.640, .141] | [-.439, .285] |
|  | (.930) | (.378) | (.825) | (.410) | (.682) | (.852) | (.210) | (.677) |
| Oklahoma | -.071 | .026 | .345 | .030 | -.041 | -.042 | -.115 | -.130 |
|  | .273 | .125 | .274 | .250 | .242 | .251 | .267 | .247 |
|  | [-.606, .464] | [-.219, .272] | [-.191, .882] | [-.460, .520] | [-.515, .434] | [-.534, .449] | [-.639, .408] | [-.615, .355] |
|  | (.795) | (.834) | (.207) | (.904) | (.866) | (.866) | (.666) | (.599) |
| Oregon | -.179 | .097 | .396 | .129 | -.152 | .122 | -.063 | .131 |
|  | .225 | .104 | .227 | .206 | .200 | .207 | .220 | .205 |
|  | [-.621, .263] | [-.107, .301] | [-.049, .840] | [-.275, .533] | [-.545, .241] | [-.283, .528] | [-.494, .369] | [-.270, .533] |
|  | (.428) | (.351) | (.081) | (.532) | (.448) | (.555) | (.776) | (.522) |
| Pennsylvania | .015 | .094 | .132 | .134 | -.190 | -.041 | -.031 | .015 |
|  | .193 | .089 | .194 | .177 | .171 | .177 | .189 | .175 |
|  | [-.364, .393] | [-.080, .267] | [-.248, .513] | [-.213, .480] | [-.525, .146] | [-.389, .306] | [-.401, .338] | [-.328, .357] |
|  | (.940) | (.291) | (.496) | (.450) | (.268) | (.815) | (.868) | (.934) |
| Rhode Island | .053 | .156 | -.168 | .462 | .031 | .136 | -.012 | -.025 |
|  | .423 | .194 | .422 | .387 | .374 | .388 | .414 | .383 |
|  | [-.775, .882] | [-.224, .535] | [-.996, .660] | [-.297, 1.221] | [-.703, .764] | [-.626, .897] | [-.822, .799] | [-.775, .725] |
|  | (.900) | (.422) | (.691) | (.233) | (.935) | (.727) | (.977) | (.949) |
| South Carolina | .062 | .044 | -.015 | .067 | -.131 | .145 | .024 | .004 |
|  | .236 | .109 | .238 | .216 | .210 | .217 | .231 | .215 |
|  | [-.402, .525] | [-.170, .257] | [-.481, .451] | [-.357, .491] | [-.543, .281] | [-.281, .570] | [-.429, .477] | [-.416, .425] |
|  | (.794) | (.689) | (.950) | (.758) | (.533) | (.505) | (.918) | (.984) |
| South Dakota | .210 | .078 | .276 | -.685 | -.719 | -.085 | -.038 | -.362 |
|  | .391 | .180 | .392 | .358 | .348 | .359 | .383 | .355 |
|  | [-.557, .977] | [-.275, .431] | [-.492, 1.044] | [-1.387, .017] | [-1.400, -.038] | [-.789, .620] | [-.788, .712] | [-1.058, .334] |
|  | (.591) | (.666) | (.482) | (.056) | (.039) | (.814) | (.921) | (.308) |
| Tennessee | .076 | .112 | .153 | .342 | .142 | .159 | -.179 | .071 |
|  | .231 | .106 | .232 | .212 | .205 | .212 | .226 | .210 |
|  | [-.377, .529] | [-.097, .320] | [-.303, .608] | [-.073, .756] | [-.260, .544] | [-.257, .575] | [-.622, .264] | [-.340, .482] |
|  | (.743) | (.293) | (.511) | (.106) | (.490) | (.454) | (.429) | (.735) |
| Texas | -.201 | .084 | .059 | .074 | -.264 | -.029 | .013 | .092 |
|  | .193 | .089 | .194 | .177 | .171 | .177 | .189 | .175 |
|  | [-.580, .177] | [-.090, .258] | [-.322, .441] | [-.273, .420] | [-.599, .072] | [-.377, .318] | [-.357, .383] | [-.251, .435] |
|  | (.297) | (.342) | (.760) | (.677) | (.123) | (.869) | (.946) | (.599) |
| Utah | -.108 | .055 | .405 | .314 | -.094 | -.197 | .163 | .115 |
|  | .268 | .124 | .269 | .245 | .238 | .246 | .262 | .243 |
|  | [-.633, .417] | [-.187, .297] | [-.123, .932] | [-.167, .794] | [-.560, .373] | [-.679, .284] | [-.350, .675] | [-.362, .592] |
|  | (.687) | (.657) | (.133) | (.201) | (.694) | (.422) | (.534) | (.636) |
| Vermont | .617 | .078 | -.049 | -.079 | -.448 | -.159 | .146 | -.655 |
|  | .387 | .177 | .387 | .354 | .343 | .355 | .379 | .350 |
|  | [-.141, 1.375] | [-.270, .426] | [-.807, .709] | [-.774, .616] | [-1.120, .223] | [-.856, .538] | [-.596, .888] | [-1.342, .031] |
|  | (.111) | (.660) | (.899) | (.824) | (.191) | (.655) | (.699) | (.061) |
| Virginia | .126 | .115 | .131 | .089 | -.182 | -.150 | -.248 | .036 |
|  | .207 | .095 | .208 | .189 | .184 | .190 | .202 | .188 |
|  | [-.279, .532] | [-.072, .302] | [-.277, .540] | [-.282, .460] | [-.542, .179] | [-.522, .223] | [-.644, .148] | [-.333, .405] |
|  | (.541) | (.227) | (.529) | (.639) | (.323) | (.431) | (.220) | (.848) |
| Washington | .373 | .083 | .272 | .266 | -.002 | .034 | -.002 | -.031 |
|  | .217 | .100 | .218 | .199 | .193 | .199 | .212 | .197 |
|  | [-.052, .799] | [-.113, .279] | [-.157, .700] | [-.124, .655] | [-.380, .376] | [-.357, .424] | [-.418, .414] | [-.417, .355] |
|  | (.085) | (.408) | (.214) | (.181) | (.992) | (.865) | (.993) | (.874) |
| West Virginia | -.227 | .131 | .171 | .212 | .027 | .025 | -.041 | .158 |
|  | .276 | .127 | .277 | .253 | .245 | .254 | .270 | .250 |
|  | [-.768, .314] | [-.117, .380] | [-.372, .713] | [-.284, .708] | [-.452, .507] | [-.472, .522] | [-.571, .488] | [-.332, .649] |
|  | (.412) | (.300) | (.538) | (.402) | (.911) | (.921) | (.878) | (.527) |
| Wisconsin | .073 | .079 | -.007 | .072 | -.385 | -.186 | -.268 | -.179 |
|  | .224 | .103 | .226 | .205 | .199 | .206 | .219 | .203 |
|  | [-.367, .512] | [-.123, .282] | [-.449, .436] | [-.331, .474] | [-.775, .005] | [-.590, .218] | [-.698, .162] | [-.578, .220] |
|  | (.746) | (.442) | (.976) | (.727) | (.053) | (.367) | (.223) | (.379) |
| Wyoming | .605 | .129 | -.630 | -.365 | -.265 | .042 | -.016 | -.009 |
|  | .654 | .305 | .658 | .597 | .586 | .599 | .637 | .598 |
|  | [-.676, 1.886] | [-.469, .728] | [-1.921, .661] | [-1.535, .804] | [-1.413, .883] | [-1.131, 1.216] | [-1.265, 1.233] | [-1.180, 1.163] |
|  | (.355) | (.672) | (.339) | (.540) | (.651) | (.944) | (.980) | (.989) |
| N | 2982 | 2980 | 2968 | 2980 | 2981 | 2981 | 2981 | 2979 |
| Pseudo-R^2^ |  |  |  |  |  |  |  |  |
| Model χ^2^ | 201.1 | 305.7 | 444.8 | 278.2 | 274.1 | 209.4 | 227.4 | 291.6 |
| d.f. | 87 | 87 | 87 | 87 | 87 | 87 | 87 | 87 |
| p-value | <.001 | <.001 | <.001 | <.001 | <.001 | <.001 | <.001 | <.001 |

S11 Table. Regression Results, Health Attitudes, Multilevel Models, Controlling for Deaths.

|  | (1) | (2) | (3) | (4) | (5) | (6) | (7) | (8) |
| --- | --- | --- | --- | --- | --- | --- | --- | --- |
|  | numberdeaths | spendingincrease | notenoughtests | getsick | friendsick | cannotreturn | schoolsclosed | hardgetitems |
| main |  |  |  |  |  |  |  |  |
| Democrat | .357 | .331 | .868 | .545 | .511 | .438 | .271 | .308 |
|  | .053 | .024 | .053 | .048 | .047 | .049 | .052 | .048 |
|  | [.254, .461] | [.284, .379] | [.764, .971] | [.450, .640] | [.419, .603] | [.343, .533] | [.169, .372] | [.214, .402] |
|  | (<.001) | (<.001) | (<.001) | (<.001) | (<.001) | (<.001) | (<.001) | (<.001) |
| Other | .099 | .099 | .504 | .192 | .131 | .096 | .063 | .058 |
|  | .054 | .025 | .054 | .049 | .047 | .049 | .052 | .049 |
|  | [-.006, .204] | [.051, .147] | [.399, .609] | [.096, .288] | [.038, .224] | [-<.001, .193] | [-.039, .166] | [-.037, .153] |
|  | (.065) | (<.001) | (<.001) | (<.001) | (.006) | (.050) | (.227) | (.233) |
| Local COVID-19 Deaths | <.001 | -<.001 | -<.001 | <.001 | <.001 | <.001 | -<.001 | <.001 |
|  | <.001 | <.001 | <.001 | <.001 | <.001 | <.001 | <.001 | <.001 |
|  | [-<.001, .000] | [-<.001, .000] | [-<.001, .000] | [-<.001, .000] | [-<.001, .000] | [-<.001, .000] | [-<.001, .000] | [-<.001, .000] |
|  | (.750) | (.989) | (.113) | (.242) | (.272) | (.069) | (.889) | (.062) |
| 35- | -.037 | .041 | .070 | .062 | .069 | -.137 | .037 | .013 |
|  | .059 | .027 | .059 | .054 | .053 | .055 | .058 | .054 |
|  | [-.153, .080] | [-.013, .094] | [-.046, .186] | [-.045, .169] | [-.034, .172] | [-.244, -.030] | [-.077, .151] | [-.092, .119] |
|  | (.539) | (.134) | (.238) | (.254) | (.192) | (.012) | (.522) | (.804) |
| 50- | .154 | .031 | .134 | -.049 | -.016 | -.214 | -.103 | -.189 |
|  | .056 | .026 | .056 | .051 | .050 | .052 | .055 | .051 |
|  | [.044, .264] | [-.020, .081] | [.024, .243] | [-.150, .051] | [-.113, .081] | [-.315, -.113] | [-.210, .005] | [-.289, -.090] |
|  | (.006) | (.235) | (.017) | (.337) | (.746) | (<.001) | (.061) | (<.001) |
| 70- | .336 | .075 | .124 | -.002 | -.008 | -.284 | -.544 | -.406 |
|  | .071 | .033 | .071 | .065 | .063 | .065 | .069 | .064 |
|  | [.197, .475] | [.012, .139] | [-.014, .263] | [-.130, .125] | [-.131, .116] | [-.412, -.156] | [-.680, -.407] | [-.531, -.280] |
|  | (<.001) | (.020) | (.079) | (.971) | (.902) | (<.001) | (<.001) | (<.001) |
| gender | .047 | -.026 | .010 | .145 | .134 | .029 | .047 | .144 |
|  | .041 | .019 | .041 | .037 | .036 | .038 | .040 | .037 |
|  | [-.033, .127] | [-.063, .010] | [-.070, .090] | [.072, .218] | [.064, .205] | [-.045, .102] | [-.032, .125] | [.071, .216] |
|  | (.247) | (.160) | (.799) | (<.001) | (<.001) | (.444) | (.243) | (<.001) |
| white | -.039 | .069 | .242 | .032 | .008 | .137 | -.076 | .030 |
|  | .049 | .022 | .049 | .045 | .043 | .045 | .048 | .044 |
|  | [-.135, .056] | [.025, .113] | [.147, .338] | [-.056, .120] | [-.077, .093] | [.050, .225] | [-.170, .017] | [-.057, .116] |
|  | (.420) | (.002) | (<.001) | (.476) | (.855) | (.002) | (.110) | (.499) |
| married | .006 | -.015 | -.120 | .102 | .082 | .022 | .068 | .102 |
|  | .045 | .021 | .045 | .041 | .040 | .041 | .044 | .041 |
|  | [-.082, .094] | [-.055, .025] | [-.208, -.032] | [.022, .183] | [.004, .160] | [-.059, .103] | [-.018, .154] | [.023, .182] |
|  | (.890) | (.467) | (.007) | (.013) | (.038) | (.592) | (.121) | (.012) |
| High school graduate | -.039 | -.057 | -.016 | -.064 | -.117 | -.070 | .014 | -.214 |
|  | .120 | .055 | .120 | .110 | .106 | .110 | .117 | .108 |
|  | [-.274, .195] | [-.165, .050] | [-.251, .219] | [-.279, .151] | [-.325, .090] | [-.286, .145] | [-.216, .244] | [-.427, -.002] |
|  | (.742) | (.296) | (.894) | (.559) | (.268) | (.522) | (.905) | (.048) |
| Some college | -.075 | .005 | .230 | -.017 | -.099 | -.064 | -.019 | -.311 |
|  | .122 | .056 | .122 | .112 | .108 | .112 | .119 | .110 |
|  | [-.314, .164] | [-.105, .115] | [-.010, .470] | [-.236, .202] | [-.311, .113] | [-.284, .155] | [-.253, .215] | [-.528, -.095] |
|  | (.540) | (.928) | (.060) | (.879) | (.360) | (.566) | (.872) | (.005) |
| 2-year | -.009 | .019 | .152 | -.012 | -.087 | -.101 | -.026 | -.346 |
|  | .128 | .059 | .129 | .118 | .114 | .118 | .126 | .116 |
|  | [-.260, .243] | [-.096, .135] | [-.100, .404] | [-.243, .218] | [-.309, .136] | [-.332, .130] | [-.272, .221] | [-.573, -.118] |
|  | (.945) | (.741) | (.236) | (.916) | (.446) | (.389) | (.839) | (.003) |
| 4-year | -.056 | .026 | .150 | -.051 | -.059 | -.042 | -.060 | -.312 |
|  | .125 | .057 | .125 | .114 | .110 | .114 | .122 | .113 |
|  | [-.300, .188] | [-.085, .138] | [-.094, .395] | [-.274, .173] | [-.276, .157] | [-.266, .182] | [-.298, .179] | [-.532, -.091] |
|  | (.651) | (.643) | (.229) | (.658) | (.591) | (.714) | (.625) | (.006) |
| Post-grad | -.052 | .046 | .202 | .055 | .011 | .004 | .003 | -.406 |
|  | .131 | .060 | .131 | .120 | .116 | .120 | .128 | .118 |
|  | [-.309, .204] | [-.072, .163] | [-.055, .459] | [-.181, .290] | [-.216, .238] | [-.232, .239] | [-.249, .254] | [-.638, -.174] |
|  | (.689) | (.443) | (.123) | (.649) | (.922) | (.977) | (.984) | (.001) |
| income=2 | .064 | -.041 | .360 | -.072 | .001 | -.158 | -.154 | -.112 |
|  | .115 | .053 | .116 | .106 | .102 | .106 | .113 | .104 |
|  | [-.162, .290] | [-.144, .063] | [.132, .587] | [-.279, .136] | [-.199, .201] | [-.366, .050] | [-.376, .067] | [-.317, .093] |
|  | (.578) | (.443) | (.002) | (.498) | (.991) | (.136) | (.172) | (.284) |
| income=3 | .171 | -.005 | .305 | -.093 | -.012 | -.110 | -.042 | -.147 |
|  | .112 | .051 | .113 | .103 | .099 | .103 | .109 | .101 |
|  | [-.048, .390] | [-.105, .096] | [.084, .525] | [-.294, .108] | [-.206, .182] | [-.311, .091] | [-.257, .172] | [-.345, .051] |
|  | (.125) | (.926) | (.007) | (.363) | (.906) | (.284) | (.699) | (.146) |
| income=4 | .122 | .025 | .283 | .015 | .094 | -.080 | -.048 | -.115 |
|  | .115 | .052 | .115 | .105 | .101 | .105 | .112 | .104 |
|  | [-.102, .347] | [-.078, .127] | [.058, .509] | [-.191, .221] | [-.105, .293] | [-.286, .127] | [-.267, .172] | [-.318, .088] |
|  | (.285) | (.639) | (.014) | (.888) | (.354) | (.450) | (.672) | (.268) |
| income=5 | .179 | .028 | .363 | -.058 | .009 | -.158 | -.035 | -.197 |
|  | .117 | .054 | .118 | .107 | .103 | .107 | .114 | .106 |
|  | [-.050, .409] | [-.077, .133] | [.133, .594] | [-.268, .152] | [-.193, .212] | [-.368, .053] | [-.260, .189] | [-.404, .010] |
|  | (.125) | (.602) | (.002) | (.586) | (.928) | (.142) | (.758) | (.062) |
| income=6 | .217 | -.004 | .362 | .014 | .009 | -.087 | .087 | -.145 |
|  | .118 | .054 | .119 | .108 | .105 | .109 | .116 | .107 |
|  | [-.015, .448] | [-.110, .102] | [.130, .595] | [-.198, .227] | [-.196, .214] | [-.300, .126] | [-.140, .313] | [-.354, .065] |
|  | (.067) | (.938) | (.002) | (.894) | (.934) | (.422) | (.454) | (.176) |
| income=7 | .082 | .001 | .389 | -.072 | -.018 | -.176 | -.099 | -.280 |
|  | .127 | .058 | .127 | .116 | .112 | .116 | .124 | .114 |
|  | [-.166, .330] | [-.112, .115] | [.140, .638] | [-.300, .155] | [-.237, .202] | [-.404, .052] | [-.342, .144] | [-.505, -.056] |
|  | (.518) | (.981) | (.002) | (.532) | (.874) | (.131) | (.423) | (.014) |
| income=8 | .117 | .010 | .326 | -.274 | -.227 | -.263 | -.181 | -.368 |
|  | .123 | .056 | .124 | .113 | .109 | .113 | .121 | .111 |
|  | [-.125, .359] | [-.101, .120] | [.084, .569] | [-.495, -.052] | [-.441, -.014] | [-.485, -.041] | [-.417, .056] | [-.587, -.150] |
|  | (.343) | (.863) | (.008) | (.015) | (.037) | (.020) | (.135) | (.001) |
| income=9 | .349 | .009 | .467 | -.189 | -.069 | -.084 | -.108 | -.311 |
|  | .122 | .056 | .122 | .112 | .108 | .112 | .119 | .110 |
|  | [.111, .588] | [-.100, .118] | [.227, .707] | [-.408, .030] | [-.280, .143] | [-.303, .136] | [-.342, .126] | [-.527, -.095] |
|  | (.004) | (.873) | (<.001) | (.091) | (.524) | (.455) | (.366) | (.005) |
| income=10 | .220 | .004 | .491 | -.181 | -.079 | -.103 | -.108 | -.376 |
|  | .127 | .058 | .127 | .116 | .112 | .116 | .124 | .115 |
|  | [-.028, .469] | [-.109, .118] | [.241, .740] | [-.408, .047] | [-.299, .141] | [-.331, .126] | [-.351, .135] | [-.601, -.151] |
|  | (.082) | (.938) | (<.001) | (.120) | (.481) | (.378) | (.382) | (.001) |
| income=11 | .370 | .001 | .466 | -.074 | -.001 | .075 | -.099 | -.353 |
|  | .130 | .060 | .130 | .119 | .115 | .120 | .127 | .118 |
|  | [.114, .625] | [-.116, .118] | [.210, .722] | [-.308, .159] | [-.227, .225] | [-.159, .309] | [-.349, .151] | [-.584, -.122] |
|  | (.005) | (.988) | (<.001) | (.533) | (.992) | (.531) | (.437) | (.003) |
| income=12 | .234 | .034 | .363 | -.138 | .003 | .024 | -.183 | -.418 |
|  | .143 | .066 | .143 | .131 | .127 | .131 | .140 | .129 |
|  | [-.046, .515] | [-.095, .163] | [.082, .644] | [-.395, .119] | [-.245, .251] | [-.234, .282] | [-.458, .091] | [-.672, -.165] |
|  | (.101) | (.605) | (.011) | (.291) | (.980) | (.854) | (.191) | (.001) |
| income=13 | .211 | .063 | .646 | -.262 | -.135 | -.086 | -.419 | -.651 |
|  | .198 | .091 | .198 | .181 | .175 | .182 | .194 | .179 |
|  | [-.177, .599] | [-.115, .241] | [.258, 1.034] | [-.618, .093] | [-.479, .208] | [-.443, .270] | [-.799, -.040] | [-1.002, -.300] |
|  | (.286) | (.489) | (.001) | (.148) | (.440) | (.634) | (.030) | (<.001) |
| income=14 | .304 | -.078 | .526 | -.240 | -.082 | -.200 | -.071 | -.451 |
|  | .210 | .096 | .209 | .192 | .186 | .193 | .205 | .190 |
|  | [-.107, .715] | [-.266, .111] | [.116, .937] | [-.617, .136] | [-.446, .282] | [-.578, .177] | [-.473, .332] | [-.823, -.080] |
|  | (.148) | (.419) | (.012) | (.211) | (.659) | (.299) | (.731) | (.017) |
| income=15 | .274 | .234 | 1.373 | .444 | .235 | .394 | -.071 | -1.134 |
|  | .635 | .290 | .633 | .582 | .562 | .584 | .622 | .574 |
|  | [-.971, 1.518] | [-.335, .803] | [.133, 2.613] | [-.696, 1.585] | [-.867, 1.336] | [-.750, 1.537] | [-1.290, 1.147] | [-2.259, -.008] |
|  | (.667) | (.421) | (.030) | (.445) | (.676) | (.500) | (.909) | (.048) |
| income=16 | .529 | .045 | .867 | -.301 | -.094 | .168 | -.037 | -.487 |
|  | .298 | .137 | .298 | .273 | .264 | .274 | .292 | .270 |
|  | [-.056, 1.114] | [-.223, .312] | [.283, 1.450] | [-.837, .235] | [-.611, .424] | [-.369, .706] | [-.610, .535] | [-1.015, .042] |
|  | (.076) | (.743) | (.004) | (.271) | (.722) | (.539) | (.899) | (.071) |
| income=17 | .272 | -.033 | .343 | -.235 | -.132 | -.229 | -.263 | -.421 |
|  | .108 | .050 | .109 | .099 | .096 | .100 | .106 | .098 |
|  | [.059, .484] | [-.130, .065] | [.129, .556] | [-.430, -.041] | [-.320, .056] | [-.424, -.033] | [-.471, -.055] | [-.614, -.229] |
|  | (.012) | (.509) | (.002) | (.018) | (.168) | (.022) | (.013) | (<.001) |
| Rural Code=2 | .010 | -.016 | -.075 | .010 | -.049 | -.016 | -.102 | .011 |
|  | .056 | .026 | .056 | .051 | .050 | .051 | .055 | .051 |
|  | [-.099, .120] | [-.067, .035] | [-.185, .035] | [-.091, .110] | [-.147, .048] | [-.117, .084] | [-.209, .006] | [-.089, .111] |
|  | (.854) | (.535) | (.179) | (.853) | (.323) | (.749) | (.063) | (.832) |
| Rural Code=3 | -.004 | -.028 | -.130 | .038 | .064 | .045 | .020 | .079 |
|  | .075 | .035 | .076 | .069 | .067 | .069 | .074 | .069 |
|  | [-.152, .144] | [-.097, .040] | [-.278, .018] | [-.097, .173] | [-.068, .195] | [-.091, .180] | [-.125, .164] | [-.056, .213] |
|  | (.959) | (.416) | (.085) | (.584) | (.342) | (.520) | (.790) | (.252) |
| Rural Code=4 | -.077 | .012 | .040 | -.121 | -.159 | -.112 | -.096 | -.019 |
|  | .104 | .048 | .104 | .096 | .092 | .096 | .102 | .094 |
|  | [-.281, .127] | [-.081, .106] | [-.164, .243] | [-.308, .066] | [-.340, .022] | [-.300, .075] | [-.296, .103] | [-.204, .166] |
|  | (.459) | (.795) | (.704) | (.206) | (.086) | (.240) | (.345) | (.840) |
| Rural Code=5 | -.165 | .001 | -.032 | -.123 | .065 | -.053 | .001 | .022 |
|  | .159 | .073 | .158 | .145 | .141 | .146 | .155 | .144 |
|  | [-.475, .146] | [-.142, .144] | [-.343, .278] | [-.407, .162] | [-.211, .341] | [-.339, .232] | [-.303, .305] | [-.260, .304] |
|  | (.299) | (.989) | (.839) | (.397) | (.645) | (.714) | (.996) | (.878) |
| Rural Code=6 | .129 | -.013 | -.071 | .020 | -.055 | .024 | .008 | .131 |
|  | .103 | .048 | .103 | .095 | .092 | .095 | .101 | .094 |
|  | [-.073, .332] | [-.106, .081] | [-.273, .132] | [-.165, .206] | [-.235, .125] | [-.162, .210] | [-.190, .206] | [-.053, .314] |
|  | (.211) | (.792) | (.494) | (.830) | (.549) | (.803) | (.939) | (.164) |
| Rural Code=7 | .058 | -.044 | .038 | .081 | .074 | .073 | -.036 | .099 |
|  | .142 | .065 | .142 | .130 | .126 | .130 | .139 | .129 |
|  | [-.220, .336] | [-.172, .084] | [-.240, .316] | [-.174, .335] | [-.172, .321] | [-.182, .329] | [-.308, .235] | [-.153, .351] |
|  | (.684) | (.498) | (.788) | (.535) | (.554) | (.573) | (.793) | (.442) |
| Rural Code=8 | .502 | -.023 | -.229 | -.406 | -.198 | -.061 | -.411 | .117 |
|  | .265 | .121 | .271 | .242 | .234 | .243 | .259 | .239 |
|  | [-.017, 1.021] | [-.260, .215] | [-.760, .301] | [-.881, .069] | [-.658, .261] | [-.537, .416] | [-.918, .097] | [-.352, .587] |
|  | (.058) | (.852) | (.397) | (.094) | (.397) | (.802) | (.113) | (.624) |
| Rural Code=9 | -.119 | -.058 | -.032 | .241 | .239 | -.008 | -.085 | -.157 |
|  | .225 | .103 | .224 | .206 | .199 | .206 | .220 | .204 |
|  | [-.560, .321] | [-.261, .144] | [-.471, .408] | [-.163, .644] | [-.152, .629] | [-.412, .397] | [-.515, .346] | [-.556, .242] |
|  | (.595) | (.572) | (.888) | (.242) | (.231) | (.970) | (.700) | (.441) |
| Alaska | .254 | -.022 | .344 | -.012 | -.466 | -.510 | .041 | -.271 |
|  | .449 | .206 | .448 | .411 | .398 | .413 | .440 | .406 |
|  | [-.626, 1.135] | [-.426, .381] | [-.535, 1.223] | [-.818, .795] | [-1.245, .314] | [-1.318, .299] | [-.820, .903] | [-1.067, .525] |
|  | (.571) | (.915) | (.443) | (.977) | (.242) | (.216) | (.925) | (.505) |
| Arizona | -.204 | .064 | .143 | -.029 | -.202 | -.124 | -.176 | .037 |
|  | .214 | .099 | .216 | .196 | .190 | .196 | .209 | .194 |
|  | [-.623, .216] | [-.129, .257] | [-.280, .566] | [-.413, .354] | [-.574, .171] | [-.508, .261] | [-.586, .234] | [-.343, .417] |
|  | (.341) | (.517) | (.507) | (.880) | (.289) | (.528) | (.400) | (.849) |
| Arkansas | .130 | .046 | -.003 | .111 | -.024 | .035 | .022 | .270 |
|  | .240 | .111 | .241 | .220 | .213 | .220 | .235 | .218 |
|  | [-.341, .601] | [-.171, .262] | [-.475, .470] | [-.320, .541] | [-.442, .393] | [-.397, .467] | [-.438, .483] | [-.156, .697] |
|  | (.588) | (.680) | (.991) | (.615) | (.909) | (.875) | (.924) | (.215) |
| California | -.011 | .090 | .027 | .173 | -.058 | -.043 | .012 | .119 |
|  | .189 | .087 | .190 | .173 | .167 | .173 | .185 | .171 |
|  | [-.381, .358] | [-.080, .260] | [-.346, .400] | [-.165, .512] | [-.386, .271] | [-.382, .297] | [-.350, .374] | [-.217, .454] |
|  | (.951) | (.300) | (.886) | (.316) | (.731) | (.805) | (.948) | (.488) |
| Colorado | .208 | .010 | -.029 | .173 | -.130 | -.197 | -.062 | -.145 |
|  | .241 | .111 | .244 | .221 | .215 | .222 | .236 | .219 |
|  | [-.265, .681] | [-.208, .229] | [-.507, .449] | [-.260, .606] | [-.551, .290] | [-.632, .237] | [-.525, .400] | [-.574, .285] |
|  | (.389) | (.926) | (.905) | (.434) | (.544) | (.373) | (.792) | (.508) |
| Connecticut | .012 | .273 | .317 | .207 | -.039 | .079 | .017 | -.020 |
|  | .264 | .122 | .265 | .242 | .235 | .242 | .258 | .239 |
|  | [-.506, .529] | [.035, .511] | [-.202, .837] | [-.267, .681] | [-.498, .421] | [-.396, .554] | [-.489, .522] | [-.490, .449] |
|  | (.964) | (.025) | (.231) | (.392) | (.869) | (.744) | (.949) | (.932) |
| Delaware | .344 | -.044 | -.270 | .158 | -.386 | -.158 | .008 | -.313 |
|  | .339 | .157 | .341 | .310 | .302 | .311 | .332 | .308 |
|  | [-.321, 1.009] | [-.351, .264] | [-.937, .398] | [-.450, .766] | [-.978, .206] | [-.768, .452] | [-.642, .657] | [-.918, .291] |
|  | (.311) | (.781) | (.428) | (.611) | (.201) | (.612) | (.982) | (.309) |
| District of Columbia | .161 | .087 | .190 | .343 | -.089 | .029 | -.219 | .394 |
|  | .423 | .194 | .447 | .388 | .375 | .389 | .414 | .383 |
|  | [-.669, .990] | [-.293, .468] | [-.687, 1.066] | [-.417, 1.103] | [-.824, .646] | [-.734, .791] | [-1.032, .593] | [-.357, 1.145] |
|  | (.704) | (.653) | (.671) | (.377) | (.813) | (.941) | (.596) | (.304) |
| Florida | .173 | .068 | .066 | .127 | -.151 | .005 | .015 | .116 |
|  | .190 | .088 | .192 | .174 | .169 | .175 | .186 | .173 |
|  | [-.200, .546] | [-.103, .240] | [-.310, .443] | [-.215, .468] | [-.482, .181] | [-.338, .347] | [-.350, .381] | [-.222, .454] |
|  | (.364) | (.435) | (.730) | (.468) | (.373) | (.979) | (.934) | (.501) |
| Georgia | .047 | .065 | .158 | -.015 | -.173 | .077 | -.162 | .015 |
|  | .206 | .095 | .208 | .189 | .183 | .189 | .202 | .187 |
|  | [-.358, .451] | [-.121, .251] | [-.249, .566] | [-.385, .355] | [-.532, .185] | [-.295, .448] | [-.557, .234] | [-.351, .382] |
|  | (.821) | (.492) | (.446) | (.936) | (.343) | (.686) | (.423) | (.934) |
| Hawaii | .023 | .301 | .555 | -.010 | -.407 | .131 | -.127 | .383 |
|  | .365 | .169 | .366 | .334 | .325 | .335 | .357 | .332 |
|  | [-.692, .739] | [-.030, .631] | [-.163, 1.272] | [-.664, .645] | [-1.044, .230] | [-.526, .787] | [-.827, .572] | [-.267, 1.033] |
|  | (.949) | (.074) | (.130) | (.977) | (.210) | (.697) | (.721) | (.248) |
| Idaho | .129 | -.114 | -.165 | -.365 | -.513 | -.344 | -.189 | -.022 |
|  | .306 | .141 | .307 | .280 | .272 | .281 | .299 | .278 |
|  | [-.470, .728] | [-.391, .163] | [-.768, .437] | [-.913, .183] | [-1.046, .021] | [-.894, .206] | [-.774, .397] | [-.567, .523] |
|  | (.673) | (.421) | (.591) | (.192) | (.060) | (.220) | (.527) | (.937) |
| Illinois | .028 | .107 | .093 | .274 | -.178 | -.117 | -.053 | .009 |
|  | .207 | .095 | .209 | .190 | .184 | .190 | .203 | .188 |
|  | [-.378, .435] | [-.080, .294] | [-.317, .502] | [-.098, .647] | [-.539, .183] | [-.490, .257] | [-.450, .345] | [-.360, .377] |
|  | (.891) | (.263) | (.657) | (.149) | (.333) | (.541) | (.795) | (.962) |
| Indiana | -.181 | .071 | .332 | .302 | -.035 | -.017 | -.063 | .038 |
|  | .225 | .103 | .226 | .206 | .199 | .206 | .220 | .203 |
|  | [-.621, .259] | [-.131, .274] | [-.111, .774] | [-.101, .705] | [-.425, .356] | [-.422, .387] | [-.494, .367] | [-.361, .436] |
|  | (.419) | (.489) | (.142) | (.142) | (.862) | (.933) | (.773) | (.854) |
| Iowa | .070 | -.033 | -.089 | .427 | -.070 | -.054 | .061 | -.021 |
|  | .267 | .122 | .267 | .244 | .236 | .245 | .261 | .241 |
|  | [-.453, .593] | [-.273, .207] | [-.613, .435] | [-.052, .906] | [-.533, .393] | [-.534, .427] | [-.451, .573] | [-.494, .452] |
|  | (.792) | (.788) | (.739) | (.081) | (.767) | (.826) | (.816) | (.930) |
| Kansas | .088 | .128 | .167 | .248 | -.029 | .177 | .324 | .077 |
|  | .323 | .148 | .323 | .296 | .287 | .297 | .316 | .293 |
|  | [-.545, .721] | [-.163, .419] | [-.466, .801] | [-.332, .828] | [-.591, .533] | [-.404, .759] | [-.295, .943] | [-.497, .651] |
|  | (.784) | (.390) | (.605) | (.401) | (.920) | (.550) | (.305) | (.792) |
| Kentucky | -.217 | -.037 | -.022 | .117 | -.217 | -.006 | -.103 | -.056 |
|  | .244 | .112 | .246 | .224 | .217 | .224 | .239 | .221 |
|  | [-.696, .262] | [-.257, .183] | [-.505, .461] | [-.321, .555] | [-.642, .207] | [-.445, .434] | [-.572, .365] | [-.489, .378] |
|  | (.374) | (.743) | (.930) | (.601) | (.316) | (.980) | (.666) | (.802) |
| Louisiana | .476 | .055 | .015 | -.051 | -.295 | .034 | -.001 | .097 |
|  | .245 | .113 | .246 | .224 | .218 | .225 | .240 | .222 |
|  | [-.005, .956] | [-.166, .277] | [-.468, .499] | [-.491, .388] | [-.722, .132] | [-.407, .475] | [-.471, .469] | [-.339, .533] |
|  | (.052) | (.624) | (.950) | (.819) | (.176) | (.881) | (.997) | (.663) |
| Maine | -.316 | .123 | -.387 | .167 | -.155 | -.338 | -.511 | -.785 |
|  | .340 | .157 | .341 | .311 | .302 | .312 | .332 | .308 |
|  | [-.982, .350] | [-.184, .431] | [-1.055, .281] | [-.443, .776] | [-.747, .437] | [-.949, .273] | [-1.162, .139] | [-1.389, -.181] |
|  | (.352) | (.431) | (.256) | (.592) | (.607) | (.278) | (.123) | (.011) |
| Maryland | -.082 | .138 | -.097 | .114 | .062 | -.036 | .042 | -.024 |
|  | .243 | .112 | .244 | .222 | .215 | .223 | .237 | .220 |
|  | [-.558, .393] | [-.081, .357] | [-.575, .380] | [-.322, .550] | [-.360, .484] | [-.473, .400] | [-.423, .507] | [-.455, .407] |
|  | (.734) | (.216) | (.690) | (.608) | (.772) | (.870) | (.860) | (.914) |
| Massachusetts | -.009 | .069 | .129 | .359 | .166 | .113 | .098 | .183 |
|  | .229 | .105 | .230 | .210 | .203 | .210 | .224 | .208 |
|  | [-.458, .439] | [-.137, .276] | [-.322, .581] | [-.052, .770] | [-.232, .564] | [-.299, .525] | [-.341, .537] | [-.224, .590] |
|  | (.967) | (.511) | (.574) | (.087) | (.414) | (.591) | (.661) | (.377) |
| Michigan | -.021 | .093 | .093 | .176 | -.173 | -.076 | -.116 | -.087 |
|  | .205 | .094 | .206 | .187 | .182 | .188 | .200 | .185 |
|  | [-.423, .380] | [-.091, .278] | [-.312, .498] | [-.192, .543] | [-.529, .183] | [-.445, .292] | [-.509, .276] | [-.451, .276] |
|  | (.917) | (.322) | (.653) | (.349) | (.341) | (.685) | (.561) | (.639) |
| Minnesota | .072 | .041 | .516 | .137 | -.183 | .027 | -.025 | -.069 |
|  | .240 | .110 | .241 | .220 | .213 | .220 | .235 | .217 |
|  | [-.399, .542] | [-.175, .257] | [.044, .988] | [-.294, .567] | [-.601, .234] | [-.405, .459] | [-.485, .435] | [-.495, .357] |
|  | (.766) | (.708) | (.032) | (.534) | (.388) | (.902) | (.915) | (.751) |
| Mississippi | .409 | -.073 | -.029 | .248 | -.192 | -.147 | -.406 | -.021 |
|  | .271 | .125 | .272 | .249 | .241 | .249 | .266 | .246 |
|  | [-.123, .942] | [-.317, .171] | [-.562, .504] | [-.239, .735] | [-.664, .279] | [-.635, .342] | [-.926, .115] | [-.503, .461] |
|  | (.132) | (.559) | (.915) | (.319) | (.424) | (.556) | (.127) | (.931) |
| Missouri | -.122 | .046 | -.065 | -.025 | -.177 | -.081 | -.082 | -.045 |
|  | .220 | .101 | .221 | .201 | .195 | .202 | .215 | .199 |
|  | [-.553, .309] | [-.152, .244] | [-.498, .369] | [-.419, .370] | [-.559, .205] | [-.476, .315] | [-.504, .339] | [-.435, .346] |
|  | (.579) | (.650) | (.770) | (.902) | (.364) | (.690) | (.702) | (.823) |
| Montana | -.208 | .056 | -.072 | .450 | -.241 | .203 | -.145 | .054 |
|  | .339 | .156 | .340 | .310 | .301 | .311 | .331 | .308 |
|  | [-.872, .457] | [-.251, .362] | [-.738, .594] | [-.158, 1.058] | [-.832, .349] | [-.407, .813] | [-.795, .504] | [-.549, .657] |
|  | (.540) | (.722) | (.831) | (.147) | (.423) | (.514) | (.661) | (.861) |
| Nebraska | .068 | -.086 | .313 | .268 | -.212 | -.059 | .322 | .086 |
|  | .349 | .165 | .349 | .320 | .309 | .321 | .342 | .316 |
|  | [-.616, .752] | [-.409, .238] | [-.371, .997] | [-.359, .895] | [-.818, .394] | [-.687, .570] | [-.347, .992] | [-.533, .705] |
|  | (.846) | (.604) | (.370) | (.402) | (.493) | (.855) | (.345) | (.786) |
| Nevada | .097 | .033 | .202 | .258 | .112 | -.033 | -.102 | .080 |
|  | .239 | .111 | .241 | .219 | .213 | .220 | .234 | .217 |
|  | [-.372, .566] | [-.185, .250] | [-.270, .674] | [-.171, .687] | [-.305, .529] | [-.463, .397] | [-.560, .357] | [-.346, .506] |
|  | (.685) | (.767) | (.402) | (.238) | (.600) | (.881) | (.663) | (.714) |
| New Hampshire | .293 | -.029 | .065 | .086 | -.108 | -.317 | -.182 | .079 |
|  | .287 | .133 | .288 | .263 | .255 | .264 | .281 | .261 |
|  | [-.270, .855] | [-.289, .231] | [-.500, .631] | [-.429, .601] | [-.609, .393] | [-.833, .200] | [-.732, .369] | [-.432, .590] |
|  | (.308) | (.827) | (.820) | (.745) | (.672) | (.229) | (.518) | (.761) |
| New Jersey | .299 | .090 | -.150 | .282 | .039 | .108 | .034 | .226 |
|  | .222 | .102 | .223 | .203 | .197 | .204 | .217 | .201 |
|  | [-.136, .735] | [-.110, .289] | [-.588, .288] | [-.117, .681] | [-.346, .425] | [-.292, .508] | [-.392, .460] | [-.168, .620] |
|  | (.178) | (.379) | (.502) | (.166) | (.841) | (.597) | (.876) | (.261) |
| New Mexico | .264 | .074 | -.196 | .118 | -.188 | -.080 | .169 | -.022 |
|  | .264 | .122 | .266 | .242 | .235 | .243 | .259 | .240 |
|  | [-.255, .782] | [-.164, .313] | [-.717, .325] | [-.356, .593] | [-.648, .273] | [-.556, .395] | [-.338, .676] | [-.492, .448] |
|  | (.319) | (.542) | (.460) | (.625) | (.424) | (.741) | (.513) | (.926) |
| New York | .216 | .071 | -.137 | .342 | .047 | -.013 | -.003 | .180 |
|  | .194 | .089 | .196 | .178 | .172 | .178 | .190 | .176 |
|  | [-.165, .596] | [-.104, .246] | [-.520, .247] | [-.006, .691] | [-.290, .385] | [-.363, .336] | [-.376, .369] | [-.165, .525] |
|  | (.267) | (.428) | (.485) | (.054) | (.784) | (.940) | (.985) | (.307) |
| North Carolina | -.137 | .104 | .044 | .244 | -.082 | .042 | .147 | .131 |
|  | .209 | .096 | .211 | .192 | .186 | .192 | .205 | .190 |
|  | [-.548, .273] | [-.085, .293] | [-.369, .457] | [-.132, .620] | [-.446, .282] | [-.335, .419] | [-.254, .549] | [-.241, .503] |
|  | (.512) | (.280) | (.834) | (.203) | (.659) | (.825) | (.472) | (.491) |
| North Dakota | .096 | .151 | .176 | .060 | -.404 | .285 | -.345 | -.168 |
|  | .425 | .196 | .426 | .389 | .378 | .390 | .416 | .386 |
|  | [-.738, .930] | [-.233, .536] | [-.659, 1.012] | [-.703, .823] | [-1.145, .338] | [-.481, 1.050] | [-1.160, .470] | [-.925, .589] |
|  | (.822) | (.441) | (.679) | (.877) | (.286) | (.466) | (.407) | (.663) |
| Ohio | .019 | .083 | .042 | .156 | -.072 | -.032 | -.250 | -.073 |
|  | .204 | .094 | .205 | .186 | .181 | .187 | .199 | .185 |
|  | [-.381, .418] | [-.101, .266] | [-.360, .443] | [-.209, .522] | [-.426, .282] | [-.398, .335] | [-.641, .140] | [-.435, .288] |
|  | (.927) | (.378) | (.839) | (.403) | (.690) | (.866) | (.209) | (.691) |
| Oklahoma | -.070 | .026 | .341 | .033 | -.038 | -.038 | -.116 | -.126 |
|  | .273 | .125 | .274 | .250 | .242 | .251 | .267 | .247 |
|  | [-.605, .465] | [-.219, .272] | [-.195, .877] | [-.458, .523] | [-.513, .436] | [-.530, .453] | [-.639, .408] | [-.611, .359] |
|  | (.798) | (.834) | (.212) | (.897) | (.874) | (.878) | (.665) | (.610) |
| Oregon | -.177 | .097 | .386 | .136 | -.146 | .132 | -.063 | .142 |
|  | .225 | .104 | .227 | .206 | .201 | .207 | .220 | .205 |
|  | [-.619, .265] | [-.107, .301] | [-.059, .830] | [-.269, .540] | [-.539, .247] | [-.273, .538] | [-.495, .369] | [-.260, .543] |
|  | (.433) | (.351) | (.089) | (.511) | (.466) | (.522) | (.774) | (.489) |
| Pennsylvania | .013 | .094 | .138 | .130 | -.193 | -.047 | -.031 | .009 |
|  | .193 | .089 | .194 | .177 | .171 | .177 | .189 | .175 |
|  | [-.365, .392] | [-.080, .267] | [-.243, .519] | [-.217, .476] | [-.528, .142] | [-.395, .300] | [-.401, .339] | [-.334, .351] |
|  | (.944) | (.291) | (.478) | (.463) | (.259) | (.790) | (.870) | (.961) |
| Rhode Island | .057 | .156 | -.186 | .474 | .042 | .154 | -.013 | -.006 |
|  | .423 | .194 | .422 | .387 | .374 | .388 | .414 | .383 |
|  | [-.772, .886] | [-.224, .536] | [-1.013, .642] | [-.286, 1.233] | [-.692, .775] | [-.607, .916] | [-.824, .798] | [-.755, .744] |
|  | (.893) | (.422) | (.660) | (.221) | (.912) | (.691) | (.975) | (.988) |
| South Carolina | .062 | .044 | -.017 | .068 | -.130 | .147 | .024 | .007 |
|  | .236 | .109 | .238 | .216 | .210 | .217 | .231 | .215 |
|  | [-.401, .526] | [-.170, .257] | [-.483, .449] | [-.356, .492] | [-.542, .282] | [-.278, .572] | [-.429, .477] | [-.414, .427] |
|  | (.792) | (.689) | (.942) | (.752) | (.537) | (.499) | (.918) | (.975) |
| South Dakota | .209 | .078 | .279 | -.687 | -.721 | -.088 | -.038 | -.366 |
|  | .391 | .180 | .392 | .358 | .347 | .359 | .383 | .355 |
|  | [-.557, .976] | [-.275, .431] | [-.489, 1.046] | [-1.389, .015] | [-1.402, -.040] | [-.792, .616] | [-.788, .712] | [-1.061, .330] |
|  | (.592) | (.666) | (.477) | (.055) | (.038) | (.807) | (.921) | (.303) |
| Tennessee | .077 | .112 | .148 | .345 | .144 | .163 | -.179 | .075 |
|  | .231 | .106 | .232 | .212 | .205 | .212 | .226 | .209 |
|  | [-.376, .530] | [-.097, .320] | [-.307, .603] | [-.070, .759] | [-.258, .546] | [-.252, .579] | [-.622, .264] | [-.335, .486] |
|  | (.740) | (.294) | (.523) | (.103) | (.482) | (.441) | (.428) | (.719) |
| Texas | -.199 | .084 | .049 | .080 | -.257 | -.019 | .012 | .103 |
|  | .193 | .089 | .195 | .177 | .171 | .177 | .189 | .175 |
|  | [-.578, .179] | [-.090, .258] | [-.332, .431] | [-.266, .427] | [-.593, .078] | [-.366, .329] | [-.358, .382] | [-.240, .446] |
|  | (.302) | (.343) | (.800) | (.649) | (.133) | (.916) | (.950) | (.557) |
| Utah | -.107 | .055 | .399 | .317 | -.091 | -.192 | .162 | .120 |
|  | .268 | .124 | .269 | .245 | .238 | .246 | .262 | .243 |
|  | [-.632, .418] | [-.187, .297] | [-.128, .927] | [-.163, .797] | [-.557, .376] | [-.673, .290] | [-.351, .675] | [-.356, .597] |
|  | (.689) | (.657) | (.138) | (.196) | (.704) | (.435) | (.535) | (.620) |
| Vermont | .616 | .078 | -.043 | -.083 | -.452 | -.165 | .147 | -.662 |
|  | .387 | .177 | .387 | .354 | .343 | .355 | .379 | .350 |
|  | [-.142, 1.374] | [-.270, .426] | [-.801, .715] | [-.778, .611] | [-1.123, .219] | [-.862, .531] | [-.595, .889] | [-1.348, .024] |
|  | (.111) | (.660) | (.911) | (.814) | (.187) | (.641) | (.698) | (.059) |
| Virginia | .129 | .115 | .118 | .098 | -.174 | -.136 | -.249 | .050 |
|  | .207 | .095 | .209 | .189 | .184 | .190 | .202 | .188 |
|  | [-.277, .535] | [-.072, .302] | [-.291, .527] | [-.274, .469] | [-.534, .187] | [-.508, .237] | [-.646, .148] | [-.319, .418] |
|  | (.533) | (.228) | (.571) | (.606) | (.345) | (.475) | (.219) | (.791) |
| Washington | .373 | .083 | .271 | .266 | -.002 | .034 | -.002 | -.031 |
|  | .217 | .100 | .218 | .199 | .193 | .199 | .212 | .197 |
|  | [-.052, .799] | [-.113, .279] | [-.157, .699] | [-.124, .655] | [-.380, .376] | [-.356, .425] | [-.418, .414] | [-.417, .354] |
|  | (.085) | (.408) | (.214) | (.181) | (.992) | (.864) | (.993) | (.873) |
| West Virginia | -.227 | .131 | .171 | .211 | .027 | .024 | -.041 | .157 |
|  | .276 | .127 | .277 | .253 | .245 | .254 | .270 | .250 |
|  | [-.768, .314] | [-.117, .380] | [-.371, .714] | [-.284, .707] | [-.453, .507] | [-.473, .521] | [-.571, .488] | [-.333, .647] |
|  | (.411) | (.300) | (.536) | (.403) | (.912) | (.924) | (.879) | (.529) |
| Wisconsin | .073 | .079 | -.009 | .073 | -.384 | -.183 | -.268 | -.176 |
|  | .224 | .103 | .226 | .205 | .199 | .206 | .219 | .203 |
|  | [-.367, .513] | [-.123, .282] | [-.451, .433] | [-.329, .476] | [-.774, .006] | [-.587, .221] | [-.698, .162] | [-.575, .222] |
|  | (.745) | (.442) | (.967) | (.721) | (.054) | (.374) | (.222) | (.386) |
| Wyoming | .604 | .129 | -.627 | -.368 | -.267 | .038 | -.016 | -.012 |
|  | .654 | .305 | .658 | .597 | .586 | .599 | .637 | .597 |
|  | [-.677, 1.885] | [-.469, .728] | [-1.917, .663] | [-1.537, .801] | [-1.415, .881] | [-1.135, 1.212] | [-1.265, 1.233] | [-1.183, 1.158] |
|  | (.355) | (.672) | (.341) | (.538) | (.648) | (.949) | (.980) | (.983) |
| N | 2982 | 2980 | 2968 | 2980 | 2981 | 2981 | 2981 | 2979 |
| Pseudo-R^2^ |  |  |  |  |  |  |  |  |
| Model χ^2^ | 201.2 | 305.7 | 447.7 | 279.7 | 275.4 | 212.9 | 227.4 | 295.5 |
| d.f. | 88 | 88 | 88 | 88 | 88 | 88 | 88 | 88 |
| p-value | <.001 | <.001 | <.001 | <.001 | <.001 | <.001 | <.001 | <.001 |

S12 Table. Regression Results, Health Attitudes, Multilevel Models, Controlling for Positive Diagnoses.

|  | (1) | (2) | (3) | (4) | (5) | (6) | (7) | (8) |
| --- | --- | --- | --- | --- | --- | --- | --- | --- |
|  | numberdeaths | spendingincrease | notenoughtests | getsick | friendsick | cannotreturn | schoolsclosed | hardgetitems |
| main |  |  |  |  |  |  |  |  |
| Democrat | .357 | .331 | .869 | .544 | .511 | .438 | .271 | .308 |
|  | .053 | .024 | .053 | .048 | .047 | .049 | .052 | .048 |
|  | [.253, .461] | [.284, .379] | [.766, .973] | [.449, .639] | [.419, .602] | [.343, .533] | [.169, .372] | [.214, .401] |
|  | (<.001) | (<.001) | (<.001) | (<.001) | (<.001) | (<.001) | (<.001) | (<.001) |
| Other | .099 | .099 | .503 | .192 | .131 | .096 | .064 | .057 |
|  | .054 | .025 | .054 | .049 | .047 | .049 | .052 | .049 |
|  | [-.006, .204] | [.051, .147] | [.399, .608] | [.095, .288] | [.038, .224] | [-.001, .192] | [-.039, .166] | [-.038, .153] |
|  | (.065) | (<.001) | (<.001) | (<.001) | (.006) | (.052) | (.226) | (.237) |
| Local COVID-19 Cases | <.001 | -<.001 | -<.001 | <.001 | <.001 | <.001 | <.001 | <.001 |
|  | <.001 | <.001 | <.001 | <.001 | <.001 | <.001 | <.001 | <.001 |
|  | [-<.001, .000] | [-<.001, .000] | [-<.001, -<.001] | [-<.001, .000] | [-<.001, .000] | [-<.001, .000] | [-<.001, .000] | [-<.001, .000] |
|  | (.588) | (.839) | (.028) | (.222) | (.308) | (.169) | (.958) | (.088) |
| 35- | -.036 | .041 | .069 | .063 | .069 | -.136 | .037 | .014 |
|  | .059 | .027 | .059 | .054 | .053 | .055 | .058 | .054 |
|  | [-.153, .080] | [-.013, .094] | [-.048, .185] | [-.044, .170] | [-.034, .172] | [-.243, -.029] | [-.077, .151] | [-.091, .119] |
|  | (.543) | (.135) | (.248) | (.249) | (.189) | (.012) | (.521) | (.792) |
| 50- | .154 | .030 | .133 | -.049 | -.016 | -.214 | -.102 | -.189 |
|  | .056 | .026 | .056 | .051 | .050 | .052 | .055 | .051 |
|  | [.044, .264] | [-.020, .081] | [.023, .243] | [-.150, .051] | [-.113, .081] | [-.315, -.113] | [-.210, .005] | [-.289, -.090] |
|  | (.006) | (.237) | (.018) | (.338) | (.746) | (<.001) | (.062) | (<.001) |
| 70- | .336 | .075 | .124 | -.003 | -.008 | -.285 | -.543 | -.406 |
|  | .071 | .033 | .071 | .065 | .063 | .065 | .069 | .064 |
|  | [.197, .476] | [.012, .139] | [-.014, .263] | [-.130, .125] | [-.131, .115] | [-.413, -.157] | [-.679, -.407] | [-.532, -.280] |
|  | (<.001) | (.021) | (.079) | (.967) | (.897) | (<.001) | (<.001) | (<.001) |
| gender | .047 | -.026 | .011 | .145 | .134 | .028 | .047 | .144 |
|  | .041 | .019 | .041 | .037 | .036 | .038 | .040 | .037 |
|  | [-.033, .127] | [-.063, .010] | [-.069, .091] | [.071, .218] | [.063, .205] | [-.045, .102] | [-.032, .125] | [.071, .216] |
|  | (.249) | (.160) | (.788) | (<.001) | (<.001) | (.448) | (.243) | (<.001) |
| white | -.039 | .069 | .241 | .032 | .008 | .137 | -.076 | .030 |
|  | .049 | .022 | .049 | .045 | .043 | .045 | .048 | .044 |
|  | [-.134, .057] | [.025, .113] | [.145, .336] | [-.055, .120] | [-.077, .093] | [.049, .225] | [-.170, .018] | [-.057, .117] |
|  | (.427) | (.002) | (<.001) | (.471) | (.854) | (.002) | (.112) | (.499) |
| married | .006 | -.015 | -.120 | .102 | .082 | .022 | .068 | .102 |
|  | .045 | .021 | .045 | .041 | .040 | .041 | .044 | .041 |
|  | [-.082, .094] | [-.055, .025] | [-.208, -.033] | [.021, .183] | [.004, .160] | [-.059, .103] | [-.018, .154] | [.022, .182] |
|  | (.889) | (.466) | (.007) | (.013) | (.039) | (.599) | (.121) | (.012) |
| High school graduate | -.040 | -.057 | -.014 | -.066 | -.119 | -.074 | .014 | -.217 |
|  | .120 | .055 | .120 | .110 | .106 | .110 | .117 | .108 |
|  | [-.274, .195] | [-.165, .050] | [-.249, .221] | [-.281, .149] | [-.327, .089] | [-.289, .142] | [-.215, .244] | [-.429, -.005] |
|  | (.740) | (.296) | (.907) | (.548) | (.261) | (.504) | (.903) | (.045) |
| Some college | -.075 | .005 | .231 | -.018 | -.100 | -.067 | -.019 | -.314 |
|  | .122 | .056 | .122 | .112 | .108 | .112 | .119 | .110 |
|  | [-.314, .164] | [-.105, .115] | [-.009, .470] | [-.238, .201] | [-.312, .111] | [-.287, .152] | [-.253, .215] | [-.530, -.097] |
|  | (.540) | (.930) | (.059) | (.870) | (.353) | (.548) | (.875) | (.004) |
| 2-year | -.009 | .019 | .154 | -.014 | -.088 | -.105 | -.025 | -.349 |
|  | .128 | .059 | .128 | .118 | .114 | .118 | .126 | .116 |
|  | [-.260, .242] | [-.096, .134] | [-.098, .406] | [-.244, .216] | [-.311, .134] | [-.336, .126] | [-.271, .221] | [-.576, -.121] |
|  | (.944) | (.742) | (.231) | (.906) | (.437) | (.375) | (.842) | (.003) |
| 4-year | -.057 | .026 | .153 | -.053 | -.061 | -.045 | -.059 | -.315 |
|  | .125 | .057 | .125 | .114 | .110 | .114 | .122 | .113 |
|  | [-.301, .187] | [-.085, .138] | [-.091, .398] | [-.276, .171] | [-.278, .155] | [-.270, .179] | [-.298, .180] | [-.536, -.094] |
|  | (.648) | (.643) | (.220) | (.644) | (.578) | (.691) | (.627) | (.005) |
| Post-grad | -.052 | .046 | .204 | .053 | .009 | -<.001 | .003 | -.409 |
|  | .131 | .060 | .131 | .120 | .116 | .120 | .128 | .118 |
|  | [-.309, .204] | [-.072, .163] | [-.053, .461] | [-.182, .288] | [-.218, .236] | [-.236, .236] | [-.248, .254] | [-.641, -.177] |
|  | (.689) | (.445) | (.120) | (.659) | (.935) | (.999) | (.980) | (.001) |
| income=2 | .065 | -.041 | .358 | -.071 | .001 | -.158 | -.154 | -.112 |
|  | .115 | .053 | .116 | .106 | .102 | .106 | .113 | .104 |
|  | [-.162, .291] | [-.144, .063] | [.131, .586] | [-.279, .136] | [-.199, .202] | [-.366, .050] | [-.375, .068] | [-.316, .093] |
|  | (.575) | (.441) | (.002) | (.501) | (.990) | (.136) | (.173) | (.285) |
| income=3 | .172 | -.005 | .302 | -.092 | -.011 | -.109 | -.042 | -.145 |
|  | .112 | .051 | .112 | .103 | .099 | .103 | .109 | .101 |
|  | [-.047, .391] | [-.105, .095] | [.082, .523] | [-.293, .109] | [-.204, .183] | [-.310, .093] | [-.257, .172] | [-.343, .053] |
|  | (.123) | (.923) | (.007) | (.371) | (.914) | (.289) | (.701) | (.151) |
| income=4 | .124 | .024 | .280 | .016 | .094 | -.081 | -.047 | -.114 |
|  | .115 | .052 | .115 | .105 | .101 | .105 | .112 | .104 |
|  | [-.101, .348] | [-.079, .127] | [.054, .506] | [-.190, .222] | [-.105, .293] | [-.287, .126] | [-.267, .173] | [-.318, .089] |
|  | (.280) | (.645) | (.015) | (.880) | (.353) | (.445) | (.677) | (.270) |
| income=5 | .180 | .028 | .361 | -.057 | .010 | -.158 | -.035 | -.196 |
|  | .117 | .054 | .118 | .107 | .103 | .107 | .114 | .106 |
|  | [-.049, .410] | [-.077, .133] | [.130, .591] | [-.267, .153] | [-.193, .213] | [-.368, .053] | [-.259, .189] | [-.403, .011] |
|  | (.123) | (.606) | (.002) | (.592) | (.924) | (.142) | (.760) | (.064) |
| income=6 | .217 | -.004 | .361 | .015 | .009 | -.088 | .087 | -.145 |
|  | .118 | .054 | .119 | .108 | .105 | .109 | .116 | .107 |
|  | [-.014, .449] | [-.110, .102] | [.129, .593] | [-.198, .227] | [-.196, .213] | [-.301, .125] | [-.140, .314] | [-.355, .065] |
|  | (.066) | (.934) | (.002) | (.892) | (.935) | (.417) | (.452) | (.175) |
| income=7 | .083 | .001 | .385 | -.071 | -.017 | -.176 | -.098 | -.280 |
|  | .127 | .058 | .127 | .116 | .112 | .116 | .124 | .115 |
|  | [-.165, .331] | [-.113, .115] | [.136, .634] | [-.299, .156] | [-.237, .202] | [-.404, .052] | [-.341, .145] | [-.504, -.055] |
|  | (.511) | (.987) | (.002) | (.539) | (.877) | (.129) | (.427) | (.015) |
| income=8 | .118 | .009 | .325 | -.274 | -.228 | -.265 | -.180 | -.369 |
|  | .123 | .056 | .124 | .113 | .109 | .113 | .121 | .111 |
|  | [-.124, .359] | [-.101, .120] | [.082, .568] | [-.495, -.052] | [-.441, -.014] | [-.487, -.043] | [-.416, .057] | [-.587, -.150] |
|  | (.340) | (.867) | (.009) | (.015) | (.037) | (.019) | (.136) | (.001) |
| income=9 | .349 | .009 | .467 | -.188 | -.068 | -.082 | -.108 | -.310 |
|  | .122 | .056 | .122 | .112 | .108 | .112 | .119 | .110 |
|  | [.111, .588] | [-.100, .118] | [.227, .707] | [-.407, .031] | [-.279, .143] | [-.302, .137] | [-.342, .126] | [-.526, -.094] |
|  | (.004) | (.872) | (<.001) | (.093) | (.528) | (.463) | (.365) | (.005) |
| income=10 | .221 | .005 | .491 | -.180 | -.079 | -.102 | -.108 | -.375 |
|  | .127 | .058 | .127 | .116 | .112 | .116 | .124 | .115 |
|  | [-.028, .469] | [-.109, .118] | [.242, .740] | [-.408, .047] | [-.298, .141] | [-.330, .127] | [-.352, .135] | [-.600, -.150] |
|  | (.082) | (.938) | (<.001) | (.121) | (.483) | (.382) | (.382) | (.001) |
| income=11 | .370 | .001 | .466 | -.075 | -.001 | .074 | -.099 | -.354 |
|  | .130 | .060 | .130 | .119 | .115 | .120 | .127 | .118 |
|  | [.115, .625] | [-.116, .118] | [.211, .722] | [-.308, .159] | [-.227, .224] | [-.160, .308] | [-.349, .151] | [-.584, -.123] |
|  | (.004) | (.990) | (<.001) | (.532) | (.990) | (.536) | (.438) | (.003) |
| income=12 | .235 | .034 | .361 | -.138 | .003 | .024 | -.183 | -.418 |
|  | .143 | .066 | .143 | .131 | .127 | .131 | .140 | .129 |
|  | [-.046, .515] | [-.095, .163] | [.080, .642] | [-.395, .119] | [-.245, .252] | [-.233, .282] | [-.457, .092] | [-.672, -.164] |
|  | (.101) | (.607) | (.012) | (.293) | (.978) | (.854) | (.192) | (.001) |
| income=13 | .210 | .063 | .650 | -.264 | -.136 | -.087 | -.420 | -.653 |
|  | .198 | .091 | .198 | .181 | .175 | .182 | .194 | .179 |
|  | [-.178, .598] | [-.115, .241] | [.262, 1.038] | [-.619, .092] | [-.480, .207] | [-.444, .269] | [-.799, -.040] | [-1.004, -.302] |
|  | (.288) | (.488) | (.001) | (.146) | (.437) | (.631) | (.030) | (<.001) |
| income=14 | .304 | -.077 | .525 | -.238 | -.080 | -.195 | -.071 | -.447 |
|  | .210 | .096 | .209 | .192 | .186 | .193 | .205 | .190 |
|  | [-.107, .715] | [-.265, .111] | [.115, .935] | [-.615, .139] | [-.443, .284] | [-.573, .183] | [-.474, .331] | [-.819, -.075] |
|  | (.147) | (.421) | (.012) | (.215) | (.668) | (.311) | (.728) | (.018) |
| income=15 | .278 | .232 | 1.360 | .448 | .236 | .392 | -.068 | -1.131 |
|  | .635 | .290 | .632 | .582 | .562 | .584 | .622 | .574 |
|  | [-.967, 1.523] | [-.337, .802] | [.120, 2.599] | [-.693, 1.589] | [-.865, 1.337] | [-.752, 1.536] | [-1.287, 1.150] | [-2.257, -.006] |
|  | (.661) | (.423) | (.032) | (.441) | (.674) | (.502) | (.912) | (.049) |
| income=16 | .530 | .045 | .866 | -.301 | -.094 | .168 | -.037 | -.487 |
|  | .298 | .137 | .297 | .273 | .264 | .274 | .292 | .270 |
|  | [-.055, 1.114] | [-.223, .312] | [.283, 1.449] | [-.837, .235] | [-.611, .424] | [-.369, .706] | [-.610, .536] | [-1.016, .042] |
|  | (.076) | (.744) | (.004) | (.271) | (.722) | (.540) | (.899) | (.071) |
| income=17 | .272 | -.033 | .340 | -.234 | -.131 | -.227 | -.263 | -.420 |
|  | .108 | .050 | .109 | .099 | .096 | .100 | .106 | .098 |
|  | [.060, .485] | [-.130, .064] | [.127, .554] | [-.429, -.039] | [-.319, .057] | [-.422, -.032] | [-.471, -.055] | [-.612, -.227] |
|  | (.012) | (.508) | (.002) | (.019) | (.171) | (.023) | (.013) | (<.001) |
| Rural Code=2 | .013 | -.017 | -.083 | .010 | -.051 | -.023 | -.099 | .008 |
|  | .056 | .026 | .056 | .051 | .050 | .051 | .055 | .051 |
|  | [-.096, .123] | [-.068, .033] | [-.193, .027] | [-.091, .110] | [-.148, .047] | [-.123, .078] | [-.206, .008] | [-.092, .108] |
|  | (.812) | (.504) | (.137) | (.850) | (.308) | (.657) | (.070) | (.875) |
| Rural Code=3 | -.001 | -.030 | -.139 | .038 | .062 | .037 | .023 | .075 |
|  | .075 | .035 | .075 | .069 | .067 | .069 | .074 | .068 |
|  | [-.148, .147] | [-.098, .039] | [-.286, .009] | [-.097, .173] | [-.069, .193] | [-.098, .173] | [-.122, .167] | [-.059, .209] |
|  | (.993) | (.394) | (.066) | (.583) | (.354) | (.590) | (.758) | (.272) |
| Rural Code=4 | -.074 | .011 | .031 | -.121 | -.161 | -.121 | -.093 | -.023 |
|  | .104 | .048 | .104 | .095 | .092 | .095 | .102 | .094 |
|  | [-.278, .130] | [-.083, .105] | [-.172, .235] | [-.308, .066] | [-.342, .020] | [-.308, .066] | [-.292, .106] | [-.208, .162] |
|  | (.477) | (.817) | (.763) | (.204) | (.081) | (.206) | (.360) | (.805) |
| Rural Code=5 | -.161 | -.001 | -.043 | -.122 | .063 | -.062 | .004 | .019 |
|  | .158 | .073 | .158 | .145 | .141 | .146 | .155 | .144 |
|  | [-.471, .150] | [-.144, .142] | [-.353, .267] | [-.407, .162] | [-.213, .339] | [-.347, .224] | [-.299, .308] | [-.263, .300] |
|  | (.311) | (.993) | (.787) | (.399) | (.654) | (.672) | (.978) | (.897) |
| Rural Code=6 | .133 | -.014 | -.080 | .021 | -.056 | .017 | .011 | .128 |
|  | .103 | .048 | .103 | .095 | .092 | .095 | .101 | .094 |
|  | [-.070, .335] | [-.107, .079] | [-.282, .122] | [-.164, .206] | [-.236, .124] | [-.168, .203] | [-.187, .209] | [-.055, .312] |
|  | (.199) | (.771) | (.439) | (.825) | (.541) | (.854) | (.916) | (.171) |
| Rural Code=7 | .061 | -.046 | .030 | .079 | .072 | .064 | -.033 | .094 |
|  | .142 | .065 | .141 | .130 | .126 | .130 | .138 | .128 |
|  | [-.217, .338] | [-.173, .082] | [-.247, .308] | [-.175, .334] | [-.175, .318] | [-.191, .319] | [-.305, .238] | [-.158, .345] |
|  | (.667) | (.484) | (.829) | (.540) | (.568) | (.622) | (.811) | (.466) |
| Rural Code=8 | .506 | -.024 | -.240 | -.405 | -.199 | -.067 | -.408 | .115 |
|  | .265 | .121 | .271 | .242 | .234 | .243 | .259 | .239 |
|  | [-.013, 1.024] | [-.262, .214] | [-.770, .290] | [-.880, .070] | [-.659, .260] | [-.544, .409] | [-.915, .100] | [-.354, .585] |
|  | (.056) | (.843) | (.375) | (.095) | (.395) | (.782) | (.116) | (.631) |
| Rural Code=9 | -.116 | -.060 | -.039 | .240 | .236 | -.017 | -.081 | -.162 |
|  | .225 | .103 | .224 | .206 | .199 | .206 | .220 | .203 |
|  | [-.556, .324] | [-.262, .142] | [-.478, .400] | [-.164, .643] | [-.154, .626] | [-.421, .387] | [-.512, .349] | [-.561, .237] |
|  | (.604) | (.562) | (.862) | (.244) | (.236) | (.934) | (.711) | (.425) |
| Alaska | .254 | -.022 | .347 | -.013 | -.466 | -.511 | .041 | -.272 |
|  | .449 | .206 | .448 | .411 | .398 | .413 | .440 | .406 |
|  | [-.627, 1.134] | [-.425, .382] | [-.531, 1.225] | [-.819, .794] | [-1.246, .313] | [-1.319, .298] | [-.820, .902] | [-1.069, .524] |
|  | (.572) | (.915) | (.439) | (.975) | (.241) | (.216) | (.926) | (.503) |
| Arizona | -.205 | .064 | .151 | -.034 | -.206 | -.130 | -.175 | .030 |
|  | .214 | .099 | .216 | .196 | .190 | .196 | .209 | .194 |
|  | [-.624, .214] | [-.129, .257] | [-.272, .574] | [-.418, .349] | [-.578, .167] | [-.515, .254] | [-.585, .234] | [-.350, .410] |
|  | (.337) | (.516) | (.484) | (.862) | (.279) | (.507) | (.401) | (.878) |
| Arkansas | .129 | .046 | .001 | .109 | -.026 | .033 | .022 | .268 |
|  | .240 | .111 | .241 | .220 | .213 | .221 | .235 | .218 |
|  | [-.342, .600] | [-.171, .262] | [-.471, .474] | [-.322, .540] | [-.443, .392] | [-.399, .466] | [-.438, .482] | [-.159, .695] |
|  | (.591) | (.679) | (.995) | (.621) | (.904) | (.880) | (.925) | (.219) |
| California | -.017 | .092 | .044 | .169 | -.060 | -.042 | .009 | .115 |
|  | .189 | .087 | .190 | .173 | .168 | .174 | .185 | .171 |
|  | [-.387, .354] | [-.079, .262] | [-.329, .417] | [-.171, .508] | [-.389, .269] | [-.382, .299] | [-.353, .372] | [-.221, .451] |
|  | (.930) | (.292) | (.817) | (.330) | (.721) | (.810) | (.960) | (.502) |
| Colorado | .208 | .010 | -.028 | .172 | -.131 | -.200 | -.062 | -.147 |
|  | .241 | .111 | .244 | .221 | .215 | .222 | .236 | .219 |
|  | [-.265, .681] | [-.208, .228] | [-.506, .450] | [-.261, .605] | [-.552, .289] | [-.634, .235] | [-.524, .401] | [-.577, .283] |
|  | (.389) | (.927) | (.908) | (.437) | (.540) | (.367) | (.794) | (.502) |
| Connecticut | .012 | .275 | .308 | .220 | -.025 | .106 | .012 | .003 |
|  | .263 | .121 | .264 | .241 | .234 | .241 | .257 | .239 |
|  | [-.503, .528] | [.037, .512] | [-.209, .825] | [-.252, .691] | [-.483, .432] | [-.367, .580] | [-.492, .516] | [-.465, .470] |
|  | (.963) | (.023) | (.243) | (.362) | (.914) | (.659) | (.964) | (.990) |
| Delaware | .342 | -.043 | -.262 | .154 | -.389 | -.162 | .007 | -.319 |
|  | .339 | .157 | .340 | .310 | .302 | .311 | .332 | .308 |
|  | [-.323, 1.007] | [-.350, .264] | [-.929, .406] | [-.454, .763] | [-.981, .203] | [-.772, .448] | [-.643, .657] | [-.923, .286] |
|  | (.314) | (.783) | (.442) | (.620) | (.197) | (.603) | (.983) | (.301) |
| District of Columbia | .160 | .087 | .194 | .341 | -.090 | .027 | -.220 | .392 |
|  | .423 | .194 | .447 | .388 | .375 | .389 | .414 | .383 |
|  | [-.670, .989] | [-.293, .468] | [-.682, 1.070] | [-.419, 1.102] | [-.825, .645] | [-.735, .789] | [-1.032, .592] | [-.359, 1.143] |
|  | (.706) | (.652) | (.665) | (.379) | (.810) | (.945) | (.596) | (.307) |
| Florida | .172 | .068 | .072 | .122 | -.155 | -.002 | .016 | .109 |
|  | .190 | .087 | .192 | .174 | .169 | .175 | .186 | .173 |
|  | [-.201, .545] | [-.103, .240] | [-.304, .448] | [-.219, .464] | [-.486, .176] | [-.345, .340] | [-.349, .381] | [-.229, .447] |
|  | (.366) | (.435) | (.707) | (.482) | (.360) | (.989) | (.930) | (.526) |
| Georgia | .047 | .065 | .160 | -.018 | -.176 | .071 | -.161 | .011 |
|  | .206 | .095 | .208 | .189 | .183 | .189 | .202 | .187 |
|  | [-.357, .451] | [-.121, .251] | [-.247, .567] | [-.388, .353] | [-.534, .183] | [-.300, .442] | [-.556, .235] | [-.355, .377] |
|  | (.821) | (.493) | (.441) | (.926) | (.336) | (.707) | (.425) | (.954) |
| Hawaii | .022 | .301 | .558 | -.011 | -.408 | .129 | -.128 | .381 |
|  | .365 | .169 | .366 | .334 | .325 | .335 | .357 | .332 |
|  | [-.693, .738] | [-.029, .632] | [-.159, 1.276] | [-.666, .643] | [-1.045, .228] | [-.527, .786] | [-.827, .572] | [-.269, 1.031] |
|  | (.951) | (.074) | (.127) | (.973) | (.209) | (.699) | (.720) | (.251) |
| Idaho | .128 | -.113 | -.160 | -.367 | -.514 | -.346 | -.189 | -.025 |
|  | .306 | .141 | .307 | .280 | .272 | .281 | .299 | .278 |
|  | [-.472, .727] | [-.391, .164] | [-.762, .442] | [-.916, .181] | [-1.048, .019] | [-.896, .204] | [-.775, .396] | [-.570, .520] |
|  | (.676) | (.423) | (.603) | (.189) | (.059) | (.218) | (.527) | (.929) |
| Illinois | .013 | .111 | .142 | .261 | -.185 | -.113 | -.061 | -.001 |
|  | .210 | .096 | .211 | .192 | .186 | .193 | .205 | .190 |
|  | [-.398, .425] | [-.078, .301] | [-.272, .556] | [-.116, .637] | [-.550, .180] | [-.490, .265] | [-.464, .341] | [-.374, .372] |
|  | (.950) | (.248) | (.501) | (.175) | (.321) | (.559) | (.765) | (.996) |
| Indiana | -.182 | .071 | .333 | .301 | -.035 | -.019 | -.063 | .036 |
|  | .225 | .103 | .226 | .206 | .199 | .206 | .220 | .203 |
|  | [-.622, .258] | [-.131, .274] | [-.109, .775] | [-.102, .704] | [-.426, .355] | [-.423, .385] | [-.494, .367] | [-.363, .435] |
|  | (.418) | (.489) | (.139) | (.143) | (.859) | (.928) | (.773) | (.859) |
| Iowa | .069 | -.033 | -.084 | .425 | -.071 | -.055 | .060 | -.024 |
|  | .267 | .122 | .267 | .244 | .236 | .245 | .261 | .242 |
|  | [-.454, .592] | [-.272, .207] | [-.608, .439] | [-.054, .904] | [-.535, .392] | [-.536, .425] | [-.451, .572] | [-.497, .450] |
|  | (.795) | (.790) | (.753) | (.082) | (.763) | (.822) | (.817) | (.922) |
| Kansas | .089 | .128 | .168 | .247 | -.030 | .174 | .324 | .074 |
|  | .323 | .148 | .323 | .296 | .287 | .297 | .316 | .293 |
|  | [-.544, .722] | [-.163, .419] | [-.465, .802] | [-.333, .826] | [-.592, .531] | [-.408, .755] | [-.295, .944] | [-.499, .648] |
|  | (.784) | (.390) | (.603) | (.404) | (.915) | (.558) | (.304) | (.799) |
| Kentucky | -.217 | -.037 | -.020 | .116 | -.219 | -.008 | -.103 | -.057 |
|  | .244 | .112 | .246 | .224 | .217 | .224 | .239 | .221 |
|  | [-.696, .261] | [-.257, .183] | [-.503, .463] | [-.323, .554] | [-.643, .206] | [-.447, .432] | [-.571, .365] | [-.491, .377] |
|  | (.373) | (.743) | (.935) | (.605) | (.313) | (.973) | (.666) | (.795) |
| Louisiana | .475 | .056 | .018 | -.052 | -.295 | .034 | -.001 | .097 |
|  | .245 | .113 | .246 | .224 | .218 | .225 | .240 | .222 |
|  | [-.006, .955] | [-.166, .277] | [-.464, .501] | [-.492, .388] | [-.722, .132] | [-.407, .475] | [-.471, .468] | [-.340, .533] |
|  | (.053) | (.622) | (.940) | (.817) | (.176) | (.879) | (.995) | (.664) |
| Maine | -.317 | .124 | -.383 | .165 | -.156 | -.340 | -.512 | -.787 |
|  | .340 | .157 | .341 | .311 | .302 | .312 | .332 | .308 |
|  | [-.983, .349] | [-.184, .431] | [-1.051, .284] | [-.444, .774] | [-.748, .436] | [-.951, .272] | [-1.162, .139] | [-1.391, -.182] |
|  | (.351) | (.430) | (.260) | (.595) | (.605) | (.276) | (.123) | (.011) |
| Maryland | -.082 | .138 | -.095 | .111 | .060 | -.042 | .043 | -.028 |
|  | .243 | .112 | .244 | .222 | .215 | .223 | .237 | .220 |
|  | [-.558, .393] | [-.081, .356] | [-.573, .382] | [-.324, .547] | [-.362, .482] | [-.479, .395] | [-.422, .508] | [-.459, .403] |
|  | (.734) | (.217) | (.695) | (.616) | (.782) | (.851) | (.856) | (.898) |
| Massachusetts | -.013 | .071 | .138 | .361 | .170 | .125 | .094 | .191 |
|  | .229 | .105 | .230 | .209 | .203 | .210 | .224 | .207 |
|  | [-.461, .435] | [-.135, .277] | [-.313, .588] | [-.049, .771] | [-.228, .568] | [-.286, .537] | [-.344, .532] | [-.216, .597] |
|  | (.955) | (.500) | (.549) | (.085) | (.403) | (.551) | (.674) | (.358) |
| Michigan | -.019 | .094 | .079 | .190 | -.159 | -.049 | -.120 | -.062 |
|  | .204 | .094 | .205 | .186 | .181 | .187 | .199 | .184 |
|  | [-.418, .380] | [-.089, .277] | [-.324, .481] | [-.176, .555] | [-.513, .195] | [-.416, .317] | [-.510, .270] | [-.424, .299] |
|  | (.924) | (.315) | (.702) | (.309) | (.380) | (.793) | (.546) | (.735) |
| Minnesota | .073 | .041 | .509 | .139 | -.182 | .028 | -.024 | -.067 |
|  | .240 | .110 | .241 | .220 | .213 | .220 | .235 | .217 |
|  | [-.397, .544] | [-.175, .257] | [.038, .981] | [-.292, .570] | [-.599, .235] | [-.404, .460] | [-.484, .436] | [-.493, .360] |
|  | (.760) | (.712) | (.034) | (.527) | (.392) | (.899) | (.918) | (.759) |
| Mississippi | .408 | -.073 | -.025 | .246 | -.194 | -.148 | -.406 | -.024 |
|  | .271 | .125 | .272 | .249 | .241 | .249 | .266 | .246 |
|  | [-.124, .941] | [-.317, .172] | [-.558, .508] | [-.241, .734] | [-.665, .278] | [-.637, .341] | [-.926, .115] | [-.505, .458] |
|  | (.133) | (.560) | (.926) | (.322) | (.421) | (.552) | (.127) | (.924) |
| Missouri | -.122 | .046 | -.064 | -.026 | -.179 | -.084 | -.082 | -.047 |
|  | .220 | .101 | .221 | .201 | .195 | .202 | .215 | .199 |
|  | [-.553, .309] | [-.153, .244] | [-.497, .369] | [-.421, .368] | [-.561, .204] | [-.480, .312] | [-.503, .340] | [-.438, .343] |
|  | (.579) | (.652) | (.773) | (.897) | (.360) | (.678) | (.704) | (.812) |
| Montana | -.209 | .056 | -.067 | .449 | -.242 | .203 | -.146 | .052 |
|  | .339 | .156 | .340 | .310 | .301 | .311 | .331 | .308 |
|  | [-.874, .455] | [-.250, .362] | [-.733, .598] | [-.159, 1.057] | [-.833, .348] | [-.407, .813] | [-.796, .504] | [-.551, .655] |
|  | (.537) | (.720) | (.843) | (.148) | (.421) | (.514) | (.660) | (.865) |
| Nebraska | .066 | -.085 | .321 | .265 | -.214 | -.061 | .322 | .082 |
|  | .349 | .165 | .349 | .320 | .309 | .321 | .342 | .316 |
|  | [-.618, .750] | [-.408, .238] | [-.363, 1.005] | [-.362, .892] | [-.820, .392] | [-.690, .567] | [-.348, .991] | [-.537, .701] |
|  | (.851) | (.606) | (.358) | (.408) | (.488) | (.849) | (.346) | (.796) |
| Nevada | .096 | .033 | .206 | .256 | .110 | -.036 | -.102 | .077 |
|  | .239 | .111 | .241 | .219 | .213 | .220 | .234 | .217 |
|  | [-.373, .565] | [-.184, .250] | [-.266, .678] | [-.173, .685] | [-.307, .527] | [-.466, .395] | [-.560, .357] | [-.349, .503] |
|  | (.688) | (.766) | (.392) | (.242) | (.606) | (.871) | (.663) | (.725) |
| New Hampshire | .292 | -.029 | .069 | .083 | -.110 | -.320 | -.181 | .076 |
|  | .287 | .133 | .288 | .263 | .255 | .264 | .281 | .261 |
|  | [-.271, .855] | [-.289, .231] | [-.496, .634] | [-.432, .598] | [-.611, .391] | [-.837, .197] | [-.731, .369] | [-.435, .587] |
|  | (.309) | (.827) | (.812) | (.751) | (.667) | (.225) | (.518) | (.771) |
| New Jersey | .298 | .091 | -.148 | .286 | .044 | .119 | .031 | .235 |
|  | .222 | .102 | .223 | .203 | .197 | .204 | .217 | .201 |
|  | [-.137, .733] | [-.109, .290] | [-.585, .289] | [-.113, .684] | [-.341, .429] | [-.280, .519] | [-.394, .456] | [-.159, .628] |
|  | (.179) | (.372) | (.507) | (.160) | (.822) | (.558) | (.886) | (.243) |
| New Mexico | .263 | .075 | -.192 | .117 | -.189 | -.082 | .169 | -.025 |
|  | .264 | .122 | .266 | .242 | .235 | .243 | .259 | .240 |
|  | [-.256, .781] | [-.164, .313] | [-.713, .328] | [-.358, .591] | [-.650, .271] | [-.558, .394] | [-.338, .676] | [-.495, .446] |
|  | (.321) | (.540) | (.469) | (.630) | (.421) | (.735) | (.514) | (.919) |
| New York | .212 | .072 | -.123 | .338 | .045 | -.014 | -.005 | .175 |
|  | .195 | .089 | .196 | .178 | .173 | .179 | .190 | .176 |
|  | [-.170, .593] | [-.103, .247] | [-.507, .260] | [-.011, .687] | [-.294, .383] | [-.365, .336] | [-.378, .368] | [-.170, .521] |
|  | (.276) | (.421) | (.528) | (.058) | (.796) | (.936) | (.978) | (.320) |
| North Carolina | -.138 | .104 | .046 | .242 | -.084 | .038 | .148 | .127 |
|  | .209 | .096 | .211 | .192 | .186 | .192 | .205 | .190 |
|  | [-.548, .273] | [-.085, .293] | [-.367, .459] | [-.134, .618] | [-.448, .280] | [-.339, .416] | [-.254, .549] | [-.245, .499] |
|  | (.511) | (.281) | (.827) | (.207) | (.651) | (.841) | (.470) | (.503) |
| North Dakota | .095 | .152 | .181 | .059 | -.405 | .284 | -.345 | -.170 |
|  | .425 | .196 | .426 | .389 | .378 | .391 | .416 | .386 |
|  | [-.739, .928] | [-.233, .536] | [-.654, 1.016] | [-.705, .822] | [-1.146, .337] | [-.482, 1.050] | [-1.160, .470] | [-.927, .588] |
|  | (.824) | (.440) | (.671) | (.880) | (.285) | (.467) | (.407) | (.661) |
| Ohio | .018 | .082 | .043 | .155 | -.073 | -.034 | -.250 | -.075 |
|  | .204 | .094 | .205 | .186 | .181 | .187 | .199 | .185 |
|  | [-.381, .418] | [-.101, .266] | [-.359, .444] | [-.211, .520] | [-.427, .281] | [-.400, .333] | [-.640, .141] | [-.437, .286] |
|  | (.928) | (.379) | (.834) | (.406) | (.686) | (.857) | (.210) | (.683) |
| Oklahoma | -.070 | .026 | .344 | .031 | -.040 | -.041 | -.115 | -.129 |
|  | .273 | .125 | .274 | .250 | .242 | .251 | .267 | .247 |
|  | [-.606, .465] | [-.220, .272] | [-.192, .880] | [-.459, .521] | [-.515, .434] | [-.533, .450] | [-.639, .408] | [-.614, .356] |
|  | (.796) | (.834) | (.209) | (.902) | (.868) | (.869) | (.666) | (.602) |
| Oregon | -.176 | .097 | .386 | .134 | -.148 | .128 | -.062 | .138 |
|  | .225 | .104 | .227 | .206 | .200 | .207 | .220 | .205 |
|  | [-.618, .266] | [-.107, .301] | [-.059, .830] | [-.270, .538] | [-.541, .245] | [-.278, .533] | [-.494, .370] | [-.263, .539] |
|  | (.434) | (.353) | (.089) | (.516) | (.460) | (.537) | (.777) | (.500) |
| Pennsylvania | .012 | .094 | .144 | .128 | -.194 | -.048 | -.032 | .006 |
|  | .193 | .089 | .194 | .177 | .171 | .177 | .189 | .175 |
|  | [-.367, .390] | [-.080, .268] | [-.236, .525] | [-.219, .474] | [-.530, .141] | [-.396, .299] | [-.402, .338] | [-.337, .349] |
|  | (.952) | (.289) | (.458) | (.470) | (.256) | (.786) | (.867) | (.971) |
| Rhode Island | .055 | .155 | -.175 | .465 | .033 | .140 | -.012 | -.020 |
|  | .423 | .194 | .422 | .387 | .374 | .388 | .414 | .382 |
|  | [-.773, .883] | [-.224, .535] | [-1.002, .652] | [-.294, 1.224] | [-.700, .767] | [-.621, .901] | [-.822, .799] | [-.769, .730] |
|  | (.897) | (.423) | (.679) | (.230) | (.929) | (.719) | (.978) | (.959) |
| South Carolina | .062 | .044 | -.014 | .066 | -.131 | .144 | .024 | .004 |
|  | .236 | .109 | .238 | .216 | .210 | .217 | .231 | .215 |
|  | [-.402, .525] | [-.170, .257] | [-.480, .451] | [-.358, .490] | [-.543, .280] | [-.281, .569] | [-.429, .477] | [-.417, .424] |
|  | (.794) | (.689) | (.952) | (.759) | (.532) | (.507) | (.918) | (.985) |
| South Dakota | .208 | .078 | .284 | -.689 | -.722 | -.089 | -.038 | -.368 |
|  | .391 | .180 | .392 | .358 | .347 | .359 | .383 | .355 |
|  | [-.559, .975] | [-.275, .431] | [-.483, 1.051] | [-1.391, .013] | [-1.404, -.041] | [-.793, .615] | [-.788, .711] | [-1.064, .327] |
|  | (.595) | (.665) | (.468) | (.054) | (.038) | (.804) | (.920) | (.300) |
| Tennessee | .076 | .112 | .151 | .342 | .142 | .160 | -.179 | .072 |
|  | .231 | .106 | .232 | .212 | .205 | .212 | .226 | .209 |
|  | [-.377, .529] | [-.097, .320] | [-.304, .606] | [-.072, .757] | [-.260, .544] | [-.256, .576] | [-.622, .264] | [-.339, .482] |
|  | (.742) | (.294) | (.514) | (.105) | (.488) | (.452) | (.429) | (.732) |
| Texas | -.201 | .084 | .057 | .075 | -.263 | -.028 | .013 | .094 |
|  | .193 | .089 | .194 | .177 | .171 | .177 | .189 | .175 |
|  | [-.579, .178] | [-.090, .258] | [-.324, .438] | [-.271, .421] | [-.598, .073] | [-.375, .320] | [-.357, .383] | [-.249, .437] |
|  | (.298) | (.343) | (.770) | (.671) | (.125) | (.876) | (.946) | (.591) |
| Utah | -.109 | .055 | .407 | .312 | -.095 | -.198 | .163 | .114 |
|  | .268 | .124 | .269 | .245 | .238 | .246 | .262 | .243 |
|  | [-.633, .416] | [-.187, .297] | [-.121, .934] | [-.168, .792] | [-.561, .372] | [-.680, .283] | [-.350, .675] | [-.363, .590] |
|  | (.685) | (.656) | (.131) | (.202) | (.691) | (.420) | (.534) | (.640) |
| Vermont | .614 | .078 | -.039 | -.084 | -.453 | -.165 | .146 | -.663 |
|  | .387 | .177 | .386 | .354 | .343 | .355 | .379 | .350 |
|  | [-.144, 1.372] | [-.269, .426] | [-.796, .719] | [-.779, .610] | [-1.124, .219] | [-.861, .532] | [-.596, .888] | [-1.349, .024] |
|  | (.112) | (.658) | (.920) | (.812) | (.187) | (.643) | (.699) | (.058) |
| Virginia | .130 | .114 | .118 | .096 | -.176 | -.142 | -.248 | .046 |
|  | .207 | .095 | .208 | .189 | .184 | .190 | .202 | .188 |
|  | [-.276, .535] | [-.072, .301] | [-.291, .526] | [-.275, .467] | [-.536, .184] | [-.514, .231] | [-.644, .149] | [-.323, .414] |
|  | (.531) | (.230) | (.573) | (.613) | (.338) | (.456) | (.221) | (.808) |
| Washington | .373 | .083 | .272 | .266 | -.002 | .034 | -.002 | -.032 |
|  | .217 | .100 | .218 | .199 | .193 | .199 | .212 | .197 |
|  | [-.052, .799] | [-.113, .279] | [-.156, .700] | [-.124, .655] | [-.380, .376] | [-.357, .425] | [-.418, .414] | [-.417, .354] |
|  | (.085) | (.408) | (.213) | (.181) | (.991) | (.865) | (.993) | (.873) |
| West Virginia | -.228 | .132 | .175 | .210 | .026 | .023 | -.041 | .155 |
|  | .276 | .127 | .277 | .253 | .245 | .254 | .270 | .250 |
|  | [-.769, .313] | [-.117, .380] | [-.367, .717] | [-.286, .705] | [-.454, .505] | [-.475, .520] | [-.571, .488] | [-.335, .645] |
|  | (.409) | (.299) | (.527) | (.407) | (.917) | (.929) | (.878) | (.535) |
| Wisconsin | .073 | .079 | -.007 | .072 | -.385 | -.186 | -.268 | -.179 |
|  | .224 | .103 | .225 | .205 | .199 | .206 | .219 | .203 |
|  | [-.367, .512] | [-.123, .282] | [-.449, .435] | [-.331, .474] | [-.775, .005] | [-.589, .218] | [-.698, .162] | [-.577, .220] |
|  | (.746) | (.442) | (.976) | (.727) | (.053) | (.368) | (.223) | (.379) |
| Wyoming | .603 | .130 | -.622 | -.370 | -.268 | .037 | -.016 | -.014 |
|  | .654 | .305 | .658 | .597 | .586 | .599 | .637 | .597 |
|  | [-.678, 1.884] | [-.469, .728] | [-1.912, .667] | [-1.539, .800] | [-1.416, .880] | [-1.137, 1.211] | [-1.265, 1.233] | [-1.185, 1.156] |
|  | (.356) | (.671) | (.344) | (.536) | (.647) | (.951) | (.980) | (.981) |
| N | 2982 | 2980 | 2968 | 2980 | 2981 | 2981 | 2981 | 2979 |
| Pseudo-R^2^ |  |  |  |  |  |  |  |  |
| Model χ^2^ | 201.4 | 305.8 | 45.4 | 279.8 | 275.3 | 211.4 | 227.4 | 294.8 |
| d.f. | 88 | 88 | 88 | 88 | 88 | 88 | 88 | 88 |
| p-value | <.001 | <.001 | <.001 | <.001 | <.001 | <.001 | <.001 | <.001 |

**S6 Fig. Health Attitudes, Multilevel Models.** This figure compares key coefficients of interest from S10-S12 Tables.

S13 Table. Regression Results, Health Policies, Multilevel Models.

|  | (1) | (2) | (3) | (4) | (5) | (6) | (7) | (8) | (9) |
| --- | --- | --- | --- | --- | --- | --- | --- | --- | --- |
|  | freetesting | waivecosts | closeschools | canceleverything | paidleave | compensatefirms | nationalguard | stopevents | stateslead |
| main |  |  |  |  |  |  |  |  |  |
| Democrat | .556 | .736 | .460 | .396 | .590 | -.174 | .215 | .497 | -.024 |
|  | .044 | .049 | .052 | .052 | .049 | .057 | .061 | .061 | .052 |
|  | [.469, .642] | [.640, .831] | [.358, .562] | [.295, .497] | [.494, .686] | [-.287, -.062] | [.097, .334] | [.377, .617] | [-.125, .078] |
|  | (<.001) | (<.001) | (<.001) | (<.001) | (<.001) | (.002) | (<.001) | (<.001) | (.648) |
| Other | .256 | .359 | .124 | .017 | .193 | -.357 | -.175 | .120 | -.166 |
|  | .045 | .049 | .053 | .052 | .050 | .058 | .061 | .062 | .052 |
|  | [.168, .343] | [.263, .456] | [.021, .227] | [-.085, .120] | [.096, .290] | [-.471, -.243] | [-.295, -.055] | [-.002, .241] | [-.269, -.064] |
|  | (<.001) | (<.001) | (.019) | (.743) | (<.001) | (<.001) | (.004) | (.053) | (.001) |
| 35- | .144 | .168 | .115 | .147 | .141 | .088 | .024 | .031 | .187 |
|  | .049 | .055 | .058 | .058 | .055 | .064 | .068 | .069 | .058 |
|  | [.046, .241] | [.061, .275] | [.001, .229] | [.033, .260] | [.034, .249] | [-.038, .214] | [-.110, .157] | [-.104, .165] | [.073, .300] |
|  | (.004) | (.002) | (.048) | (.011) | (.010) | (.173) | (.727) | (.655) | (.001) |
| 50- | .152 | .118 | .104 | .142 | .073 | .031 | -.077 | .176 | .178 |
|  | .047 | .052 | .055 | .055 | .052 | .061 | .064 | .065 | .055 |
|  | [.061, .244] | [.017, .219] | [-.004, .212] | [.035, .250] | [-.028, .175] | [-.088, .150] | [-.203, .049] | [.049, .304] | [.070, .285] |
|  | (.001) | (.022) | (.058) | (.009) | (.158) | (.611) | (.228) | (.007) | (.001) |
| 70- | .203 | .011 | .126 | .331 | -.007 | -.042 | .225 | .236 | .309 |
|  | .059 | .065 | .069 | .069 | .065 | .077 | .081 | .082 | .069 |
|  | [.087, .319] | [-.116, .139] | [-.010, .262] | [.196, .467] | [-.135, .121] | [-.192, .109] | [.065, .384] | [.075, .397] | [.173, .445] |
|  | (.001) | (.862) | (.070) | (<.001) | (.915) | (.590) | (.006) | (.004) | (<.001) |
| gender | .105 | .053 | .181 | .233 | .140 | .321 | .193 | .209 | .098 |
|  | .034 | .038 | .040 | .040 | .038 | .044 | .047 | .047 | .040 |
|  | [.039, .172] | [-.020, .127] | [.103, .260] | [.155, .311] | [.066, .214] | [.234, .407] | [.101, .285] | [.116, .301] | [.020, .176] |
|  | (.002) | (.157) | (<.001) | (<.001) | (<.001) | (<.001) | (<.001) | (<.001) | (.014) |
| white | .120 | .158 | .018 | .014 | .141 | -.060 | -.182 | .256 | -.027 |
|  | .041 | .045 | .048 | .048 | .045 | .053 | .056 | .056 | .048 |
|  | [.040, .199] | [.070, .246] | [-.076, .112] | [-.079, .107] | [.053, .229] | [-.164, .044] | [-.292, -.073] | [.145, .367] | [-.120, .066] |
|  | (.003) | (<.001) | (.709) | (.765) | (.002) | (.256) | (.001) | (<.001) | (.572) |
| married | -.045 | -.023 | -.016 | .036 | -.024 | -.007 | .128 | -.060 | .002 |
|  | .037 | .041 | .044 | .044 | .041 | .049 | .051 | .052 | .044 |
|  | [-.118, .028] | [-.104, .058] | [-.102, .071] | [-.050, .122] | [-.106, .057] | [-.102, .088] | [.027, .229] | [-.162, .042] | [-.084, .088] |
|  | (.229) | (.579) | (.724) | (.413) | (.558) | (.884) | (.013) | (.247) | (.962) |
| High school graduate | -.180 | -.161 | -.118 | -.076 | -.221 | -.269 | -.128 | .109 | -.234 |
|  | .100 | .110 | .117 | .117 | .111 | .130 | .137 | .138 | .117 |
|  | [-.375, .015] | [-.377, .056] | [-.348, .111] | [-.305, .154] | [-.439, -.004] | [-.523, -.014] | [-.397, .141] | [-.162, .381] | [-.464, -.004] |
|  | (.071) | (.145) | (.312) | (.518) | (.046) | (.038) | (.351) | (.429) | (.046) |
| Some college | -.100 | -.079 | -.156 | -.076 | -.127 | -.425 | -.389 | .322 | -.246 |
|  | .102 | .113 | .119 | .119 | .113 | .132 | .140 | .141 | .120 |
|  | [-.300, .099] | [-.300, .142] | [-.390, .078] | [-.310, .157] | [-.349, .094] | [-.684, -.166] | [-.663, -.115] | [.046, .599] | [-.480, -.011] |
|  | (.323) | (.483) | (.191) | (.522) | (.260) | (.001) | (.005) | (.022) | (.040) |
| 2-year | -.072 | -.088 | -.163 | -.065 | -.141 | -.263 | -.322 | .304 | -.254 |
|  | .107 | .118 | .126 | .125 | .119 | .139 | .147 | .148 | .126 |
|  | [-.281, .138] | [-.320, .144] | [-.409, .083] | [-.311, .181] | [-.374, .092] | [-.535, .010] | [-.610, -.034] | [.013, .594] | [-.500, -.007] |
|  | (.501) | (.457) | (.194) | (.602) | (.237) | (.059) | (.028) | (.041) | (.044) |
| 4-year | -.230 | -.228 | -.225 | -.122 | -.235 | -.426 | -.384 | .300 | -.280 |
|  | .104 | .115 | .122 | .122 | .115 | .135 | .143 | .144 | .122 |
|  | [-.434, -.027] | [-.453, -.003] | [-.464, .013] | [-.361, .116] | [-.461, -.009] | [-.691, -.162] | [-.664, -.104] | [.018, .582] | [-.520, -.041] |
|  | (.026) | (.047) | (.064) | (.315) | (.042) | (.002) | (.007) | (.037) | (.022) |
| Post-grad | -.209 | -.224 | -.307 | -.092 | -.248 | -.527 | -.349 | .385 | -.420 |
|  | .109 | .121 | .128 | .128 | .121 | .142 | .150 | .151 | .128 |
|  | [-.423, .004] | [-.460, .013] | [-.558, -.056] | [-.343, .158] | [-.486, -.010] | [-.805, -.249] | [-.642, -.055] | [.088, .681] | [-.671, -.169] |
|  | (.055) | (.064) | (.017) | (.471) | (.041) | (<.001) | (.020) | (.011) | (.001) |
| income=2 | .118 | .056 | .065 | .048 | -.005 | -.087 | .064 | .001 | .064 |
|  | .096 | .106 | .113 | .113 | .107 | .125 | .133 | .134 | .113 |
|  | [-.071, .307] | [-.152, .264] | [-.158, .287] | [-.173, .269] | [-.215, .204] | [-.332, .158] | [-.196, .324] | [-.261, .263] | [-.158, .285] |
|  | (.222) | (.597) | (.569) | (.669) | (.960) | (.485) | (.627) | (.992) | (.574) |
| income=3 | .188 | .116 | .116 | .054 | .136 | -.089 | -.028 | -.096 | .226 |
|  | .093 | .103 | .110 | .109 | .104 | .121 | .128 | .129 | .109 |
|  | [.005, .371] | [-.086, .318] | [-.099, .331] | [-.160, .267] | [-.067, .340] | [-.326, .149] | [-.279, .224] | [-.350, .157] | [.012, .440] |
|  | (.044) | (.259) | (.291) | (.622) | (.189) | (.465) | (.829) | (.456) | (.039) |
| income=4 | .212 | .189 | .115 | .142 | .092 | -.162 | .092 | .187 | .140 |
|  | .096 | .105 | .113 | .111 | .106 | .124 | .132 | .133 | .112 |
|  | [.024, .399] | [-.017, .396] | [-.106, .335] | [-.076, .361] | [-.116, .300] | [-.406, .081] | [-.166, .350] | [-.073, .447] | [-.079, .359] |
|  | (.027) | (.073) | (.308) | (.201) | (.384) | (.191) | (.484) | (.158) | (.211) |
| income=5 | .182 | .173 | -.036 | .031 | .090 | -.315 | -.154 | .020 | .088 |
|  | .098 | .108 | .115 | .114 | .108 | .127 | .134 | .135 | .114 |
|  | [-.009, .374] | [-.038, .384] | [-.262, .189] | [-.193, .254] | [-.122, .303] | [-.563, -.066] | [-.417, .109] | [-.245, .285] | [-.136, .312] |
|  | (.062) | (.108) | (.754) | (.789) | (.404) | (.013) | (.252) | (.881) | (.443) |
| income=6 | .168 | .101 | .137 | .137 | .119 | -.141 | .100 | .109 | .153 |
|  | .099 | .109 | .116 | .115 | .109 | .128 | .136 | .137 | .115 |
|  | [-.025, .362] | [-.112, .314] | [-.090, .365] | [-.089, .363] | [-.095, .334] | [-.391, .110] | [-.166, .366] | [-.159, .377] | [-.073, .379] |
|  | (.088) | (.353) | (.237) | (.236) | (.275) | (.272) | (.462) | (.425) | (.186) |
| income=7 | .235 | .021 | .235 | .213 | .117 | .015 | .036 | .267 | .022 |
|  | .106 | .116 | .124 | .123 | .118 | .137 | .145 | .147 | .124 |
|  | [.028, .442] | [-.207, .249] | [-.009, .479] | [-.028, .455] | [-.114, .347] | [-.254, .283] | [-.249, .321] | [-.021, .554] | [-.221, .264] |
|  | (.026) | (.854) | (.059) | (.084) | (.321) | (.915) | (.805) | (.069) | (.862) |
| income=8 | .193 | .088 | .084 | .051 | .205 | -.059 | -.142 | .051 | .083 |
|  | .103 | .113 | .121 | .120 | .114 | .134 | .142 | .143 | .120 |
|  | [-.009, .395] | [-.134, .310] | [-.153, .321] | [-.185, .286] | [-.018, .429] | [-.321, .203] | [-.419, .136] | [-.229, .330] | [-.153, .318] |
|  | (.061) | (.439) | (.488) | (.673) | (.072) | (.660) | (.318) | (.723) | (.493) |
| income=9 | .013 | -.137 | .072 | -.018 | .030 | -.168 | -.246 | .200 | .040 |
|  | .102 | .112 | .120 | .119 | .113 | .132 | .140 | .141 | .119 |
|  | [-.187, .213] | [-.357, .083] | [-.163, .307] | [-.250, .215] | [-.191, .251] | [-.427, .091] | [-.521, .028] | [-.076, .477] | [-.193, .274] |
|  | (.900) | (.222) | (.547) | (.883) | (.788) | (.204) | (.078) | (.156) | (.734) |
| income=10 | .187 | .039 | .022 | .153 | .124 | -.073 | -.086 | .201 | .071 |
|  | .106 | .117 | .124 | .123 | .117 | .137 | .145 | .147 | .124 |
|  | [-.021, .394] | [-.189, .268] | [-.222, .266] | [-.089, .395] | [-.106, .354] | [-.342, .196] | [-.371, .199] | [-.086, .489] | [-.171, .314] |
|  | (.078) | (.736) | (.860) | (.214) | (.290) | (.595) | (.553) | (.170) | (.566) |
| income=11 | .213 | -.053 | .185 | .224 | .010 | -.090 | -.132 | .270 | .105 |
|  | .109 | .120 | .128 | .127 | .120 | .141 | .149 | .151 | .127 |
|  | [-.001, .426] | [-.288, .182] | [-.066, .435] | [-.024, .473] | [-.226, .246] | [-.367, .186] | [-.425, .161] | [-.025, .566] | [-.145, .354] |
|  | (.051) | (.657) | (.148) | (.077) | (.935) | (.521) | (.377) | (.073) | (.411) |
| income=12 | .071 | -.128 | .068 | .104 | .001 | -.107 | -.276 | .348 | .083 |
|  | .120 | .132 | .141 | .140 | .132 | .155 | .165 | .166 | .140 |
|  | [-.164, .305] | [-.386, .130] | [-.209, .344] | [-.170, .378] | [-.258, .260] | [-.412, .197] | [-.599, .046] | [.023, .673] | [-.191, .357] |
|  | (.555) | (.331) | (.630) | (.458) | (.994) | (.489) | (.093) | (.036) | (.551) |
| income=13 | .392 | .187 | .123 | .340 | .165 | -.387 | -.070 | .364 | .042 |
|  | .165 | .182 | .196 | .195 | .183 | .214 | .227 | .229 | .193 |
|  | [.069, .715] | [-.169, .543] | [-.262, .507] | [-.042, .722] | [-.193, .523] | [-.807, .033] | [-.515, .374] | [-.085, .813] | [-.337, .421] |
|  | (.018) | (.303) | (.531) | (.081) | (.367) | (.071) | (.756) | (.112) | (.828) |
| income=14 | -.122 | -.187 | -.266 | -.011 | -.095 | -.239 | -.513 | .279 | -.272 |
|  | .175 | .193 | .205 | .204 | .194 | .227 | .240 | .242 | .205 |
|  | [-.464, .221] | [-.564, .191] | [-.668, .137] | [-.411, .389] | [-.475, .284] | [-.685, .206] | [-.983, -.042] | [-.196, .754] | [-.673, .129] |
|  | (.487) | (.333) | (.196) | (.958) | (.623) | (.292) | (.033) | (.250) | (.183) |
| income=15 | .697 | .756 | .015 | .293 | .493 | .175 | 1.428 | .846 | 1.240 |
|  | .529 | .583 | .621 | .618 | .586 | .688 | .727 | .734 | .619 |
|  | [-.338, 1.733] | [-.387, 1.898] | [-1.202, 1.233] | [-.918, 1.505] | [-.655, 1.641] | [-1.173, 1.523] | [.004, 2.852] | [-.592, 2.283] | [.027, 2.453] |
|  | (.187) | (.195) | (.980) | (.635) | (.400) | (.799) | (.049) | (.249) | (.045) |
| income=16 | .408 | .450 | .410 | .473 | .563 | -.021 | .104 | -.032 | .150 |
|  | .248 | .274 | .292 | .291 | .275 | .323 | .342 | .345 | .291 |
|  | [-.079, .895] | [-.087, .987] | [-.162, .983] | [-.096, 1.042] | [.023, 1.103] | [-.655, .613] | [-.565, .774] | [-.707, .644] | [-.420, .720] |
|  | (.101) | (.100) | (.160) | (.104) | (.041) | (.948) | (.760) | (.927) | (.607) |
| income=17 | .072 | .001 | -.007 | .005 | -.035 | -.184 | -.334 | .184 | .101 |
|  | .091 | .100 | .107 | .106 | .100 | .118 | .125 | .126 | .106 |
|  | [-.106, .250] | [-.195, .197] | [-.216, .202] | [-.202, .212] | [-.232, .162] | [-.414, .047] | [-.578, -.090] | [-.062, .431] | [-.107, .309] |
|  | (.426) | (.992) | (.948) | (.960) | (.726) | (.118) | (.007) | (.143) | (.340) |
| Rural Code=2 | -.057 | -.059 | -.118 | -.089 | -.006 | -.110 | -.125 | .012 | -.038 |
|  | .045 | .050 | .053 | .053 | .050 | .059 | .062 | .063 | .053 |
|  | [-.145, .032] | [-.157, .038] | [-.221, -.014] | [-.192, .015] | [-.104, .092] | [-.225, .005] | [-.247, -.003] | [-.111, .135] | [-.143, .066] |
|  | (.208) | (.232) | (.026) | (.092) | (.905) | (.060) | (.045) | (.846) | (.470) |
| Rural Code=3 | -.098 | -.202 | -.163 | -.045 | -.031 | -.027 | -.049 | .038 | -.068 |
|  | .061 | .068 | .072 | .072 | .068 | .080 | .085 | .085 | .072 |
|  | [-.218, .022] | [-.335, -.070] | [-.304, -.022] | [-.186, .095] | [-.164, .102] | [-.184, .130] | [-.215, .117] | [-.129, .206] | [-.210, .074] |
|  | (.109) | (.003) | (.024) | (.527) | (.646) | (.735) | (.562) | (.652) | (.349) |
| Rural Code=4 | -.051 | .038 | -.247 | -.066 | .084 | -.053 | -.149 | -.142 | .055 |
|  | .085 | .094 | .100 | .100 | .095 | .111 | .118 | .119 | .101 |
|  | [-.218, .117] | [-.147, .223] | [-.444, -.051] | [-.262, .130] | [-.101, .270] | [-.271, .165] | [-.380, .082] | [-.375, .091] | [-.143, .253] |
|  | (.551) | (.687) | (.014) | (.507) | (.374) | (.636) | (.205) | (.233) | (.585) |
| Rural Code=5 | -.018 | -.109 | .071 | .088 | .118 | -.094 | .017 | .313 | -.120 |
|  | .131 | .144 | .154 | .153 | .145 | .170 | .180 | .182 | .154 |
|  | [-.274, .239] | [-.392, .174] | [-.231, .373] | [-.212, .388] | [-.166, .403] | [-.428, .240] | [-.336, .371] | [-.043, .670] | [-.422, .182] |
|  | (.893) | (.450) | (.644) | (.565) | (.414) | (.580) | (.925) | (.085) | (.437) |
| Rural Code=6 | -.005 | -.040 | -.107 | -.140 | .039 | .001 | .016 | -.078 | -.229 |
|  | .085 | .094 | .100 | .099 | .094 | .111 | .117 | .118 | .100 |
|  | [-.172, .161] | [-.224, .144] | [-.303, .089] | [-.335, .054] | [-.146, .223] | [-.215, .218] | [-.214, .245] | [-.309, .154] | [-.426, -.033] |
|  | (.949) | (.671) | (.284) | (.158) | (.682) | (.989) | (.892) | (.511) | (.022) |
| Rural Code=7 | -.205 | -.103 | -.334 | -.184 | -.214 | -.167 | -.115 | -.214 | -.226 |
|  | .118 | .129 | .138 | .137 | .130 | .152 | .161 | .163 | .139 |
|  | [-.436, .026] | [-.356, .150] | [-.606, -.063] | [-.452, .084] | [-.468, .040] | [-.465, .132] | [-.431, .201] | [-.533, .105] | [-.498, .045] |
|  | (.082) | (.423) | (.016) | (.179) | (.099) | (.274) | (.476) | (.189) | (.102) |
| Rural Code=8 | -.060 | -.118 | -.242 | -.410 | -.018 | .102 | -.725 | -.033 | .432 |
|  | .220 | .242 | .258 | .257 | .250 | .286 | .310 | .305 | .258 |
|  | [-.490, .371] | [-.593, .357] | [-.748, .264] | [-.913, .094] | [-.508, .472] | [-.458, .663] | [-1.333, -.117] | [-.631, .565] | [-.073, .937] |
|  | (.786) | (.626) | (.349) | (.111) | (.943) | (.720) | (.019) | (.914) | (.094) |
| Rural Code=9 | -.018 | .062 | .072 | -.059 | .049 | -.440 | .046 | .051 | -.033 |
|  | .186 | .205 | .219 | .218 | .206 | .243 | .256 | .259 | .219 |
|  | [-.383, .347] | [-.341, .464] | [-.357, .501] | [-.486, .368] | [-.355, .454] | [-.915, .036] | [-.456, .549] | [-.457, .558] | [-.462, .396] |
|  | (.924) | (.764) | (.742) | (.787) | (.811) | (.070) | (.857) | (.845) | (.881) |
| Alaska | .001 | -.031 | .297 | .255 | .014 | .385 | .148 | .352 | -.018 |
|  | .374 | .412 | .439 | .437 | .415 | .487 | .514 | .520 | .439 |
|  | [-.731, .733] | [-.839, .777] | [-.564, 1.158] | [-.601, 1.112] | [-.799, .827] | [-.570, 1.340] | [-.860, 1.155] | [-.667, 1.371] | [-.878, .843] |
|  | (.998) | (.940) | (.499) | (.559) | (.973) | (.429) | (.774) | (.499) | (.968) |
| Arizona | -.216 | -.079 | -.015 | -.126 | -.069 | -.286 | -.322 | .092 | -.282 |
|  | .178 | .196 | .210 | .208 | .199 | .233 | .245 | .250 | .211 |
|  | [-.565, .132] | [-.463, .305] | [-.426, .396] | [-.534, .281] | [-.459, .321] | [-.744, .172] | [-.802, .158] | [-.397, .581] | [-.696, .131] |
|  | (.223) | (.686) | (.943) | (.544) | (.728) | (.221) | (.189) | (.712) | (.181) |
| Arkansas | -.175 | -.150 | -.074 | -.106 | -.133 | -.263 | -.054 | .132 | -.120 |
|  | .200 | .220 | .235 | .234 | .223 | .262 | .275 | .280 | .236 |
|  | [-.567, .216] | [-.582, .281] | [-.534, .386] | [-.564, .352] | [-.569, .304] | [-.776, .250] | [-.593, .485] | [-.416, .680] | [-.583, .343] |
|  | (.380) | (.495) | (.754) | (.650) | (.551) | (.314) | (.844) | (.636) | (.612) |
| California | -.079 | .094 | .073 | -.039 | .025 | -.251 | .128 | .268 | -.187 |
|  | .157 | .173 | .184 | .183 | .175 | .206 | .216 | .220 | .186 |
|  | [-.386, .228] | [-.244, .433] | [-.287, .434] | [-.399, .320] | [-.319, .369] | [-.655, .154] | [-.294, .551] | [-.164, .700] | [-.551, .178] |
|  | (.615) | (.584) | (.690) | (.830) | (.887) | (.224) | (.552) | (.224) | (.315) |
| Colorado | -.108 | -.342 | -.103 | -.070 | -.146 | -.237 | .053 | .312 | -.150 |
|  | .201 | .222 | .236 | .235 | .224 | .263 | .277 | .281 | .238 |
|  | [-.501, .286] | [-.778, .094] | [-.566, .359] | [-.530, .390] | [-.585, .293] | [-.753, .279] | [-.489, .595] | [-.240, .863] | [-.617, .316] |
|  | (.591) | (.125) | (.662) | (.766) | (.515) | (.368) | (.848) | (.268) | (.527) |
| Connecticut | .111 | .305 | .519 | .444 | .100 | .152 | .255 | .199 | .221 |
|  | .218 | .241 | .256 | .255 | .243 | .286 | .301 | .305 | .258 |
|  | [-.316, .539] | [-.167, .777] | [.017, 1.022] | [-.056, .944] | [-.377, .576] | [-.408, .712] | [-.335, .844] | [-.399, .798] | [-.285, .727] |
|  | (.609) | (.205) | (.043) | (.082) | (.681) | (.595) | (.397) | (.514) | (.392) |
| Delaware | -.322 | .189 | .111 | .130 | -.343 | -.623 | .305 | -.037 | -.285 |
|  | .282 | .311 | .331 | .330 | .314 | .368 | .389 | .394 | .343 |
|  | [-.874, .231] | [-.420, .799] | [-.538, .761] | [-.517, .776] | [-.958, .271] | [-1.345, .099] | [-.458, 1.068] | [-.810, .736] | [-.957, .387] |
|  | (.254) | (.542) | (.737) | (.694) | (.274) | (.091) | (.433) | (.926) | (.406) |
| District of Columbia | -.638 | -.088 | .125 | -.215 | -.615 | .063 | .499 | .568 | .124 |
|  | .352 | .389 | .438 | .412 | .391 | .460 | .485 | .490 | .438 |
|  | [-1.328, .053] | [-.850, .673] | [-.733, .984] | [-1.022, .593] | [-1.381, .152] | [-.838, .964] | [-.451, 1.449] | [-.393, 1.529] | [-.734, .982] |
|  | (.070) | (.820) | (.775) | (.602) | (.116) | (.891) | (.303) | (.247) | (.777) |
| Florida | -.133 | -.067 | .168 | -.085 | -.102 | -.160 | .244 | .293 | -.058 |
|  | .158 | .175 | .186 | .185 | .177 | .209 | .218 | .223 | .188 |
|  | [-.444, .177] | [-.410, .275] | [-.197, .532] | [-.448, .278] | [-.450, .245] | [-.569, .249] | [-.183, .672] | [-.144, .729] | [-.426, .311] |
|  | (.401) | (.699) | (.368) | (.648) | (.564) | (.443) | (.263) | (.189) | (.759) |
| Georgia | -.203 | -.306 | .026 | -.220 | -.059 | -.344 | .196 | .039 | -.142 |
|  | .171 | .189 | .202 | .201 | .192 | .225 | .236 | .241 | .203 |
|  | [-.539, .133] | [-.676, .065] | [-.369, .422] | [-.613, .173] | [-.435, .317] | [-.785, .098] | [-.267, .659] | [-.432, .511] | [-.540, .256] |
|  | (.237) | (.106) | (.897) | (.272) | (.757) | (.127) | (.406) | (.870) | (.484) |
| Hawaii | .258 | .597 | .617 | .363 | .091 | -.132 | .420 | -.017 | .039 |
|  | .303 | .335 | .356 | .355 | .337 | .396 | .419 | .424 | .359 |
|  | [-.336, .852] | [-.058, 1.253] | [-.082, 1.315] | [-.332, 1.059] | [-.570, .752] | [-.908, .644] | [-.401, 1.240] | [-.848, .814] | [-.665, .743] |
|  | (.395) | (.074) | (.084) | (.306) | (.787) | (.739) | (.316) | (.968) | (.913) |
| Idaho | -.362 | -.407 | .203 | -.309 | -.487 | -.338 | -.504 | .347 | -.285 |
|  | .254 | .280 | .299 | .297 | .283 | .332 | .351 | .356 | .301 |
|  | [-.860, .136] | [-.956, .142] | [-.382, .788] | [-.891, .274] | [-1.041, .067] | [-.989, .313] | [-1.192, .183] | [-.350, 1.044] | [-.875, .306] |
|  | (.154) | (.146) | (.497) | (.299) | (.085) | (.308) | (.150) | (.330) | (.345) |
| Illinois | -.111 | -.095 | .146 | .048 | .022 | -.223 | .202 | .294 | -.066 |
|  | .169 | .186 | .198 | .197 | .189 | .222 | .233 | .237 | .200 |
|  | [-.442, .220] | [-.460, .270] | [-.242, .535] | [-.339, .435] | [-.348, .392] | [-.658, .213] | [-.253, .658] | [-.171, .759] | [-.459, .326] |
|  | (.512) | (.609) | (.461) | (.808) | (.906) | (.316) | (.384) | (.216) | (.741) |
| Indiana | .009 | .256 | .483 | .220 | .012 | -.243 | .075 | .675 | -.201 |
|  | .187 | .206 | .220 | .218 | .209 | .245 | .258 | .262 | .222 |
|  | [-.357, .375] | [-.147, .660] | [.051, .914] | [-.208, .648] | [-.397, .420] | [-.723, .238] | [-.431, .580] | [.162, 1.188] | [-.635, .234] |
|  | (.961) | (.213) | (.028) | (.315) | (.956) | (.322) | (.773) | (.010) | (.365) |
| Iowa | -.192 | -.195 | .024 | .051 | -.109 | .121 | .327 | .570 | -.049 |
|  | .222 | .245 | .261 | .260 | .247 | .291 | .305 | .310 | .262 |
|  | [-.627, .243] | [-.675, .285] | [-.487, .536] | [-.458, .560] | [-.593, .376] | [-.448, .691] | [-.272, .926] | [-.037, 1.178] | [-.562, .464] |
|  | (.388) | (.426) | (.926) | (.844) | (.661) | (.676) | (.284) | (.066) | (.851) |
| Kansas | -.023 | .154 | .013 | .342 | .135 | -.205 | .151 | .576 | -.178 |
|  | .269 | .296 | .316 | .314 | .299 | .351 | .370 | .375 | .317 |
|  | [-.549, .504] | [-.426, .735] | [-.606, .631] | [-.274, .957] | [-.451, .720] | [-.893, .483] | [-.574, .876] | [-.159, 1.311] | [-.799, .443] |
|  | (.932) | (.603) | (.968) | (.277) | (.652) | (.559) | (.683) | (.124) | (.574) |
| Kentucky | -.353 | -.111 | .052 | -.290 | -.059 | .107 | -.178 | .258 | -.074 |
|  | .203 | .224 | .239 | .238 | .228 | .266 | .281 | .284 | .240 |
|  | [-.752, .045] | [-.550, .328] | [-.416, .520] | [-.756, .176] | [-.506, .387] | [-.415, .629] | [-.729, .373] | [-.300, .815] | [-.545, .397] |
|  | (.082) | (.621) | (.828) | (.222) | (.794) | (.688) | (.527) | (.365) | (.757) |
| Louisiana | -.177 | .192 | .070 | -.173 | -.090 | -.219 | .128 | .128 | -.137 |
|  | .204 | .225 | .239 | .238 | .227 | .267 | .281 | .286 | .242 |
|  | [-.576, .223] | [-.249, .632] | [-.399, .540] | [-.640, .294] | [-.535, .356] | [-.742, .305] | [-.423, .679] | [-.432, .687] | [-.610, .337] |
|  | (.386) | (.393) | (.769) | (.468) | (.693) | (.413) | (.649) | (.655) | (.571) |
| Maine | -.153 | -.031 | .743 | .371 | .098 | -.112 | -.363 | .300 | .308 |
|  | .282 | .311 | .332 | .330 | .314 | .369 | .389 | .395 | .334 |
|  | [-.707, .400] | [-.641, .579] | [.093, 1.393] | [-.276, 1.018] | [-.517, .714] | [-.835, .611] | [-1.126, .400] | [-.473, 1.074] | [-.347, .963] |
|  | (.587) | (.921) | (.025) | (.262) | (.754) | (.761) | (.351) | (.447) | (.356) |
| Maryland | -.125 | -.015 | -.039 | -.189 | .158 | -.246 | .271 | .154 | -.067 |
|  | .202 | .223 | .237 | .236 | .225 | .265 | .278 | .283 | .239 |
|  | [-.520, .271] | [-.451, .421] | [-.503, .426] | [-.652, .273] | [-.283, .599] | [-.764, .273] | [-.274, .816] | [-.400, .708] | [-.535, .401] |
|  | (.536) | (.947) | (.870) | (.422) | (.482) | (.353) | (.329) | (.586) | (.778) |
| Massachusetts | -.044 | .084 | -.026 | -.038 | .063 | -.134 | .091 | .483 | -.319 |
|  | .189 | .209 | .223 | .222 | .211 | .249 | .261 | .266 | .224 |
|  | [-.415, .327] | [-.326, .493] | [-.462, .410] | [-.472, .396] | [-.351, .477] | [-.621, .353] | [-.421, .603] | [-.038, 1.004] | [-.759, .120] |
|  | (.817) | (.689) | (.907) | (.864) | (.766) | (.590) | (.727) | (.069) | (.155) |
| Michigan | -.152 | .002 | .244 | -.181 | -.157 | -.175 | .026 | .366 | -.081 |
|  | .169 | .186 | .199 | .198 | .189 | .222 | .233 | .238 | .200 |
|  | [-.484, .179] | [-.364, .367] | [-.145, .633] | [-.568, .207] | [-.528, .213] | [-.610, .261] | [-.431, .482] | [-.100, .831] | [-.473, .312] |
|  | (.368) | (.993) | (.219) | (.361) | (.406) | (.432) | (.913) | (.124) | (.687) |
| Minnesota | -.323 | -.175 | .120 | -.013 | -.231 | -.197 | .036 | .199 | .032 |
|  | .199 | .220 | .234 | .233 | .223 | .262 | .275 | .279 | .236 |
|  | [-.714, .068] | [-.607, .256] | [-.340, .579] | [-.471, .444] | [-.667, .205] | [-.710, .316] | [-.502, .575] | [-.349, .747] | [-.430, .494] |
|  | (.105) | (.425) | (.609) | (.955) | (.300) | (.451) | (.895) | (.476) | (.891) |
| Mississippi | -.396 | -.307 | .138 | -.028 | -.091 | .068 | .270 | .071 | .069 |
|  | .226 | .249 | .265 | .264 | .252 | .296 | .311 | .315 | .266 |
|  | [-.838, .047] | [-.796, .181] | [-.382, .659] | [-.546, .490] | [-.584, .402] | [-.512, .647] | [-.340, .879] | [-.547, .690] | [-.453, .591] |
|  | (.080) | (.217) | (.602) | (.916) | (.717) | (.819) | (.386) | (.821) | (.796) |
| Missouri | -.318 | -.165 | -.055 | -.289 | -.039 | -.315 | .095 | .138 | -.091 |
|  | .184 | .202 | .215 | .214 | .204 | .241 | .252 | .257 | .217 |
|  | [-.679, .042] | [-.562, .231] | [-.476, .367] | [-.708, .130] | [-.440, .361] | [-.786, .157] | [-.399, .588] | [-.367, .642] | [-.517, .334] |
|  | (.083) | (.414) | (.799) | (.176) | (.847) | (.191) | (.707) | (.593) | (.674) |
| Montana | -.721 | -.691 | -.183 | -.563 | -.122 | -.812 | -.327 | -.391 | -.507 |
|  | .282 | .311 | .331 | .330 | .313 | .368 | .388 | .394 | .333 |
|  | [-1.273, -.169] | [-1.300, -.082] | [-.832, .466] | [-1.209, .083] | [-.736, .492] | [-1.533, -.090] | [-1.089, .434] | [-1.163, .380] | [-1.160, .145] |
|  | (.011) | (.026) | (.580) | (.087) | (.697) | (.027) | (.400) | (.320) | (.128) |
| Nebraska | -.087 | .239 | -.391 | -.173 | .011 | .038 | .439 | -.080 | -.094 |
|  | .299 | .320 | .341 | .340 | .323 | .391 | .399 | .405 | .342 |
|  | [-.673, .499] | [-.389, .867] | [-1.060, .278] | [-.839, .492] | [-.622, .644] | [-.727, .804] | [-.344, 1.222] | [-.873, .713] | [-.764, .575] |
|  | (.771) | (.456) | (.252) | (.610) | (.973) | (.922) | (.271) | (.843) | (.783) |
| Nevada | -.041 | .063 | .187 | .002 | -.145 | -.375 | -.178 | .400 | -.002 |
|  | .199 | .219 | .234 | .233 | .223 | .261 | .274 | .279 | .236 |
|  | [-.431, .348] | [-.367, .493] | [-.271, .645] | [-.454, .458] | [-.582, .292] | [-.886, .137] | [-.716, .360] | [-.147, .947] | [-.464, .461] |
|  | (.835) | (.775) | (.424) | (.994) | (.516) | (.151) | (.516) | (.151) | (.995) |
| New Hampshire | .003 | .207 | -.035 | -.155 | .026 | -.275 | .344 | .041 | .033 |
|  | .239 | .263 | .280 | .279 | .266 | .312 | .329 | .334 | .283 |
|  | [-.465, .470] | [-.308, .723] | [-.585, .515] | [-.702, .392] | [-.494, .547] | [-.886, .337] | [-.301, .989] | [-.614, .696] | [-.521, .587] |
|  | (.991) | (.431) | (.900) | (.579) | (.921) | (.379) | (.296) | (.903) | (.908) |
| New Jersey | -.099 | .192 | .195 | -.039 | .027 | .027 | .390 | .200 | -.030 |
|  | .185 | .204 | .218 | .216 | .206 | .242 | .254 | .259 | .218 |
|  | [-.461, .263] | [-.208, .591] | [-.231, .622] | [-.462, .385] | [-.376, .431] | [-.447, .501] | [-.108, .889] | [-.308, .707] | [-.456, .397] |
|  | (.593) | (.346) | (.370) | (.857) | (.894) | (.911) | (.125) | (.441) | (.892) |
| New Mexico | .141 | -.148 | .265 | -.135 | -.143 | -.345 | -.407 | .298 | -.354 |
|  | .220 | .242 | .258 | .257 | .245 | .288 | .303 | .308 | .260 |
|  | [-.290, .572] | [-.624, .327] | [-.241, .772] | [-.639, .369] | [-.623, .337] | [-.909, .219] | [-1.001, .187] | [-.305, .902] | [-.864, .156] |
|  | (.520) | (.541) | (.305) | (.600) | (.560) | (.231) | (.180) | (.332) | (.174) |
| New York | -.279 | -.017 | .108 | -.103 | -.096 | -.182 | .210 | .011 | -.048 |
|  | .162 | .178 | .190 | .189 | .181 | .212 | .222 | .227 | .191 |
|  | [-.595, .038] | [-.366, .332] | [-.264, .480] | [-.473, .267] | [-.450, .259] | [-.599, .234] | [-.226, .645] | [-.434, .455] | [-.423, .327] |
|  | (.084) | (.923) | (.570) | (.584) | (.597) | (.391) | (.346) | (.963) | (.801) |
| North Carolina | -.127 | .085 | .060 | .092 | -.055 | -.382 | .256 | .075 | -.265 |
|  | .174 | .192 | .205 | .204 | .195 | .230 | .240 | .244 | .207 |
|  | [-.468, .214] | [-.292, .461] | [-.342, .461] | [-.308, .492] | [-.436, .327] | [-.832, .068] | [-.214, .726] | [-.404, .554] | [-.670, .141] |
|  | (.466) | (.659) | (.771) | (.652) | (.779) | (.096) | (.287) | (.758) | (.201) |
| North Dakota | -.189 | .428 | -.366 | -.258 | .197 | -.172 | .544 | -.093 | -.487 |
|  | .354 | .390 | .416 | .414 | .393 | .461 | .488 | .493 | .418 |
|  | [-.882, .504] | [-.337, 1.192] | [-1.180, .449] | [-1.069, .553] | [-.573, .967] | [-1.076, .732] | [-.412, 1.500] | [-1.060, .874] | [-1.306, .332] |
|  | (.593) | (.273) | (.379) | (.533) | (.616) | (.710) | (.264) | (.850) | (.244) |
| Ohio | -.197 | -.022 | .161 | -.014 | -.029 | -.080 | -.025 | .340 | .073 |
|  | .169 | .187 | .199 | .198 | .189 | .223 | .233 | .238 | .201 |
|  | [-.529, .135] | [-.388, .344] | [-.229, .551] | [-.403, .375] | [-.400, .342] | [-.517, .356] | [-.483, .432] | [-.126, .806] | [-.320, .467] |
|  | (.244) | (.905) | (.419) | (.944) | (.878) | (.718) | (.913) | (.152) | (.715) |
| Oklahoma | -.268 | -.001 | .202 | .174 | -.149 | .237 | .415 | .017 | -.228 |
|  | .227 | .250 | .267 | .266 | .253 | .297 | .313 | .317 | .268 |
|  | [-.713, .177] | [-.492, .489] | [-.321, .725] | [-.347, .694] | [-.645, .347] | [-.346, .819] | [-.198, 1.027] | [-.605, .639] | [-.753, .297] |
|  | (.237) | (.995) | (.449) | (.513) | (.557) | (.426) | (.185) | (.958) | (.395) |
| Oregon | .036 | .132 | -.281 | -.138 | -.032 | -.340 | -.073 | .251 | -.392 |
|  | .188 | .207 | .220 | .219 | .209 | .246 | .258 | .263 | .222 |
|  | [-.332, .404] | [-.273, .537] | [-.712, .150] | [-.568, .291] | [-.442, .378] | [-.822, .142] | [-.580, .433] | [-.264, .767] | [-.828, .044] |
|  | (.847) | (.522) | (.202) | (.528) | (.879) | (.166) | (.777) | (.340) | (.078) |
| Pennsylvania | -.124 | -.038 | .089 | -.033 | -.001 | -.180 | .008 | .324 | -.018 |
|  | .161 | .177 | .189 | .188 | .180 | .211 | .221 | .225 | .190 |
|  | [-.439, .191] | [-.385, .309] | [-.281, .459] | [-.400, .335] | [-.353, .351] | [-.594, .234] | [-.425, .440] | [-.118, .766] | [-.391, .355] |
|  | (.441) | (.830) | (.638) | (.862) | (.996) | (.395) | (.973) | (.151) | (.925) |
| Rhode Island | .326 | .300 | .302 | -.043 | .268 | .826 | .037 | -.100 | -.037 |
|  | .352 | .388 | .413 | .411 | .391 | .459 | .484 | .490 | .413 |
|  | [-.364, 1.015] | [-.461, 1.060] | [-.508, 1.112] | [-.850, .763] | [-.498, 1.034] | [-.074, 1.725] | [-.911, .985] | [-1.060, .859] | [-.847, .773] |
|  | (.354) | (.440) | (.465) | (.916) | (.493) | (.072) | (.939) | (.838) | (.928) |
| South Carolina | -.128 | -.120 | .270 | .164 | -.320 | -.120 | .021 | .389 | -.028 |
|  | .196 | .217 | .231 | .230 | .219 | .258 | .271 | .275 | .233 |
|  | [-.513, .257] | [-.545, .305] | [-.183, .722] | [-.286, .614] | [-.749, .110] | [-.624, .385] | [-.510, .552] | [-.151, .929] | [-.485, .429] |
|  | (.515) | (.579) | (.243) | (.476) | (.145) | (.642) | (.939) | (.158) | (.905) |
| South Dakota | -.276 | -.144 | -.508 | -.240 | -.231 | -.217 | .237 | -.383 | -.057 |
|  | .325 | .359 | .382 | .380 | .361 | .424 | .448 | .454 | .384 |
|  | [-.913, .362] | [-.847, .560] | [-1.257, .242] | [-.986, .506] | [-.939, .477] | [-1.049, .615] | [-.642, 1.115] | [-1.273, .506] | [-.810, .695] |
|  | (.397) | (.689) | (.184) | (.528) | (.523) | (.609) | (.598) | (.398) | (.881) |
| Tennessee | .035 | .182 | .025 | -.285 | -.087 | -.208 | -.106 | .319 | -.234 |
|  | .192 | .212 | .226 | .225 | .214 | .252 | .265 | .269 | .227 |
|  | [-.341, .412] | [-.234, .597] | [-.418, .467] | [-.726, .155] | [-.508, .333] | [-.702, .286] | [-.625, .413] | [-.208, .847] | [-.680, .212] |
|  | (.855) | (.392) | (.912) | (.204) | (.684) | (.409) | (.688) | (.236) | (.304) |
| Texas | -.175 | -.095 | -.014 | -.108 | -.240 | -.282 | -.007 | .091 | -.092 |
|  | .161 | .177 | .189 | .188 | .180 | .211 | .221 | .226 | .190 |
|  | [-.490, .139] | [-.442, .252] | [-.384, .356] | [-.476, .260] | [-.592, .113] | [-.696, .133] | [-.440, .426] | [-.351, .533] | [-.465, .281] |
|  | (.275) | (.591) | (.941) | (.565) | (.182) | (.183) | (.974) | (.686) | (.629) |
| Utah | -.208 | -.020 | -.068 | .026 | -.062 | -.454 | .222 | .524 | -.301 |
|  | .222 | .245 | .261 | .260 | .248 | .291 | .307 | .312 | .264 |
|  | [-.644, .228] | [-.501, .461] | [-.580, .444] | [-.484, .536] | [-.548, .424] | [-1.025, .116] | [-.379, .824] | [-.087, 1.135] | [-.818, .216] |
|  | (.350) | (.935) | (.794) | (.921) | (.801) | (.119) | (.469) | (.092) | (.254) |
| Vermont | .020 | -.069 | .321 | .312 | -.378 | -.285 | .264 | .663 | -.042 |
|  | .322 | .355 | .378 | .376 | .358 | .420 | .443 | .448 | .378 |
|  | [-.611, .651] | [-.765, .627] | [-.421, 1.062] | [-.426, 1.050] | [-1.079, .323] | [-1.108, .538] | [-.603, 1.132] | [-.215, 1.541] | [-.784, .699] |
|  | (.951) | (.846) | (.396) | (.408) | (.291) | (.498) | (.551) | (.139) | (.911) |
| Virginia | -.100 | -.043 | -.001 | -.129 | -.068 | -.377 | .085 | .311 | .013 |
|  | .172 | .190 | .202 | .201 | .193 | .226 | .237 | .242 | .204 |
|  | [-.437, .237] | [-.415, .328] | [-.398, .395] | [-.523, .266] | [-.446, .309] | [-.820, .067] | [-.380, .550] | [-.162, .785] | [-.387, .413] |
|  | (.561) | (.819) | (.995) | (.523) | (.722) | (.096) | (.721) | (.198) | (.949) |
| Washington | -.108 | -.012 | -.105 | -.150 | .028 | -.333 | -.210 | .306 | -.278 |
|  | .181 | .199 | .212 | .211 | .202 | .237 | .249 | .254 | .214 |
|  | [-.462, .246] | [-.402, .378] | [-.520, .311] | [-.564, .263] | [-.368, .423] | [-.797, .132] | [-.697, .277] | [-.192, .803] | [-.698, .141] |
|  | (.551) | (.952) | (.622) | (.477) | (.891) | (.160) | (.398) | (.229) | (.194) |
| West Virginia | -.642 | -.077 | .289 | -.037 | -.196 | -.319 | .144 | .397 | .023 |
|  | .230 | .253 | .273 | .269 | .256 | .301 | .316 | .321 | .275 |
|  | [-1.092, -.192] | [-.573, .420] | [-.247, .825] | [-.564, .489] | [-.697, .306] | [-.908, .270] | [-.476, .763] | [-.232, 1.026] | [-.515, .561] |
|  | (.005) | (.762) | (.291) | (.890) | (.445) | (.288) | (.649) | (.216) | (.934) |
| Wisconsin | -.339 | -.081 | .074 | -.096 | -.324 | -.362 | .041 | .317 | -.123 |
|  | .187 | .206 | .219 | .218 | .208 | .245 | .257 | .262 | .221 |
|  | [-.705, .027] | [-.486, .323] | [-.355, .504] | [-.523, .332] | [-.732, .085] | [-.841, .118] | [-.462, .545] | [-.196, .829] | [-.556, .309] |
|  | (.069) | (.693) | (.734) | (.661) | (.120) | (.140) | (.872) | (.226) | (.576) |
| Wyoming | .651 | .764 | .306 | .015 | -.016 | -.272 | 1.299 | -.479 | -.246 |
|  | .542 | .598 | .637 | .634 | .601 | .706 | .751 | .760 | .647 |
|  | [-.411, 1.712] | [-.408, 1.935] | [-.942, 1.553] | [-1.227, 1.257] | [-1.194, 1.162] | [-1.656, 1.111] | [-.173, 2.772] | [-1.968, 1.010] | [-1.515, 1.022] |
|  | (.230) | (.201) | (.631) | (.981) | (.979) | (.700) | (.084) | (.528) | (.703) |
| N | 2970 | 2974 | 2967 | 2973 | 2973 | 2973 | 2974 | 2975 | 2973 |
| Pseudo-R^2^ |  |  |  |  |  |  |  |  |  |
| Model χ^2^ | 308.3 | 397.8 | 238.9 | 245.4 | 283.6 | 209.5 | 288.5 | 267.0 | 136.3 |
| d.f. | 87 | 87 | 87 | 87 | 87 | 87 | 87 | 87 | 87 |
| p-value | <.001 | <.001 | <.001 | <.001 | <.001 | <.001 | <.001 | <.001 | <.001 |

S14 Table. Regression Results, Health Attitudes, Multilevel Models, Controlling for Deaths.

|  | (1) | (2) | (3) | (4) | (5) | (6) | (7) | (8) | (9) |
| --- | --- | --- | --- | --- | --- | --- | --- | --- | --- |
|  | freetesting | waivecosts | closeschools | canceleverything | paidleave | compensatefirms | nationalguard | stopevents | stateslead |
| main |  |  |  |  |  |  |  |  |  |
| Democrat | .555 | .735 | .459 | .395 | .589 | -.175 | .215 | .500 | -.025 |
|  | .044 | .049 | .052 | .052 | .049 | .057 | .061 | .061 | .052 |
|  | [.468, .641] | [.640, .830] | [.358, .561] | [.294, .496] | [.493, .685] | [-.288, -.063] | [.096, .334] | [.380, .619] | [-.126, .076] |
|  | (<.001) | (<.001) | (<.001) | (<.001) | (<.001) | (.002) | (<.001) | (<.001) | (.628) |
| Other | .257 | .361 | .125 | .018 | .195 | -.356 | -.174 | .117 | -.165 |
|  | .045 | .049 | .053 | .052 | .050 | .058 | .061 | .062 | .052 |
|  | [.170, .345] | [.264, .457] | [.022, .228] | [-.084, .121] | [.097, .292] | [-.470, -.242] | [-.295, -.054] | [-.005, .238] | [-.267, -.062] |
|  | (<.001) | (<.001) | (.017) | (.724) | (<.001) | (<.001) | (.005) | (.060) | (.002) |
| Local COVID-19 Deaths | <.001 | <.001 | <.001 | <.001 | <.001 | <.001 | <.001 | -<.001 | <.001 |
|  | <.001 | <.001 | <.001 | <.001 | <.001 | <.001 | <.001 | <.001 | <.001 |
|  | [-<.001, .000] | [-<.001, .000] | [-<.001, .000] | [-<.001, .000] | [-<.001, .000] | [-<.001, .000] | [-<.001, .000] | [-<.001, -<.001] | [-<.001, .000] |
|  | (.116) | (.328) | (.247) | (.305) | (.310) | (.194) | (.642) | (.020) | (.085) |
| 35- | .144 | .169 | .116 | .147 | .142 | .088 | .024 | .029 | .187 |
|  | .049 | .055 | .058 | .058 | .055 | .064 | .068 | .069 | .058 |
|  | [.047, .241] | [.062, .276] | [.002, .230] | [.034, .261] | [.034, .249] | [-.038, .215] | [-.109, .157] | [-.105, .164] | [.074, .301] |
|  | (.004) | (.002) | (.047) | (.011) | (.010) | (.170) | (.725) | (.669) | (.001) |
| 50- | .154 | .119 | .106 | .144 | .074 | .033 | -.077 | .173 | .180 |
|  | .047 | .052 | .055 | .055 | .052 | .061 | .064 | .065 | .055 |
|  | [.062, .246] | [.018, .220] | [-.002, .213] | [.036, .251] | [-.027, .176] | [-.087, .152] | [-.203, .049] | [.046, .300] | [.072, .287] |
|  | (.001) | (.021) | (.055) | (.009) | (.151) | (.592) | (.232) | (.008) | (.001) |
| 70- | .205 | .013 | .127 | .333 | -.006 | -.040 | .225 | .233 | .311 |
|  | .059 | .065 | .069 | .069 | .065 | .077 | .081 | .082 | .069 |
|  | [.089, .321] | [-.115, .140] | [-.009, .264] | [.197, .468] | [-.134, .123] | [-.190, .111] | [.066, .385] | [.072, .394] | [.175, .447] |
|  | (.001) | (.846) | (.067) | (<.001) | (.931) | (.608) | (.006) | (.005) | (<.001) |
| gender | .105 | .053 | .181 | .233 | .140 | .320 | .193 | .209 | .098 |
|  | .034 | .038 | .040 | .040 | .038 | .044 | .047 | .047 | .040 |
|  | [.039, .172] | [-.020, .127] | [.103, .260] | [.155, .311] | [.066, .214] | [.233, .407] | [.101, .285] | [.116, .301] | [.020, .176] |
|  | (.002) | (.157) | (<.001) | (<.001) | (<.001) | (<.001) | (<.001) | (<.001) | (.014) |
| white | .122 | .160 | .020 | .016 | .143 | -.057 | -.181 | .251 | -.024 |
|  | .041 | .045 | .048 | .048 | .045 | .053 | .056 | .056 | .048 |
|  | [.042, .202] | [.072, .248] | [-.074, .114] | [-.077, .109] | [.054, .231] | [-.161, .046] | [-.291, -.072] | [.141, .362] | [-.117, .070] |
|  | (.003) | (<.001) | (.676) | (.735) | (.002) | (.279) | (.001) | (<.001) | (.620) |
| married | -.044 | -.022 | -.015 | .036 | -.024 | -.006 | .128 | -.062 | .003 |
|  | .037 | .041 | .044 | .044 | .041 | .049 | .051 | .052 | .044 |
|  | [-.118, .029] | [-.103, .058] | [-.101, .071] | [-.049, .122] | [-.105, .058] | [-.102, .089] | [.027, .229] | [-.163, .040] | [-.083, .089] |
|  | (.236) | (.587) | (.736) | (.405) | (.567) | (.897) | (.013) | (.235) | (.946) |
| High school graduate | -.177 | -.159 | -.116 | -.074 | -.219 | -.265 | -.127 | .104 | -.230 |
|  | .100 | .110 | .117 | .117 | .111 | .130 | .137 | .138 | .117 |
|  | [-.372, .018] | [-.375, .058] | [-.345, .114] | [-.303, .156] | [-.437, -.001] | [-.519, -.011] | [-.395, .142] | [-.167, .374] | [-.460, -<.001] |
|  | (.076) | (.151) | (.323) | (.528) | (.049) | (.041) | (.356) | (.454) | (.050) |
| Some college | -.096 | -.076 | -.152 | -.074 | -.124 | -.421 | -.387 | .314 | -.240 |
|  | .102 | .113 | .119 | .119 | .113 | .132 | .140 | .141 | .120 |
|  | [-.295, .103] | [-.297, .145] | [-.387, .082] | [-.307, .160] | [-.346, .098] | [-.680, -.161] | [-.661, -.113] | [.038, .591] | [-.475, -.006] |
|  | (.344) | (.500) | (.202) | (.537) | (.272) | (.001) | (.006) | (.026) | (.045) |
| 2-year | -.068 | -.085 | -.160 | -.063 | -.138 | -.259 | -.320 | .295 | -.249 |
|  | .107 | .118 | .126 | .125 | .119 | .139 | .147 | .148 | .126 |
|  | [-.277, .142] | [-.317, .147] | [-.406, .086] | [-.309, .183] | [-.371, .095] | [-.531, .014] | [-.608, -.032] | [.005, .586] | [-.495, -.003] |
|  | (.526) | (.472) | (.203) | (.617) | (.246) | (.063) | (.029) | (.046) | (.047) |
| 4-year | -.227 | -.225 | -.223 | -.120 | -.233 | -.423 | -.383 | .294 | -.276 |
|  | .104 | .115 | .122 | .122 | .115 | .135 | .143 | .144 | .122 |
|  | [-.430, -.024] | [-.450, -<.001] | [-.462, .016] | [-.359, .118] | [-.459, -.006] | [-.687, -.158] | [-.662, -.103] | [.012, .576] | [-.516, -.037] |
|  | (.029) | (.050) | (.068) | (.323) | (.044) | (.002) | (.007) | (.041) | (.024) |
| Post-grad | -.204 | -.220 | -.302 | -.089 | -.244 | -.522 | -.347 | .375 | -.414 |
|  | .109 | .121 | .128 | .128 | .121 | .142 | .150 | .151 | .128 |
|  | [-.418, .009] | [-.456, .016] | [-.554, -.051] | [-.339, .162] | [-.482, -.007] | [-.800, -.244] | [-.640, -.053] | [.078, .671] | [-.665, -.163] |
|  | (.061) | (.068) | (.018) | (.487) | (.044) | (<.001) | (.021) | (.013) | (.001) |
| income=2 | .120 | .058 | .067 | .050 | -.004 | -.085 | .065 | -.004 | .067 |
|  | .096 | .106 | .113 | .113 | .107 | .125 | .133 | .134 | .113 |
|  | [-.069, .309] | [-.150, .266] | [-.156, .289] | [-.171, .271] | [-.214, .206] | [-.330, .160] | [-.194, .325] | [-.266, .258] | [-.154, .288] |
|  | (.214) | (.585) | (.556) | (.655) | (.970) | (.498) | (.622) | (.975) | (.553) |
| income=3 | .189 | .117 | .117 | .055 | .137 | -.087 | -.027 | -.099 | .228 |
|  | .093 | .103 | .110 | .109 | .104 | .121 | .128 | .129 | .109 |
|  | [.005, .372] | [-.085, .319] | [-.098, .332] | [-.159, .268] | [-.067, .340] | [-.325, .150] | [-.279, .224] | [-.352, .155] | [.013, .442] |
|  | (.044) | (.255) | (.286) | (.615) | (.188) | (.471) | (.832) | (.445) | (.037) |
| income=4 | .217 | .194 | .120 | .147 | .096 | -.156 | .095 | .175 | .147 |
|  | .096 | .106 | .113 | .111 | .106 | .124 | .132 | .133 | .112 |
|  | [.030, .405] | [-.013, .400] | [-.101, .341] | [-.072, .365] | [-.112, .304] | [-.400, .087] | [-.164, .353] | [-.085, .435] | [-.072, .366] |
|  | (.023) | (.067) | (.287) | (.188) | (.366) | (.208) | (.473) | (.187) | (.188) |
| income=5 | .185 | .175 | -.034 | .033 | .092 | -.312 | -.153 | .015 | .092 |
|  | .098 | .108 | .115 | .114 | .108 | .127 | .134 | .135 | .114 |
|  | [-.007, .376] | [-.036, .386] | [-.259, .192] | [-.190, .256] | [-.120, .304] | [-.561, -.063] | [-.416, .110] | [-.250, .280] | [-.132, .316] |
|  | (.059) | (.104) | (.770) | (.774) | (.396) | (.014) | (.256) | (.914) | (.423) |
| income=6 | .172 | .104 | .140 | .140 | .122 | -.137 | .101 | .102 | .158 |
|  | .099 | .109 | .116 | .115 | .109 | .128 | .136 | .137 | .115 |
|  | [-.022, .365] | [-.109, .317] | [-.087, .368] | [-.086, .366] | [-.093, .336] | [-.387, .114] | [-.165, .367] | [-.166, .369] | [-.068, .384] |
|  | (.082) | (.339) | (.226) | (.226) | (.267) | (.286) | (.455) | (.458) | (.172) |
| income=7 | .240 | .025 | .239 | .218 | .120 | .021 | .038 | .255 | .029 |
|  | .106 | .116 | .125 | .123 | .118 | .137 | .146 | .147 | .124 |
|  | [.033, .447] | [-.203, .253] | [-.005, .484] | [-.024, .459] | [-.110, .351] | [-.248, .289] | [-.247, .323] | [-.033, .542] | [-.213, .271] |
|  | (.023) | (.827) | (.055) | (.078) | (.307) | (.880) | (.793) | (.082) | (.815) |
| income=8 | .197 | .091 | .088 | .054 | .208 | -.054 | -.140 | .041 | .089 |
|  | .103 | .113 | .121 | .120 | .114 | .134 | .142 | .143 | .120 |
|  | [-.005, .399] | [-.131, .313] | [-.149, .325] | [-.181, .290] | [-.015, .432] | [-.316, .208] | [-.417, .138] | [-.239, .320] | [-.147, .325] |
|  | (.055) | (.421) | (.467) | (.650) | (.068) | (.688) | (.324) | (.775) | (.461) |
| income=9 | .010 | -.138 | .070 | -.019 | .028 | -.170 | -.247 | .205 | .038 |
|  | .102 | .112 | .120 | .119 | .113 | .132 | .140 | .141 | .119 |
|  | [-.190, .210] | [-.358, .082] | [-.164, .305] | [-.252, .214] | [-.193, .249] | [-.429, .089] | [-.521, .027] | [-.072, .481] | [-.195, .271] |
|  | (.920) | (.218) | (.557) | (.873) | (.802) | (.198) | (.077) | (.146) | (.750) |
| income=10 | .185 | .039 | .021 | .153 | .123 | -.074 | -.087 | .203 | .070 |
|  | .106 | .117 | .124 | .123 | .117 | .137 | .145 | .147 | .124 |
|  | [-.022, .392] | [-.190, .267] | [-.223, .265] | [-.089, .395] | [-.107, .353] | [-.343, .195] | [-.372, .198] | [-.084, .490] | [-.172, .313] |
|  | (.080) | (.739) | (.866) | (.216) | (.295) | (.589) | (.551) | (.166) | (.571) |
| income=11 | .214 | -.052 | .186 | .226 | .011 | -.089 | -.131 | .267 | .107 |
|  | .109 | .120 | .128 | .127 | .120 | .141 | .149 | .150 | .127 |
|  | [.001, .427] | [-.287, .183] | [-.064, .437] | [-.023, .474] | [-.225, .246] | [-.365, .188] | [-.424, .161] | [-.028, .562] | [-.142, .356] |
|  | (.049) | (.664) | (.145) | (.075) | (.930) | (.529) | (.380) | (.076) | (.401) |
| income=12 | .072 | -.127 | .069 | .105 | .002 | -.106 | -.276 | .344 | .085 |
|  | .120 | .132 | .141 | .140 | .132 | .155 | .165 | .166 | .140 |
|  | [-.163, .306] | [-.384, .131] | [-.207, .346] | [-.169, .379] | [-.258, .261] | [-.410, .198] | [-.598, .047] | [.019, .669] | [-.189, .359] |
|  | (.549) | (.336) | (.623) | (.452) | (.990) | (.495) | (.094) | (.038) | (.542) |
| income=13 | .389 | .186 | .120 | .337 | .163 | -.390 | -.071 | .369 | .040 |
|  | .165 | .182 | .196 | .195 | .183 | .214 | .227 | .229 | .193 |
|  | [.066, .712] | [-.171, .542] | [-.264, .504] | [-.045, .719] | [-.195, .521] | [-.810, .030] | [-.516, .373] | [-.080, .817] | [-.339, .418] |
|  | (.018) | (.307) | (.540) | (.083) | (.373) | (.069) | (.754) | (.107) | (.837) |
| income=14 | -.129 | -.191 | -.272 | -.016 | -.101 | -.247 | -.516 | .294 | -.281 |
|  | .175 | .193 | .205 | .204 | .194 | .227 | .240 | .242 | .204 |
|  | [-.471, .213] | [-.569, .186] | [-.674, .131] | [-.416, .384] | [-.480, .279] | [-.692, .198] | [-.986, -.045] | [-.181, .769] | [-.682, .120] |
|  | (.461) | (.321) | (.186) | (.937) | (.603) | (.277) | (.032) | (.225) | (.169) |
| income=15 | .715 | .768 | .031 | .307 | .506 | .194 | 1.435 | .808 | 1.263 |
|  | .528 | .583 | .621 | .618 | .586 | .688 | .727 | .733 | .619 |
|  | [-.321, 1.751] | [-.374, 1.911] | [-1.186, 1.249] | [-.904, 1.519] | [-.643, 1.654] | [-1.154, 1.543] | [.011, 2.860] | [-.629, 2.245] | [.051, 2.475] |
|  | (.176) | (.187) | (.960) | (.619) | (.388) | (.777) | (.048) | (.270) | (.041) |
| income=16 | .409 | .451 | .412 | .474 | .564 | -.020 | .105 | -.035 | .152 |
|  | .248 | .274 | .292 | .290 | .275 | .323 | .342 | .345 | .291 |
|  | [-.078, .896] | [-.086, .988] | [-.161, .984] | [-.095, 1.043] | [.024, 1.103] | [-.653, .614] | [-.565, .775] | [-.710, .641] | [-.418, .722] |
|  | (.100) | (.100) | (.159) | (.103) | (.041) | (.951) | (.758) | (.920) | (.602) |
| income=17 | .072 | .001 | -.006 | .006 | -.035 | -.183 | -.334 | .184 | .102 |
|  | .091 | .100 | .107 | .106 | .100 | .117 | .125 | .126 | .106 |
|  | [-.106, .250] | [-.194, .197] | [-.215, .202] | [-.201, .212] | [-.232, .161] | [-.414, .047] | [-.578, -.089] | [-.062, .430] | [-.106, .309] |
|  | (.428) | (.989) | (.952) | (.958) | (.724) | (.118) | (.007) | (.142) | (.338) |
| Rural Code=2 | -.038 | -.047 | -.102 | -.075 | .007 | -.090 | -.117 | -.026 | -.014 |
|  | .047 | .051 | .055 | .054 | .052 | .061 | .064 | .065 | .055 |
|  | [-.129, .053] | [-.147, .054] | [-.209, .006] | [-.181, .032] | [-.094, .108] | [-.209, .029] | [-.243, .009] | [-.153, .101] | [-.122, .094] |
|  | (.413) | (.364) | (.064) | (.171) | (.886) | (.137) | (.068) | (.686) | (.796) |
| Rural Code=3 | -.077 | -.188 | -.145 | -.029 | -.016 | -.004 | -.041 | -.005 | -.041 |
|  | .063 | .069 | .074 | .073 | .070 | .082 | .087 | .087 | .074 |
|  | [-.200, .046] | [-.323, -.053] | [-.289, -<.001] | [-.173, .114] | [-.152, .120] | [-.165, .156] | [-.210, .129] | [-.176, .166] | [-.186, .105] |
|  | (.218) | (.007) | (.050) | (.689) | (.816) | (.957) | (.640) | (.956) | (.583) |
| Rural Code=4 | -.029 | .053 | -.228 | -.049 | .100 | -.029 | -.140 | -.188 | .084 |
|  | .087 | .095 | .102 | .101 | .096 | .113 | .119 | .120 | .102 |
|  | [-.198, .141] | [-.134, .240] | [-.427, -.029] | [-.248, .149] | [-.088, .288] | [-.249, .192] | [-.374, .094] | [-.424, .048] | [-.116, .284] |
|  | (.741) | (.577) | (.025) | (.627) | (.297) | (.800) | (.241) | (.118) | (.411) |
| Rural Code=5 | .007 | -.092 | .092 | .107 | .136 | -.068 | .027 | .263 | -.088 |
|  | .132 | .145 | .155 | .154 | .146 | .172 | .182 | .183 | .155 |
|  | [-.251, .265] | [-.377, .193] | [-.211, .396] | [-.195, .409] | [-.150, .422] | [-.404, .269] | [-.329, .383] | [-.096, .622] | [-.392, .216] |
|  | (.958) | (.526) | (.551) | (.488) | (.352) | (.693) | (.881) | (.152) | (.570) |
| Rural Code=6 | .015 | -.026 | -.090 | -.125 | .053 | .023 | .024 | -.119 | -.203 |
|  | .086 | .095 | .101 | .100 | .095 | .112 | .118 | .119 | .101 |
|  | [-.154, .183] | [-.212, .160] | [-.287, .108] | [-.322, .072] | [-.134, .239] | [-.196, .242] | [-.208, .256] | [-.353, .115] | [-.402, -.005] |
|  | (.865) | (.783) | (.375) | (.213) | (.578) | (.836) | (.839) | (.317) | (.044) |
| Rural Code=7 | -.182 | -.087 | -.314 | -.166 | -.197 | -.141 | -.105 | -.263 | -.196 |
|  | .119 | .130 | .139 | .138 | .131 | .153 | .162 | .164 | .140 |
|  | [-.415, .050] | [-.342, .168] | [-.587, -.040] | [-.436, .105] | [-.453, .059] | [-.442, .160] | [-.424, .213] | [-.584, .058] | [-.469, .078] |
|  | (.124) | (.503) | (.024) | (.230) | (.131) | (.358) | (.517) | (.109) | (.161) |
| Rural Code=8 | -.038 | -.103 | -.223 | -.393 | -.002 | .126 | -.716 | -.078 | .460 |
|  | .220 | .243 | .259 | .258 | .250 | .287 | .311 | .305 | .258 |
|  | [-.469, .394] | [-.579, .373] | [-.730, .284] | [-.898, .112] | [-.493, .489] | [-.436, .688] | [-1.325, -.107] | [-.677, .521] | [-.046, .966] |
|  | (.864) | (.671) | (.389) | (.127) | (.994) | (.660) | (.021) | (.798) | (.074) |
| Rural Code=9 | .006 | .078 | .092 | -.041 | .066 | -.414 | .056 | .003 | -.002 |
|  | .187 | .206 | .220 | .219 | .207 | .243 | .257 | .259 | .219 |
|  | [-.360, .372] | [-.326, .482] | [-.338, .523] | [-.469, .388] | [-.340, .472] | [-.891, .063] | [-.449, .560] | [-.506, .511] | [-.433, .428] |
|  | (.976) | (.706) | (.674) | (.852) | (.749) | (.089) | (.828) | (.992) | (.991) |
| Alaska | -<.001 | -.032 | .296 | .254 | .013 | .384 | .147 | .354 | -.020 |
|  | .374 | .412 | .439 | .437 | .415 | .487 | .514 | .519 | .439 |
|  | [-.732, .732] | [-.840, .776] | [-.565, 1.156] | [-.602, 1.111] | [-.800, .826] | [-.571, 1.339] | [-.860, 1.154] | [-.664, 1.372] | [-.880, .840] |
|  | (.999) | (.938) | (.500) | (.561) | (.975) | (.431) | (.775) | (.496) | (.964) |
| Arizona | -.213 | -.077 | -.012 | -.123 | -.066 | -.281 | -.320 | .084 | -.277 |
|  | .178 | .196 | .210 | .208 | .199 | .233 | .245 | .249 | .211 |
|  | [-.561, .135] | [-.461, .307] | [-.423, .399] | [-.531, .284] | [-.457, .324] | [-.739, .176] | [-.800, .160] | [-.405, .573] | [-.691, .136] |
|  | (.231) | (.696) | (.954) | (.553) | (.739) | (.228) | (.191) | (.737) | (.189) |
| Arkansas | -.177 | -.151 | -.075 | -.107 | -.134 | -.264 | -.055 | .134 | -.122 |
|  | .200 | .220 | .235 | .234 | .223 | .262 | .275 | .279 | .236 |
|  | [-.568, .215] | [-.583, .280] | [-.535, .385] | [-.565, .351] | [-.570, .303] | [-.777, .248] | [-.594, .485] | [-.413, .682] | [-.585, .341] |
|  | (.376) | (.492) | (.750) | (.647) | (.549) | (.312) | (.842) | (.631) | (.607) |
| California | -.097 | .082 | .057 | -.053 | .012 | -.270 | .121 | .305 | -.210 |
|  | .157 | .173 | .185 | .184 | .176 | .207 | .216 | .221 | .186 |
|  | [-.405, .210] | [-.258, .422] | [-.305, .419] | [-.413, .307] | [-.333, .356] | [-.675, .135] | [-.303, .545] | [-.127, .738] | [-.575, .155] |
|  | (.535) | (.636) | (.756) | (.772) | (.947) | (.192) | (.577) | (.167) | (.259) |
| Colorado | -.104 | -.339 | -.100 | -.067 | -.143 | -.232 | .055 | .303 | -.145 |
|  | .201 | .222 | .236 | .235 | .224 | .263 | .277 | .281 | .238 |
|  | [-.497, .289] | [-.775, .097] | [-.562, .362] | [-.527, .393] | [-.582, .296] | [-.748, .284] | [-.488, .597] | [-.248, .854] | [-.611, .321] |
|  | (.605) | (.127) | (.671) | (.776) | (.523) | (.378) | (.844) | (.281) | (.541) |
| Connecticut | .073 | .279 | .486 | .415 | .073 | .112 | .239 | .277 | .172 |
|  | .219 | .242 | .258 | .257 | .244 | .287 | .303 | .307 | .260 |
|  | [-.357, .503] | [-.196, .753] | [-.019, .992] | [-.088, .918] | [-.406, .552] | [-.451, .675] | [-.354, .832] | [-.324, .878] | [-.337, .680] |
|  | (.738) | (.249) | (.059) | (.106) | (.766) | (.697) | (.429) | (.367) | (.508) |
| Delaware | -.323 | .189 | .111 | .129 | -.343 | -.624 | .304 | -.035 | -.288 |
|  | .282 | .311 | .331 | .330 | .313 | .368 | .389 | .394 | .343 |
|  | [-.875, .230] | [-.421, .798] | [-.539, .760] | [-.517, .775] | [-.958, .271] | [-1.345, .098] | [-.458, 1.067] | [-.807, .736] | [-.960, .384] |
|  | (.252) | (.544) | (.738) | (.696) | (.273) | (.090) | (.434) | (.929) | (.401) |
| District of Columbia | -.638 | -.089 | .124 | -.215 | -.615 | .063 | .499 | .568 | .123 |
|  | .352 | .389 | .438 | .412 | .391 | .460 | .485 | .490 | .438 |
|  | [-1.329, .052] | [-.850, .672] | [-.734, .983] | [-1.023, .592] | [-1.382, .152] | [-.838, .964] | [-.451, 1.448] | [-.392, 1.529] | [-.735, .981] |
|  | (.070) | (.819) | (.776) | (.601) | (.116) | (.891) | (.304) | (.246) | (.779) |
| Florida | -.126 | -.063 | .173 | -.079 | -.097 | -.152 | .247 | .278 | -.048 |
|  | .158 | .175 | .186 | .185 | .177 | .209 | .218 | .222 | .188 |
|  | [-.437, .184] | [-.405, .280] | [-.191, .538] | [-.442, .284] | [-.445, .250] | [-.561, .257] | [-.181, .674] | [-.158, .714] | [-.416, .320] |
|  | (.425) | (.719) | (.351) | (.669) | (.584) | (.466) | (.258) | (.212) | (.796) |
| Georgia | -.195 | -.300 | .033 | -.214 | -.054 | -.335 | .199 | .023 | -.132 |
|  | .171 | .189 | .202 | .201 | .192 | .225 | .236 | .240 | .203 |
|  | [-.531, .141] | [-.671, .070] | [-.363, .428] | [-.608, .179] | [-.430, .322] | [-.776, .107] | [-.264, .662] | [-.448, .494] | [-.530, .266] |
|  | (.255) | (.112) | (.871) | (.285) | (.780) | (.137) | (.399) | (.923) | (.516) |
| Hawaii | .257 | .596 | .615 | .362 | .090 | -.133 | .419 | -.014 | .038 |
|  | .303 | .334 | .356 | .355 | .337 | .396 | .419 | .423 | .359 |
|  | [-.338, .851] | [-.059, 1.252] | [-.083, 1.314] | [-.333, 1.058] | [-.570, .751] | [-.909, .643] | [-.401, 1.240] | [-.844, .815] | [-.666, .741] |
|  | (.397) | (.075) | (.084) | (.307) | (.789) | (.737) | (.317) | (.973) | (.917) |
| Idaho | -.364 | -.409 | .201 | -.310 | -.488 | -.340 | -.505 | .350 | -.287 |
|  | .254 | .280 | .298 | .297 | .283 | .332 | .351 | .355 | .301 |
|  | [-.862, .133] | [-.957, .140] | [-.384, .786] | [-.892, .272] | [-1.042, .066] | [-.990, .311] | [-1.193, .182] | [-.346, 1.046] | [-.877, .304] |
|  | (.151) | (.145) | (.500) | (.297) | (.084) | (.306) | (.150) | (.324) | (.341) |
| Illinois | -.167 | -.134 | .098 | .005 | -.018 | -.282 | .180 | .408 | -.137 |
|  | .173 | .190 | .203 | .202 | .193 | .227 | .238 | .242 | .204 |
|  | [-.506, .171] | [-.507, .239] | [-.300, .495] | [-.390, .401] | [-.396, .360] | [-.726, .162] | [-.286, .646] | [-.066, .882] | [-.538, .263] |
|  | (.332) | (.482) | (.630) | (.980) | (.927) | (.213) | (.449) | (.092) | (.502) |
| Indiana | .010 | .257 | .484 | .220 | .012 | -.241 | .075 | .673 | -.199 |
|  | .187 | .206 | .220 | .218 | .209 | .245 | .258 | .261 | .222 |
|  | [-.356, .376] | [-.147, .661] | [.052, .915] | [-.208, .648] | [-.396, .421] | [-.721, .239] | [-.431, .580] | [.160, 1.185] | [-.633, .235] |
|  | (.958) | (.212) | (.028) | (.313) | (.953) | (.325) | (.771) | (.010) | (.369) |
| Iowa | -.194 | -.196 | .023 | .050 | -.110 | .120 | .326 | .574 | -.052 |
|  | .222 | .245 | .261 | .260 | .247 | .291 | .305 | .310 | .262 |
|  | [-.629, .241] | [-.676, .284] | [-.489, .534] | [-.459, .559] | [-.595, .375] | [-.450, .689] | [-.272, .925] | [-.033, 1.181] | [-.564, .461] |
|  | (.383) | (.423) | (.931) | (.848) | (.657) | (.680) | (.285) | (.064) | (.844) |
| Kansas | -.018 | .158 | .017 | .346 | .139 | -.199 | .153 | .564 | -.171 |
|  | .268 | .296 | .316 | .314 | .299 | .351 | .370 | .375 | .317 |
|  | [-.544, .509] | [-.423, .738] | [-.601, .636] | [-.270, .962] | [-.447, .724] | [-.887, .489] | [-.572, .878] | [-.170, 1.298] | [-.791, .450] |
|  | (.948) | (.594) | (.956) | (.271) | (.643) | (.571) | (.679) | (.132) | (.590) |
| Kentucky | -.352 | -.110 | .053 | -.289 | -.058 | .109 | -.177 | .253 | -.072 |
|  | .203 | .224 | .239 | .238 | .228 | .266 | .281 | .284 | .240 |
|  | [-.750, .046] | [-.549, .329] | [-.415, .521] | [-.755, .177] | [-.504, .388] | [-.413, .631] | [-.728, .374] | [-.304, .810] | [-.543, .399] |
|  | (.083) | (.625) | (.824) | (.224) | (.799) | (.682) | (.529) | (.373) | (.764) |
| Louisiana | -.180 | .189 | .067 | -.176 | -.092 | -.222 | .127 | .135 | -.141 |
|  | .204 | .225 | .239 | .238 | .227 | .267 | .281 | .285 | .241 |
|  | [-.580, .219] | [-.251, .630] | [-.402, .536] | [-.643, .291] | [-.538, .353] | [-.745, .301] | [-.424, .677] | [-.424, .694] | [-.614, .332] |
|  | (.376) | (.400) | (.779) | (.461) | (.685) | (.405) | (.652) | (.636) | (.559) |
| Maine | -.154 | -.031 | .742 | .370 | .098 | -.113 | -.364 | .302 | .307 |
|  | .282 | .311 | .332 | .330 | .314 | .369 | .389 | .394 | .334 |
|  | [-.707, .399] | [-.642, .579] | [.092, 1.392] | [-.277, 1.017] | [-.518, .713] | [-.835, .610] | [-1.127, .400] | [-.470, 1.074] | [-.347, .961] |
|  | (.585) | (.920) | (.025) | (.262) | (.756) | (.760) | (.351) | (.443) | (.358) |
| Maryland | -.117 | -.010 | -.032 | -.184 | .164 | -.237 | .274 | .137 | -.057 |
|  | .202 | .223 | .237 | .236 | .225 | .265 | .278 | .282 | .239 |
|  | [-.512, .278] | [-.446, .427] | [-.497, .433] | [-.646, .279] | [-.277, .605] | [-.755, .282] | [-.271, .819] | [-.416, .691] | [-.525, .411] |
|  | (.562) | (.965) | (.893) | (.437) | (.466) | (.371) | (.324) | (.627) | (.811) |
| Massachusetts | -.073 | .064 | -.051 | -.060 | .042 | -.164 | .079 | .542 | -.357 |
|  | .190 | .210 | .224 | .223 | .212 | .250 | .262 | .267 | .225 |
|  | [-.446, .300] | [-.348, .475] | [-.490, .387] | [-.496, .376] | [-.374, .459] | [-.654, .325] | [-.435, .593] | [.020, 1.065] | [-.799, .085] |
|  | (.702) | (.762) | (.819) | (.787) | (.842) | (.510) | (.763) | (.042) | (.113) |
| Michigan | -.184 | -.020 | .216 | -.205 | -.180 | -.208 | .013 | .430 | -.122 |
|  | .170 | .188 | .200 | .199 | .190 | .224 | .234 | .239 | .202 |
|  | [-.518, .149] | [-.388, .348] | [-.176, .608] | [-.595, .185] | [-.553, .193] | [-.647, .230] | [-.447, .472] | [-.038, .899] | [-.517, .273] |
|  | (.279) | (.914) | (.280) | (.303) | (.345) | (.352) | (.957) | (.072) | (.546) |
| Minnesota | -.318 | -.172 | .124 | -.009 | -.227 | -.191 | .038 | .188 | .039 |
|  | .199 | .220 | .234 | .233 | .223 | .262 | .275 | .279 | .236 |
|  | [-.709, .073] | [-.603, .259] | [-.335, .584] | [-.467, .448] | [-.663, .209] | [-.704, .322] | [-.500, .577] | [-.359, .735] | [-.423, .501] |
|  | (.111) | (.435) | (.596) | (.968) | (.308) | (.465) | (.889) | (.500) | (.868) |
| Mississippi | -.397 | -.308 | .137 | -.029 | -.092 | .067 | .269 | .073 | .068 |
|  | .226 | .249 | .265 | .264 | .252 | .295 | .311 | .315 | .266 |
|  | [-.839, .046] | [-.796, .180] | [-.383, .657] | [-.546, .489] | [-.585, .401] | [-.512, .646] | [-.340, .878] | [-.545, .690] | [-.454, .590] |
|  | (.079) | (.216) | (.605) | (.914) | (.715) | (.820) | (.387) | (.818) | (.799) |
| Missouri | -.314 | -.162 | -.050 | -.285 | -.036 | -.309 | .097 | .127 | -.084 |
|  | .184 | .202 | .215 | .214 | .204 | .241 | .252 | .257 | .217 |
|  | [-.674, .046] | [-.558, .234] | [-.471, .371] | [-.705, .134] | [-.436, .365] | [-.780, .163] | [-.397, .590] | [-.377, .630] | [-.510, .341] |
|  | (.088) | (.423) | (.815) | (.182) | (.861) | (.200) | (.701) | (.622) | (.698) |
| Montana | -.726 | -.694 | -.188 | -.567 | -.126 | -.817 | -.329 | -.381 | -.514 |
|  | .282 | .311 | .331 | .330 | .313 | .368 | .389 | .393 | .333 |
|  | [-1.278, -.174] | [-1.303, -.085] | [-.837, .461] | [-1.213, .079] | [-.740, .488] | [-1.538, -.096] | [-1.091, .432] | [-1.152, .390] | [-1.166, .139] |
|  | (.010) | (.025) | (.570) | (.085) | (.688) | (.026) | (.397) | (.333) | (.123) |
| Nebraska | -.091 | .236 | -.394 | -.176 | .008 | .035 | .438 | -.073 | -.099 |
|  | .299 | .320 | .341 | .340 | .323 | .390 | .399 | .404 | .341 |
|  | [-.677, .495] | [-.391, .864] | [-1.063, .275] | [-.842, .489] | [-.625, .641] | [-.731, .800] | [-.345, 1.221] | [-.865, .719] | [-.768, .570] |
|  | (.761) | (.460) | (.248) | (.603) | (.979) | (.929) | (.273) | (.857) | (.772) |
| Nevada | -.041 | .063 | .187 | .002 | -.144 | -.373 | -.178 | .398 | -<.001 |
|  | .199 | .219 | .234 | .233 | .223 | .261 | .274 | .279 | .236 |
|  | [-.430, .349] | [-.367, .493] | [-.271, .645] | [-.454, .458] | [-.582, .293] | [-.884, .138] | [-.715, .360] | [-.148, .944] | [-.463, .462] |
|  | (.837) | (.774) | (.423) | (.993) | (.517) | (.152) | (.517) | (.153) | (.999) |
| New Hampshire | .005 | .209 | -.033 | -.153 | .028 | -.271 | .345 | .034 | .037 |
|  | .239 | .263 | .280 | .279 | .266 | .312 | .329 | .334 | .283 |
|  | [-.462, .473] | [-.306, .725] | [-.582, .517] | [-.700, .394] | [-.492, .549] | [-.883, .341] | [-.300, .990] | [-.620, .688] | [-.517, .590] |
|  | (.982) | (.426) | (.907) | (.584) | (.915) | (.385) | (.295) | (.919) | (.897) |
| New Jersey | -.119 | .178 | .178 | -.055 | .012 | .005 | .382 | .240 | -.057 |
|  | .185 | .204 | .218 | .217 | .206 | .243 | .255 | .259 | .218 |
|  | [-.482, .244] | [-.222, .578] | [-.250, .606] | [-.480, .369] | [-.392, .417] | [-.471, .480] | [-.117, .882] | [-.268, .749] | [-.484, .371] |
|  | (.521) | (.384) | (.415) | (.798) | (.953) | (.984) | (.134) | (.354) | (.794) |
| New Mexico | .141 | -.149 | .264 | -.135 | -.143 | -.345 | -.407 | .299 | -.355 |
|  | .220 | .242 | .258 | .257 | .245 | .288 | .303 | .307 | .260 |
|  | [-.290, .571] | [-.624, .326] | [-.242, .771] | [-.639, .369] | [-.624, .337] | [-.909, .219] | [-1.001, .187] | [-.303, .902] | [-.865, .155] |
|  | (.523) | (.539) | (.306) | (.599) | (.558) | (.230) | (.179) | (.330) | (.172) |
| New York | -.290 | -.025 | .097 | -.112 | -.104 | -.194 | .205 | .035 | -.064 |
|  | .162 | .178 | .190 | .189 | .181 | .213 | .223 | .227 | .191 |
|  | [-.607, .027] | [-.375, .324] | [-.275, .470] | [-.483, .258] | [-.458, .251] | [-.611, .222] | [-.231, .641] | [-.410, .479] | [-.439, .311] |
|  | (.073) | (.887) | (.608) | (.552) | (.567) | (.361) | (.358) | (.879) | (.739) |
| North Carolina | -.122 | .088 | .064 | .096 | -.051 | -.376 | .258 | .064 | -.258 |
|  | .174 | .192 | .205 | .204 | .195 | .230 | .240 | .244 | .207 |
|  | [-.463, .219] | [-.288, .465] | [-.338, .466] | [-.304, .496] | [-.433, .331] | [-.826, .074] | [-.212, .728] | [-.415, .542] | [-.663, .147] |
|  | (.483) | (.646) | (.755) | (.638) | (.794) | (.101) | (.283) | (.794) | (.212) |
| North Dakota | -.192 | .426 | -.368 | -.260 | .196 | -.174 | .543 | -.089 | -.490 |
|  | .353 | .390 | .415 | .414 | .393 | .461 | .488 | .493 | .418 |
|  | [-.884, .501] | [-.338, 1.190] | [-1.182, .446] | [-1.070, .551] | [-.574, .965] | [-1.078, .730] | [-.413, 1.499] | [-1.055, .877] | [-1.308, .329] |
|  | (.588) | (.275) | (.376) | (.530) | (.619) | (.706) | (.265) | (.857) | (.241) |
| Ohio | -.194 | -.020 | .163 | -.012 | -.027 | -.077 | -.024 | .333 | .077 |
|  | .169 | .187 | .199 | .198 | .189 | .223 | .233 | .238 | .201 |
|  | [-.526, .137] | [-.386, .346] | [-.227, .553] | [-.400, .377] | [-.398, .344] | [-.513, .359] | [-.481, .433] | [-.132, .799] | [-.316, .470] |
|  | (.251) | (.913) | (.412) | (.952) | (.887) | (.730) | (.917) | (.160) | (.701) |
| Oklahoma | -.265 | .001 | .205 | .176 | -.146 | .240 | .416 | .010 | -.223 |
|  | .227 | .250 | .267 | .266 | .253 | .297 | .313 | .317 | .268 |
|  | [-.710, .180] | [-.490, .491] | [-.318, .728] | [-.345, .697] | [-.642, .349] | [-.342, .823] | [-.197, 1.029] | [-.611, .631] | [-.748, .301] |
|  | (.242) | (.998) | (.443) | (.508) | (.563) | (.419) | (.183) | (.975) | (.404) |
| Oregon | .044 | .138 | -.274 | -.132 | -.026 | -.331 | -.070 | .234 | -.381 |
|  | .188 | .207 | .220 | .219 | .209 | .246 | .258 | .263 | .222 |
|  | [-.324, .412] | [-.267, .543] | [-.706, .157] | [-.562, .297] | [-.436, .384] | [-.813, .151] | [-.576, .437] | [-.281, .749] | [-.817, .055] |
|  | (.815) | (.505) | (.213) | (.546) | (.901) | (.178) | (.787) | (.373) | (.087) |
| Pennsylvania | -.129 | -.041 | .085 | -.036 | -.004 | -.184 | .006 | .333 | -.024 |
|  | .161 | .177 | .189 | .188 | .180 | .211 | .221 | .225 | .190 |
|  | [-.443, .186] | [-.388, .306] | [-.285, .455] | [-.404, .332] | [-.356, .348] | [-.598, .230] | [-.427, .439] | [-.109, .774] | [-.397, .349] |
|  | (.423) | (.816) | (.653) | (.848) | (.981) | (.383) | (.979) | (.139) | (.901) |
| Rhode Island | .340 | .310 | .314 | -.032 | .278 | .842 | .043 | -.131 | -.018 |
|  | .352 | .388 | .413 | .411 | .391 | .459 | .484 | .489 | .413 |
|  | [-.349, 1.029] | [-.451, 1.070] | [-.496, 1.125] | [-.839, .774] | [-.487, 1.044] | [-.058, 1.741] | [-.906, .991] | [-1.090, .828] | [-.828, .792] |
|  | (.333) | (.425) | (.447) | (.938) | (.476) | (.067) | (.929) | (.789) | (.965) |
| South Carolina | -.126 | -.119 | .271 | .165 | -.318 | -.117 | .021 | .385 | -.025 |
|  | .196 | .217 | .231 | .230 | .219 | .257 | .271 | .275 | .233 |
|  | [-.511, .259] | [-.544, .306] | [-.181, .724] | [-.285, .616] | [-.748, .112] | [-.622, .387] | [-.510, .552] | [-.154, .924] | [-.482, .431] |
|  | (.521) | (.583) | (.240) | (.472) | (.147) | (.649) | (.937) | (.162) | (.913) |
| South Dakota | -.278 | -.145 | -.510 | -.242 | -.233 | -.220 | .236 | -.379 | -.061 |
|  | .325 | .359 | .382 | .380 | .361 | .424 | .448 | .453 | .384 |
|  | [-.916, .359] | [-.849, .558] | [-1.259, .239] | [-.988, .503] | [-.941, .475] | [-1.051, .612] | [-.643, 1.114] | [-1.267, .510] | [-.813, .691] |
|  | (.392) | (.685) | (.182) | (.524) | (.519) | (.605) | (.599) | (.403) | (.875) |
| Tennessee | .039 | .184 | .028 | -.283 | -.085 | -.204 | -.105 | .312 | -.229 |
|  | .192 | .212 | .226 | .225 | .214 | .252 | .265 | .269 | .227 |
|  | [-.338, .415] | [-.231, .599] | [-.414, .470] | [-.723, .158] | [-.505, .336] | [-.697, .290] | [-.624, .414] | [-.215, .839] | [-.675, .217] |
|  | (.841) | (.385) | (.901) | (.208) | (.693) | (.419) | (.692) | (.246) | (.314) |
| Texas | -.167 | -.090 | -.007 | -.102 | -.234 | -.272 | -.004 | .073 | -.081 |
|  | .161 | .177 | .189 | .188 | .180 | .211 | .221 | .225 | .190 |
|  | [-.482, .148] | [-.437, .258] | [-.377, .363] | [-.470, .266] | [-.586, .119] | [-.687, .142] | [-.437, .430] | [-.368, .515] | [-.454, .292] |
|  | (.298) | (.613) | (.970) | (.588) | (.194) | (.198) | (.987) | (.745) | (.670) |
| Utah | -.204 | -.017 | -.065 | .029 | -.059 | -.449 | .224 | .515 | -.296 |
|  | .222 | .245 | .261 | .260 | .248 | .291 | .307 | .311 | .264 |
|  | [-.639, .232] | [-.498, .464] | [-.577, .448] | [-.481, .539] | [-.545, .427] | [-1.020, .121] | [-.378, .826] | [-.095, 1.126] | [-.813, .221] |
|  | (.360) | (.944) | (.805) | (.911) | (.811) | (.123) | (.465) | (.098) | (.262) |
| Vermont | .015 | -.073 | .316 | .308 | -.381 | -.290 | .262 | .673 | -.049 |
|  | .322 | .355 | .378 | .376 | .358 | .420 | .443 | .448 | .378 |
|  | [-.616, .645] | [-.768, .623] | [-.425, 1.058] | [-.430, 1.045] | [-1.082, .320] | [-1.113, .533] | [-.606, 1.130] | [-.204, 1.550] | [-.790, .693] |
|  | (.964) | (.838) | (.403) | (.414) | (.286) | (.490) | (.554) | (.133) | (.897) |
| Virginia | -.089 | -.036 | .008 | -.120 | -.061 | -.364 | .089 | .289 | .027 |
|  | .172 | .190 | .203 | .202 | .193 | .226 | .238 | .242 | .204 |
|  | [-.426, .248] | [-.408, .336] | [-.389, .405] | [-.515, .275] | [-.438, .317] | [-.808, .079] | [-.376, .555] | [-.185, .762] | [-.373, .427] |
|  | (.605) | (.850) | (.968) | (.551) | (.753) | (.108) | (.707) | (.232) | (.894) |
| Washington | -.108 | -.012 | -.105 | -.150 | .028 | -.332 | -.210 | .305 | -.278 |
|  | .181 | .199 | .212 | .211 | .202 | .237 | .249 | .254 | .214 |
|  | [-.462, .246] | [-.402, .378] | [-.520, .311] | [-.564, .263] | [-.367, .423] | [-.796, .132] | [-.697, .277] | [-.192, .803] | [-.697, .141] |
|  | (.549) | (.951) | (.621) | (.477) | (.891) | (.161) | (.398) | (.228) | (.194) |
| West Virginia | -.643 | -.077 | .288 | -.038 | -.196 | -.319 | .144 | .398 | .022 |
|  | .230 | .253 | .273 | .269 | .256 | .300 | .316 | .321 | .274 |
|  | [-1.093, -.193] | [-.574, .419] | [-.247, .824] | [-.564, .489] | [-.698, .306] | [-.908, .269] | [-.476, .763] | [-.230, 1.026] | [-.516, .560] |
|  | (.005) | (.760) | (.292) | (.889) | (.444) | (.288) | (.650) | (.215) | (.936) |
| Wisconsin | -.337 | -.080 | .076 | -.094 | -.322 | -.359 | .042 | .311 | -.121 |
|  | .186 | .206 | .219 | .218 | .208 | .245 | .257 | .261 | .221 |
|  | [-.702, .028] | [-.485, .325] | [-.353, .506] | [-.522, .334] | [-.730, .086] | [-.839, .121] | [-.462, .546] | [-.201, .823] | [-.553, .312] |
|  | (.071) | (.698) | (.728) | (.667) | (.122) | (.143) | (.870) | (.233) | (.585) |
| Wyoming | .648 | .761 | .303 | .013 | -.018 | -.275 | 1.298 | -.474 | -.250 |
|  | .542 | .597 | .637 | .634 | .601 | .706 | .752 | .758 | .647 |
|  | [-.413, 1.709] | [-.410, 1.932] | [-.945, 1.551] | [-1.229, 1.255] | [-1.196, 1.160] | [-1.658, 1.108] | [-.175, 2.771] | [-1.959, 1.012] | [-1.518, 1.017] |
|  | (.232) | (.203) | (.634) | (.984) | (.976) | (.697) | (.084) | (.532) | (.699) |
| N | 2970 | 2974 | 2967 | 2973 | 2973 | 2973 | 2974 | 2975 | 2973 |
| Pseudo-R^2^ |  |  |  |  |  |  |  |  |  |
| Model χ^2^ | 311.0 | 398.8 | 24.3 | 246.5 | 284.7 | 211.3 | 288.7 | 273.2 | 139.4 |
| d.f. | 88 | 88 | 88 | 88 | 88 | 88 | 88 | 88 | 88 |
| p-value | <.001 | <.001 | <.001 | <.001 | <.001 | <.001 | <.001 | <.001 | <.001 |

S15 Table. Regression Results, Health Attitudes, Multilevel Models, Controlling for Positive Diagnoses.

|  | (1) | (2) | (3) | (4) | (5) | (6) | (7) | (8) | (9) |
| --- | --- | --- | --- | --- | --- | --- | --- | --- | --- |
|  | freetesting | waivecosts | closeschools | canceleverything | paidleave | compensatefirms | nationalguard | stopevents | stateslead |
| main |  |  |  |  |  |  |  |  |  |
| Democrat | .554 | .735 | .459 | .395 | .589 | -.176 | .215 | .501 | -.025 |
|  | .044 | .049 | .052 | .052 | .049 | .057 | .061 | .061 | .052 |
|  | [.468, .641] | [.640, .831] | [.357, .561] | [.294, .496] | [.493, .685] | [-.289, -.063] | [.097, .334] | [.381, .621] | [-.126, .076] |
|  | (<.001) | (<.001) | (<.001) | (<.001) | (<.001) | (.002) | (<.001) | (<.001) | (.626) |
| Other | .257 | .360 | .125 | .018 | .194 | -.356 | -.175 | .117 | -.165 |
|  | .045 | .049 | .053 | .052 | .050 | .058 | .061 | .062 | .052 |
|  | [.169, .344] | [.263, .457] | [.022, .228] | [-.085, .120] | [.097, .291] | [-.470, -.242] | [-.295, -.055] | [-.005, .238] | [-.268, -.063] |
|  | (<.001) | (<.001) | (.018) | (.736) | (<.001) | (<.001) | (.004) | (.059) | (.002) |
| Local COVID-19 Cases | <.001 | <.001 | <.001 | <.001 | <.001 | <.001 | <.001 | -<.001 | <.001 |
|  | <.001 | <.001 | <.001 | <.001 | <.001 | <.001 | <.001 | <.001 | <.001 |
|  | [-<.001, .000] | [-<.001, .000] | [-<.001, .000] | [-<.001, .000] | [-<.001, .000] | [-<.001, .000] | [-<.001, .000] | [-<.001, -<.001] | [-<.001, .000] |
|  | (.137) | (.590) | (.270) | (.655) | (.387) | (.145) | (.982) | (.011) | (.169) |
| 35- | .145 | .169 | .116 | .147 | .142 | .089 | .024 | .028 | .188 |
|  | .049 | .055 | .058 | .058 | .055 | .064 | .068 | .069 | .058 |
|  | [.048, .242] | [.062, .276] | [.002, .230] | [.034, .261] | [.035, .250] | [-.037, .216] | [-.110, .157] | [-.107, .162] | [.074, .302] |
|  | (.003) | (.002) | (.046) | (.011) | (.010) | (.165) | (.726) | (.688) | (.001) |
| 50- | .154 | .119 | .105 | .143 | .074 | .033 | -.077 | .173 | .179 |
|  | .047 | .052 | .055 | .055 | .052 | .061 | .064 | .065 | .055 |
|  | [.062, .246] | [.018, .220] | [-.002, .213] | [.036, .250] | [-.027, .176] | [-.086, .152] | [-.203, .049] | [.046, .300] | [.072, .287] |
|  | (.001) | (.021) | (.055) | (.009) | (.152) | (.589) | (.228) | (.008) | (.001) |
| 70- | .204 | .012 | .127 | .332 | -.006 | -.040 | .225 | .233 | .310 |
|  | .059 | .065 | .069 | .069 | .065 | .077 | .081 | .082 | .069 |
|  | [.088, .320] | [-.116, .140] | [-.009, .263] | [.196, .467] | [-.135, .122] | [-.191, .111] | [.065, .384] | [.073, .394] | [.174, .446] |
|  | (.001) | (.856) | (.068) | (<.001) | (.925) | (.604) | (.006) | (.004) | (<.001) |
| gender | .105 | .053 | .181 | .233 | .139 | .320 | .193 | .210 | .098 |
|  | .034 | .038 | .040 | .040 | .038 | .044 | .047 | .047 | .040 |
|  | [.038, .172] | [-.021, .127] | [.102, .259] | [.155, .311] | [.065, .213] | [.233, .407] | [.101, .285] | [.117, .302] | [.019, .176] |
|  | (.002) | (.158) | (<.001) | (<.001) | (<.001) | (<.001) | (<.001) | (<.001) | (.014) |
| white | .122 | .159 | .020 | .015 | .143 | -.057 | -.182 | .250 | -.024 |
|  | .041 | .045 | .048 | .048 | .045 | .053 | .056 | .056 | .048 |
|  | [.043, .202] | [.071, .248] | [-.074, .114] | [-.078, .109] | [.054, .231] | [-.160, .047] | [-.292, -.072] | [.139, .361] | [-.117, .070] |
|  | (.003) | (<.001) | (.673) | (.751) | (.002) | (.285) | (.001) | (<.001) | (.616) |
| married | -.045 | -.023 | -.015 | .036 | -.024 | -.006 | .128 | -.061 | .003 |
|  | .037 | .041 | .044 | .044 | .041 | .049 | .051 | .052 | .044 |
|  | [-.118, .029] | [-.104, .058] | [-.101, .071] | [-.050, .122] | [-.105, .057] | [-.102, .089] | [.027, .229] | [-.163, .040] | [-.083, .088] |
|  | (.234) | (.582) | (.734) | (.410) | (.564) | (.894) | (.013) | (.237) | (.952) |
| High school graduate | -.179 | -.160 | -.118 | -.076 | -.220 | -.267 | -.128 | .108 | -.233 |
|  | .100 | .110 | .117 | .117 | .111 | .130 | .137 | .138 | .117 |
|  | [-.374, .016] | [-.377, .056] | [-.347, .112] | [-.305, .154] | [-.438, -.003] | [-.521, -.013] | [-.397, .141] | [-.163, .379] | [-.464, -.003] |
|  | (.072) | (.146) | (.315) | (.519) | (.047) | (.039) | (.351) | (.436) | (.047) |
| Some college | -.098 | -.078 | -.154 | -.076 | -.126 | -.422 | -.389 | .317 | -.243 |
|  | .102 | .113 | .119 | .119 | .113 | .132 | .140 | .141 | .120 |
|  | [-.297, .101] | [-.299, .143] | [-.388, .080] | [-.310, .158] | [-.348, .096] | [-.681, -.163] | [-.663, -.115] | [.041, .593] | [-.478, -.009] |
|  | (.335) | (.489) | (.197) | (.526) | (.267) | (.001) | (.005) | (.024) | (.042) |
| 2-year | -.070 | -.087 | -.162 | -.065 | -.139 | -.261 | -.322 | .299 | -.252 |
|  | .107 | .118 | .126 | .125 | .119 | .139 | .147 | .148 | .126 |
|  | [-.279, .139] | [-.319, .145] | [-.408, .084] | [-.311, .181] | [-.373, .094] | [-.533, .012] | [-.610, -.034] | [.009, .590] | [-.499, -.006] |
|  | (.513) | (.461) | (.198) | (.605) | (.241) | (.061) | (.029) | (.043) | (.045) |
| 4-year | -.230 | -.227 | -.225 | -.122 | -.235 | -.425 | -.384 | .299 | -.280 |
|  | .104 | .115 | .122 | .122 | .115 | .135 | .143 | .144 | .122 |
|  | [-.433, -.026] | [-.452, -.002] | [-.464, .014] | [-.361, .116] | [-.461, -.008] | [-.690, -.161] | [-.664, -.104] | [.017, .581] | [-.519, -.041] |
|  | (.027) | (.048) | (.065) | (.315) | (.042) | (.002) | (.007) | (.038) | (.022) |
| Post-grad | -.207 | -.222 | -.304 | -.091 | -.246 | -.524 | -.349 | .378 | -.417 |
|  | .109 | .121 | .128 | .128 | .121 | .142 | .150 | .151 | .128 |
|  | [-.420, .007] | [-.459, .014] | [-.555, -.054] | [-.342, .159] | [-.484, -.009] | [-.802, -.246] | [-.642, -.055] | [.082, .675] | [-.669, -.166] |
|  | (.058) | (.065) | (.017) | (.475) | (.042) | (<.001) | (.020) | (.012) | (.001) |
| income=2 | .120 | .057 | .067 | .049 | -.004 | -.084 | .064 | -.006 | .067 |
|  | .096 | .106 | .113 | .113 | .107 | .125 | .133 | .134 | .113 |
|  | [-.069, .309] | [-.151, .265] | [-.155, .289] | [-.172, .270] | [-.214, .206] | [-.329, .161] | [-.196, .324] | [-.267, .256] | [-.155, .288] |
|  | (.214) | (.589) | (.555) | (.662) | (.969) | (.502) | (.627) | (.967) | (.555) |
| income=3 | .190 | .117 | .119 | .055 | .138 | -.085 | -.028 | -.103 | .229 |
|  | .093 | .103 | .110 | .109 | .104 | .121 | .128 | .129 | .109 |
|  | [.007, .373] | [-.085, .319] | [-.097, .334] | [-.159, .268] | [-.066, .341] | [-.323, .153] | [-.279, .224] | [-.356, .151] | [.015, .443] |
|  | (.042) | (.255) | (.280) | (.615) | (.185) | (.483) | (.829) | (.426) | (.036) |
| income=4 | .218 | .192 | .120 | .145 | .096 | -.154 | .092 | .172 | .147 |
|  | .096 | .106 | .113 | .112 | .106 | .124 | .132 | .133 | .112 |
|  | [.030, .405] | [-.015, .399] | [-.100, .341] | [-.074, .363] | [-.112, .304] | [-.398, .089] | [-.166, .350] | [-.088, .432] | [-.073, .366] |
|  | (.023) | (.069) | (.285) | (.195) | (.366) | (.214) | (.484) | (.194) | (.190) |
| income=5 | .185 | .175 | -.033 | .032 | .092 | -.310 | -.154 | .012 | .092 |
|  | .098 | .108 | .115 | .114 | .108 | .127 | .134 | .135 | .114 |
|  | [-.006, .377] | [-.036, .385] | [-.258, .193] | [-.191, .255] | [-.120, .304] | [-.559, -.062] | [-.417, .109] | [-.253, .277] | [-.132, .316] |
|  | (.058) | (.105) | (.775) | (.780) | (.395) | (.014) | (.252) | (.931) | (.422) |
| income=6 | .171 | .103 | .140 | .138 | .121 | -.136 | .100 | .101 | .157 |
|  | .099 | .109 | .116 | .115 | .109 | .128 | .136 | .137 | .115 |
|  | [-.022, .365] | [-.111, .316] | [-.087, .368] | [-.088, .364] | [-.093, .336] | [-.387, .115] | [-.166, .366] | [-.167, .368] | [-.069, .383] |
|  | (.083) | (.345) | (.226) | (.231) | (.268) | (.289) | (.462) | (.462) | (.174) |
| income=7 | .241 | .024 | .240 | .215 | .120 | .023 | .036 | .251 | .028 |
|  | .106 | .116 | .125 | .123 | .118 | .137 | .146 | .147 | .124 |
|  | [.034, .448] | [-.204, .252] | [-.004, .484] | [-.026, .457] | [-.110, .351] | [-.246, .292] | [-.249, .321] | [-.036, .538] | [-.214, .271] |
|  | (.023) | (.837) | (.054) | (.081) | (.307) | (.868) | (.805) | (.087) | (.818) |
| income=8 | .197 | .090 | .088 | .052 | .208 | -.053 | -.141 | .040 | .087 |
|  | .103 | .113 | .121 | .120 | .114 | .134 | .142 | .143 | .120 |
|  | [-.005, .398] | [-.133, .312] | [-.150, .325] | [-.183, .288] | [-.016, .431] | [-.315, .209] | [-.419, .136] | [-.239, .320] | [-.149, .323] |
|  | (.056) | (.429) | (.469) | (.663) | (.069) | (.690) | (.318) | (.778) | (.469) |
| income=9 | .011 | -.137 | .071 | -.018 | .029 | -.169 | -.246 | .203 | .039 |
|  | .102 | .112 | .120 | .119 | .113 | .132 | .140 | .141 | .119 |
|  | [-.189, .211] | [-.357, .083] | [-.164, .306] | [-.251, .215] | [-.192, .250] | [-.428, .090] | [-.521, .028] | [-.073, .479] | [-.194, .273] |
|  | (.914) | (.221) | (.553) | (.880) | (.797) | (.200) | (.078) | (.149) | (.742) |
| income=10 | .185 | .039 | .022 | .153 | .123 | -.074 | -.086 | .202 | .071 |
|  | .106 | .117 | .124 | .123 | .117 | .137 | .145 | .147 | .124 |
|  | [-.022, .393] | [-.189, .268] | [-.222, .265] | [-.089, .395] | [-.107, .353] | [-.343, .195] | [-.371, .199] | [-.085, .489] | [-.172, .313] |
|  | (.080) | (.736) | (.862) | (.214) | (.293) | (.592) | (.553) | (.168) | (.568) |
| income=11 | .213 | -.053 | .186 | .225 | .010 | -.089 | -.132 | .267 | .106 |
|  | .109 | .120 | .128 | .127 | .120 | .141 | .149 | .150 | .127 |
|  | [-<.001, .426] | [-.288, .182] | [-.064, .436] | [-.024, .473] | [-.226, .246] | [-.365, .187] | [-.425, .161] | [-.027, .562] | [-.143, .355] |
|  | (.050) | (.660) | (.145) | (.076) | (.933) | (.528) | (.377) | (.075) | (.405) |
| income=12 | .072 | -.127 | .070 | .105 | .002 | -.105 | -.276 | .342 | .086 |
|  | .120 | .132 | .141 | .140 | .132 | .155 | .165 | .166 | .140 |
|  | [-.162, .306] | [-.385, .131] | [-.207, .346] | [-.169, .378] | [-.258, .261] | [-.409, .199] | [-.599, .046] | [.017, .667] | [-.188, .359] |
|  | (.547) | (.335) | (.621) | (.454) | (.989) | (.499) | (.093) | (.039) | (.541) |
| income=13 | .387 | .186 | .119 | .338 | .162 | -.392 | -.071 | .373 | .039 |
|  | .165 | .182 | .196 | .195 | .183 | .214 | .227 | .229 | .193 |
|  | [.064, .711] | [-.171, .542] | [-.265, .503] | [-.044, .720] | [-.196, .520] | [-.812, .028] | [-.515, .374] | [-.075, .821] | [-.340, .417] |
|  | (.019) | (.307) | (.545) | (.083) | (.376) | (.067) | (.756) | (.103) | (.842) |
| income=14 | -.126 | -.188 | -.269 | -.012 | -.098 | -.244 | -.513 | .288 | -.276 |
|  | .175 | .193 | .205 | .204 | .194 | .227 | .240 | .242 | .204 |
|  | [-.468, .217] | [-.566, .190] | [-.672, .133] | [-.412, .388] | [-.478, .281] | [-.689, .201] | [-.984, -.042] | [-.186, .763] | [-.677, .124] |
|  | (.471) | (.329) | (.190) | (.953) | (.613) | (.283) | (.033) | (.234) | (.177) |
| income=15 | .717 | .764 | .033 | .301 | .506 | .201 | 1.428 | .796 | 1.262 |
|  | .528 | .583 | .621 | .618 | .586 | .688 | .727 | .733 | .619 |
|  | [-.319, 1.753] | [-.379, 1.907] | [-1.184, 1.251] | [-.911, 1.513] | [-.643, 1.654] | [-1.147, 1.550] | [.004, 2.853] | [-.640, 2.233] | [.049, 2.475] |
|  | (.175) | (.190) | (.957) | (.627) | (.388) | (.770) | (.049) | (.277) | (.041) |
| income=16 | .409 | .451 | .412 | .474 | .563 | -.019 | .104 | -.035 | .151 |
|  | .248 | .274 | .292 | .291 | .275 | .323 | .342 | .344 | .291 |
|  | [-.078, .896] | [-.086, .988] | [-.161, .984] | [-.096, 1.043] | [.024, 1.103] | [-.653, .614] | [-.565, .774] | [-.711, .640] | [-.419, .721] |
|  | (.100) | (.100) | (.158) | (.103) | (.041) | (.953) | (.760) | (.918) | (.603) |
| income=17 | .073 | .002 | -.005 | .006 | -.035 | -.182 | -.334 | .181 | .103 |
|  | .091 | .100 | .107 | .106 | .100 | .117 | .125 | .126 | .106 |
|  | [-.105, .251] | [-.194, .197] | [-.214, .204] | [-.201, .213] | [-.231, .162] | [-.412, .049] | [-.578, -.090] | [-.065, .427] | [-.105, .311] |
|  | (.421) | (.985) | (.961) | (.955) | (.731) | (.122) | (.007) | (.150) | (.331) |
| Rural Code=2 | -.040 | -.053 | -.103 | -.083 | .005 | -.088 | -.124 | -.029 | -.020 |
|  | .046 | .051 | .055 | .054 | .051 | .060 | .064 | .065 | .055 |
|  | [-.131, .051] | [-.153, .048] | [-.210, .004] | [-.189, .024] | [-.096, .106] | [-.207, .030] | [-.250, .001] | [-.155, .098] | [-.127, .088] |
|  | (.394) | (.306) | (.060) | (.128) | (.922) | (.144) | (.052) | (.658) | (.720) |
| Rural Code=3 | -.079 | -.195 | -.146 | -.039 | -.019 | -.003 | -.049 | -.007 | -.047 |
|  | .063 | .069 | .074 | .073 | .069 | .082 | .086 | .087 | .074 |
|  | [-.202, .044] | [-.330, -.060] | [-.290, -.002] | [-.182, .105] | [-.155, .117] | [-.163, .157] | [-.218, .121] | [-.178, .164] | [-.192, .098] |
|  | (.206) | (.005) | (.047) | (.597) | (.785) | (.975) | (.573) | (.936) | (.525) |
| Rural Code=4 | -.031 | .046 | -.230 | -.059 | .097 | -.027 | -.149 | -.189 | .077 |
|  | .086 | .095 | .102 | .101 | .096 | .113 | .119 | .120 | .102 |
|  | [-.200, .138] | [-.141, .233] | [-.429, -.031] | [-.257, .139] | [-.091, .285] | [-.248, .193] | [-.382, .085] | [-.425, .046] | [-.123, .277] |
|  | (.719) | (.630) | (.023) | (.557) | (.312) | (.809) | (.212) | (.115) | (.452) |
| Rural Code=5 | .005 | -.100 | .091 | .096 | .133 | -.065 | .018 | .259 | -.095 |
|  | .132 | .145 | .155 | .154 | .146 | .172 | .182 | .183 | .155 |
|  | [-.253, .263] | [-.385, .185] | [-.213, .394] | [-.206, .398] | [-.153, .419] | [-.401, .271] | [-.338, .373] | [-.100, .618] | [-.399, .209] |
|  | (.969) | (.491) | (.558) | (.533) | (.362) | (.704) | (.923) | (.157) | (.540) |
| Rural Code=6 | .013 | -.032 | -.090 | -.134 | .051 | .026 | .016 | -.123 | -.209 |
|  | .086 | .095 | .101 | .100 | .095 | .112 | .118 | .119 | .101 |
|  | [-.155, .182] | [-.218, .154] | [-.288, .107] | [-.331, .063] | [-.136, .237] | [-.193, .245] | [-.216, .248] | [-.357, .111] | [-.407, -.010] |
|  | (.875) | (.733) | (.370) | (.183) | (.594) | (.818) | (.891) | (.302) | (.039) |
| Rural Code=7 | -.186 | -.095 | -.317 | -.177 | -.201 | -.141 | -.115 | -.263 | -.204 |
|  | .119 | .130 | .139 | .138 | .131 | .153 | .162 | .164 | .139 |
|  | [-.418, .046] | [-.350, .159] | [-.590, -.044] | [-.447, .093] | [-.457, .055] | [-.441, .160] | [-.433, .203] | [-.583, .058] | [-.477, .069] |
|  | (.117) | (.464) | (.023) | (.200) | (.123) | (.359) | (.480) | (.109) | (.143) |
| Rural Code=8 | -.039 | -.110 | -.224 | -.402 | -.004 | .129 | -.724 | -.083 | .455 |
|  | .220 | .243 | .259 | .258 | .250 | .287 | .311 | .305 | .258 |
|  | [-.470, .393] | [-.586, .366] | [-.731, .283] | [-.907, .102] | [-.495, .487] | [-.432, .691] | [-1.333, -.115] | [-.682, .516] | [-.051, .961] |
|  | (.860) | (.651) | (.387) | (.118) | (.987) | (.652) | (.020) | (.786) | (.078) |
| Rural Code=9 | .002 | .070 | .090 | -.052 | .062 | -.414 | .047 | .003 | -.011 |
|  | .187 | .206 | .219 | .218 | .207 | .243 | .257 | .259 | .219 |
|  | [-.364, .368] | [-.334, .473] | [-.341, .520] | [-.480, .376] | [-.343, .468] | [-.890, .063] | [-.457, .551] | [-.505, .511] | [-.441, .419] |
|  | (.990) | (.735) | (.683) | (.813) | (.763) | (.089) | (.856) | (.991) | (.960) |
| Alaska | -.001 | -.032 | .295 | .254 | .012 | .382 | .148 | .357 | -.021 |
|  | .374 | .412 | .439 | .437 | .415 | .487 | .514 | .519 | .439 |
|  | [-.733, .731] | [-.840, .776] | [-.566, 1.156] | [-.602, 1.111] | [-.801, .826] | [-.573, 1.337] | [-.860, 1.155] | [-.661, 1.375] | [-.881, .840] |
|  | (.998) | (.938) | (.502) | (.561) | (.976) | (.433) | (.774) | (.492) | (.963) |
| Arizona | -.218 | -.080 | -.017 | -.127 | -.070 | -.288 | -.322 | .096 | -.284 |
|  | .178 | .196 | .210 | .208 | .199 | .233 | .245 | .249 | .211 |
|  | [-.566, .130] | [-.464, .304] | [-.428, .394] | [-.534, .281] | [-.460, .320] | [-.745, .170] | [-.802, .158] | [-.393, .584] | [-.698, .129] |
|  | (.219) | (.684) | (.936) | (.542) | (.724) | (.218) | (.189) | (.701) | (.178) |
| Arkansas | -.178 | -.152 | -.076 | -.107 | -.135 | -.267 | -.054 | .139 | -.123 |
|  | .200 | .220 | .235 | .234 | .223 | .262 | .275 | .279 | .236 |
|  | [-.569, .213] | [-.583, .280] | [-.536, .384] | [-.565, .351] | [-.571, .302] | [-.780, .246] | [-.593, .485] | [-.408, .686] | [-.587, .340] |
|  | (.372) | (.491) | (.745) | (.647) | (.545) | (.308) | (.844) | (.619) | (.601) |
| California | -.101 | .086 | .054 | -.047 | .011 | -.278 | .128 | .320 | -.211 |
|  | .157 | .174 | .185 | .184 | .176 | .207 | .217 | .221 | .187 |
|  | [-.409, .207] | [-.254, .426] | [-.308, .417] | [-.408, .314] | [-.335, .356] | [-.684, .128] | [-.297, .552] | [-.114, .753] | [-.576, .155] |
|  | (.521) | (.621) | (.769) | (.798) | (.952) | (.179) | (.555) | (.148) | (.259) |
| Colorado | -.105 | -.341 | -.101 | -.069 | -.144 | -.233 | .053 | .306 | -.148 |
|  | .201 | .222 | .236 | .235 | .224 | .263 | .277 | .281 | .238 |
|  | [-.499, .288] | [-.777, .095] | [-.564, .361] | [-.529, .391] | [-.583, .294] | [-.749, .282] | [-.489, .595] | [-.245, .857] | [-.614, .318] |
|  | (.599) | (.126) | (.667) | (.769) | (.519) | (.375) | (.848) | (.276) | (.534) |
| Connecticut | .091 | .297 | .501 | .437 | .087 | .126 | .254 | .248 | .198 |
|  | .218 | .241 | .257 | .256 | .244 | .286 | .301 | .306 | .259 |
|  | [-.337, .519] | [-.176, .769] | [-.002, 1.005] | [-.064, .938] | [-.391, .564] | [-.434, .687] | [-.336, .845] | [-.351, .847] | [-.309, .705] |
|  | (.678) | (.218) | (.051) | (.087) | (.722) | (.659) | (.399) | (.417) | (.443) |
| Delaware | -.327 | .187 | .107 | .128 | -.346 | -.629 | .305 | -.025 | -.293 |
|  | .282 | .311 | .331 | .330 | .314 | .368 | .389 | .394 | .343 |
|  | [-.879, .225] | [-.422, .797] | [-.542, .756] | [-.519, .774] | [-.961, .268] | [-1.351, .092] | [-.458, 1.067] | [-.796, .747] | [-.965, .379] |
|  | (.246) | (.547) | (.747) | (.698) | (.269) | (.087) | (.434) | (.950) | (.393) |
| District of Columbia | -.640 | -.089 | .123 | -.216 | -.616 | .060 | .499 | .573 | .121 |
|  | .352 | .389 | .438 | .412 | .391 | .460 | .485 | .490 | .438 |
|  | [-1.330, .050] | [-.851, .672] | [-.736, .981] | [-1.023, .592] | [-1.383, .151] | [-.840, .961] | [-.451, 1.448] | [-.387, 1.533] | [-.737, .979] |
|  | (.069) | (.818) | (.779) | (.601) | (.115) | (.895) | (.303) | (.242) | (.782) |
| Florida | -.132 | -.067 | .169 | -.084 | -.101 | -.157 | .244 | .288 | -.056 |
|  | .158 | .175 | .186 | .185 | .177 | .208 | .218 | .222 | .188 |
|  | [-.442, .179] | [-.409, .275] | [-.196, .534] | [-.447, .279] | [-.449, .246] | [-.566, .251] | [-.183, .672] | [-.148, .724] | [-.424, .312] |
|  | (.406) | (.702) | (.364) | (.650) | (.568) | (.450) | (.263) | (.195) | (.766) |
| Georgia | -.199 | -.304 | .030 | -.219 | -.056 | -.337 | .196 | .029 | -.137 |
|  | .171 | .189 | .202 | .201 | .192 | .225 | .236 | .240 | .203 |
|  | [-.535, .137] | [-.675, .067] | [-.365, .425] | [-.612, .174] | [-.432, .320] | [-.779, .104] | [-.267, .659] | [-.442, .500] | [-.535, .261] |
|  | (.247) | (.108) | (.882) | (.276) | (.769) | (.134) | (.406) | (.905) | (.499) |
| Hawaii | .255 | .596 | .614 | .362 | .089 | -.135 | .420 | -.010 | .036 |
|  | .303 | .335 | .356 | .355 | .337 | .396 | .419 | .423 | .359 |
|  | [-.339, .849] | [-.060, 1.252] | [-.085, 1.312] | [-.333, 1.058] | [-.571, .750] | [-.911, .641] | [-.401, 1.240] | [-.839, .820] | [-.668, .739] |
|  | (.400) | (.075) | (.085) | (.307) | (.791) | (.733) | (.316) | (.982) | (.921) |
| Idaho | -.367 | -.409 | .199 | -.310 | -.490 | -.343 | -.505 | .357 | -.289 |
|  | .254 | .280 | .298 | .297 | .283 | .332 | .351 | .355 | .301 |
|  | [-.864, .131] | [-.958, .140] | [-.386, .784] | [-.892, .272] | [-1.044, .064] | [-.994, .307] | [-1.192, .183] | [-.339, 1.053] | [-.880, .301] |
|  | (.149) | (.144) | (.505) | (.297) | (.083) | (.301) | (.150) | (.315) | (.337) |
| Illinois | -.178 | -.122 | .089 | .025 | -.020 | -.306 | .201 | .450 | -.137 |
|  | .175 | .193 | .205 | .204 | .195 | .229 | .240 | .245 | .207 |
|  | [-.520, .165] | [-.499, .256] | [-.313, .491] | [-.376, .425] | [-.403, .362] | [-.756, .143] | [-.270, .672] | [-.029, .930] | [-.543, .268] |
|  | (.309) | (.527) | (.665) | (.904) | (.917) | (.182) | (.403) | (.066) | (.507) |
| Indiana | .009 | .256 | .483 | .220 | .011 | -.242 | .075 | .675 | -.201 |
|  | .187 | .206 | .220 | .218 | .209 | .245 | .258 | .261 | .222 |
|  | [-.357, .374] | [-.147, .660] | [.051, .914] | [-.209, .648] | [-.397, .420] | [-.723, .238] | [-.431, .580] | [.163, 1.188] | [-.635, .233] |
|  | (.963) | (.213) | (.028) | (.315) | (.956) | (.322) | (.773) | (.010) | (.365) |
| Iowa | -.196 | -.196 | .021 | .050 | -.111 | .117 | .327 | .579 | -.053 |
|  | .222 | .245 | .261 | .260 | .247 | .290 | .305 | .310 | .262 |
|  | [-.631, .239] | [-.676, .284] | [-.490, .532] | [-.459, .559] | [-.596, .374] | [-.453, .686] | [-.272, .926] | [-.028, 1.186] | [-.566, .459] |
|  | (.378) | (.423) | (.936) | (.848) | (.653) | (.688) | (.284) | (.061) | (.838) |
| Kansas | -.020 | .155 | .016 | .343 | .137 | -.200 | .151 | .568 | -.174 |
|  | .268 | .296 | .316 | .314 | .299 | .351 | .370 | .374 | .317 |
|  | [-.546, .507] | [-.425, .736] | [-.603, .634] | [-.273, .959] | [-.449, .722] | [-.888, .487] | [-.574, .876] | [-.166, 1.301] | [-.795, .447] |
|  | (.942) | (.600) | (.961) | (.275) | (.647) | (.568) | (.683) | (.130) | (.582) |
| Kentucky | -.353 | -.111 | .052 | -.290 | -.059 | .108 | -.178 | .256 | -.074 |
|  | .203 | .224 | .239 | .238 | .228 | .266 | .281 | .284 | .240 |
|  | [-.751, .045] | [-.550, .328] | [-.416, .520] | [-.756, .176] | [-.505, .387] | [-.414, .629] | [-.729, .373] | [-.301, .813] | [-.545, .397] |
|  | (.082) | (.621) | (.828) | (.222) | (.795) | (.686) | (.527) | (.367) | (.758) |
| Louisiana | -.181 | .190 | .067 | -.174 | -.092 | -.223 | .128 | .137 | -.141 |
|  | .204 | .225 | .239 | .238 | .227 | .267 | .281 | .285 | .241 |
|  | [-.580, .218] | [-.250, .631] | [-.402, .536] | [-.641, .293] | [-.538, .353] | [-.746, .300] | [-.423, .679] | [-.422, .696] | [-.614, .332] |
|  | (.375) | (.398) | (.780) | (.465) | (.685) | (.403) | (.649) | (.630) | (.559) |
| Maine | -.156 | -.032 | .741 | .370 | .097 | -.115 | -.363 | .306 | .305 |
|  | .282 | .311 | .332 | .330 | .314 | .369 | .389 | .394 | .334 |
|  | [-.709, .397] | [-.642, .578] | [.091, 1.391] | [-.277, 1.017] | [-.519, .712] | [-.837, .608] | [-1.127, .400] | [-.466, 1.078] | [-.349, .960] |
|  | (.581) | (.919) | (.026) | (.263) | (.758) | (.755) | (.351) | (.437) | (.361) |
| Maryland | -.120 | -.013 | -.035 | -.188 | .161 | -.240 | .271 | .143 | -.062 |
|  | .202 | .223 | .237 | .236 | .225 | .265 | .278 | .282 | .239 |
|  | [-.516, .275] | [-.449, .423] | [-.500, .430] | [-.651, .275] | [-.280, .602] | [-.758, .279] | [-.274, .816] | [-.410, .696] | [-.530, .405] |
|  | (.550) | (.953) | (.883) | (.426) | (.474) | (.365) | (.329) | (.613) | (.794) |
| Massachusetts | -.068 | .074 | -.047 | -.047 | .047 | -.164 | .091 | .541 | -.346 |
|  | .190 | .210 | .223 | .222 | .212 | .249 | .262 | .266 | .225 |
|  | [-.441, .304] | [-.337, .485] | [-.485, .391] | [-.482, .389] | [-.369, .463] | [-.653, .325] | [-.423, .604] | [.019, 1.063] | [-.787, .096] |
|  | (.720) | (.725) | (.833) | (.834) | (.823) | (.510) | (.730) | (.042) | (.125) |
| Michigan | -.166 | -.004 | .232 | -.185 | -.166 | -.191 | .025 | .397 | -.095 |
|  | .169 | .187 | .199 | .198 | .189 | .222 | .233 | .238 | .201 |
|  | [-.497, .166] | [-.370, .362] | [-.157, .622] | [-.573, .203] | [-.537, .205] | [-.627, .245] | [-.432, .482] | [-.069, .863] | [-.488, .298] |
|  | (.328) | (.985) | (.243) | (.349) | (.381) | (.390) | (.914) | (.095) | (.635) |
| Minnesota | -.316 | -.173 | .126 | -.011 | -.226 | -.188 | .036 | .182 | .040 |
|  | .199 | .220 | .234 | .233 | .223 | .262 | .275 | .279 | .236 |
|  | [-.707, .075] | [-.604, .259] | [-.333, .586] | [-.468, .447] | [-.662, .210] | [-.700, .325] | [-.502, .575] | [-.365, .729] | [-.422, .502] |
|  | (.113) | (.433) | (.591) | (.963) | (.310) | (.473) | (.895) | (.515) | (.865) |
| Mississippi | -.398 | -.309 | .136 | -.029 | -.093 | .065 | .269 | .077 | .066 |
|  | .226 | .249 | .265 | .264 | .252 | .295 | .311 | .315 | .266 |
|  | [-.841, .044] | [-.797, .180] | [-.384, .656] | [-.547, .489] | [-.586, .400] | [-.515, .644] | [-.340, .879] | [-.540, .695] | [-.456, .588] |
|  | (.078) | (.215) | (.609) | (.913) | (.711) | (.827) | (.386) | (.806) | (.805) |
| Missouri | -.316 | -.164 | -.052 | -.288 | -.037 | -.310 | .095 | .130 | -.088 |
|  | .184 | .202 | .215 | .214 | .204 | .241 | .252 | .257 | .217 |
|  | [-.676, .044] | [-.561, .232] | [-.473, .369] | [-.707, .131] | [-.438, .363] | [-.782, .161] | [-.399, .588] | [-.373, .634] | [-.513, .338] |
|  | (.086) | (.417) | (.809) | (.178) | (.855) | (.197) | (.707) | (.612) | (.686) |
| Montana | -.727 | -.693 | -.189 | -.565 | -.126 | -.820 | -.327 | -.376 | -.514 |
|  | .282 | .311 | .331 | .330 | .313 | .368 | .389 | .393 | .333 |
|  | [-1.279, -.175] | [-1.302, -.084] | [-.838, .460] | [-1.211, .081] | [-.740, .488] | [-1.541, -.098] | [-1.089, .434] | [-1.147, .394] | [-1.167, .139] |
|  | (.010) | (.026) | (.568) | (.086) | (.687) | (.026) | (.400) | (.338) | (.123) |
| Nebraska | -.094 | .236 | -.397 | -.176 | .006 | .030 | .439 | -.064 | -.102 |
|  | .299 | .320 | .341 | .340 | .323 | .390 | .399 | .404 | .342 |
|  | [-.680, .492] | [-.392, .864] | [-1.066, .272] | [-.842, .490] | [-.627, .639] | [-.736, .795] | [-.344, 1.222] | [-.856, .729] | [-.772, .567] |
|  | (.752) | (.461) | (.245) | (.604) | (.984) | (.940) | (.272) | (.875) | (.765) |
| Nevada | -.043 | .062 | .185 | .001 | -.146 | -.376 | -.178 | .404 | -.003 |
|  | .199 | .219 | .234 | .233 | .223 | .261 | .274 | .279 | .236 |
|  | [-.433, .346] | [-.368, .492] | [-.273, .643] | [-.455, .457] | [-.583, .291] | [-.887, .135] | [-.716, .360] | [-.142, .950] | [-.466, .459] |
|  | (.828) | (.778) | (.428) | (.997) | (.512) | (.149) | (.516) | (.147) | (.989) |
| New Hampshire | .003 | .208 | -.035 | -.155 | .026 | -.274 | .344 | .039 | .033 |
|  | .239 | .263 | .280 | .279 | .266 | .312 | .329 | .334 | .283 |
|  | [-.465, .470] | [-.308, .723] | [-.585, .514] | [-.702, .392] | [-.494, .547] | [-.886, .338] | [-.301, .989] | [-.614, .693] | [-.521, .587] |
|  | (.990) | (.430) | (.900) | (.579) | (.921) | (.380) | (.296) | (.906) | (.908) |
| New Jersey | -.114 | .186 | .182 | -.044 | .018 | .008 | .390 | .234 | -.046 |
|  | .185 | .204 | .218 | .216 | .206 | .242 | .255 | .259 | .218 |
|  | [-.476, .249] | [-.214, .586] | [-.245, .610] | [-.469, .380] | [-.386, .421] | [-.467, .483] | [-.109, .889] | [-.273, .742] | [-.473, .381] |
|  | (.539) | (.362) | (.403) | (.838) | (.932) | (.973) | (.125) | (.365) | (.832) |
| New Mexico | .139 | -.149 | .263 | -.136 | -.145 | -.348 | -.407 | .304 | -.357 |
|  | .220 | .242 | .258 | .257 | .245 | .288 | .303 | .307 | .260 |
|  | [-.292, .569] | [-.625, .326] | [-.244, .769] | [-.640, .368] | [-.625, .336] | [-.912, .216] | [-1.001, .187] | [-.298, .907] | [-.867, .153] |
|  | (.528) | (.538) | (.309) | (.598) | (.555) | (.227) | (.180) | (.322) | (.170) |
| New York | -.294 | -.023 | .094 | -.109 | -.105 | -.202 | .209 | .048 | -.066 |
|  | .162 | .178 | .190 | .189 | .181 | .213 | .223 | .227 | .192 |
|  | [-.611, .023] | [-.373, .326] | [-.279, .467] | [-.480, .262] | [-.460, .250] | [-.619, .215] | [-.227, .646] | [-.397, .493] | [-.442, .310] |
|  | (.069) | (.895) | (.621) | (.565) | (.561) | (.343) | (.348) | (.833) | (.731) |
| North Carolina | -.125 | .086 | .062 | .093 | -.053 | -.379 | .256 | .069 | -.262 |
|  | .174 | .192 | .205 | .204 | .195 | .230 | .240 | .244 | .207 |
|  | [-.466, .217] | [-.291, .462] | [-.340, .463] | [-.307, .493] | [-.435, .329] | [-.829, .071] | [-.214, .726] | [-.410, .547] | [-.667, .143] |
|  | (.474) | (.655) | (.764) | (.649) | (.785) | (.099) | (.286) | (.779) | (.205) |
| North Dakota | -.193 | .426 | -.369 | -.259 | .195 | -.176 | .544 | -.085 | -.491 |
|  | .353 | .390 | .415 | .414 | .393 | .461 | .488 | .493 | .418 |
|  | [-.886, .500] | [-.338, 1.190] | [-1.184, .445] | [-1.070, .551] | [-.575, .965] | [-1.080, .727] | [-.412, 1.500] | [-1.050, .881] | [-1.310, .328] |
|  | (.585) | (.274) | (.374) | (.530) | (.620) | (.702) | (.264) | (.864) | (.240) |
| Ohio | -.196 | -.022 | .162 | -.013 | -.028 | -.078 | -.025 | .336 | .075 |
|  | .169 | .187 | .199 | .198 | .189 | .223 | .233 | .237 | .201 |
|  | [-.528, .136] | [-.388, .344] | [-.228, .552] | [-.402, .375] | [-.399, .343] | [-.514, .358] | [-.482, .432] | [-.129, .802] | [-.319, .468] |
|  | (.247) | (.907) | (.416) | (.946) | (.881) | (.725) | (.913) | (.157) | (.710) |
| Oklahoma | -.268 | -.001 | .203 | .174 | -.148 | .238 | .415 | .015 | -.227 |
|  | .227 | .250 | .267 | .266 | .253 | .297 | .313 | .317 | .268 |
|  | [-.713, .177] | [-.492, .490] | [-.320, .726] | [-.347, .694] | [-.644, .348] | [-.344, .820] | [-.198, 1.027] | [-.607, .636] | [-.752, .298] |
|  | (.238) | (.996) | (.448) | (.513) | (.558) | (.423) | (.185) | (.963) | (.397) |
| Oregon | .042 | .134 | -.276 | -.136 | -.028 | -.333 | -.073 | .238 | -.386 |
|  | .188 | .207 | .220 | .219 | .209 | .246 | .258 | .263 | .222 |
|  | [-.327, .410] | [-.271, .539] | [-.708, .155] | [-.566, .293] | [-.438, .382] | [-.814, .149] | [-.580, .433] | [-.277, .752] | [-.822, .050] |
|  | (.825) | (.515) | (.209) | (.534) | (.892) | (.176) | (.777) | (.366) | (.083) |
| Pennsylvania | -.131 | -.041 | .083 | -.035 | -.005 | -.188 | .007 | .340 | -.025 |
|  | .161 | .177 | .189 | .188 | .180 | .211 | .221 | .225 | .190 |
|  | [-.445, .184] | [-.388, .306] | [-.287, .453] | [-.403, .333] | [-.358, .347] | [-.602, .226] | [-.426, .441] | [-.102, .781] | [-.398, .348] |
|  | (.416) | (.818) | (.660) | (.853) | (.977) | (.374) | (.973) | (.132) | (.895) |
| Rhode Island | .329 | .301 | .305 | -.042 | .270 | .831 | .037 | -.110 | -.033 |
|  | .352 | .388 | .413 | .411 | .391 | .459 | .484 | .489 | .413 |
|  | [-.360, 1.018] | [-.459, 1.061] | [-.505, 1.115] | [-.848, .764] | [-.495, 1.036] | [-.068, 1.730] | [-.911, .985] | [-1.069, .848] | [-.843, .777] |
|  | (.349) | (.438) | (.460) | (.919) | (.489) | (.070) | (.939) | (.822) | (.936) |
| South Carolina | -.128 | -.120 | .269 | .164 | -.320 | -.120 | .021 | .389 | -.028 |
|  | .196 | .217 | .231 | .230 | .219 | .257 | .271 | .275 | .233 |
|  | [-.513, .257] | [-.545, .304] | [-.183, .722] | [-.287, .614] | [-.749, .110] | [-.624, .385] | [-.510, .552] | [-.150, .929] | [-.485, .428] |
|  | (.514) | (.579) | (.243) | (.476) | (.145) | (.642) | (.939) | (.157) | (.904) |
| South Dakota | -.280 | -.145 | -.512 | -.242 | -.234 | -.223 | .237 | -.373 | -.062 |
|  | .325 | .359 | .382 | .380 | .361 | .424 | .448 | .453 | .384 |
|  | [-.918, .357] | [-.849, .558] | [-1.261, .237] | [-.988, .504] | [-.942, .474] | [-1.055, .609] | [-.642, 1.115] | [-1.261, .515] | [-.814, .690] |
|  | (.388) | (.685) | (.181) | (.525) | (.517) | (.599) | (.598) | (.411) | (.871) |
| Tennessee | .036 | .182 | .026 | -.285 | -.087 | -.207 | -.106 | .317 | -.233 |
|  | .192 | .212 | .226 | .225 | .214 | .252 | .265 | .269 | .227 |
|  | [-.340, .412] | [-.233, .597] | [-.417, .468] | [-.726, .155] | [-.507, .333] | [-.700, .287] | [-.625, .413] | [-.209, .844] | [-.679, .213] |
|  | (.852) | (.391) | (.910) | (.204) | (.685) | (.412) | (.688) | (.238) | (.305) |
| Texas | -.174 | -.095 | -.013 | -.108 | -.239 | -.279 | -.007 | .087 | -.090 |
|  | .160 | .177 | .189 | .188 | .180 | .211 | .221 | .225 | .190 |
|  | [-.488, .141] | [-.442, .252] | [-.383, .357] | [-.476, .260] | [-.591, .114] | [-.693, .135] | [-.440, .426] | [-.355, .529] | [-.463, .282] |
|  | (.279) | (.593) | (.946) | (.567) | (.184) | (.186) | (.975) | (.699) | (.635) |
| Utah | -.209 | -.020 | -.069 | .026 | -.063 | -.455 | .222 | .527 | -.303 |
|  | .222 | .245 | .261 | .260 | .248 | .291 | .307 | .311 | .264 |
|  | [-.645, .227] | [-.501, .460] | [-.582, .443] | [-.484, .535] | [-.549, .423] | [-1.026, .115] | [-.379, .824] | [-.083, 1.137] | [-.820, .214] |
|  | (.347) | (.933) | (.791) | (.922) | (.799) | (.118) | (.469) | (.090) | (.251) |
| Vermont | .014 | -.071 | .316 | .310 | -.382 | -.292 | .264 | .676 | -.049 |
|  | .322 | .355 | .378 | .376 | .358 | .420 | .443 | .448 | .378 |
|  | [-.617, .645] | [-.767, .624] | [-.426, 1.057] | [-.428, 1.047] | [-1.083, .320] | [-1.115, .531] | [-.604, 1.132] | [-.201, 1.554] | [-.790, .693] |
|  | (.965) | (.841) | (.404) | (.411) | (.286) | (.487) | (.551) | (.131) | (.897) |
| Virginia | -.092 | -.040 | .006 | -.126 | -.063 | -.366 | .085 | .293 | .022 |
|  | .172 | .190 | .202 | .202 | .193 | .226 | .237 | .241 | .204 |
|  | [-.429, .245] | [-.412, .332] | [-.391, .402] | [-.521, .269] | [-.441, .314] | [-.810, .077] | [-.380, .550] | [-.181, .766] | [-.379, .422] |
|  | (.592) | (.832) | (.978) | (.532) | (.742) | (.106) | (.720) | (.225) | (.916) |
| Washington | -.108 | -.012 | -.105 | -.150 | .028 | -.332 | -.210 | .306 | -.278 |
|  | .181 | .199 | .212 | .211 | .202 | .237 | .249 | .254 | .214 |
|  | [-.463, .246] | [-.402, .378] | [-.520, .311] | [-.564, .263] | [-.368, .423] | [-.797, .132] | [-.697, .277] | [-.191, .803] | [-.697, .141] |
|  | (.549) | (.951) | (.621) | (.477) | (.891) | (.161) | (.398) | (.228) | (.194) |
| West Virginia | -.645 | -.078 | .287 | -.038 | -.197 | -.322 | .144 | .402 | .020 |
|  | .230 | .253 | .273 | .269 | .256 | .300 | .316 | .320 | .274 |
|  | [-1.095, -.195] | [-.574, .419] | [-.249, .822] | [-.565, .489] | [-.699, .304] | [-.911, .267] | [-.476, .763] | [-.226, 1.031] | [-.518, .558] |
|  | (.005) | (.759) | (.294) | (.888) | (.441) | (.284) | (.649) | (.209) | (.942) |
| Wisconsin | -.339 | -.081 | .075 | -.096 | -.324 | -.361 | .041 | .316 | -.123 |
|  | .186 | .206 | .219 | .218 | .208 | .245 | .257 | .261 | .221 |
|  | [-.704, .027] | [-.486, .323] | [-.355, .504] | [-.523, .332] | [-.732, .085] | [-.841, .119] | [-.462, .545] | [-.196, .827] | [-.556, .310] |
|  | (.069) | (.693) | (.734) | (.662) | (.120) | (.140) | (.872) | (.227) | (.577) |
| Wyoming | .646 | .762 | .301 | .013 | -.019 | -.278 | 1.299 | -.468 | -.252 |
|  | .542 | .598 | .637 | .634 | .601 | .706 | .751 | .758 | .647 |
|  | [-.415, 1.708] | [-.410, 1.933] | [-.946, 1.549] | [-1.229, 1.256] | [-1.197, 1.159] | [-1.661, 1.105] | [-.174, 2.772] | [-1.954, 1.017] | [-1.520, 1.016] |
|  | (.233) | (.202) | (.636) | (.983) | (.975) | (.694) | (.084) | (.536) | (.697) |
| N | 2970 | 2974 | 2967 | 2973 | 2973 | 2973 | 2974 | 2975 | 2973 |
| Pseudo-R^2^ |  |  |  |  |  |  |  |  |  |
| Model χ^2^ | 31.7 | 398.1 | 24.2 | 245.6 | 284.4 | 211.7 | 288.5 | 274.4 | 138.3 |
| d.f. | 88 | 88 | 88 | 88 | 88 | 88 | 88 | 88 | 88 |
| p-value | <.001 | <.001 | <.001 | <.001 | <.001 | <.001 | <.001 | <.001 | <.001 |

**S7 Fig. Health Policy, Multilevel Models.** This figure compares key coefficients of interest from S13-S15 Tables.

S16 Table. Regression Results, Public Policies, Multilevel Models.

|  | (1) | (2) | (3) | (4) | (5) | (6) | (7) | (8) | (9) | (10) |
| --- | --- | --- | --- | --- | --- | --- | --- | --- | --- | --- |
|  | increasetrade | importtaxes | lowinterest | notreliant | noairtravel | banentry | entrymexico | quarantine | rescueUS | delayelections |
| main |  |  |  |  |  |  |  |  |  |  |
| Democrat | .630 | -.726 | -.253 | -.706 | -.360 | -.656 | -.992 | -.509 | .103 | -.326 |
|  | .050 | .054 | .050 | .051 | .057 | .055 | .054 | .063 | .056 | .068 |
|  | [.532, .728] | [-.831, -.621] | [-.351, -.155] | [-.806, -.606] | [-.471, -.249] | [-.765, -.548] | [-1.099, -.886] | [-.632, -.387] | [-.007, .214] | [-.460, -.193] |
|  | (<.001) | (<.001) | (<.001) | (<.001) | (<.001) | (<.001) | (<.001) | (<.001) | (.066) | (<.001) |
| Other | .114 | -.428 | -.289 | -.339 | -.252 | -.474 | -.532 | -.452 | -.072 | -.205 |
|  | .050 | .054 | .051 | .052 | .057 | .056 | .055 | .063 | .057 | .069 |
|  | [.015, .213] | [-.535, -.322] | [-.389, -.190] | [-.440, -.238] | [-.364, -.139] | [-.584, -.364] | [-.640, -.425] | [-.576, -.328] | [-.183, .040] | [-.340, -.070] |
|  | (.024) | (<.001) | (<.001) | (<.001) | (<.001) | (<.001) | (<.001) | (<.001) | (.209) | (.003) |
| 35- | -.089 | .171 | .169 | .304 | .207 | .146 | .206 | .129 | .040 | -.118 |
|  | .056 | .060 | .056 | .057 | .064 | .062 | .061 | .070 | .063 | .076 |
|  | [-.198, .021] | [.053, .289] | [.060, .279] | [.192, .416] | [.082, .332] | [.024, .267] | [.086, .325] | [-.008, .267] | [-.084, .164] | [-.268, .032] |
|  | (.113) | (.005) | (.002) | (<.001) | (.001) | (.019) | (.001) | (.065) | (.527) | (.122) |
| 50- | -.010 | .127 | .207 | .450 | .177 | .285 | .398 | -.014 | .119 | -.385 |
|  | .053 | .057 | .053 | .054 | .060 | .059 | .058 | .066 | .060 | .072 |
|  | [-.113, .094] | [.016, .239] | [.104, .311] | [.345, .556] | [.059, .295] | [.170, .400] | [.285, .511] | [-.144, .116] | [.002, .236] | [-.527, -.244] |
|  | (.855) | (.025) | (<.001) | (<.001) | (.003) | (<.001) | (<.001) | (.832) | (.047) | (<.001) |
| 70- | .056 | .029 | .227 | .429 | .116 | .218 | .421 | -.199 | .278 | -.268 |
|  | .067 | .072 | .067 | .068 | .076 | .074 | .073 | .084 | .076 | .091 |
|  | [-.075, .187] | [-.112, .170] | [.096, .359] | [.295, .563] | [-.032, .265] | [.072, .363] | [.278, .564] | [-.363, -.034] | [.130, .427] | [-.447, -.090] |
|  | (.401) | (.688) | (.001) | (<.001) | (.125) | (.003) | (<.001) | (.018) | (<.001) | (.003) |
| gender | -.136 | .161 | .098 | -.064 | .257 | .115 | .011 | .107 | -.062 | .432 |
|  | .038 | .041 | .038 | .039 | .044 | .043 | .042 | .048 | .043 | .053 |
|  | [-.212, -.061] | [.080, .243] | [.022, .173] | [-.141, .013] | [.172, .343] | [.031, .198] | [-.071, .094] | [.012, .201] | [-.147, .023] | [.329, .535] |
|  | (<.001) | (<.001) | (.011) | (.103) | (<.001) | (.007) | (.786) | (.027) | (.150) | (<.001) |
| white | -.024 | -.099 | -.076 | -.101 | -.117 | -.034 | -.089 | -.285 | .048 | -.105 |
|  | .046 | .050 | .046 | .047 | .052 | .051 | .050 | .058 | .052 | .063 |
|  | [-.114, .066] | [-.196, -.002] | [-.166, .014] | [-.193, -.009] | [-.219, -.014] | [-.134, .066] | [-.187, .009] | [-.398, -.171] | [-.054, .150] | [-.228, .018] |
|  | (.600) | (.045) | (.099) | (.031) | (.025) | (.506) | (.076) | (<.001) | (.353) | (.095) |
| married | -.035 | .125 | -.016 | <.001 | .073 | .017 | .006 | .068 | -.063 | -.060 |
|  | .042 | .045 | .042 | .043 | .048 | .047 | .046 | .053 | .048 | .058 |
|  | [-.118, .048] | [.035, .214] | [-.099, .067] | [-.084, .085] | [-.022, .167] | [-.075, .109] | [-.084, .096] | [-.036, .172] | [-.157, .030] | [-.173, .053] |
|  | (.411) | (.006) | (.708) | (.998) | (.131) | (.713) | (.900) | (.200) | (.183) | (.299) |
| High school graduate | -.332 | -.108 | -.042 | -.105 | .013 | -.317 | -.180 | -.455 | .058 | .005 |
|  | .113 | .121 | .113 | .115 | .128 | .126 | .123 | .141 | .128 | .155 |
|  | [-.554, -.110] | [-.346, .130] | [-.264, .180] | [-.330, .120] | [-.237, .264] | [-.563, -.071] | [-.420, .061] | [-.732, -.178] | [-.193, .309] | [-.300, .309] |
|  | (.003) | (.373) | (.711) | (.362) | (.917) | (.012) | (.142) | (.001) | (.651) | (.976) |
| Some college | -.303 | -.273 | -.088 | -.119 | -.228 | -.490 | -.337 | -.902 | .115 | -.206 |
|  | .115 | .124 | .115 | .117 | .131 | .128 | .125 | .144 | .131 | .158 |
|  | [-.529, -.077] | [-.515, -.031] | [-.313, .138] | [-.349, .110] | [-.483, .028] | [-.741, -.239] | [-.582, -.091] | [-1.185, -.620] | [-.141, .371] | [-.516, .105] |
|  | (.009) | (.027) | (.448) | (.309) | (.081) | (<.001) | (.007) | (<.001) | (.377) | (.194) |
| 2-year | -.260 | -.124 | -.062 | -.059 | -.127 | -.331 | -.246 | -.843 | .015 | -.094 |
|  | .121 | .130 | .121 | .123 | .137 | .135 | .132 | .151 | .137 | .166 |
|  | [-.497, -.023] | [-.378, .131] | [-.299, .175] | [-.300, .182] | [-.396, .142] | [-.595, -.067] | [-.503, .012] | [-1.140, -.546] | [-.254, .284] | [-.420, .232] |
|  | (.032) | (.341) | (.607) | (.633) | (.355) | (.014) | (.062) | (<.001) | (.913) | (.572) |
| 4-year | -.112 | -.414 | -.196 | -.303 | -.394 | -.684 | -.448 | -.967 | .057 | -.211 |
|  | .118 | .126 | .118 | .120 | .133 | .131 | .128 | .147 | .133 | .161 |
|  | [-.342, .118] | [-.661, -.166] | [-.427, .035] | [-.537, -.068] | [-.655, -.133] | [-.941, -.428] | [-.699, -.198] | [-1.255, -.678] | [-.204, .318] | [-.527, .106] |
|  | (.341) | (.001) | (.096) | (.011) | (.003) | (<.001) | (<.001) | (<.001) | (.669) | (.192) |
| Post-grad | -.050 | -.502 | -.221 | -.379 | -.588 | -.670 | -.582 | -1.139 | .011 | -.420 |
|  | .123 | .132 | .123 | .126 | .140 | .137 | .134 | .154 | .140 | .170 |
|  | [-.292, .192] | [-.761, -.242] | [-.463, .021] | [-.625, -.133] | [-.862, -.314] | [-.939, -.401] | [-.845, -.320] | [-1.442, -.837] | [-.263, .285] | [-.752, -.088] |
|  | (.685) | (<.001) | (.074) | (.003) | (<.001) | (<.001) | (<.001) | (<.001) | (.938) | (.013) |
| income=2 | -.189 | .137 | .141 | .004 | .150 | .158 | .174 | -.013 | .025 | .163 |
|  | .109 | .117 | .109 | .111 | .123 | .121 | .119 | .136 | .123 | .149 |
|  | [-.402, .024] | [-.093, .367] | [-.073, .355] | [-.213, .222] | [-.092, .392] | [-.080, .395] | [-.059, .407] | [-.281, .254] | [-.216, .266] | [-.129, .455] |
|  | (.083) | (.242) | (.197) | (.968) | (.225) | (.193) | (.143) | (.923) | (.839) | (.273) |
| income=3 | -.082 | .100 | .139 | .125 | .087 | .153 | .235 | -.233 | .143 | .209 |
|  | .105 | .113 | .106 | .108 | .120 | .117 | .115 | .132 | .119 | .144 |
|  | [-.288, .124] | [-.123, .322] | [-.069, .346] | [-.086, .336] | [-.148, .321] | [-.077, .383] | [.010, .461] | [-.492, .025] | [-.091, .376] | [-.075, .492] |
|  | (.433) | (.380) | (.190) | (.244) | (.468) | (.192) | (.041) | (.077) | (.231) | (.149) |
| income=4 | -.072 | .069 | .223 | .067 | .055 | -.019 | .130 | -.262 | .012 | .118 |
|  | .108 | .116 | .108 | .110 | .123 | .120 | .118 | .136 | .122 | .148 |
|  | [-.283, .139] | [-.159, .297] | [.011, .435] | [-.149, .283] | [-.185, .295] | [-.254, .217] | [-.101, .361] | [-.528, .004] | [-.227, .251] | [-.172, .407] |
|  | (.506) | (.554) | (.039) | (.541) | (.655) | (.875) | (.270) | (.053) | (.920) | (.426) |
| income=5 | -.075 | .009 | .105 | .188 | .046 | .089 | .238 | -.430 | .131 | .025 |
|  | .110 | .119 | .111 | .113 | .125 | .123 | .120 | .138 | .124 | .151 |
|  | [-.291, .141] | [-.224, .242] | [-.112, .322] | [-.033, .409] | [-.199, .292] | [-.152, .329] | [.002, .473] | [-.701, -.158] | [-.113, .375] | [-.271, .320] |
|  | (.495) | (.941) | (.343) | (.095) | (.712) | (.470) | (.048) | (.002) | (.293) | (.869) |
| income=6 | -.088 | -.021 | .132 | .252 | .106 | .142 | .256 | -.305 | .087 | .190 |
|  | .111 | .120 | .112 | .114 | .126 | .124 | .122 | .140 | .126 | .153 |
|  | [-.306, .130] | [-.256, .214] | [-.087, .351] | [.029, .474] | [-.142, .354] | [-.101, .385] | [.018, .495] | [-.578, -.031] | [-.159, .333] | [-.109, .489] |
|  | (.429) | (.861) | (.237) | (.027) | (.400) | (.251) | (.035) | (.029) | (.488) | (.213) |
| income=7 | -.142 | -.027 | .151 | .134 | .227 | .275 | .142 | -.309 | -.077 | .054 |
|  | .119 | .128 | .120 | .122 | .135 | .133 | .130 | .150 | .134 | .163 |
|  | [-.375, .092] | [-.279, .225] | [-.084, .385] | [-.105, .373] | [-.039, .492] | [.014, .535] | [-.113, .398] | [-.603, -.016] | [-.341, .186] | [-.265, .374] |
|  | (.235) | (.832) | (.208) | (.270) | (.094) | (.039) | (.275) | (.039) | (.566) | (.740) |
| income=8 | -.197 | -.009 | .198 | .140 | .016 | .042 | .196 | -.363 | .004 | .016 |
|  | .116 | .125 | .117 | .118 | .132 | .129 | .127 | .146 | .131 | .159 |
|  | [-.425, .030] | [-.254, .236] | [-.031, .426] | [-.092, .372] | [-.243, .274] | [-.211, .296] | [-.053, .444] | [-.648, -.077] | [-.253, .260] | [-.295, .328] |
|  | (.089) | (.945) | (.090) | (.237) | (.904) | (.744) | (.123) | (.013) | (.977) | (.918) |
| income=9 | .013 | -.164 | .164 | .064 | -.186 | .094 | .063 | -.480 | .011 | -.176 |
|  | .115 | .124 | .115 | .117 | .130 | .128 | .125 | .144 | .130 | .157 |
|  | [-.212, .238] | [-.407, .078] | [-.063, .390] | [-.166, .294] | [-.441, .070] | [-.156, .345] | [-.183, .309] | [-.763, -.198] | [-.243, .265] | [-.484, .132] |
|  | (.910) | (.184) | (.156) | (.587) | (.154) | (.461) | (.614) | (.001) | (.934) | (.264) |
| income=10 | .006 | -.174 | .164 | .013 | -.029 | -.024 | .148 | -.512 | .028 | -.189 |
|  | .119 | .129 | .120 | .122 | .136 | .133 | .130 | .150 | .135 | .163 |
|  | [-.227, .240] | [-.426, .078] | [-.071, .399] | [-.226, .252] | [-.295, .237] | [-.284, .236] | [-.107, .404] | [-.806, -.219] | [-.236, .292] | [-.509, .131] |
|  | (.958) | (.176) | (.172) | (.914) | (.831) | (.856) | (.255) | (.001) | (.834) | (.248) |
| income=11 | -.025 | -.212 | .176 | -.088 | -.176 | -.003 | .053 | -.634 | -.029 | .041 |
|  | .122 | .132 | .123 | .125 | .139 | .137 | .134 | .154 | .138 | .168 |
|  | [-.265, .215] | [-.471, .047] | [-.065, .417] | [-.333, .158] | [-.449, .097] | [-.271, .265] | [-.210, .315] | [-.935, -.332] | [-.301, .242] | [-.288, .370] |
|  | (.839) | (.109) | (.152) | (.484) | (.207) | (.981) | (.694) | (<.001) | (.832) | (.807) |
| income=12 | -.065 | -.147 | .125 | -.098 | -.107 | .026 | .220 | -.659 | .003 | -.191 |
|  | .135 | .146 | .136 | .138 | .153 | .150 | .147 | .170 | .152 | .185 |
|  | [-.330, .201] | [-.433, .139] | [-.141, .390] | [-.367, .172] | [-.407, .193] | [-.268, .320] | [-.069, .508] | [-.992, -.326] | [-.296, .301] | [-.553, .171] |
|  | (.633) | (.315) | (.357) | (.479) | (.486) | (.862) | (.135) | (<.001) | (.986) | (.300) |
| income=13 | .109 | -.369 | -.157 | -.188 | -.277 | .075 | .041 | -.809 | -.173 | -.258 |
|  | .188 | .203 | .189 | .190 | .214 | .207 | .203 | .234 | .213 | .255 |
|  | [-.261, .478] | [-.767, .029] | [-.528, .213] | [-.561, .184] | [-.697, .143] | [-.331, .481] | [-.357, .439] | [-1.267, -.351] | [-.590, .244] | [-.758, .241] |
|  | (.565) | (.069) | (.405) | (.322) | (.196) | (.719) | (.841) | (.001) | (.416) | (.311) |
| income=14 | -.120 | -.144 | .142 | .048 | -.120 | .155 | .216 | -.694 | -.318 | .040 |
|  | .197 | .212 | .197 | .201 | .224 | .219 | .215 | .247 | .222 | .270 |
|  | [-.506, .266] | [-.560, .272] | [-.245, .529] | [-.346, .443] | [-.559, .320] | [-.275, .585] | [-.205, .638] | [-1.179, -.209] | [-.754, .118] | [-.488, .569] |
|  | (.543) | (.498) | (.473) | (.810) | (.594) | (.480) | (.315) | (.005) | (.153) | (.881) |
| income=15 | .234 | -.185 | -.374 | .442 | .721 | .720 | -.383 | -.189 | 1.017 | .513 |
|  | .596 | .641 | .596 | .608 | .679 | .664 | .651 | .748 | .673 | .816 |
|  | [-.935, 1.402] | [-1.442, 1.072] | [-1.542, .795] | [-.749, 1.633] | [-.610, 2.052] | [-.581, 2.021] | [-1.658, .892] | [-1.655, 1.278] | [-.301, 2.335] | [-1.087, 2.113] |
|  | (.695) | (.773) | (.531) | (.467) | (.288) | (.278) | (.556) | (.801) | (.131) | (.530) |
| income=16 | -.182 | .038 | .381 | -.047 | .071 | .461 | .578 | -.661 | -.158 | .067 |
|  | .280 | .302 | .281 | .286 | .319 | .312 | .306 | .352 | .316 | .384 |
|  | [-.731, .368] | [-.553, .630] | [-.169, .931] | [-.607, .514] | [-.555, .696] | [-.151, 1.072] | [-.021, 1.178] | [-1.351, .029] | [-.778, .462] | [-.685, .819] |
|  | (.517) | (.899) | (.175) | (.870) | (.824) | (.140) | (.059) | (.060) | (.617) | (.861) |
| income=17 | -.243 | .021 | -.012 | .107 | -.063 | .108 | .193 | -.340 | -.061 | -.043 |
|  | .102 | .110 | .103 | .104 | .116 | .114 | .112 | .128 | .116 | .140 |
|  | [-.444, -.043] | [-.195, .237] | [-.213, .190] | [-.098, .311] | [-.290, .165] | [-.115, .331] | [-.026, .412] | [-.592, -.089] | [-.288, .165] | [-.317, .231] |
|  | (.017) | (.849) | (.908) | (.308) | (.588) | (.343) | (.084) | (.008) | (.596) | (.759) |
| Rural Code=2 | -.112 | -.041 | -.060 | .071 | -.045 | -.035 | -.077 | -.024 | -.111 | -.155 |
|  | .051 | .056 | .052 | .053 | .058 | .057 | .056 | .064 | .058 | .070 |
|  | [-.213, -.012] | [-.150, .068] | [-.161, .041] | [-.033, .174] | [-.159, .068] | [-.146, .076] | [-.186, .033] | [-.150, .103] | [-.225, .003] | [-.292, -.019] |
|  | (.029) | (.463) | (.244) | (.180) | (.432) | (.537) | (.169) | (.713) | (.056) | (.026) |
| Rural Code=3 | -.088 | -.040 | -.012 | .048 | .015 | .086 | .010 | .057 | -.131 | .006 |
|  | .070 | .075 | .070 | .072 | .079 | .077 | .076 | .088 | .079 | .095 |
|  | [-.225, .049] | [-.188, .108] | [-.149, .125] | [-.093, .188] | [-.139, .169] | [-.065, .236] | [-.139, .159] | [-.115, .228] | [-.286, .024] | [-.180, .191] |
|  | (.209) | (.593) | (.865) | (.505) | (.847) | (.266) | (.894) | (.516) | (.098) | (.951) |
| Rural Code=4 | -.214 | .050 | .075 | .126 | .060 | .050 | .259 | .153 | .062 | -.195 |
|  | .097 | .105 | .097 | .099 | .110 | .107 | .105 | .122 | .109 | .132 |
|  | [-.403, -.024] | [-.155, .256] | [-.116, .265] | [-.068, .321] | [-.155, .276] | [-.160, .260] | [.053, .466] | [-.085, .391] | [-.152, .276] | [-.454, .064] |
|  | (.027) | (.629) | (.443) | (.202) | (.582) | (.640) | (.014) | (.209) | (.571) | (.139) |
| Rural Code=5 | -.081 | -.063 | -.151 | -.113 | .178 | -.015 | .019 | -.044 | -.336 | .138 |
|  | .149 | .160 | .149 | .155 | .168 | .164 | .162 | .186 | .169 | .202 |
|  | [-.374, .211] | [-.377, .251] | [-.443, .141] | [-.417, .190] | [-.152, .508] | [-.337, .307] | [-.298, .336] | [-.409, .321] | [-.667, -.004] | [-.258, .535] |
|  | (.586) | (.694) | (.310) | (.463) | (.290) | (.927) | (.907) | (.814) | (.047) | (.494) |
| Rural Code=6 | -.128 | .111 | .074 | .097 | .117 | .087 | .087 | .208 | -.067 | -.017 |
|  | .097 | .104 | .097 | .099 | .109 | .107 | .105 | .121 | .109 | .131 |
|  | [-.317, .062] | [-.094, .315] | [-.115, .264] | [-.096, .291] | [-.097, .331] | [-.122, .296] | [-.119, .292] | [-.030, .445] | [-.280, .147] | [-.274, .240] |
|  | (.187) | (.288) | (.442) | (.325) | (.285) | (.415) | (.408) | (.086) | (.541) | (.898) |
| Rural Code=7 | -.065 | -.014 | -.224 | .075 | .006 | .253 | -.021 | .012 | .064 | -.137 |
|  | .133 | .143 | .133 | .136 | .150 | .147 | .144 | .168 | .150 | .181 |
|  | [-.325, .195] | [-.295, .266] | [-.484, .036] | [-.191, .341] | [-.288, .301] | [-.035, .541] | [-.304, .262] | [-.316, .340] | [-.229, .358] | [-.492, .217] |
|  | (.624) | (.920) | (.092) | (.582) | (.967) | (.085) | (.883) | (.943) | (.668) | (.447) |
| Rural Code=8 | -.543 | -.034 | -.065 | .285 | .185 | -.046 | .266 | -.057 | .375 | .043 |
|  | .248 | .268 | .249 | .254 | .282 | .276 | .271 | .312 | .280 | .339 |
|  | [-1.029, -.056] | [-.558, .491] | [-.552, .423] | [-.213, .782] | [-.368, .738] | [-.587, .495] | [-.264, .797] | [-.668, .553] | [-.175, .924] | [-.622, .708] |
|  | (.029) | (.900) | (.795) | (.262) | (.512) | (.867) | (.325) | (.854) | (.181) | (.900) |
| Rural Code=9 | -.072 | -.159 | -.127 | -.141 | -.104 | -.086 | -.442 | -.253 | -.380 | .233 |
|  | .211 | .228 | .211 | .216 | .239 | .234 | .230 | .265 | .238 | .288 |
|  | [-.485, .342] | [-.605, .287] | [-.541, .287] | [-.564, .282] | [-.574, .365] | [-.545, .372] | [-.893, .008] | [-.772, .266] | [-.847, .087] | [-.332, .797] |
|  | (.734) | (.485) | (.547) | (.512) | (.663) | (.712) | (.054) | (.340) | (.111) | (.419) |
| Alaska | -.038 | .753 | .209 | .216 | .060 | -.556 | .016 | -.033 | -.083 | -.338 |
|  | .423 | .455 | .423 | .431 | .480 | .469 | .460 | .531 | .477 | .578 |
|  | [-.866, .791] | [-.139, 1.645] | [-.619, 1.038] | [-.629, 1.062] | [-.881, 1.001] | [-1.476, .364] | [-.886, .918] | [-1.074, 1.007] | [-1.018, .851] | [-1.471, .796] |
|  | (.929) | (.098) | (.620) | (.616) | (.901) | (.236) | (.973) | (.950) | (.861) | (.559) |
| Arizona | .011 | -.145 | -.434 | -.282 | -.380 | -.059 | -.393 | -.312 | -.081 | -.804 |
|  | .204 | .218 | .203 | .207 | .228 | .223 | .220 | .257 | .229 | .277 |
|  | [-.388, .410] | [-.573, .282] | [-.832, -.037] | [-.687, .124] | [-.828, .067] | [-.497, .379] | [-.823, .038] | [-.815, .191] | [-.529, .367] | [-1.348, -.261] |
|  | (.957) | (.505) | (.032) | (.173) | (.096) | (.792) | (.074) | (.224) | (.723) | (.004) |
| Arkansas | .076 | .232 | -.076 | .084 | -.004 | .174 | .073 | .133 | -.019 | .004 |
|  | .229 | .245 | .227 | .232 | .257 | .251 | .247 | .286 | .256 | .311 |
|  | [-.373, .525] | [-.247, .711] | [-.521, .368] | [-.371, .538] | [-.507, .499] | [-.317, .666] | [-.410, .556] | [-.427, .694] | [-.520, .483] | [-.605, .613] |
|  | (.739) | (.343) | (.737) | (.717) | (.988) | (.487) | (.768) | (.641) | (.942) | (.991) |
| California | -.031 | .034 | -.284 | .022 | -.171 | -.041 | -.097 | -.037 | -.003 | -.472 |
|  | .179 | .191 | .178 | .182 | .201 | .197 | .193 | .225 | .200 | .245 |
|  | [-.382, .321] | [-.341, .409] | [-.632, .064] | [-.334, .378] | [-.566, .223] | [-.427, .344] | [-.476, .282] | [-.478, .404] | [-.396, .389] | [-.952, .008] |
|  | (.864) | (.859) | (.110) | (.905) | (.395) | (.833) | (.615) | (.870) | (.987) | (.054) |
| Colorado | -.380 | .174 | -.144 | -.075 | -.262 | .065 | -.097 | -.285 | -.323 | -.221 |
|  | .229 | .246 | .229 | .234 | .258 | .252 | .248 | .288 | .258 | .312 |
|  | [-.830, .069] | [-.309, .657] | [-.592, .304] | [-.533, .383] | [-.767, .243] | [-.429, .560] | [-.584, .389] | [-.849, .280] | [-.828, .182] | [-.833, .392] |
|  | (.097) | (.479) | (.529) | (.748) | (.310) | (.795) | (.694) | (.323) | (.210) | (.480) |
| Connecticut | .417 | .244 | -.238 | -.196 | -.192 | .362 | .069 | .072 | .183 | -.269 |
|  | .251 | .268 | .251 | .254 | .280 | .277 | .270 | .313 | .282 | .342 |
|  | [-.075, .910] | [-.280, .768] | [-.730, .253] | [-.694, .301] | [-.742, .357] | [-.180, .905] | [-.459, .597] | [-.541, .684] | [-.370, .737] | [-.940, .402] |
|  | (.097) | (.362) | (.342) | (.439) | (.493) | (.190) | (.799) | (.818) | (.516) | (.432) |
| Delaware | .087 | .143 | .062 | -.058 | .039 | -.249 | .166 | -.124 | .244 | -.892 |
|  | .322 | .347 | .322 | .329 | .362 | .354 | .349 | .404 | .363 | .438 |
|  | [-.544, .717] | [-.537, .823] | [-.569, .692] | [-.703, .587] | [-.671, .749] | [-.943, .445] | [-.518, .850] | [-.916, .668] | [-.466, .955] | [-1.750, -.035] |
|  | (.788) | (.680) | (.848) | (.861) | (.913) | (.482) | (.633) | (.758) | (.501) | (.041) |
| District of Columbia | -.237 | .014 | .094 | -.341 | -.409 | .327 | .295 | -.223 | .003 | .481 |
|  | .399 | .429 | .422 | .407 | .453 | .468 | .434 | .501 | .449 | .545 |
|  | [-1.018, .545] | [-.827, .855] | [-.732, .920] | [-1.138, .457] | [-1.296, .479] | [-.591, 1.245] | [-.555, 1.146] | [-1.205, .758] | [-.877, .884] | [-.588, 1.550] |
|  | (.553) | (.974) | (.823) | (.402) | (.367) | (.485) | (.496) | (.656) | (.994) | (.378) |
| Florida | -.069 | .094 | -.129 | -.119 | -.229 | .064 | -.162 | .060 | -.018 | -.523 |
|  | .181 | .194 | .180 | .184 | .203 | .199 | .195 | .228 | .203 | .248 |
|  | [-.425, .286] | [-.285, .474] | [-.482, .223] | [-.479, .240] | [-.628, .170] | [-.326, .454] | [-.545, .221] | [-.387, .506] | [-.415, .379] | [-1.008, -.038] |
|  | (.702) | (.626) | (.471) | (.515) | (.260) | (.749) | (.407) | (.793) | (.929) | (.035) |
| Georgia | -.145 | .120 | -.282 | -.083 | -.404 | .061 | -.108 | .020 | -.184 | -.264 |
|  | .196 | .210 | .194 | .199 | .221 | .215 | .212 | .246 | .220 | .267 |
|  | [-.530, .240] | [-.291, .532] | [-.664, .099] | [-.473, .307] | [-.836, .028] | [-.361, .483] | [-.523, .307] | [-.463, .502] | [-.614, .247] | [-.788, .260] |
|  | (.461) | (.566) | (.147) | (.678) | (.067) | (.778) | (.609) | (.937) | (.403) | (.324) |
| Hawaii | .352 | .065 | .029 | -.505 | -.143 | .479 | -.348 | .112 | -.232 | -.127 |
|  | .346 | .374 | .346 | .355 | .390 | .381 | .375 | .434 | .390 | .470 |
|  | [-.326, 1.030] | [-.667, .797] | [-.650, .708] | [-1.199, .190] | [-.907, .621] | [-.267, 1.226] | [-1.084, .388] | [-.739, .963] | [-.997, .533] | [-1.049, .795] |
|  | (.309) | (.862) | (.933) | (.155) | (.714) | (.208) | (.354) | (.797) | (.553) | (.786) |
| Idaho | -.234 | .008 | -.108 | -.101 | -.507 | -.370 | -.428 | .128 | -.461 | -.031 |
|  | .290 | .313 | .290 | .297 | .326 | .319 | .315 | .365 | .327 | .394 |
|  | [-.803, .335] | [-.606, .622] | [-.677, .461] | [-.684, .482] | [-1.147, .132] | [-.995, .255] | [-1.045, .188] | [-.587, .842] | [-1.102, .180] | [-.804, .742] |
|  | (.420) | (.980) | (.710) | (.734) | (.120) | (.246) | (.173) | (.726) | (.158) | (.937) |
| Illinois | -.173 | .219 | -.213 | .152 | -.086 | .156 | .030 | .247 | -.170 | -.112 |
|  | .193 | .207 | .192 | .196 | .217 | .212 | .208 | .243 | .216 | .263 |
|  | [-.552, .205] | [-.186, .624] | [-.589, .163] | [-.232, .536] | [-.511, .339] | [-.260, .572] | [-.378, .438] | [-.228, .723] | [-.594, .253] | [-.628, .404] |
|  | (.370) | (.290) | (.267) | (.438) | (.691) | (.462) | (.885) | (.308) | (.431) | (.671) |
| Indiana | -.027 | .001 | -.059 | .018 | -.182 | .063 | -.098 | -.162 | -.006 | -.199 |
|  | .214 | .228 | .212 | .217 | .240 | .235 | .230 | .268 | .239 | .291 |
|  | [-.446, .392] | [-.447, .448] | [-.474, .356] | [-.408, .444] | [-.653, .288] | [-.397, .523] | [-.549, .354] | [-.686, .362] | [-.474, .463] | [-.768, .371] |
|  | (.900) | (.998) | (.780) | (.934) | (.448) | (.788) | (.671) | (.545) | (.981) | (.495) |
| Iowa | -.119 | .331 | -.270 | .034 | -.497 | .336 | .104 | .117 | -.198 | -.146 |
|  | .252 | .271 | .251 | .256 | .285 | .279 | .274 | .317 | .283 | .345 |
|  | [-.613, .375] | [-.199, .861] | [-.763, .222] | [-.469, .537] | [-1.056, .062] | [-.210, .883] | [-.432, .640] | [-.504, .737] | [-.753, .358] | [-.822, .530] |
|  | (.637) | (.221) | (.282) | (.895) | (.082) | (.228) | (.705) | (.712) | (.486) | (.672) |
| Kansas | -.511 | -.282 | -.225 | .121 | -.077 | .216 | -.105 | -.479 | -.432 | -.715 |
|  | .305 | .328 | .305 | .311 | .345 | .337 | .331 | .383 | .344 | .417 |
|  | [-1.110, .087] | [-.926, .362] | [-.823, .372] | [-.490, .731] | [-.754, .599] | [-.445, .878] | [-.755, .544] | [-1.231, .272] | [-1.105, .242] | [-1.532, .101] |
|  | (.094) | (.390) | (.460) | (.699) | (.822) | (.521) | (.751) | (.211) | (.209) | (.086) |
| Kentucky | -.201 | .369 | -.076 | .149 | -.114 | .238 | .257 | .009 | .153 | -.257 |
|  | .232 | .249 | .231 | .237 | .261 | .255 | .251 | .291 | .260 | .316 |
|  | [-.655, .253] | [-.118, .856] | [-.528, .376] | [-.316, .613] | [-.625, .398] | [-.262, .738] | [-.234, .749] | [-.561, .579] | [-.357, .662] | [-.876, .363] |
|  | (.386) | (.137) | (.741) | (.530) | (.663) | (.350) | (.305) | (.976) | (.557) | (.417) |
| Louisiana | -.183 | .358 | -.208 | -.259 | -.210 | .388 | .040 | .164 | -.077 | -.082 |
|  | .233 | .250 | .232 | .237 | .262 | .256 | .252 | .292 | .261 | .317 |
|  | [-.639, .273] | [-.132, .848] | [-.663, .246] | [-.724, .206] | [-.724, .303] | [-.114, .889] | [-.454, .533] | [-.409, .737] | [-.589, .436] | [-.703, .539] |
|  | (.431) | (.152) | (.369) | (.274) | (.422) | (.130) | (.875) | (.576) | (.769) | (.796) |
| Maine | -.402 | .272 | .161 | .121 | .289 | .308 | .370 | .045 | -.488 | -.530 |
|  | .322 | .347 | .322 | .329 | .363 | .355 | .349 | .404 | .363 | .438 |
|  | [-1.033, .228] | [-.408, .952] | [-.470, .791] | [-.524, .766] | [-.422, 1.000] | [-.387, 1.003] | [-.314, 1.055] | [-.747, .837] | [-1.199, .223] | [-1.388, .328] |
|  | (.211) | (.433) | (.618) | (.713) | (.426) | (.385) | (.289) | (.912) | (.179) | (.226) |
| Maryland | .129 | -.116 | -.534 | -.313 | -.267 | -.034 | -.180 | -.168 | -.113 | -.261 |
|  | .230 | .247 | .229 | .234 | .261 | .253 | .249 | .289 | .258 | .314 |
|  | [-.323, .580] | [-.600, .368] | [-.983, -.085] | [-.772, .146] | [-.778, .244] | [-.531, .463] | [-.668, .308] | [-.734, .399] | [-.619, .393] | [-.876, .355] |
|  | (.576) | (.638) | (.020) | (.181) | (.306) | (.894) | (.469) | (.562) | (.662) | (.406) |
| Massachusetts | -.099 | -.179 | -.071 | -.224 | -.367 | .079 | -.068 | -.411 | .008 | -.037 |
|  | .217 | .232 | .215 | .220 | .243 | .238 | .234 | .272 | .243 | .295 |
|  | [-.523, .326] | [-.634, .276] | [-.494, .351] | [-.655, .208] | [-.844, .110] | [-.387, .546] | [-.527, .391] | [-.943, .122] | [-.468, .484] | [-.616, .541] |
|  | (.649) | (.440) | (.740) | (.310) | (.131) | (.739) | (.771) | (.131) | (.974) | (.899) |
| Michigan | -.169 | .191 | -.122 | -.074 | -.262 | .109 | .006 | -.107 | -.235 | -.452 |
|  | .193 | .207 | .192 | .196 | .217 | .212 | .209 | .243 | .216 | .264 |
|  | [-.548, .210] | [-.214, .596] | [-.498, .254] | [-.458, .310] | [-.688, .163] | [-.307, .525] | [-.403, .415] | [-.583, .368] | [-.659, .188] | [-.968, .065] |
|  | (.383) | (.356) | (.525) | (.707) | (.227) | (.608) | (.977) | (.658) | (.276) | (.087) |
| Minnesota | -.230 | .181 | -.555 | .144 | -.248 | -.071 | -.005 | -.365 | -.364 | -.741 |
|  | .227 | .244 | .226 | .231 | .256 | .251 | .248 | .286 | .256 | .310 |
|  | [-.676, .216] | [-.297, .659] | [-.998, -.111] | [-.309, .598] | [-.751, .254] | [-.562, .421] | [-.490, .480] | [-.925, .194] | [-.867, .138] | [-1.349, -.132] |
|  | (.311) | (.458) | (.014) | (.532) | (.333) | (.778) | (.984) | (.201) | (.155) | (.017) |
| Mississippi | -.076 | .253 | -.592 | .040 | -.320 | -.211 | .205 | -.031 | -.332 | -.082 |
|  | .259 | .276 | .256 | .261 | .290 | .284 | .278 | .322 | .289 | .351 |
|  | [-.583, .432] | [-.287, .794] | [-1.093, -.090] | [-.472, .553] | [-.889, .249] | [-.767, .345] | [-.340, .751] | [-.663, .601] | [-.897, .234] | [-.770, .605] |
|  | (.770) | (.358) | (.021) | (.877) | (.271) | (.456) | (.461) | (.924) | (.251) | (.815) |
| Missouri | -.283 | .355 | -.143 | .126 | -.208 | .327 | .043 | .022 | -.163 | -.056 |
|  | .209 | .224 | .208 | .212 | .236 | .230 | .226 | .262 | .234 | .285 |
|  | [-.693, .126] | [-.084, .793] | [-.550, .264] | [-.289, .542] | [-.669, .254] | [-.123, .777] | [-.399, .485] | [-.491, .536] | [-.621, .296] | [-.614, .503] |
|  | (.175) | (.113) | (.492) | (.551) | (.378) | (.155) | (.849) | (.932) | (.487) | (.845) |
| Montana | .194 | -.155 | -.461 | -.001 | -1.152 | -.791 | -.875 | -.333 | -.553 | -1.256 |
|  | .321 | .346 | .321 | .328 | .362 | .354 | .348 | .403 | .362 | .437 |
|  | [-.435, .823] | [-.833, .523] | [-1.090, .168] | [-.644, .642] | [-1.861, -.442] | [-1.485, -.098] | [-1.558, -.193] | [-1.123, .456] | [-1.262, .155] | [-2.112, -.400] |
|  | (.546) | (.654) | (.151) | (.997) | (.001) | (.025) | (.012) | (.408) | (.126) | (.004) |
| Nebraska | -.262 | .181 | -.043 | .351 | -.198 | -.312 | -.213 | -.214 | -.110 | -.263 |
|  | .339 | .364 | .338 | .335 | .373 | .376 | .358 | .426 | .370 | .463 |
|  | [-.926, .402] | [-.533, .895] | [-.706, .620] | [-.306, 1.008] | [-.929, .534] | [-1.048, .425] | [-.914, .489] | [-1.048, .620] | [-.836, .616] | [-1.171, .646] |
|  | (.439) | (.619) | (.899) | (.295) | (.597) | (.406) | (.552) | (.615) | (.767) | (.571) |
| Nevada | -.107 | -.035 | -.092 | -.055 | -.169 | .049 | -.120 | -.266 | -.322 | -.532 |
|  | .228 | .245 | .227 | .232 | .256 | .250 | .247 | .286 | .256 | .310 |
|  | [-.553, .340] | [-.514, .445] | [-.536, .353] | [-.510, .399] | [-.670, .331] | [-.441, .539] | [-.604, .365] | [-.826, .294] | [-.823, .179] | [-1.139, .075] |
|  | (.640) | (.886) | (.686) | (.811) | (.507) | (.844) | (.628) | (.352) | (.207) | (.086) |
| New Hampshire | -.051 | -.084 | -.347 | .137 | -.173 | .094 | .109 | .077 | .449 | -.728 |
|  | .272 | .294 | .272 | .278 | .307 | .300 | .295 | .342 | .307 | .371 |
|  | [-.585, .484] | [-.660, .491] | [-.881, .186] | [-.409, .683] | [-.774, .428] | [-.494, .681] | [-.470, .687] | [-.593, .748] | [-.152, 1.050] | [-1.455, -.002] |
|  | (.853) | (.774) | (.202) | (.623) | (.572) | (.755) | (.713) | (.821) | (.143) | (.049) |
| New Jersey | -.233 | -.097 | -.061 | -.243 | -.060 | .032 | -.114 | -.093 | .017 | -.345 |
|  | .210 | .225 | .209 | .214 | .237 | .232 | .228 | .265 | .236 | .288 |
|  | [-.645, .178] | [-.538, .345] | [-.470, .348] | [-.662, .176] | [-.525, .406] | [-.423, .487] | [-.560, .332] | [-.611, .426] | [-.445, .480] | [-.909, .220] |
|  | (.267) | (.668) | (.771) | (.255) | (.802) | (.889) | (.617) | (.726) | (.941) | (.231) |
| New Mexico | -.194 | .606 | -.047 | .119 | -.036 | .189 | .193 | .142 | -.129 | -.358 |
|  | .251 | .270 | .250 | .256 | .283 | .276 | .272 | .315 | .285 | .342 |
|  | [-.685, .298] | [.077, 1.134] | [-.538, .443] | [-.383, .620] | [-.590, .518] | [-.353, .730] | [-.340, .725] | [-.475, .759] | [-.687, .429] | [-1.028, .312] |
|  | (.440) | (.025) | (.850) | (.643) | (.899) | (.494) | (.478) | (.652) | (.652) | (.294) |
| New York | -.151 | .278 | -.254 | .066 | -.056 | .199 | .124 | .165 | -.015 | -.237 |
|  | .185 | .197 | .183 | .187 | .207 | .203 | .199 | .232 | .207 | .252 |
|  | [-.514, .211] | [-.109, .665] | [-.613, .105] | [-.300, .433] | [-.463, .350] | [-.199, .596] | [-.266, .514] | [-.290, .620] | [-.420, .390] | [-.731, .257] |
|  | (.412) | (.159) | (.166) | (.723) | (.787) | (.328) | (.534) | (.477) | (.942) | (.347) |
| North Carolina | -.076 | .066 | -.256 | -.142 | .183 | .210 | .044 | .071 | -.210 | -.226 |
|  | .199 | .213 | .198 | .202 | .224 | .219 | .215 | .250 | .223 | .272 |
|  | [-.467, .314] | [-.352, .483] | [-.644, .131] | [-.538, .254] | [-.255, .622] | [-.219, .639] | [-.377, .466] | [-.419, .561] | [-.647, .227] | [-.758, .306] |
|  | (.701) | (.758) | (.195) | (.482) | (.413) | (.338) | (.836) | (.777) | (.345) | (.405) |
| North Dakota | .277 | -.093 | -.591 | -.515 | -.595 | -.751 | -.536 | -.110 | -.166 | -1.478 |
|  | .402 | .435 | .403 | .412 | .454 | .444 | .437 | .505 | .454 | .548 |
|  | [-.512, 1.065] | [-.945, .759] | [-1.381, .199] | [-1.323, .293] | [-1.486, .295] | [-1.621, .120] | [-1.393, .321] | [-1.101, .881] | [-1.056, .724] | [-2.552, -.404] |
|  | (.492) | (.830) | (.142) | (.211) | (.190) | (.091) | (.220) | (.828) | (.714) | (.007) |
| Ohio | -.088 | .253 | -.260 | .091 | -.089 | .185 | -.017 | -.244 | -.228 | .073 |
|  | .194 | .207 | .192 | .196 | .218 | .213 | .209 | .243 | .217 | .264 |
|  | [-.468, .293] | [-.153, .659] | [-.637, .117] | [-.294, .476] | [-.515, .338] | [-.232, .602] | [-.427, .393] | [-.721, .232] | [-.652, .197] | [-.445, .591] |
|  | (.651) | (.222) | (.176) | (.642) | (.683) | (.383) | (.934) | (.315) | (.293) | (.782) |
| Oklahoma | -.284 | .037 | -.659 | -.162 | .005 | -.276 | -.255 | .033 | -.250 | -.640 |
|  | .258 | .277 | .257 | .263 | .292 | .285 | .280 | .324 | .290 | .353 |
|  | [-.791, .222] | [-.507, .580] | [-1.164, -.155] | [-.678, .353] | [-.567, .577] | [-.835, .283] | [-.804, .293] | [-.602, .669] | [-.819, .319] | [-1.331, .051] |
|  | (.271) | (.894) | (.010) | (.537) | (.987) | (.334) | (.362) | (.919) | (.388) | (.070) |
| Oregon | -.256 | -.007 | -.300 | .053 | -.829 | -.074 | -.410 | -.576 | .293 | -.419 |
|  | .215 | .230 | .214 | .219 | .241 | .235 | .232 | .269 | .241 | .292 |
|  | [-.676, .165] | [-.458, .445] | [-.719, .118] | [-.375, .481] | [-1.300, -.357] | [-.535, .387] | [-.864, .044] | [-1.104, -.048] | [-.178, .765] | [-.991, .153] |
|  | (.234) | (.977) | (.160) | (.808) | (.001) | (.752) | (.077) | (.032) | (.223) | (.151) |
| Pennsylvania | -.135 | .100 | -.168 | .097 | -.011 | .138 | .148 | -.189 | -.118 | -.075 |
|  | .184 | .196 | .182 | .186 | .206 | .202 | .198 | .230 | .205 | .251 |
|  | [-.494, .225] | [-.284, .484] | [-.525, .188] | [-.268, .461] | [-.415, .393] | [-.257, .533] | [-.240, .536] | [-.641, .263] | [-.520, .284] | [-.566, .416] |
|  | (.464) | (.610) | (.355) | (.603) | (.956) | (.493) | (.454) | (.412) | (.566) | (.765) |
| Rhode Island | -.080 | .884 | .030 | -.033 | .122 | .230 | .266 | .489 | -.255 | -.180 |
|  | .421 | .428 | .398 | .406 | .452 | .442 | .433 | .500 | .449 | .544 |
|  | [-.905, .745] | [.044, 1.724] | [-.750, .809] | [-.829, .763] | [-.764, 1.008] | [-.636, 1.096] | [-.583, 1.116] | [-.491, 1.469] | [-1.134, .624] | [-1.247, .887] |
|  | (.849) | (.039) | (.941) | (.935) | (.788) | (.602) | (.539) | (.328) | (.570) | (.740) |
| South Carolina | -.140 | -.093 | -.151 | -.003 | -.068 | .218 | .078 | -.382 | -.114 | -.200 |
|  | .225 | .241 | .224 | .229 | .252 | .247 | .243 | .282 | .252 | .306 |
|  | [-.580, .300] | [-.566, .380] | [-.589, .288] | [-.452, .446] | [-.563, .427] | [-.265, .702] | [-.398, .554] | [-.935, .171] | [-.608, .381] | [-.799, .400] |
|  | (.533) | (.699) | (.501) | (.989) | (.787) | (.376) | (.749) | (.176) | (.652) | (.514) |
| South Dakota | -.034 | .275 | -.573 | .069 | -.125 | .108 | -.215 | -.699 | -.216 | -.179 |
|  | .370 | .399 | .370 | .378 | .418 | .409 | .402 | .464 | .417 | .504 |
|  | [-.759, .691] | [-.506, 1.057] | [-1.298, .152] | [-.672, .810] | [-.945, .694] | [-.692, .909] | [-1.002, .573] | [-1.609, .211] | [-1.033, .601] | [-1.167, .809] |
|  | (.926) | (.490) | (.122) | (.855) | (.764) | (.791) | (.593) | (.132) | (.605) | (.723) |
| Tennessee | -.174 | -.150 | -.308 | -.240 | -.486 | -.131 | -.106 | .032 | -.006 | -.493 |
|  | .219 | .235 | .218 | .223 | .247 | .241 | .237 | .275 | .246 | .299 |
|  | [-.604, .256] | [-.611, .311] | [-.736, .120] | [-.678, .197] | [-.970, -.003] | [-.603, .342] | [-.571, .359] | [-.508, .572] | [-.488, .476] | [-1.079, .093] |
|  | (.428) | (.523) | (.158) | (.282) | (.049) | (.589) | (.656) | (.907) | (.980) | (.099) |
| Texas | -.121 | .075 | -.347 | -.094 | -.226 | .023 | -.012 | -.059 | -.182 | -.490 |
|  | .184 | .196 | .182 | .186 | .206 | .202 | .198 | .231 | .205 | .251 |
|  | [-.481, .239] | [-.309, .460] | [-.704, .010] | [-.459, .271] | [-.631, .178] | [-.373, .418] | [-.401, .376] | [-.511, .393] | [-.584, .220] | [-.982, .001] |
|  | (.508) | (.701) | (.057) | (.613) | (.272) | (.910) | (.950) | (.799) | (.376) | (.050) |
| Utah | -.016 | -.262 | -.507 | -.349 | -.320 | -.083 | -.026 | -.598 | .035 | -1.002 |
|  | .254 | .274 | .254 | .260 | .286 | .279 | .275 | .319 | .286 | .346 |
|  | [-.515, .482] | [-.798, .275] | [-1.004, -.009] | [-.858, .160] | [-.880, .240] | [-.631, .464] | [-.565, .514] | [-1.223, .028] | [-.526, .595] | [-1.680, -.324] |
|  | (.948) | (.339) | (.046) | (.179) | (.263) | (.765) | (.925) | (.061) | (.904) | (.004) |
| Vermont | .298 | -.059 | -.079 | -.028 | -.326 | .268 | -.279 | -.807 | -.425 | -1.391 |
|  | .364 | .392 | .364 | .372 | .414 | .404 | .396 | .458 | .411 | .498 |
|  | [-.416, 1.012] | [-.827, .710] | [-.792, .635] | [-.757, .700] | [-1.137, .485] | [-.525, 1.060] | [-1.056, .499] | [-1.704, .090] | [-1.230, .379] | [-2.368, -.414] |
|  | (.413) | (.881) | (.828) | (.939) | (.431) | (.508) | (.482) | (.078) | (.300) | (.005) |
| Virginia | -.066 | .111 | -.228 | -.072 | -.302 | .123 | -.076 | -.242 | .055 | -.532 |
|  | .197 | .211 | .196 | .200 | .221 | .216 | .213 | .248 | .221 | .268 |
|  | [-.452, .320] | [-.302, .524] | [-.611, .156] | [-.464, .320] | [-.735, .131] | [-.301, .547] | [-.493, .341] | [-.728, .243] | [-.377, .487] | [-1.058, -.006] |
|  | (.738) | (.599) | (.245) | (.718) | (.171) | (.571) | (.720) | (.328) | (.804) | (.047) |
| Washington | -.179 | -.151 | -.121 | -.172 | -.741 | -.178 | -.479 | -.338 | -.278 | -.891 |
|  | .206 | .222 | .206 | .210 | .232 | .227 | .223 | .259 | .232 | .281 |
|  | [-.583, .226] | [-.585, .284] | [-.524, .282] | [-.583, .239] | [-1.195, -.286] | [-.623, .266] | [-.916, -.042] | [-.846, .170] | [-.733, .176] | [-1.442, -.340] |
|  | (.387) | (.496) | (.556) | (.413) | (.001) | (.431) | (.032) | (.192) | (.230) | (.002) |
| West Virginia | -.150 | .041 | -.239 | -.418 | .048 | .234 | .128 | .150 | .021 | -.486 |
|  | .264 | .280 | .260 | .266 | .295 | .289 | .283 | .332 | .294 | .357 |
|  | [-.668, .368] | [-.508, .591] | [-.749, .271] | [-.939, .103] | [-.530, .626] | [-.332, .799] | [-.427, .683] | [-.501, .800] | [-.554, .596] | [-1.185, .213] |
|  | (.571) | (.882) | (.359) | (.116) | (.871) | (.418) | (.650) | (.652) | (.943) | (.173) |
| Wisconsin | .074 | -.016 | -.348 | -.095 | -.195 | .017 | -.159 | -.195 | -.418 | -.368 |
|  | .213 | .228 | .212 | .216 | .240 | .234 | .230 | .267 | .240 | .291 |
|  | [-.344, .491] | [-.464, .431] | [-.763, .067] | [-.519, .329] | [-.665, .275] | [-.442, .476] | [-.611, .292] | [-.719, .330] | [-.888, .051] | [-.938, .201] |
|  | (.730) | (.944) | (.101) | (.661) | (.415) | (.942) | (.489) | (.467) | (.081) | (.205) |
| Wyoming | -.624 | .968 | .147 | -.360 | .531 | -.496 | .673 | .922 | 1.029 | -.497 |
|  | .623 | .678 | .627 | .644 | .696 | .681 | .675 | .782 | .706 | .840 |
|  | [-1.844, .596] | [-.362, 2.298] | [-1.083, 1.377] | [-1.623, .903] | [-.834, 1.896] | [-1.830, .838] | [-.650, 1.997] | [-.611, 2.456] | [-.354, 2.412] | [-2.144, 1.149] |
|  | (.316) | (.154) | (.815) | (.576) | (.446) | (.466) | (.319) | (.238) | (.145) | (.554) |
| N | 2967 | 2973 | 2969 | 2970 | 2976 | 2973 | 2972 | 2967 | 2970 | 2975 |
| Pseudo-R^2^ |  |  |  |  |  |  |  |  |  |  |
| Model χ^2^ | 381.9 | 43.3 | 166.5 | 47.3 | 368.2 | 378.4 | 654.1 | 492.3 | 13.2 | 331.7 |
| d.f. | 87 | 87 | 87 | 87 | 87 | 87 | 87 | 87 | 87 | 87 |
| p-value | <.001 | <.001 | <.001 | <.001 | <.001 | <.001 | <.001 | <.001 | <.001 | <.001 |

S17 Table. Regression Results, Public Policies, Multilevel Models, Controlling for Deaths.

|  | (1) | (2) | (3) | (4) | (5) | (6) | (7) | (8) | (9) | (10) |
| --- | --- | --- | --- | --- | --- | --- | --- | --- | --- | --- |
|  | increasetrade | importtaxes | lowinterest | notreliant | noairtravel | banentry | entrymexico | quarantine | rescueUS | delayelections |
| main |  |  |  |  |  |  |  |  |  |  |
| Democrat | .629 | -.727 | -.254 | -.706 | -.362 | -.657 | -.993 | -.510 | .103 | -.327 |
|  | .050 | .054 | .050 | .051 | .057 | .055 | .054 | .063 | .056 | .068 |
|  | [.531, .726] | [-.832, -.622] | [-.351, -.156] | [-.806, -.606] | [-.473, -.251] | [-.765, -.548] | [-1.099, -.886] | [-.633, -.388] | [-.007, .213] | [-.461, -.194] |
|  | (<.001) | (<.001) | (<.001) | (<.001) | (<.001) | (<.001) | (<.001) | (<.001) | (.067) | (<.001) |
| Other | .116 | -.426 | -.288 | -.338 | -.249 | -.473 | -.532 | -.451 | -.071 | -.204 |
|  | .050 | .054 | .051 | .052 | .057 | .056 | .055 | .063 | .057 | .069 |
|  | [.017, .215] | [-.532, -.319] | [-.387, -.189] | [-.439, -.237] | [-.361, -.137] | [-.582, -.363] | [-.640, -.424] | [-.575, -.326] | [-.183, .041] | [-.339, -.069] |
|  | (.022) | (<.001) | (<.001) | (<.001) | (<.001) | (<.001) | (<.001) | (<.001) | (.214) | (.003) |
| Local COVID-19 Deaths | <.001 | <.001 | <.001 | <.001 | <.001 | <.001 | <.001 | <.001 | <.001 | <.001 |
|  | <.001 | <.001 | <.001 | <.001 | <.001 | <.001 | <.001 | <.001 | <.001 | <.001 |
|  | [-<.001, .000] | [-<.001, .000] | [-<.001, .000] | [-<.001, .000] | [-<.001, .000] | [-<.001, .000] | [-<.001, .000] | [-<.001, .000] | [-<.001, .000] | [-<.001, .000] |
|  | (.186) | (.093) | (.307) | (.797) | (.052) | (.432) | (.633) | (.392) | (.633) | (.473) |
| 35- | -.088 | .171 | .170 | .304 | .208 | .146 | .206 | .130 | .040 | -.118 |
|  | .056 | .060 | .056 | .057 | .064 | .062 | .061 | .070 | .063 | .076 |
|  | [-.198, .021] | [.053, .289] | [.060, .279] | [.192, .416] | [.083, .332] | [.024, .268] | [.086, .325] | [-.008, .267] | [-.084, .164] | [-.267, .032] |
|  | (.114) | (.004) | (.002) | (<.001) | (.001) | (.019) | (.001) | (.064) | (.525) | (.124) |
| 50- | -.008 | .129 | .209 | .451 | .180 | .286 | .399 | -.013 | .119 | -.384 |
|  | .053 | .057 | .053 | .054 | .060 | .059 | .058 | .066 | .060 | .072 |
|  | [-.112, .095] | [.018, .241] | [.105, .312] | [.345, .556] | [.062, .297] | [.171, .402] | [.286, .512] | [-.143, .117] | [.002, .236] | [-.526, -.243] |
|  | (.878) | (.023) | (<.001) | (<.001) | (.003) | (<.001) | (<.001) | (.847) | (.046) | (<.001) |
| 70- | .058 | .031 | .229 | .429 | .119 | .219 | .422 | -.197 | .279 | -.267 |
|  | .067 | .072 | .067 | .068 | .076 | .074 | .073 | .084 | .076 | .091 |
|  | [-.073, .189] | [-.110, .172] | [.098, .360] | [.295, .563] | [-.029, .268] | [.074, .365] | [.279, .564] | [-.362, -.033] | [.131, .427] | [-.446, -.088] |
|  | (.386) | (.664) | (.001) | (<.001) | (.115) | (.003) | (<.001) | (.019) | (<.001) | (.003) |
| gender | -.136 | .161 | .098 | -.064 | .257 | .114 | .011 | .107 | -.062 | .432 |
|  | .038 | .041 | .038 | .039 | .044 | .043 | .042 | .048 | .043 | .053 |
|  | [-.212, -.061] | [.080, .242] | [.022, .173] | [-.141, .013] | [.172, .343] | [.031, .198] | [-.071, .094] | [.012, .201] | [-.147, .023] | [.329, .535] |
|  | (<.001) | (<.001) | (.011) | (.103) | (<.001) | (.007) | (.787) | (.027) | (.150) | (<.001) |
| white | -.022 | -.096 | -.074 | -.101 | -.113 | -.032 | -.088 | -.283 | .049 | -.103 |
|  | .046 | .050 | .046 | .047 | .052 | .051 | .050 | .058 | .052 | .063 |
|  | [-.112, .069] | [-.193, .001] | [-.164, .016] | [-.193, -.009] | [-.215, -.010] | [-.133, .068] | [-.187, .010] | [-.396, -.170] | [-.053, .151] | [-.226, .020] |
|  | (.637) | (.053) | (.108) | (.032) | (.031) | (.527) | (.079) | (<.001) | (.343) | (.101) |
| married | -.034 | .125 | -.015 | <.001 | .074 | .018 | .006 | .069 | -.063 | -.059 |
|  | .042 | .045 | .042 | .043 | .048 | .047 | .046 | .053 | .048 | .058 |
|  | [-.117, .049] | [.036, .215] | [-.098, .068] | [-.084, .085] | [-.020, .168] | [-.074, .110] | [-.084, .096] | [-.035, .173] | [-.157, .030] | [-.173, .054] |
|  | (.420) | (.006) | (.717) | (.996) | (.125) | (.707) | (.897) | (.196) | (.185) | (.303) |
| High school graduate | -.330 | -.104 | -.040 | -.104 | .018 | -.315 | -.179 | -.452 | .059 | .007 |
|  | .113 | .121 | .113 | .115 | .128 | .126 | .123 | .141 | .128 | .155 |
|  | [-.551, -.108] | [-.342, .133] | [-.262, .182] | [-.329, .121] | [-.232, .269] | [-.562, -.069] | [-.419, .062] | [-.729, -.175] | [-.192, .310] | [-.298, .311] |
|  | (.004) | (.390) | (.725) | (.364) | (.886) | (.012) | (.145) | (.001) | (.645) | (.966) |
| Some college | -.300 | -.268 | -.085 | -.118 | -.221 | -.488 | -.335 | -.899 | .117 | -.203 |
|  | .115 | .124 | .115 | .117 | .130 | .128 | .125 | .144 | .131 | .158 |
|  | [-.525, -.074] | [-.510, -.025] | [-.311, .141] | [-.348, .111] | [-.477, .035] | [-.739, -.237] | [-.580, -.090] | [-1.182, -.617] | [-.139, .373] | [-.513, .107] |
|  | (.009) | (.030) | (.463) | (.312) | (.090) | (<.001) | (.007) | (<.001) | (.371) | (.200) |
| 2-year | -.257 | -.119 | -.060 | -.058 | -.121 | -.328 | -.244 | -.840 | .016 | -.091 |
|  | .121 | .130 | .121 | .123 | .137 | .135 | .132 | .151 | .137 | .166 |
|  | [-.494, -.020] | [-.373, .136] | [-.297, .178] | [-.299, .183] | [-.390, .148] | [-.592, -.064] | [-.502, .014] | [-1.137, -.543] | [-.253, .285] | [-.417, .235] |
|  | (.034) | (.360) | (.622) | (.637) | (.379) | (.015) | (.064) | (<.001) | (.904) | (.583) |
| 4-year | -.109 | -.410 | -.194 | -.302 | -.389 | -.682 | -.447 | -.964 | .058 | -.208 |
|  | .117 | .126 | .118 | .120 | .133 | .131 | .128 | .147 | .133 | .161 |
|  | [-.340, .121] | [-.657, -.162] | [-.424, .037] | [-.537, -.068] | [-.650, -.128] | [-.939, -.426] | [-.698, -.197] | [-1.253, -.676] | [-.203, .319] | [-.525, .108] |
|  | (.352) | (.001) | (.100) | (.012) | (.004) | (<.001) | (<.001) | (<.001) | (.663) | (.197) |
| Post-grad | -.046 | -.496 | -.217 | -.378 | -.580 | -.667 | -.581 | -1.136 | .013 | -.417 |
|  | .123 | .132 | .123 | .126 | .140 | .137 | .134 | .154 | .140 | .170 |
|  | [-.288, .196] | [-.755, -.236] | [-.459, .025] | [-.624, -.132] | [-.854, -.306] | [-.936, -.398] | [-.843, -.318] | [-1.438, -.833] | [-.261, .287] | [-.749, -.084] |
|  | (.710) | (<.001) | (.078) | (.003) | (<.001) | (<.001) | (<.001) | (<.001) | (.928) | (.014) |
| income=2 | -.186 | .140 | .142 | .005 | .154 | .159 | .175 | -.011 | .026 | .164 |
|  | .109 | .117 | .109 | .111 | .123 | .121 | .119 | .136 | .123 | .149 |
|  | [-.400, .027] | [-.090, .370] | [-.072, .357] | [-.213, .223] | [-.088, .396] | [-.078, .396] | [-.058, .408] | [-.279, .256] | [-.215, .267] | [-.128, .456] |
|  | (.086) | (.232) | (.193) | (.965) | (.213) | (.189) | (.141) | (.935) | (.834) | (.270) |
| income=3 | -.081 | .101 | .139 | .126 | .089 | .154 | .236 | -.232 | .143 | .209 |
|  | .105 | .113 | .106 | .108 | .119 | .117 | .115 | .132 | .119 | .144 |
|  | [-.287, .125] | [-.122, .323] | [-.068, .347] | [-.085, .337] | [-.146, .323] | [-.076, .384] | [.010, .461] | [-.491, .027] | [-.091, .377] | [-.074, .492] |
|  | (.439) | (.374) | (.188) | (.243) | (.458) | (.190) | (.041) | (.079) | (.230) | (.148) |
| income=4 | -.067 | .076 | .227 | .068 | .064 | -.015 | .132 | -.257 | .014 | .122 |
|  | .108 | .116 | .108 | .110 | .123 | .120 | .118 | .136 | .122 | .148 |
|  | [-.278, .145] | [-.152, .304] | [.014, .439] | [-.148, .285] | [-.176, .304] | [-.251, .220] | [-.099, .363] | [-.523, .008] | [-.225, .253] | [-.168, .411] |
|  | (.537) | (.513) | (.036) | (.535) | (.600) | (.899) | (.262) | (.058) | (.907) | (.411) |
| income=5 | -.072 | .012 | .107 | .189 | .051 | .090 | .239 | -.427 | .132 | .026 |
|  | .110 | .119 | .111 | .113 | .125 | .123 | .120 | .138 | .124 | .151 |
|  | [-.288, .143] | [-.221, .245] | [-.110, .324] | [-.032, .410] | [-.195, .296] | [-.150, .331] | [.003, .475] | [-.698, -.156] | [-.112, .376] | [-.269, .322] |
|  | (.511) | (.918) | (.335) | (.094) | (.686) | (.462) | (.047) | (.002) | (.290) | (.861) |
| income=6 | -.084 | -.017 | .135 | .252 | .112 | .145 | .258 | -.302 | .088 | .192 |
|  | .111 | .120 | .112 | .114 | .126 | .124 | .122 | .140 | .126 | .153 |
|  | [-.302, .133] | [-.251, .218] | [-.084, .354] | [.030, .475] | [-.136, .360] | [-.099, .388] | [.020, .496] | [-.575, -.028] | [-.158, .334] | [-.107, .491] |
|  | (.448) | (.890) | (.228) | (.026) | (.375) | (.244) | (.034) | (.031) | (.483) | (.208) |
| income=7 | -.136 | -.020 | .154 | .135 | .236 | .278 | .145 | -.305 | -.075 | .058 |
|  | .119 | .128 | .120 | .122 | .135 | .133 | .131 | .150 | .135 | .163 |
|  | [-.370, .097] | [-.272, .231] | [-.080, .389] | [-.104, .374] | [-.030, .501] | [.018, .538] | [-.111, .401] | [-.598, -.011] | [-.339, .188] | [-.262, .377] |
|  | (.252) | (.874) | (.198) | (.267) | (.082) | (.036) | (.267) | (.042) | (.576) | (.724) |
| income=8 | -.193 | -.003 | .201 | .141 | .023 | .045 | .197 | -.358 | .005 | .019 |
|  | .116 | .125 | .117 | .118 | .132 | .129 | .127 | .146 | .131 | .159 |
|  | [-.421, .035] | [-.248, .242] | [-.028, .429] | [-.091, .373] | [-.235, .282] | [-.208, .299] | [-.051, .446] | [-.644, -.073] | [-.251, .262] | [-.292, .331] |
|  | (.097) | (.982) | (.085) | (.234) | (.859) | (.727) | (.120) | (.014) | (.967) | (.904) |
| income=9 | .011 | -.167 | .162 | .063 | -.189 | .093 | .063 | -.482 | .010 | -.178 |
|  | .115 | .124 | .115 | .117 | .130 | .128 | .125 | .144 | .130 | .157 |
|  | [-.214, .236] | [-.410, .075] | [-.064, .388] | [-.167, .293] | [-.445, .066] | [-.158, .343] | [-.183, .309] | [-.764, -.199] | [-.244, .264] | [-.486, .131] |
|  | (.924) | (.176) | (.161) | (.589) | (.147) | (.468) | (.617) | (.001) | (.940) | (.258) |
| income=10 | .005 | -.176 | .163 | .013 | -.031 | -.025 | .148 | -.513 | .028 | -.190 |
|  | .119 | .129 | .120 | .122 | .135 | .133 | .130 | .150 | .135 | .163 |
|  | [-.228, .239] | [-.428, .076] | [-.072, .398] | [-.226, .252] | [-.296, .235] | [-.285, .236] | [-.107, .403] | [-.806, -.219] | [-.237, .292] | [-.510, .130] |
|  | (.965) | (.172) | (.174) | (.915) | (.821) | (.852) | (.256) | (.001) | (.838) | (.244) |
| income=11 | -.023 | -.210 | .177 | -.087 | -.174 | -.002 | .053 | -.632 | -.029 | .042 |
|  | .122 | .132 | .123 | .125 | .139 | .137 | .134 | .154 | .138 | .168 |
|  | [-.263, .217] | [-.469, .049] | [-.064, .418] | [-.333, .158] | [-.447, .099] | [-.270, .266] | [-.209, .316] | [-.934, -.331] | [-.300, .242] | [-.287, .371] |
|  | (.848) | (.112) | (.150) | (.486) | (.213) | (.988) | (.690) | (<.001) | (.834) | (.803) |
| income=12 | -.063 | -.145 | .126 | -.097 | -.104 | .027 | .220 | -.657 | .003 | -.191 |
|  | .135 | .146 | .135 | .138 | .153 | .150 | .147 | .170 | .152 | .185 |
|  | [-.328, .202] | [-.431, .141] | [-.140, .391] | [-.367, .173] | [-.404, .195] | [-.267, .321] | [-.068, .509] | [-.990, -.324] | [-.295, .301] | [-.553, .171] |
|  | (.640) | (.319) | (.354) | (.480) | (.495) | (.857) | (.134) | (<.001) | (.985) | (.301) |
| income=13 | .106 | -.373 | -.159 | -.189 | -.282 | .073 | .040 | -.810 | -.174 | -.261 |
|  | .188 | .203 | .189 | .190 | .214 | .207 | .203 | .234 | .213 | .255 |
|  | [-.263, .475] | [-.770, .025] | [-.529, .211] | [-.562, .184] | [-.702, .137] | [-.333, .479] | [-.358, .439] | [-1.269, -.352] | [-.591, .243] | [-.760, .239] |
|  | (.574) | (.066) | (.399) | (.321) | (.188) | (.725) | (.843) | (.001) | (.413) | (.306) |
| income=14 | -.127 | -.153 | .137 | .047 | -.131 | .150 | .214 | -.700 | -.321 | .035 |
|  | .197 | .212 | .197 | .201 | .224 | .219 | .215 | .247 | .223 | .270 |
|  | [-.513, .259] | [-.569, .263] | [-.251, .524] | [-.347, .442] | [-.570, .309] | [-.280, .581] | [-.208, .636] | [-1.185, -.215] | [-.757, .116] | [-.494, .564] |
|  | (.520) | (.471) | (.489) | (.815) | (.560) | (.493) | (.320) | (.005) | (.150) | (.897) |
| income=15 | .251 | -.163 | -.362 | .445 | .750 | .731 | -.376 | -.174 | 1.024 | .524 |
|  | .596 | .641 | .596 | .608 | .679 | .664 | .651 | .749 | .673 | .816 |
|  | [-.918, 1.419] | [-1.420, 1.093] | [-1.531, .806] | [-.746, 1.636] | [-.580, 2.081] | [-.570, 2.033] | [-1.652, .899] | [-1.641, 1.293] | [-.295, 2.342] | [-1.076, 2.124] |
|  | (.674) | (.799) | (.543) | (.464) | (.269) | (.271) | (.563) | (.816) | (.128) | (.521) |
| income=16 | -.181 | .040 | .381 | -.047 | .073 | .461 | .579 | -.660 | -.158 | .068 |
|  | .280 | .302 | .281 | .286 | .319 | .312 | .306 | .352 | .316 | .384 |
|  | [-.730, .369] | [-.552, .631] | [-.169, .931] | [-.607, .514] | [-.552, .698] | [-.150, 1.073] | [-.021, 1.178] | [-1.349, .030] | [-.778, .462] | [-.685, .820] |
|  | (.519) | (.895) | (.174) | (.871) | (.819) | (.139) | (.058) | (.061) | (.618) | (.860) |
| income=17 | -.243 | .021 | -.012 | .107 | -.063 | .108 | .193 | -.340 | -.061 | -.043 |
|  | .102 | .110 | .103 | .104 | .116 | .114 | .112 | .128 | .116 | .140 |
|  | [-.444, -.043] | [-.195, .237] | [-.214, .190] | [-.098, .311] | [-.290, .165] | [-.115, .331] | [-.026, .412] | [-.591, -.089] | [-.288, .165] | [-.318, .231] |
|  | (.017) | (.851) | (.907) | (.308) | (.590) | (.343) | (.084) | (.008) | (.595) | (.757) |
| Rural Code=2 | -.094 | -.016 | -.046 | .074 | -.016 | -.023 | -.070 | -.009 | -.104 | -.142 |
|  | .053 | .057 | .053 | .054 | .060 | .058 | .058 | .067 | .060 | .072 |
|  | [-.198, .010] | [-.129, .096] | [-.150, .058] | [-.033, .181] | [-.133, .101] | [-.138, .091] | [-.183, .043] | [-.140, .121] | [-.221, .014] | [-.283, -.001] |
|  | (.076) | (.778) | (.387) | (.174) | (.790) | (.692) | (.227) | (.892) | (.084) | (.048) |
| Rural Code=3 | -.068 | -.013 | .004 | .052 | .048 | .099 | .018 | .073 | -.123 | .021 |
|  | .072 | .077 | .072 | .073 | .080 | .079 | .078 | .090 | .081 | .097 |
|  | [-.208, .073] | [-.164, .139] | [-.137, .144] | [-.092, .195] | [-.109, .206] | [-.056, .253] | [-.134, .170] | [-.102, .249] | [-.281, .036] | [-.169, .211] |
|  | (.345) | (.871) | (.959) | (.480) | (.547) | (.210) | (.817) | (.414) | (.130) | (.830) |
| Rural Code=4 | -.192 | .080 | .091 | .131 | .096 | .064 | .268 | .170 | .071 | -.179 |
|  | .098 | .106 | .098 | .101 | .111 | .109 | .107 | .123 | .111 | .134 |
|  | [-.384, .000] | [-.128, .288] | [-.102, .284] | [-.066, .328] | [-.122, .314] | [-.149, .277] | [.058, .477] | [-.071, .412] | [-.147, .288] | [-.442, .083] |
|  | (.050) | (.451) | (.354) | (.194) | (.389) | (.555) | (.012) | (.167) | (.524) | (.180) |
| Rural Code=5 | -.058 | -.031 | -.133 | -.109 | .217 | <.001 | .028 | -.025 | -.326 | .156 |
|  | .150 | .161 | .150 | .156 | .169 | .166 | .163 | .188 | .170 | .204 |
|  | [-.352, .237] | [-.347, .286] | [-.426, .161] | [-.414, .197] | [-.115, .549] | [-.324, .325] | [-.291, .347] | [-.392, .343] | [-.660, .008] | [-.244, .555] |
|  | (.701) | (.849) | (.376) | (.485) | (.200) | (.998) | (.863) | (.895) | (.056) | (.445) |
| Rural Code=6 | -.108 | .137 | .089 | .101 | .148 | .100 | .094 | .223 | -.059 | -.003 |
|  | .098 | .105 | .098 | .100 | .110 | .108 | .106 | .122 | .110 | .133 |
|  | [-.300, .083] | [-.069, .343] | [-.102, .281] | [-.095, .297] | [-.068, .365] | [-.112, .311] | [-.114, .302] | [-.017, .463] | [-.275, .157] | [-.263, .258] |
|  | (.268) | (.193) | (.362) | (.311) | (.178) | (.356) | (.374) | (.068) | (.594) | (.984) |
| Rural Code=7 | -.042 | .017 | -.207 | .079 | .044 | .268 | -.012 | .030 | .074 | -.121 |
|  | .134 | .144 | .134 | .137 | .151 | .148 | .146 | .169 | .151 | .182 |
|  | [-.304, .220] | [-.266, .300] | [-.469, .056] | [-.189, .348] | [-.253, .341] | [-.022, .558] | [-.298, .273] | [-.301, .361] | [-.222, .370] | [-.478, .237] |
|  | (.752) | (.907) | (.123) | (.563) | (.771) | (.070) | (.933) | (.857) | (.626) | (.508) |
| Rural Code=8 | -.522 | -.005 | -.048 | .289 | .220 | -.032 | .275 | -.040 | .383 | .058 |
|  | .249 | .268 | .249 | .254 | .283 | .277 | .271 | .312 | .281 | .340 |
|  | [-1.009, -.034] | [-.530, .521] | [-.536, .440] | [-.210, .787] | [-.334, .774] | [-.575, .510] | [-.257, .806] | [-.652, .572] | [-.167, .934] | [-.608, .725] |
|  | (.036) | (.986) | (.846) | (.256) | (.437) | (.907) | (.311) | (.897) | (.173) | (.864) |
| Rural Code=9 | -.049 | -.128 | -.110 | -.137 | -.067 | -.071 | -.433 | -.234 | -.371 | .249 |
|  | .211 | .228 | .212 | .217 | .240 | .235 | .230 | .266 | .239 | .289 |
|  | [-.464, .365] | [-.575, .319] | [-.525, .306] | [-.561, .287] | [-.537, .403] | [-.532, .389] | [-.885, .018] | [-.755, .286] | [-.839, .097] | [-.317, .815] |
|  | (.816) | (.575) | (.605) | (.527) | (.780) | (.761) | (.060) | (.377) | (.121) | (.388) |
| Alaska | -.038 | .751 | .208 | .216 | .057 | -.557 | .015 | -.034 | -.084 | -.339 |
|  | .423 | .455 | .423 | .431 | .480 | .469 | .460 | .531 | .477 | .578 |
|  | [-.867, .790] | [-.141, 1.642] | [-.620, 1.036] | [-.630, 1.062] | [-.883, .998] | [-1.477, .363] | [-.887, .917] | [-1.075, 1.006] | [-1.018, .850] | [-1.472, .794] |
|  | (.928) | (.099) | (.622) | (.617) | (.905) | (.235) | (.974) | (.949) | (.860) | (.558) |
| Arizona | .015 | -.140 | -.432 | -.281 | -.374 | -.057 | -.392 | -.309 | -.079 | -.802 |
|  | .203 | .218 | .203 | .207 | .228 | .223 | .220 | .257 | .229 | .277 |
|  | [-.384, .414] | [-.568, .287] | [-.829, -.034] | [-.686, .124] | [-.822, .073] | [-.494, .381] | [-.822, .039] | [-.812, .194] | [-.527, .368] | [-1.345, -.258] |
|  | (.941) | (.520) | (.033) | (.174) | (.101) | (.800) | (.075) | (.229) | (.728) | (.004) |
| Arkansas | .076 | .230 | -.078 | .084 | -.006 | .174 | .072 | .132 | -.019 | .003 |
|  | .229 | .244 | .227 | .232 | .256 | .251 | .247 | .286 | .256 | .311 |
|  | [-.373, .525] | [-.249, .709] | [-.522, .367] | [-.371, .538] | [-.509, .496] | [-.318, .665] | [-.411, .555] | [-.428, .693] | [-.520, .482] | [-.606, .612] |
|  | (.741) | (.347) | (.732) | (.718) | (.981) | (.489) | (.770) | (.643) | (.940) | (.993) |
| California | -.048 | .010 | -.298 | .018 | -.201 | -.053 | -.104 | -.051 | -.011 | -.485 |
|  | .180 | .192 | .178 | .182 | .202 | .197 | .194 | .226 | .201 | .245 |
|  | [-.400, .305] | [-.367, .386] | [-.647, .051] | [-.339, .375] | [-.596, .194] | [-.440, .334] | [-.484, .276] | [-.493, .392] | [-.404, .383] | [-.966, -.004] |
|  | (.790) | (.960) | (.094) | (.920) | (.319) | (.788) | (.591) | (.822) | (.958) | (.048) |
| Colorado | -.376 | .179 | -.141 | -.074 | -.256 | .068 | -.096 | -.282 | -.322 | -.218 |
|  | .229 | .246 | .229 | .234 | .258 | .252 | .248 | .288 | .258 | .312 |
|  | [-.826, .074] | [-.304, .662] | [-.589, .307] | [-.533, .384] | [-.761, .249] | [-.426, .562] | [-.582, .390] | [-.846, .283] | [-.827, .183] | [-.830, .394] |
|  | (.101) | (.467) | (.537) | (.750) | (.321) | (.788) | (.699) | (.328) | (.212) | (.485) |
| Connecticut | .383 | .194 | -.266 | -.204 | -.253 | .339 | .054 | .043 | .169 | -.295 |
|  | .253 | .269 | .252 | .255 | .282 | .278 | .271 | .314 | .284 | .344 |
|  | [-.113, .878] | [-.334, .721] | [-.760, .228] | [-.704, .297] | [-.805, .300] | [-.206, .885] | [-.477, .586] | [-.574, .659] | [-.388, .726] | [-.970, .380] |
|  | (.130) | (.472) | (.291) | (.425) | (.370) | (.223) | (.841) | (.892) | (.552) | (.392) |
| Delaware | .086 | .142 | .062 | -.058 | .038 | -.249 | .166 | -.125 | .244 | -.892 |
|  | .322 | .347 | .322 | .329 | .362 | .354 | .349 | .404 | .363 | .437 |
|  | [-.544, .717] | [-.537, .822] | [-.568, .693] | [-.703, .587] | [-.672, .748] | [-.943, .445] | [-.518, .850] | [-.916, .667] | [-.467, .955] | [-1.750, -.035] |
|  | (.788) | (.681) | (.847) | (.860) | (.916) | (.481) | (.634) | (.758) | (.501) | (.041) |
| District of Columbia | -.237 | .013 | .094 | -.341 | -.410 | .326 | .295 | -.224 | .003 | .480 |
|  | .399 | .429 | .421 | .407 | .452 | .468 | .434 | .501 | .449 | .545 |
|  | [-1.018, .545] | [-.827, .854] | [-.733, .920] | [-1.138, .456] | [-1.297, .477] | [-.591, 1.244] | [-.556, 1.145] | [-1.205, .758] | [-.878, .884] | [-.588, 1.549] |
|  | (.553) | (.975) | (.824) | (.402) | (.365) | (.486) | (.497) | (.655) | (.994) | (.378) |
| Florida | -.062 | .103 | -.124 | -.118 | -.218 | .068 | -.159 | .065 | -.015 | -.518 |
|  | .181 | .194 | .180 | .184 | .203 | .199 | .195 | .228 | .203 | .248 |
|  | [-.418, .293] | [-.276, .483] | [-.477, .228] | [-.478, .242] | [-.617, .180] | [-.322, .458] | [-.542, .224] | [-.381, .512] | [-.413, .382] | [-1.004, -.033] |
|  | (.731) | (.594) | (.489) | (.520) | (.283) | (.733) | (.415) | (.775) | (.940) | (.036) |
| Georgia | -.137 | .131 | -.277 | -.081 | -.392 | .066 | -.105 | .026 | -.181 | -.258 |
|  | .196 | .210 | .195 | .199 | .221 | .215 | .212 | .246 | .220 | .268 |
|  | [-.522, .248] | [-.281, .542] | [-.658, .105] | [-.471, .309] | [-.824, .041] | [-.357, .488] | [-.520, .310] | [-.457, .509] | [-.611, .250] | [-.783, .266] |
|  | (.486) | (.534) | (.155) | (.683) | (.076) | (.761) | (.619) | (.916) | (.411) | (.334) |
| Hawaii | .351 | .063 | .028 | -.505 | -.145 | .478 | -.348 | .111 | -.232 | -.128 |
|  | .346 | .373 | .346 | .355 | .390 | .381 | .375 | .434 | .390 | .470 |
|  | [-.327, 1.029] | [-.669, .795] | [-.650, .707] | [-1.200, .190] | [-.909, .618] | [-.268, 1.225] | [-1.084, .387] | [-.740, .962] | [-.997, .533] | [-1.050, .794] |
|  | (.310) | (.865) | (.935) | (.154) | (.709) | (.209) | (.354) | (.798) | (.552) | (.786) |
| Idaho | -.235 | .005 | -.109 | -.101 | -.510 | -.371 | -.429 | .127 | -.462 | -.033 |
|  | .290 | .313 | .290 | .297 | .326 | .319 | .315 | .365 | .327 | .394 |
|  | [-.804, .334] | [-.609, .619] | [-.678, .459] | [-.684, .481] | [-1.150, .129] | [-.997, .254] | [-1.045, .188] | [-.588, .841] | [-1.103, .179] | [-.806, .740] |
|  | (.418) | (.987) | (.706) | (.733) | (.118) | (.245) | (.173) | (.728) | (.158) | (.933) |
| Illinois | -.226 | .146 | -.254 | .141 | -.175 | .121 | .009 | .205 | -.192 | -.151 |
|  | .197 | .211 | .196 | .200 | .222 | .217 | .213 | .248 | .221 | .269 |
|  | [-.612, .161] | [-.267, .560] | [-.638, .130] | [-.251, .533] | [-.609, .259] | [-.304, .546] | [-.408, .426] | [-.280, .691] | [-.624, .241] | [-.679, .376] |
|  | (.253) | (.488) | (.195) | (.480) | (.429) | (.577) | (.966) | (.407) | (.385) | (.574) |
| Indiana | -.025 | .002 | -.059 | .018 | -.181 | .064 | -.098 | -.161 | -.005 | -.198 |
|  | .214 | .228 | .212 | .217 | .240 | .235 | .230 | .267 | .239 | .291 |
|  | [-.444, .393] | [-.446, .449] | [-.474, .356] | [-.408, .444] | [-.651, .289] | [-.396, .523] | [-.549, .354] | [-.685, .363] | [-.473, .463] | [-.768, .372] |
|  | (.905) | (.994) | (.782) | (.934) | (.450) | (.786) | (.672) | (.547) | (.982) | (.496) |
| Iowa | -.120 | .329 | -.272 | .034 | -.500 | .335 | .103 | .116 | -.198 | -.147 |
|  | .252 | .270 | .251 | .256 | .285 | .279 | .274 | .317 | .283 | .345 |
|  | [-.614, .374] | [-.201, .859] | [-.764, .220] | [-.469, .536] | [-1.059, .059] | [-.211, .882] | [-.433, .639] | [-.505, .736] | [-.753, .357] | [-.823, .528] |
|  | (.634) | (.224) | (.279) | (.896) | (.080) | (.229) | (.707) | (.715) | (.484) | (.669) |
| Kansas | -.505 | -.275 | -.221 | .122 | -.069 | .220 | -.103 | -.475 | -.430 | -.711 |
|  | .305 | .328 | .305 | .311 | .345 | .337 | .331 | .383 | .344 | .417 |
|  | [-1.104, .093] | [-.919, .369] | [-.818, .376] | [-.489, .732] | [-.745, .607] | [-.442, .881] | [-.753, .546] | [-1.226, .276] | [-1.103, .244] | [-1.528, .105] |
|  | (.098) | (.402) | (.468) | (.696) | (.842) | (.515) | (.755) | (.215) | (.211) | (.088) |
| Kentucky | -.199 | .371 | -.075 | .149 | -.111 | .239 | .258 | .010 | .153 | -.256 |
|  | .232 | .248 | .231 | .237 | .261 | .255 | .251 | .291 | .260 | .316 |
|  | [-.653, .255] | [-.116, .858] | [-.527, .377] | [-.315, .614] | [-.623, .400] | [-.261, .740] | [-.234, .749] | [-.560, .580] | [-.356, .663] | [-.875, .364] |
|  | (.391) | (.135) | (.745) | (.529) | (.670) | (.348) | (.304) | (.972) | (.556) | (.419) |
| Louisiana | -.186 | .353 | -.211 | -.260 | -.216 | .385 | .038 | .161 | -.078 | -.085 |
|  | .233 | .250 | .232 | .237 | .262 | .256 | .252 | .292 | .261 | .317 |
|  | [-.642, .270] | [-.137, .843] | [-.666, .244] | [-.725, .205] | [-.729, .297] | [-.116, .887] | [-.455, .532] | [-.412, .734] | [-.591, .434] | [-.706, .537] |
|  | (.424) | (.158) | (.363) | (.273) | (.409) | (.132) | (.880) | (.582) | (.765) | (.789) |
| Maine | -.403 | .271 | .160 | .121 | .287 | .307 | .370 | .044 | -.488 | -.530 |
|  | .322 | .347 | .322 | .329 | .363 | .355 | .349 | .404 | .363 | .438 |
|  | [-1.033, .228] | [-.409, .951] | [-.471, .790] | [-.524, .766] | [-.423, .998] | [-.388, 1.002] | [-.314, 1.054] | [-.748, .836] | [-1.199, .223] | [-1.389, .328] |
|  | (.211) | (.435) | (.620) | (.713) | (.428) | (.386) | (.289) | (.913) | (.178) | (.226) |
| Maryland | .137 | -.106 | -.528 | -.312 | -.255 | -.029 | -.177 | -.161 | -.110 | -.255 |
|  | .230 | .247 | .229 | .234 | .261 | .254 | .249 | .289 | .258 | .314 |
|  | [-.315, .588] | [-.590, .378] | [-.977, -.079] | [-.771, .147] | [-.766, .256] | [-.526, .468] | [-.666, .311] | [-.728, .405] | [-.616, .396] | [-.871, .360] |
|  | (.553) | (.668) | (.021) | (.183) | (.328) | (.909) | (.476) | (.577) | (.671) | (.417) |
| Massachusetts | -.126 | -.218 | -.093 | -.229 | -.413 | .061 | -.079 | -.433 | -.003 | -.058 |
|  | .217 | .233 | .216 | .221 | .244 | .239 | .235 | .273 | .244 | .296 |
|  | [-.552, .300] | [-.675, .239] | [-.517, .331] | [-.663, .204] | [-.892, .066] | [-.407, .530] | [-.540, .382] | [-.968, .102] | [-.482, .475] | [-.639, .523] |
|  | (.563) | (.351) | (.667) | (.300) | (.091) | (.798) | (.737) | (.113) | (.989) | (.845) |
| Michigan | -.199 | .149 | -.146 | -.080 | -.313 | .089 | -.006 | -.132 | -.248 | -.474 |
|  | .195 | .208 | .193 | .197 | .219 | .214 | .210 | .244 | .218 | .265 |
|  | [-.580, .183] | [-.259, .556] | [-.524, .233] | [-.467, .307] | [-.742, .116] | [-.330, .508] | [-.418, .406] | [-.611, .347] | [-.675, .179] | [-.994, .046] |
|  | (.307) | (.475) | (.451) | (.686) | (.152) | (.678) | (.977) | (.589) | (.255) | (.074) |
| Minnesota | -.225 | .188 | -.551 | .145 | -.240 | -.067 | -.003 | -.361 | -.362 | -.737 |
|  | .227 | .244 | .226 | .231 | .256 | .251 | .248 | .286 | .257 | .310 |
|  | [-.670, .221] | [-.290, .665] | [-.994, -.107] | [-.308, .599] | [-.742, .262] | [-.559, .424] | [-.488, .482] | [-.921, .199] | [-.865, .140] | [-1.346, -.129] |
|  | (.323) | (.441) | (.015) | (.529) | (.349) | (.788) | (.990) | (.206) | (.158) | (.018) |
| Mississippi | -.076 | .252 | -.593 | .040 | -.322 | -.212 | .205 | -.031 | -.332 | -.083 |
|  | .259 | .276 | .256 | .261 | .290 | .284 | .278 | .322 | .289 | .351 |
|  | [-.583, .432] | [-.288, .792] | [-1.094, -.091] | [-.472, .552] | [-.890, .247] | [-.768, .344] | [-.341, .751] | [-.663, .601] | [-.897, .234] | [-.770, .605] |
|  | (.770) | (.360) | (.020) | (.878) | (.268) | (.455) | (.462) | (.923) | (.250) | (.813) |
| Missouri | -.278 | .361 | -.139 | .127 | -.200 | .330 | .045 | .027 | -.161 | -.052 |
|  | .209 | .224 | .208 | .212 | .235 | .230 | .226 | .262 | .234 | .285 |
|  | [-.687, .132] | [-.077, .800] | [-.546, .268] | [-.288, .543] | [-.661, .262] | [-.120, .780] | [-.397, .487] | [-.487, .541] | [-.619, .298] | [-.610, .506] |
|  | (.184) | (.106) | (.503) | (.548) | (.396) | (.151) | (.842) | (.919) | (.492) | (.855) |
| Montana | .189 | -.162 | -.465 | -.002 | -1.160 | -.795 | -.877 | -.337 | -.555 | -1.260 |
|  | .321 | .346 | .321 | .328 | .362 | .354 | .348 | .403 | .362 | .437 |
|  | [-.440, .818] | [-.840, .516] | [-1.094, .163] | [-.645, .641] | [-1.869, -.451] | [-1.488, -.101] | [-1.560, -.195] | [-1.127, .453] | [-1.264, .153] | [-2.116, -.404] |
|  | (.555) | (.640) | (.147) | (.995) | (.001) | (.025) | (.012) | (.403) | (.125) | (.004) |
| Nebraska | -.265 | .176 | -.046 | .350 | -.203 | -.314 | -.214 | -.217 | -.111 | -.265 |
|  | .339 | .364 | .338 | .335 | .373 | .376 | .358 | .426 | .370 | .463 |
|  | [-.930, .399] | [-.538, .890] | [-.709, .617] | [-.307, 1.007] | [-.934, .527] | [-1.051, .422] | [-.915, .487] | [-1.051, .617] | [-.837, .615] | [-1.174, .643] |
|  | (.433) | (.629) | (.892) | (.296) | (.585) | (.403) | (.550) | (.611) | (.764) | (.567) |
| Nevada | -.105 | -.034 | -.091 | -.055 | -.168 | .049 | -.119 | -.265 | -.322 | -.531 |
|  | .228 | .245 | .227 | .232 | .255 | .250 | .247 | .286 | .256 | .310 |
|  | [-.551, .341] | [-.513, .445] | [-.535, .353] | [-.510, .400] | [-.669, .332] | [-.440, .539] | [-.604, .365] | [-.825, .295] | [-.823, .179] | [-1.138, .076] |
|  | (.644) | (.889) | (.688) | (.812) | (.509) | (.843) | (.629) | (.354) | (.208) | (.086) |
| New Hampshire | -.047 | -.080 | -.345 | .138 | -.169 | .095 | .110 | .080 | .450 | -.727 |
|  | .272 | .293 | .272 | .278 | .306 | .300 | .295 | .342 | .307 | .371 |
|  | [-.581, .487] | [-.656, .495] | [-.878, .188] | [-.408, .683] | [-.770, .432] | [-.492, .683] | [-.469, .688] | [-.591, .750] | [-.151, 1.051] | [-1.453, -<.001] |
|  | (.862) | (.784) | (.205) | (.621) | (.581) | (.751) | (.710) | (.815) | (.142) | (.050) |
| New Jersey | -.253 | -.123 | -.076 | -.247 | -.091 | .020 | -.122 | -.108 | .010 | -.359 |
|  | .211 | .226 | .209 | .214 | .238 | .233 | .228 | .265 | .236 | .289 |
|  | [-.666, .160] | [-.566, .319] | [-.486, .334] | [-.667, .173] | [-.558, .375] | [-.436, .476] | [-.569, .326] | [-.628, .412] | [-.454, .473] | [-.924, .207] |
|  | (.229) | (.585) | (.715) | (.249) | (.701) | (.932) | (.595) | (.683) | (.968) | (.214) |
| New Mexico | -.194 | .604 | -.048 | .118 | -.037 | .188 | .192 | .141 | -.129 | -.359 |
|  | .251 | .270 | .250 | .256 | .282 | .276 | .272 | .315 | .285 | .342 |
|  | [-.686, .297] | [.076, 1.133] | [-.538, .442] | [-.383, .620] | [-.591, .516] | [-.353, .729] | [-.340, .725] | [-.476, .759] | [-.687, .429] | [-1.029, .311] |
|  | (.439) | (.025) | (.847) | (.643) | (.895) | (.496) | (.479) | (.653) | (.651) | (.293) |
| New York | -.162 | .262 | -.263 | .064 | -.075 | .191 | .119 | .156 | -.020 | -.245 |
|  | .185 | .197 | .183 | .187 | .208 | .203 | .199 | .232 | .207 | .252 |
|  | [-.525, .200] | [-.125, .649] | [-.622, .097] | [-.303, .431] | [-.482, .332] | [-.207, .589] | [-.271, .510] | [-.299, .611] | [-.425, .385] | [-.740, .250] |
|  | (.379) | (.184) | (.152) | (.733) | (.718) | (.347) | (.550) | (.503) | (.924) | (.332) |
| North Carolina | -.071 | .072 | -.252 | -.141 | .191 | .213 | .046 | .075 | -.208 | -.222 |
|  | .199 | .213 | .198 | .202 | .224 | .219 | .215 | .250 | .223 | .272 |
|  | [-.461, .319] | [-.345, .490] | [-.640, .135] | [-.537, .255] | [-.247, .630] | [-.216, .642] | [-.375, .468] | [-.415, .565] | [-.645, .228] | [-.755, .310] |
|  | (.721) | (.735) | (.202) | (.485) | (.393) | (.330) | (.829) | (.764) | (.350) | (.413) |
| North Dakota | .275 | -.097 | -.593 | -.516 | -.599 | -.752 | -.537 | -.111 | -.167 | -1.479 |
|  | .402 | .434 | .403 | .412 | .454 | .444 | .437 | .505 | .454 | .548 |
|  | [-.514, 1.063] | [-.948, .755] | [-1.383, .196] | [-1.324, .292] | [-1.489, .291] | [-1.623, .118] | [-1.394, .320] | [-1.102, .879] | [-1.057, .723] | [-2.553, -.406] |
|  | (.495) | (.824) | (.141) | (.211) | (.187) | (.090) | (.219) | (.825) | (.713) | (.007) |
| Ohio | -.085 | .257 | -.258 | .092 | -.084 | .187 | -.016 | -.242 | -.227 | .075 |
|  | .194 | .207 | .192 | .196 | .217 | .213 | .209 | .243 | .217 | .264 |
|  | [-.465, .296] | [-.149, .662] | [-.635, .119] | [-.293, .477] | [-.511, .342] | [-.230, .604] | [-.426, .394] | [-.718, .235] | [-.651, .198] | [-.442, .593] |
|  | (.663) | (.215) | (.180) | (.640) | (.698) | (.379) | (.938) | (.320) | (.295) | (.776) |
| Oklahoma | -.281 | .041 | -.657 | -.162 | .010 | -.274 | -.254 | .036 | -.249 | -.638 |
|  | .258 | .277 | .257 | .263 | .292 | .285 | .280 | .324 | .290 | .353 |
|  | [-.787, .225] | [-.502, .584] | [-1.161, -.152] | [-.677, .354] | [-.562, .581] | [-.833, .285] | [-.803, .295] | [-.600, .671] | [-.818, .320] | [-1.329, .054] |
|  | (.277) | (.882) | (.011) | (.539) | (.974) | (.337) | (.364) | (.912) | (.391) | (.071) |
| Oregon | -.247 | .004 | -.294 | .055 | -.816 | -.069 | -.407 | -.570 | .297 | -.414 |
|  | .215 | .230 | .214 | .219 | .241 | .235 | .232 | .269 | .241 | .292 |
|  | [-.668, .173] | [-.448, .455] | [-.713, .124] | [-.374, .483] | [-1.287, -.344] | [-.531, .392] | [-.861, .047] | [-1.098, -.042] | [-.175, .768] | [-.986, .159] |
|  | (.249) | (.987) | (.168) | (.802) | (.001) | (.769) | (.079) | (.034) | (.218) | (.157) |
| Pennsylvania | -.138 | .094 | -.172 | .096 | -.019 | .135 | .146 | -.192 | -.120 | -.078 |
|  | .184 | .196 | .182 | .186 | .206 | .202 | .198 | .230 | .205 | .251 |
|  | [-.498, .221] | [-.290, .478] | [-.529, .185] | [-.269, .460] | [-.423, .385] | [-.260, .530] | [-.242, .534] | [-.644, .259] | [-.522, .282] | [-.569, .413] |
|  | (.451) | (.632) | (.345) | (.607) | (.928) | (.502) | (.460) | (.404) | (.560) | (.755) |
| Rhode Island | -.065 | .903 | .040 | -.030 | .145 | .240 | .272 | .500 | -.249 | -.170 |
|  | .421 | .428 | .398 | .406 | .452 | .442 | .433 | .500 | .449 | .545 |
|  | [-.890, .760] | [.064, 1.743] | [-.739, .820] | [-.826, .766] | [-.741, 1.030] | [-.627, 1.106] | [-.577, 1.121] | [-.480, 1.481] | [-1.129, .630] | [-1.237, .897] |
|  | (.877) | (.035) | (.919) | (.941) | (.749) | (.588) | (.530) | (.317) | (.578) | (.755) |
| South Carolina | -.138 | -.091 | -.149 | -.003 | -.065 | .220 | .079 | -.380 | -.113 | -.198 |
|  | .225 | .241 | .224 | .229 | .252 | .247 | .243 | .282 | .252 | .306 |
|  | [-.578, .303] | [-.564, .382] | [-.588, .289] | [-.451, .446] | [-.560, .429] | [-.264, .703] | [-.397, .555] | [-.933, .173] | [-.607, .381] | [-.798, .401] |
|  | (.540) | (.706) | (.505) | (.990) | (.796) | (.374) | (.746) | (.178) | (.654) | (.516) |
| South Dakota | -.036 | .272 | -.575 | .068 | -.130 | .107 | -.216 | -.701 | -.217 | -.181 |
|  | .370 | .399 | .370 | .378 | .418 | .409 | .402 | .464 | .417 | .504 |
|  | [-.761, .688] | [-.509, 1.053] | [-1.299, .150] | [-.673, .810] | [-.948, .689] | [-.694, .908] | [-1.003, .572] | [-1.611, .209] | [-1.034, .600] | [-1.169, .807] |
|  | (.922) | (.495) | (.120) | (.856) | (.756) | (.794) | (.591) | (.131) | (.603) | (.720) |
| Tennessee | -.170 | -.146 | -.305 | -.240 | -.481 | -.128 | -.104 | .035 | -.005 | -.490 |
|  | .219 | .235 | .218 | .223 | .247 | .241 | .237 | .275 | .246 | .299 |
|  | [-.600, .260] | [-.607, .315] | [-.733, .122] | [-.677, .198] | [-.964, .003] | [-.601, .345] | [-.569, .360] | [-.505, .575] | [-.487, .477] | [-1.077, .096] |
|  | (.438) | (.536) | (.162) | (.283) | (.051) | (.595) | (.660) | (.898) | (.984) | (.101) |
| Texas | -.113 | .086 | -.341 | -.092 | -.213 | .028 | -.009 | -.052 | -.179 | -.484 |
|  | .184 | .196 | .182 | .186 | .206 | .202 | .198 | .231 | .205 | .251 |
|  | [-.473, .247] | [-.298, .471] | [-.698, .016] | [-.457, .272] | [-.618, .191] | [-.368, .423] | [-.398, .379] | [-.504, .400] | [-.581, .224] | [-.976, .007] |
|  | (.539) | (.660) | (.061) | (.620) | (.301) | (.890) | (.962) | (.821) | (.384) | (.053) |
| Utah | -.012 | -.256 | -.504 | -.348 | -.314 | -.081 | -.024 | -.594 | .036 | -.999 |
|  | .254 | .274 | .254 | .260 | .286 | .279 | .275 | .319 | .286 | .346 |
|  | [-.510, .486] | [-.793, .280] | [-1.001, -.006] | [-.857, .161] | [-.873, .246] | [-.628, .467] | [-.564, .515] | [-1.220, .032] | [-.524, .597] | [-1.677, -.321] |
|  | (.962) | (.349) | (.047) | (.180) | (.272) | (.772) | (.930) | (.063) | (.899) | (.004) |
| Vermont | .294 | -.065 | -.083 | -.029 | -.334 | .264 | -.281 | -.811 | -.427 | -1.394 |
|  | .364 | .392 | .364 | .372 | .413 | .404 | .396 | .458 | .411 | .498 |
|  | [-.420, 1.008] | [-.834, .703] | [-.796, .631] | [-.758, .699] | [-1.144, .476] | [-.528, 1.057] | [-1.058, .497] | [-1.708, .086] | [-1.232, .377] | [-2.371, -.418] |
|  | (.420) | (.867) | (.820) | (.937) | (.419) | (.513) | (.479) | (.076) | (.298) | (.005) |
| Virginia | -.055 | .125 | -.220 | -.070 | -.285 | .129 | -.072 | -.234 | .059 | -.524 |
|  | .197 | .211 | .196 | .200 | .221 | .216 | .213 | .248 | .221 | .269 |
|  | [-.441, .331] | [-.288, .539] | [-.603, .164] | [-.463, .322] | [-.718, .148] | [-.295, .554] | [-.490, .345] | [-.719, .252] | [-.374, .491] | [-1.051, .002] |
|  | (.780) | (.553) | (.261) | (.726) | (.197) | (.550) | (.734) | (.346) | (.789) | (.051) |
| Washington | -.178 | -.151 | -.122 | -.172 | -.741 | -.178 | -.479 | -.338 | -.279 | -.891 |
|  | .206 | .222 | .206 | .210 | .232 | .227 | .223 | .259 | .232 | .281 |
|  | [-.582, .227] | [-.586, .283] | [-.525, .281] | [-.583, .239] | [-1.195, -.287] | [-.623, .266] | [-.916, -.042] | [-.845, .170] | [-.733, .176] | [-1.442, -.340] |
|  | (.389) | (.495) | (.554) | (.413) | (.001) | (.431) | (.032) | (.193) | (.230) | (.002) |
| West Virginia | -.150 | .040 | -.239 | -.418 | .047 | .233 | .128 | .150 | .021 | -.487 |
|  | .264 | .280 | .260 | .266 | .295 | .288 | .283 | .332 | .293 | .357 |
|  | [-.668, .368] | [-.509, .590] | [-.749, .271] | [-.939, .103] | [-.531, .625] | [-.332, .798] | [-.427, .683] | [-.501, .800] | [-.554, .596] | [-1.185, .212] |
|  | (.571) | (.885) | (.358) | (.116) | (.874) | (.419) | (.651) | (.652) | (.944) | (.172) |
| Wisconsin | .076 | -.013 | -.346 | -.095 | -.192 | .018 | -.159 | -.193 | -.418 | -.367 |
|  | .213 | .228 | .212 | .216 | .240 | .234 | .230 | .267 | .240 | .291 |
|  | [-.341, .493] | [-.461, .434] | [-.761, .069] | [-.519, .330] | [-.662, .277] | [-.441, .478] | [-.610, .293] | [-.717, .332] | [-.887, .052] | [-.936, .202] |
|  | (.721) | (.953) | (.102) | (.662) | (.423) | (.938) | (.491) | (.471) | (.081) | (.207) |
| Wyoming | -.626 | .965 | .144 | -.361 | .526 | -.498 | .672 | .920 | 1.028 | -.499 |
|  | .623 | .678 | .627 | .644 | .696 | .681 | .675 | .782 | .706 | .840 |
|  | [-1.847, .594] | [-.365, 2.294] | [-1.085, 1.373] | [-1.624, .903] | [-.837, 1.890] | [-1.832, .836] | [-.651, 1.996] | [-.613, 2.453] | [-.355, 2.411] | [-2.145, 1.147] |
|  | (.314) | (.155) | (.818) | (.576) | (.449) | (.464) | (.319) | (.240) | (.145) | (.552) |
| N | 2967 | 2973 | 2969 | 2970 | 2976 | 2973 | 2972 | 2967 | 2970 | 2975 |
| Pseudo-R^2^ |  |  |  |  |  |  |  |  |  |  |
| Model χ^2^ | 383.8 | 433.5 | 167.7 | 47.4 | 372.4 | 379.1 | 654.4 | 493.1 | 13.4 | 332.4 |
| d.f. | 88 | 88 | 88 | 88 | 88 | 88 | 88 | 88 | 88 | 88 |
| p-value | <.001 | <.001 | <.001 | <.001 | <.001 | <.001 | <.001 | <.001 | <.001 | <.001 |

S18 Table. Regression Results, Public Policies, Multilevel Models, Controlling for Positive Diagnoses.

|  | (1) | (2) | (3) | (4) | (5) | (6) | (7) | (8) | (9) | (10) |
| --- | --- | --- | --- | --- | --- | --- | --- | --- | --- | --- |
|  | increasetrade | importtaxes | lowinterest | notreliant | noairtravel | banentry | entrymexico | quarantine | rescueUS | delayelections |
| main |  |  |  |  |  |  |  |  |  |  |
| Democrat | .628 | -.727 | -.254 | -.706 | -.362 | -.657 | -.993 | -.511 | .103 | -.327 |
|  | .050 | .054 | .050 | .051 | .057 | .055 | .054 | .063 | .056 | .068 |
|  | [.531, .726] | [-.832, -.622] | [-.352, -.156] | [-.805, -.606] | [-.473, -.251] | [-.766, -.549] | [-1.100, -.887] | [-.633, -.388] | [-.008, .213] | [-.460, -.193] |
|  | (<.001) | (<.001) | (<.001) | (<.001) | (<.001) | (<.001) | (<.001) | (<.001) | (.068) | (<.001) |
| Other | .116 | -.427 | -.288 | -.339 | -.250 | -.473 | -.532 | -.451 | -.071 | -.205 |
|  | .050 | .054 | .051 | .052 | .057 | .056 | .055 | .063 | .057 | .069 |
|  | [.017, .215] | [-.533, -.320] | [-.387, -.189] | [-.440, -.238] | [-.363, -.138] | [-.583, -.363] | [-.640, -.424] | [-.575, -.327] | [-.183, .041] | [-.340, -.070] |
|  | (.022) | (<.001) | (<.001) | (<.001) | (<.001) | (<.001) | (<.001) | (<.001) | (.213) | (.003) |
| Local COVID-19 Cases | <.001 | <.001 | <.001 | -<.001 | <.001 | <.001 | <.001 | <.001 | <.001 | <.001 |
|  | <.001 | <.001 | <.001 | <.001 | <.001 | <.001 | <.001 | <.001 | <.001 | <.001 |
|  | [-<.001, .000] | [-<.001, .000] | [-<.001, .000] | [-<.001, .000] | [-<.001, .000] | [-<.001, .000] | [-<.001, .000] | [-<.001, .000] | [-<.001, .000] | [-<.001, .000] |
|  | (.128) | (.207) | (.278) | (.898) | (.190) | (.438) | (.554) | (.434) | (.651) | (.770) |
| 35- | -.087 | .172 | .170 | .304 | .208 | .146 | .206 | .130 | .040 | -.118 |
|  | .056 | .060 | .056 | .057 | .064 | .062 | .061 | .070 | .063 | .076 |
|  | [-.197, .022] | [.054, .290] | [.061, .280] | [.192, .416] | [.084, .333] | [.025, .268] | [.087, .326] | [-.007, .268] | [-.083, .164] | [-.267, .032] |
|  | (.118) | (.004) | (.002) | (<.001) | (.001) | (.018) | (.001) | (.063) | (.522) | (.124) |
| 50- | -.008 | .129 | .209 | .450 | .179 | .286 | .399 | -.013 | .119 | -.385 |
|  | .053 | .057 | .053 | .054 | .060 | .059 | .058 | .066 | .060 | .072 |
|  | [-.111, .096] | [.017, .240] | [.105, .313] | [.345, .556] | [.061, .296] | [.171, .402] | [.286, .512] | [-.143, .117] | [.002, .236] | [-.526, -.243] |
|  | (.882) | (.023) | (<.001) | (<.001) | (.003) | (<.001) | (<.001) | (.846) | (.046) | (<.001) |
| 70- | .058 | .030 | .229 | .429 | .118 | .219 | .421 | -.198 | .279 | -.268 |
|  | .067 | .072 | .067 | .068 | .076 | .074 | .073 | .084 | .076 | .091 |
|  | [-.073, .188] | [-.111, .171] | [.098, .360] | [.295, .562] | [-.031, .267] | [.073, .364] | [.279, .564] | [-.362, -.033] | [.131, .427] | [-.447, -.089] |
|  | (.388) | (.675) | (.001) | (<.001) | (.120) | (.003) | (<.001) | (.018) | (<.001) | (.003) |
| gender | -.137 | .161 | .097 | -.064 | .257 | .114 | .011 | .106 | -.063 | .432 |
|  | .038 | .041 | .038 | .039 | .044 | .043 | .042 | .048 | .043 | .053 |
|  | [-.212, -.061] | [.080, .242] | [.022, .173] | [-.141, .013] | [.171, .343] | [.030, .198] | [-.071, .093] | [.012, .201] | [-.148, .022] | [.329, .535] |
|  | (<.001) | (<.001) | (.011) | (.103) | (<.001) | (.008) | (.790) | (.028) | (.149) | (<.001) |
| white | -.021 | -.096 | -.074 | -.101 | -.114 | -.032 | -.088 | -.283 | .049 | -.104 |
|  | .046 | .050 | .046 | .047 | .052 | .051 | .050 | .058 | .052 | .063 |
|  | [-.111, .069] | [-.193, .001] | [-.164, .017] | [-.193, -.009] | [-.216, -.011] | [-.132, .068] | [-.186, .011] | [-.396, -.169] | [-.053, .151] | [-.227, .019] |
|  | (.648) | (.052) | (.110) | (.031) | (.030) | (.530) | (.080) | (<.001) | (.343) | (.098) |
| married | -.034 | .125 | -.015 | <.001 | .073 | .018 | .006 | .068 | -.063 | -.060 |
|  | .042 | .045 | .042 | .043 | .048 | .047 | .046 | .053 | .048 | .058 |
|  | [-.117, .049] | [.036, .214] | [-.098, .067] | [-.084, .085] | [-.021, .167] | [-.075, .110] | [-.084, .096] | [-.035, .172] | [-.157, .030] | [-.173, .053] |
|  | (.419) | (.006) | (.715) | (.999) | (.128) | (.708) | (.897) | (.197) | (.185) | (.300) |
| High school graduate | -.331 | -.107 | -.041 | -.105 | .014 | -.317 | -.180 | -.454 | .058 | .005 |
|  | .113 | .121 | .113 | .115 | .128 | .126 | .123 | .141 | .128 | .155 |
|  | [-.553, -.110] | [-.345, .130] | [-.263, .181] | [-.330, .120] | [-.236, .265] | [-.563, -.070] | [-.420, .061] | [-.731, -.177] | [-.193, .310] | [-.300, .309] |
|  | (.003) | (.377) | (.716) | (.361) | (.910) | (.012) | (.143) | (.001) | (.650) | (.975) |
| Some college | -.301 | -.271 | -.086 | -.119 | -.225 | -.489 | -.336 | -.901 | .116 | -.205 |
|  | .115 | .124 | .115 | .117 | .130 | .128 | .125 | .144 | .131 | .158 |
|  | [-.527, -.075] | [-.513, -.028] | [-.312, .140] | [-.349, .110] | [-.481, .031] | [-.740, -.238] | [-.581, -.090] | [-1.183, -.618] | [-.140, .372] | [-.515, .105] |
|  | (.009) | (.029) | (.458) | (.308) | (.085) | (<.001) | (.007) | (<.001) | (.374) | (.195) |
| 2-year | -.258 | -.122 | -.061 | -.059 | -.125 | -.330 | -.245 | -.842 | .016 | -.093 |
|  | .121 | .130 | .121 | .123 | .137 | .135 | .132 | .151 | .137 | .166 |
|  | [-.495, -.021] | [-.377, .132] | [-.298, .176] | [-.300, .182] | [-.394, .144] | [-.594, -.066] | [-.503, .013] | [-1.139, -.545] | [-.253, .285] | [-.419, .232] |
|  | (.033) | (.347) | (.614) | (.632) | (.362) | (.014) | (.063) | (<.001) | (.909) | (.574) |
| 4-year | -.112 | -.413 | -.196 | -.303 | -.393 | -.684 | -.448 | -.966 | .057 | -.211 |
|  | .117 | .126 | .118 | .120 | .133 | .131 | .128 | .147 | .133 | .161 |
|  | [-.342, .118] | [-.661, -.166] | [-.426, .035] | [-.537, -.068] | [-.654, -.132] | [-.940, -.428] | [-.699, -.198] | [-1.255, -.678] | [-.204, .318] | [-.527, .106] |
|  | (.341) | (.001) | (.096) | (.011) | (.003) | (<.001) | (<.001) | (<.001) | (.668) | (.192) |
| Post-grad | -.048 | -.499 | -.219 | -.379 | -.585 | -.668 | -.581 | -1.137 | .012 | -.419 |
|  | .123 | .132 | .123 | .126 | .140 | .137 | .134 | .154 | .140 | .170 |
|  | [-.289, .194] | [-.759, -.240] | [-.461, .023] | [-.625, -.133] | [-.859, -.311] | [-.937, -.399] | [-.844, -.318] | [-1.440, -.835] | [-.262, .286] | [-.751, -.087] |
|  | (.700) | (<.001) | (.076) | (.003) | (<.001) | (<.001) | (<.001) | (<.001) | (.933) | (.013) |
| income=2 | -.186 | .139 | .143 | .004 | .153 | .159 | .175 | -.011 | .026 | .164 |
|  | .109 | .117 | .109 | .111 | .123 | .121 | .119 | .136 | .123 | .149 |
|  | [-.399, .027] | [-.090, .369] | [-.072, .357] | [-.214, .222] | [-.089, .395] | [-.078, .397] | [-.057, .408] | [-.279, .257] | [-.215, .267] | [-.128, .456] |
|  | (.088) | (.234) | (.192) | (.970) | (.216) | (.189) | (.140) | (.936) | (.834) | (.272) |
| income=3 | -.079 | .102 | .141 | .125 | .090 | .155 | .237 | -.231 | .144 | .209 |
|  | .105 | .113 | .106 | .108 | .120 | .117 | .115 | .132 | .119 | .144 |
|  | [-.285, .127] | [-.120, .324] | [-.067, .348] | [-.086, .336] | [-.145, .324] | [-.075, .385] | [.011, .462] | [-.490, .028] | [-.090, .377] | [-.074, .492] |
|  | (.451) | (.369) | (.184) | (.245) | (.453) | (.187) | (.040) | (.080) | (.228) | (.148) |
| income=4 | -.065 | .075 | .228 | .067 | .062 | -.015 | .133 | -.257 | .014 | .119 |
|  | .108 | .116 | .108 | .110 | .123 | .120 | .118 | .136 | .122 | .148 |
|  | [-.276, .147] | [-.153, .303] | [.015, .440] | [-.149, .283] | [-.178, .302] | [-.251, .221] | [-.098, .364] | [-.523, .009] | [-.225, .253] | [-.170, .409] |
|  | (.549) | (.521) | (.036) | (.545) | (.613) | (.902) | (.258) | (.058) | (.906) | (.420) |
| income=5 | -.071 | .012 | .107 | .188 | .050 | .091 | .240 | -.427 | .132 | .026 |
|  | .110 | .119 | .111 | .113 | .125 | .123 | .120 | .138 | .124 | .151 |
|  | [-.286, .145] | [-.221, .245] | [-.110, .325] | [-.033, .409] | [-.195, .296] | [-.150, .331] | [.004, .476] | [-.698, -.155] | [-.112, .376] | [-.270, .321] |
|  | (.520) | (.918) | (.332) | (.096) | (.688) | (.459) | (.046) | (.002) | (.289) | (.865) |
| income=6 | -.084 | -.018 | .135 | .251 | .110 | .145 | .258 | -.302 | .088 | .191 |
|  | .111 | .120 | .112 | .114 | .126 | .124 | .122 | .140 | .126 | .153 |
|  | [-.301, .134] | [-.253, .217] | [-.084, .354] | [.029, .474] | [-.138, .358] | [-.098, .388] | [.020, .497] | [-.576, -.028] | [-.158, .334] | [-.108, .490] |
|  | (.451) | (.882) | (.228) | (.027) | (.383) | (.244) | (.034) | (.031) | (.483) | (.211) |
| income=7 | -.134 | -.021 | .155 | .134 | .234 | .279 | .146 | -.304 | -.075 | .056 |
|  | .119 | .128 | .120 | .122 | .135 | .133 | .131 | .150 | .135 | .163 |
|  | [-.368, .099] | [-.273, .231] | [-.080, .390] | [-.105, .373] | [-.031, .499] | [.018, .539] | [-.110, .402] | [-.598, -.011] | [-.339, .189] | [-.264, .376] |
|  | (.260) | (.869) | (.195) | (.273) | (.084) | (.036) | (.264) | (.042) | (.577) | (.732) |
| income=8 | -.192 | -.005 | .201 | .140 | .021 | .045 | .198 | -.359 | .005 | .017 |
|  | .116 | .125 | .117 | .118 | .132 | .129 | .127 | .146 | .131 | .159 |
|  | [-.420, .035] | [-.250, .240] | [-.028, .429] | [-.093, .372] | [-.238, .279] | [-.208, .299] | [-.051, .446] | [-.644, -.073] | [-.252, .262] | [-.294, .329] |
|  | (.098) | (.970) | (.085) | (.238) | (.875) | (.728) | (.119) | (.014) | (.969) | (.913) |
| income=9 | .012 | -.166 | .162 | .064 | -.187 | .093 | .063 | -.481 | .010 | -.177 |
|  | .115 | .124 | .115 | .117 | .130 | .128 | .125 | .144 | .130 | .157 |
|  | [-.213, .236] | [-.408, .077] | [-.064, .388] | [-.166, .294] | [-.443, .068] | [-.157, .344] | [-.183, .309] | [-.763, -.198] | [-.244, .264] | [-.485, .132] |
|  | (.920) | (.180) | (.160) | (.586) | (.151) | (.465) | (.615) | (.001) | (.939) | (.262) |
| income=10 | .006 | -.175 | .163 | .013 | -.029 | -.024 | .148 | -.512 | .028 | -.189 |
|  | .119 | .129 | .120 | .122 | .136 | .133 | .130 | .150 | .135 | .163 |
|  | [-.228, .239] | [-.427, .077] | [-.072, .398] | [-.226, .252] | [-.295, .236] | [-.285, .236] | [-.107, .404] | [-.806, -.219] | [-.236, .292] | [-.510, .131] |
|  | (.961) | (.174) | (.174) | (.914) | (.828) | (.854) | (.255) | (.001) | (.837) | (.247) |
| income=11 | -.023 | -.211 | .177 | -.088 | -.175 | -.002 | .053 | -.633 | -.029 | .041 |
|  | .122 | .132 | .123 | .125 | .139 | .137 | .134 | .154 | .138 | .168 |
|  | [-.263, .216] | [-.470, .048] | [-.064, .418] | [-.333, .158] | [-.448, .098] | [-.270, .266] | [-.209, .316] | [-.934, -.331] | [-.301, .242] | [-.288, .370] |
|  | (.848) | (.110) | (.151) | (.484) | (.210) | (.987) | (.690) | (<.001) | (.833) | (.806) |
| income=12 | -.062 | -.145 | .126 | -.098 | -.104 | .027 | .221 | -.657 | .003 | -.191 |
|  | .135 | .146 | .135 | .138 | .153 | .150 | .147 | .170 | .152 | .185 |
|  | [-.327, .203] | [-.431, .141] | [-.140, .392] | [-.368, .172] | [-.404, .195] | [-.267, .322] | [-.067, .509] | [-.990, -.324] | [-.295, .301] | [-.553, .171] |
|  | (.645) | (.319) | (.352) | (.478) | (.495) | (.856) | (.133) | (<.001) | (.984) | (.301) |
| income=13 | .104 | -.374 | -.161 | -.188 | -.282 | .072 | .039 | -.811 | -.175 | -.260 |
|  | .188 | .203 | .189 | .190 | .214 | .207 | .203 | .234 | .213 | .255 |
|  | [-.265, .473] | [-.772, .024] | [-.531, .209] | [-.561, .185] | [-.702, .137] | [-.334, .478] | [-.359, .438] | [-1.270, -.353] | [-.592, .242] | [-.759, .240] |
|  | (.581) | (.066) | (.394) | (.323) | (.187) | (.728) | (.846) | (.001) | (.411) | (.308) |
| income=14 | -.124 | -.148 | .138 | .049 | -.124 | .152 | .215 | -.697 | -.319 | .039 |
|  | .197 | .212 | .197 | .201 | .224 | .219 | .215 | .247 | .222 | .270 |
|  | [-.510, .262] | [-.564, .268] | [-.249, .525] | [-.346, .443] | [-.563, .316] | [-.278, .582] | [-.207, .636] | [-1.182, -.212] | [-.755, .117] | [-.490, .568] |
|  | (.528) | (.486) | (.483) | (.808) | (.581) | (.487) | (.318) | (.005) | (.151) | (.885) |
| income=15 | .257 | -.165 | -.359 | .440 | .745 | .733 | -.373 | -.173 | 1.024 | .518 |
|  | .596 | .641 | .596 | .608 | .679 | .664 | .651 | .749 | .673 | .817 |
|  | [-.911, 1.425] | [-1.422, 1.091] | [-1.528, .809] | [-.751, 1.631] | [-.586, 2.076] | [-.568, 2.035] | [-1.648, .902] | [-1.640, 1.295] | [-.294, 2.343] | [-1.082, 2.119] |
|  | (.666) | (.796) | (.547) | (.469) | (.273) | (.269) | (.567) | (.818) | (.128) | (.525) |
| income=16 | -.180 | .039 | .381 | -.047 | .073 | .462 | .579 | -.660 | -.158 | .067 |
|  | .280 | .302 | .281 | .286 | .319 | .312 | .306 | .352 | .316 | .384 |
|  | [-.729, .369] | [-.552, .631] | [-.169, .931] | [-.608, .514] | [-.553, .698] | [-.150, 1.073] | [-.020, 1.179] | [-1.349, .030] | [-.778, .462] | [-.685, .820] |
|  | (.520) | (.896) | (.174) | (.870) | (.820) | (.139) | (.058) | (.061) | (.618) | (.861) |
| income=17 | -.241 | .022 | -.011 | .106 | -.061 | .109 | .194 | -.339 | -.061 | -.043 |
|  | .102 | .110 | .103 | .104 | .116 | .114 | .112 | .128 | .116 | .140 |
|  | [-.442, -.041] | [-.194, .238] | [-.213, .191] | [-.098, .311] | [-.288, .166] | [-.114, .332] | [-.025, .413] | [-.590, -.088] | [-.287, .166] | [-.317, .232] |
|  | (.018) | (.841) | (.915) | (.309) | (.599) | (.338) | (.082) | (.008) | (.598) | (.760) |
| Rural Code=2 | -.092 | -.023 | -.046 | .069 | -.026 | -.024 | -.068 | -.011 | -.104 | -.150 |
|  | .053 | .057 | .053 | .054 | .060 | .058 | .058 | .066 | .060 | .072 |
|  | [-.196, .012] | [-.135, .089] | [-.150, .058] | [-.038, .175] | [-.143, .091] | [-.138, .091] | [-.181, .045] | [-.141, .120] | [-.222, .013] | [-.291, -.009] |
|  | (.082) | (.690) | (.389) | (.206) | (.661) | (.685) | (.235) | (.872) | (.081) | (.037) |
| Rural Code=3 | -.065 | -.020 | .004 | .046 | .037 | .098 | .020 | .071 | -.123 | .012 |
|  | .071 | .077 | .071 | .073 | .080 | .079 | .078 | .089 | .081 | .097 |
|  | [-.205, .074] | [-.171, .131] | [-.136, .144] | [-.098, .189] | [-.121, .194] | [-.056, .252] | [-.133, .172] | [-.104, .246] | [-.282, .035] | [-.178, .201] |
|  | (.359) | (.792) | (.956) | (.531) | (.647) | (.212) | (.801) | (.426) | (.127) | (.904) |
| Rural Code=4 | -.190 | .071 | .091 | .124 | .083 | .063 | .269 | .168 | .070 | -.189 |
|  | .098 | .106 | .098 | .100 | .111 | .109 | .107 | .123 | .111 | .134 |
|  | [-.382, .002] | [-.136, .279] | [-.101, .284] | [-.072, .321] | [-.135, .300] | [-.150, .276] | [.060, .478] | [-.073, .409] | [-.147, .287] | [-.451, .073] |
|  | (.052) | (.501) | (.354) | (.215) | (.455) | (.561) | (.012) | (.173) | (.528) | (.157) |
| Rural Code=5 | -.055 | -.039 | -.132 | -.116 | .204 | -<.001 | .030 | -.027 | -.327 | .145 |
|  | .150 | .161 | .150 | .156 | .169 | .166 | .163 | .188 | .170 | .204 |
|  | [-.349, .240] | [-.356, .277] | [-.426, .161] | [-.421, .189] | [-.128, .536] | [-.325, .324] | [-.289, .349] | [-.394, .341] | [-.661, .007] | [-.254, .544] |
|  | (.715) | (.807) | (.378) | (.457) | (.229) | (.999) | (.854) | (.886) | (.055) | (.476) |
| Rural Code=6 | -.105 | .130 | .090 | .095 | .138 | .099 | .096 | .222 | -.059 | -.011 |
|  | .098 | .105 | .098 | .100 | .110 | .108 | .106 | .122 | .110 | .133 |
|  | [-.297, .086] | [-.076, .337] | [-.101, .282] | [-.101, .291] | [-.078, .354] | [-.112, .311] | [-.112, .304] | [-.018, .462] | [-.275, .157] | [-.271, .249] |
|  | (.280) | (.216) | (.357) | (.340) | (.211) | (.357) | (.365) | (.070) | (.591) | (.934) |
| Rural Code=7 | -.041 | .007 | -.207 | .073 | .029 | .266 | -.011 | .027 | .072 | -.131 |
|  | .133 | .144 | .134 | .137 | .151 | .148 | .145 | .169 | .151 | .182 |
|  | [-.303, .220] | [-.276, .289] | [-.469, .055] | [-.195, .341] | [-.267, .326] | [-.023, .556] | [-.296, .274] | [-.303, .358] | [-.223, .368] | [-.488, .226] |
|  | (.757) | (.962) | (.121) | (.595) | (.846) | (.072) | (.939) | (.871) | (.632) | (.471) |
| Rural Code=8 | -.518 | -.012 | -.047 | .282 | .209 | -.033 | .277 | -.042 | .383 | .049 |
|  | .249 | .268 | .249 | .254 | .283 | .277 | .271 | .312 | .281 | .340 |
|  | [-1.006, -.031] | [-.537, .514] | [-.535, .441] | [-.216, .781] | [-.346, .763] | [-.575, .510] | [-.255, .808] | [-.654, .570] | [-.168, .933] | [-.617, .716] |
|  | (.037) | (.965) | (.850) | (.267) | (.461) | (.906) | (.307) | (.894) | (.173) | (.885) |
| Rural Code=9 | -.048 | -.138 | -.110 | -.143 | -.082 | -.073 | -.432 | -.238 | -.372 | .239 |
|  | .211 | .228 | .212 | .216 | .240 | .235 | .230 | .265 | .239 | .289 |
|  | [-.462, .366] | [-.585, .309] | [-.525, .305] | [-.568, .281] | [-.552, .389] | [-.533, .387] | [-.884, .019] | [-.758, .283] | [-.840, .096] | [-.327, .804] |
|  | (.820) | (.546) | (.602) | (.507) | (.734) | (.755) | (.061) | (.371) | (.119) | (.408) |
| Alaska | -.040 | .750 | .207 | .217 | .057 | -.558 | .014 | -.035 | -.084 | -.339 |
|  | .423 | .455 | .423 | .431 | .480 | .469 | .460 | .531 | .477 | .578 |
|  | [-.869, .789] | [-.142, 1.642] | [-.621, 1.035] | [-.629, 1.062] | [-.884, .998] | [-1.478, .362] | [-.888, .917] | [-1.076, 1.006] | [-1.018, .850] | [-1.472, .795] |
|  | (.925) | (.099) | (.624) | (.616) | (.905) | (.235) | (.975) | (.947) | (.859) | (.558) |
| Arizona | .009 | -.147 | -.436 | -.282 | -.382 | -.060 | -.394 | -.313 | -.082 | -.805 |
|  | .203 | .218 | .203 | .207 | .228 | .223 | .220 | .257 | .229 | .277 |
|  | [-.389, .408] | [-.574, .280] | [-.833, -.039] | [-.687, .124] | [-.830, .065] | [-.498, .378] | [-.824, .037] | [-.816, .190] | [-.529, .366] | [-1.348, -.261] |
|  | (.963) | (.500) | (.031) | (.174) | (.094) | (.788) | (.073) | (.223) | (.721) | (.004) |
| Arkansas | .073 | .228 | -.079 | .084 | -.008 | .172 | .071 | .131 | -.020 | .003 |
|  | .229 | .244 | .227 | .232 | .257 | .251 | .247 | .286 | .256 | .311 |
|  | [-.376, .522] | [-.251, .708] | [-.524, .365] | [-.370, .539] | [-.510, .495] | [-.319, .664] | [-.412, .554] | [-.430, .692] | [-.521, .481] | [-.606, .612] |
|  | (.749) | (.350) | (.727) | (.716) | (.977) | (.492) | (.773) | (.647) | (.938) | (.993) |
| California | -.056 | .011 | -.303 | .024 | -.196 | -.056 | -.108 | -.053 | -.012 | -.479 |
|  | .180 | .192 | .178 | .182 | .202 | .198 | .194 | .226 | .201 | .246 |
|  | [-.409, .297] | [-.366, .388] | [-.653, .047] | [-.334, .381] | [-.592, .200] | [-.443, .332] | [-.489, .273] | [-.496, .390] | [-.406, .382] | [-.960, .003] |
|  | (.756) | (.955) | (.090) | (.896) | (.332) | (.778) | (.578) | (.814) | (.953) | (.051) |
| Colorado | -.377 | .177 | -.142 | -.075 | -.260 | .067 | -.096 | -.283 | -.322 | -.220 |
|  | .229 | .246 | .229 | .234 | .258 | .252 | .248 | .288 | .258 | .312 |
|  | [-.827, .072] | [-.306, .660] | [-.590, .306] | [-.534, .383] | [-.765, .246] | [-.427, .561] | [-.583, .390] | [-.848, .282] | [-.827, .183] | [-.832, .392] |
|  | (.100) | (.474) | (.534) | (.747) | (.314) | (.791) | (.698) | (.326) | (.211) | (.481) |
| Connecticut | .395 | .222 | -.255 | -.194 | -.215 | .350 | .058 | .057 | .176 | -.275 |
|  | .252 | .268 | .251 | .254 | .281 | .277 | .270 | .313 | .283 | .343 |
|  | [-.098, .888] | [-.303, .747] | [-.747, .237] | [-.693, .304] | [-.766, .335] | [-.194, .893] | [-.471, .588] | [-.557, .670] | [-.379, .730] | [-.947, .397] |
|  | (.117) | (.407) | (.309) | (.445) | (.443) | (.207) | (.829) | (.857) | (.535) | (.423) |
| Delaware | .081 | .138 | .059 | -.057 | .034 | -.252 | .164 | -.128 | .242 | -.894 |
|  | .322 | .347 | .322 | .329 | .362 | .354 | .349 | .404 | .363 | .438 |
|  | [-.549, .712] | [-.542, .818] | [-.572, .689] | [-.702, .588] | [-.676, .743] | [-.946, .442] | [-.520, .848] | [-.920, .664] | [-.468, .953] | [-1.751, -.036] |
|  | (.800) | (.691) | (.855) | (.862) | (.926) | (.476) | (.638) | (.752) | (.504) | (.041) |
| District of Columbia | -.239 | .012 | .092 | -.340 | -.412 | .325 | .294 | -.225 | .002 | .480 |
|  | .399 | .429 | .421 | .407 | .453 | .468 | .434 | .501 | .449 | .545 |
|  | [-1.020, .543] | [-.829, .852] | [-.734, .918] | [-1.138, .457] | [-1.299, .475] | [-.593, 1.243] | [-.557, 1.144] | [-1.206, .757] | [-.878, .883] | [-.589, 1.549] |
|  | (.549) | (.978) | (.827) | (.403) | (.363) | (.488) | (.498) | (.653) | (.996) | (.379) |
| Florida | -.067 | .096 | -.128 | -.120 | -.227 | .065 | -.161 | .061 | -.017 | -.523 |
|  | .181 | .194 | .180 | .184 | .203 | .199 | .195 | .228 | .203 | .248 |
|  | [-.422, .288] | [-.283, .475] | [-.480, .224] | [-.479, .240] | [-.626, .171] | [-.325, .455] | [-.544, .222] | [-.385, .508] | [-.415, .380] | [-1.008, -.038] |
|  | (.711) | (.620) | (.476) | (.515) | (.264) | (.745) | (.409) | (.788) | (.932) | (.035) |
| Georgia | -.139 | .125 | -.279 | -.083 | -.399 | .064 | -.106 | .023 | -.182 | -.263 |
|  | .196 | .210 | .194 | .199 | .221 | .215 | .212 | .246 | .220 | .267 |
|  | [-.524, .246] | [-.286, .537] | [-.660, .103] | [-.473, .307] | [-.831, .033] | [-.359, .486] | [-.521, .309] | [-.460, .506] | [-.612, .248] | [-.787, .262] |
|  | (.479) | (.551) | (.152) | (.676) | (.070) | (.768) | (.617) | (.925) | (.407) | (.326) |
| Hawaii | .349 | .062 | .027 | -.504 | -.146 | .477 | -.349 | .110 | -.233 | -.128 |
|  | .346 | .373 | .346 | .355 | .390 | .381 | .375 | .434 | .390 | .470 |
|  | [-.329, 1.027] | [-.670, .794] | [-.652, .705] | [-1.199, .191] | [-.910, .617] | [-.269, 1.224] | [-1.085, .386] | [-.741, .961] | [-.998, .532] | [-1.050, .794] |
|  | (.313) | (.868) | (.939) | (.155) | (.707) | (.210) | (.352) | (.800) | (.551) | (.785) |
| Idaho | -.239 | .003 | -.112 | -.101 | -.512 | -.373 | -.430 | .125 | -.463 | -.033 |
|  | .290 | .313 | .290 | .297 | .326 | .319 | .315 | .365 | .327 | .394 |
|  | [-.807, .330] | [-.611, .617] | [-.681, .457] | [-.683, .482] | [-1.152, .127] | [-.998, .252] | [-1.047, .186] | [-.589, .840] | [-1.104, .178] | [-.806, .740] |
|  | (.411) | (.992) | (.700) | (.735) | (.116) | (.243) | (.171) | (.732) | (.157) | (.934) |
| Illinois | -.249 | .151 | -.267 | .158 | -.161 | .113 | -.002 | .199 | -.196 | -.132 |
|  | .199 | .213 | .198 | .202 | .224 | .219 | .215 | .251 | .223 | .272 |
|  | [-.640, .142] | [-.268, .569] | [-.656, .121] | [-.238, .555] | [-.601, .278] | [-.317, .543] | [-.425, .420] | [-.292, .690] | [-.634, .242] | [-.665, .401] |
|  | (.212) | (.481) | (.177) | (.434) | (.473) | (.607) | (.991) | (.426) | (.381) | (.628) |
| Indiana | -.027 | <.001 | -.059 | .018 | -.183 | .063 | -.098 | -.162 | -.006 | -.199 |
|  | .214 | .228 | .212 | .217 | .240 | .235 | .230 | .267 | .239 | .291 |
|  | [-.445, .392] | [-.447, .448] | [-.475, .356] | [-.408, .444] | [-.653, .288] | [-.397, .523] | [-.550, .354] | [-.686, .362] | [-.474, .462] | [-.768, .371] |
|  | (.901) | (.999) | (.779) | (.934) | (.447) | (.789) | (.671) | (.545) | (.981) | (.495) |
| Iowa | -.123 | .327 | -.274 | .034 | -.501 | .334 | .102 | .114 | -.199 | -.147 |
|  | .252 | .270 | .251 | .256 | .285 | .279 | .274 | .317 | .283 | .345 |
|  | [-.617, .371] | [-.203, .857] | [-.766, .218] | [-.468, .537] | [-1.060, .058] | [-.213, .881] | [-.434, .638] | [-.506, .735] | [-.754, .356] | [-.823, .529] |
|  | (.626) | (.227) | (.275) | (.893) | (.079) | (.231) | (.710) | (.718) | (.482) | (.670) |
| Kansas | -.507 | -.279 | -.222 | .120 | -.074 | .219 | -.104 | -.477 | -.430 | -.714 |
|  | .305 | .328 | .305 | .311 | .345 | .337 | .331 | .383 | .344 | .417 |
|  | [-1.105, .091] | [-.922, .365] | [-.820, .375] | [-.490, .731] | [-.750, .603] | [-.443, .880] | [-.753, .546] | [-1.228, .275] | [-1.104, .243] | [-1.531, .102] |
|  | (.097) | (.396) | (.466) | (.700) | (.831) | (.517) | (.754) | (.214) | (.210) | (.086) |
| Kentucky | -.200 | .369 | -.076 | .149 | -.114 | .238 | .257 | .009 | .153 | -.257 |
|  | .232 | .249 | .231 | .237 | .261 | .255 | .251 | .291 | .260 | .316 |
|  | [-.654, .254] | [-.118, .857] | [-.528, .376] | [-.316, .613] | [-.625, .398] | [-.262, .739] | [-.234, .749] | [-.561, .579] | [-.357, .662] | [-.876, .363] |
|  | (.387) | (.137) | (.742) | (.530) | (.663) | (.350) | (.305) | (.975) | (.557) | (.417) |
| Louisiana | -.188 | .354 | -.212 | -.259 | -.215 | .385 | .038 | .161 | -.078 | -.083 |
|  | .233 | .250 | .232 | .237 | .262 | .256 | .252 | .292 | .261 | .317 |
|  | [-.644, .269] | [-.136, .844] | [-.666, .243] | [-.724, .206] | [-.728, .298] | [-.117, .886] | [-.456, .531] | [-.412, .734] | [-.591, .434] | [-.705, .538] |
|  | (.420) | (.157) | (.362) | (.275) | (.411) | (.133) | (.882) | (.582) | (.764) | (.793) |
| Maine | -.405 | .270 | .158 | .121 | .286 | .306 | .369 | .043 | -.489 | -.531 |
|  | .322 | .347 | .322 | .329 | .363 | .355 | .349 | .404 | .363 | .438 |
|  | [-1.035, .226] | [-.411, .950] | [-.472, .789] | [-.524, .767] | [-.425, .997] | [-.389, 1.001] | [-.315, 1.053] | [-.749, .835] | [-1.200, .222] | [-1.389, .328] |
|  | (.208) | (.437) | (.623) | (.712) | (.430) | (.388) | (.291) | (.915) | (.178) | (.226) |
| Maryland | .134 | -.112 | -.530 | -.314 | -.262 | -.031 | -.178 | -.164 | -.111 | -.259 |
|  | .230 | .247 | .229 | .234 | .261 | .253 | .249 | .289 | .258 | .314 |
|  | [-.317, .585] | [-.595, .372] | [-.979, -.081] | [-.772, .145] | [-.773, .249] | [-.528, .466] | [-.666, .310] | [-.730, .402] | [-.617, .395] | [-.875, .356] |
|  | (.560) | (.651) | (.021) | (.180) | (.315) | (.903) | (.474) | (.570) | (.667) | (.409) |
| Massachusetts | -.126 | -.205 | -.092 | -.221 | -.395 | .063 | -.080 | -.429 | -.002 | -.045 |
|  | .217 | .233 | .216 | .221 | .244 | .239 | .235 | .273 | .244 | .296 |
|  | [-.552, .299] | [-.661, .252] | [-.515, .332] | [-.654, .212] | [-.873, .084] | [-.405, .531] | [-.540, .380] | [-.963, .106] | [-.479, .476] | [-.625, .535] |
|  | (.561) | (.379) | (.671) | (.317) | (.106) | (.791) | (.733) | (.116) | (.995) | (.880) |
| Michigan | -.184 | .177 | -.133 | -.072 | -.277 | .100 | -.001 | -.117 | -.241 | -.456 |
|  | .193 | .207 | .192 | .196 | .217 | .213 | .209 | .243 | .217 | .264 |
|  | [-.563, .195] | [-.229, .582] | [-.509, .243] | [-.457, .312] | [-.704, .149] | [-.317, .517] | [-.410, .409] | [-.593, .359] | [-.665, .184] | [-.973, .062] |
|  | (.342) | (.393) | (.488) | (.712) | (.202) | (.638) | (.998) | (.630) | (.266) | (.084) |
| Minnesota | -.221 | .188 | -.549 | .144 | -.240 | -.066 | -.001 | -.360 | -.362 | -.739 |
|  | .227 | .244 | .226 | .231 | .256 | .251 | .248 | .286 | .257 | .311 |
|  | [-.667, .224] | [-.290, .666] | [-.992, -.105] | [-.310, .597] | [-.743, .262] | [-.557, .425] | [-.487, .484] | [-.920, .200] | [-.864, .141] | [-1.347, -.130] |
|  | (.330) | (.440) | (.015) | (.534) | (.349) | (.793) | (.995) | (.208) | (.159) | (.017) |
| Mississippi | -.078 | .251 | -.594 | .041 | -.323 | -.213 | .204 | -.033 | -.333 | -.083 |
|  | .259 | .276 | .256 | .261 | .290 | .284 | .278 | .322 | .289 | .351 |
|  | [-.586, .429] | [-.290, .791] | [-1.096, -.093] | [-.472, .553] | [-.892, .246] | [-.769, .343] | [-.342, .750] | [-.664, .599] | [-.898, .233] | [-.771, .604] |
|  | (.763) | (.363) | (.020) | (.876) | (.266) | (.452) | (.464) | (.920) | (.249) | (.813) |
| Missouri | -.279 | .358 | -.140 | .126 | -.204 | .329 | .045 | .025 | -.162 | -.055 |
|  | .209 | .224 | .208 | .212 | .236 | .230 | .226 | .262 | .234 | .285 |
|  | [-.688, .130] | [-.080, .796] | [-.547, .266] | [-.290, .542] | [-.666, .257] | [-.121, .779] | [-.398, .487] | [-.489, .539] | [-.620, .297] | [-.613, .504] |
|  | (.181) | (.109) | (.499) | (.552) | (.386) | (.152) | (.843) | (.924) | (.490) | (.848) |
| Montana | .187 | -.162 | -.467 | -.001 | -1.159 | -.795 | -.878 | -.338 | -.556 | -1.258 |
|  | .321 | .346 | .321 | .328 | .362 | .354 | .348 | .403 | .362 | .437 |
|  | [-.442, .816] | [-.840, .516] | [-1.095, .162] | [-.644, .643] | [-1.868, -.449] | [-1.489, -.102] | [-1.561, -.196] | [-1.127, .452] | [-1.265, .153] | [-2.114, -.401] |
|  | (.560) | (.640) | (.146) | (.999) | (.001) | (.025) | (.012) | (.402) | (.124) | (.004) |
| Nebraska | -.270 | .173 | -.049 | .352 | -.205 | -.317 | -.216 | -.219 | -.113 | -.265 |
|  | .339 | .364 | .338 | .335 | .373 | .376 | .358 | .426 | .370 | .463 |
|  | [-.934, .394] | [-.541, .887] | [-.712, .614] | [-.306, 1.009] | [-.937, .526] | [-1.053, .420] | [-.917, .485] | [-1.053, .615] | [-.839, .613] | [-1.173, .644] |
|  | (.425) | (.634) | (.885) | (.294) | (.582) | (.399) | (.546) | (.607) | (.761) | (.568) |
| Nevada | -.108 | -.037 | -.093 | -.055 | -.172 | .048 | -.121 | -.267 | -.323 | -.532 |
|  | .228 | .245 | .227 | .232 | .255 | .250 | .247 | .286 | .256 | .310 |
|  | [-.554, .338] | [-.516, .443] | [-.537, .351] | [-.510, .400] | [-.672, .329] | [-.442, .537] | [-.605, .364] | [-.827, .293] | [-.824, .178] | [-1.139, .075] |
|  | (.634) | (.880) | (.682) | (.812) | (.502) | (.848) | (.626) | (.350) | (.206) | (.086) |
| New Hampshire | -.050 | -.084 | -.347 | .137 | -.173 | .094 | .109 | .078 | .449 | -.728 |
|  | .272 | .293 | .272 | .278 | .307 | .300 | .295 | .342 | .307 | .371 |
|  | [-.584, .484] | [-.659, .491] | [-.880, .186] | [-.409, .683] | [-.774, .428] | [-.494, .681] | [-.470, .687] | [-.593, .748] | [-.152, 1.050] | [-1.455, -.002] |
|  | (.855) | (.775) | (.202) | (.623) | (.572) | (.755) | (.713) | (.820) | (.143) | (.049) |
| New Jersey | -.251 | -.112 | -.073 | -.241 | -.076 | .023 | -.121 | -.104 | .012 | -.349 |
|  | .210 | .226 | .209 | .214 | .238 | .232 | .228 | .265 | .236 | .288 |
|  | [-.663, .162] | [-.554, .330] | [-.483, .336] | [-.661, .178] | [-.542, .390] | [-.433, .478] | [-.568, .326] | [-.623, .416] | [-.451, .475] | [-.914, .216] |
|  | (.233) | (.619) | (.725) | (.259) | (.748) | (.922) | (.595) | (.696) | (.961) | (.226) |
| New Mexico | -.197 | .603 | -.050 | .119 | -.039 | .187 | .191 | .140 | -.130 | -.359 |
|  | .251 | .270 | .250 | .256 | .282 | .276 | .272 | .315 | .285 | .342 |
|  | [-.688, .295] | [.074, 1.131] | [-.540, .440] | [-.382, .620] | [-.593, .515] | [-.354, .728] | [-.341, .724] | [-.477, .757] | [-.688, .428] | [-1.029, .311] |
|  | (.433) | (.025) | (.842) | (.642) | (.890) | (.498) | (.481) | (.656) | (.649) | (.293) |
| New York | -.170 | .262 | -.267 | .068 | -.074 | .188 | .116 | .153 | -.021 | -.242 |
|  | .185 | .198 | .183 | .187 | .208 | .203 | .200 | .232 | .207 | .253 |
|  | [-.532, .193] | [-.126, .649] | [-.626, .093] | [-.299, .435] | [-.481, .334] | [-.210, .587] | [-.275, .507] | [-.303, .609] | [-.427, .384] | [-.737, .254] |
|  | (.359) | (.185) | (.146) | (.717) | (.722) | (.354) | (.561) | (.511) | (.919) | (.339) |
| North Carolina | -.073 | .068 | -.254 | -.142 | .186 | .211 | .046 | .073 | -.209 | -.225 |
|  | .199 | .213 | .198 | .202 | .224 | .219 | .215 | .250 | .223 | .272 |
|  | [-.463, .317] | [-.349, .485] | [-.642, .133] | [-.538, .254] | [-.253, .624] | [-.217, .640] | [-.376, .467] | [-.417, .563] | [-.646, .227] | [-.758, .307] |
|  | (.713) | (.750) | (.198) | (.481) | (.406) | (.334) | (.832) | (.771) | (.347) | (.407) |
| North Dakota | .272 | -.097 | -.595 | -.515 | -.600 | -.753 | -.538 | -.113 | -.168 | -1.479 |
|  | .402 | .435 | .403 | .412 | .454 | .444 | .437 | .505 | .454 | .548 |
|  | [-.516, 1.061] | [-.949, .754] | [-1.385, .195] | [-1.323, .293] | [-1.490, .290] | [-1.624, .117] | [-1.395, .319] | [-1.103, .878] | [-1.058, .722] | [-2.553, -.405] |
|  | (.498) | (.823) | (.140) | (.212) | (.187) | (.090) | (.218) | (.824) | (.712) | (.007) |
| Ohio | -.086 | .254 | -.259 | .091 | -.087 | .186 | -.017 | -.243 | -.227 | .074 |
|  | .194 | .207 | .192 | .196 | .218 | .213 | .209 | .243 | .217 | .264 |
|  | [-.466, .294] | [-.151, .660] | [-.636, .118] | [-.294, .476] | [-.514, .339] | [-.231, .603] | [-.427, .393] | [-.719, .234] | [-.652, .197] | [-.444, .591] |
|  | (.658) | (.219) | (.178) | (.643) | (.688) | (.381) | (.936) | (.318) | (.294) | (.780) |
| Oklahoma | -.283 | .038 | -.658 | -.162 | .006 | -.275 | -.255 | .034 | -.250 | -.640 |
|  | .258 | .277 | .257 | .263 | .292 | .285 | .280 | .324 | .290 | .353 |
|  | [-.789, .223] | [-.506, .581] | [-1.163, -.154] | [-.678, .353] | [-.566, .577] | [-.834, .284] | [-.804, .294] | [-.601, .670] | [-.819, .319] | [-1.331, .052] |
|  | (.273) | (.892) | (.011) | (.537) | (.985) | (.335) | (.362) | (.916) | (.389) | (.070) |
| Oregon | -.249 | -.001 | -.296 | .053 | -.822 | -.071 | -.407 | -.572 | .296 | -.418 |
|  | .215 | .230 | .214 | .219 | .241 | .235 | .232 | .269 | .241 | .292 |
|  | [-.669, .172] | [-.452, .450] | [-.714, .123] | [-.376, .481] | [-1.294, -.351] | [-.532, .391] | [-.861, .047] | [-1.100, -.044] | [-.176, .767] | [-.990, .155] |
|  | (.246) | (.997) | (.166) | (.810) | (.001) | (.764) | (.079) | (.034) | (.219) | (.153) |
| Pennsylvania | -.142 | .093 | -.174 | .097 | -.019 | .134 | .145 | -.194 | -.121 | -.077 |
|  | .184 | .196 | .182 | .186 | .206 | .202 | .198 | .230 | .205 | .251 |
|  | [-.502, .218] | [-.291, .477] | [-.531, .183] | [-.267, .462] | [-.423, .385] | [-.261, .529] | [-.243, .533] | [-.645, .258] | [-.523, .282] | [-.568, .414] |
|  | (.440) | (.635) | (.339) | (.601) | (.927) | (.507) | (.465) | (.401) | (.557) | (.759) |
| Rhode Island | -.073 | .888 | .033 | -.033 | .126 | .233 | .268 | .492 | -.254 | -.179 |
|  | .421 | .428 | .398 | .406 | .452 | .442 | .433 | .500 | .449 | .544 |
|  | [-.898, .752] | [.049, 1.727] | [-.747, .812] | [-.829, .762] | [-.759, 1.012] | [-.633, 1.099] | [-.581, 1.117] | [-.488, 1.472] | [-1.133, .626] | [-1.246, .888] |
|  | (.862) | (.038) | (.934) | (.934) | (.780) | (.598) | (.536) | (.325) | (.572) | (.742) |
| South Carolina | -.140 | -.094 | -.151 | -.003 | -.068 | .218 | .078 | -.382 | -.114 | -.200 |
|  | .224 | .241 | .224 | .229 | .252 | .247 | .243 | .282 | .252 | .306 |
|  | [-.580, .300] | [-.566, .379] | [-.589, .288] | [-.452, .446] | [-.563, .426] | [-.265, .702] | [-.398, .554] | [-.935, .171] | [-.608, .380] | [-.799, .400] |
|  | (.533) | (.698) | (.500) | (.989) | (.786) | (.377) | (.749) | (.176) | (.652) | (.514) |
| South Dakota | -.039 | .270 | -.577 | .069 | -.131 | .105 | -.217 | -.702 | -.218 | -.180 |
|  | .370 | .399 | .370 | .378 | .418 | .409 | .402 | .464 | .417 | .504 |
|  | [-.764, .685] | [-.511, 1.052] | [-1.301, .148] | [-.672, .811] | [-.950, .688] | [-.695, .906] | [-1.005, .570] | [-1.612, .208] | [-1.035, .599] | [-1.168, .808] |
|  | (.915) | (.498) | (.119) | (.854) | (.754) | (.797) | (.589) | (.131) | (.601) | (.721) |
| Tennessee | -.173 | -.149 | -.307 | -.240 | -.486 | -.130 | -.105 | .033 | -.006 | -.493 |
|  | .219 | .235 | .218 | .223 | .247 | .241 | .237 | .275 | .246 | .299 |
|  | [-.602, .257] | [-.610, .312] | [-.735, .120] | [-.678, .197] | [-.969, -.002] | [-.603, .343] | [-.570, .359] | [-.507, .573] | [-.488, .476] | [-1.079, .094] |
|  | (.431) | (.525) | (.159) | (.281) | (.049) | (.590) | (.657) | (.904) | (.981) | (.100) |
| Texas | -.119 | .077 | -.346 | -.094 | -.225 | .024 | -.012 | -.058 | -.181 | -.490 |
|  | .184 | .196 | .182 | .186 | .206 | .202 | .198 | .231 | .205 | .251 |
|  | [-.479, .241] | [-.307, .461] | [-.703, .011] | [-.459, .270] | [-.629, .179] | [-.372, .419] | [-.400, .376] | [-.510, .394] | [-.583, .221] | [-.981, .001] |
|  | (.516) | (.695) | (.058) | (.613) | (.276) | (.906) | (.953) | (.803) | (.377) | (.051) |
| Utah | -.017 | -.263 | -.508 | -.349 | -.321 | -.084 | -.026 | -.598 | .034 | -1.002 |
|  | .254 | .274 | .254 | .260 | .286 | .279 | .275 | .319 | .286 | .346 |
|  | [-.516, .481] | [-.799, .273] | [-1.005, -.010] | [-.858, .160] | [-.881, .239] | [-.632, .463] | [-.566, .513] | [-1.224, .028] | [-.526, .595] | [-1.680, -.324] |
|  | (.945) | (.337) | (.045) | (.179) | (.261) | (.763) | (.923) | (.061) | (.905) | (.004) |
| Vermont | .292 | -.065 | -.084 | -.028 | -.333 | .264 | -.281 | -.811 | -.428 | -1.393 |
|  | .364 | .392 | .364 | .372 | .413 | .404 | .396 | .458 | .411 | .498 |
|  | [-.422, 1.006] | [-.833, .703] | [-.797, .630] | [-.756, .701] | [-1.143, .478] | [-.529, 1.056] | [-1.059, .496] | [-1.708, .086] | [-1.232, .377] | [-2.369, -.416] |
|  | (.423) | (.869) | (.818) | (.940) | (.421) | (.514) | (.478) | (.076) | (.298) | (.005) |
| Virginia | -.056 | .119 | -.221 | -.073 | -.293 | .128 | -.073 | -.236 | .058 | -.530 |
|  | .197 | .211 | .196 | .200 | .221 | .216 | .213 | .248 | .221 | .268 |
|  | [-.443, .330] | [-.294, .533] | [-.605, .162] | [-.465, .319] | [-.727, .140] | [-.296, .552] | [-.490, .345] | [-.722, .249] | [-.375, .490] | [-1.056, -.004] |
|  | (.774) | (.572) | (.258) | (.715) | (.184) | (.555) | (.733) | (.340) | (.793) | (.048) |
| Washington | -.178 | -.151 | -.122 | -.172 | -.741 | -.179 | -.479 | -.338 | -.279 | -.891 |
|  | .206 | .222 | .206 | .210 | .232 | .227 | .223 | .259 | .232 | .281 |
|  | [-.583, .226] | [-.586, .283] | [-.525, .281] | [-.583, .239] | [-1.195, -.287] | [-.623, .266] | [-.916, -.042] | [-.846, .170] | [-.733, .176] | [-1.443, -.340] |
|  | (.388) | (.495) | (.554) | (.413) | (.001) | (.431) | (.032) | (.192) | (.230) | (.002) |
| West Virginia | -.152 | .039 | -.241 | -.418 | .045 | .232 | .127 | .148 | .020 | -.487 |
|  | .264 | .280 | .260 | .266 | .295 | .289 | .283 | .332 | .293 | .357 |
|  | [-.670, .366] | [-.511, .588] | [-.751, .269] | [-.939, .103] | [-.533, .623] | [-.334, .797] | [-.428, .682] | [-.503, .799] | [-.555, .595] | [-1.186, .212] |
|  | (.564) | (.890) | (.355) | (.116) | (.879) | (.422) | (.654) | (.655) | (.945) | (.172) |
| Wisconsin | .074 | -.016 | -.348 | -.095 | -.195 | .017 | -.159 | -.194 | -.418 | -.368 |
|  | .213 | .228 | .212 | .216 | .240 | .234 | .230 | .267 | .240 | .291 |
|  | [-.343, .491] | [-.463, .431] | [-.763, .068] | [-.519, .329] | [-.665, .275] | [-.442, .476] | [-.611, .292] | [-.718, .330] | [-.888, .051] | [-.938, .201] |
|  | (.728) | (.944) | (.101) | (.661) | (.415) | (.942) | (.489) | (.468) | (.081) | (.205) |
| Wyoming | -.629 | .964 | .142 | -.360 | .526 | -.499 | .671 | .919 | 1.028 | -.498 |
|  | .622 | .678 | .627 | .644 | .696 | .681 | .675 | .782 | .706 | .840 |
|  | [-1.849, .591] | [-.366, 2.293] | [-1.086, 1.371] | [-1.623, .904] | [-.838, 1.890] | [-1.833, .835] | [-.652, 1.994] | [-.615, 2.452] | [-.355, 2.411] | [-2.145, 1.148] |
|  | (.312) | (.155) | (.820) | (.577) | (.450) | (.463) | (.320) | (.240) | (.145) | (.553) |
| N | 2967 | 2973 | 2969 | 2970 | 2976 | 2973 | 2972 | 2967 | 2970 | 2975 |
| Pseudo-R^2^ |  |  |  |  |  |  |  |  |  |  |
| Model χ^2^ | 384.5 | 432.1 | 167.8 | 47.3 | 37.1 | 379.1 | 654.5 | 493.0 | 13.4 | 331.8 |
| d.f. | 88 | 88 | 88 | 88 | 88 | 88 | 88 | 88 | 88 | 88 |
| p-value | <.001 | <.001 | <.001 | <.001 | <.001 | <.001 | <.001 | <.001 | <.001 | <.001 |

**S8 Fig. Public Policy, Multilevel Models.** This figure compares key coefficients of interest from S16-S18 Tables.

# Regularized Regression Models

Below, we show the results of our regularized regression analysis using a linear lasso. Each table provides the OLS regression coefficients from post-lasso selected predictors only. Empty cells imply that the lasso did not select that predictor.

S19 Table. Lasso Selected Predictors, Health Behaviors.

|  | washhands | bought  sanitizer | visit  doctor | changed  travel | avoid  contact | avoid  gatherings | soughtinfo | self  quarantine | change  health |
| --- | --- | --- | --- | --- | --- | --- | --- | --- | --- |
| Democrat | .05 | .02 |  | .00 | .06 | .03 | .08 | .05 | .50 |
| 4-year | .00 |  |  |  |  |  |  |  |  |
| white |  | -.02 |  |  |  |  |  |  |  |
| income=13 |  |  | -.03 |  |  |  |  |  |  |
| Rural Code=5 |  |  | -.03 |  |  |  |  |  |  |
| Connecticut |  |  | -.03 |  |  |  |  |  |  |
| Idaho |  |  | -.03 |  |  |  |  |  |  |
| Massachusetts |  |  | -.03 |  |  |  |  |  |  |
| Montana |  |  | -.01 |  |  |  |  |  |  |
| Utah |  |  | -.03 |  |  |  |  |  |  |
| High school graduate |  |  |  | -.06 |  | -.01 | -.09 |  | -.18 |
| Post-grad |  |  |  | .09 | .03 | .01 | .04 |  | .33 |
| income=14 |  |  |  | .04 |  |  |  |  |  |
| Kentucky |  |  |  | -.05 |  |  |  |  |  |
| Delaware |  |  |  |  | .22 |  |  |  |  |
| 70- |  |  |  |  |  | .04 |  |  |  |
| 50- |  |  |  |  |  |  |  | -.01 |  |
| Observations | 2982 | 2982 | 2982 | 2982 | 2982 | 2982 | 2982 | 2982 | 2982 |

S20 Table. Lasso Selected Predictors, Health Attitudes.

|  | number  deaths | spending  increase | notenough  tests | getsick | negative  economy | friendsick | cannot  return | schools  closed | hardget  items |
| --- | --- | --- | --- | --- | --- | --- | --- | --- | --- |
| Democrat | .12 | .20 | .41 | .30 | .13 | .30 | .23 | .09 | .13 |
| 70- | .04 |  |  |  |  |  |  | -.31 | -.11 |
| Other |  |  | .02 |  |  |  |  |  |  |
| white |  |  | .04 |  |  |  |  |  |  |
| High school graduate |  |  | -.07 |  | -.01 |  |  |  |  |
| gender |  |  |  | .00 |  |  |  |  | .02 |
| income=11 |  |  |  |  | .05 |  |  |  |  |
| 35- |  |  |  |  |  |  |  |  | .01 |
| Observations | 2982 | 2980 | 2968 | 2980 | 2980 | 2981 | 2981 | 2981 | 2979 |

S21 Table. Lasso Selected Predictors, Health Policy Preferences.

|  | free  testing | waive  costs | close  schools | cancel  everything | paid  leave | compensate  firms | national  guard | stop  events | travel  ban | stateslead |
| --- | --- | --- | --- | --- | --- | --- | --- | --- | --- | --- |
| Democrat | .27 | .37 | .25 | .24 | .33 |  | .13 | .23 |  |  |
| gender |  |  | .02 | .07 |  | .14 | .02 | .00 | .36 |  |
| 70- |  |  |  | .03 |  |  |  |  |  |  |
| Other |  |  |  |  |  | -.07 | -.07 |  | -.01 | -.01 |
| Rhode Island |  |  |  |  |  | .12 |  |  |  |  |
| High school graduate |  |  |  |  |  |  | .06 | -.03 | .18 |  |
| white |  |  |  |  |  |  |  | .07 | -.16 |  |
| Post-grad |  |  |  |  |  |  |  |  | -.07 |  |
| Observations | 2970 | 2974 | 2967 | 2973 | 2973 | 2973 | 2974 | 2975 | 2970 | 2973 |

S22 Table. Lasso Selected Predictors, Public Policy Preferences.

|  | increase  trade | import  taxes | lowinterest | notreliant | noair  travel | banentry | entry  mexico | quarantine | rescueUS | delay  elections |
| --- | --- | --- | --- | --- | --- | --- | --- | --- | --- | --- |
| Democrat | .43 | -.30 |  | -.35 | -.01 | -.23 | -.59 | -.01 |  |  |
| High school graduate | -.00 | .11 |  | .03 | .21 | .11 | .14 | .31 |  | .02 |
| Other |  | -.00 |  |  |  | -.05 | -.14 |  |  |  |
| gender |  | .01 |  |  | .08 |  |  |  |  | .24 |
| Post-grad |  | -.05 |  |  | -.12 |  |  | -.17 |  | -.07 |
| 50- |  |  |  | .08 |  | .03 | .08 |  |  | -.03 |
| 4-year |  |  |  |  |  | -.03 |  |  |  |  |
| 70- |  |  |  |  |  |  | .04 |  |  |  |
| white |  |  |  |  |  |  |  | -.12 |  |  |
| income=2 |  |  |  |  |  |  |  | .03 |  |  |
| Observations | 2967 | 2973 | 2969 | 2970 | 2976 | 2973 | 2972 | 2967 | 2970 | 2975 |

# Results by News Consumption

We hand-code media sources from open-ended survey responses in which respondents specify their main source of news. These include sources such as Fox News, programs or anchors who appear on Fox News (i.e. “Hannity”), or sources (i.e. “One America News Network”) or programs (“Daily Shoah”). The command that we entered to select right-wing news (rightnews) sources searches for the following strings from a variable containing the respondents’ news sources (news):

```
replace rightnews = regexm(news, "alex jones") | regexm(news, "brei") | regexm(news, "bart") | regexm(news, "carlson") |
	regexm(news, "conservative") | regexm(news, "caller") | regexm(news, "drudge") | ///
	regexm(news, "epoch") | regexm(news, "fox") | regexm(news, "freerepublic") | regexm(news, "beck") |
	regexm(news, "hannity") | regexm(news, "limbaugh") | regexm(news, "national review") | ///
	regexm(news, "newsmax") | regexm(news, "oan") | regexm(news, "one america") | ///
	regexm(news, "oneamericanewsnetwork") | regexm(news, "trump") | regexm(news, "shoah") | ///
	regexm(news, "epoch") | regexm(news, "blaze") | regexm(news, "worldnet") | regexm(news, "wnd") |
	regexm(news, "rt a") | regexm(news, "wire") | regexm(news, "reilly") | regexm(news, "christian") |
	regexm(news, "god news") | regexm(news, "rt news") | regexm(news, "rush") | regexm(news, "shapiro") |
	news1=="rt" | news2=="rt" | news3=="rt" | news4=="rt"
```

We analyze these data using ordinary least squares regression.

S23 Table. Regression Results, Health Behaviors, Adjusting for News Source.

|  | (1) | (2) | (3) | (4) | (5) | (6) | (7) | (8) | (9) |
| --- | --- | --- | --- | --- | --- | --- | --- | --- | --- |
|  | washhands | boughtsanitizer | visitdoctor | changedtravel | avoidcontact | avoidgatherings | soughtinfo | selfquarantine | changehealth |
| main |  |  |  |  |  |  |  |  |  |
| Republican | -.755 | -.402 | .252 | -.258 | -.658 | -.649 | -.692 | -.456 | -1.149 |
|  | .169 | .113 | .255 | .123 | .118 | .136 | .115 | .116 | .266 |
|  | [-1.086, -.423] | [-.624, -.180] | [-.248, .753] | [-.499, -.018] | [-.890, -.426] | [-.916, -.381] | [-.917, -.468] | [-.682, -.229] | [-1.669, -.628] |
|  | (<.001) | (<.001) | (.324) | (.035) | (<.001) | (<.001) | (<.001) | (<.001) | (<.001) |
| Other | -.860 | -.394 | -.289 | -.308 | -.546 | -.523 | -.494 | -.376 | -1.364 |
|  | .139 | .094 | .226 | .099 | .098 | .111 | .095 | .096 | .227 |
|  | [-1.133, -.587] | [-.578, -.211] | [-.733, .154] | [-.502, -.114] | [-.739, -.353] | [-.740, -.305] | [-.679, -.308] | [-.564, -.188] | [-1.809, -.920] |
|  | (<.001) | (<.001) | (.201) | (.002) | (<.001) | (<.001) | (<.001) | (<.001) | (<.001) |
| rightnews=1 | -.192 | .094 | -.404 | -.128 | -.400 | -.169 | -.369 | -.211 | -.187 |
|  | .144 | .111 | .296 | .121 | .110 | .127 | .111 | .115 | .197 |
|  | [-.475, .091] | [-.124, .311] | [-.984, .176] | [-.366, .109] | [-.615, -.185] | [-.417, .079] | [-.587, -.151] | [-.436, .014] | [-.572, .199] |
|  | (.183) | (.398) | (.172) | (.290) | (<.001) | (.182) | (.001) | (.066) | (.342) |
| 35- | .053 | .221 | .023 | -.236 | .008 | -.050 | -.027 | -.189 | -.285 |
|  | .157 | .116 | .256 | .122 | .118 | .130 | .118 | .118 | .241 |
|  | [-.255, .362] | [-.008, .449] | [-.478, .525] | [-.475, .003] | [-.223, .239] | [-.306, .205] | [-.259, .205] | [-.420, .042] | [-.758, .188] |
|  | (.735) | (.058) | (.927) | (.053) | (.948) | (.699) | (.821) | (.109) | (.238) |
| 50- | .224 | .216 | -.126 | -.306 | .124 | .336 | -.113 | -.315 | -.286 |
|  | .154 | .111 | .264 | .115 | .113 | .129 | .111 | .112 | .231 |
|  | [-.078, .525] | [-.001, .432] | [-.644, .392] | [-.532, -.080] | [-.097, .346] | [.082, .589] | [-.331, .105] | [-.535, -.095] | [-.740, .167] |
|  | (.146) | (.051) | (.633) | (.008) | (.271) | (.009) | (.309) | (.005) | (.216) |
| 70- | .583 | .286 | -.231 | -.092 | .581 | 1.012 | -.260 | .311 | .425 |
|  | .211 | .139 | .340 | .147 | .152 | .186 | .139 | .139 | .339 |
|  | [.169, .997] | [.014, .559] | [-.898, .436] | [-.381, .196] | [.283, .880] | [.649, 1.376] | [-.533, .012] | [.038, .585] | [-.239, 1.088] |
|  | (.006) | (.039) | (.497) | (.530) | (<.001) | (<.001) | (.061) | (.026) | (.209) |
| gender | .336 | .076 | -.090 | -.073 | .010 | .251 | .140 | .266 | .676 |
|  | .111 | .079 | .183 | .085 | .083 | .093 | .081 | .083 | .162 |
|  | [.119, .553] | [-.080, .231] | [-.449, .269] | [-.240, .094] | [-.152, .173] | [.068, .433] | [-.018, .298] | [.103, .428] | [.359, .993] |
|  | (.002) | (.341) | (.622) | (.393) | (.903) | (.007) | (.082) | (.001) | (<.001) |
| white | -.074 | -.358 | -.650 | -.173 | .212 | .297 | .363 | .010 | .102 |
|  | .135 | .094 | .209 | .102 | .098 | .110 | .097 | .098 | .199 |
|  | [-.339, .192] | [-.541, -.175] | [-1.059, -.242] | [-.372, .027] | [.021, .403] | [.082, .513] | [.174, .553] | [-.183, .203] | [-.288, .492] |
|  | (.585) | (<.001) | (.002) | (.090) | (.030) | (.007) | (<.001) | (.919) | (.608) |
| married | -.033 | .198 | .356 | .220 | .177 | -.023 | .110 | .052 | .368 |
|  | .121 | .087 | .213 | .093 | .091 | .103 | .088 | .090 | .169 |
|  | [-.271, .204] | [.027, .369] | [-.062, .774] | [.038, .402] | [-.002, .355] | [-.225, .180] | [-.062, .283] | [-.125, .228] | [.036, .700] |
|  | (.784) | (.023) | (.095) | (.018) | (.052) | (.827) | (.209) | (.566) | (.030) |
| High school graduate | -.147 | .032 | -.054 | .564 | -.598 | -.243 | -.014 | -.019 | -.113 |
|  | .291 | .231 | .493 | .313 | .246 | .256 | .235 | .242 | .364 |
|  | [-.717, .422] | [-.420, .484] | [-1.021, .913] | [-.049, 1.178] | [-1.080, -.116] | [-.745, .259] | [-.475, .447] | [-.493, .456] | [-.827, .601] |
|  | (.612) | (.891) | (.913) | (.072) | (.015) | (.342) | (.953) | (.938) | (.756) |
| Some college | .153 | -.035 | -.486 | 1.027 | -.319 | .113 | .564 | .059 | .380 |
|  | .297 | .235 | .517 | .314 | .250 | .262 | .238 | .247 | .379 |
|  | [-.429, .736] | [-.496, .426] | [-1.500, .528] | [.412, 1.642] | [-.809, .172] | [-.402, .627] | [.097, 1.032] | [-.425, .542] | [-.362, 1.122] |
|  | (.606) | (.881) | (.348) | (.001) | (.203) | (.668) | (.018) | (.811) | (.316) |
| 2-year | .096 | .041 | -.230 | .799 | -.369 | .064 | .512 | -.057 | .007 |
|  | .316 | .248 | .541 | .326 | .261 | .277 | .250 | .260 | .401 |
|  | [-.523, .714] | [-.444, .527] | [-1.291, .831] | [.161, 1.438] | [-.881, .143] | [-.478, .607] | [.021, 1.003] | [-.567, .453] | [-.779, .794] |
|  | (.761) | (.867) | (.671) | (.014) | (.158) | (.816) | (.041) | (.827) | (.985) |
| 4-year | .546 | -.113 | .269 | 1.165 | -.247 | .299 | .624 | .104 | 1.199 |
|  | .317 | .240 | .520 | .317 | .256 | .272 | .244 | .251 | .441 |
|  | [-.075, 1.168] | [-.585, .358] | [-.750, 1.287] | [.543, 1.787] | [-.749, .255] | [-.233, .832] | [.146, 1.101] | [-.389, .597] | [.334,2.064] |
|  | (.085) | (.637) | (.605) | (<.001) | (.334) | (.270) | (.011) | (.679) | (.007) |
| Post-grad | .477 | -.071 | .430 | 1.608 | .105 | .559 | .984 | .281 | 1.039 |
|  | .344 | .253 | .564 | .324 | .274 | .298 | .260 | .262 | .490 |
|  | [-.197, 1.150] | [-.567, .424] | [-.676, 1.536] | [.972,2.243] | [-.433, .643] | [-.025, 1.143] | [.474, 1.494] | [-.232, .794] | [.079, 1.998] |
|  | (.166) | (.777) | (.446) | (<.001) | (.702) | (.060) | (<.001) | (.283) | (.034) |
| income=2 | -.102 | -.173 | -1.307 | -.039 | -.011 | -.142 | .100 | -.267 | .055 |
|  | .294 | .224 | .494 | .260 | .229 | .245 | .226 | .228 | .385 |
|  | [-.678, .474] | [-.612, .266] | [-2.275, -.340] | [-.549, .471] | [-.460, .439] | [-.623, .339] | [-.343, .542] | [-.713, .180] | [-.700, .809] |
|  | (.729) | (.441) | (.008) | (.880) | (.963) | (.563) | (.659) | (.242) | (.887) |
| income=3 | -.255 | -.228 | -.807 | -.132 | -.185 | .005 | .215 | -.570 | -.010 |
|  | .280 | .218 | .406 | .253 | .221 | .240 | .215 | .221 | .360 |
|  | [-.804, .294] | [-.655, .199] | [-1.603, -.012] | [-.627, .364] | [-.618, .249] | [-.466, .475] | [-.205, .636] | [-1.003, -.136] | [-.715, .696] |
|  | (.362) | (.296) | (.047) | (.603) | (.403) | (.984) | (.315) | (.010) | (.978) |
| income=4 | .138 | .265 | -.931 | .011 | .160 | .338 | .452 | -.564 | .569 |
|  | .298 | .219 | .424 | .257 | .230 | .253 | .220 | .227 | .420 |
|  | [-.446, .722] | [-.164, .694] | [-1.762, -.099] | [-.492, .515] | [-.289, .610] | [-.157, .833] | [.021, .884] | [-1.008, -.119] | [-.254, 1.391] |
|  | (.644) | (.226) | (.028) | (.965) | (.484) | (.181) | (.040) | (.013) | (.175) |
| income=5 | .279 | -.085 | -.884 | -.228 | -.065 | .252 | .539 | -.571 | .129 |
|  | .304 | .227 | .445 | .264 | .232 | .255 | .228 | .231 | .387 |
|  | [-.317, .875] | [-.531, .360] | [-1.756, -.011] | [-.745, .290] | [-.520, .389] | [-.247, .751] | [.093, .986] | [-1.023, -.118] | [-.629, .887] |
|  | (.359) | (.707) | (.047) | (.389) | (.778) | (.323) | (.018) | (.013) | (.739) |
| income=6 | .358 | .017 | -.762 | -.035 | .237 | .469 | .318 | -.514 | .811 |
|  | .320 | .229 | .431 | .260 | .242 | .267 | .230 | .234 | .479 |
|  | [-.270, .985] | [-.432, .466] | [-1.607, .083] | [-.544, .475] | [-.236, .711] | [-.053, .992] | [-.133, .768] | [-.974, -.055] | [-.128, 1.749] |
|  | (.264) | (.941) | (.077) | (.893) | (.326) | (.079) | (.167) | (.028) | (.091) |
| income=7 | .196 | -.037 | -.726 | .549 | -.021 | .407 | .497 | -.680 | .353 |
|  | .335 | .245 | .423 | .269 | .252 | .279 | .243 | .255 | .477 |
|  | [-.460, .853] | [-.516, .443] | [-1.555, .102] | [.021, 1.076] | [-.515, .473] | [-.140, .954] | [.022, .972] | [-1.179, -.180] | [-.582, 1.287] |
|  | (.558) | (.880) | (.086) | (.041) | (.933) | (.144) | (.040) | (.008) | (.460) |
| income=8 | -.023 | -.363 | -1.451 | .304 | -.120 | .244 | .070 | -.335 | .222 |
|  | .323 | .241 | .520 | .263 | .244 | .270 | .237 | .241 | .450 |
|  | [-.657, .611] | [-.835, .109] | [-2.471, -.431] | [-.211, .819] | [-.598, .359] | [-.285, .773] | [-.395, .535] | [-.807, .137] | [-.661, 1.104] |
|  | (.943) | (.132) | (.005) | (.248) | (.624) | (.366) | (.767) | (.164) | (.622) |
| income=9 | .731 | .237 | -1.295 | .276 | .081 | .242 | .497 | -.670 | .793 |
|  | .354 | .233 | .493 | .261 | .250 | .271 | .238 | .242 | .485 |
|  | [.038, 1.425] | [-.220, .695] | [-2.261, -.328] | [-.235, .787] | [-.408, .570] | [-.289, .773] | [.031, .964] | [-1.143, -.196] | [-.158, 1.745] |
|  | (.039) | (.309) | (.009) | (.289) | (.746) | (.373) | (.037) | (.006) | (.102) |
| income=10 | .282 | -.129 | -1.339 | .417 | .278 | .417 | .137 | -.636 | .755 |
|  | .341 | .247 | .494 | .268 | .256 | .280 | .247 | .253 | .502 |
|  | [-.387, .952] | [-.613, .356] | [-2.308, -.371] | [-.109, .943] | [-.223, .779] | [-.133, .966] | [-.347, .620] | [-1.131, -.141] | [-.230, 1.740] |
|  | (.408) | (.603) | (.007) | (.120) | (.277) | (.137) | (.580) | (.012) | (.133) |
| income=11 | .136 | .183 | -1.466 | .517 | .056 | .248 | .310 | -.486 | -.175 |
|  | .346 | .250 | .515 | .272 | .260 | .284 | .251 | .252 | .458 |
|  | [-.542, .814] | [-.307, .673] | [-2.477, -.456] | [-.015, 1.050] | [-.453, .565] | [-.310, .805] | [-.182, .802] | [-.980, .007] | [-1.072, .723] |
|  | (.694) | (.464) | (.004) | (.057) | (.829) | (.384) | (.217) | (.053) | (.703) |
| income=12 | .836 | .192 | -1.163 | .507 | .314 | .343 | .546 | -.462 | .602 |
|  | .454 | .276 | .571 | .295 | .302 | .329 | .290 | .281 | .637 |
|  | [-.054, 1.726] | [-.348, .733] | [-2.281, -.045] | [-.071, 1.084] | [-.279, .907] | [-.302, .988] | [-.022, 1.115] | [-1.013, .089] | [-.645, 1.850] |
|  | (.066) | (.486) | (.042) | (.086) | (.299) | (.298) | (.059) | (.100) | (.344) |
| income=13 | .511 | .044 | .000 | .973 | .363 | .863 | 1.194 | -.274 | .794 |
|  | .604 | .387 | . | .404 | .431 | .542 | .435 | .386 | 1.020 |
|  | [-.673, 1.694] | [-.716, .803] | [.000, .000] | [.180, 1.766] | [-.482, 1.208] | [-.200, 1.927] | [.341,2.048] | [-1.030, .482] | [-1.204,2.792] |
|  | (.398) | (.910) | (.) | (.016) | (.400) | (.111) | (.006) | (.477) | (.436) |
| income=14 | -.153 | .342 | -.648 | 1.434 | .595 | .710 | .311 | -.152 | .000 |
|  | .631 | .398 | .673 | .439 | .502 | .565 | .411 | .396 | . |
|  | [-1.390, 1.084] | [-.438, 1.122] | [-1.966, .671] | [.573,2.296] | [-.389, 1.578] | [-.397, 1.817] | [-.495, 1.117] | [-.928, .624] | [.000, .000] |
|  | (.809) | (.390) | (.336) | (.001) | (.236) | (.209) | (.449) | (.701) | (.) |
| income=15 | .000 | .766 | .423 | 1.162 | .000 | .000 | .000 | .880 | .000 |
|  | . | 1.264 | 1.181 | 1.478 | . | . | . | 1.590 | . |
|  | [.000, .000] | [-1.711,3.243] | [-1.892,2.738] | [-1.735,4.059] | [.000, .000] | [.000, .000] | [.000, .000] | [-2.237,3.997] | [.000, .000] |
|  | (.) | (.544) | (.720) | (.432) | (.) | (.) | (.) | (.580) | (.) |
| income=16 | .000 | 1.730 | -.724 | .871 | 1.064 | .574 | .924 | -.473 | .000 |
|  | . | .660 | 1.187 | .533 | .844 | .830 | .690 | .568 | . |
|  | [.000, .000] | [.436,3.025] | [-3.050, 1.602] | [-.174, 1.917] | [-.590,2.719] | [-1.052,2.200] | [-.429,2.276] | [-1.586, .641] | [.000, .000] |
|  | (.) | (.009) | (.542) | (.102) | (.207) | (.489) | (.181) | (.405) | (.) |
| income=17 | -.037 | -.049 | -1.784 | .102 | -.044 | .328 | .153 | -.431 | -.005 |
|  | .279 | .210 | .461 | .239 | .215 | .237 | .209 | .212 | .363 |
|  | [-.584, .511] | [-.460, .363] | [-2.687, -.880] | [-.366, .570] | [-.464, .377] | [-.136, .792] | [-.257, .564] | [-.846, -.016] | [-.716, .706] |
|  | (.895) | (.816) | (<.001) | (.670) | (.838) | (.166) | (.464) | (.042) | (.989) |
| Rural Code=2 | -.141 | -.140 | -.349 | .015 | .185 | .118 | -.017 | -.056 | -.428 |
|  | .148 | .105 | .247 | .113 | .110 | .126 | .104 | .108 | .214 |
|  | [-.431, .149] | [-.346, .067] | [-.834, .136] | [-.206, .235] | [-.030, .400] | [-.129, .365] | [-.221, .188] | [-.267, .156] | [-.848, -.008] |
|  | (.340) | (.186) | (.158) | (.897) | (.091) | (.350) | (.874) | (.606) | (.046) |
| Rural Code=3 | -.078 | -.137 | -.362 | .034 | .193 | -.149 | .009 | -.197 | -.399 |
|  | .199 | .144 | .383 | .152 | .149 | .165 | .140 | .151 | .265 |
|  | [-.469, .312] | [-.420, .145] | [-1.113, .389] | [-.264, .331] | [-.099, .485] | [-.473, .175] | [-.266, .284] | [-.493, .099] | [-.919, .121] |
|  | (.694) | (.340) | (.345) | (.825) | (.196) | (.367) | (.950) | (.192) | (.132) |
| Rural Code=4 | -.599 | .083 | .063 | -.237 | -.145 | -.006 | -.316 | -.570 | -.565 |
|  | .257 | .197 | .384 | .225 | .208 | .236 | .190 | .224 | .372 |
|  | [-1.103, -.095] | [-.302, .469] | [-.690, .815] | [-.678, .204] | [-.552, .262] | [-.468, .456] | [-.688, .056] | [-1.010, -.131] | [-1.294, .165] |
|  | (.020) | (.672) | (.871) | (.292) | (.484) | (.980) | (.096) | (.011) | (.129) |
| Rural Code=5 | -.361 | .014 | .000 | .646 | .330 | .423 | -.327 | -.617 | -.796 |
|  | .396 | .304 | . | .336 | .329 | .412 | .299 | .333 | .574 |
|  | [-1.136, .415] | [-.582, .610] | [.000, .000] | [-.013, 1.305] | [-.314, .974] | [-.384, 1.229] | [-.913, .260] | [-1.270, .035] | [-1.921, .330] |
|  | (.362) | (.962) | (.) | (.055) | (.315) | (.305) | (.275) | (.064) | (.166) |
| Rural Code=6 | -.402 | .024 | -.971 | -.020 | .032 | -.241 | -.069 | -.024 | -.684 |
|  | .253 | .195 | .624 | .220 | .201 | .218 | .202 | .206 | .361 |
|  | [-.898, .095] | [-.358, .406] | [-2.194, .251] | [-.451, .412] | [-.363, .426] | [-.668, .185] | [-.464, .326] | [-.428, .379] | [-1.391, .022] |
|  | (.113) | (.901) | (.120) | (.929) | (.874) | (.268) | (.733) | (.906) | (.058) |
| Rural Code=7 | -.762 | -.012 | .131 | .044 | -.357 | -.155 | -.117 | -.409 | -.221 |
|  | .335 | .274 | .562 | .304 | .268 | .294 | .295 | .315 | .497 |
|  | [-1.418, -.107] | [-.550, .526] | [-.970, 1.232] | [-.552, .641] | [-.883, .168] | [-.731, .420] | [-.696, .462] | [-1.026, .207] | [-1.195, .753] |
|  | (.023) | (.966) | (.815) | (.885) | (.183) | (.597) | (.692) | (.193) | (.656) |
| Rural Code=8 | -.752 | -.122 | .000 | -1.248 | -.700 | -.500 | -.704 | -.931 | -1.202 |
|  | .585 | .538 | . | .837 | .533 | .592 | .548 | .717 | .776 |
|  | [-1.899, .396] | [-1.177, .933] | [.000, .000] | [-2.889, .392] | [-1.746, .345] | [-1.660, .660] | [-1.779, .371] | [-2.336, .474] | [-2.723, .319] |
|  | (.199) | (.820) | (.) | (.136) | (.189) | (.398) | (.199) | (.194) | (.121) |
| Rural Code=9 | -.920 | -.487 | -.079 | -.245 | -.147 | -.092 | .244 | -.072 | -.077 |
|  | .557 | .480 | .976 | .613 | .434 | .514 | .457 | .453 | .878 |
|  | [-2.011, .171] | [-1.427, .453] | [-1.992, 1.833] | [-1.446, .956] | [-.997, .703] | [-1.100, .915] | [-.651, 1.140] | [-.960, .816] | [-1.797, 1.644] |
|  | (.098) | (.310) | (.935) | (.689) | (.734) | (.857) | (.593) | (.874) | (.931) |
| Alaska | .000 | .677 | .000 | 1.387 | -.076 | .975 | -.177 | .944 | .000 |
|  | . | .788 | . | .762 | .850 | 1.002 | .956 | .840 | . |
|  | [.000, .000] | [-.868,2.222] | [.000, .000] | [-.106,2.880] | [-1.743, 1.591] | [-.989,2.939] | [-2.051, 1.697] | [-.701,2.590] | [.000, .000] |
|  | (.) | (.390) | (.) | (.069) | (.928) | (.330) | (.853) | (.261) | (.) |
| Arizona | .331 | -.837 | -.652 | -.080 | .095 | .059 | -.543 | .510 | -.101 |
|  | .485 | .408 | .831 | .444 | .415 | .417 | .432 | .447 | .641 |
|  | [-.619, 1.282] | [-1.637, -.037] | [-2.281, .977] | [-.949, .790] | [-.718, .908] | [-.757, .876] | [-1.389, .303] | [-.366, 1.386] | [-1.356, 1.154] |
|  | (.495) | (.040) | (.433) | (.858) | (.819) | (.887) | (.209) | (.254) | (.875) |
| Arkansas | .810 | -.278 | -.531 | -.060 | .040 | -.069 | -.253 | .405 | .321 |
|  | .591 | .446 | .960 | .498 | .455 | .473 | .475 | .504 | .768 |
|  | [-.348, 1.969] | [-1.152, .596] | [-2.413, 1.351] | [-1.037, .916] | [-.852, .933] | [-.997, .858] | [-1.184, .678] | [-.583, 1.393] | [-1.186, 1.827] |
|  | (.170) | (.533) | (.580) | (.903) | (.929) | (.883) | (.595) | (.422) | (.676) |
| California | .585 | -.099 | -.744 | .319 | .268 | .592 | -.237 | .838 | 1.088 |
|  | .427 | .345 | .680 | .377 | .359 | .364 | .385 | .397 | .599 |
|  | [-.252, 1.423] | [-.774, .576] | [-2.077, .588] | [-.420, 1.058] | [-.436, .973] | [-.121, 1.306] | [-.992, .518] | [.061, 1.615] | [-.086,2.261] |
|  | (.171) | (.774) | (.274) | (.398) | (.455) | (.104) | (.538) | (.035) | (.069) |
| Colorado | 1.943 | .019 | -1.246 | .853 | .724 | .911 | .240 | .717 | 1.045 |
|  | .838 | .456 | 1.178 | .474 | .480 | .526 | .511 | .496 | .917 |
|  | [.302,3.585] | [-.875, .912] | [-3.556, 1.064] | [-.076, 1.782] | [-.217, 1.665] | [-.120, 1.941] | [-.761, 1.241] | [-.254, 1.689] | [-.752,2.841] |
|  | (.020) | (.968) | (.290) | (.072) | (.132) | (.083) | (.639) | (.148) | (.254) |
| Connecticut | -.060 | -.077 | .000 | .199 | .938 | .738 | -.203 | 1.130 | 1.590 |
|  | .616 | .485 | . | .528 | .554 | .585 | .510 | .539 | 1.230 |
|  | [-1.267, 1.147] | [-1.028, .873] | [.000, .000] | [-.836, 1.233] | [-.148,2.023] | [-.408, 1.885] | [-1.202, .797] | [.074,2.186] | [-.820,4.000] |
|  | (.922) | (.873) | (.) | (.707) | (.090) | (.207) | (.691) | (.036) | (.196) |
| Delaware | .000 | .951 | .000 | -.178 | .000 | .670 | .330 | 1.179 | .000 |
|  | . | .722 | . | .705 | . | .869 | .640 | .681 | . |
|  | [.000, .000] | [-.464,2.366] | [.000, .000] | [-1.559, 1.204] | [.000, .000] | [-1.034,2.373] | [-.924, 1.585] | [-.154,2.513] | [.000, .000] |
|  | (.) | (.188) | (.) | (.801) | (.) | (.441) | (.606) | (.083) | (.) |
| District of Columbia | .403 | -.870 | .000 | 1.489 | 1.021 | 1.270 | -.509 | 1.509 | -.422 |
|  | 1.078 | .843 | . | .728 | .827 | 1.033 | .797 | .791 | 1.081 |
|  | [-1.709,2.515] | [-2.521, .782] | [.000, .000] | [.062,2.915] | [-.599,2.642] | [-.755,3.294] | [-2.071, 1.053] | [-.043,3.060] | [-2.542, 1.697] |
|  | (.708) | (.302) | (.) | (.041) | (.217) | (.219) | (.523) | (.057) | (.696) |
| Florida | .661 | .125 | -.830 | .024 | .411 | .404 | -.312 | .630 | 1.037 |
|  | .436 | .347 | .703 | .383 | .363 | .367 | .388 | .402 | .614 |
|  | [-.193, 1.516] | [-.556, .806] | [-2.207, .548] | [-.727, .775] | [-.301, 1.123] | [-.315, 1.123] | [-1.073, .449] | [-.159, 1.418] | [-.166,2.240] |
|  | (.129) | (.719) | (.238) | (.950) | (.258) | (.271) | (.422) | (.118) | (.091) |
| Georgia | .378 | -.101 | -.820 | .725 | .385 | .607 | -.444 | .510 | .515 |
|  | .473 | .382 | .790 | .404 | .395 | .406 | .416 | .434 | .653 |
|  | [-.550, 1.305] | [-.849, .648] | [-2.367, .728] | [-.067, 1.517] | [-.389, 1.159] | [-.188, 1.402] | [-1.259, .371] | [-.340, 1.361] | [-.765, 1.795] |
|  | (.425) | (.792) | (.299) | (.073) | (.330) | (.135) | (.285) | (.240) | (.431) |
| Hawaii | .893 | .122 | .203 | -2.114 | -.440 | 1.430 | -.154 | .380 | .000 |
|  | 1.203 | .707 | 1.304 | 1.106 | .689 | 1.026 | .738 | .739 | . |
|  | [-1.464,3.250] | [-1.265, 1.508] | [-2.353,2.758] | [-4.283, .055] | [-1.792, .911] | [-.581,3.441] | [-1.601, 1.292] | [-1.069, 1.828] | [.000, .000] |
|  | (.458) | (.864) | (.877) | (.056) | (.523) | (.163) | (.834) | (.607) | (.) |
| Idaho | -.510 | -.370 | .000 | .432 | -.401 | .023 | -1.346 | -.340 | -.700 |
|  | .621 | .583 | . | .572 | .588 | .585 | .616 | .716 | .735 |
|  | [-1.727, .707] | [-1.512, .771] | [.000, .000] | [-.689, 1.553] | [-1.553, .751] | [-1.125, 1.170] | [-2.553, -.139] | [-1.743, 1.063] | [-2.140, .740] |
|  | (.412) | (.525) | (.) | (.450) | (.495) | (.969) | (.029) | (.635) | (.341) |
| Illinois | .126 | -.605 | -1.016 | .534 | .119 | .257 | -.412 | .902 | .596 |
|  | .454 | .383 | .818 | .406 | .388 | .395 | .413 | .421 | .652 |
|  | [-.764, 1.017] | [-1.356, .146] | [-2.618, .587] | [-.261, 1.329] | [-.641, .879] | [-.517, 1.031] | [-1.221, .397] | [.076, 1.728] | [-.682, 1.874] |
|  | (.781) | (.114) | (.214) | (.188) | (.760) | (.516) | (.318) | (.032) | (.361) |
| Indiana | .268 | -.554 | -.856 | .502 | .554 | .961 | -.623 | .861 | .380 |
|  | .487 | .434 | .945 | .448 | .437 | .476 | .447 | .454 | .682 |
|  | [-.686, 1.223] | [-1.404, .297] | [-2.709, .997] | [-.375, 1.380] | [-.302, 1.410] | [.027, 1.895] | [-1.500, .253] | [-.030, 1.751] | [-.957, 1.718] |
|  | (.582) | (.202) | (.365) | (.262) | (.204) | (.044) | (.163) | (.058) | (.577) |
| Iowa | 1.596 | .042 | .154 | .156 | -.241 | .529 | -.236 | -.354 | .000 |
|  | .832 | .492 | 1.044 | .567 | .498 | .566 | .520 | .606 | . |
|  | [-.035,3.226] | [-.922, 1.006] | [-1.891,2.200] | [-.954, 1.267] | [-1.218, .736] | [-.581, 1.639] | [-1.255, .783] | [-1.541, .833] | [.000, .000] |
|  | (.055) | (.932) | (.883) | (.783) | (.628) | (.350) | (.650) | (.559) | (.) |
| Kansas | 1.741 | -.746 | .000 | -.113 | .358 | .968 | -.324 | .459 | 1.127 |
|  | 1.087 | .661 | . | .675 | .595 | .726 | .610 | .642 | 1.108 |
|  | [-.389,3.871] | [-2.041, .550] | [.000, .000] | [-1.435, 1.209] | [-.807, 1.524] | [-.456,2.392] | [-1.520, .872] | [-.800, 1.717] | [-1.044,3.298] |
|  | (.109) | (.259) | (.) | (.867) | (.547) | (.183) | (.595) | (.475) | (.309) |
| Kentucky | 1.019 | -.483 | -.650 | -1.283 | .556 | .289 | -.278 | -.069 | .533 |
|  | .634 | .460 | .933 | .658 | .485 | .496 | .482 | .533 | .865 |
|  | [-.223,2.262] | [-1.385, .419] | [-2.478, 1.178] | [-2.573, .006] | [-.395, 1.508] | [-.683, 1.262] | [-1.224, .667] | [-1.114, .976] | [-1.163,2.229] |
|  | (.108) | (.294) | (.486) | (.051) | (.252) | (.560) | (.564) | (.898) | (.538) |
| Louisiana | .775 | -.412 | -1.698 | -.553 | .607 | .860 | -.253 | .463 | 1.846 |
|  | .614 | .462 | 1.171 | .543 | .481 | .518 | .483 | .510 | 1.128 |
|  | [-.428, 1.978] | [-1.318, .494] | [-3.993, .597] | [-1.618, .512] | [-.335, 1.549] | [-.156, 1.875] | [-1.199, .694] | [-.537, 1.463] | [-.365,4.057] |
|  | (.207) | (.373) | (.147) | (.309) | (.207) | (.097) | (.601) | (.364) | (.102) |
| Maine | 1.006 | -1.063 | .000 | .883 | 1.244 | 1.346 | 1.675 | 1.065 | 1.077 |
|  | .817 | .732 | . | .691 | .721 | .773 | .811 | .677 | 1.059 |
|  | [-.595,2.607] | [-2.498, .371] | [.000, .000] | [-.471,2.238] | [-.170,2.657] | [-.168,2.861] | [.086,3.265] | [-.261,2.392] | [-.998,3.153] |
|  | (.218) | (.146) | (.) | (.201) | (.085) | (.082) | (.039) | (.115) | (.309) |
| Maryland | 1.035 | -.125 | -1.581 | .045 | .420 | .940 | -.145 | -.117 | .546 |
|  | .722 | .452 | 1.236 | .477 | .474 | .555 | .479 | .548 | .893 |
|  | [-.380,2.449] | [-1.011, .760] | [-4.004, .842] | [-.890, .981] | [-.510, 1.350] | [-.147,2.027] | [-1.085, .794] | [-1.192, .958] | [-1.204,2.296] |
|  | (.152) | (.781) | (.201) | (.924) | (.376) | (.090) | (.762) | (.831) | (.541) |
| Massachusetts | .483 | -.409 | .000 | .527 | .792 | 1.047 | -.829 | .810 | .796 |
|  | .534 | .436 | . | .448 | .454 | .494 | .456 | .466 | .742 |
|  | [-.563, 1.530] | [-1.263, .445] | [.000, .000] | [-.352, 1.406] | [-.098, 1.682] | [.078,2.016] | [-1.722, .064] | [-.103, 1.724] | [-.658,2.250] |
|  | (.365) | (.348) | (.) | (.240) | (.081) | (.034) | (.069) | (.082) | (.283) |
| Michigan | .941 | -.217 | -.243 | -.285 | .518 | .480 | -.518 | .429 | 1.407 |
|  | .491 | .375 | .734 | .416 | .389 | .403 | .406 | .431 | .733 |
|  | [-.021, 1.904] | [-.951, .517] | [-1.683, 1.196] | [-1.100, .531] | [-.245, 1.281] | [-.311, 1.270] | [-1.315, .278] | [-.415, 1.274] | [-.030,2.844] |
|  | (.055) | (.562) | (.740) | (.494) | (.184) | (.234) | (.202) | (.319) | (.055) |
| Minnesota | .975 | -.529 | -1.521 | .371 | .490 | .527 | -.158 | .651 | 1.663 |
|  | .644 | .464 | 1.202 | .478 | .479 | .504 | .502 | .499 | 1.121 |
|  | [-.288,2.238] | [-1.439, .381] | [-3.877, .835] | [-.567, 1.309] | [-.449, 1.429] | [-.460, 1.515] | [-1.143, .826] | [-.327, 1.630] | [-.534,3.860] |
|  | (.130) | (.255) | (.206) | (.438) | (.307) | (.295) | (.753) | (.192) | (.138) |
| Mississippi | .639 | -.312 | -.335 | -.636 | .621 | .354 | .146 | 1.112 | 1.694 |
|  | .653 | .505 | .980 | .614 | .534 | .545 | .523 | .555 | 1.220 |
|  | [-.641, 1.918] | [-1.303, .678] | [-2.256, 1.585] | [-1.839, .567] | [-.426, 1.669] | [-.713, 1.422] | [-.880, 1.172] | [.024,2.200] | [-.697,4.086] |
|  | (.328) | (.537) | (.732) | (.300) | (.245) | (.516) | (.780) | (.045) | (.165) |
| Missouri | .084 | -.572 | -.566 | .021 | .159 | .834 | -.395 | .199 | .299 |
|  | .479 | .419 | .872 | .449 | .417 | .457 | .441 | .465 | .666 |
|  | [-.854, 1.023] | [-1.392, .249] | [-2.276, 1.144] | [-.860, .902] | [-.659, .977] | [-.062, 1.730] | [-1.258, .469] | [-.712, 1.110] | [-1.005, 1.604] |
|  | (.860) | (.172) | (.516) | (.963) | (.703) | (.068) | (.371) | (.669) | (.653) |
| Montana | 1.066 | -.337 | .000 | .392 | .311 | .644 | -.818 | .749 | .683 |
|  | .817 | .684 | . | .672 | .616 | .712 | .663 | .677 | 1.185 |
|  | [-.536,2.668] | [-1.678, 1.005] | [.000, .000] | [-.925, 1.709] | [-.897, 1.518] | [-.752,2.039] | [-2.118, .481] | [-.577,2.074] | [-1.639,3.005] |
|  | (.192) | (.623) | (.) | (.559) | (.614) | (.366) | (.217) | (.269) | (.564) |
| Nebraska | .695 | -.090 | .000 | -.125 | .407 | .574 | -.240 | .023 | -.011 |
|  | .863 | .649 | . | .820 | .746 | .745 | .710 | .768 | .882 |
|  | [-.996,2.386] | [-1.362, 1.181] | [.000, .000] | [-1.732, 1.482] | [-1.055, 1.870] | [-.885,2.033] | [-1.631, 1.152] | [-1.483, 1.529] | [-1.739, 1.718] |
|  | (.420) | (.889) | (.) | (.879) | (.585) | (.441) | (.736) | (.976) | (.990) |
| Nevada | .489 | -.718 | -.813 | -.270 | 1.090 | .774 | -.212 | 1.086 | .767 |
|  | .602 | .458 | .971 | .512 | .513 | .509 | .490 | .489 | .826 |
|  | [-.692, 1.669] | [-1.615, .179] | [-2.717, 1.090] | [-1.273, .733] | [.084,2.096] | [-.223, 1.772] | [-1.172, .748] | [.128,2.044] | [-.853,2.386] |
|  | (.417) | (.116) | (.402) | (.598) | (.034) | (.128) | (.665) | (.026) | (.354) |
| New Hampshire | 1.099 | -.859 | -.462 | -.423 | .030 | .291 | -.292 | -.099 | 1.157 |
|  | .833 | .592 | 1.194 | .630 | .536 | .598 | .554 | .655 | 1.187 |
|  | [-.533,2.731] | [-2.020, .301] | [-2.802, 1.878] | [-1.657, .811] | [-1.020, 1.079] | [-.881, 1.464] | [-1.377, .793] | [-1.383, 1.184] | [-1.170,3.485] |
|  | (.187) | (.147) | (.699) | (.502) | (.956) | (.627) | (.598) | (.879) | (.330) |
| New Jersey | 1.005 | .162 | -.707 | .278 | .586 | .780 | -.248 | .278 | 1.479 |
|  | .574 | .408 | .851 | .440 | .430 | .457 | .438 | .464 | .875 |
|  | [-.119,2.129] | [-.637, .961] | [-2.375, .961] | [-.584, 1.140] | [-.257, 1.430] | [-.116, 1.675] | [-1.106, .609] | [-.631, 1.186] | [-.235,3.193] |
|  | (.080) | (.691) | (.406) | (.528) | (.173) | (.088) | (.571) | (.549) | (.091) |
| New Mexico | .841 | -.527 | -1.052 | .230 | .538 | -.236 | .243 | .643 | 1.013 |
|  | .684 | .509 | 1.114 | .548 | .569 | .534 | .553 | .553 | .970 |
|  | [-.500,2.181] | [-1.526, .471] | [-3.235, 1.130] | [-.845, 1.305] | [-.578, 1.653] | [-1.282, .810] | [-.841, 1.327] | [-.441, 1.726] | [-.887,2.913] |
|  | (.219) | (.301) | (.345) | (.675) | (.345) | (.658) | (.661) | (.245) | (.296) |
| New York | .087 | .126 | -.449 | .247 | .382 | .753 | -.214 | .494 | .405 |
|  | .433 | .355 | .687 | .390 | .374 | .387 | .399 | .411 | .606 |
|  | [-.761, .935] | [-.570, .822] | [-1.795, .897] | [-.518, 1.012] | [-.350, 1.115] | [-.006, 1.512] | [-.995, .568] | [-.312, 1.300] | [-.783, 1.592] |
|  | (.840) | (.723) | (.513) | (.526) | (.306) | (.052) | (.592) | (.230) | (.504) |
| North Carolina | .674 | .077 | -.408 | .274 | .458 | .670 | -.003 | .273 | .813 |
|  | .493 | .384 | .785 | .421 | .404 | .428 | .429 | .447 | .706 |
|  | [-.293, 1.641] | [-.676, .829] | [-1.947, 1.131] | [-.551, 1.099] | [-.334, 1.251] | [-.170, 1.509] | [-.844, .837] | [-.603, 1.149] | [-.571,2.196] |
|  | (.172) | (.842) | (.603) | (.515) | (.257) | (.118) | (.994) | (.541) | (.250) |
| North Dakota | .613 | 1.461 | .000 | -.185 | -.037 | 1.271 | -.150 | .681 | -.018 |
|  | 1.061 | .926 | . | .944 | .752 | 1.085 | .728 | .725 | 1.070 |
|  | [-1.467,2.693] | [-.354,3.277] | [.000, .000] | [-2.036, 1.666] | [-1.511, 1.437] | [-.856,3.398] | [-1.578, 1.277] | [-.741,2.103] | [-2.116,2.080] |
|  | (.563) | (.115) | (.) | (.845) | (.961) | (.242) | (.836) | (.348) | (.986) |
| Ohio | .776 | -.155 | -.683 | .270 | .425 | .810 | -.066 | .695 | 1.086 |
|  | .485 | .374 | .769 | .413 | .391 | .415 | .416 | .422 | .699 |
|  | [-.175, 1.727] | [-.888, .577] | [-2.191, .825] | [-.539, 1.080] | [-.341, 1.190] | [-.003, 1.624] | [-.881, .750] | [-.131, 1.522] | [-.284,2.455] |
|  | (.110) | (.678) | (.375) | (.513) | (.277) | (.051) | (.875) | (.099) | (.120) |
| Oklahoma | .377 | .348 | .341 | .398 | .642 | .541 | -.376 | .530 | 1.686 |
|  | .611 | .525 | .837 | .548 | .567 | .579 | .535 | .558 | 1.174 |
|  | [-.820, 1.575] | [-.680, 1.376] | [-1.300, 1.982] | [-.675, 1.471] | [-.470, 1.754] | [-.593, 1.675] | [-1.424, .672] | [-.564, 1.623] | [-.615,3.986] |
|  | (.537) | (.507) | (.683) | (.467) | (.258) | (.350) | (.482) | (.342) | (.151) |
| Oregon | .079 | -.573 | -1.730 | .314 | .315 | .619 | -.185 | .716 | .300 |
|  | .524 | .425 | 1.219 | .447 | .431 | .478 | .462 | .461 | .745 |
|  | [-.947, 1.106] | [-1.407, .261] | [-4.120, .659] | [-.562, 1.191] | [-.529, 1.159] | [-.317, 1.555] | [-1.090, .720] | [-.187, 1.619] | [-1.161, 1.760] |
|  | (.880) | (.178) | (.156) | (.482) | (.464) | (.195) | (.688) | (.120) | (.688) |
| Pennsylvania | .865 | -.490 | -.897 | .182 | .448 | .827 | -.394 | .516 | 1.045 |
|  | .448 | .356 | .731 | .387 | .370 | .381 | .392 | .409 | .619 |
|  | [-.013, 1.742] | [-1.188, .208] | [-2.329, .535] | [-.576, .940] | [-.276, 1.173] | [.080, 1.574] | [-1.162, .375] | [-.285, 1.317] | [-.168,2.258] |
|  | (.053) | (.169) | (.220) | (.638) | (.225) | (.030) | (.315) | (.207) | (.091) |
| Rhode Island | .758 | 1.320 | .543 | .765 | .953 | .538 | -.417 | .642 | .037 |
|  | 1.121 | .837 | 1.311 | .863 | .853 | .847 | .826 | .808 | 1.102 |
|  | [-1.439,2.956] | [-.320,2.960] | [-2.027,3.113] | [-.927,2.457] | [-.719,2.624] | [-1.122,2.199] | [-2.037, 1.203] | [-.942,2.226] | [-2.123,2.198] |
|  | (.499) | (.115) | (.679) | (.375) | (.264) | (.525) | (.614) | (.427) | (.973) |
| South Carolina | .677 | .042 | -1.498 | .192 | .881 | 1.083 | .129 | .647 | .717 |
|  | .564 | .434 | 1.213 | .487 | .482 | .522 | .473 | .478 | .744 |
|  | [-.429, 1.783] | [-.809, .892] | [-3.875, .879] | [-.763, 1.147] | [-.064, 1.826] | [.061,2.106] | [-.797, 1.056] | [-.289, 1.584] | [-.741,2.175] |
|  | (.230) | (.924) | (.217) | (.694) | (.068) | (.038) | (.785) | (.176) | (.335) |
| South Dakota | .060 | -1.848 | .000 | -.695 | -.640 | .494 | -.944 | .000 | -.293 |
|  | .894 | 1.101 | . | 1.016 | .743 | .836 | .819 | . | 1.070 |
|  | [-1.693, 1.812] | [-4.007, .310] | [.000, .000] | [-2.686, 1.296] | [-2.096, .816] | [-1.144,2.132] | [-2.550, .661] | [.000, .000] | [-2.389, 1.804] |
|  | (.947) | (.093) | (.) | (.494) | (.389) | (.555) | (.249) | (.) | (.784) |
| Tennessee | 1.313 | .378 | -.660 | .238 | .498 | 1.392 | .365 | .658 | 1.605 |
|  | .641 | .423 | .958 | .471 | .447 | .552 | .472 | .478 | .930 |
|  | [.057,2.570] | [-.452, 1.208] | [-2.537, 1.217] | [-.684, 1.160] | [-.378, 1.374] | [.310,2.474] | [-.559, 1.290] | [-.278, 1.595] | [-.218,3.427] |
|  | (.040) | (.372) | (.491) | (.613) | (.265) | (.012) | (.439) | (.168) | (.084) |
| Texas | .325 | -.059 | -.556 | .194 | .059 | .200 | -.651 | .027 | .154 |
|  | .431 | .352 | .687 | .390 | .365 | .370 | .394 | .414 | .575 |
|  | [-.519, 1.170] | [-.750, .632] | [-1.902, .791] | [-.570, .959] | [-.656, .773] | [-.525, .925] | [-1.423, .121] | [-.784, .838] | [-.972, 1.280] |
|  | (.450) | (.868) | (.419) | (.618) | (.872) | (.589) | (.098) | (.948) | (.789) |
| Utah | .439 | -.662 | .000 | .734 | .146 | .558 | -.204 | .534 | -.124 |
|  | .619 | .544 | . | .517 | .515 | .548 | .517 | .547 | .752 |
|  | [-.774, 1.652] | [-1.729, .405] | [.000, .000] | [-.280, 1.748] | [-.864, 1.155] | [-.517, 1.633] | [-1.217, .808] | [-.539, 1.607] | [-1.598, 1.351] |
|  | (.478) | (.224) | (.) | (.156) | (.777) | (.309) | (.693) | (.329) | (.869) |
| Vermont | .000 | -.607 | .000 | 1.045 | .947 | -.088 | .290 | -.888 | .000 |
|  | . | .756 | . | .777 | .917 | .850 | .692 | 1.160 | . |
|  | [.000, .000] | [-2.088, .874] | [.000, .000] | [-.477,2.567] | [-.850,2.745] | [-1.755, 1.579] | [-1.067, 1.647] | [-3.162, 1.387] | [.000, .000] |
|  | (.) | (.422) | (.) | (.178) | (.302) | (.917) | (.675) | (.444) | (.) |
| Virginia | .465 | .114 | -.779 | .227 | .199 | .752 | -.165 | .713 | .295 |
|  | .475 | .378 | .768 | .410 | .390 | .422 | .419 | .429 | .645 |
|  | [-.467, 1.397] | [-.627, .855] | [-2.284, .726] | [-.577, 1.031] | [-.566, .964] | [-.076, 1.580] | [-.986, .655] | [-.129, 1.554] | [-.968, 1.558] |
|  | (.328) | (.763) | (.310) | (.580) | (.611) | (.075) | (.693) | (.097) | (.647) |
| Washington | .896 | -.366 | -.022 | .315 | .730 | 1.065 | -.126 | .539 | .944 |
|  | .534 | .407 | .775 | .438 | .429 | .473 | .440 | .456 | .720 |
|  | [-.151, 1.943] | [-1.164, .431] | [-1.540, 1.496] | [-.544, 1.173] | [-.111, 1.572] | [.137, 1.992] | [-.987, .736] | [-.355, 1.433] | [-.467,2.356] |
|  | (.094) | (.368) | (.977) | (.473) | (.089) | (.024) | (.775) | (.238) | (.190) |
| West Virginia | .045 | -.165 | -.355 | .326 | -.140 | .201 | -.650 | -.127 | .508 |
|  | .635 | .541 | 1.255 | .595 | .539 | .590 | .531 | .614 | .796 |
|  | [-1.199, 1.290] | [-1.225, .894] | [-2.815,2.104] | [-.841, 1.492] | [-1.197, .918] | [-.956, 1.357] | [-1.691, .390] | [-1.332, 1.077] | [-1.052,2.068] |
|  | (.943) | (.760) | (.777) | (.584) | (.796) | (.734) | (.221) | (.836) | (.523) |
| Wisconsin | .519 | -.330 | -.305 | .505 | .373 | .700 | -.472 | .139 | 1.222 |
|  | .529 | .419 | .872 | .459 | .433 | .459 | .469 | .488 | .838 |
|  | [-.519, 1.557] | [-1.151, .490] | [-2.013, 1.404] | [-.393, 1.404] | [-.476, 1.222] | [-.200, 1.600] | [-1.391, .447] | [-.817, 1.096] | [-.420,2.864] |
|  | (.327) | (.430) | (.727) | (.271) | (.390) | (.128) | (.314) | (.776) | (.145) |
| Wyoming | .000 | 1.049 | 1.702 | .213 | .607 | .305 | .659 | 2.199 | .000 |
|  | . | 1.418 | 1.155 | 1.084 | 1.117 | 1.153 | 1.152 | 1.294 | . |
|  | [.000, .000] | [-1.730,3.828] | [-.561,3.965] | [-1.912,2.337] | [-1.582,2.796] | [-1.954,2.564] | [-1.599,2.918] | [-.337,4.735] | [.000, .000] |
|  | (.) | (.460) | (.140) | (.845) | (.587) | (.791) | (.567) | (.089) | (.) |
| Observations | 2930 | 2982 | 2630 | 2982 | 2965 | 2979 | 2979 | 2972 | 2853 |
| Pseudo-R^2^ | .0794 | .0475 | .0763 | .0809 | .0564 | .0696 | .0744 | .0531 | .142 |
| Model χ^2^ | 188.7 | 172.0 | 107.0 | 267.9 | 186.2 | 203.7 | 272.2 | 185.5 | 196.1 |
| d.f. | 82 | 88 | 71 | 88 | 86 | 87 | 87 | 87 | 79 |
| p-value | 2.18e-10 | .000000224 | .00372 | 4.60e-20 | 2.35e-09 | 2.37e-11 | 5.91e-21 | 4.26e-09 | 6.06e-12 |

S24 Table. Regression Results, Health Views, Adjusting for News Source.

|  | (1) | (2) | (3) | (4) | (5) | (6) | (7) | (8) | (9) |
| --- | --- | --- | --- | --- | --- | --- | --- | --- | --- |
|  | numberdeaths | spendingincrease | notenoughtests | getsick | negativeeconomy | friendsick | cannotreturn | schoolsclosed | hardgetitems |
| Republican | -.351 | -.271 | -.721 | -.463 | -.313 | -.421 | -.383 | -.243 | -.244 |
|  | .060 | .027 | .060 | .053 | .049 | .051 | .054 | .056 | .054 |
|  | [-.469, -.233] | [-.325, -.218] | [-.839, -.603] | [-.568, -.359] | [-.409, -.217] | [-.521, -.321] | [-.490, -.277] | [-.352, -.133] | [-.350, -.138] |
|  | (<.001) | (<.001) | (<.001) | (<.001) | (<.001) | (<.001) | (<.001) | (<.001) | (<.001) |
| Other | -.257 | -.211 | -.311 | -.326 | -.253 | -.349 | -.326 | -.197 | -.230 |
|  | .049 | .022 | .049 | .045 | .040 | .044 | .045 | .049 | .045 |
|  | [-.353, -.161] | [-.254, -.169] | [-.407, -.215] | [-.415, -.238] | [-.332, -.174] | [-.436, -.263] | [-.414, -.237] | [-.293, -.102] | [-.318, -.143] |
|  | (<.001) | (<.001) | (<.001) | (<.001) | (<.001) | (<.001) | (<.001) | (<.001) | (<.001) |
| rightnews=1 | -.016 | -.144 | -.348 | -.193 | -.025 | -.214 | -.132 | -.066 | -.155 |
|  | .057 | .029 | .059 | .052 | .049 | .050 | .052 | .052 | .051 |
|  | [-.128, .096] | [-.202, -.087] | [-.464, -.232] | [-.295, -.091] | [-.121, .070] | [-.311, -.116] | [-.234, -.029] | [-.168, .037] | [-.254, -.056] |
|  | (.779) | (<.001) | (<.001) | (<.001) | (.604) | (<.001) | (.012) | (.210) | (.002) |
| 35- | -.036 | .044 | .078 | .066 | -.045 | .071 | -.135 | .039 | .015 |
|  | .062 | .028 | .063 | .056 | .051 | .055 | .056 | .059 | .055 |
|  | [-.157, .084] | [-.012, .100] | [-.046, .202] | [-.044, .175] | [-.145, .055] | [-.036, .178] | [-.244, -.026] | [-.078, .155] | [-.093, .123] |
|  | (.555) | (.121) | (.216) | (.240) | (.378) | (.195) | (.015) | (.515) | (.784) |
| 50- | .155 | .047 | .176 | -.029 | .049 | .005 | -.201 | -.095 | -.175 |
|  | .059 | .027 | .058 | .053 | .048 | .051 | .052 | .057 | .052 |
|  | [.039, .272] | [-.005, .099] | [.061, .290] | [-.133, .074] | [-.045, .144] | [-.095, .105] | [-.304, -.098] | [-.207, .017] | [-.277, -.073] |
|  | (.009) | (.078) | (.003) | (.581) | (.303) | (.925) | (<.001) | (.096) | (.001) |
| 70- | .338 | .096 | .180 | .025 | -.036 | .021 | -.267 | -.534 | -.388 |
|  | .073 | .032 | .068 | .065 | .060 | .062 | .065 | .068 | .064 |
|  | [.195, .481] | [.034, .158] | [.046, .314] | [-.102, .152] | [-.154, .081] | [-.102, .143] | [-.394, -.139] | [-.667, -.400] | [-.514, -.261] |
|  | (<.001) | (.002) | (.008) | (.703) | (.542) | (.741) | (<.001) | (<.001) | (<.001) |
| gender | .047 | -.034 | -.007 | .134 | -.045 | .121 | .022 | .043 | .134 |
|  | .041 | .019 | .041 | .038 | .035 | .037 | .039 | .041 | .038 |
|  | [-.034, .127] | [-.071, .002] | [-.089, .074] | [.060, .209] | [-.113, .022] | [.049, .193] | [-.054, .098] | [-.037, .124] | [.059, .208] |
|  | (.257) | (.066) | (.857) | (<.001) | (.190) | (.001) | (.575) | (.294) | (<.001) |
| white | -.040 | .071 | .245 | .029 | .109 | .004 | .134 | -.076 | .026 |
|  | .051 | .023 | .052 | .047 | .042 | .045 | .047 | .049 | .046 |
|  | [-.140, .059] | [.025, .116] | [.143, .347] | [-.064, .121] | [.026, .192] | [-.084, .092] | [.042, .225] | [-.173, .020] | [-.064, .117] |
|  | (.428) | (.003) | (<.001) | (.540) | (.010) | (.926) | (.004) | (.119) | (.568) |
| married | .006 | -.008 | -.107 | .110 | .085 | .091 | .027 | .071 | .108 |
|  | .046 | .021 | .046 | .041 | .037 | .040 | .042 | .044 | .041 |
|  | [-.083, .096] | [-.049, .033] | [-.197, -.016] | [.029, .191] | [.012, .159] | [.012, .170] | [-.055, .108] | [-.016, .158] | [.028, .188] |
|  | (.889) | (.705) | (.022) | (.008) | (.022) | (.024) | (.521) | (.110) | (.008) |
| High school graduate | -.041 | -.070 | -.042 | -.082 | -.100 | -.131 | -.085 | .009 | -.228 |
|  | .144 | .067 | .125 | .122 | .103 | .107 | .120 | .126 | .114 |
|  | [-.324, .241] | [-.201, .060] | [-.288, .204] | [-.321, .157] | [-.302, .102] | [-.342, .079] | [-.320, .151] | [-.238, .256] | [-.451, -.005] |
|  | (.774) | (.291) | (.738) | (.501) | (.333) | (.221) | (.481) | (.943) | (.045) |
| Some college | -.077 | -.007 | .203 | -.037 | .004 | -.114 | -.081 | -.025 | -.328 |
|  | .144 | .068 | .127 | .124 | .105 | .109 | .122 | .128 | .115 |
|  | [-.359, .205] | [-.140, .126] | [-.046, .452] | [-.280, .205] | [-.202, .209] | [-.328, .101] | [-.321, .159] | [-.276, .227] | [-.555, -.102] |
|  | (.593) | (.916) | (.110) | (.762) | (.973) | (.299) | (.507) | (.848) | (.004) |
| 2-year | -.011 | .004 | .121 | -.035 | .001 | -.105 | -.120 | -.032 | -.362 |
|  | .150 | .070 | .134 | .128 | .109 | .115 | .128 | .134 | .122 |
|  | [-.306, .283] | [-.133, .140] | [-.142, .384] | [-.286, .216] | [-.213, .216] | [-.330, .120] | [-.370, .131] | [-.295, .232] | [-.601, -.124] |
|  | (.939) | (.958) | (.367) | (.782) | (.992) | (.360) | (.350) | (.812) | (.003) |
| 4-year | -.059 | .006 | .100 | -.083 | .025 | -.085 | -.066 | -.069 | -.337 |
|  | .146 | .068 | .130 | .126 | .107 | .112 | .123 | .130 | .118 |
|  | [-.345, .226] | [-.128, .140] | [-.155, .355] | [-.329, .164] | [-.184, .235] | [-.304, .133] | [-.308, .176] | [-.325, .186] | [-.568, -.106] |
|  | (.683) | (.930) | (.441) | (.510) | (.813) | (.444) | (.592) | (.595) | (.004) |
| Post-grad | -.056 | .025 | .151 | .021 | .102 | -.017 | -.023 | -.007 | -.431 |
|  | .152 | .069 | .137 | .131 | .111 | .116 | .129 | .137 | .123 |
|  | [-.354, .241] | [-.110, .161] | [-.117, .420] | [-.235, .277] | [-.114, .319] | [-.243, .210] | [-.275, .230] | [-.275, .261] | [-.673, -.189] |
|  | (.711) | (.714) | (.270) | (.872) | (.355) | (.886) | (.860) | (.960) | (<.001) |
| income=2 | .065 | -.039 | .371 | -.068 | .010 | .004 | -.158 | -.152 | -.112 |
|  | .134 | .057 | .123 | .112 | .104 | .109 | .115 | .116 | .117 |
|  | [-.196, .327] | [-.150, .072] | [.130, .613] | [-.287, .151] | [-.194, .215] | [-.210, .219] | [-.382, .067] | [-.379, .075] | [-.342, .117] |
|  | (.624) | (.493) | (.003) | (.542) | (.921) | (.969) | (.169) | (.188) | (.338) |
| income=3 | .172 | -.004 | .306 | -.095 | .115 | -.010 | -.112 | -.042 | -.147 |
|  | .129 | .055 | .120 | .108 | .101 | .107 | .109 | .115 | .113 |
|  | [-.081, .426] | [-.112, .105] | [.071, .542] | [-.306, .117] | [-.083, .312] | [-.219, .199] | [-.326, .103] | [-.269, .184] | [-.369, .074] |
|  | (.183) | (.949) | (.011) | (.380) | (.255) | (.925) | (.307) | (.715) | (.191) |
| income=4 | .123 | .025 | .295 | .012 | .176 | .094 | -.085 | -.046 | -.118 |
|  | .130 | .057 | .128 | .109 | .103 | .107 | .113 | .116 | .115 |
|  | [-.131, .378] | [-.087, .137] | [.044, .546] | [-.201, .225] | [-.026, .379] | [-.116, .305] | [-.306, .136] | [-.273, .181] | [-.343, .107] |
|  | (.343) | (.661) | (.021) | (.912) | (.088) | (.379) | (.449) | (.689) | (.304) |
| income=5 | .181 | .033 | .377 | -.055 | .147 | .015 | -.157 | -.033 | -.194 |
|  | .131 | .057 | .127 | .111 | .104 | .108 | .112 | .114 | .114 |
|  | [-.076, .437] | [-.079, .144] | [.129, .626] | [-.273, .164] | [-.057, .352] | [-.197, .227] | [-.377, .063] | [-.257, .191] | [-.418, .030] |
|  | (.168) | (.565) | (.003) | (.624) | (.158) | (.890) | (.162) | (.773) | (.089) |
| income=6 | .217 | .001 | .378 | .018 | .136 | .015 | -.087 | .089 | -.142 |
|  | .134 | .059 | .126 | .112 | .106 | .110 | .116 | .119 | .118 |
|  | [-.045, .480] | [-.115, .117] | [.130, .626] | [-.202, .237] | [-.073, .345] | [-.201, .231] | [-.314, .139] | [-.144, .322] | [-.374, .090] |
|  | (.105) | (.987) | (.003) | (.874) | (.201) | (.892) | (.450) | (.454) | (.229) |
| income=7 | .080 | .002 | .396 | -.078 | .069 | -.018 | -.183 | -.099 | -.287 |
|  | .139 | .061 | .132 | .118 | .111 | .115 | .123 | .130 | .124 |
|  | [-.193, .353] | [-.117, .121] | [.137, .656] | [-.310, .154] | [-.150, .288] | [-.243, .207] | [-.424, .058] | [-.354, .157] | [-.530, -.043] |
|  | (.565) | (.972) | (.003) | (.511) | (.536) | (.879) | (.136) | (.448) | (.021) |
| income=8 | .119 | .026 | .370 | -.258 | .032 | -.210 | -.256 | -.173 | -.361 |
|  | .136 | .059 | .131 | .117 | .109 | .113 | .120 | .125 | .122 |
|  | [-.147, .385] | [-.091, .142] | [.114, .626] | [-.488, -.028] | [-.181, .245] | [-.431, .011] | [-.490, -.021] | [-.418, .071] | [-.599, -.122] |
|  | (.380) | (.667) | (.005) | (.028) | (.768) | (.063) | (.033) | (.165) | (.003) |
| income=9 | .352 | .016 | .481 | -.178 | .143 | -.057 | -.075 | -.105 | -.301 |
|  | .135 | .058 | .131 | .114 | .106 | .112 | .116 | .124 | .117 |
|  | [.087, .616] | [-.098, .129] | [.225, .738] | [-.401, .046] | [-.065, .351] | [-.276, .162] | [-.303, .154] | [-.348, .138] | [-.531, -.071] |
|  | (.009) | (.787) | (<.001) | (.119) | (.177) | (.612) | (.521) | (.397) | (.010) |
| income=10 | .223 | .012 | .503 | -.171 | .193 | -.067 | -.095 | -.105 | -.368 |
|  | .134 | .061 | .135 | .117 | .112 | .115 | .120 | .125 | .125 |
|  | [-.039, .485] | [-.107, .130] | [.237, .768] | [-.399, .058] | [-.025, .412] | [-.293, .159] | [-.330, .140] | [-.351, .140] | [-.612, -.123] |
|  | (.096) | (.845) | (<.001) | (.143) | (.083) | (.561) | (.429) | (.400) | (.003) |
| income=11 | .371 | .008 | .482 | -.067 | .331 | .008 | .079 | -.096 | -.347 |
|  | .139 | .061 | .137 | .122 | .112 | .121 | .124 | .130 | .127 |
|  | [.098, .643] | [-.111, .127] | [.213, .751] | [-.307, .173] | [.111, .551] | [-.230, .245] | [-.164, .321] | [-.350, .158] | [-.596, -.098] |
|  | (.008) | (.896) | (<.001) | (.583) | (.003) | (.950) | (.524) | (.459) | (.006) |
| income=12 | .235 | .038 | .373 | -.135 | .231 | .010 | .026 | -.181 | -.419 |
|  | .148 | .063 | .163 | .134 | .119 | .138 | .138 | .149 | .143 |
|  | [-.055, .525] | [-.086, .162] | [.054, .693] | [-.397, .127] | [-.003, .465] | [-.261, .281] | [-.244, .295] | [-.474, .111] | [-.700, -.138] |
|  | (.112) | (.544) | (.022) | (.312) | (.053) | (.942) | (.853) | (.224) | (.004) |
| income=13 | .214 | .065 | .643 | -.258 | .028 | -.123 | -.082 | -.419 | -.647 |
|  | .212 | .088 | .189 | .165 | .178 | .166 | .177 | .214 | .178 |
|  | [-.203, .630] | [-.107, .238] | [.273, 1.013] | [-.582, .065] | [-.321, .378] | [-.449, .202] | [-.429, .265] | [-.838, -.000] | [-.996, -.297] |
|  | (.315) | (.458) | (.001) | (.117) | (.873) | (.458) | (.642) | (.050) | (<.001) |
| income=14 | .306 | -.069 | .542 | -.225 | .082 | -.066 | -.184 | -.068 | -.436 |
|  | .205 | .088 | .167 | .210 | .186 | .209 | .224 | .193 | .193 |
|  | [-.096, .709] | [-.242, .104] | [.215, .869] | [-.636, .187] | [-.282, .447] | [-.476, .344] | [-.623, .254] | [-.446, .310] | [-.814, -.057] |
|  | (.136) | (.434) | (.001) | (.284) | (.657) | (.753) | (.410) | (.724) | (.024) |
| income=15 | .278 | .198 | 1.351 | .403 | .670 | .186 | .350 | -.078 | -1.188 |
|  | .782 | .107 | .249 | .543 | .189 | .698 | .455 | .642 | .425 |
|  | [-1.255, 1.812] | [-.012, .408] | [.863, 1.840] | [-.662, 1.468] | [.299, 1.042] | [-1.182, 1.553] | [-.543, 1.243] | [-1.336, 1.180] | [-2.020, -.355] |
|  | (.722) | (.064) | (<.001) | (.458) | (<.001) | (.790) | (.443) | (.903) | (.005) |
| income=16 | .526 | .063 | .901 | -.278 | .127 | -.056 | .184 | -.029 | -.459 |
|  | .262 | .126 | .345 | .313 | .267 | .324 | .272 | .305 | .270 |
|  | [.012, 1.039] | [-.184, .310] | [.225, 1.577] | [-.891, .335] | [-.397, .650] | [-.692, .580] | [-.348, .717] | [-.627, .569] | [-.988, .070] |
|  | (.045) | (.618) | (.009) | (.374) | (.635) | (.863) | (.498) | (.925) | (.089) |
| income=17 | .274 | -.024 | .365 | -.225 | .024 | -.120 | -.221 | -.260 | -.413 |
|  | .126 | .055 | .117 | .105 | .101 | .103 | .107 | .111 | .109 |
|  | [.027, .520] | [-.132, .084] | [.135, .594] | [-.430, -.019] | [-.173, .222] | [-.321, .082] | [-.432, -.011] | [-.477, -.042] | [-.626, -.199] |
|  | (.029) | (.664) | (.002) | (.032) | (.809) | (.245) | (.039) | (.019) | (<.001) |
| Rural Code=2 | .006 | -.011 | -.042 | -.000 | -.055 | -.055 | -.037 | -.098 | -.011 |
|  | .055 | .025 | .053 | .051 | .046 | .049 | .051 | .054 | .051 |
|  | [-.101, .113] | [-.060, .039] | [-.147, .063] | [-.101, .100] | [-.144, .035] | [-.150, .041] | [-.136, .063] | [-.203, .007] | [-.111, .089] |
|  | (.911) | (.679) | (.432) | (.994) | (.234) | (.261) | (.470) | (.068) | (.832) |
| Rural Code=3 | -.009 | -.025 | -.098 | .024 | -.038 | .050 | .020 | .023 | .052 |
|  | .075 | .034 | .073 | .068 | .063 | .065 | .067 | .073 | .064 |
|  | [-.156, .139] | [-.092, .042] | [-.242, .046] | [-.109, .157] | [-.161, .085] | [-.077, .177] | [-.113, .152] | [-.121, .166] | [-.075, .178] |
|  | (.908) | (.468) | (.181) | (.728) | (.545) | (.437) | (.771) | (.754) | (.423) |
| Rural Code=4 | -.082 | .018 | .077 | -.133 | -.219 | -.170 | -.137 | -.092 | -.045 |
|  | .106 | .047 | .102 | .091 | .087 | .093 | .095 | .104 | .094 |
|  | [-.290, .126] | [-.075, .111] | [-.122, .277] | [-.312, .046] | [-.389, -.049] | [-.352, .011] | [-.323, .049] | [-.296, .113] | [-.229, .139] |
|  | (.440) | (.703) | (.447) | (.145) | (.012) | (.066) | (.148) | (.378) | (.632) |
| Rural Code=5 | -.171 | -.011 | -.030 | -.157 | -.034 | .033 | -.094 | -.001 | -.022 |
|  | .173 | .065 | .138 | .137 | .136 | .130 | .134 | .158 | .139 |
|  | [-.511, .169] | [-.138, .116] | [-.301, .241] | [-.427, .112] | [-.301, .232] | [-.222, .288] | [-.357, .168] | [-.311, .308] | [-.294, .251] |
|  | (.324) | (.866) | (.828) | (.253) | (.800) | (.800) | (.481) | (.993) | (.875) |
| Rural Code=6 | .126 | -.007 | -.028 | .013 | .021 | -.060 | .004 | .013 | .109 |
|  | .114 | .048 | .102 | .097 | .080 | .096 | .098 | .105 | .098 |
|  | [-.097, .349] | [-.102, .087] | [-.229, .172] | [-.177, .203] | [-.135, .177] | [-.248, .128] | [-.188, .196] | [-.194, .219] | [-.084, .301] |
|  | (.267) | (.879) | (.781) | (.897) | (.793) | (.529) | (.968) | (.903) | (.268) |
| Rural Code=7 | .053 | -.040 | .074 | .064 | -.105 | .060 | .045 | -.033 | .069 |
|  | .143 | .067 | .146 | .126 | .119 | .130 | .133 | .145 | .121 |
|  | [-.227, .333] | [-.171, .091] | [-.211, .360] | [-.183, .312] | [-.338, .127] | [-.194, .314] | [-.216, .307] | [-.317, .251] | [-.168, .305] |
|  | (.713) | (.547) | (.610) | (.609) | (.375) | (.645) | (.733) | (.821) | (.569) |
| Rural Code=8 | .499 | -.004 | -.157 | -.401 | .008 | -.188 | -.073 | -.401 | .108 |
|  | .265 | .124 | .247 | .247 | .197 | .234 | .226 | .253 | .172 |
|  | [-.021, 1.018] | [-.246, .239] | [-.642, .327] | [-.886, .084] | [-.378, .394] | [-.647, .271] | [-.516, .370] | [-.896, .095] | [-.230, .445] |
|  | (.060) | (.977) | (.524) | (.105) | (.969) | (.422) | (.746) | (.113) | (.531) |
| Rural Code=9 | -.126 | -.067 | -.031 | .206 | -.121 | .211 | -.048 | -.087 | -.196 |
|  | .200 | .115 | .200 | .198 | .179 | .182 | .190 | .213 | .224 |
|  | [-.519, .267] | [-.292, .159] | [-.423, .361] | [-.182, .595] | [-.472, .229] | [-.146, .568] | [-.421, .326] | [-.504, .330] | [-.635, .243] |
|  | (.529) | (.561) | (.876) | (.298) | (.497) | (.247) | (.803) | (.682) | (.381) |
| Alaska | .254 | -.034 | .318 | -.024 | -.128 | -.479 | -.517 | .036 | -.280 |
|  | .456 | .259 | .402 | .324 | .300 | .261 | .399 | .435 | .303 |
|  | [-.639, 1.147] | [-.542, .474] | [-.470, 1.107] | [-.659, .611] | [-.717, .461] | [-.992, .034] | [-1.299, .264] | [-.816, .889] | [-.873, .314] |
|  | (.577) | (.896) | (.429) | (.940) | (.670) | (.067) | (.194) | (.933) | (.356) |
| Arizona | -.204 | .071 | .158 | -.028 | -.164 | -.192 | -.125 | -.174 | .040 |
|  | .220 | .100 | .220 | .201 | .169 | .189 | .206 | .195 | .188 |
|  | [-.636, .228] | [-.125, .266] | [-.274, .590] | [-.421, .365] | [-.495, .167] | [-.563, .179] | [-.529, .279] | [-.557, .209] | [-.329, .409] |
|  | (.354) | (.478) | (.474) | (.889) | (.331) | (.310) | (.543) | (.373) | (.831) |
| Arkansas | .129 | .032 | -.034 | .097 | -.267 | -.035 | .026 | .017 | .263 |
|  | .251 | .119 | .255 | .213 | .199 | .203 | .232 | .221 | .210 |
|  | [-.364, .621] | [-.202, .266] | [-.534, .466] | [-.320, .514] | [-.656, .123] | [-.433, .362] | [-.428, .481] | [-.415, .450] | [-.148, .675] |
|  | (.609) | (.788) | (.894) | (.649) | (.180) | (.861) | (.909) | (.938) | (.210) |
| California | -.008 | .081 | -.019 | .176 | -.148 | -.052 | -.028 | .006 | .134 |
|  | .203 | .090 | .201 | .174 | .142 | .166 | .184 | .173 | .166 |
|  | [-.407, .390] | [-.095, .256] | [-.414, .376] | [-.166, .518] | [-.426, .129] | [-.378, .274] | [-.388, .333] | [-.332, .344] | [-.192, .459] |
|  | (.967) | (.369) | (.925) | (.313) | (.295) | (.755) | (.881) | (.973) | (.421) |
| Colorado | .205 | .005 | -.032 | .166 | -.370 | -.141 | -.205 | -.063 | -.156 |
|  | .251 | .121 | .259 | .222 | .194 | .208 | .233 | .209 | .214 |
|  | [-.287, .697] | [-.232, .242] | [-.541, .476] | [-.270, .601] | [-.750, .010] | [-.548, .267] | [-.661, .251] | [-.473, .347] | [-.577, .264] |
|  | (.414) | (.967) | (.901) | (.455) | (.056) | (.499) | (.379) | (.763) | (.467) |
| Connecticut | .021 | .261 | .238 | .221 | -.039 | -.035 | .116 | .007 | .020 |
|  | .260 | .116 | .259 | .237 | .208 | .208 | .261 | .244 | .237 |
|  | [-.490, .531] | [.034, .488] | [-.269, .745] | [-.245, .686] | [-.446, .368] | [-.442, .373] | [-.397, .628] | [-.472, .485] | [-.445, .484] |
|  | (.937) | (.024) | (.358) | (.353) | (.851) | (.868) | (.658) | (.979) | (.933) |
| Delaware | .344 | -.048 | -.309 | .142 | -.131 | -.376 | -.167 | .002 | -.327 |
|  | .323 | .152 | .314 | .283 | .228 | .290 | .276 | .315 | .284 |
|  | [-.288, .977] | [-.346, .250] | [-.924, .307] | [-.414, .698] | [-.579, .316] | [-.945, .194] | [-.709, .374] | [-.616, .620] | [-.884, .229] |
|  | (.286) | (.751) | (.326) | (.616) | (.565) | (.196) | (.544) | (.995) | (.249) |
| District of Columbia | .165 | .125 | .251 | .394 | -.133 | -.032 | .064 | -.202 | .434 |
|  | .548 | .140 | .355 | .281 | .306 | .220 | .371 | .281 | .320 |
|  | [-.910, 1.240] | [-.150, .399] | [-.444, .946] | [-.157, .944] | [-.733, .467] | [-.465, .400] | [-.665, .792] | [-.753, .348] | [-.194, 1.062] |
|  | (.763) | (.373) | (.479) | (.161) | (.664) | (.883) | (.864) | (.471) | (.176) |
| Florida | .171 | .061 | .056 | .113 | -.224 | -.162 | -.010 | .013 | .102 |
|  | .204 | .090 | .202 | .176 | .146 | .166 | .185 | .178 | .168 |
|  | [-.230, .571] | [-.115, .238] | [-.341, .453] | [-.232, .457] | [-.511, .062] | [-.487, .164] | [-.374, .354] | [-.335, .361] | [-.228, .431] |
|  | (.403) | (.495) | (.783) | (.522) | (.124) | (.331) | (.958) | (.940) | (.546) |
| Georgia | .044 | .060 | .154 | -.029 | -.328 | -.185 | .062 | -.164 | .000 |
|  | .230 | .099 | .214 | .196 | .163 | .185 | .201 | .193 | .183 |
|  | [-.407, .495] | [-.134, .254] | [-.266, .574] | [-.413, .355] | [-.648, -.009] | [-.548, .177] | [-.332, .455] | [-.542, .215] | [-.358, .359] |
|  | (.848) | (.543) | (.471) | (.882) | (.044) | (.317) | (.759) | (.397) | (.998) |
| Hawaii | .020 | .282 | .508 | -.034 | .066 | -.432 | .115 | -.136 | .366 |
|  | .379 | .094 | .372 | .377 | .275 | .284 | .347 | .378 | .351 |
|  | [-.722, .762] | [.098, .465] | [-.222, 1.238] | [-.774, .706] | [-.473, .605] | [-.988, .125] | [-.565, .794] | [-.878, .606] | [-.321, 1.054] |
|  | (.958) | (.003) | (.173) | (.928) | (.810) | (.129) | (.740) | (.719) | (.297) |
| Idaho | .130 | -.121 | -.185 | -.375 | -.200 | -.523 | -.349 | -.193 | -.031 |
|  | .293 | .178 | .347 | .298 | .231 | .280 | .258 | .298 | .267 |
|  | [-.445, .705] | [-.470, .227] | [-.866, .496] | [-.958, .209] | [-.654, .253] | [-1.072, .026] | [-.854, .156] | [-.777, .391] | [-.556, .493] |
|  | (.658) | (.495) | (.595) | (.208) | (.387) | (.062) | (.175) | (.517) | (.907) |
| Illinois | .044 | .105 | .021 | .317 | -.282 | -.140 | -.048 | -.060 | .071 |
|  | .216 | .095 | .213 | .186 | .161 | .182 | .198 | .192 | .178 |
|  | [-.380, .467] | [-.081, .290] | [-.396, .439] | [-.048, .682] | [-.597, .033] | [-.497, .216] | [-.436, .341] | [-.437, .317] | [-.277, .419] |
|  | (.839) | (.268) | (.920) | (.089) | (.079) | (.440) | (.809) | (.757) | (.690) |
| Indiana | -.180 | .072 | .329 | .300 | -.195 | -.032 | -.019 | -.064 | .033 |
|  | .225 | .098 | .228 | .202 | .169 | .196 | .214 | .216 | .197 |
|  | [-.621, .261] | [-.120, .265] | [-.118, .777] | [-.097, .696] | [-.527, .137] | [-.416, .352] | [-.439, .401] | [-.488, .360] | [-.352, .419] |
|  | (.424) | (.461) | (.149) | (.138) | (.250) | (.871) | (.929) | (.768) | (.865) |
| Iowa | .072 | -.029 | -.080 | .434 | -.017 | -.059 | -.047 | .062 | -.011 |
|  | .290 | .121 | .279 | .230 | .194 | .225 | .239 | .269 | .231 |
|  | [-.497, .640] | [-.266, .208] | [-.627, .467] | [-.017, .885] | [-.397, .363] | [-.500, .382] | [-.517, .422] | [-.466, .591] | [-.465, .443] |
|  | (.805) | (.813) | (.775) | (.059) | (.931) | (.792) | (.843) | (.817) | (.962) |
| Kansas | .088 | .117 | .141 | .226 | .154 | -.051 | .158 | .318 | .053 |
|  | .305 | .166 | .337 | .315 | .245 | .301 | .306 | .313 | .273 |
|  | [-.509, .686] | [-.209, .443] | [-.520, .801] | [-.392, .844] | [-.327, .635] | [-.641, .539] | [-.443, .759] | [-.296, .933] | [-.481, .588] |
|  | (.772) | (.480) | (.676) | (.473) | (.531) | (.865) | (.606) | (.310) | (.845) |
| Kentucky | -.218 | -.056 | -.052 | .097 | .051 | -.236 | -.020 | -.109 | -.070 |
|  | .245 | .121 | .242 | .231 | .179 | .205 | .230 | .235 | .204 |
|  | [-.698, .263] | [-.294, .182] | [-.527, .422] | [-.356, .550] | [-.299, .402] | [-.637, .165] | [-.472, .432] | [-.570, .352] | [-.470, .331] |
|  | (.375) | (.643) | (.829) | (.675) | (.773) | (.249) | (.930) | (.642) | (.733) |
| Louisiana | .474 | .030 | -.057 | -.083 | -.209 | -.333 | .014 | -.013 | .072 |
|  | .282 | .121 | .259 | .249 | .218 | .231 | .243 | .238 | .225 |
|  | [-.078, 1.026] | [-.207, .267] | [-.564, .450] | [-.571, .405] | [-.636, .219] | [-.786, .119] | [-.463, .491] | [-.481, .454] | [-.369, .512] |
|  | (.093) | (.804) | (.826) | (.738) | (.339) | (.149) | (.955) | (.956) | (.749) |
| Maine | -.315 | .134 | -.367 | .178 | -.167 | -.139 | -.330 | -.508 | -.783 |
|  | .278 | .145 | .369 | .314 | .238 | .270 | .336 | .296 | .264 |
|  | [-.859, .229] | [-.151, .419] | [-1.091, .357] | [-.438, .794] | [-.634, .301] | [-.668, .391] | [-.989, .329] | [-1.089, .073] | [-1.300, -.266] |
|  | (.257) | (.356) | (.321) | (.571) | (.484) | (.608) | (.327) | (.087) | (.003) |
| Maryland | -.085 | .136 | -.101 | .103 | -.097 | .048 | -.050 | .041 | -.044 |
|  | .237 | .110 | .269 | .228 | .179 | .219 | .230 | .231 | .223 |
|  | [-.550, .381] | [-.079, .351] | [-.628, .426] | [-.344, .549] | [-.449, .254] | [-.382, .479] | [-.501, .401] | [-.412, .494] | [-.482, .394] |
|  | (.722) | (.216) | (.707) | (.652) | (.588) | (.825) | (.828) | (.859) | (.844) |
| Massachusetts | -.005 | .060 | .083 | .380 | -.172 | .182 | .147 | .094 | .214 |
|  | .247 | .103 | .228 | .212 | .180 | .199 | .213 | .222 | .196 |
|  | [-.489, .480] | [-.142, .262] | [-.365, .531] | [-.036, .796] | [-.524, .180] | [-.209, .573] | [-.270, .565] | [-.342, .530] | [-.170, .597] |
|  | (.984) | (.559) | (.716) | (.074) | (.339) | (.360) | (.489) | (.673) | (.275) |
| Michigan | -.012 | .094 | .057 | .203 | -.152 | -.148 | -.034 | -.119 | -.042 |
|  | .214 | .096 | .214 | .186 | .152 | .178 | .196 | .190 | .180 |
|  | [-.432, .408] | [-.094, .282] | [-.363, .476] | [-.161, .568] | [-.450, .146] | [-.497, .201] | [-.418, .350] | [-.491, .253] | [-.395, .310] |
|  | (.955) | (.328) | (.791) | (.274) | (.317) | (.405) | (.861) | (.530) | (.814) |
| Minnesota | .071 | .041 | .527 | .133 | -.076 | -.185 | .021 | -.024 | -.079 |
|  | .251 | .107 | .232 | .221 | .177 | .209 | .231 | .223 | .208 |
|  | [-.420, .562] | [-.169, .251] | [.072, .982] | [-.301, .566] | [-.423, .272] | [-.595, .225] | [-.431, .473] | [-.462, .413] | [-.487, .328] |
|  | (.777) | (.700) | (.023) | (.549) | (.670) | (.376) | (.929) | (.913) | (.704) |
| Mississippi | .409 | -.084 | -.055 | .237 | -.300 | -.205 | -.154 | -.410 | -.028 |
|  | .285 | .135 | .270 | .264 | .236 | .230 | .259 | .271 | .243 |
|  | [-.150, .969] | [-.348, .179] | [-.585, .474] | [-.281, .755] | [-.762, .162] | [-.657, .246] | [-.662, .355] | [-.941, .122] | [-.505, .449] |
|  | (.152) | (.531) | (.838) | (.370) | (.203) | (.373) | (.553) | (.131) | (.908) |
| Missouri | -.124 | .047 | -.050 | -.025 | -.249 | -.176 | -.084 | -.080 | -.047 |
|  | .236 | .103 | .225 | .200 | .164 | .190 | .205 | .199 | .184 |
|  | [-.587, .340] | [-.155, .249] | [-.491, .391] | [-.417, .367] | [-.571, .073] | [-.548, .197] | [-.485, .317] | [-.471, .310] | [-.407, .312] |
|  | (.601) | (.648) | (.826) | (.902) | (.130) | (.354) | (.682) | (.687) | (.796) |
| Montana | -.208 | .046 | -.105 | .441 | .182 | -.249 | .201 | -.150 | .054 |
|  | .337 | .180 | .374 | .262 | .232 | .301 | .366 | .342 | .280 |
|  | [-.869, .453] | [-.307, .399] | [-.838, .629] | [-.072, .954] | [-.274, .637] | [-.838, .341] | [-.516, .918] | [-.821, .520] | [-.495, .603] |
|  | (.537) | (.800) | (.780) | (.092) | (.434) | (.408) | (.583) | (.660) | (.847) |
| Nebraska | .065 | -.125 | .207 | .215 | -.066 | -.270 | -.092 | .303 | .047 |
|  | .315 | .204 | .281 | .366 | .274 | .285 | .344 | .326 | .364 |
|  | [-.554, .683] | [-.524, .274] | [-.344, .757] | [-.502, .932] | [-.604, .472] | [-.829, .290] | [-.766, .581] | [-.337, .943] | [-.667, .760] |
|  | (.837) | (.540) | (.462) | (.556) | (.811) | (.344) | (.788) | (.354) | (.898) |
| Nevada | .097 | .022 | .168 | .242 | -.083 | .093 | -.044 | -.107 | .069 |
|  | .245 | .108 | .245 | .220 | .201 | .210 | .240 | .236 | .218 |
|  | [-.384, .578] | [-.189, .234] | [-.313, .649] | [-.190, .674] | [-.477, .311] | [-.320, .505] | [-.516, .427] | [-.570, .355] | [-.358, .496] |
|  | (.693) | (.837) | (.493) | (.272) | (.680) | (.660) | (.854) | (.650) | (.751) |
| New Hampshire | .290 | -.062 | .007 | .047 | -.442 | -.150 | -.345 | -.194 | .050 |
|  | .301 | .160 | .280 | .276 | .242 | .235 | .268 | .302 | .258 |
|  | [-.301, .881] | [-.374, .251] | [-.542, .556] | [-.494, .589] | [-.917, .033] | [-.612, .311] | [-.870, .181] | [-.786, .399] | [-.457, .557] |
|  | (.335) | (.699) | (.980) | (.864) | (.068) | (.523) | (.198) | (.522) | (.846) |
| New Jersey | .304 | .080 | -.199 | .287 | -.281 | .043 | .126 | .027 | .243 |
|  | .237 | .101 | .232 | .209 | .184 | .200 | .216 | .201 | .207 |
|  | [-.161, .768] | [-.119, .278] | [-.653, .255] | [-.124, .697] | [-.641, .079] | [-.350, .436] | [-.298, .550] | [-.366, .421] | [-.163, .648] |
|  | (.200) | (.431) | (.391) | (.171) | (.126) | (.830) | (.559) | (.892) | (.241) |
| New Mexico | .264 | .060 | -.221 | .101 | -.024 | -.202 | -.091 | .163 | -.034 |
|  | .274 | .118 | .295 | .250 | .198 | .229 | .236 | .267 | .229 |
|  | [-.274, .803] | [-.171, .291] | [-.798, .357] | [-.390, .592] | [-.411, .364] | [-.652, .247] | [-.553, .371] | [-.360, .686] | [-.482, .414] |
|  | (.336) | (.613) | (.454) | (.687) | (.904) | (.378) | (.699) | (.541) | (.881) |
| New York | .218 | .060 | -.183 | .337 | -.156 | .045 | -.008 | -.010 | .187 |
|  | .212 | .091 | .210 | .179 | .147 | .172 | .186 | .180 | .168 |
|  | [-.199, .634] | [-.118, .239] | [-.594, .228] | [-.013, .687] | [-.445, .132] | [-.292, .382] | [-.373, .356] | [-.362, .343] | [-.142, .516] |
|  | (.306) | (.507) | (.382) | (.059) | (.288) | (.792) | (.964) | (.957) | (.266) |
| North Carolina | -.140 | .095 | .023 | .228 | -.084 | -.101 | .027 | .144 | .112 |
|  | .234 | .097 | .214 | .195 | .161 | .188 | .202 | .200 | .190 |
|  | [-.599, .320] | [-.095, .285] | [-.398, .443] | [-.156, .611] | [-.400, .233] | [-.470, .268] | [-.368, .423] | [-.249, .537] | [-.260, .484] |
|  | (.551) | (.328) | (.916) | (.244) | (.604) | (.591) | (.892) | (.473) | (.556) |
| North Dakota | .098 | .120 | .121 | .030 | -.201 | -.442 | .265 | -.356 | -.200 |
|  | .392 | .159 | .455 | .341 | .272 | .302 | .339 | .465 | .410 |
|  | [-.671, .867] | [-.191, .431] | [-.772, 1.014] | [-.639, .698] | [-.735, .333] | [-1.033, .150] | [-.399, .929] | [-1.269, .556] | [-1.005, .605] |
|  | (.803) | (.449) | (.790) | (.931) | (.461) | (.143) | (.434) | (.444) | (.626) |
| Ohio | .018 | .076 | .025 | .147 | -.108 | -.080 | -.039 | -.252 | -.082 |
|  | .215 | .094 | .211 | .189 | .153 | .179 | .196 | .187 | .178 |
|  | [-.404, .440] | [-.108, .261] | [-.388, .439] | [-.223, .517] | [-.409, .192] | [-.432, .272] | [-.423, .344] | [-.620, .115] | [-.430, .267] |
|  | (.932) | (.418) | (.904) | (.435) | (.480) | (.655) | (.840) | (.179) | (.645) |
| Oklahoma | -.072 | .017 | .317 | .015 | -.148 | -.057 | -.053 | -.120 | -.142 |
|  | .304 | .152 | .290 | .261 | .236 | .238 | .267 | .251 | .266 |
|  | [-.667, .524] | [-.282, .316] | [-.252, .885] | [-.497, .527] | [-.611, .315] | [-.523, .409] | [-.577, .471] | [-.612, .372] | [-.663, .379] |
|  | (.813) | (.912) | (.275) | (.955) | (.530) | (.810) | (.844) | (.631) | (.592) |
| Oregon | -.177 | .101 | .409 | .135 | .085 | -.143 | .126 | -.061 | .137 |
|  | .225 | .101 | .215 | .206 | .163 | .193 | .213 | .206 | .204 |
|  | [-.618, .264] | [-.097, .298] | [-.011, .830] | [-.270, .539] | [-.234, .405] | [-.521, .235] | [-.291, .543] | [-.465, .344] | [-.262, .537] |
|  | (.431) | (.316) | (.056) | (.514) | (.600) | (.458) | (.554) | (.769) | (.499) |
| Pennsylvania | .014 | .086 | .115 | .123 | -.243 | -.198 | -.048 | -.035 | .006 |
|  | .205 | .091 | .198 | .178 | .149 | .168 | .188 | .179 | .171 |
|  | [-.387, .415] | [-.092, .265] | [-.273, .503] | [-.226, .473] | [-.535, .049] | [-.528, .132] | [-.417, .320] | [-.385, .315] | [-.328, .341] |
|  | (.944) | (.343) | (.561) | (.489) | (.103) | (.239) | (.797) | (.845) | (.971) |
| Rhode Island | .054 | .156 | -.168 | .462 | -.108 | .034 | .136 | -.012 | -.024 |
|  | .407 | .159 | .350 | .381 | .301 | .396 | .356 | .379 | .360 |
|  | [-.743, .852] | [-.156, .468] | [-.855, .520] | [-.285, 1.209] | [-.699, .483] | [-.743, .811] | [-.561, .833] | [-.754, .731] | [-.729, .681] |
|  | (.894) | (.328) | (.632) | (.225) | (.721) | (.932) | (.702) | (.976) | (.946) |
| South Carolina | .061 | .043 | -.024 | .060 | .004 | -.137 | .140 | .021 | -.002 |
|  | .252 | .112 | .242 | .213 | .173 | .209 | .226 | .224 | .206 |
|  | [-.433, .555] | [-.178, .263] | [-.500, .451] | [-.358, .477] | [-.335, .343] | [-.546, .273] | [-.303, .582] | [-.419, .462] | [-.407, .403] |
|  | (.809) | (.705) | (.920) | (.779) | (.981) | (.513) | (.536) | (.924) | (.993) |
| South Dakota | .208 | .049 | .225 | -.713 | .009 | -.749 | -.104 | -.048 | -.383 |
|  | .317 | .181 | .321 | .265 | .273 | .311 | .348 | .386 | .310 |
|  | [-.413, .829] | [-.306, .403] | [-.404, .854] | [-1.233, -.193] | [-.527, .544] | [-1.359, -.140] | [-.787, .579] | [-.804, .709] | [-.991, .224] |
|  | (.512) | (.788) | (.484) | (.007) | (.975) | (.016) | (.765) | (.902) | (.216) |
| Tennessee | .077 | .118 | .169 | .350 | -.107 | .150 | .165 | -.176 | .073 |
|  | .252 | .107 | .217 | .216 | .172 | .199 | .215 | .215 | .210 |
|  | [-.416, .571] | [-.093, .329] | [-.255, .594] | [-.072, .773] | [-.445, .231] | [-.240, .540] | [-.257, .587] | [-.598, .247] | [-.339, .485] |
|  | (.759) | (.272) | (.435) | (.104) | (.536) | (.449) | (.444) | (.414) | (.727) |
| Texas | -.201 | .077 | .041 | .065 | -.186 | -.272 | -.035 | .010 | .085 |
|  | .210 | .092 | .205 | .181 | .148 | .173 | .189 | .179 | .172 |
|  | [-.612, .210] | [-.104, .258] | [-.361, .442] | [-.291, .420] | [-.476, .104] | [-.611, .067] | [-.407, .336] | [-.342, .361] | [-.252, .421] |
|  | (.337) | (.403) | (.843) | (.722) | (.208) | (.116) | (.852) | (.957) | (.622) |
| Utah | -.106 | .054 | .385 | .304 | -.153 | -.106 | -.204 | .159 | .107 |
|  | .292 | .140 | .252 | .258 | .203 | .244 | .261 | .285 | .247 |
|  | [-.678, .466] | [-.221, .329] | [-.110, .880] | [-.202, .810] | [-.551, .246] | [-.583, .372] | [-.716, .308] | [-.400, .719] | [-.377, .591] |
|  | (.715) | (.700) | (.128) | (.239) | (.452) | (.665) | (.435) | (.576) | (.665) |
| Vermont | .619 | .088 | -.023 | -.064 | -.237 | -.431 | -.149 | .152 | -.643 |
|  | .333 | .172 | .379 | .300 | .290 | .319 | .296 | .323 | .261 |
|  | [-.034, 1.271] | [-.249, .424] | [-.767, .721] | [-.652, .524] | [-.806, .332] | [-1.056, .194] | [-.730, .433] | [-.482, .785] | [-1.155, -.130] |
|  | (.063) | (.610) | (.951) | (.831) | (.415) | (.177) | (.616) | (.639) | (.014) |
| Virginia | .126 | .114 | .129 | .084 | -.177 | -.183 | -.153 | -.249 | .029 |
|  | .215 | .094 | .211 | .191 | .156 | .180 | .201 | .194 | .185 |
|  | [-.295, .547] | [-.070, .299] | [-.284, .543] | [-.291, .459] | [-.484, .129] | [-.536, .169] | [-.546, .241] | [-.631, .132] | [-.334, .391] |
|  | (.557) | (.224) | (.540) | (.660) | (.257) | (.309) | (.446) | (.199) | (.877) |
| Washington | .374 | .083 | .280 | .269 | -.244 | .000 | .036 | -.001 | -.032 |
|  | .228 | .106 | .210 | .201 | .166 | .190 | .204 | .199 | .189 |
|  | [-.072, .820] | [-.125, .290] | [-.131, .692] | [-.126, .664] | [-.570, .082] | [-.372, .372] | [-.365, .437] | [-.390, .389] | [-.403, .340] |
|  | (.100) | (.436) | (.181) | (.181) | (.143) | (.999) | (.860) | (.997) | (.867) |
| West Virginia | -.227 | .129 | .175 | .215 | -.214 | .031 | .027 | -.040 | .161 |
|  | .295 | .133 | .270 | .261 | .220 | .260 | .234 | .279 | .236 |
|  | [-.806, .352] | [-.133, .391] | [-.354, .704] | [-.297, .727] | [-.645, .217] | [-.478, .541] | [-.431, .485] | [-.588, .507] | [-.302, .624] |
|  | (.443) | (.334) | (.518) | (.411) | (.330) | (.904) | (.909) | (.885) | (.496) |
| Wisconsin | .073 | .080 | -.010 | .069 | -.326 | -.385 | -.188 | -.269 | -.181 |
|  | .243 | .105 | .223 | .207 | .183 | .199 | .216 | .203 | .194 |
|  | [-.404, .549] | [-.125, .285] | [-.447, .426] | [-.337, .475] | [-.685, .033] | [-.775, .005] | [-.611, .235] | [-.666, .129] | [-.560, .199] |
|  | (.765) | (.446) | (.963) | (.739) | (.075) | (.053) | (.384) | (.186) | (.350) |
| Wyoming | .607 | .133 | -.660 | -.381 | .127 | -.281 | .032 | -.022 | -.022 |
|  | .317 | .242 | .860 | .840 | .295 | .794 | .574 | .783 | .792 |
|  | [-.015, 1.228] | [-.342, .607] | [-2.346, 1.026] | [-2.029, 1.266] | [-.451, .705] | [-1.838, 1.277] | [-1.093, 1.157] | [-1.557, 1.514] | [-1.575, 1.531] |
|  | (.056) | (.584) | (.443) | (.650) | (.667) | (.724) | (.956) | (.978) | (.977) |
| Observations | 2982 | 2980 | 2968 | 2980 | 2980 | 2981 | 2981 | 2981 | 2979 |
| Pseudo-R^2^ |  |  |  |  |  |  |  |  |  |
| Model χ^2^ |  |  |  |  |  |  |  |  |  |
| d.f. | 88 | 88 | 88 | 88 | 88 | 88 | 88 | 88 | 88 |
| p-value | 2.75e-12 | 1.81e-53 | 1.18e-67 | 1.01e-28 | 6.11e-15 | 1.85e-27 | 3.55e-15 | 3.81e-17 | 9.21e-29 |

S25 Table. Regression Results, Health Policies, Adjusting for News Source.

|  | (1) | (2) | (3) | (4) | (5) | (6) | (7) | (8) | (9) |
| --- | --- | --- | --- | --- | --- | --- | --- | --- | --- |
|  | freetesting | waivecosts | closeschools | canceleverything | paidleave | compensatefirms | nationalguard | stopevents | stateslead |
| Republican | -.464 | -.628 | -.368 | -.307 | -.467 | .110 | -.170 | -.393 | .011 |
|  | .054 | .056 | .060 | .058 | .056 | .061 | .068 | .070 | .056 |
|  | [-.570, -.358] | [-.738, -.518] | [-.485, -.251] | [-.420, -.193] | [-.577, -.356] | [-.011, .230] | [-.304, -.037] | [-.531, -.255] | [-.100, .121] |
|  | (<.001) | (<.001) | (<.001) | (<.001) | (<.001) | (.074) | (.012) | (<.001) | (.850) |
| Other | -.268 | -.338 | -.304 | -.348 | -.353 | -.206 | -.374 | -.341 | -.145 |
|  | .039 | .043 | .047 | .047 | .043 | .055 | .056 | .056 | .048 |
|  | [-.344, -.193] | [-.423, -.254] | [-.396, -.213] | [-.440, -.256] | [-.438, -.269] | [-.313, -.098] | [-.482, -.265] | [-.450, -.232] | [-.238, -.051] |
|  | (<.001) | (<.001) | (<.001) | (<.001) | (<.001) | (<.001) | (<.001) | (<.001) | (.002) |
| rightnews=1 | -.215 | -.255 | -.217 | -.210 | -.291 | .151 | -.104 | -.248 | .028 |
|  | .055 | .058 | .061 | .061 | .058 | .057 | .068 | .070 | .056 |
|  | [-.322, -.108] | [-.368, -.141] | [-.338, -.097] | [-.328, -.091] | [-.405, -.177] | [.040, .263] | [-.237, .030] | [-.386, -.110] | [-.082, .137] |
|  | (<.001) | (<.001) | (<.001) | (.001) | (<.001) | (.008) | (.127) | (<.001) | (.620) |
| 35- | .148 | .173 | .120 | .151 | .147 | .085 | .026 | .035 | .188 |
|  | .049 | .055 | .061 | .059 | .057 | .068 | .069 | .070 | .059 |
|  | [.051, .244] | [.064, .282] | [.000, .239] | [.036, .267] | [.035, .259] | [-.048, .217] | [-.109, .160] | [-.103, .172] | [.072, .304] |
|  | (.003) | (.002) | (.050) | (.010) | (.010) | (.210) | (.710) | (.620) | (.001) |
| 50- | .176 | .146 | .128 | .166 | .105 | .014 | -.066 | .204 | .175 |
|  | .049 | .053 | .057 | .058 | .054 | .064 | .065 | .066 | .056 |
|  | [.079, .273] | [.042, .249] | [.017, .239] | [.053, .279] | [-.001, .211] | [-.111, .139] | [-.193, .061] | [.075, .333] | [.065, .285] |
|  | (<.001) | (.006) | (.024) | (.004) | (.053) | (.825) | (.306) | (.002) | (.002) |
| 70- | .234 | .049 | .158 | .363 | .036 | -.064 | .239 | .273 | .307 |
|  | .060 | .068 | .070 | .068 | .066 | .078 | .082 | .083 | .068 |
|  | [.117, .351] | [-.084, .182] | [.020, .296] | [.230, .495] | [-.094, .166] | [-.216, .088] | [.079, .399] | [.112, .435] | [.173, .441] |
|  | (<.001) | (.472) | (.025) | (<.001) | (.584) | (.410) | (.003) | (.001) | (<.001) |
| gender | .094 | .039 | .170 | .221 | .124 | .329 | .186 | .196 | .099 |
|  | .034 | .038 | .041 | .040 | .038 | .045 | .049 | .048 | .041 |
|  | [.027, .161] | [-.036, .114] | [.090, .249] | [.142, .300] | [.050, .198] | [.240, .418] | [.091, .281] | [.102, .290] | [.019, .179] |
|  | (.006) | (.308) | (<.001) | (<.001) | (.001) | (<.001) | (<.001) | (<.001) | (.016) |
| white | .118 | .158 | .017 | .013 | .140 | -.060 | -.182 | .257 | -.031 |
|  | .042 | .046 | .049 | .048 | .046 | .055 | .057 | .059 | .050 |
|  | [.037, .200] | [.067, .248] | [-.079, .112] | [-.082, .107] | [.050, .229] | [-.168, .049] | [-.294, -.070] | [.140, .373] | [-.128, .067] |
|  | (.004) | (.001) | (.735) | (.791) | (.002) | (.282) | (.001) | (<.001) | (.538) |
| married | -.036 | -.012 | -.006 | .045 | -.012 | -.014 | .133 | -.050 | -.003 |
|  | .038 | .041 | .043 | .043 | .041 | .050 | .052 | .053 | .044 |
|  | [-.109, .038] | [-.093, .069] | [-.090, .078] | [-.040, .130] | [-.092, .069] | [-.112, .084] | [.032, .234] | [-.153, .054] | [-.090, .083] |
|  | (.344) | (.775) | (.883) | (.298) | (.779) | (.785) | (.010) | (.347) | (.940) |
| High school graduate | -.197 | -.180 | -.136 | -.092 | -.244 | -.257 | -.139 | .092 | -.233 |
|  | .087 | .101 | .119 | .128 | .115 | .123 | .141 | .158 | .107 |
|  | [-.368, -.026] | [-.377, .018] | [-.368, .097] | [-.344, .159] | [-.470, -.018] | [-.499, -.015] | [-.414, .137] | [-.217, .401] | [-.444, -.023] |
|  | (.024) | (.074) | (.254) | (.471) | (.034) | (.038) | (.324) | (.559) | (.030) |
| Some college | -.119 | -.101 | -.175 | -.095 | -.153 | -.412 | -.399 | .302 | -.241 |
|  | .090 | .104 | .122 | .132 | .119 | .128 | .144 | .159 | .111 |
|  | [-.295, .057] | [-.305, .103] | [-.415, .064] | [-.353, .163] | [-.387, .080] | [-.663, -.161] | [-.681, -.117] | [-.010, .614] | [-.459, -.023] |
|  | (.186) | (.332) | (.152) | (.472) | (.198) | (.001) | (.006) | (.058) | (.030) |
| 2-year | -.094 | -.113 | -.185 | -.087 | -.171 | -.248 | -.334 | .276 | -.248 |
|  | .093 | .109 | .128 | .137 | .123 | .134 | .151 | .166 | .118 |
|  | [-.276, .089] | [-.327, .100] | [-.437, .067] | [-.355, .182] | [-.411, .070] | [-.511, .015] | [-.630, -.037] | [-.048, .601] | [-.480, -.016] |
|  | (.315) | (.299) | (.150) | (.527) | (.164) | (.065) | (.027) | (.095) | (.036) |
| 4-year | -.263 | -.266 | -.259 | -.154 | -.280 | -.403 | -.402 | .264 | -.274 |
|  | .093 | .106 | .123 | .134 | .119 | .130 | .147 | .162 | .114 |
|  | [-.444, -.082] | [-.474, -.058] | [-.500, -.017] | [-.416, .108] | [-.514, -.046] | [-.657, -.149] | [-.690, -.113] | [-.054, .581] | [-.498, -.051] |
|  | (.005) | (.012) | (.036) | (.248) | (.019) | (.002) | (.006) | (.104) | (.016) |
| Post-grad | -.241 | -.262 | -.340 | -.124 | -.293 | -.504 | -.366 | .348 | -.418 |
|  | .098 | .113 | .131 | .138 | .126 | .138 | .155 | .170 | .123 |
|  | [-.433, -.049] | [-.484, -.039] | [-.597, -.082] | [-.395, .147] | [-.540, -.046] | [-.775, -.233] | [-.670, -.062] | [.015, .680] | [-.659, -.178] |
|  | (.014) | (.021) | (.010) | (.369) | (.020) | (<.001) | (.018) | (.040) | (.001) |
| income=2 | .123 | .065 | .071 | .053 | .002 | -.092 | .069 | .009 | .060 |
|  | .105 | .114 | .114 | .122 | .113 | .131 | .131 | .140 | .116 |
|  | [-.083, .330] | [-.159, .289] | [-.153, .296] | [-.185, .292] | [-.219, .224] | [-.349, .165] | [-.188, .325] | [-.266, .284] | [-.167, .286] |
|  | (.242) | (.569) | (.534) | (.660) | (.983) | (.485) | (.600) | (.949) | (.606) |
| income=3 | .187 | .117 | .116 | .053 | .136 | -.088 | -.025 | -.092 | .226 |
|  | .101 | .110 | .111 | .116 | .105 | .126 | .130 | .136 | .112 |
|  | [-.011, .385] | [-.099, .333] | [-.102, .334] | [-.175, .281] | [-.070, .341] | [-.336, .159] | [-.279, .230] | [-.359, .174] | [.005, .446] |
|  | (.064) | (.288) | (.295) | (.647) | (.196) | (.484) | (.850) | (.497) | (.045) |
| income=4 | .214 | .193 | .117 | .144 | .094 | -.164 | .097 | .191 | .142 |
|  | .104 | .112 | .116 | .119 | .108 | .130 | .128 | .141 | .117 |
|  | [.011, .416] | [-.026, .411] | [-.110, .344] | [-.088, .377] | [-.119, .306] | [-.419, .091] | [-.154, .349] | [-.084, .467] | [-.086, .371] |
|  | (.039) | (.084) | (.312) | (.224) | (.388) | (.208) | (.447) | (.174) | (.222) |
| income=5 | .189 | .183 | -.028 | .037 | .099 | -.320 | -.148 | .033 | .089 |
|  | .107 | .112 | .121 | .122 | .111 | .133 | .133 | .141 | .118 |
|  | [-.021, .399] | [-.036, .401] | [-.265, .209] | [-.203, .277] | [-.119, .317] | [-.581, -.059] | [-.409, .112] | [-.243, .309] | [-.141, .320] |
|  | (.077) | (.102) | (.815) | (.761) | (.372) | (.016) | (.265) | (.816) | (.448) |
| income=6 | .175 | .111 | .145 | .143 | .128 | -.145 | .106 | .121 | .152 |
|  | .106 | .116 | .119 | .124 | .116 | .137 | .134 | .145 | .119 |
|  | [-.033, .384] | [-.116, .338] | [-.089, .378] | [-.099, .385] | [-.099, .356] | [-.414, .123] | [-.157, .370] | [-.163, .405] | [-.082, .385] |
|  | (.100) | (.337) | (.224) | (.247) | (.268) | (.288) | (.429) | (.405) | (.202) |
| income=7 | .234 | .022 | .233 | .212 | .113 | .015 | .036 | .267 | .025 |
|  | .108 | .122 | .119 | .123 | .115 | .138 | .138 | .147 | .126 |
|  | [.021, .447] | [-.217, .261] | [-.001, .467] | [-.030, .454] | [-.114, .339] | [-.256, .287] | [-.234, .307] | [-.021, .554] | [-.222, .272] |
|  | (.031) | (.857) | (.051) | (.086) | (.329) | (.912) | (.792) | (.069) | (.842) |
| income=8 | .214 | .115 | .107 | .072 | .235 | -.074 | -.129 | .079 | .082 |
|  | .105 | .118 | .121 | .126 | .112 | .138 | .144 | .146 | .123 |
|  | [.008, .421] | [-.117, .348] | [-.130, .343] | [-.176, .320] | [.016, .453] | [-.345, .197] | [-.412, .154] | [-.206, .365] | [-.158, .322] |
|  | (.042) | (.330) | (.376) | (.568) | (.036) | (.593) | (.372) | (.586) | (.503) |
| income=9 | .023 | -.123 | .084 | -.008 | .043 | -.175 | -.238 | .216 | .047 |
|  | .112 | .124 | .122 | .126 | .116 | .134 | .136 | .142 | .126 |
|  | [-.197, .243] | [-.366, .119] | [-.156, .324] | [-.255, .240] | [-.185, .271] | [-.439, .088] | [-.504, .028] | [-.062, .494] | [-.201, .294] |
|  | (.837) | (.319) | (.495) | (.951) | (.709) | (.193) | (.080) | (.128) | (.712) |
| income=10 | .196 | .053 | .033 | .163 | .137 | -.080 | -.078 | .214 | .076 |
|  | .112 | .120 | .126 | .128 | .118 | .143 | .143 | .153 | .129 |
|  | [-.023, .415] | [-.183, .288] | [-.214, .280] | [-.089, .415] | [-.094, .369] | [-.360, .200] | [-.359, .203] | [-.086, .513] | [-.177, .329] |
|  | (.079) | (.661) | (.795) | (.204) | (.245) | (.575) | (.585) | (.162) | (.554) |
| income=11 | .222 | -.040 | .195 | .233 | .022 | -.097 | -.121 | .284 | .108 |
|  | .113 | .132 | .131 | .131 | .124 | .151 | .150 | .151 | .133 |
|  | [-.000, .444] | [-.298, .218] | [-.061, .452] | [-.023, .490] | [-.222, .266] | [-.392, .198] | [-.415, .172] | [-.011, .579] | [-.153, .369] |
|  | (.050) | (.760) | (.136) | (.075) | (.858) | (.520) | (.418) | (.059) | (.417) |
| income=12 | .076 | -.120 | .075 | .109 | .008 | -.111 | -.271 | .357 | .085 |
|  | .127 | .140 | .144 | .149 | .135 | .166 | .170 | .170 | .145 |
|  | [-.173, .324] | [-.395, .155] | [-.208, .358] | [-.182, .401] | [-.256, .272] | [-.438, .215] | [-.604, .062] | [.024, .690] | [-.200, .370] |
|  | (.551) | (.391) | (.605) | (.462) | (.955) | (.503) | (.111) | (.035) | (.558) |
| income=13 | .393 | .191 | .128 | .344 | .167 | -.388 | -.074 | .370 | .048 |
|  | .138 | .167 | .196 | .195 | .170 | .232 | .246 | .221 | .194 |
|  | [.122, .665] | [-.136, .518] | [-.256, .511] | [-.039, .727] | [-.166, .500] | [-.843, .066] | [-.556, .407] | [-.062, .803] | [-.332, .428] |
|  | (.005) | (.253) | (.514) | (.078) | (.325) | (.094) | (.762) | (.093) | (.805) |
| income=14 | -.111 | -.172 | -.254 | -.000 | -.081 | -.247 | -.503 | .298 | -.263 |
|  | .213 | .212 | .224 | .209 | .207 | .221 | .270 | .222 | .226 |
|  | [-.528, .306] | [-.587, .243] | [-.694, .186] | [-.409, .409] | [-.486, .325] | [-.679, .186] | [-1.033, .027] | [-.138, .734] | [-.707, .180] |
|  | (.601) | (.417) | (.258) | (1.000) | (.697) | (.264) | (.063) | (.180) | (.245) |
| income=15 | .668 | .723 | -.013 | .265 | .453 | .196 | 1.397 | .812 | 1.252 |
|  | .198 | .282 | .577 | .671 | .119 | .407 | .339 | .161 | .152 |
|  | [.279, 1.056] | [.171, 1.276] | [-1.144, 1.117] | [-1.051, 1.580] | [.219, .688] | [-.603, .994] | [.732,2.061] | [.496, 1.128] | [.953, 1.551] |
|  | (.001) | (.010) | (.982) | (.693) | (<.001) | (.631) | (<.001) | (<.001) | (<.001) |
| income=16 | .434 | .484 | .438 | .499 | .599 | -.040 | .110 | -.000 | .164 |
|  | .211 | .221 | .274 | .256 | .179 | .336 | .382 | .389 | .344 |
|  | [.022, .847] | [.051, .917] | [-.099, .975] | [-.003, 1.002] | [.248, .950] | [-.700, .620] | [-.640, .860] | [-.763, .763] | [-.510, .838] |
|  | (.039) | (.029) | (.110) | (.051) | (.001) | (.905) | (.773) | (1.000) | (.634) |
| income=17 | .084 | .017 | .005 | .017 | -.020 | -.192 | -.326 | .204 | .105 |
|  | .101 | .109 | .110 | .114 | .106 | .124 | .124 | .128 | .109 |
|  | [-.114, .282] | [-.198, .231] | [-.210, .221] | [-.206, .240] | [-.228, .189] | [-.435, .051] | [-.570, -.082] | [-.048, .455] | [-.110, .319] |
|  | (.404) | (.879) | (.960) | (.882) | (.854) | (.122) | (.009) | (.113) | (.338) |
| Rural Code=2 | -.051 | -.053 | -.111 | -.083 | .003 | -.114 | -.121 | .021 | -.038 |
|  | .046 | .050 | .055 | .054 | .050 | .060 | .063 | .063 | .054 |
|  | [-.140, .038] | [-.151, .046] | [-.218, -.005] | [-.189, .024] | [-.095, .100] | [-.233, .004] | [-.245, .002] | [-.102, .144] | [-.144, .069] |
|  | (.263) | (.294) | (.041) | (.127) | (.959) | (.058) | (.055) | (.735) | (.486) |
| Rural Code=3 | -.095 | -.199 | -.159 | -.042 | -.026 | -.029 | -.045 | .045 | -.065 |
|  | .064 | .071 | .074 | .071 | .070 | .082 | .084 | .084 | .071 |
|  | [-.221, .031] | [-.338, -.059] | [-.305, -.014] | [-.182, .098] | [-.164, .111] | [-.191, .133] | [-.211, .120] | [-.119, .209] | [-.204, .074] |
|  | (.139) | (.005) | (.032) | (.558) | (.707) | (.724) | (.592) | (.592) | (.358) |
| Rural Code=4 | -.045 | .046 | -.241 | -.059 | .093 | -.057 | -.145 | -.135 | .052 |
|  | .094 | .089 | .112 | .108 | .093 | .117 | .117 | .123 | .098 |
|  | [-.230, .140] | [-.129, .220] | [-.460, -.021] | [-.272, .153] | [-.089, .275] | [-.286, .172] | [-.375, .085] | [-.377, .107] | [-.140, .243] |
|  | (.636) | (.608) | (.032) | (.584) | (.315) | (.627) | (.215) | (.275) | (.597) |
| Rural Code=5 | -.034 | -.128 | .055 | .073 | .097 | -.083 | .011 | .297 | -.118 |
|  | .150 | .159 | .151 | .146 | .145 | .171 | .186 | .170 | .153 |
|  | [-.328, .259] | [-.440, .184] | [-.242, .352] | [-.214, .360] | [-.187, .381] | [-.418, .253] | [-.353, .375] | [-.036, .630] | [-.418, .181] |
|  | (.820) | (.421) | (.717) | (.618) | (.503) | (.628) | (.955) | (.080) | (.438) |
| Rural Code=6 | .004 | -.029 | -.097 | -.131 | .052 | -.005 | .021 | -.066 | -.232 |
|  | .084 | .093 | .098 | .105 | .088 | .102 | .127 | .119 | .111 |
|  | [-.161, .169] | [-.211, .152] | [-.290, .095] | [-.337, .076] | [-.121, .225] | [-.206, .195] | [-.228, .269] | [-.298, .167] | [-.450, -.014] |
|  | (.962) | (.752) | (.323) | (.215) | (.555) | (.959) | (.871) | (.579) | (.037) |
| Rural Code=7 | -.201 | -.099 | -.330 | -.180 | -.209 | -.169 | -.113 | -.208 | -.225 |
|  | .124 | .131 | .148 | .143 | .144 | .146 | .158 | .177 | .152 |
|  | [-.445, .042] | [-.356, .158] | [-.620, -.041] | [-.461, .101] | [-.492, .074] | [-.456, .118] | [-.423, .197] | [-.554, .139] | [-.523, .073] |
|  | (.106) | (.451) | (.025) | (.209) | (.147) | (.248) | (.476) | (.240) | (.139) |
| Rural Code=8 | -.035 | -.088 | -.216 | -.385 | .020 | .085 | -.711 | -.001 | .429 |
|  | .277 | .270 | .324 | .277 | .284 | .275 | .290 | .307 | .203 |
|  | [-.578, .509] | [-.617, .442] | [-.852, .420] | [-.928, .158] | [-.536, .576] | [-.455, .624] | [-1.280, -.142] | [-.603, .602] | [.031, .827] |
|  | (.901) | (.745) | (.505) | (.165) | (.944) | (.758) | (.014) | (.998) | (.035) |
| Rural Code=9 | -.035 | .042 | .055 | -.075 | .027 | -.428 | .039 | .036 | -.023 |
|  | .194 | .181 | .207 | .241 | .223 | .250 | .245 | .248 | .160 |
|  | [-.416, .346] | [-.313, .397] | [-.351, .461] | [-.547, .398] | [-.411, .465] | [-.918, .063] | [-.442, .520] | [-.449, .522] | [-.337, .292] |
|  | (.857) | (.818) | (.790) | (.757) | (.905) | (.088) | (.873) | (.884) | (.888) |
| Alaska | -.014 | -.050 | .281 | .240 | -.008 | .397 | .136 | .333 | -.017 |
|  | .222 | .449 | .364 | .306 | .295 | .354 | .524 | .420 | .423 |
|  | [-.450, .421] | [-.929, .830] | [-.432, .995] | [-.359, .839] | [-.586, .570] | [-.297, 1.091] | [-.891, 1.163] | [-.490, 1.157] | [-.848, .813] |
|  | (.949) | (.912) | (.439) | (.432) | (.978) | (.262) | (.795) | (.428) | (.967) |
| Arizona | -.211 | -.074 | -.011 | -.121 | -.065 | -.289 | -.325 | .099 | -.283 |
|  | .159 | .189 | .222 | .199 | .187 | .220 | .222 | .298 | .226 |
|  | [-.523, .100] | [-.444, .297] | [-.447, .425] | [-.512, .269] | [-.432, .302] | [-.719, .142] | [-.760, .109] | [-.485, .683] | [-.726, .159] |
|  | (.183) | (.696) | (.961) | (.543) | (.728) | (.189) | (.142) | (.740) | (.209) |
| Arkansas | -.191 | -.170 | -.090 | -.122 | -.156 | -.251 | -.065 | .111 | -.119 |
|  | .158 | .201 | .247 | .207 | .217 | .267 | .262 | .313 | .250 |
|  | [-.502, .119] | [-.565, .225] | [-.575, .394] | [-.528, .284] | [-.580, .269] | [-.774, .272] | [-.578, .448] | [-.502, .724] | [-.610, .372] |
|  | (.227) | (.399) | (.715) | (.556) | (.471) | (.347) | (.804) | (.722) | (.636) |
| California | -.093 | .076 | .058 | -.053 | .004 | -.240 | .119 | .250 | -.186 |
|  | .122 | .157 | .187 | .169 | .157 | .200 | .190 | .265 | .198 |
|  | [-.333, .147] | [-.233, .385] | [-.308, .424] | [-.384, .278] | [-.304, .312] | [-.631, .152] | [-.254, .492] | [-.270, .771] | [-.575, .203] |
|  | (.447) | (.630) | (.755) | (.754) | (.980) | (.230) | (.532) | (.345) | (.349) |
| Colorado | -.112 | -.346 | -.108 | -.074 | -.153 | -.233 | .045 | .308 | -.146 |
|  | .186 | .230 | .253 | .242 | .226 | .258 | .270 | .319 | .247 |
|  | [-.476, .252] | [-.797, .105] | [-.604, .388] | [-.549, .401] | [-.595, .289] | [-.738, .272] | [-.484, .574] | [-.317, .934] | [-.630, .338] |
|  | (.545) | (.132) | (.670) | (.760) | (.498) | (.366) | (.867) | (.334) | (.554) |
| Connecticut | .092 | .281 | .499 | .425 | .072 | .167 | .242 | .176 | .215 |
|  | .199 | .184 | .208 | .205 | .211 | .278 | .282 | .359 | .249 |
|  | [-.298, .481] | [-.079, .642] | [.091, .906] | [.022, .828] | [-.342, .486] | [-.378, .711] | [-.312, .795] | [-.528, .880] | [-.273, .703] |
|  | (.644) | (.126) | (.016) | (.039) | (.733) | (.548) | (.392) | (.624) | (.388) |
| Delaware | -.340 | .167 | .092 | .112 | -.369 | -.609 | .305 | -.053 | -.269 |
|  | .229 | .243 | .295 | .240 | .302 | .444 | .364 | .434 | .333 |
|  | [-.788, .108] | [-.309, .643] | [-.486, .671] | [-.359, .583] | [-.961, .222] | [-1.480, .261] | [-.409, 1.019] | [-.905, .799] | [-.922, .384] |
|  | (.137) | (.492) | (.754) | (.642) | (.221) | (.170) | (.402) | (.903) | (.420) |
| District of Columbia | -.582 | -.023 | .165 | -.160 | -.540 | .025 | .523 | .634 | .114 |
|  | .423 | .276 | .465 | .367 | .566 | .291 | .495 | .431 | .405 |
|  | [-1.411, .247] | [-.564, .518] | [-.747, 1.077] | [-.881, .560] | [-1.649, .569] | [-.545, .594] | [-.447, 1.494] | [-.211, 1.478] | [-.679, .907] |
|  | (.169) | (.934) | (.723) | (.663) | (.340) | (.933) | (.291) | (.141) | (.778) |
| Florida | -.142 | -.080 | .158 | -.094 | -.117 | -.152 | .236 | .283 | -.054 |
|  | .124 | .161 | .188 | .169 | .160 | .199 | .189 | .264 | .199 |
|  | [-.386, .102] | [-.395, .236] | [-.210, .526] | [-.427, .238] | [-.431, .198] | [-.543, .238] | [-.134, .606] | [-.235, .800] | [-.445, .336] |
|  | (.253) | (.621) | (.401) | (.577) | (.467) | (.444) | (.211) | (.284) | (.785) |
| Georgia | -.211 | -.316 | .018 | -.229 | -.072 | -.337 | .188 | .032 | -.141 |
|  | .145 | .184 | .204 | .191 | .174 | .210 | .210 | .281 | .211 |
|  | [-.496, .073] | [-.677, .044] | [-.383, .418] | [-.603, .146] | [-.412, .268] | [-.749, .076] | [-.223, .599] | [-.518, .582] | [-.554, .272] |
|  | (.146) | (.086) | (.932) | (.232) | (.679) | (.110) | (.369) | (.910) | (.504) |
| Hawaii | .230 | .564 | .588 | .335 | .052 | -.111 | .411 | -.045 | .052 |
|  | .161 | .173 | .240 | .268 | .259 | .421 | .370 | .523 | .317 |
|  | [-.085, .545] | [.225, .902] | [.117, 1.058] | [-.189, .860] | [-.457, .560] | [-.936, .713] | [-.314, 1.136] | [-1.070, .981] | [-.568, .673] |
|  | (.153) | (.001) | (.014) | (.210) | (.842) | (.791) | (.267) | (.932) | (.869) |
| Idaho | -.374 | -.422 | .190 | -.321 | -.505 | -.329 | -.518 | .334 | -.278 |
|  | .298 | .326 | .275 | .319 | .297 | .296 | .291 | .389 | .323 |
|  | [-.959, .210] | [-1.062, .218] | [-.350, .730] | [-.947, .306] | [-1.087, .077] | [-.909, .252] | [-1.089, .053] | [-.429, 1.097] | [-.910, .354] |
|  | (.209) | (.196) | (.490) | (.315) | (.089) | (.267) | (.075) | (.391) | (.389) |
| Illinois | -.114 | -.100 | .143 | .045 | .017 | -.219 | .191 | .291 | -.070 |
|  | .134 | .175 | .198 | .180 | .175 | .215 | .208 | .281 | .209 |
|  | [-.376, .148] | [-.443, .244] | [-.246, .532] | [-.309, .398] | [-.326, .359] | [-.642, .203] | [-.218, .600] | [-.260, .842] | [-.480, .340] |
|  | (.393) | (.569) | (.472) | (.804) | (.924) | (.309) | (.359) | (.300) | (.738) |
| Indiana | .008 | .255 | .482 | .219 | .009 | -.241 | .071 | .673 | -.196 |
|  | .149 | .177 | .208 | .192 | .198 | .241 | .238 | .287 | .236 |
|  | [-.285, .300] | [-.092, .601] | [.074, .890] | [-.158, .595] | [-.379, .396] | [-.713, .231] | [-.395, .538] | [.109, 1.237] | [-.658, .266] |
|  | (.958) | (.150) | (.021) | (.255) | (.965) | (.317) | (.764) | (.019) | (.406) |
| Iowa | -.185 | -.188 | .031 | .057 | -.101 | .118 | .329 | .579 | -.052 |
|  | .199 | .233 | .268 | .227 | .212 | .243 | .288 | .312 | .274 |
|  | [-.575, .204] | [-.645, .270] | [-.495, .556] | [-.387, .501] | [-.516, .314] | [-.358, .593] | [-.236, .893] | [-.032, 1.190] | [-.589, .486] |
|  | (.351) | (.421) | (.909) | (.801) | (.633) | (.627) | (.254) | (.063) | (.850) |
| Kansas | -.043 | .130 | -.007 | .323 | .107 | -.190 | .141 | .556 | -.178 |
|  | .236 | .319 | .278 | .258 | .344 | .336 | .389 | .371 | .335 |
|  | [-.505, .419] | [-.495, .756] | [-.552, .538] | [-.184, .829] | [-.567, .781] | [-.849, .468] | [-.621, .904] | [-.172, 1.283] | [-.835, .479] |
|  | (.856) | (.683) | (.979) | (.212) | (.756) | (.571) | (.716) | (.134) | (.595) |
| Kentucky | -.374 | -.136 | .031 | -.310 | -.087 | .122 | -.192 | .231 | -.074 |
|  | .185 | .213 | .245 | .227 | .203 | .239 | .242 | .319 | .235 |
|  | [-.737, -.011] | [-.554, .283] | [-.450, .512] | [-.756, .135] | [-.486, .312] | [-.346, .590] | [-.666, .283] | [-.394, .857] | [-.535, .387] |
|  | (.043) | (.525) | (.899) | (.172) | (.670) | (.609) | (.428) | (.468) | (.753) |
| Louisiana | -.216 | .145 | .031 | -.211 | -.144 | -.190 | .104 | .086 | -.133 |
|  | .216 | .224 | .264 | .251 | .233 | .284 | .254 | .341 | .250 |
|  | [-.639, .208] | [-.294, .585] | [-.487, .548] | [-.703, .281] | [-.601, .313] | [-.748, .367] | [-.394, .603] | [-.582, .755] | [-.623, .357] |
|  | (.318) | (.517) | (.908) | (.401) | (.537) | (.503) | (.681) | (.800) | (.596) |
| Maine | -.142 | -.017 | .755 | .383 | .113 | -.120 | -.361 | .319 | .306 |
|  | .287 | .284 | .214 | .332 | .250 | .368 | .358 | .423 | .284 |
|  | [-.704, .420] | [-.575, .540] | [.335, 1.175] | [-.268, 1.033] | [-.378, .604] | [-.841, .602] | [-1.063, .340] | [-.509, 1.148] | [-.251, .863] |
|  | (.621) | (.952) | (<.001) | (.249) | (.652) | (.745) | (.313) | (.450) | (.282) |
| Maryland | -.130 | -.022 | -.045 | -.195 | .150 | -.241 | .268 | .149 | -.071 |
|  | .167 | .208 | .252 | .231 | .201 | .253 | .273 | .312 | .253 |
|  | [-.459, .198] | [-.431, .387] | [-.538, .449] | [-.647, .257] | [-.245, .545] | [-.738, .256] | [-.268, .805] | [-.464, .761] | [-.567, .425] |
|  | (.436) | (.916) | (.859) | (.398) | (.457) | (.342) | (.327) | (.634) | (.778) |
| Massachusetts | -.047 | .079 | -.030 | -.041 | .057 | -.130 | .086 | .479 | -.325 |
|  | .154 | .193 | .224 | .216 | .185 | .247 | .226 | .290 | .242 |
|  | [-.348, .254] | [-.300, .458] | [-.468, .409] | [-.466, .383] | [-.305, .419] | [-.615, .354] | [-.357, .529] | [-.089, 1.046] | [-.800, .150] |
|  | (.758) | (.684) | (.894) | (.848) | (.757) | (.598) | (.703) | (.099) | (.180) |
| Michigan | -.150 | .003 | .246 | -.179 | -.156 | -.175 | .024 | .367 | -.083 |
|  | .140 | .170 | .196 | .189 | .176 | .213 | .214 | .278 | .213 |
|  | [-.426, .125] | [-.330, .337] | [-.139, .631] | [-.550, .192] | [-.500, .189] | [-.593, .243] | [-.395, .443] | [-.178, .911] | [-.499, .334] |
|  | (.284) | (.984) | (.211) | (.345) | (.376) | (.411) | (.910) | (.187) | (.698) |
| Minnesota | -.323 | -.176 | .120 | -.013 | -.232 | -.196 | .034 | .204 | .036 |
|  | .182 | .211 | .253 | .209 | .196 | .246 | .256 | .327 | .248 |
|  | [-.679, .033] | [-.590, .238] | [-.376, .616] | [-.423, .397] | [-.615, .152] | [-.678, .285] | [-.467, .535] | [-.437, .845] | [-.451, .523] |
|  | (.075) | (.405) | (.636) | (.950) | (.237) | (.424) | (.893) | (.533) | (.885) |
| Mississippi | -.409 | -.323 | .125 | -.041 | -.110 | .078 | .261 | .054 | .069 |
|  | .250 | .265 | .258 | .236 | .232 | .258 | .275 | .356 | .248 |
|  | [-.900, .082] | [-.843, .196] | [-.381, .631] | [-.504, .422] | [-.564, .344] | [-.427, .583] | [-.278, .801] | [-.644, .752] | [-.417, .555] |
|  | (.102) | (.222) | (.629) | (.862) | (.634) | (.762) | (.343) | (.880) | (.782) |
| Missouri | -.313 | -.160 | -.050 | -.285 | -.034 | -.317 | .094 | .143 | -.090 |
|  | .168 | .189 | .215 | .210 | .182 | .238 | .221 | .295 | .229 |
|  | [-.642, .017] | [-.530, .210] | [-.472, .372] | [-.696, .126] | [-.391, .323] | [-.783, .149] | [-.339, .527] | [-.435, .720] | [-.539, .359] |
|  | (.063) | (.398) | (.816) | (.174) | (.852) | (.182) | (.672) | (.628) | (.694) |
| Montana | -.736 | -.709 | -.199 | -.578 | -.143 | -.800 | -.340 | -.408 | -.513 |
|  | .361 | .386 | .342 | .368 | .328 | .319 | .432 | .424 | .386 |
|  | [-1.443, -.029] | [-1.466, .048] | [-.870, .473] | [-1.300, .144] | [-.787, .500] | [-1.426, -.175] | [-1.187, .507] | [-1.238, .423] | [-1.270, .245] |
|  | (.041) | (.066) | (.562) | (.117) | (.662) | (.012) | (.431) | (.336) | (.185) |
| Nebraska | -.144 | .165 | -.454 | -.235 | -.075 | .080 | .405 | -.152 | -.087 |
|  | .224 | .250 | .364 | .341 | .275 | .376 | .451 | .421 | .322 |
|  | [-.584, .296] | [-.326, .656] | [-1.168, .259] | [-.902, .433] | [-.614, .464] | [-.657, .816] | [-.479, 1.290] | [-.976, .673] | [-.718, .544] |
|  | (.520) | (.510) | (.212) | (.491) | (.785) | (.832) | (.369) | (.718) | (.788) |
| Nevada | -.059 | .041 | .169 | -.015 | -.170 | -.361 | -.189 | .380 | -.003 |
|  | .160 | .202 | .229 | .206 | .224 | .258 | .244 | .312 | .228 |
|  | [-.372, .254] | [-.355, .438] | [-.281, .619] | [-.420, .389] | [-.609, .269] | [-.867, .144] | [-.668, .291] | [-.231, .991] | [-.451, .445] |
|  | (.712) | (.838) | (.461) | (.940) | (.447) | (.161) | (.440) | (.223) | (.989) |
| New Hampshire | -.038 | .159 | -.076 | -.195 | -.029 | -.245 | .316 | -.010 | .052 |
|  | .205 | .285 | .306 | .292 | .283 | .295 | .334 | .373 | .295 |
|  | [-.439, .364] | [-.400, .719] | [-.677, .524] | [-.766, .377] | [-.584, .525] | [-.823, .332] | [-.338, .970] | [-.741, .722] | [-.527, .631] |
|  | (.854) | (.577) | (.803) | (.505) | (.917) | (.405) | (.344) | (.979) | (.860) |
| New Jersey | -.113 | .175 | .182 | -.054 | .007 | .038 | .381 | .184 | -.027 |
|  | .166 | .191 | .219 | .200 | .195 | .230 | .228 | .288 | .229 |
|  | [-.437, .212] | [-.199, .549] | [-.248, .612] | [-.446, .337] | [-.376, .390] | [-.413, .489] | [-.066, .828] | [-.380, .749] | [-.476, .421] |
|  | (.496) | (.359) | (.407) | (.785) | (.971) | (.869) | (.095) | (.522) | (.906) |
| New Mexico | .121 | -.172 | .245 | -.154 | -.171 | -.330 | -.421 | .275 | -.352 |
|  | .175 | .281 | .269 | .276 | .243 | .288 | .333 | .356 | .286 |
|  | [-.221, .464] | [-.723, .379] | [-.283, .772] | [-.696, .387] | [-.647, .305] | [-.895, .234] | [-1.075, .233] | [-.422, .972] | [-.914, .209] |
|  | (.488) | (.540) | (.363) | (.576) | (.481) | (.251) | (.207) | (.439) | (.219) |
| New York | -.295 | -.037 | .091 | -.120 | -.119 | -.170 | .202 | -.008 | -.044 |
|  | .133 | .163 | .193 | .176 | .164 | .203 | .195 | .272 | .204 |
|  | [-.557, -.034] | [-.358, .283] | [-.287, .469] | [-.465, .226] | [-.440, .202] | [-.567, .228] | [-.180, .584] | [-.542, .526] | [-.445, .357] |
|  | (.027) | (.819) | (.638) | (.497) | (.468) | (.403) | (.299) | (.976) | (.830) |
| North Carolina | -.141 | .068 | .046 | .079 | -.074 | -.372 | .245 | .056 | -.262 |
|  | .144 | .173 | .212 | .185 | .178 | .223 | .215 | .290 | .225 |
|  | [-.424, .142] | [-.271, .408] | [-.369, .461] | [-.284, .442] | [-.424, .275] | [-.810, .065] | [-.177, .666] | [-.513, .626] | [-.704, .179] |
|  | (.329) | (.694) | (.828) | (.669) | (.677) | (.095) | (.255) | (.846) | (.244) |
| North Dakota | -.225 | .384 | -.403 | -.294 | .146 | -.145 | .520 | -.138 | -.484 |
|  | .436 | .265 | .445 | .295 | .251 | .533 | .359 | .420 | .370 |
|  | [-1.081, .630] | [-.135, .903] | [-1.276, .470] | [-.873, .285] | [-.345, .638] | [-1.191, .901] | [-.185, 1.225] | [-.961, .685] | [-1.208, .241] |
|  | (.606) | (.147) | (.366) | (.320) | (.559) | (.785) | (.148) | (.742) | (.191) |
| Ohio | -.204 | -.031 | .153 | -.023 | -.040 | -.074 | -.030 | .330 | .074 |
|  | .141 | .168 | .204 | .187 | .168 | .217 | .205 | .280 | .206 |
|  | [-.480, .071] | [-.362, .299] | [-.246, .552] | [-.389, .344] | [-.369, .288] | [-.500, .351] | [-.431, .372] | [-.219, .879] | [-.330, .478] |
|  | (.146) | (.852) | (.452) | (.904) | (.811) | (.731) | (.884) | (.239) | (.721) |
| Oklahoma | -.285 | -.022 | .185 | .157 | -.172 | .249 | .403 | .001 | -.220 |
|  | .193 | .224 | .265 | .216 | .236 | .277 | .280 | .373 | .290 |
|  | [-.664, .094] | [-.461, .418] | [-.334, .704] | [-.267, .581] | [-.635, .290] | [-.294, .793] | [-.147, .953] | [-.731, .733] | [-.788, .348] |
|  | (.140) | (.923) | (.485) | (.468) | (.465) | (.368) | (.151) | (.998) | (.448) |
| Oregon | .044 | .139 | -.275 | -.132 | -.025 | -.344 | -.074 | .262 | -.394 |
|  | .140 | .186 | .230 | .204 | .195 | .245 | .236 | .297 | .234 |
|  | [-.230, .318] | [-.227, .505] | [-.726, .176] | [-.532, .267] | [-.407, .357] | [-.824, .137] | [-.536, .389] | [-.322, .845] | [-.853, .066] |
|  | (.753) | (.456) | (.232) | (.516) | (.899) | (.161) | (.755) | (.379) | (.093) |
| Pennsylvania | -.135 | -.052 | .078 | -.044 | -.018 | -.171 | -.002 | .314 | -.018 |
|  | .128 | .161 | .191 | .172 | .160 | .203 | .190 | .266 | .200 |
|  | [-.386, .117] | [-.367, .263] | [-.298, .453] | [-.381, .294] | [-.332, .297] | [-.570, .227] | [-.375, .371] | [-.209, .836] | [-.409, .374] |
|  | (.294) | (.747) | (.685) | (.800) | (.913) | (.399) | (.991) | (.239) | (.930) |
| Rhode Island | .326 | .299 | .302 | -.043 | .267 | .826 | .034 | -.099 | -.038 |
|  | .169 | .333 | .377 | .433 | .297 | .283 | .498 | .584 | .377 |
|  | [-.006, .658] | [-.353, .952] | [-.437, 1.041] | [-.891, .806] | [-.315, .849] | [.271, 1.382] | [-.942, 1.010] | [-1.245, 1.046] | [-.776, .701] |
|  | (.054) | (.368) | (.423) | (.921) | (.368) | (.004) | (.945) | (.865) | (.920) |
| South Carolina | -.136 | -.130 | .261 | .156 | -.331 | -.113 | .011 | .384 | -.030 |
|  | .199 | .233 | .229 | .214 | .242 | .247 | .260 | .303 | .247 |
|  | [-.525, .254] | [-.587, .327] | [-.188, .710] | [-.264, .576] | [-.806, .143] | [-.597, .370] | [-.498, .519] | [-.210, .978] | [-.515, .455] |
|  | (.494) | (.578) | (.254) | (.467) | (.171) | (.646) | (.968) | (.205) | (.903) |
| South Dakota | -.307 | -.181 | -.539 | -.271 | -.274 | -.195 | .213 | -.419 | -.062 |
|  | .433 | .332 | .354 | .373 | .445 | .441 | .372 | .413 | .244 |
|  | [-1.157, .543] | [-.833, .471] | [-1.233, .155] | [-1.001, .460] | [-1.147, .599] | [-1.060, .671] | [-.516, .942] | [-1.230, .391] | [-.540, .416] |
|  | (.479) | (.587) | (.128) | (.467) | (.538) | (.660) | (.566) | (.310) | (.798) |
| Tennessee | .045 | .193 | .034 | -.276 | -.075 | -.214 | -.103 | .335 | -.228 |
|  | .152 | .204 | .240 | .231 | .206 | .235 | .243 | .314 | .249 |
|  | [-.254, .344] | [-.208, .593] | [-.436, .505] | [-.728, .177] | [-.479, .329] | [-.675, .247] | [-.579, .374] | [-.281, .950] | [-.717, .261] |
|  | (.768) | (.346) | (.886) | (.232) | (.714) | (.363) | (.672) | (.287) | (.361) |
| Texas | -.186 | -.107 | -.024 | -.118 | -.254 | -.273 | -.013 | .081 | -.088 |
|  | .129 | .167 | .193 | .175 | .168 | .204 | .194 | .272 | .203 |
|  | [-.438, .067] | [-.434, .219] | [-.403, .354] | [-.462, .226] | [-.584, .076] | [-.673, .126] | [-.394, .368] | [-.453, .614] | [-.485, .309] |
|  | (.149) | (.519) | (.900) | (.501) | (.131) | (.180) | (.946) | (.767) | (.664) |
| Utah | -.218 | -.033 | -.079 | .016 | -.078 | -.446 | .215 | .514 | -.286 |
|  | .213 | .242 | .258 | .257 | .232 | .330 | .290 | .321 | .281 |
|  | [-.635, .199] | [-.507, .441] | [-.586, .428] | [-.488, .520] | [-.532, .377] | [-1.094, .202] | [-.353, .783] | [-.115, 1.143] | [-.838, .265] |
|  | (.304) | (.892) | (.760) | (.951) | (.737) | (.177) | (.458) | (.109) | (.309) |
| Vermont | .037 | -.049 | .337 | .328 | -.356 | -.296 | .268 | .683 | -.047 |
|  | .186 | .339 | .330 | .265 | .367 | .353 | .481 | .397 | .366 |
|  | [-.328, .402] | [-.714, .616] | [-.310, .984] | [-.193, .848] | [-1.075, .363] | [-.987, .396] | [-.674, 1.210] | [-.096, 1.461] | [-.765, .670] |
|  | (.843) | (.884) | (.307) | (.217) | (.331) | (.402) | (.577) | (.086) | (.897) |
| Virginia | -.105 | -.050 | -.007 | -.134 | -.076 | -.373 | .080 | .310 | .005 |
|  | .139 | .177 | .205 | .189 | .176 | .220 | .210 | .283 | .213 |
|  | [-.379, .168] | [-.397, .297] | [-.408, .395] | [-.504, .237] | [-.422, .269] | [-.804, .058] | [-.331, .491] | [-.245, .865] | [-.413, .423] |
|  | (.451) | (.777) | (.974) | (.479) | (.665) | (.090) | (.703) | (.273) | (.981) |
| Washington | -.103 | -.008 | -.101 | -.147 | .032 | -.335 | -.215 | .312 | -.289 |
|  | .157 | .196 | .215 | .193 | .187 | .225 | .210 | .278 | .225 |
|  | [-.412, .205] | [-.393, .376] | [-.523, .321] | [-.526, .233] | [-.336, .399] | [-.776, .107] | [-.627, .198] | [-.234, .858] | [-.731, .152] |
|  | (.511) | (.967) | (.638) | (.449) | (.866) | (.137) | (.308) | (.262) | (.199) |
| West Virginia | -.639 | -.074 | .292 | -.034 | -.193 | -.320 | .138 | .399 | .020 |
|  | .279 | .236 | .259 | .261 | .261 | .285 | .310 | .342 | .290 |
|  | [-1.186, -.093] | [-.537, .390] | [-.216, .801] | [-.546, .478] | [-.705, .319] | [-.879, .238] | [-.469, .745] | [-.272, 1.070] | [-.548, .588] |
|  | (.022) | (.756) | (.260) | (.896) | (.459) | (.260) | (.655) | (.244) | (.946) |
| Wisconsin | -.342 | -.088 | .071 | -.099 | -.329 | -.359 | .036 | .312 | -.125 |
|  | .170 | .187 | .226 | .208 | .196 | .228 | .231 | .288 | .222 |
|  | [-.674, -.010] | [-.454, .279] | [-.372, .514] | [-.506, .309] | [-.712, .055] | [-.805, .088] | [-.417, .489] | [-.253, .877] | [-.561, .311] |
|  | (.044) | (.639) | (.752) | (.636) | (.093) | (.116) | (.875) | (.279) | (.575) |
| Wyoming | .633 | .742 | .288 | -.003 | -.040 | -.259 | 1.296 | -.499 | -.234 |
|  | .185 | .267 | .678 | .737 | .589 | .685 | .424 | 1.013 | .610 |
|  | [.269, .996] | [.218, 1.266] | [-1.041, 1.617] | [-1.447, 1.442] | [-1.196, 1.115] | [-1.603, 1.085] | [.465,2.127] | [-2.486, 1.488] | [-1.431, .963] |
|  | (.001) | (.006) | (.671) | (.997) | (.945) | (.705) | (.002) | (.622) | (.701) |
| Observations | 2970 | 2974 | 2967 | 2973 | 2973 | 2973 | 2974 | 2975 | 2973 |
| Pseudo-R^2^ |  |  |  |  |  |  |  |  |  |
| Model χ^2^ |  |  |  |  |  |  |  |  |  |
| d.f. | 88 | 88 | 88 | 88 | 88 | 88 | 88 | 88 | 88 |
| p-value | 1.92e-33 | 2.38e-53 | 6.30e-23 | 1.42e-20 | 3.21e-34 | 1.40e-16 | 6.03e-30 | 4.54e-37 | 1.92e-20 |

S26 Table. Regression Results, Public Policies, Adjusting for News Source.

|  | (1) | (2) | (3) | (4) | (5) | (6) | (7) | (8) | (9) | (10) |
| --- | --- | --- | --- | --- | --- | --- | --- | --- | --- | --- |
|  | increasetrade | importtaxes | lowinterest | notreliant | noairtravel | banentry | entrymexico | quarantine | rescueUS | delayelections |
| Republican | -.522 | .562 | .129 | .497 | .261 | .485 | .780 | .431 | -.138 | .375 |
|  | .057 | .058 | .054 | .055 | .062 | .060 | .055 | .071 | .063 | .075 |
|  | [-.633, -.411] | [.447, .676] | [.023, .236] | [.390, .604] | [.140, .381] | [.367, .602] | [.673, .888] | [.293, .570] | [-.262, -.014] | [.228, .521] |
|  | (<.001) | (<.001) | (.017) | (<.001) | (<.001) | (<.001) | (<.001) | (<.001) | (.030) | (<.001) |
| Other | -.477 | .238 | -.079 | .295 | .074 | .123 | .385 | .030 | -.186 | .138 |
|  | .045 | .049 | .047 | .050 | .053 | .053 | .054 | .057 | .052 | .064 |
|  | [-.565, -.389] | [.141, .335] | [-.172, .014] | [.198, .392] | [-.030, .178] | [.019, .227] | [.279, .490] | [-.081, .142] | [-.289, -.083] | [.012, .263] |
|  | (<.001) | (<.001) | (.094) | (<.001) | (.162) | (.020) | (<.001) | (.594) | (<.001) | (.031) |
| rightnews=1 | -.253 | .388 | .293 | .491 | .234 | .405 | .501 | .190 | .077 | -.114 |
|  | .059 | .057 | .051 | .045 | .059 | .054 | .047 | .069 | .062 | .073 |
|  | [-.368, -.137] | [.277, .500] | [.193, .392] | [.402, .580] | [.118, .350] | [.299, .510] | [.407, .594] | [.054, .325] | [-.045, .199] | [-.256, .029] |
|  | (<.001) | (<.001) | (<.001) | (<.001) | (<.001) | (<.001) | (<.001) | (.006) | (.218) | (.118) |
| 35- | -.083 | .161 | .167 | .295 | .202 | .138 | .198 | .123 | .036 | -.116 |
|  | .055 | .061 | .058 | .059 | .064 | .064 | .064 | .072 | .064 | .076 |
|  | [-.191, .025] | [.042, .281] | [.053, .280] | [.180, .410] | [.076, .328] | [.013, .263] | [.073, .323] | [-.018, .265] | [-.089, .162] | [-.264, .033] |
|  | (.131) | (.008) | (.004) | (<.001) | (.002) | (.030) | (.002) | (.087) | (.569) | (.128) |
| 50- | .017 | .084 | .178 | .394 | .151 | .241 | .344 | -.039 | .111 | -.373 |
|  | .053 | .057 | .055 | .056 | .062 | .060 | .061 | .069 | .060 | .072 |
|  | [-.086, .121] | [-.028, .197] | [.070, .285] | [.284, .504] | [.029, .273] | [.122, .359] | [.225, .462] | [-.174, .097] | [-.008, .229] | [-.515, -.231] |
|  | (.747) | (.143) | (.001) | (<.001) | (.015) | (<.001) | (<.001) | (.577) | (.066) | (<.001) |
| 70- | .094 | -.027 | .189 | .357 | .082 | .158 | .349 | -.228 | .267 | -.252 |
|  | .068 | .070 | .068 | .071 | .077 | .077 | .073 | .083 | .075 | .090 |
|  | [-.039, .227] | [-.163, .110] | [.057, .322] | [.218, .496] | [-.068, .232] | [.008, .308] | [.206, .493] | [-.391, -.064] | [.120, .415] | [-.429, -.075] |
|  | (.164) | (.704) | (.005) | (<.001) | (.286) | (.039) | (<.001) | (.006) | (<.001) | (.005) |
| gender | -.150 | .182 | .114 | -.036 | .270 | .137 | .040 | .112 | -.057 | .426 |
|  | .039 | .042 | .040 | .040 | .044 | .043 | .043 | .049 | .044 | .054 |
|  | [-.227, -.074] | [.099, .266] | [.036, .192] | [-.114, .042] | [.183, .357] | [.052, .222] | [-.043, .124] | [.016, .209] | [-.144, .030] | [.321, .531] |
|  | (<.001) | (<.001) | (.004) | (.360) | (<.001) | (.002) | (.342) | (.023) | (.198) | (<.001) |
| white | -.026 | -.097 | -.072 | -.095 | -.115 | -.032 | -.087 | -.284 | .050 | -.106 |
|  | .047 | .052 | .048 | .048 | .052 | .053 | .051 | .061 | .054 | .064 |
|  | [-.119, .067] | [-.198, .005] | [-.165, .022] | [-.189, -.002] | [-.217, -.014] | [-.135, .071] | [-.188, .013] | [-.403, -.165] | [-.056, .156] | [-.230, .019] |
|  | (.582) | (.062) | (.132) | (.045) | (.026) | (.543) | (.088) | (<.001) | (.356) | (.098) |
| married | -.024 | .107 | -.028 | -.022 | .063 | -.000 | -.016 | .060 | -.065 | -.055 |
|  | .044 | .046 | .043 | .043 | .047 | .047 | .047 | .054 | .049 | .059 |
|  | [-.110, .061] | [.017, .198] | [-.112, .056] | [-.107, .063] | [-.029, .154] | [-.093, .093] | [-.108, .075] | [-.046, .167] | [-.162, .031] | [-.170, .060] |
|  | (.578) | (.020) | (.511) | (.610) | (.179) | (.994) | (.725) | (.268) | (.185) | (.349) |
| High school graduate | -.352 | -.069 | -.014 | -.054 | .032 | -.284 | -.136 | -.442 | .058 | -.005 |
|  | .111 | .122 | .123 | .115 | .118 | .111 | .109 | .144 | .134 | .151 |
|  | [-.571, -.134] | [-.309, .171] | [-.255, .228] | [-.280, .171] | [-.200, .263] | [-.501, -.067] | [-.350, .078] | [-.725, -.159] | [-.204, .321] | [-.301, .291] |
|  | (.002) | (.573) | (.911) | (.637) | (.788) | (.010) | (.213) | (.002) | (.663) | (.973) |
| Some college | -.324 | -.232 | -.052 | -.068 | -.207 | -.454 | -.288 | -.885 | .118 | -.216 |
|  | .114 | .126 | .126 | .119 | .122 | .114 | .114 | .146 | .137 | .155 |
|  | [-.548, -.100] | [-.479, .015] | [-.300, .195] | [-.301, .164] | [-.447, .032] | [-.678, -.230] | [-.512, -.064] | [-1.172, -.598] | [-.150, .386] | [-.519, .088] |
|  | (.005) | (.065) | (.680) | (.565) | (.090) | (<.001) | (.012) | (<.001) | (.388) | (.164) |
| 2-year | -.286 | -.072 | -.024 | .004 | -.103 | -.288 | -.188 | -.818 | .022 | -.106 |
|  | .118 | .130 | .131 | .122 | .128 | .120 | .118 | .154 | .142 | .163 |
|  | [-.518, -.053] | [-.327, .183] | [-.281, .234] | [-.237, .244] | [-.355, .148] | [-.523, -.053] | [-.420, .044] | [-1.120, -.515] | [-.256, .301] | [-.424, .213] |
|  | (.016) | (.578) | (.856) | (.977) | (.421) | (.016) | (.112) | (<.001) | (.875) | (.516) |
| 4-year | -.148 | -.343 | -.143 | -.219 | -.358 | -.621 | -.368 | -.937 | .066 | -.228 |
|  | .116 | .128 | .127 | .121 | .126 | .119 | .117 | .151 | .138 | .158 |
|  | [-.376, .080] | [-.593, -.092] | [-.392, .106] | [-.455, .018] | [-.606, -.110] | [-.854, -.388] | [-.597, -.139] | [-1.233, -.640] | [-.204, .337] | [-.539, .083] |
|  | (.203) | (.007) | (.261) | (.070) | (.005) | (<.001) | (.002) | (<.001) | (.631) | (.150) |
| Post-grad | -.090 | -.436 | -.171 | -.290 | -.552 | -.607 | -.501 | -1.109 | .017 | -.438 |
|  | .123 | .136 | .133 | .129 | .136 | .127 | .127 | .159 | .146 | .169 |
|  | [-.331, .150] | [-.703, -.169] | [-.433, .091] | [-.543, -.038] | [-.819, -.286] | [-.856, -.359] | [-.750, -.252] | [-1.421, -.798] | [-.269, .303] | [-.770, -.106] |
|  | (.461) | (.001) | (.200) | (.024) | (<.001) | (<.001) | (<.001) | (<.001) | (.909) | (.010) |
| income=2 | -.184 | .119 | .131 | -.012 | .143 | .145 | .156 | -.020 | .020 | .166 |
|  | .111 | .119 | .117 | .117 | .119 | .117 | .125 | .136 | .131 | .155 |
|  | [-.401, .032] | [-.115, .352] | [-.098, .359] | [-.240, .217] | [-.090, .376] | [-.085, .376] | [-.088, .401] | [-.286, .246] | [-.237, .278] | [-.138, .469] |
|  | (.096) | (.319) | (.261) | (.920) | (.229) | (.216) | (.209) | (.881) | (.877) | (.284) |
| income=3 | -.082 | .094 | .141 | .126 | .087 | .153 | .235 | -.234 | .143 | .208 |
|  | .105 | .114 | .116 | .110 | .115 | .116 | .120 | .132 | .126 | .152 |
|  | [-.287, .124] | [-.130, .319] | [-.085, .368] | [-.088, .341] | [-.139, .313] | [-.074, .379] | [.000, .470] | [-.492, .024] | [-.104, .391] | [-.089, .506] |
|  | (.436) | (.410) | (.221) | (.249) | (.450) | (.187) | (.050) | (.075) | (.257) | (.170) |
| income=4 | -.070 | .058 | .225 | .062 | .052 | -.024 | .125 | -.256 | .014 | .120 |
|  | .108 | .119 | .117 | .113 | .118 | .123 | .123 | .137 | .129 | .154 |
|  | [-.282, .142] | [-.175, .292] | [-.004, .453] | [-.160, .283] | [-.180, .285] | [-.265, .218] | [-.116, .366] | [-.526, .013] | [-.239, .268] | [-.182, .421] |
|  | (.518) | (.624) | (.054) | (.586) | (.658) | (.848) | (.310) | (.062) | (.912) | (.436) |
| income=5 | -.065 | -.011 | .095 | .177 | .039 | .074 | .219 | -.438 | .133 | .029 |
|  | .108 | .117 | .120 | .112 | .121 | .123 | .125 | .134 | .130 | .155 |
|  | [-.278, .147] | [-.239, .218] | [-.139, .330] | [-.042, .396] | [-.198, .275] | [-.166, .315] | [-.026, .464] | [-.701, -.176] | [-.121, .388] | [-.275, .332] |
|  | (.546) | (.928) | (.425) | (.114) | (.750) | (.544) | (.079) | (.001) | (.304) | (.853) |
| income=6 | -.080 | -.035 | .125 | .242 | .099 | .128 | .239 | -.310 | .081 | .193 |
|  | .108 | .123 | .120 | .114 | .122 | .122 | .124 | .141 | .130 | .161 |
|  | [-.293, .132] | [-.276, .206] | [-.111, .361] | [.019, .465] | [-.139, .338] | [-.112, .368] | [-.005, .483] | [-.585, -.034] | [-.174, .335] | [-.123, .510] |
|  | (.458) | (.775) | (.298) | (.034) | (.415) | (.296) | (.055) | (.028) | (.535) | (.231) |
| income=7 | -.142 | -.026 | .155 | .146 | .228 | .277 | .147 | -.310 | -.076 | .053 |
|  | .118 | .126 | .125 | .124 | .123 | .131 | .138 | .153 | .144 | .168 |
|  | [-.374, .090] | [-.273, .221] | [-.091, .400] | [-.096, .389] | [-.014, .469] | [.020, .534] | [-.123, .418] | [-.610, -.010] | [-.358, .205] | [-.276, .382] |
|  | (.229) | (.834) | (.216) | (.236) | (.065) | (.035) | (.286) | (.043) | (.595) | (.751) |
| income=8 | -.169 | -.067 | .169 | .085 | -.008 | -.000 | .143 | -.389 | -.001 | .027 |
|  | .117 | .128 | .123 | .121 | .128 | .131 | .129 | .145 | .138 | .165 |
|  | [-.399, .061] | [-.318, .184] | [-.073, .410] | [-.152, .321] | [-.260, .243] | [-.258, .257] | [-.110, .396] | [-.674, -.105] | [-.272, .271] | [-.295, .350] |
|  | (.150) | (.600) | (.171) | (.483) | (.948) | (.998) | (.268) | (.007) | (.996) | (.869) |
| income=9 | .027 | -.188 | .152 | .045 | -.197 | .074 | .040 | -.494 | .008 | -.170 |
|  | .115 | .123 | .124 | .122 | .131 | .131 | .136 | .140 | .140 | .164 |
|  | [-.198, .252] | [-.430, .054] | [-.091, .395] | [-.193, .284] | [-.453, .059] | [-.182, .330] | [-.227, .307] | [-.769, -.219] | [-.267, .284] | [-.493, .152] |
|  | (.812) | (.128) | (.220) | (.711) | (.132) | (.570) | (.770) | (<.001) | (.953) | (.300) |
| income=10 | .023 | -.198 | .156 | -.011 | -.040 | -.044 | .120 | -.525 | .024 | -.185 |
|  | .116 | .129 | .127 | .127 | .139 | .136 | .140 | .151 | .143 | .174 |
|  | [-.205, .251] | [-.451, .056] | [-.092, .405] | [-.261, .239] | [-.312, .232] | [-.311, .224] | [-.154, .394] | [-.821, -.230] | [-.256, .305] | [-.526, .156] |
|  | (.845) | (.126) | (.217) | (.932) | (.772) | (.748) | (.390) | (<.001) | (.865) | (.288) |
| income=11 | -.009 | -.229 | .168 | -.101 | -.187 | -.021 | .032 | -.639 | -.032 | .047 |
|  | .121 | .139 | .133 | .132 | .148 | .147 | .144 | .152 | .144 | .178 |
|  | [-.246, .228] | [-.502, .044] | [-.093, .428] | [-.361, .158] | [-.478, .104] | [-.310, .268] | [-.250, .313] | [-.936, -.341] | [-.314, .249] | [-.302, .396] |
|  | (.942) | (.101) | (.207) | (.445) | (.208) | (.888) | (.825) | (<.001) | (.821) | (.791) |
| income=12 | -.057 | -.176 | .121 | -.118 | -.113 | .014 | .205 | -.666 | -.003 | -.189 |
|  | .139 | .161 | .147 | .152 | .162 | .159 | .163 | .167 | .160 | .194 |
|  | [-.329, .215] | [-.491, .139] | [-.167, .409] | [-.416, .181] | [-.430, .204] | [-.297, .326] | [-.114, .525] | [-.994, -.337] | [-.317, .312] | [-.570, .191] |
|  | (.680) | (.273) | (.411) | (.439) | (.486) | (.928) | (.208) | (<.001) | (.987) | (.330) |
| income=13 | .123 | -.411 | -.156 | -.199 | -.282 | .070 | .041 | -.821 | -.166 | -.259 |
|  | .154 | .189 | .214 | .189 | .236 | .204 | .208 | .210 | .210 | .272 |
|  | [-.180, .426] | [-.781, -.041] | [-.575, .263] | [-.568, .171] | [-.746, .181] | [-.330, .471] | [-.367, .449] | [-1.233, -.408] | [-.577, .246] | [-.792, .274] |
|  | (.425) | (.029) | (.466) | (.292) | (.233) | (.730) | (.843) | (<.001) | (.430) | (.341) |
| income=14 | -.099 | -.178 | .132 | .036 | -.132 | .134 | .183 | -.707 | -.328 | .046 |
|  | .203 | .223 | .167 | .216 | .255 | .216 | .225 | .218 | .238 | .297 |
|  | [-.497, .298] | [-.615, .259] | [-.196, .460] | [-.387, .460] | [-.631, .368] | [-.289, .556] | [-.259, .624] | [-1.135, -.279] | [-.794, .139] | [-.537, .629] |
|  | (.625) | (.424) | (.431) | (.866) | (.605) | (.536) | (.418) | (.001) | (.169) | (.877) |
| income=15 | .185 | -.130 | -.378 | .516 | .753 | .774 | -.316 | -.205 | 1.090 | .491 |
|  | .696 | .935 | .588 | .529 | .299 | .231 | .936 | .971 | .347 | .773 |
|  | [-1.180, 1.550] | [-1.963, 1.703] | [-1.532, .775] | [-.521, 1.552] | [.167, 1.339] | [.321, 1.226] | [-2.151, 1.519] | [-2.109, 1.700] | [.411, 1.770] | [-1.025,2.008] |
|  | (.790) | (.890) | (.520) | (.329) | (.012) | (.001) | (.736) | (.833) | (.002) | (.525) |
| income=16 | -.143 | -.026 | .340 | -.107 | .041 | .409 | .503 | -.686 | -.150 | .082 |
|  | .304 | .321 | .281 | .313 | .300 | .232 | .217 | .371 | .427 | .357 |
|  | [-.739, .453] | [-.656, .604] | [-.211, .890] | [-.720, .506] | [-.547, .630] | [-.046, .864] | [.078, .928] | [-1.415, .042] | [-.986, .687] | [-.619, .782] |
|  | (.637) | (.936) | (.227) | (.732) | (.891) | (.078) | (.020) | (.065) | (.726) | (.819) |
| income=17 | -.229 | -.010 | -.025 | .079 | -.076 | .084 | .164 | -.353 | -.073 | -.036 |
|  | .102 | .111 | .112 | .106 | .113 | .114 | .117 | .129 | .122 | .145 |
|  | [-.429, -.029] | [-.227, .208] | [-.245, .196] | [-.129, .287] | [-.298, .146] | [-.138, .307] | [-.065, .393] | [-.606, -.100] | [-.312, .166] | [-.321, .248] |
|  | (.025) | (.930) | (.825) | (.455) | (.501) | (.458) | (.161) | (.006) | (.548) | (.803) |
| Rural Code=2 | -.107 | -.047 | -.070 | .060 | -.052 | -.047 | -.089 | -.031 | -.115 | -.152 |
|  | .052 | .055 | .052 | .050 | .060 | .056 | .056 | .064 | .059 | .072 |
|  | [-.209, -.004] | [-.156, .062] | [-.172, .031] | [-.039, .159] | [-.169, .065] | [-.157, .064] | [-.199, .021] | [-.156, .094] | [-.231, .001] | [-.292, -.011] |
|  | (.041) | (.395) | (.175) | (.235) | (.381) | (.407) | (.112) | (.625) | (.052) | (.035) |
| Rural Code=3 | -.085 | -.043 | -.021 | .039 | .011 | .079 | .006 | .054 | -.133 | .008 |
|  | .071 | .073 | .069 | .067 | .078 | .076 | .072 | .085 | .080 | .093 |
|  | [-.225, .055] | [-.187, .100] | [-.157, .115] | [-.093, .171] | [-.141, .164] | [-.070, .228] | [-.134, .146] | [-.114, .221] | [-.289, .023] | [-.173, .190] |
|  | (.232) | (.552) | (.765) | (.559) | (.884) | (.298) | (.932) | (.529) | (.094) | (.927) |
| Rural Code=4 | -.208 | .041 | .066 | .104 | .053 | .038 | .244 | .147 | .053 | -.191 |
|  | .099 | .101 | .099 | .099 | .108 | .104 | .095 | .115 | .113 | .127 |
|  | [-.403, -.014] | [-.156, .238] | [-.128, .259] | [-.091, .298] | [-.158, .264] | [-.166, .241] | [.056, .431] | [-.079, .373] | [-.168, .274] | [-.439, .057] |
|  | (.036) | (.682) | (.505) | (.297) | (.622) | (.716) | (.011) | (.203) | (.638) | (.131) |
| Rural Code=5 | -.101 | -.032 | -.129 | -.078 | .195 | .015 | .056 | -.026 | -.333 | .131 |
|  | .135 | .133 | .152 | .135 | .158 | .170 | .171 | .196 | .192 | .204 |
|  | [-.364, .163] | [-.293, .228] | [-.428, .170] | [-.342, .186] | [-.115, .505] | [-.319, .348] | [-.279, .391] | [-.411, .358] | [-.710, .043] | [-.270, .531] |
|  | (.455) | (.808) | (.397) | (.563) | (.217) | (.931) | (.743) | (.893) | (.083) | (.523) |
| Rural Code=6 | -.116 | .101 | .060 | .076 | .106 | .068 | .066 | .201 | -.073 | -.011 |
|  | .110 | .107 | .095 | .096 | .101 | .104 | .098 | .126 | .110 | .131 |
|  | [-.331, .099] | [-.110, .311] | [-.127, .247] | [-.112, .265] | [-.093, .305] | [-.136, .273] | [-.126, .258] | [-.046, .448] | [-.290, .144] | [-.269, .246] |
|  | (.291) | (.348) | (.532) | (.426) | (.296) | (.513) | (.500) | (.111) | (.509) | (.932) |
| Rural Code=7 | -.062 | -.016 | -.229 | .073 | .002 | .246 | -.027 | .007 | .062 | -.134 |
|  | .117 | .149 | .135 | .139 | .148 | .141 | .151 | .170 | .140 | .176 |
|  | [-.291, .167] | [-.309, .277] | [-.494, .037] | [-.199, .345] | [-.288, .292] | [-.030, .522] | [-.322, .268] | [-.326, .340] | [-.213, .336] | [-.480, .211] |
|  | (.594) | (.916) | (.091) | (.600) | (.988) | (.081) | (.858) | (.967) | (.661) | (.446) |
| Rural Code=8 | -.514 | -.077 | -.099 | .230 | .157 | -.094 | .210 | -.080 | .363 | .056 |
|  | .268 | .204 | .211 | .229 | .240 | .316 | .205 | .344 | .263 | .339 |
|  | [-1.040, .012] | [-.477, .324] | [-.512, .315] | [-.219, .679] | [-.312, .627] | [-.713, .525] | [-.192, .611] | [-.755, .595] | [-.152, .878] | [-.607, .720] |
|  | (.055) | (.707) | (.639) | (.315) | (.512) | (.765) | (.306) | (.816) | (.167) | (.868) |
| Rural Code=9 | -.087 | -.120 | -.099 | -.099 | -.086 | -.055 | -.400 | -.237 | -.371 | .225 |
|  | .211 | .207 | .168 | .239 | .241 | .209 | .243 | .222 | .172 | .240 |
|  | [-.500, .326] | [-.526, .285] | [-.429, .231] | [-.567, .370] | [-.559, .386] | [-.464, .355] | [-.878, .077] | [-.672, .197] | [-.707, -.034] | [-.244, .695] |
|  | (.680) | (.560) | (.558) | (.680) | (.720) | (.793) | (.100) | (.284) | (.031) | (.347) |
| Alaska | -.064 | .769 | .225 | .243 | .077 | -.527 | .051 | -.029 | -.087 | -.347 |
|  | .382 | .343 | .298 | .247 | .524 | .507 | .328 | .530 | .343 | .685 |
|  | [-.813, .685] | [.096, 1.441] | [-.360, .809] | [-.241, .728] | [-.950, 1.104] | [-1.521, .466] | [-.593, .695] | [-1.068, 1.010] | [-.759, .586] | [-1.689, .996] |
|  | (.868) | (.025) | (.451) | (.325) | (.883) | (.298) | (.876) | (.956) | (.800) | (.613) |
| Arizona | .008 | -.157 | -.451 | -.295 | -.386 | -.068 | -.411 | -.326 | -.103 | -.802 |
|  | .184 | .226 | .186 | .215 | .255 | .236 | .208 | .249 | .227 | .290 |
|  | [-.353, .369] | [-.600, .287] | [-.816, -.086] | [-.715, .126] | [-.885, .114] | [-.531, .394] | [-.818, -.003] | [-.815, .163] | [-.549, .343] | [-1.369, -.234] |
|  | (.967) | (.488) | (.016) | (.170) | (.130) | (.772) | (.048) | (.191) | (.651) | (.006) |
| Arkansas | .052 | .256 | -.062 | .111 | .014 | .205 | .109 | .139 | -.016 | -.005 |
|  | .210 | .250 | .216 | .218 | .270 | .257 | .224 | .282 | .245 | .323 |
|  | [-.361, .464] | [-.234, .745] | [-.486, .362] | [-.316, .538] | [-.516, .544] | [-.298, .708] | [-.331, .548] | [-.414, .692] | [-.496, .465] | [-.639, .628] |
|  | (.805) | (.306) | (.774) | (.611) | (.959) | (.424) | (.628) | (.622) | (.949) | (.987) |
| California | -.052 | .055 | -.271 | .051 | -.156 | -.014 | -.062 | -.029 | .000 | -.479 |
|  | .158 | .208 | .157 | .188 | .226 | .204 | .174 | .228 | .198 | .260 |
|  | [-.362, .258] | [-.352, .462] | [-.579, .037] | [-.318, .420] | [-.599, .287] | [-.413, .386] | [-.402, .278] | [-.475, .418] | [-.388, .388] | [-.989, .031] |
|  | (.743) | (.792) | (.085) | (.785) | (.490) | (.947) | (.721) | (.900) | (1.000) | (.066) |
| Colorado | -.395 | .187 | -.142 | -.060 | -.257 | .074 | -.074 | -.293 | -.332 | -.223 |
|  | .227 | .242 | .185 | .250 | .283 | .260 | .235 | .275 | .256 | .314 |
|  | [-.839, .049] | [-.288, .661] | [-.504, .220] | [-.551, .430] | [-.813, .298] | [-.435, .583] | [-.536, .387] | [-.832, .247] | [-.833, .169] | [-.838, .392] |
|  | (.081) | (.440) | (.441) | (.809) | (.364) | (.775) | (.752) | (.288) | (.194) | (.477) |
| Connecticut | .388 | .274 | -.226 | -.143 | -.171 | .396 | .111 | .081 | .187 | -.278 |
|  | .201 | .287 | .250 | .246 | .313 | .263 | .242 | .303 | .276 | .357 |
|  | [-.006, .782] | [-.289, .837] | [-.717, .264] | [-.627, .340] | [-.784, .443] | [-.119, .912] | [-.364, .586] | [-.514, .675] | [-.354, .728] | [-.977, .422] |
|  | (.054) | (.340) | (.365) | (.560) | (.585) | (.132) | (.646) | (.790) | (.499) | (.436) |
| Delaware | .071 | .182 | .122 | .016 | .060 | -.214 | .222 | -.126 | .237 | -.899 |
|  | .276 | .297 | .290 | .242 | .359 | .338 | .283 | .408 | .369 | .470 |
|  | [-.470, .613] | [-.399, .764] | [-.448, .691] | [-.459, .490] | [-.645, .764] | [-.877, .449] | [-.332, .776] | [-.926, .674] | [-.487, .960] | [-1.820, .022] |
|  | (.796) | (.539) | (.675) | (.949) | (.868) | (.527) | (.433) | (.758) | (.521) | (.056) |
| District of Columbia | -.180 | -.089 | .038 | -.468 | -.470 | .252 | .168 | -.282 | -.025 | .511 |
|  | .241 | .360 | .283 | .366 | .467 | .471 | .501 | .393 | .395 | .525 |
|  | [-.653, .294] | [-.796, .617] | [-.517, .593] | [-1.186, .250] | [-1.386, .447] | [-.671, 1.175] | [-.814, 1.151] | [-1.052, .488] | [-.799, .749] | [-.519, 1.540] |
|  | (.457) | (.805) | (.894) | (.201) | (.315) | (.592) | (.737) | (.473) | (.949) | (.331) |
| Florida | -.086 | .103 | -.117 | -.093 | -.219 | .082 | -.140 | .055 | -.019 | -.529 |
|  | .159 | .209 | .157 | .189 | .226 | .203 | .172 | .230 | .202 | .260 |
|  | [-.398, .225] | [-.307, .513] | [-.426, .191] | [-.463, .277] | [-.662, .224] | [-.316, .479] | [-.477, .197] | [-.396, .506] | [-.414, .376] | [-1.038, -.019] |
|  | (.587) | (.621) | (.455) | (.620) | (.333) | (.688) | (.416) | (.810) | (.924) | (.042) |
| Georgia | -.161 | .127 | -.268 | -.062 | -.395 | .077 | -.085 | .014 | -.189 | -.269 |
|  | .174 | .221 | .177 | .204 | .243 | .215 | .185 | .247 | .219 | .278 |
|  | [-.504, .181] | [-.306, .561] | [-.614, .078] | [-.462, .337] | [-.872, .082] | [-.345, .498] | [-.449, .278] | [-.470, .499] | [-.618, .240] | [-.814, .276] |
|  | (.355) | (.565) | (.129) | (.760) | (.105) | (.721) | (.646) | (.953) | (.388) | (.333) |
| Hawaii | .315 | .109 | .066 | -.437 | -.112 | .533 | -.269 | .121 | -.198 | -.138 |
|  | .283 | .329 | .283 | .494 | .469 | .318 | .381 | .444 | .399 | .473 |
|  | [-.240, .870] | [-.535, .754] | [-.489, .621] | [-1.405, .532] | [-1.031, .807] | [-.091, 1.157] | [-1.017, .478] | [-.751, .992] | [-.981, .585] | [-1.065, .789] |
|  | (.266) | (.740) | (.816) | (.377) | (.811) | (.094) | (.480) | (.786) | (.620) | (.770) |
| Idaho | -.257 | .023 | -.086 | -.077 | -.494 | -.347 | -.404 | .117 | -.465 | -.040 |
|  | .254 | .343 | .252 | .271 | .322 | .351 | .304 | .371 | .327 | .384 |
|  | [-.755, .240] | [-.649, .695] | [-.580, .409] | [-.610, .455] | [-1.125, .138] | [-1.035, .342] | [-1.000, .193] | [-.610, .844] | [-1.106, .176] | [-.793, .713] |
|  | (.311) | (.946) | (.734) | (.776) | (.125) | (.324) | (.184) | (.753) | (.155) | (.917) |
| Illinois | -.182 | .207 | -.215 | .148 | -.083 | .163 | .036 | .231 | -.178 | -.115 |
|  | .175 | .220 | .175 | .198 | .241 | .217 | .191 | .252 | .217 | .273 |
|  | [-.525, .162] | [-.225, .639] | [-.558, .129] | [-.241, .537] | [-.556, .390] | [-.263, .588] | [-.338, .411] | [-.263, .726] | [-.603, .247] | [-.651, .422] |
|  | (.300) | (.347) | (.221) | (.455) | (.732) | (.454) | (.850) | (.359) | (.411) | (.675) |
| Indiana | -.031 | .008 | -.057 | .021 | -.181 | .066 | -.094 | -.165 | -.011 | -.198 |
|  | .191 | .241 | .195 | .214 | .257 | .240 | .217 | .277 | .244 | .303 |
|  | [-.405, .343] | [-.464, .481] | [-.440, .326] | [-.399, .442] | [-.684, .322] | [-.404, .535] | [-.520, .332] | [-.708, .378] | [-.490, .467] | [-.793, .396] |
|  | (.870) | (.972) | (.770) | (.920) | (.481) | (.784) | (.665) | (.552) | (.963) | (.513) |
| Iowa | -.115 | .307 | -.288 | .005 | -.504 | .325 | .089 | .100 | -.202 | -.143 |
|  | .225 | .263 | .243 | .257 | .303 | .273 | .262 | .313 | .276 | .350 |
|  | [-.556, .325] | [-.210, .823] | [-.764, .189] | [-.499, .508] | [-1.097, .090] | [-.211, .861] | [-.424, .603] | [-.514, .715] | [-.743, .340] | [-.829, .542] |
|  | (.608) | (.244) | (.236) | (.986) | (.096) | (.235) | (.733) | (.749) | (.465) | (.682) |
| Kansas | -.546 | -.240 | -.197 | .166 | -.056 | .253 | -.058 | -.468 | -.433 | -.724 |
|  | .331 | .330 | .286 | .235 | .331 | .268 | .272 | .329 | .414 | .433 |
|  | [-1.195, .102] | [-.887, .406] | [-.758, .364] | [-.295, .628] | [-.706, .594] | [-.272, .779] | [-.592, .476] | [-1.113, .177] | [-1.245, .379] | [-1.573, .125] |
|  | (.099) | (.466) | (.491) | (.480) | (.866) | (.344) | (.832) | (.155) | (.296) | (.095) |
| Kentucky | -.229 | .404 | -.047 | .186 | -.092 | .277 | .306 | .020 | .150 | -.269 |
|  | .234 | .245 | .203 | .244 | .283 | .231 | .218 | .283 | .255 | .318 |
|  | [-.688, .230] | [-.076, .885] | [-.445, .352] | [-.292, .665] | [-.646, .463] | [-.176, .730] | [-.123, .734] | [-.536, .576] | [-.350, .651] | [-.893, .356] |
|  | (.328) | (.099) | (.819) | (.446) | (.746) | (.230) | (.162) | (.944) | (.556) | (.399) |
| Louisiana | -.239 | .420 | -.151 | -.173 | -.168 | .461 | .132 | .192 | -.057 | -.105 |
|  | .243 | .262 | .237 | .250 | .283 | .228 | .225 | .283 | .262 | .348 |
|  | [-.715, .238] | [-.095, .934] | [-.615, .313] | [-.662, .316] | [-.723, .388] | [.014, .908] | [-.308, .573] | [-.364, .748] | [-.571, .456] | [-.787, .577] |
|  | (.326) | (.110) | (.523) | (.489) | (.554) | (.043) | (.556) | (.498) | (.827) | (.763) |
| Maine | -.395 | .248 | .132 | .102 | .276 | .286 | .333 | .006 | -.468 | -.524 |
|  | .346 | .322 | .258 | .261 | .355 | .282 | .374 | .459 | .393 | .437 |
|  | [-1.074, .284] | [-.383, .879] | [-.374, .638] | [-.409, .613] | [-.421, .972] | [-.268, .839] | [-.401, 1.067] | [-.895, .907] | [-1.239, .303] | [-1.382, .334] |
|  | (.254) | (.440) | (.609) | (.695) | (.438) | (.311) | (.374) | (.989) | (.234) | (.231) |
| Maryland | .112 | -.106 | -.532 | -.299 | -.257 | -.023 | -.161 | -.171 | -.113 | -.264 |
|  | .189 | .257 | .226 | .250 | .286 | .285 | .246 | .289 | .254 | .337 |
|  | [-.259, .484] | [-.610, .398] | [-.976, -.089] | [-.790, .191] | [-.819, .305] | [-.583, .537] | [-.643, .321] | [-.738, .396] | [-.612, .385] | [-.924, .396] |
|  | (.552) | (.681) | (.019) | (.232) | (.370) | (.935) | (.512) | (.554) | (.655) | (.433) |
| Massachusetts | -.112 | -.165 | -.067 | -.215 | -.363 | .086 | -.057 | -.413 | .005 | -.040 |
|  | .193 | .234 | .189 | .228 | .268 | .245 | .213 | .249 | .233 | .304 |
|  | [-.491, .267] | [-.624, .295] | [-.438, .305] | [-.662, .232] | [-.889, .162] | [-.394, .567] | [-.475, .362] | [-.902, .076] | [-.452, .461] | [-.635, .555] |
|  | (.562) | (.482) | (.725) | (.345) | (.175) | (.725) | (.791) | (.098) | (.984) | (.894) |
| Michigan | -.172 | .182 | -.127 | -.077 | -.264 | .105 | .005 | -.115 | -.237 | -.451 |
|  | .181 | .222 | .169 | .204 | .242 | .215 | .190 | .239 | .213 | .278 |
|  | [-.528, .183] | [-.253, .617] | [-.459, .205] | [-.477, .323] | [-.740, .211] | [-.316, .527] | [-.367, .377] | [-.584, .355] | [-.654, .180] | [-.996, .094] |
|  | (.343) | (.411) | (.453) | (.706) | (.275) | (.624) | (.978) | (.632) | (.266) | (.105) |
| Minnesota | -.243 | .176 | -.556 | .145 | -.248 | -.071 | .005 | -.374 | -.372 | -.741 |
|  | .216 | .268 | .224 | .216 | .286 | .265 | .232 | .266 | .264 | .326 |
|  | [-.666, .181] | [-.349, .701] | [-.995, -.117] | [-.278, .568] | [-.809, .312] | [-.591, .449] | [-.450, .459] | [-.896, .148] | [-.891, .147] | [-1.381, -.101] |
|  | (.262) | (.512) | (.013) | (.501) | (.385) | (.789) | (.984) | (.160) | (.160) | (.023) |
| Mississippi | -.096 | .265 | -.581 | .072 | -.305 | -.186 | .234 | -.030 | -.331 | -.089 |
|  | .218 | .260 | .256 | .244 | .300 | .287 | .224 | .327 | .269 | .356 |
|  | [-.523, .332] | [-.246, .775] | [-1.082, -.080] | [-.406, .551] | [-.893, .283] | [-.748, .376] | [-.206, .673] | [-.671, .610] | [-.859, .197] | [-.786, .609] |
|  | (.661) | (.310) | (.023) | (.767) | (.309) | (.516) | (.297) | (.926) | (.219) | (.803) |
| Missouri | -.284 | .349 | -.157 | .119 | -.213 | .318 | .034 | .009 | -.172 | -.053 |
|  | .194 | .237 | .185 | .206 | .258 | .219 | .194 | .259 | .230 | .294 |
|  | [-.665, .097] | [-.116, .813] | [-.520, .205] | [-.284, .522] | [-.718, .292] | [-.111, .747] | [-.347, .415] | [-.498, .516] | [-.624, .279] | [-.630, .524] |
|  | (.144) | (.141) | (.394) | (.562) | (.408) | (.146) | (.861) | (.973) | (.454) | (.858) |
| Montana | .168 | -.149 | -.454 | .021 | -1.135 | -.763 | -.854 | -.333 | -.568 | -1.266 |
|  | .252 | .383 | .274 | .268 | .410 | .369 | .365 | .398 | .371 | .444 |
|  | [-.325, .662] | [-.900, .602] | [-.992, .083] | [-.504, .547] | [-1.939, -.332] | [-1.487, -.038] | [-1.571, -.138] | [-1.113, .447] | [-1.295, .159] | [-2.137, -.395] |
|  | (.504) | (.698) | (.098) | (.937) | (.006) | (.039) | (.019) | (.402) | (.126) | (.004) |
| Nebraska | -.335 | .280 | .034 | .494 | -.129 | -.204 | -.065 | -.171 | -.091 | -.293 |
|  | .400 | .401 | .280 | .287 | .427 | .424 | .339 | .444 | .367 | .498 |
|  | [-1.120, .450] | [-.506, 1.067] | [-.515, .584] | [-.069, 1.057] | [-.967, .708] | [-1.035, .628] | [-.730, .600] | [-1.042, .700] | [-.811, .629] | [-1.269, .683] |
|  | (.403) | (.485) | (.902) | (.086) | (.762) | (.631) | (.848) | (.701) | (.804) | (.556) |
| Nevada | -.140 | .004 | -.066 | -.008 | -.150 | .082 | -.076 | -.251 | -.319 | -.539 |
|  | .205 | .251 | .210 | .239 | .275 | .255 | .233 | .278 | .258 | .317 |
|  | [-.542, .263] | [-.488, .496] | [-.478, .347] | [-.476, .461] | [-.691, .390] | [-.418, .583] | [-.533, .381] | [-.795, .293] | [-.825, .188] | [-1.161, .083] |
|  | (.497) | (.987) | (.755) | (.974) | (.585) | (.748) | (.744) | (.366) | (.217) | (.090) |
| New Hampshire | -.100 | -.015 | -.292 | .245 | -.129 | .170 | .206 | .098 | .463 | -.753 |
|  | .262 | .292 | .307 | .267 | .300 | .297 | .256 | .356 | .307 | .405 |
|  | [-.613, .413] | [-.587, .557] | [-.893, .309] | [-.278, .768] | [-.718, .460] | [-.412, .751] | [-.296, .708] | [-.601, .797] | [-.138, 1.064] | [-1.547, .041] |
|  | (.702) | (.959) | (.341) | (.359) | (.667) | (.567) | (.421) | (.784) | (.131) | (.063) |
| New Jersey | -.256 | -.067 | -.038 | -.208 | -.044 | .058 | -.077 | -.089 | .018 | -.352 |
|  | .210 | .255 | .176 | .227 | .261 | .248 | .209 | .271 | .226 | .309 |
|  | [-.668, .157] | [-.566, .433] | [-.384, .307] | [-.653, .237] | [-.555, .466] | [-.428, .545] | [-.488, .333] | [-.620, .443] | [-.425, .460] | [-.958, .255] |
|  | (.224) | (.794) | (.828) | (.360) | (.864) | (.814) | (.712) | (.743) | (.938) | (.256) |
| New Mexico | -.226 | .630 | -.030 | .170 | -.014 | .227 | .237 | .150 | -.130 | -.370 |
|  | .260 | .301 | .228 | .256 | .298 | .269 | .238 | .320 | .316 | .357 |
|  | [-.735, .284] | [.040, 1.220] | [-.477, .417] | [-.333, .672] | [-.598, .570] | [-.300, .754] | [-.229, .703] | [-.477, .776] | [-.750, .489] | [-1.071, .331] |
|  | (.386) | (.036) | (.895) | (.508) | (.962) | (.399) | (.318) | (.639) | (.680) | (.301) |
| New York | -.176 | .307 | -.237 | .100 | -.038 | .230 | .165 | .182 | -.012 | -.244 |
|  | .166 | .212 | .164 | .191 | .231 | .206 | .178 | .236 | .206 | .265 |
|  | [-.502, .150] | [-.108, .722] | [-.558, .083] | [-.274, .474] | [-.491, .415] | [-.173, .633] | [-.185, .515] | [-.280, .644] | [-.416, .392] | [-.764, .275] |
|  | (.290) | (.147) | (.147) | (.598) | (.869) | (.264) | (.355) | (.440) | (.953) | (.357) |
| North Carolina | -.101 | .091 | -.239 | -.112 | .198 | .236 | .081 | .074 | -.217 | -.232 |
|  | .185 | .228 | .187 | .218 | .237 | .218 | .194 | .250 | .226 | .290 |
|  | [-.465, .262] | [-.356, .538] | [-.606, .127] | [-.540, .316] | [-.266, .662] | [-.192, .664] | [-.299, .462] | [-.416, .564] | [-.661, .227] | [-.801, .336] |
|  | (.585) | (.689) | (.200) | (.607) | (.402) | (.280) | (.676) | (.768) | (.337) | (.423) |
| North Dakota | .227 | -.015 | -.546 | -.419 | -.556 | -.682 | -.446 | -.083 | -.175 | -1.496 |
|  | .266 | .408 | .377 | .501 | .516 | .361 | .447 | .355 | .395 | .491 |
|  | [-.295, .749] | [-.816, .785] | [-1.284, .193] | [-1.401, .563] | [-1.568, .456] | [-1.389, .025] | [-1.323, .431] | [-.780, .613] | [-.949, .600] | [-2.459, -.533] |
|  | (.394) | (.970) | (.147) | (.403) | (.282) | (.059) | (.319) | (.815) | (.658) | (.002) |
| Ohio | -.105 | .265 | -.247 | .109 | -.081 | .199 | .005 | -.251 | -.229 | .069 |
|  | .176 | .219 | .177 | .200 | .240 | .220 | .189 | .245 | .217 | .271 |
|  | [-.450, .241] | [-.164, .694] | [-.594, .100] | [-.283, .502] | [-.551, .389] | [-.231, .630] | [-.366, .377] | [-.731, .229] | [-.654, .197] | [-.462, .600] |
|  | (.553) | (.226) | (.163) | (.586) | (.736) | (.364) | (.978) | (.305) | (.292) | (.798) |
| Oklahoma | -.306 | .058 | -.631 | -.122 | .023 | -.244 | -.212 | .033 | -.243 | -.649 |
|  | .289 | .295 | .283 | .310 | .284 | .310 | .296 | .323 | .324 | .371 |
|  | [-.872, .260] | [-.521, .637] | [-1.187, -.075] | [-.729, .485] | [-.533, .580] | [-.852, .365] | [-.793, .369] | [-.601, .668] | [-.878, .392] | [-1.376, .078] |
|  | (.289) | (.845) | (.026) | (.692) | (.934) | (.433) | (.475) | (.918) | (.453) | (.080) |
| Oregon | -.254 | -.026 | -.313 | .056 | -.835 | -.086 | -.424 | -.597 | .279 | -.416 |
|  | .207 | .239 | .201 | .217 | .274 | .253 | .220 | .245 | .232 | .301 |
|  | [-.660, .151] | [-.494, .442] | [-.706, .081] | [-.370, .482] | [-1.373, -.298] | [-.583, .411] | [-.856, .008] | [-1.078, -.116] | [-.176, .735] | [-1.007, .175] |
|  | (.219) | (.914) | (.120) | (.796) | (.002) | (.735) | (.054) | (.015) | (.229) | (.168) |
| Pennsylvania | -.151 | .109 | -.159 | .114 | .001 | .160 | .178 | -.190 | -.121 | -.081 |
|  | .158 | .209 | .161 | .190 | .230 | .206 | .174 | .230 | .204 | .260 |
|  | [-.461, .160] | [-.301, .519] | [-.474, .157] | [-.258, .486] | [-.449, .451] | [-.245, .565] | [-.163, .519] | [-.642, .261] | [-.520, .279] | [-.592, .429] |
|  | (.341) | (.601) | (.324) | (.548) | (.996) | (.439) | (.305) | (.408) | (.554) | (.755) |
| Rhode Island | -.081 | .882 | .026 | -.036 | .121 | .230 | .269 | .482 | -.263 | -.180 |
|  | .560 | .411 | .257 | .517 | .425 | .370 | .326 | .529 | .490 | .564 |
|  | [-1.180, 1.018] | [.076, 1.688] | [-.478, .530] | [-1.051, .978] | [-.713, .955] | [-.495, .955] | [-.371, .909] | [-.555, 1.519] | [-1.223, .698] | [-1.285, .925] |
|  | (.886) | (.032) | (.918) | (.944) | (.776) | (.534) | (.411) | (.362) | (.592) | (.750) |
| South Carolina | -.162 | -.090 | -.137 | .011 | -.059 | .233 | .102 | -.380 | -.106 | -.206 |
|  | .231 | .252 | .201 | .237 | .294 | .248 | .245 | .281 | .277 | .321 |
|  | [-.614, .291] | [-.584, .404] | [-.531, .256] | [-.453, .475] | [-.636, .518] | [-.254, .720] | [-.379, .583] | [-.931, .171] | [-.649, .436] | [-.835, .424] |
|  | (.484) | (.720) | (.493) | (.963) | (.840) | (.347) | (.677) | (.176) | (.701) | (.522) |
| South Dakota | -.080 | .322 | -.530 | .149 | -.091 | .167 | -.143 | -.680 | -.216 | -.198 |
|  | .311 | .369 | .371 | .323 | .396 | .439 | .401 | .413 | .371 | .363 |
|  | [-.689, .530] | [-.401, 1.045] | [-1.256, .197] | [-.483, .782] | [-.868, .685] | [-.695, 1.029] | [-.929, .643] | [-1.489, .130] | [-.944, .511] | [-.910, .515] |
|  | (.798) | (.382) | (.153) | (.644) | (.817) | (.704) | (.722) | (.100) | (.560) | (.586) |
| Tennessee | -.170 | -.177 | -.322 | -.258 | -.497 | -.149 | -.126 | .013 | -.010 | -.488 |
|  | .200 | .243 | .199 | .224 | .268 | .241 | .222 | .304 | .247 | .319 |
|  | [-.561, .222] | [-.653, .299] | [-.713, .069] | [-.698, .181] | [-1.023, .030] | [-.620, .323] | [-.561, .309] | [-.583, .609] | [-.495, .475] | [-1.114, .138] |
|  | (.395) | (.466) | (.106) | (.249) | (.064) | (.537) | (.571) | (.965) | (.969) | (.127) |
| Texas | -.139 | .094 | -.334 | -.073 | -.215 | .043 | .012 | -.056 | -.183 | -.495 |
|  | .165 | .211 | .162 | .191 | .231 | .208 | .176 | .232 | .205 | .266 |
|  | [-.463, .185] | [-.320, .509] | [-.651, -.016] | [-.448, .301] | [-.669, .238] | [-.366, .451] | [-.332, .357] | [-.512, .400] | [-.585, .220] | [-1.017, .028] |
|  | (.401) | (.655) | (.039) | (.701) | (.352) | (.837) | (.944) | (.809) | (.373) | (.063) |
| Utah | -.032 | -.237 | -.486 | -.325 | -.309 | -.063 | -.004 | -.606 | .043 | -1.009 |
|  | .241 | .287 | .273 | .256 | .292 | .267 | .257 | .307 | .306 | .377 |
|  | [-.504, .440] | [-.800, .326] | [-1.023, .050] | [-.827, .177] | [-.881, .264] | [-.586, .460] | [-.509, .501] | [-1.209, -.003] | [-.558, .643] | [-1.747, -.270] |
|  | (.894) | (.410) | (.075) | (.205) | (.290) | (.814) | (.988) | (.049) | (.890) | (.007) |
| Vermont | .311 | -.091 | -.105 | -.067 | -.344 | .236 | -.318 | -.832 | -.438 | -1.382 |
|  | .234 | .365 | .253 | .374 | .367 | .368 | .389 | .342 | .442 | .496 |
|  | [-.149, .771] | [-.806, .624] | [-.601, .391] | [-.800, .666] | [-1.063, .375] | [-.485, .957] | [-1.081, .444] | [-1.503, -.162] | [-1.305, .429] | [-2.355, -.410] |
|  | (.185) | (.802) | (.679) | (.857) | (.348) | (.521) | (.413) | (.015) | (.322) | (.005) |
| Virginia | -.081 | .120 | -.226 | -.076 | -.296 | .132 | -.064 | -.246 | .035 | -.534 |
|  | .175 | .223 | .173 | .213 | .245 | .223 | .194 | .247 | .219 | .287 |
|  | [-.425, .263] | [-.317, .557] | [-.566, .114] | [-.492, .341] | [-.778, .185] | [-.304, .568] | [-.444, .317] | [-.730, .238] | [-.394, .464] | [-1.098, .029] |
|  | (.646) | (.591) | (.193) | (.722) | (.227) | (.553) | (.743) | (.319) | (.872) | (.063) |
| Washington | -.183 | -.166 | -.134 | -.187 | -.745 | -.185 | -.488 | -.352 | -.287 | -.891 |
|  | .197 | .233 | .185 | .214 | .259 | .233 | .202 | .248 | .237 | .292 |
|  | [-.569, .203] | [-.622, .291] | [-.496, .228] | [-.607, .233] | [-1.253, -.237] | [-.642, .271] | [-.885, -.091] | [-.838, .134] | [-.752, .179] | [-1.463, -.318] |
|  | (.353) | (.477) | (.467) | (.383) | (.004) | (.426) | (.016) | (.155) | (.227) | (.002) |
| West Virginia | -.153 | .033 | -.242 | -.421 | .045 | .228 | .125 | .136 | .015 | -.485 |
|  | .265 | .269 | .252 | .297 | .310 | .304 | .291 | .343 | .281 | .377 |
|  | [-.673, .367] | [-.494, .559] | [-.736, .252] | [-1.004, .162] | [-.563, .653] | [-.368, .825] | [-.445, .696] | [-.537, .809] | [-.537, .567] | [-1.225, .255] |
|  | (.564) | (.904) | (.337) | (.157) | (.885) | (.453) | (.667) | (.692) | (.958) | (.199) |
| Wisconsin | .062 | -.020 | -.340 | -.097 | -.192 | .023 | -.151 | -.202 | -.421 | -.371 |
|  | .185 | .225 | .193 | .225 | .257 | .231 | .211 | .260 | .233 | .290 |
|  | [-.300, .425] | [-.461, .422] | [-.719, .039] | [-.539, .344] | [-.697, .313] | [-.430, .475] | [-.564, .262] | [-.712, .308] | [-.878, .036] | [-.939, .197] |
|  | (.736) | (.930) | (.078) | (.665) | (.456) | (.921) | (.474) | (.437) | (.071) | (.201) |
| Wyoming | -.654 | .994 | .149 | -.346 | .551 | -.463 | .718 | .970 | 1.057 | -.508 |
|  | 1.083 | .482 | .593 | .705 | .325 | 1.294 | .303 | 1.004 | .453 | .967 |
|  | [-2.778, 1.470] | [.049, 1.940] | [-1.013, 1.311] | [-1.729, 1.037] | [-.087, 1.188] | [-3.000,2.075] | [.125, 1.311] | [-.999,2.939] | [.169, 1.946] | [-2.403, 1.388] |
|  | (.546) | (.039) | (.802) | (.624) | (.090) | (.721) | (.018) | (.334) | (.020) | (.599) |
| Observations | 2967 | 2973 | 2969 | 2970 | 2976 | 2973 | 2972 | 2967 | 2970 | 2975 |
| Pseudo-R^2^ |  |  |  |  |  |  |  |  |  |  |
| Model χ^2^ |  |  |  |  |  |  |  |  |  |  |
| d.f. | 88 | 88 | 88 | 88 | 88 | 88 | 88 | 88 | 88 | 88 |
| p-value | 3.28e-48 | 3.74e-57 | 3.54e-13 | 8.85e-87 | 3.41e-39 | 9.15e-58 | 1.47e-133 | 2.08e-69 | .0000264 | 2.66e-34 |

S27 Table. Regression Results, Health Behaviors, Interacting with News Source.

|  | (1) | (2) | (3) | (4) | (5) | (6) | (7) | (8) | (9) |
| --- | --- | --- | --- | --- | --- | --- | --- | --- | --- |
|  | washhands | boughtsanitizer | visitdoctor | changedtravel | avoidcontact | avoidgatherings | soughtinfo | selfquarantine | changehealth |
| main |  |  |  |  |  |  |  |  |  |
| Republican | -.981 | -.323 | .485 | -.265 | -.859 | -.878 | -.861 | -.582 | -1.271 |
|  | .182 | .127 | .253 | .139 | .132 | .148 | .129 | .134 | .288 |
|  | [-1.338, -.624] | [-.572, -.074] | [-.010, .980] | [-.538, .007] | [-1.118, -.600] | [-1.168, -.588] | [-1.114, -.608] | [-.844, -.320] | [-1.835, -.707] |
|  | (<.001) | (.011) | (.055) | (.056) | (<.001) | (<.001) | (<.001) | (<.001) | (<.001) |
| Other | -.795 | -.351 | -.257 | -.283 | -.481 | -.448 | -.449 | -.353 | -1.283 |
|  | .148 | .098 | .239 | .103 | .104 | .117 | .099 | .100 | .236 |
|  | [-1.085, -.505] | [-.543, -.158] | [-.725, .212] | [-.486, -.081] | [-.685, -.276] | [-.679, -.218] | [-.643, -.255] | [-.549, -.157] | [-1.745, -.821] |
|  | (<.001) | (<.001) | (.284) | (.006) | (<.001) | (<.001) | (<.001) | (<.001) | (<.001) |
| rightnews=1 | -.491 | .945 | .839 | .116 | -.631 | -.429 | -.668 | -.557 | .311 |
|  | .465 | .353 | .485 | .349 | .325 | .358 | .366 | .347 | 1.074 |
|  | [-1.402, .419] | [.252, 1.637] | [-.112, 1.791] | [-.568, .800] | [-1.268, .007] | [-1.130, .272] | [-1.385, .049] | [-1.237, .124] | [-1.794, 2.416] |
|  | (.290) | (.008) | (.084) | (.739) | (.052) | (.231) | (.068) | (.109) | (.772) |
| Republican # rightnews=1 | .714 | -.967 | -1.802 | -.221 | .599 | .692 | .613 | .581 | -.246 |
|  | .506 | .387 | .615 | .390 | .360 | .397 | .398 | .385 | 1.114 |
|  | [-.278, 1.707] | [-1.725, -.209] | [-3.008, -.597] | [-.986, .545] | [-.107, 1.305] | [-.086, 1.470] | [-.167, 1.393] | [-.173, 1.335] | [-2.430, 1.937] |
|  | (.158) | (.012) | (.003) | (.572) | (.096) | (.081) | (.123) | (.131) | (.825) |
| Other # rightnews=1 | -.069 | -.933 | -1.057 | -.339 | -.152 | -.179 | -.008 | .144 | -.762 |
|  | .503 | .395 | .695 | .397 | .367 | .403 | .407 | .395 | 1.103 |
|  | [-1.054, .917] | [-1.708, -.158] | [-2.419, .304] | [-1.118, .440] | [-.872, .568] | [-.969, .611] | [-.806, .791] | [-.630, .919] | [-2.925, 1.400] |
|  | (.891) | (.018) | (.128) | (.394) | (.679) | (.657) | (.985) | (.715) | (.490) |
| 35- | .080 | .222 | .014 | -.232 | .029 | -.022 | -.011 | -.179 | -.257 |
|  | .158 | .117 | .260 | .122 | .118 | .131 | .119 | .118 | .242 |
|  | [-.230, .390] | [-.007, .451] | [-.497, .524] | [-.472, .007] | [-.203, .260] | [-.278, .234] | [-.243, .222] | [-.411, .052] | [-.731, .218] |
|  | (.613) | (.057) | (.957) | (.057) | (.808) | (.865) | (.929) | (.128) | (.289) |
| 50- | .244 | .227 | -.106 | -.301 | .139 | .355 | -.105 | -.311 | -.262 |
|  | .154 | .111 | .266 | .115 | .113 | .129 | .111 | .113 | .231 |
|  | [-.057, .545] | [.009, .444] | [-.627, .416] | [-.527, -.075] | [-.083, .360] | [.102, .609] | [-.323, .113] | [-.532, -.090] | [-.715, .191] |
|  | (.113) | (.041) | (.692) | (.009) | (.220) | (.006) | (.344) | (.006) | (.257) |
| 70- | .612 | .304 | -.184 | -.085 | .600 | 1.035 | -.252 | .315 | .456 |
|  | .212 | .140 | .340 | .147 | .153 | .185 | .140 | .140 | .341 |
|  | [.195, 1.028] | [.030, .578] | [-.851, .482] | [-.373, .204] | [.300, .900] | [.672, 1.398] | [-.526, .021] | [.041, .590] | [-.212, 1.124] |
|  | (.004) | (.029) | (.588) | (.565) | (<.001) | (<.001) | (.071) | (.024) | (.181) |
| gender | .326 | .070 | -.089 | -.076 | .001 | .241 | .135 | .263 | .666 |
|  | .111 | .080 | .184 | .085 | .083 | .094 | .081 | .083 | .162 |
|  | [.108, .544] | [-.086, .226] | [-.450, .271] | [-.243, .092] | [-.162, .164] | [.058, .425] | [-.023, .294] | [.101, .426] | [.348, .983] |
|  | (.003) | (.377) | (.627) | (.375) | (.989) | (.010) | (.093) | (.002) | (<.001) |
| white | -.068 | -.347 | -.631 | -.168 | .219 | .306 | .368 | .009 | .112 |
|  | .137 | .094 | .210 | .102 | .098 | .111 | .097 | .098 | .200 |
|  | [-.336, .200] | [-.531, -.163] | [-1.042, -.220] | [-.368, .032] | [.027, .411] | [.088, .523] | [.178, .557] | [-.184, .202] | [-.280, .505] |
|  | (.619) | (<.001) | (.003) | (.099) | (.026) | (.006) | (<.001) | (.925) | (.575) |
| married | -.009 | .200 | .344 | .224 | .200 | .003 | .127 | .062 | .386 |
|  | .122 | .087 | .215 | .093 | .092 | .104 | .088 | .090 | .172 |
|  | [-.248, .229] | [.029, .371] | [-.076, .765] | [.041, .407] | [.021, .380] | [-.201, .207] | [-.046, .301] | [-.115, .239] | [.049, .723] |
|  | (.939) | (.022) | (.108) | (.016) | (.029) | (.975) | (.150) | (.495) | (.025) |
| High school graduate | -.111 | .036 | -.066 | .572 | -.572 | -.213 | .012 | -.005 | -.096 |
|  | .292 | .233 | .500 | .313 | .248 | .257 | .236 | .241 | .370 |
|  | [-.683, .462] | [-.420, .492] | [-1.046, .913] | [-.042, 1.186] | [-1.058, -.086] | [-.717, .292] | [-.451, .475] | [-.478, .467] | [-.820, .628] |
|  | (.704) | (.878) | (.894) | (.068) | (.021) | (.408) | (.960) | (.982) | (.795) |
| Some college | .185 | -.028 | -.503 | 1.034 | -.299 | .140 | .585 | .069 | .393 |
|  | .299 | .237 | .522 | .314 | .252 | .263 | .240 | .246 | .385 |
|  | [-.401, .771] | [-.492, .436] | [-1.526, .519] | [.419, 1.650] | [-.792, .195] | [-.377, .656] | [.115, 1.055] | [-.413, .551] | [-.361, 1.147] |
|  | (.536) | (.905) | (.335) | (.001) | (.236) | (.596) | (.015) | (.779) | (.307) |
| 2-year | .119 | .046 | -.244 | .806 | -.354 | .083 | .530 | -.049 | .012 |
|  | .317 | .249 | .545 | .326 | .263 | .278 | .252 | .259 | .406 |
|  | [-.501, .739] | [-.443, .535] | [-1.312, .824] | [.167, 1.445] | [-.870, .161] | [-.462, .628] | [.036, 1.023] | [-.557, .459] | [-.783, .807] |
|  | (.707) | (.853) | (.655) | (.013) | (.178) | (.766) | (.036) | (.849) | (.977) |
| 4-year | .594 | -.109 | .250 | 1.174 | -.214 | .341 | .655 | .119 | 1.223 |
|  | .319 | .242 | .524 | .318 | .258 | .273 | .245 | .251 | .447 |
|  | [-.031, 1.220] | [-.584, .366] | [-.777, 1.278] | [.551, 1.796] | [-.719, .291] | [-.195, .876] | [.174, 1.135] | [-.372, .611] | [.347, 2.099] |
|  | (.063) | (.652) | (.633) | (<.001) | (.406) | (.213) | (.008) | (.634) | (.006) |
| Post-grad | .505 | -.064 | .423 | 1.616 | .125 | .591 | 1.005 | .289 | 1.059 |
|  | .346 | .254 | .570 | .325 | .276 | .300 | .261 | .261 | .498 |
|  | [-.172, 1.182] | [-.562, .434] | [-.694, 1.539] | [.980, 2.252] | [-.417, .667] | [.002, 1.179] | [.492, 1.517] | [-.223, .801] | [.083, 2.036] |
|  | (.144) | (.802) | (.458) | (<.001) | (.651) | (.049) | (<.001) | (.269) | (.033) |
| income=2 | -.123 | -.168 | -1.306 | -.039 | -.022 | -.155 | .094 | -.275 | .042 |
|  | .297 | .224 | .498 | .260 | .231 | .248 | .227 | .228 | .389 |
|  | [-.706, .460] | [-.607, .270] | [-2.282, -.329] | [-.549, .472] | [-.474, .430] | [-.641, .331] | [-.351, .538] | [-.723, .173] | [-.720, .805] |
|  | (.679) | (.452) | (.009) | (.882) | (.925) | (.531) | (.679) | (.229) | (.913) |
| income=3 | -.246 | -.237 | -.831 | -.131 | -.173 | .018 | .227 | -.563 | -.009 |
|  | .283 | .218 | .410 | .253 | .222 | .242 | .216 | .221 | .362 |
|  | [-.801, .308] | [-.664, .191] | [-1.634, -.027] | [-.627, .365] | [-.608, .262] | [-.456, .493] | [-.196, .651] | [-.997, -.129] | [-.720, .701] |
|  | (.384) | (.278) | (.043) | (.604) | (.435) | (.939) | (.293) | (.011) | (.979) |
| income=4 | .127 | .261 | -.957 | .010 | .156 | .332 | .452 | -.565 | .554 |
|  | .299 | .219 | .423 | .257 | .230 | .255 | .221 | .227 | .423 |
|  | [-.460, .714] | [-.168, .690] | [-1.786, -.128] | [-.494, .514] | [-.295, .608] | [-.167, .831] | [.019, .886] | [-1.010, -.120] | [-.274, 1.382] |
|  | (.671) | (.233) | (.024) | (.968) | (.498) | (.192) | (.041) | (.013) | (.190) |
| income=5 | .262 | -.085 | -.896 | -.228 | -.076 | .242 | .534 | -.578 | .116 |
|  | .306 | .227 | .451 | .264 | .232 | .256 | .229 | .231 | .388 |
|  | [-.337, .861] | [-.531, .361] | [-1.780, -.011] | [-.745, .290] | [-.532, .380] | [-.260, .743] | [.085, .982] | [-1.030, -.125] | [-.644, .876] |
|  | (.392) | (.709) | (.047) | (.389) | (.743) | (.345) | (.020) | (.012) | (.765) |
| income=6 | .356 | -<.001 | -.794 | -.039 | .240 | .476 | .321 | -.513 | .803 |
|  | .323 | .228 | .438 | .260 | .243 | .269 | .231 | .234 | .480 |
|  | [-.277, .988] | [-.448, .447] | [-1.652, .064] | [-.549, .471] | [-.236, .716] | [-.051, 1.003] | [-.132, .775] | [-.972, -.053] | [-.138, 1.744] |
|  | (.270) | (.999) | (.070) | (.880) | (.323) | (.077) | (.164) | (.029) | (.094) |
| income=7 | .202 | -.034 | -.729 | .552 | -.016 | .414 | .503 | -.679 | .353 |
|  | .338 | .244 | .425 | .269 | .254 | .281 | .244 | .255 | .480 |
|  | [-.461, .865] | [-.513, .444] | [-1.562, .104] | [.024, 1.079] | [-.513, .481] | [-.138, .966] | [.025, .980] | [-1.179, -.179] | [-.588, 1.294] |
|  | (.551) | (.889) | (.086) | (.040) | (.948) | (.141) | (.039) | (.008) | (.462) |
| income=8 | -.036 | -.347 | -1.413 | .309 | -.133 | .231 | .060 | -.346 | .218 |
|  | .327 | .240 | .524 | .263 | .245 | .272 | .239 | .241 | .451 |
|  | [-.677, .605] | [-.818, .123] | [-2.439, -.386] | [-.207, .824] | [-.614, .348] | [-.303, .764] | [-.408, .528] | [-.818, .127] | [-.666, 1.103] |
|  | (.912) | (.148) | (.007) | (.241) | (.587) | (.397) | (.802) | (.152) | (.628) |
| income=9 | .718 | .253 | -1.264 | .281 | .068 | .227 | .487 | -.682 | .794 |
|  | .355 | .233 | .495 | .261 | .250 | .272 | .239 | .242 | .485 |
|  | [.022, 1.414] | [-.203, .709] | [-2.233, -.295] | [-.230, .793] | [-.423, .558] | [-.307, .760] | [.019, .955] | [-1.156, -.208] | [-.157, 1.745] |
|  | (.043) | (.277) | (.011) | (.281) | (.786) | (.405) | (.041) | (.005) | (.102) |
| income=10 | .249 | -.124 | -1.318 | .416 | .253 | .383 | .117 | -.649 | .732 |
|  | .344 | .247 | .498 | .269 | .257 | .282 | .247 | .253 | .505 |
|  | [-.426, .924] | [-.608, .359] | [-2.293, -.343] | [-.111, .942] | [-.250, .756] | [-.169, .936] | [-.368, .603] | [-1.145, -.154] | [-.258, 1.722] |
|  | (.470) | (.614) | (.008) | (.122) | (.324) | (.174) | (.635) | (.010) | (.147) |
| income=11 | .118 | .192 | -1.443 | .519 | .038 | .227 | .295 | -.498 | -.180 |
|  | .350 | .250 | .516 | .272 | .260 | .287 | .252 | .252 | .461 |
|  | [-.568, .804] | [-.298, .681] | [-2.455, -.431] | [-.014, 1.051] | [-.471, .547] | [-.335, .788] | [-.198, .788] | [-.991, -.005] | [-1.084, .723] |
|  | (.736) | (.443) | (.005) | (.056) | (.884) | (.429) | (.240) | (.048) | (.695) |
| income=12 | .812 | .195 | -1.158 | .505 | .285 | .311 | .525 | -.477 | .581 |
|  | .456 | .275 | .580 | .295 | .304 | .332 | .291 | .282 | .638 |
|  | [-.082, 1.705] | [-.344, .734] | [-2.294, -.022] | [-.073, 1.082] | [-.310, .880] | [-.339, .961] | [-.046, 1.096] | [-1.029, .076] | [-.670, 1.832] |
|  | (.075) | (.479) | (.046) | (.087) | (.348) | (.349) | (.072) | (.091) | (.363) |
| income=13 | .493 | .049 | <.001 | .975 | .355 | .860 | 1.196 | -.278 | .800 |
|  | .593 | .388 | . | .404 | .428 | .538 | .438 | .387 | 1.005 |
|  | [-.669, 1.655] | [-.711, .809] | [.000, .000] | [.182, 1.768] | [-.485, 1.195] | [-.195, 1.915] | [.337, 2.054] | [-1.036, .480] | [-1.170, 2.770] |
|  | (.406) | (.899) | (.) | (.016) | (.407) | (.110) | (.006) | (.472) | (.426) |
| income=14 | -.157 | .339 | -.704 | 1.435 | .602 | .723 | .317 | -.149 | <.001 |
|  | .632 | .398 | .662 | .440 | .507 | .573 | .413 | .397 | . |
|  | [-1.396, 1.082] | [-.440, 1.119] | [-2.001, .593] | [.572, 2.298] | [-.392, 1.596] | [-.399, 1.845] | [-.494, 1.127] | [-.928, .629] | [.000, .000] |
|  | (.804) | (.393) | (.287) | (.001) | (.235) | (.207) | (.443) | (.707) | (.) |
| income=15 | <.001 | .762 | .389 | 1.169 | <.001 | <.001 | <.001 | .912 | <.001 |
|  | . | 1.249 | 1.144 | 1.481 | . | . | . | 1.651 | . |
|  | [.000, .000] | [-1.686, 3.210] | [-1.853, 2.630] | [-1.733, 4.071] | [.000, .000] | [.000, .000] | [.000, .000] | [-2.324, 4.147] | [.000, .000] |
|  | (.) | (.542) | (.734) | (.430) | (.) | (.) | (.) | (.581) | (.) |
| income=16 | <.001 | 1.744 | -.648 | .865 | .988 | .504 | .861 | -.512 | <.001 |
|  | . | .661 | 1.218 | .535 | .835 | .828 | .681 | .571 | . |
|  | [.000, .000] | [.449, 3.039] | [-3.035, 1.740] | [-.183, 1.914] | [-.648, 2.623] | [-1.119, 2.126] | [-.473, 2.196] | [-1.632, .607] | [.000, .000] |
|  | (.) | (.008) | (.595) | (.106) | (.237) | (.543) | (.206) | (.370) | (.) |
| income=17 | -.061 | -.042 | -1.783 | .103 | -.058 | .312 | .144 | -.440 | -.022 |
|  | .283 | .210 | .462 | .239 | .215 | .239 | .211 | .212 | .367 |
|  | [-.614, .493] | [-.453, .369] | [-2.689, -.877] | [-.365, .571] | [-.480, .364] | [-.156, .780] | [-.269, .557] | [-.854, -.025] | [-.741, .696] |
|  | (.830) | (.842) | (<.001) | (.665) | (.788) | (.192) | (.494) | (.038) | (.952) |
| Rural Code=2 | -.137 | -.136 | -.356 | .017 | .191 | .120 | -.013 | -.054 | -.426 |
|  | .148 | .106 | .246 | .113 | .110 | .126 | .105 | .108 | .214 |
|  | [-.427, .153] | [-.343, .071] | [-.839, .126] | [-.204, .237] | [-.024, .406] | [-.126, .366] | [-.218, .191] | [-.266, .158] | [-.846, -.007] |
|  | (.355) | (.198) | (.148) | (.882) | (.082) | (.339) | (.897) | (.616) | (.046) |
| Rural Code=3 | -.090 | -.129 | -.357 | .035 | .187 | -.157 | .003 | -.202 | -.410 |
|  | .200 | .145 | .388 | .152 | .150 | .167 | .141 | .151 | .265 |
|  | [-.482, .303] | [-.413, .154] | [-1.118, .404] | [-.263, .333] | [-.107, .482] | [-.483, .170] | [-.273, .278] | [-.499, .094] | [-.929, .109] |
|  | (.654) | (.371) | (.358) | (.818) | (.212) | (.347) | (.984) | (.181) | (.121) |
| Rural Code=4 | -.601 | .066 | .021 | -.241 | -.141 | .002 | -.312 | -.565 | -.570 |
|  | .259 | .198 | .375 | .225 | .210 | .239 | .190 | .225 | .374 |
|  | [-1.108, -.094] | [-.323, .454] | [-.715, .757] | [-.682, .200] | [-.552, .270] | [-.466, .469] | [-.685, .061] | [-1.006, -.123] | [-1.304, .164] |
|  | (.020) | (.741) | (.955) | (.284) | (.500) | (.995) | (.101) | (.012) | (.128) |
| Rural Code=5 | -.351 | .017 | <.001 | .651 | .349 | .448 | -.316 | -.615 | -.778 |
|  | .393 | .303 | . | .337 | .330 | .419 | .301 | .333 | .574 |
|  | [-1.121, .419] | [-.576, .610] | [.000, .000] | [-.009, 1.310] | [-.298, .996] | [-.374, 1.270] | [-.906, .274] | [-1.268, .038] | [-1.903, .347] |
|  | (.372) | (.956) | (.) | (.053) | (.290) | (.286) | (.294) | (.065) | (.176) |
| Rural Code=6 | -.415 | .028 | -.993 | -.019 | .025 | -.251 | -.073 | -.026 | -.702 |
|  | .254 | .195 | .637 | .221 | .202 | .218 | .203 | .206 | .357 |
|  | [-.913, .083] | [-.355, .410] | [-2.242, .256] | [-.451, .413] | [-.370, .421] | [-.678, .175] | [-.472, .325] | [-.430, .378] | [-1.402, -.001] |
|  | (.103) | (.887) | (.119) | (.931) | (.901) | (.248) | (.718) | (.898) | (.050) |
| Rural Code=7 | -.735 | <.001 | .153 | .053 | -.334 | -.132 | -.102 | -.401 | -.184 |
|  | .337 | .274 | .567 | .305 | .268 | .294 | .297 | .317 | .503 |
|  | [-1.395, -.074] | [-.537, .538] | [-.957, 1.264] | [-.545, .650] | [-.859, .190] | [-.709, .445] | [-.685, .481] | [-1.022, .220] | [-1.170, .803] |
|  | (.029) | (.999) | (.787) | (.863) | (.212) | (.655) | (.731) | (.206) | (.715) |
| Rural Code=8 | -.743 | -.097 | <.001 | -1.244 | -.692 | -.496 | -.716 | -.932 | -1.186 |
|  | .619 | .539 | . | .842 | .538 | .605 | .563 | .723 | .798 |
|  | [-1.955, .470] | [-1.154, .960] | [.000, .000] | [-2.894, .407] | [-1.747, .363] | [-1.681, .690] | [-1.820, .387] | [-2.349, .485] | [-2.750, .378] |
|  | (.230) | (.857) | (.) | (.140) | (.199) | (.413) | (.203) | (.197) | (.137) |
| Rural Code=9 | -.936 | -.482 | -.039 | -.250 | -.167 | -.120 | .227 | -.083 | -.083 |
|  | .553 | .479 | .975 | .614 | .442 | .511 | .460 | .457 | .870 |
|  | [-2.020, .149] | [-1.421, .457] | [-1.951, 1.872] | [-1.453, .954] | [-1.032, .699] | [-1.122, .882] | [-.676, 1.129] | [-.979, .812] | [-1.789, 1.623] |
|  | (.091) | (.315) | (.968) | (.684) | (.706) | (.815) | (.622) | (.856) | (.924) |
| Alaska | <.001 | .683 | <.001 | 1.395 | -.063 | .981 | -.161 | .946 | <.001 |
|  | . | .786 | . | .762 | .835 | 1.047 | .949 | .841 | . |
|  | [.000, .000] | [-.858, 2.223] | [.000, .000] | [-.098, 2.888] | [-1.701, 1.574] | [-1.070, 3.032] | [-2.021, 1.700] | [-.702, 2.595] | [.000, .000] |
|  | (.) | (.385) | (.) | (.067) | (.939) | (.349) | (.866) | (.261) | (.) |
| Arizona | .374 | -.830 | -.685 | -.069 | .124 | .098 | -.520 | .517 | -.063 |
|  | .497 | .409 | .844 | .446 | .418 | .424 | .436 | .444 | .652 |
|  | [-.601, 1.348] | [-1.631, -.029] | [-2.339, .969] | [-.942, .804] | [-.694, .943] | [-.733, .928] | [-1.375, .335] | [-.354, 1.387] | [-1.340, 1.214] |
|  | (.452) | (.042) | (.417) | (.877) | (.766) | (.818) | (.233) | (.245) | (.923) |
| Arkansas | .844 | -.289 | -.563 | -.055 | .073 | -.034 | -.222 | .422 | .336 |
|  | .590 | .446 | .979 | .500 | .457 | .475 | .481 | .501 | .766 |
|  | [-.313, 2.001] | [-1.164, .585] | [-2.482, 1.356] | [-1.035, .924] | [-.824, .969] | [-.965, .898] | [-1.164, .721] | [-.560, 1.404] | [-1.166, 1.838] |
|  | (.153) | (.517) | (.565) | (.912) | (.874) | (.943) | (.645) | (.400) | (.661) |
| California | .633 | -.103 | -.774 | .327 | .305 | .638 | -.205 | .851 | 1.113 |
|  | .433 | .345 | .685 | .379 | .361 | .368 | .389 | .393 | .603 |
|  | [-.215, 1.481] | [-.779, .573] | [-2.117, .569] | [-.416, 1.069] | [-.401, 1.012] | [-.083, 1.359] | [-.968, .558] | [.081, 1.622] | [-.069, 2.294] |
|  | (.144) | (.765) | (.259) | (.389) | (.397) | (.083) | (.599) | (.030) | (.065) |
| Colorado | 1.940 | .032 | -1.204 | .861 | .731 | .921 | .245 | .713 | 1.056 |
|  | .841 | .455 | 1.173 | .476 | .480 | .530 | .512 | .492 | .924 |
|  | [.293, 3.587] | [-.860, .923] | [-3.503, 1.095] | [-.072, 1.793] | [-.209, 1.672] | [-.117, 1.959] | [-.758, 1.249] | [-.252, 1.678] | [-.756, 2.867] |
|  | (.021) | (.944) | (.305) | (.070) | (.128) | (.082) | (.632) | (.148) | (.253) |
| Connecticut | -.041 | -.067 | <.001 | .207 | .953 | .762 | -.191 | 1.130 | 1.608 |
|  | .619 | .486 | . | .529 | .549 | .582 | .509 | .534 | 1.232 |
|  | [-1.255, 1.172] | [-1.020, .886] | [.000, .000] | [-.829, 1.244] | [-.122, 2.029] | [-.378, 1.903] | [-1.188, .806] | [.082, 2.177] | [-.807, 4.023] |
|  | (.947) | (.890) | (.) | (.695) | (.082) | (.190) | (.707) | (.035) | (.192) |
| Delaware | <.001 | .965 | <.001 | -.169 | <.001 | .671 | .326 | 1.169 | <.001 |
|  | . | .718 | . | .706 | . | .875 | .644 | .682 | . |
|  | [.000, .000] | [-.443, 2.372] | [.000, .000] | [-1.553, 1.215] | [.000, .000] | [-1.043, 2.386] | [-.936, 1.588] | [-.168, 2.506] | [.000, .000] |
|  | (.) | (.179) | (.) | (.811) | (.) | (.443) | (.612) | (.087) | (.) |
| District of Columbia | .577 | -.973 | <.001 | 1.493 | 1.180 | 1.451 | -.400 | 1.602 | -.368 |
|  | 1.054 | .794 | . | .723 | .813 | .992 | .795 | .814 | 1.065 |
|  | [-1.489, 2.643] | [-2.529, .584] | [.000, .000] | [.076, 2.909] | [-.414, 2.775] | [-.493, 3.394] | [-1.957, 1.158] | [.006, 3.198] | [-2.455, 1.719] |
|  | (.584) | (.221) | (.) | (.039) | (.147) | (.143) | (.615) | (.049) | (.730) |
| Florida | .682 | .133 | -.814 | .033 | .429 | .429 | -.296 | .632 | 1.050 |
|  | .440 | .348 | .711 | .385 | .364 | .370 | .392 | .399 | .618 |
|  | [-.181, 1.545] | [-.548, .815] | [-2.208, .580] | [-.722, .788] | [-.285, 1.143] | [-.297, 1.154] | [-1.065, .473] | [-.150, 1.414] | [-.161, 2.260] |
|  | (.122) | (.701) | (.252) | (.932) | (.239) | (.247) | (.451) | (.113) | (.089) |
| Georgia | .393 | -.092 | -.803 | .732 | .394 | .626 | -.437 | .505 | .532 |
|  | .480 | .382 | .793 | .406 | .396 | .410 | .419 | .430 | .660 |
|  | [-.548, 1.335] | [-.840, .656] | [-2.358, .751] | [-.064, 1.527] | [-.383, 1.171] | [-.177, 1.429] | [-1.257, .384] | [-.339, 1.348] | [-.761, 1.825] |
|  | (.413) | (.810) | (.311) | (.072) | (.320) | (.126) | (.297) | (.241) | (.420) |
| Hawaii | .916 | .134 | .222 | -2.107 | -.420 | 1.464 | -.135 | .382 | <.001 |
|  | 1.202 | .703 | 1.299 | 1.106 | .688 | 1.028 | .739 | .737 | . |
|  | [-1.440, 3.272] | [-1.243, 1.511] | [-2.324, 2.769] | [-4.275, .061] | [-1.768, .928] | [-.550, 3.479] | [-1.584, 1.314] | [-1.063, 1.827] | [.000, .000] |
|  | (.446) | (.849) | (.864) | (.057) | (.542) | (.154) | (.855) | (.604) | (.) |
| Idaho | -.456 | -.420 | <.001 | .428 | -.360 | .067 | -1.301 | -.312 | -.687 |
|  | .639 | .580 | . | .577 | .589 | .594 | .628 | .712 | .746 |
|  | [-1.708, .796] | [-1.556, .716] | [.000, .000] | [-.704, 1.559] | [-1.513, .794] | [-1.098, 1.231] | [-2.532, -.070] | [-1.707, 1.082] | [-2.148, .775] |
|  | (.475) | (.469) | (.) | (.459) | (.541) | (.911) | (.038) | (.661) | (.357) |
| Illinois | .164 | -.615 | -1.058 | .541 | .155 | .298 | -.379 | .916 | .614 |
|  | .457 | .384 | .827 | .407 | .388 | .396 | .416 | .418 | .656 |
|  | [-.732, 1.060] | [-1.368, .138] | [-2.679, .564] | [-.257, 1.339] | [-.606, .915] | [-.479, 1.075] | [-1.194, .437] | [.096, 1.736] | [-.671, 1.899] |
|  | (.719) | (.109) | (.201) | (.184) | (.690) | (.452) | (.363) | (.029) | (.349) |
| Indiana | .309 | -.555 | -.916 | .511 | .593 | 1.003 | -.590 | .875 | .412 |
|  | .488 | .433 | .957 | .450 | .438 | .476 | .449 | .451 | .685 |
|  | [-.647, 1.264] | [-1.404, .293] | [-2.792, .959] | [-.371, 1.392] | [-.265, 1.451] | [.069, 1.937] | [-1.470, .290] | [-.010, 1.759] | [-.929, 1.754] |
|  | (.527) | (.200) | (.338) | (.256) | (.176) | (.035) | (.189) | (.053) | (.547) |
| Iowa | 1.682 | .053 | .146 | .172 | -.188 | .615 | -.191 | -.338 | <.001 |
|  | .826 | .489 | 1.036 | .569 | .499 | .573 | .520 | .602 | . |
|  | [.064, 3.301] | [-.906, 1.013] | [-1.884, 2.177] | [-.943, 1.286] | [-1.166, .791] | [-.508, 1.738] | [-1.210, .829] | [-1.518, .842] | [.000, .000] |
|  | (.042) | (.913) | (.888) | (.763) | (.707) | (.283) | (.714) | (.575) | (.) |
| Kansas | 1.727 | -.745 | <.001 | -.108 | .370 | .971 | -.311 | .461 | 1.094 |
|  | 1.087 | .657 | . | .676 | .604 | .725 | .619 | .645 | 1.108 |
|  | [-.403, 3.857] | [-2.032, .542] | [.000, .000] | [-1.432, 1.216] | [-.814, 1.554] | [-.451, 2.392] | [-1.523, .902] | [-.804, 1.726] | [-1.077, 3.265] |
|  | (.112) | (.257) | (.) | (.873) | (.540) | (.181) | (.616) | (.475) | (.323) |
| Kentucky | 1.061 | -.476 | -.637 | -1.270 | .584 | .321 | -.256 | -.061 | .550 |
|  | .643 | .460 | .938 | .659 | .486 | .500 | .485 | .531 | .867 |
|  | [-.198, 2.321] | [-1.377, .424] | [-2.475, 1.201] | [-2.561, .021] | [-.370, 1.537] | [-.659, 1.301] | [-1.207, .694] | [-1.101, .980] | [-1.149, 2.248] |
|  | (.099) | (.300) | (.497) | (.054) | (.230) | (.520) | (.597) | (.909) | (.526) |
| Louisiana | .813 | -.405 | -1.717 | -.539 | .651 | .908 | -.213 | .481 | 1.858 |
|  | .614 | .461 | 1.194 | .545 | .482 | .518 | .485 | .508 | 1.128 |
|  | [-.390, 2.016] | [-1.308, .498] | [-4.057, .623] | [-1.607, .528] | [-.294, 1.596] | [-.106, 1.923] | [-1.163, .737] | [-.514, 1.477] | [-.353, 4.068] |
|  | (.185) | (.379) | (.150) | (.322) | (.177) | (.079) | (.660) | (.343) | (.100) |
| Maine | 1.119 | -1.040 | <.001 | .907 | 1.336 | 1.447 | 1.769 | 1.092 | 1.141 |
|  | .837 | .733 | . | .696 | .741 | .775 | .798 | .682 | 1.036 |
|  | [-.521, 2.759] | [-2.476, .396] | [.000, .000] | [-.456, 2.271] | [-.116, 2.788] | [-.073, 2.966] | [.204, 3.334] | [-.245, 2.429] | [-.890, 3.171] |
|  | (.181) | (.156) | (.) | (.192) | (.071) | (.062) | (.027) | (.110) | (.271) |
| Maryland | 1.053 | -.126 | -1.635 | .050 | .438 | .962 | -.124 | -.112 | .547 |
|  | .727 | .452 | 1.249 | .478 | .470 | .557 | .477 | .545 | .897 |
|  | [-.373, 2.478] | [-1.012, .759] | [-4.083, .813] | [-.887, .987] | [-.482, 1.359] | [-.131, 2.054] | [-1.060, .811] | [-1.180, .956] | [-1.211, 2.304] |
|  | (.148) | (.780) | (.190) | (.917) | (.350) | (.084) | (.795) | (.837) | (.542) |
| Massachusetts | .530 | -.395 | <.001 | .540 | .834 | 1.102 | -.806 | .820 | .834 |
|  | .538 | .436 | . | .450 | .461 | .501 | .460 | .463 | .744 |
|  | [-.525, 1.585] | [-1.249, .459] | [.000, .000] | [-.343, 1.423] | [-.070, 1.738] | [.120, 2.085] | [-1.707, .095] | [-.089, 1.728] | [-.625, 2.293] |
|  | (.325) | (.364) | (.) | (.230) | (.071) | (.028) | (.079) | (.077) | (.263) |
| Michigan | 1.013 | -.234 | -.306 | -.279 | .574 | .550 | -.471 | .452 | 1.447 |
|  | .498 | .375 | .743 | .418 | .391 | .407 | .411 | .428 | .733 |
|  | [.037, 1.989] | [-.969, .502] | [-1.763, 1.150] | [-1.098, .541] | [-.192, 1.341] | [-.248, 1.347] | [-1.275, .334] | [-.387, 1.291] | [.010, 2.883] |
|  | (.042) | (.533) | (.680) | (.505) | (.142) | (.176) | (.251) | (.291) | (.048) |
| Minnesota | 1.060 | -.537 | -1.549 | .383 | .560 | .604 | -.103 | .682 | 1.719 |
|  | .649 | .464 | 1.205 | .481 | .485 | .511 | .509 | .498 | 1.112 |
|  | [-.212, 2.332] | [-1.446, .372] | [-3.911, .813] | [-.559, 1.324] | [-.391, 1.510] | [-.398, 1.605] | [-1.100, .894] | [-.293, 1.657] | [-.460, 3.897] |
|  | (.103) | (.247) | (.199) | (.426) | (.249) | (.237) | (.840) | (.171) | (.122) |
| Mississippi | .745 | -.306 | -.387 | -.620 | .715 | .468 | .220 | 1.152 | 1.798 |
|  | .658 | .508 | .982 | .616 | .537 | .548 | .526 | .555 | 1.226 |
|  | [-.545, 2.036] | [-1.302, .689] | [-2.313, 1.538] | [-1.828, .588] | [-.337, 1.767] | [-.606, 1.542] | [-.811, 1.251] | [.065, 2.240] | [-.605, 4.201] |
|  | (.258) | (.546) | (.694) | (.314) | (.183) | (.393) | (.676) | (.038) | (.142) |
| Missouri | .134 | -.555 | -.555 | .035 | .196 | .882 | -.365 | .207 | .337 |
|  | .482 | .419 | .882 | .451 | .418 | .458 | .446 | .462 | .667 |
|  | [-.810, 1.078] | [-1.377, .266] | [-2.284, 1.173] | [-.849, .918] | [-.623, 1.015] | [-.015, 1.780] | [-1.240, .510] | [-.698, 1.112] | [-.970, 1.644] |
|  | (.781) | (.185) | (.529) | (.939) | (.639) | (.054) | (.413) | (.654) | (.613) |
| Montana | 1.035 | -.339 | <.001 | .389 | .284 | .606 | -.835 | .733 | .643 |
|  | .833 | .685 | . | .671 | .624 | .726 | .664 | .677 | 1.189 |
|  | [-.598, 2.668] | [-1.681, 1.003] | [.000, .000] | [-.926, 1.705] | [-.940, 1.508] | [-.818, 2.029] | [-2.136, .467] | [-.594, 2.059] | [-1.688, 2.974] |
|  | (.214) | (.621) | (.) | (.562) | (.649) | (.404) | (.209) | (.279) | (.589) |
| Nebraska | .803 | -.101 | <.001 | -.113 | .493 | .666 | -.171 | .059 | .041 |
|  | .899 | .649 | . | .820 | .765 | .765 | .722 | .766 | .898 |
|  | [-.958, 2.565] | [-1.372, 1.170] | [.000, .000] | [-1.720, 1.494] | [-1.007, 1.993] | [-.833, 2.166] | [-1.586, 1.244] | [-1.441, 1.560] | [-1.720, 1.802] |
|  | (.371) | (.876) | (.) | (.891) | (.519) | (.384) | (.813) | (.938) | (.963) |
| Nevada | .450 | -.705 | -.771 | -.264 | 1.059 | .741 | -.225 | 1.064 | .738 |
|  | .603 | .458 | .981 | .513 | .513 | .512 | .491 | .486 | .829 |
|  | [-.732, 1.631] | [-1.603, .193] | [-2.694, 1.152] | [-1.269, .741] | [.053, 2.065] | [-.263, 1.745] | [-1.188, .738] | [.113, 2.016] | [-.888, 2.364] |
|  | (.456) | (.124) | (.432) | (.606) | (.039) | (.148) | (.647) | (.028) | (.374) |
| New Hampshire | 1.161 | -.863 | -.467 | -.416 | .063 | .337 | -.261 | -.086 | 1.181 |
|  | .830 | .592 | 1.181 | .630 | .535 | .594 | .562 | .651 | 1.188 |
|  | [-.466, 2.787] | [-2.023, .297] | [-2.782, 1.848] | [-1.652, .820] | [-.986, 1.113] | [-.828, 1.502] | [-1.363, .841] | [-1.361, 1.189] | [-1.147, 3.508] |
|  | (.162) | (.145) | (.693) | (.509) | (.906) | (.570) | (.642) | (.895) | (.320) |
| New Jersey | 1.024 | .161 | -.716 | .284 | .603 | .802 | -.231 | .282 | 1.462 |
|  | .582 | .407 | .863 | .441 | .430 | .464 | .440 | .460 | .881 |
|  | [-.117, 2.165] | [-.638, .960] | [-2.408, .976] | [-.580, 1.148] | [-.239, 1.445] | [-.107, 1.711] | [-1.093, .631] | [-.620, 1.184] | [-.265, 3.188] |
|  | (.079) | (.693) | (.407) | (.520) | (.160) | (.084) | (.599) | (.540) | (.097) |
| New Mexico | .902 | -.521 | -1.047 | .241 | .589 | -.179 | .288 | .659 | 1.064 |
|  | .702 | .509 | 1.158 | .551 | .576 | .535 | .561 | .550 | .984 |
|  | [-.474, 2.278] | [-1.518, .477] | [-3.317, 1.222] | [-.838, 1.321] | [-.539, 1.717] | [-1.227, .869] | [-.811, 1.387] | [-.420, 1.737] | [-.864, 2.993] |
|  | (.199) | (.306) | (.366) | (.661) | (.306) | (.738) | (.608) | (.231) | (.279) |
| New York | .128 | .139 | -.445 | .260 | .416 | .799 | -.186 | .501 | .433 |
|  | .437 | .355 | .694 | .392 | .375 | .391 | .402 | .408 | .610 |
|  | [-.729, .985] | [-.557, .834] | [-1.804, .915] | [-.508, 1.029] | [-.319, 1.152] | [.033, 1.566] | [-.975, .602] | [-.299, 1.301] | [-.762, 1.629] |
|  | (.770) | (.696) | (.522) | (.507) | (.267) | (.041) | (.644) | (.220) | (.477) |
| North Carolina | .687 | .071 | -.409 | .277 | .472 | .688 | .013 | .279 | .812 |
|  | .494 | .384 | .792 | .423 | .403 | .428 | .432 | .443 | .707 |
|  | [-.282, 1.656] | [-.681, .824] | [-1.961, 1.143] | [-.551, 1.106] | [-.317, 1.262] | [-.150, 1.526] | [-.834, .860] | [-.590, 1.148] | [-.574, 2.198] |
|  | (.165) | (.852) | (.606) | (.512) | (.241) | (.108) | (.976) | (.530) | (.251) |
| North Dakota | .808 | 1.446 | <.001 | -.161 | .119 | 1.490 | -.023 | .747 | .123 |
|  | 1.070 | .924 | . | .950 | .767 | 1.098 | .737 | .716 | 1.088 |
|  | [-1.289, 2.905] | [-.364, 3.257] | [.000, .000] | [-2.024, 1.702] | [-1.384, 1.623] | [-.662, 3.642] | [-1.468, 1.422] | [-.656, 2.151] | [-2.010, 2.256] |
|  | (.450) | (.117) | (.) | (.866) | (.876) | (.175) | (.975) | (.297) | (.910) |
| Ohio | .847 | -.166 | -.722 | .278 | .477 | .876 | -.021 | .717 | 1.127 |
|  | .489 | .376 | .776 | .415 | .393 | .419 | .421 | .419 | .705 |
|  | [-.112, 1.805] | [-.903, .572] | [-2.242, .798] | [-.535, 1.091] | [-.293, 1.247] | [.054, 1.697] | [-.846, .805] | [-.104, 1.537] | [-.254, 2.508] |
|  | (.084) | (.660) | (.352) | (.503) | (.225) | (.037) | (.961) | (.087) | (.110) |
| Oklahoma | .424 | .355 | .364 | .410 | .685 | .586 | -.343 | .545 | 1.721 |
|  | .620 | .524 | .839 | .550 | .572 | .588 | .540 | .555 | 1.192 |
|  | [-.791, 1.639] | [-.671, 1.381] | [-1.281, 2.009] | [-.668, 1.487] | [-.436, 1.805] | [-.566, 1.739] | [-1.402, .716] | [-.543, 1.633] | [-.616, 4.058] |
|  | (.494) | (.498) | (.664) | (.456) | (.231) | (.319) | (.525) | (.326) | (.149) |
| Oregon | .138 | -.588 | -1.860 | .319 | .366 | .687 | -.143 | .738 | .331 |
|  | .533 | .427 | 1.238 | .448 | .431 | .483 | .467 | .458 | .752 |
|  | [-.906, 1.181] | [-1.425, .249] | [-4.287, .567] | [-.559, 1.197] | [-.479, 1.212] | [-.259, 1.634] | [-1.057, .772] | [-.160, 1.637] | [-1.143, 1.805] |
|  | (.796) | (.169) | (.133) | (.476) | (.396) | (.155) | (.760) | (.107) | (.660) |
| Pennsylvania | .894 | -.488 | -.901 | .190 | .472 | .858 | -.373 | .520 | 1.065 |
|  | .453 | .356 | .739 | .389 | .371 | .385 | .396 | .405 | .625 |
|  | [.006, 1.782] | [-1.186, .211] | [-2.349, .548] | [-.572, .951] | [-.255, 1.200] | [.104, 1.611] | [-1.149, .404] | [-.274, 1.315] | [-.160, 2.289] |
|  | (.048) | (.171) | (.223) | (.626) | (.203) | (.026) | (.347) | (.199) | (.088) |
| Rhode Island | .748 | 1.331 | .565 | .775 | .956 | .551 | -.417 | .629 | .012 |
|  | 1.123 | .839 | 1.318 | .857 | .829 | .822 | .814 | .803 | 1.103 |
|  | [-1.454, 2.949] | [-.314, 2.976] | [-2.019, 3.148] | [-.904, 2.454] | [-.669, 2.582] | [-1.061, 2.163] | [-2.013, 1.179] | [-.944, 2.202] | [-2.149, 2.174] |
|  | (.506) | (.113) | (.668) | (.366) | (.249) | (.503) | (.609) | (.433) | (.991) |
| South Carolina | .712 | .057 | -1.472 | .205 | .911 | 1.124 | .152 | .650 | .739 |
|  | .562 | .434 | 1.216 | .488 | .483 | .520 | .474 | .475 | .745 |
|  | [-.390, 1.814] | [-.793, .906] | [-3.856, .912] | [-.751, 1.161] | [-.036, 1.857] | [.104, 2.143] | [-.777, 1.080] | [-.281, 1.580] | [-.721, 2.199] |
|  | (.205) | (.896) | (.226) | (.675) | (.059) | (.031) | (.749) | (.171) | (.321) |
| South Dakota | .105 | -1.861 | <.001 | -.689 | -.601 | .534 | -.906 | <.001 | -.275 |
|  | .891 | 1.095 | . | 1.016 | .757 | .839 | .819 | . | 1.053 |
|  | [-1.641, 1.852] | [-4.007, .284] | [.000, .000] | [-2.680, 1.302] | [-2.085, .882] | [-1.110, 2.178] | [-2.512, .700] | [.000, .000] | [-2.338, 1.788] |
|  | (.906) | (.089) | (.) | (.497) | (.427) | (.524) | (.269) | (.) | (.794) |
| Tennessee | 1.306 | .398 | -.607 | .247 | .493 | 1.395 | .360 | .647 | 1.612 |
|  | .644 | .423 | .971 | .472 | .449 | .560 | .473 | .475 | .934 |
|  | [.044, 2.568] | [-.431, 1.228] | [-2.510, 1.296] | [-.679, 1.172] | [-.386, 1.373] | [.298, 2.492] | [-.566, 1.286] | [-.284, 1.578] | [-.220, 3.443] |
|  | (.042) | (.347) | (.532) | (.602) | (.272) | (.013) | (.446) | (.173) | (.085) |
| Texas | .360 | -.057 | -.599 | .204 | .092 | .242 | -.621 | .039 | .172 |
|  | .436 | .353 | .694 | .392 | .367 | .374 | .398 | .411 | .580 |
|  | [-.494, 1.214] | [-.748, .635] | [-1.959, .760] | [-.563, .972] | [-.626, .811] | [-.492, .975] | [-1.401, .159] | [-.766, .844] | [-.965, 1.308] |
|  | (.409) | (.872) | (.388) | (.602) | (.801) | (.519) | (.119) | (.925) | (.767) |
| Utah | .500 | -.688 | <.001 | .739 | .191 | .623 | -.158 | .554 | -.110 |
|  | .612 | .552 | . | .519 | .509 | .547 | .515 | .542 | .747 |
|  | [-.699, 1.699] | [-1.770, .394] | [.000, .000] | [-.278, 1.755] | [-.807, 1.188] | [-.449, 1.695] | [-1.167, .850] | [-.509, 1.618] | [-1.574, 1.354] |
|  | (.413) | (.213) | (.) | (.154) | (.708) | (.255) | (.758) | (.307) | (.883) |
| Vermont | <.001 | -.584 | <.001 | 1.061 | 1.007 | -.043 | .330 | -.889 | <.001 |
|  | . | .757 | . | .778 | .919 | .860 | .715 | 1.175 | . |
|  | [.000, .000] | [-2.067, .899] | [.000, .000] | [-.464, 2.585] | [-.795, 2.809] | [-1.728, 1.641] | [-1.071, 1.731] | [-3.191, 1.414] | [.000, .000] |
|  | (.) | (.440) | (.) | (.173) | (.273) | (.960) | (.644) | (.449) | (.) |
| Virginia | .533 | .126 | -.786 | .242 | .252 | .814 | -.120 | .730 | .350 |
|  | .479 | .378 | .773 | .413 | .392 | .425 | .422 | .427 | .642 |
|  | [-.406, 1.472] | [-.615, .866] | [-2.301, .728] | [-.567, 1.051] | [-.516, 1.020] | [-.019, 1.647] | [-.947, .708] | [-.106, 1.566] | [-.910, 1.609] |
|  | (.266) | (.740) | (.309) | (.557) | (.520) | (.055) | (.777) | (.087) | (.586) |
| Washington | .982 | -.364 | -.044 | .329 | .802 | 1.158 | -.070 | .562 | 1.001 |
|  | .547 | .409 | .771 | .440 | .438 | .485 | .444 | .455 | .736 |
|  | [-.090, 2.054] | [-1.165, .438] | [-1.556, 1.468] | [-.533, 1.190] | [-.056, 1.661] | [.207, 2.109] | [-.941, .801] | [-.330, 1.454] | [-.442, 2.444] |
|  | (.073) | (.374) | (.955) | (.455) | (.067) | (.017) | (.876) | (.217) | (.174) |
| West Virginia | .135 | -.194 | -.411 | .333 | -.065 | .301 | -.583 | -.091 | .557 |
|  | .636 | .535 | 1.264 | .596 | .552 | .598 | .539 | .613 | .804 |
|  | [-1.111, 1.381] | [-1.243, .854] | [-2.888, 2.066] | [-.834, 1.500] | [-1.148, 1.017] | [-.871, 1.474] | [-1.639, .474] | [-1.293, 1.110] | [-1.020, 2.133] |
|  | (.832) | (.716) | (.745) | (.576) | (.906) | (.614) | (.280) | (.882) | (.489) |
| Wisconsin | .533 | -.313 | -.278 | .515 | .384 | .720 | -.462 | .135 | 1.236 |
|  | .538 | .418 | .880 | .460 | .435 | .467 | .473 | .485 | .841 |
|  | [-.522, 1.587] | [-1.132, .506] | [-2.002, 1.447] | [-.387, 1.417] | [-.468, 1.236] | [-.195, 1.634] | [-1.388, .465] | [-.815, 1.085] | [-.413, 2.885] |
|  | (.322) | (.454) | (.752) | (.263) | (.377) | (.123) | (.329) | (.781) | (.142) |
| Wyoming | <.001 | 1.045 | 1.681 | .218 | .617 | .349 | .657 | 2.204 | <.001 |
|  | . | 1.405 | 1.148 | 1.088 | 1.176 | 1.186 | 1.187 | 1.348 | . |
|  | [.000, .000] | [-1.709, 3.799] | [-.569, 3.931] | [-1.914, 2.351] | [-1.688, 2.923] | [-1.974, 2.673] | [-1.671, 2.984] | [-.438, 4.846] | [.000, .000] |
|  | (.) | (.457) | (.143) | (.841) | (.600) | (.768) | (.580) | (.102) | (.) |
| N | 2930 | 2982 | 2630 | 2982 | 2965 | 2979 | 2979 | 2972 | 2853 |
| Pseudo-R^2^ | .0827 | .0492 | .0825 | .0811 | .0594 | .0735 | .0764 | .0542 | .144 |
| Model χ^2^ | 192.3 | 178.1 | 118.9 | 268.6 | 194.1 | 211.0 | 276.6 | 19.6 | 198.1 |
| d.f. | 84 | 90 | 73 | 90 | 88 | 89 | 89 | 89 | 81 |
| p-value | <.001 | <.001 | <.001 | <.001 | <.001 | <.001 | <.001 | <.001 | <.001 |

**S9 Fig. Health Behaviors, by News Source.** Estimates are the predicted effects comparing Republicans and Others (unaffiliated or identifying with a third party) to self-identified Democrats for respondents who report watching right-wing news versus those who do not. Confidence intervals are not adjusted for multiple comparisons in this exploratory analysis.

S28 Table. Regression Results, Health Attitudes, Interacting with News Source.

|  | (1) | (2) | (3) | (4) | (5) | (6) | (7) | (8) | (9) |
| --- | --- | --- | --- | --- | --- | --- | --- | --- | --- |
|  | numberdeaths | spendingincrease | notenoughtests | getsick | negativeeconomy | friendsick | cannotreturn | schoolsclosed | hardgetitems |
| Republican | -.422 | -.323 | -.895 | -.520 | -.379 | -.473 | -.436 | -.266 | -.244 |
|  | .068 | .031 | .068 | .060 | .055 | .058 | .062 | .063 | .062 |
|  | [-.556, -.288] | [-.384, -.262] | [-1.029, -.762] | [-.638, -.402] | [-.488, -.270] | [-.587, -.360] | [-.558, -.314] | [-.390, -.143] | [-.365, -.123] |
|  | (<.001) | (<.001) | (<.001) | (<.001) | (<.001) | (<.001) | (<.001) | (<.001) | (<.001) |
| Other | -.283 | -.201 | -.282 | -.292 | -.236 | -.320 | -.298 | -.188 | -.199 |
|  | .051 | .023 | .051 | .047 | .042 | .046 | .047 | .052 | .047 |
|  | [-.383, -.182] | [-.246, -.157] | [-.382, -.182] | [-.385, -.199] | [-.319, -.153] | [-.411, -.229] | [-.391, -.205] | [-.290, -.087] | [-.292, -.107] |
|  | (<.001) | (<.001) | (<.001) | (<.001) | (<.001) | (<.001) | (<.001) | (<.001) | (<.001) |
| rightnews=1 | -.641 | -.284 | -.864 | -.091 | -.150 | -.142 | -.082 | -.081 | .184 |
|  | .181 | .092 | .202 | .164 | .153 | .171 | .166 | .160 | .152 |
|  | [-.996, -.286] | [-.464, -.104] | [-1.261, -.468] | [-.414, .231] | [-.450, .151] | [-.478, .193] | [-.407, .244] | [-.395, .234] | [-.115, .482] |
|  | (<.001) | (.002) | (<.001) | (.579) | (.329) | (.405) | (.623) | (.615) | (.228) |
| Republican # rightnews=1 | .735 | .233 | .832 | .013 | .246 | .034 | .055 | .061 | -.323 |
|  | .197 | .100 | .217 | .181 | .168 | .184 | .182 | .177 | .169 |
|  | [.347, 1.122] | [.037, .430] | [.406, 1.258] | [-.341, .367] | [-.083, .576] | [-.328, .395] | [-.303, .413] | [-.287, .408] | [-.654, .008] |
|  | (<.001) | (.020) | (<.001) | (.942) | (.142) | (.854) | (.764) | (.733) | (.056) |
| Other # rightnews=1 | .650 | .062 | .269 | -.265 | .009 | -.216 | -.190 | -.036 | -.443 |
|  | .200 | .103 | .221 | .183 | .171 | .187 | .183 | .180 | .169 |
|  | [.258, 1.042] | [-.140, .264] | [-.164, .702] | [-.624, .093] | [-.325, .344] | [-.583, .150] | [-.549, .170] | [-.388, .316] | [-.775, -.111] |
|  | (.001) | (.547) | (.224) | (.147) | (.958) | (.247) | (.301) | (.842) | (.009) |
| 35- | -.036 | .048 | .092 | .073 | -.039 | .078 | -.128 | .041 | .019 |
|  | .061 | .028 | .063 | .056 | .051 | .055 | .056 | .059 | .055 |
|  | [-.156, .085] | [-.007, .104] | [-.031, .215] | [-.037, .183] | [-.139, .061] | [-.030, .186] | [-.238, -.019] | [-.075, .158] | [-.089, .127] |
|  | (.563) | (.089) | (.144) | (.191) | (.445) | (.157) | (.021) | (.489) | (.731) |
| 50- | .149 | .048 | .180 | -.023 | .052 | .010 | -.196 | -.094 | -.169 |
|  | .059 | .027 | .058 | .053 | .048 | .051 | .053 | .057 | .052 |
|  | [.033, .265] | [-.004, .100] | [.065, .294] | [-.127, .081] | [-.042, .146] | [-.090, .111] | [-.299, -.093] | [-.206, .019] | [-.271, -.066] |
|  | (.012) | (.070) | (.002) | (.669) | (.276) | (.840) | (<.001) | (.102) | (.001) |
| 70- | .327 | .098 | .182 | .034 | -.033 | .028 | -.260 | -.532 | -.378 |
|  | .073 | .032 | .068 | .065 | .060 | .063 | .065 | .068 | .065 |
|  | [.184, .470] | [.035, .160] | [.049, .316] | [-.094, .161] | [-.150, .084] | [-.095, .151] | [-.387, -.132] | [-.665, -.398] | [-.505, -.251] |
|  | (<.001) | (.002) | (.007) | (.605) | (.578) | (.653) | (<.001) | (<.001) | (<.001) |
| gender | .050 | -.036 | -.011 | .131 | -.047 | .118 | .019 | .042 | .130 |
|  | .041 | .019 | .041 | .038 | .034 | .037 | .039 | .041 | .038 |
|  | [-.031, .130] | [-.072, .001] | [-.091, .070] | [.056, .205] | [-.115, .020] | [.046, .190] | [-.058, .095] | [-.038, .123] | [.056, .204] |
|  | (.225) | (.058) | (.798) | (.001) | (.172) | (.001) | (.631) | (.306) | (.001) |
| white | -.048 | .070 | .244 | .033 | .110 | .008 | .137 | -.076 | .033 |
|  | .051 | .023 | .052 | .047 | .042 | .045 | .047 | .049 | .046 |
|  | [-.148, .051] | [.025, .116] | [.142, .345] | [-.059, .126] | [.027, .192] | [-.080, .096] | [.046, .229] | [-.172, .021] | [-.058, .123] |
|  | (.341) | (.003) | (<.001) | (.479) | (.009) | (.859) | (.003) | (.124) | (.480) |
| married | .007 | -.004 | -.093 | .118 | .091 | .098 | .034 | .074 | .112 |
|  | .046 | .021 | .046 | .041 | .037 | .040 | .042 | .045 | .041 |
|  | [-.083, .097] | [-.045, .037] | [-.183, -.002] | [.037, .199] | [.018, .165] | [.019, .177] | [-.048, .115] | [-.014, .161] | [.032, .193] |
|  | (.884) | (.858) | (.045) | (.004) | (.015) | (.016) | (.422) | (.099) | (.006) |
| High school graduate | -.042 | -.065 | -.023 | -.070 | -.091 | -.121 | -.075 | .013 | -.221 |
|  | .143 | .066 | .125 | .122 | .103 | .108 | .120 | .126 | .114 |
|  | [-.322, .238] | [-.195, .066] | [-.269, .223] | [-.310, .170] | [-.293, .110] | [-.333, .091] | [-.311, .161] | [-.235, .260] | [-.445, .002] |
|  | (.768) | (.331) | (.854) | (.566) | (.374) | (.262) | (.535) | (.919) | (.052) |
| Some college | -.080 | -.003 | .215 | -.028 | .010 | -.105 | -.073 | -.022 | -.321 |
|  | .143 | .068 | .127 | .124 | .105 | .110 | .123 | .128 | .116 |
|  | [-.360, .200] | [-.136, .129] | [-.033, .464] | [-.271, .216] | [-.196, .215] | [-.320, .110] | [-.313, .167] | [-.273, .230] | [-.548, -.095] |
|  | (.574) | (.961) | (.090) | (.823) | (.926) | (.338) | (.551) | (.866) | (.005) |
| 2-year | -.013 | .007 | .131 | -.028 | .006 | -.099 | -.113 | -.030 | -.357 |
|  | .149 | .069 | .134 | .128 | .109 | .115 | .128 | .134 | .122 |
|  | [-.306, .279] | [-.129, .143] | [-.131, .393] | [-.280, .224] | [-.208, .220] | [-.324, .127] | [-.364, .138] | [-.293, .234] | [-.596, -.118] |
|  | (.928) | (.921) | (.327) | (.827) | (.955) | (.393) | (.376) | (.825) | (.003) |
| 4-year | -.060 | .013 | .122 | -.069 | .035 | -.074 | -.055 | -.065 | -.329 |
|  | .144 | .068 | .130 | .126 | .107 | .112 | .124 | .130 | .118 |
|  | [-.344, .223] | [-.121, .146] | [-.133, .376] | [-.317, .178] | [-.174, .244] | [-.293, .146] | [-.297, .188] | [-.321, .191] | [-.560, -.097] |
|  | (.676) | (.852) | (.349) | (.582) | (.742) | (.512) | (.658) | (.618) | (.005) |
| Post-grad | -.060 | .029 | .163 | .030 | .108 | -.009 | -.015 | -.004 | -.424 |
|  | .151 | .069 | .137 | .131 | .110 | .116 | .129 | .137 | .124 |
|  | [-.356, .235] | [-.106, .164] | [-.106, .431] | [-.227, .288] | [-.108, .324] | [-.236, .219] | [-.268, .237] | [-.272, .264] | [-.667, -.182] |
|  | (.690) | (.676) | (.235) | (.818) | (.327) | (.941) | (.907) | (.975) | (.001) |
| income=2 | .062 | -.041 | .364 | -.071 | .007 | .002 | -.160 | -.153 | -.112 |
|  | .134 | .057 | .123 | .112 | .104 | .110 | .114 | .116 | .117 |
|  | [-.200, .324] | [-.153, .071] | [.123, .604] | [-.291, .149] | [-.197, .212] | [-.213, .217] | [-.384, .065] | [-.380, .074] | [-.341, .117] |
|  | (.645) | (.470) | (.003) | (.529) | (.944) | (.985) | (.163) | (.186) | (.339) |
| income=3 | .179 | -<.001 | .319 | -.092 | .119 | -.007 | -.109 | -.041 | -.149 |
|  | .129 | .056 | .119 | .108 | .101 | .107 | .109 | .116 | .113 |
|  | [-.074, .432] | [-.109, .109] | [.086, .552] | [-.304, .120] | [-.078, .316] | [-.217, .202] | [-.323, .105] | [-.267, .186] | [-.370, .072] |
|  | (.166) | (.999) | (.007) | (.394) | (.237) | (.945) | (.318) | (.724) | (.186) |
| income=4 | .127 | .025 | .296 | .010 | .176 | .092 | -.087 | -.047 | -.121 |
|  | .130 | .057 | .127 | .109 | .103 | .107 | .112 | .116 | .115 |
|  | [-.127, .381] | [-.087, .137] | [.047, .544] | [-.204, .223] | [-.026, .377] | [-.118, .303] | [-.307, .133] | [-.274, .180] | [-.346, .104] |
|  | (.329) | (.664) | (.020) | (.930) | (.088) | (.391) | (.439) | (.686) | (.292) |
| income=5 | .180 | .031 | .371 | -.058 | .144 | .011 | -.160 | -.034 | -.196 |
|  | .131 | .057 | .126 | .112 | .104 | .108 | .112 | .114 | .114 |
|  | [-.077, .437] | [-.081, .142] | [.125, .617] | [-.277, .160] | [-.060, .348] | [-.200, .223] | [-.380, .059] | [-.258, .190] | [-.420, .027] |
|  | (.170) | (.593) | (.003) | (.601) | (.165) | (.916) | (.152) | (.764) | (.086) |
| income=6 | .229 | .003 | .386 | .014 | .137 | .012 | -.090 | .089 | -.150 |
|  | .134 | .059 | .125 | .112 | .106 | .110 | .115 | .119 | .118 |
|  | [-.033, .492] | [-.113, .118] | [.141, .630] | [-.206, .234] | [-.071, .345] | [-.204, .228] | [-.316, .136] | [-.145, .322] | [-.381, .081] |
|  | (.086) | (.963) | (.002) | (.901) | (.197) | (.914) | (.436) | (.456) | (.203) |
| income=7 | .079 | .003 | .401 | -.075 | .070 | -.015 | -.181 | -.098 | -.285 |
|  | .139 | .061 | .131 | .119 | .112 | .115 | .123 | .130 | .124 |
|  | [-.194, .351] | [-.116, .122] | [.145, .657] | [-.309, .158] | [-.148, .289] | [-.241, .210] | [-.422, .060] | [-.354, .158] | [-.528, -.041] |
|  | (.572) | (.961) | (.002) | (.526) | (.528) | (.894) | (.140) | (.451) | (.022) |
| income=8 | .108 | .022 | .359 | -.258 | .028 | -.211 | -.257 | -.174 | -.356 |
|  | .135 | .060 | .129 | .118 | .109 | .113 | .120 | .125 | .122 |
|  | [-.158, .373] | [-.095, .139] | [.105, .612] | [-.489, -.028] | [-.185, .241] | [-.433, .011] | [-.492, -.022] | [-.419, .070] | [-.594, -.117] |
|  | (.427) | (.714) | (.006) | (.028) | (.796) | (.063) | (.032) | (.163) | (.004) |
| income=9 | .340 | .012 | .468 | -.179 | .139 | -.058 | -.076 | -.106 | -.296 |
|  | .135 | .058 | .130 | .114 | .106 | .112 | .116 | .124 | .117 |
|  | [.075, .605] | [-.103, .126] | [.213, .723] | [-.402, .045] | [-.069, .347] | [-.277, .161] | [-.304, .152] | [-.349, .137] | [-.526, -.066] |
|  | (.012) | (.841) | (<.001) | (.117) | (.189) | (.605) | (.513) | (.392) | (.012) |
| income=10 | .219 | .006 | .485 | -.179 | .186 | -.075 | -.102 | -.108 | -.371 |
|  | .134 | .061 | .133 | .117 | .111 | .115 | .120 | .125 | .125 |
|  | [-.043, .481] | [-.113, .125] | [.223, .746] | [-.408, .050] | [-.033, .404] | [-.301, .152] | [-.337, .133] | [-.354, .138] | [-.615, -.127] |
|  | (.102) | (.918) | (<.001) | (.125) | (.095) | (.518) | (.394) | (.388) | (.003) |
| income=11 | .364 | .003 | .467 | -.072 | .326 | .003 | .074 | -.098 | -.347 |
|  | .139 | .061 | .136 | .123 | .112 | .121 | .123 | .130 | .127 |
|  | [.091, .637] | [-.116, .123] | [.200, .734] | [-.312, .169] | [.106, .546] | [-.234, .241] | [-.168, .317] | [-.353, .157] | [-.595, -.098] |
|  | (.009) | (.954) | (.001) | (.558) | (.004) | (.978) | (.546) | (.450) | (.006) |
| income=12 | .232 | .032 | .355 | -.144 | .223 | .002 | .018 | -.184 | -.422 |
|  | .148 | .063 | .161 | .134 | .119 | .138 | .137 | .149 | .143 |
|  | [-.058, .522] | [-.091, .156] | [.039, .671] | [-.406, .118] | [-.010, .457] | [-.269, .273] | [-.252, .287] | [-.477, .108] | [-.703, -.141] |
|  | (.117) | (.609) | (.028) | (.282) | (.061) | (.989) | (.898) | (.217) | (.003) |
| income=13 | .210 | .063 | .637 | -.260 | .026 | -.125 | -.084 | -.420 | -.646 |
|  | .211 | .087 | .187 | .163 | .178 | .164 | .177 | .214 | .178 |
|  | [-.204, .623] | [-.107, .234] | [.270, 1.004] | [-.580, .060] | [-.323, .375] | [-.447, .198] | [-.430, .263] | [-.840, .000] | [-.994, -.298] |
|  | (.320) | (.467) | (.001) | (.111) | (.883) | (.448) | (.636) | (.050) | (<.001) |
| income=14 | .312 | -.068 | .547 | -.226 | .083 | -.067 | -.185 | -.068 | -.439 |
|  | .204 | .087 | .165 | .211 | .186 | .209 | .224 | .193 | .192 |
|  | [-.087, .712] | [-.239, .103] | [.223, .870] | [-.639, .186] | [-.281, .448] | [-.477, .343] | [-.624, .253] | [-.446, .310] | [-.815, -.064] |
|  | (.125) | (.436) | (.001) | (.282) | (.654) | (.749) | (.408) | (.725) | (.022) |
| income=15 | .283 | .207 | 1.381 | .417 | .682 | .198 | .362 | -.073 | -1.182 |
|  | .770 | .119 | .291 | .561 | .205 | .713 | .472 | .649 | .427 |
|  | [-1.226, 1.792] | [-.027, .441] | [.811, 1.951] | [-.683, 1.517] | [.280, 1.085] | [-1.200, 1.597] | [-.563, 1.287] | [-1.345, 1.199] | [-2.020, -.344] |
|  | (.713) | (.083) | (<.001) | (.457) | (.001) | (.781) | (.443) | (.910) | (.006) |
| income=16 | .511 | .047 | .849 | -.299 | .106 | -.075 | .165 | -.037 | -.464 |
|  | .262 | .128 | .344 | .309 | .264 | .320 | .269 | .306 | .270 |
|  | [-.003, 1.024] | [-.204, .298] | [.175, 1.523] | [-.905, .307] | [-.413, .624] | [-.703, .553] | [-.363, .693] | [-.636, .563] | [-.993, .065] |
|  | (.051) | (.712) | (.014) | (.333) | (.689) | (.814) | (.540) | (.904) | (.085) |
| income=17 | .268 | -.027 | .355 | -.228 | .020 | -.123 | -.224 | -.261 | -.412 |
|  | .125 | .055 | .116 | .105 | .101 | .103 | .107 | .111 | .109 |
|  | [.022, .514] | [-.135, .081] | [.128, .582] | [-.434, -.022] | [-.177, .217] | [-.325, .080] | [-.435, -.014] | [-.479, -.043] | [-.626, -.199] |
|  | (.033) | (.622) | (.002) | (.030) | (.840) | (.234) | (.036) | (.019) | (<.001) |
| Rural Code=2 | .004 | -.010 | -.041 | .002 | -.053 | -.053 | -.035 | -.097 | -.008 |
|  | .054 | .025 | .053 | .051 | .046 | .049 | .051 | .054 | .051 |
|  | [-.103, .110] | [-.060, .040] | [-.145, .064] | [-.098, .102] | [-.143, .036] | [-.148, .043] | [-.134, .065] | [-.202, .008] | [-.108, .092] |
|  | (.945) | (.693) | (.445) | (.967) | (.244) | (.279) | (.494) | (.070) | (.869) |
| Rural Code=3 | -.015 | -.027 | -.106 | .023 | -.040 | .050 | .019 | .022 | .054 |
|  | .076 | .034 | .073 | .068 | .063 | .065 | .067 | .073 | .064 |
|  | [-.164, .134] | [-.094, .040] | [-.250, .037] | [-.110, .156] | [-.164, .083] | [-.077, .177] | [-.113, .150] | [-.121, .166] | [-.072, .180] |
|  | (.844) | (.427) | (.146) | (.734) | (.519) | (.444) | (.781) | (.762) | (.401) |
| Rural Code=4 | -.069 | .021 | .088 | -.136 | -.216 | -.172 | -.138 | -.091 | -.052 |
|  | .106 | .047 | .102 | .092 | .086 | .093 | .095 | .104 | .094 |
|  | [-.276, .139] | [-.071, .113] | [-.113, .288] | [-.316, .045] | [-.385, -.047] | [-.355, .011] | [-.325, .048] | [-.296, .113] | [-.237, .133] |
|  | (.518) | (.655) | (.390) | (.140) | (.012) | (.066) | (.146) | (.380) | (.579) |
| Rural Code=5 | -.172 | -.008 | -.021 | -.151 | -.030 | .038 | -.089 | <.001 | -.018 |
|  | .173 | .065 | .140 | .136 | .137 | .130 | .134 | .158 | .139 |
|  | [-.511, .168] | [-.135, .119] | [-.296, .253] | [-.419, .116] | [-.298, .238] | [-.217, .293] | [-.353, .174] | [-.309, .310] | [-.290, .254] |
|  | (.322) | (.900) | (.879) | (.267) | (.825) | (.769) | (.505) | (.998) | (.895) |
| Rural Code=6 | .123 | -.009 | -.035 | .011 | .019 | -.062 | .002 | .012 | .109 |
|  | .113 | .048 | .102 | .097 | .080 | .096 | .098 | .105 | .098 |
|  | [-.098, .344] | [-.103, .085] | [-.235, .165] | [-.179, .200] | [-.137, .175] | [-.250, .126] | [-.191, .195] | [-.195, .219] | [-.084, .302] |
|  | (.275) | (.847) | (.732) | (.912) | (.815) | (.518) | (.983) | (.909) | (.267) |
| Rural Code=7 | .046 | -.037 | .085 | .075 | -.099 | .069 | .054 | -.030 | .078 |
|  | .142 | .067 | .145 | .126 | .119 | .129 | .133 | .145 | .121 |
|  | [-.233, .325] | [-.168, .095] | [-.200, .370] | [-.172, .322] | [-.332, .134] | [-.184, .323] | [-.207, .316] | [-.314, .254] | [-.159, .314] |
|  | (.747) | (.586) | (.560) | (.551) | (.404) | (.592) | (.683) | (.837) | (.519) |
| Rural Code=8 | .483 | -.004 | -.159 | -.394 | .009 | -.181 | -.067 | -.399 | .119 |
|  | .263 | .127 | .237 | .248 | .199 | .237 | .228 | .253 | .173 |
|  | [-.033, 1.000] | [-.253, .245] | [-.624, .306] | [-.880, .093] | [-.382, .399] | [-.647, .284] | [-.514, .379] | [-.894, .096] | [-.219, .458] |
|  | (.067) | (.974) | (.503) | (.113) | (.966) | (.445) | (.767) | (.114) | (.491) |
| Rural Code=9 | -.130 | -.072 | -.048 | .199 | -.128 | .204 | -.054 | -.090 | -.199 |
|  | .198 | .116 | .205 | .198 | .179 | .184 | .192 | .213 | .225 |
|  | [-.518, .259] | [-.299, .156] | [-.450, .354] | [-.190, .588] | [-.478, .223] | [-.156, .565] | [-.431, .323] | [-.507, .327] | [-.639, .242] |
|  | (.512) | (.538) | (.815) | (.315) | (.474) | (.266) | (.779) | (.673) | (.377) |
| Alaska | .250 | -.031 | .328 | -.016 | -.123 | -.471 | -.510 | .039 | -.273 |
|  | .450 | .261 | .385 | .326 | .299 | .265 | .393 | .435 | .304 |
|  | [-.633, 1.132] | [-.543, .481] | [-.426, 1.083] | [-.656, .624] | [-.709, .463] | [-.991, .048] | [-1.280, .260] | [-.814, .892] | [-.868, .323] |
|  | (.579) | (.906) | (.393) | (.962) | (.681) | (.075) | (.194) | (.929) | (.370) |
| Arizona | -.209 | .076 | .175 | -.015 | -.155 | -.181 | -.114 | -.170 | .050 |
|  | .221 | .100 | .220 | .201 | .169 | .190 | .206 | .196 | .188 |
|  | [-.642, .225] | [-.119, .272] | [-.256, .607] | [-.409, .380] | [-.487, .177] | [-.552, .191] | [-.518, .290] | [-.554, .214] | [-.320, .419] |
|  | (.345) | (.445) | (.426) | (.941) | (.359) | (.341) | (.580) | (.386) | (.792) |
| Arkansas | .138 | .040 | -.007 | .107 | -.256 | -.026 | .036 | .021 | .265 |
|  | .250 | .118 | .252 | .213 | .199 | .203 | .233 | .221 | .210 |
|  | [-.352, .628] | [-.192, .272] | [-.501, .488] | [-.310, .524] | [-.647, .135] | [-.424, .371] | [-.421, .492] | [-.412, .455] | [-.147, .676] |
|  | (.580) | (.733) | (.979) | (.615) | (.199) | (.897) | (.879) | (.924) | (.207) |
| California | -.004 | .088 | .007 | .188 | -.138 | -.041 | -.017 | .010 | .138 |
|  | .203 | .090 | .201 | .175 | .142 | .167 | .184 | .173 | .167 |
|  | [-.402, .394] | [-.088, .264] | [-.387, .401] | [-.155, .532] | [-.417, .142] | [-.368, .286] | [-.379, .345] | [-.329, .350] | [-.188, .465] |
|  | (.985) | (.324) | (.973) | (.283) | (.334) | (.806) | (.927) | (.953) | (.406) |
| Colorado | .195 | .005 | -.034 | .172 | -.369 | -.136 | -.201 | -.062 | -.148 |
|  | .250 | .121 | .257 | .224 | .194 | .209 | .233 | .209 | .215 |
|  | [-.295, .685] | [-.233, .243] | [-.539, .471] | [-.267, .610] | [-.749, .012] | [-.545, .273] | [-.657, .256] | [-.473, .348] | [-.570, .273] |
|  | (.435) | (.967) | (.896) | (.443) | (.057) | (.515) | (.389) | (.767) | (.490) |
| Connecticut | .013 | .263 | .242 | .228 | -.036 | -.028 | .122 | .008 | .028 |
|  | .260 | .116 | .254 | .238 | .206 | .208 | .260 | .244 | .237 |
|  | [-.497, .523] | [.035, .490] | [-.256, .741] | [-.238, .694] | [-.440, .368] | [-.437, .380] | [-.388, .632] | [-.470, .487] | [-.437, .492] |
|  | (.960) | (.023) | (.341) | (.337) | (.862) | (.893) | (.639) | (.973) | (.907) |
| Delaware | .331 | -.050 | -.315 | .147 | -.132 | -.372 | -.164 | .002 | -.319 |
|  | .323 | .154 | .314 | .286 | .230 | .294 | .278 | .316 | .284 |
|  | [-.302, .965] | [-.352, .252] | [-.930, .300] | [-.414, .707] | [-.584, .319] | [-.948, .204] | [-.710, .381] | [-.617, .622] | [-.876, .238] |
|  | (.305) | (.746) | (.316) | (.608) | (.566) | (.205) | (.555) | (.994) | (.262) |
| District of Columbia | .228 | .161 | .359 | .425 | -.089 | -.003 | .094 | -.188 | .423 |
|  | .512 | .137 | .374 | .286 | .313 | .224 | .376 | .281 | .299 |
|  | [-.777, 1.233] | [-.108, .429] | [-.375, 1.092] | [-.136, .986] | [-.703, .524] | [-.442, .437] | [-.642, .831] | [-.739, .364] | [-.163, 1.010] |
|  | (.656) | (.241) | (.338) | (.137) | (.776) | (.990) | (.802) | (.505) | (.157) |
| Florida | .165 | .064 | .065 | .122 | -.219 | -.154 | -.002 | .016 | .110 |
|  | .204 | .090 | .203 | .176 | .147 | .167 | .186 | .178 | .169 |
|  | [-.235, .565] | [-.112, .241] | [-.332, .463] | [-.224, .468] | [-.507, .069] | [-.480, .173] | [-.367, .363] | [-.333, .365] | [-.222, .441] |
|  | (.419) | (.475) | (.747) | (.490) | (.135) | (.357) | (.990) | (.929) | (.517) |
| Georgia | .038 | .061 | .158 | -.024 | -.326 | -.181 | .066 | -.162 | .006 |
|  | .230 | .099 | .214 | .197 | .164 | .186 | .201 | .194 | .184 |
|  | [-.413, .489] | [-.133, .255] | [-.261, .577] | [-.409, .362] | [-.647, -.005] | [-.544, .183] | [-.329, .461] | [-.542, .218] | [-.354, .366] |
|  | (.869) | (.536) | (.459) | (.904) | (.046) | (.331) | (.744) | (.402) | (.973) |
| Hawaii | .012 | .285 | .517 | -.023 | .072 | -.422 | .124 | -.133 | .376 |
|  | .380 | .094 | .377 | .380 | .276 | .284 | .346 | .379 | .349 |
|  | [-.733, .756] | [.101, .469] | [-.221, 1.256] | [-.769, .723] | [-.470, .613] | [-.978, .135] | [-.555, .803] | [-.876, .609] | [-.309, 1.062] |
|  | (.976) | (.002) | (.170) | (.952) | (.795) | (.137) | (.720) | (.725) | (.282) |
| Idaho | .162 | -.108 | -.138 | -.368 | -.185 | -.516 | -.342 | -.188 | -.042 |
|  | .305 | .181 | .335 | .299 | .233 | .282 | .258 | .300 | .263 |
|  | [-.436, .760] | [-.463, .246] | [-.795, .519] | [-.954, .217] | [-.642, .272] | [-1.070, .038] | [-.848, .164] | [-.776, .399] | [-.558, .474] |
|  | (.595) | (.550) | (.680) | (.218) | (.428) | (.068) | (.186) | (.530) | (.873) |
| Illinois | .050 | .113 | .049 | .329 | -.271 | -.130 | -.037 | -.055 | .075 |
|  | .216 | .094 | .212 | .187 | .161 | .182 | .199 | .193 | .178 |
|  | [-.373, .474] | [-.072, .298] | [-.368, .465] | [-.037, .696] | [-.587, .045] | [-.487, .227] | [-.427, .352] | [-.433, .323] | [-.275, .424] |
|  | (.815) | (.231) | (.819) | (.078) | (.093) | (.477) | (.851) | (.774) | (.676) |
| Indiana | -.179 | .080 | .354 | .314 | -.184 | -.020 | -.007 | -.059 | .041 |
|  | .224 | .099 | .228 | .202 | .170 | .196 | .214 | .217 | .197 |
|  | [-.618, .261] | [-.113, .273] | [-.093, .801] | [-.082, .710] | [-.518, .149] | [-.405, .365] | [-.428, .413] | [-.484, .366] | [-.346, .427] |
|  | (.426) | (.416) | (.121) | (.120) | (.279) | (.920) | (.973) | (.785) | (.837) |
| Iowa | .067 | -.018 | -.045 | .458 | -.001 | -.038 | -.027 | .070 | .004 |
|  | .290 | .119 | .283 | .228 | .196 | .223 | .238 | .270 | .231 |
|  | [-.500, .635] | [-.251, .216] | [-.601, .511] | [.010, .905] | [-.386, .384] | [-.476, .399] | [-.495, .440] | [-.460, .599] | [-.448, .456] |
|  | (.816) | (.882) | (.874) | (.045) | (.997) | (.863) | (.908) | (.797) | (.986) |
| Kansas | .088 | .120 | .148 | .231 | .157 | -.047 | .162 | .320 | .056 |
|  | .304 | .161 | .335 | .315 | .249 | .300 | .304 | .313 | .276 |
|  | [-.507, .684] | [-.196, .436] | [-.510, .806] | [-.386, .847] | [-.331, .645] | [-.636, .542] | [-.434, .758] | [-.294, .933] | [-.485, .597] |
|  | (.772) | (.458) | (.659) | (.464) | (.528) | (.875) | (.594) | (.307) | (.840) |
| Kentucky | -.220 | -.051 | -.035 | .109 | .059 | -.226 | -.011 | -.106 | -.062 |
|  | .244 | .122 | .240 | .231 | .179 | .205 | .230 | .235 | .205 |
|  | [-.699, .258] | [-.290, .188] | [-.506, .436] | [-.345, .562] | [-.291, .410] | [-.628, .176] | [-.461, .440] | [-.568, .356] | [-.464, .340] |
|  | (.366) | (.675) | (.884) | (.639) | (.740) | (.271) | (.963) | (.653) | (.762) |
| Louisiana | .472 | .039 | -.028 | -.065 | -.195 | -.317 | .029 | -.008 | .083 |
|  | .283 | .121 | .262 | .250 | .219 | .232 | .244 | .239 | .225 |
|  | [-.084, 1.028] | [-.198, .276] | [-.541, .486] | [-.556, .425] | [-.626, .235] | [-.772, .138] | [-.449, .508] | [-.476, .461] | [-.358, .524] |
|  | (.096) | (.747) | (.916) | (.795) | (.373) | (.172) | (.904) | (.975) | (.713) |
| Maine | -.327 | .148 | -.325 | .211 | -.146 | -.110 | -.302 | -.498 | -.759 |
|  | .278 | .145 | .376 | .319 | .235 | .271 | .331 | .297 | .266 |
|  | [-.872, .219] | [-.136, .431] | [-1.063, .414] | [-.415, .837] | [-.606, .314] | [-.642, .423] | [-.951, .347] | [-1.080, .084] | [-1.281, -.238] |
|  | (.241) | (.308) | (.389) | (.509) | (.534) | (.686) | (.361) | (.094) | (.004) |
| Maryland | -.083 | .140 | -.086 | .110 | -.091 | .055 | -.043 | .044 | -.040 |
|  | .238 | .109 | .265 | .227 | .179 | .218 | .229 | .231 | .222 |
|  | [-.550, .384] | [-.073, .353] | [-.606, .433] | [-.335, .556] | [-.442, .260] | [-.373, .483] | [-.493, .406] | [-.410, .497] | [-.476, .395] |
|  | (.727) | (.198) | (.744) | (.628) | (.611) | (.800) | (.850) | (.851) | (.856) |
| Massachusetts | -.012 | .066 | .102 | .396 | -.162 | .196 | .161 | .099 | .226 |
|  | .247 | .103 | .229 | .214 | .181 | .201 | .214 | .223 | .197 |
|  | [-.496, .471] | [-.135, .268] | [-.347, .551] | [-.023, .814] | [-.517, .193] | [-.197, .590] | [-.259, .580] | [-.339, .536] | [-.159, .611] |
|  | (.960) | (.519) | (.656) | (.064) | (.371) | (.328) | (.452) | (.659) | (.250) |
| Michigan | .003 | .107 | .103 | .219 | -.135 | -.134 | -.020 | -.113 | -.040 |
|  | .214 | .095 | .213 | .187 | .152 | .179 | .196 | .190 | .181 |
|  | [-.416, .423] | [-.080, .294] | [-.316, .521] | [-.147, .585] | [-.434, .164] | [-.484, .217] | [-.404, .365] | [-.486, .260] | [-.395, .314] |
|  | (.987) | (.263) | (.630) | (.240) | (.376) | (.454) | (.920) | (.553) | (.823) |
| Minnesota | .080 | .056 | .575 | .155 | -.056 | -.165 | .040 | -.016 | -.070 |
|  | .249 | .107 | .235 | .221 | .177 | .209 | .231 | .224 | .208 |
|  | [-.408, .567] | [-.154, .266] | [.115, 1.034] | [-.278, .588] | [-.404, .292] | [-.575, .246] | [-.413, .494] | [-.455, .422] | [-.479, .338] |
|  | (.748) | (.603) | (.014) | (.483) | (.753) | (.431) | (.861) | (.942) | (.736) |
| Mississippi | .411 | -.066 | .004 | .271 | -.273 | -.175 | -.124 | -.399 | -.009 |
|  | .283 | .136 | .274 | .266 | .237 | .232 | .260 | .272 | .245 |
|  | [-.145, .966] | [-.332, .200] | [-.533, .541] | [-.250, .792] | [-.738, .191] | [-.630, .280] | [-.634, .386] | [-.931, .134] | [-.491, .472] |
|  | (.147) | (.627) | (.988) | (.308) | (.249) | (.451) | (.633) | (.142) | (.970) |
| Missouri | -.133 | .053 | -.031 | -.008 | -.239 | -.161 | -.070 | -.076 | -.034 |
|  | .236 | .102 | .226 | .201 | .166 | .191 | .206 | .200 | .185 |
|  | [-.596, .331] | [-.148, .254] | [-.474, .412] | [-.402, .386] | [-.563, .086] | [-.535, .213] | [-.474, .334] | [-.468, .317] | [-.396, .328] |
|  | (.574) | (.604) | (.891) | (.969) | (.149) | (.398) | (.734) | (.706) | (.855) |
| Montana | -.210 | .040 | -.123 | .431 | .174 | -.258 | .192 | -.154 | .049 |
|  | .337 | .179 | .380 | .262 | .232 | .302 | .365 | .342 | .280 |
|  | [-.871, .451] | [-.311, .391] | [-.867, .621] | [-.083, .945] | [-.282, .629] | [-.849, .334] | [-.523, .907] | [-.824, .517] | [-.501, .599] |
|  | (.534) | (.824) | (.746) | (.100) | (.455) | (.393) | (.599) | (.653) | (.861) |
| Nebraska | .077 | -.110 | .267 | .242 | -.041 | -.245 | -.068 | .313 | .056 |
|  | .313 | .207 | .284 | .369 | .277 | .291 | .348 | .327 | .365 |
|  | [-.537, .692] | [-.516, .297] | [-.290, .824] | [-.482, .966] | [-.584, .501] | [-.816, .326] | [-.751, .615] | [-.328, .953] | [-.659, .772] |
|  | (.805) | (.596) | (.347) | (.512) | (.882) | (.400) | (.845) | (.339) | (.877) |
| Nevada | .085 | .016 | .147 | .238 | -.090 | .088 | -.049 | -.110 | .072 |
|  | .245 | .108 | .246 | .221 | .202 | .211 | .241 | .236 | .219 |
|  | [-.396, .565] | [-.196, .228] | [-.335, .630] | [-.196, .672] | [-.486, .306] | [-.325, .502] | [-.522, .424] | [-.573, .354] | [-.356, .501] |
|  | (.730) | (.880) | (.549) | (.282) | (.656) | (.675) | (.840) | (.643) | (.741) |
| New Hampshire | .295 | -.054 | .032 | .058 | -.432 | -.140 | -.335 | -.190 | .054 |
|  | .303 | .159 | .276 | .277 | .244 | .237 | .268 | .303 | .260 |
|  | [-.298, .889] | [-.365, .257] | [-.509, .572] | [-.485, .602] | [-.911, .047] | [-.604, .324] | [-.861, .191] | [-.784, .405] | [-.455, .563] |
|  | (.329) | (.733) | (.909) | (.833) | (.077) | (.553) | (.212) | (.532) | (.835) |
| New Jersey | .305 | .084 | -.186 | .293 | -.276 | .049 | .132 | .029 | .245 |
|  | .235 | .101 | .232 | .210 | .184 | .201 | .216 | .201 | .208 |
|  | [-.156, .767] | [-.115, .282] | [-.641, .269] | [-.118, .704] | [-.637, .085] | [-.345, .442] | [-.292, .556] | [-.365, .424] | [-.162, .652] |
|  | (.195) | (.409) | (.424) | (.163) | (.133) | (.808) | (.543) | (.883) | (.237) |
| New Mexico | .263 | .070 | -.188 | .121 | -.009 | -.184 | -.074 | .169 | -.022 |
|  | .276 | .118 | .291 | .253 | .200 | .230 | .237 | .267 | .229 |
|  | [-.278, .803] | [-.162, .301] | [-.759, .382] | [-.374, .616] | [-.402, .384] | [-.636, .267] | [-.538, .391] | [-.355, .694] | [-.471, .427] |
|  | (.341) | (.555) | (.518) | (.631) | (.964) | (.423) | (.756) | (.527) | (.924) |
| New York | .209 | .066 | -.166 | .352 | -.148 | .059 | .004 | -.005 | .199 |
|  | .212 | .091 | .210 | .180 | .148 | .173 | .187 | .181 | .169 |
|  | [-.207, .625] | [-.113, .245] | [-.577, .245] | [-<.001, .705] | [-.439, .143] | [-.280, .397] | [-.362, .370] | [-.360, .349] | [-.132, .530] |
|  | (.324) | (.471) | (.428) | (.050) | (.320) | (.735) | (.982) | (.976) | (.238) |
| North Carolina | -.136 | .098 | .034 | .232 | -.079 | -.097 | .031 | .145 | .112 |
|  | .234 | .097 | .213 | .196 | .162 | .189 | .202 | .201 | .191 |
|  | [-.594, .323] | [-.092, .288] | [-.384, .453] | [-.153, .617] | [-.397, .239] | [-.468, .273] | [-.365, .428] | [-.248, .539] | [-.262, .487] |
|  | (.562) | (.310) | (.873) | (.238) | (.625) | (.607) | (.877) | (.469) | (.556) |
| North Dakota | .117 | .152 | .227 | .079 | -.157 | -.397 | .309 | -.339 | -.180 |
|  | .389 | .161 | .438 | .342 | .283 | .306 | .339 | .466 | .408 |
|  | [-.646, .880] | [-.163, .467] | [-.632, 1.086] | [-.591, .750] | [-.712, .397] | [-.998, .204] | [-.356, .975] | [-1.253, .576] | [-.980, .619] |
|  | (.764) | (.344) | (.604) | (.816) | (.579) | (.195) | (.362) | (.468) | (.659) |
| Ohio | .028 | .087 | .062 | .163 | -.094 | -.066 | -.025 | -.246 | -.077 |
|  | .215 | .094 | .211 | .189 | .154 | .180 | .196 | .188 | .179 |
|  | [-.394, .450] | [-.097, .272] | [-.352, .476] | [-.208, .534] | [-.395, .208] | [-.419, .287] | [-.410, .360] | [-.615, .122] | [-.427, .273] |
|  | (.898) | (.353) | (.769) | (.389) | (.543) | (.714) | (.897) | (.190) | (.666) |
| Oklahoma | -.075 | .024 | .340 | .031 | -.137 | -.043 | -.039 | -.115 | -.131 |
|  | .304 | .150 | .288 | .262 | .236 | .237 | .268 | .252 | .267 |
|  | [-.672, .521] | [-.269, .318] | [-.224, .904] | [-.483, .545] | [-.600, .325] | [-.508, .422] | [-.564, .486] | [-.609, .378] | [-.654, .391] |
|  | (.804) | (.872) | (.238) | (.906) | (.560) | (.857) | (.884) | (.646) | (.622) |
| Oregon | -.166 | .112 | .446 | .149 | .100 | -.130 | .139 | -.055 | .141 |
|  | .224 | .101 | .214 | .207 | .164 | .192 | .213 | .207 | .204 |
|  | [-.605, .273] | [-.086, .310] | [.027, .866] | [-.256, .554] | [-.221, .421] | [-.507, .248] | [-.279, .557] | [-.461, .351] | [-.259, .540] |
|  | (.459) | (.268) | (.037) | (.471) | (.541) | (.500) | (.514) | (.790) | (.490) |
| Pennsylvania | .012 | .091 | .129 | .133 | -.236 | -.190 | -.040 | -.032 | .012 |
|  | .204 | .091 | .198 | .179 | .150 | .169 | .188 | .179 | .171 |
|  | [-.388, .413] | [-.088, .270] | [-.259, .517] | [-.218, .483] | [-.530, .058] | [-.521, .141] | [-.410, .329] | [-.383, .319] | [-.323, .348] |
|  | (.952) | (.320) | (.515) | (.458) | (.115) | (.261) | (.830) | (.858) | (.942) |
| Rhode Island | .043 | .154 | -.176 | .464 | -.109 | .035 | .137 | -.012 | -.018 |
|  | .404 | .153 | .347 | .377 | .309 | .390 | .358 | .378 | .364 |
|  | [-.748, .835] | [-.147, .454] | [-.856, .505] | [-.276, 1.204] | [-.716, .497] | [-.728, .799] | [-.565, .839] | [-.754, .730] | [-.733, .696] |
|  | (.914) | (.316) | (.613) | (.219) | (.723) | (.927) | (.701) | (.975) | (.960) |
| South Carolina | .051 | .046 | -.014 | .072 | .010 | -.126 | .150 | .025 | .010 |
|  | .252 | .111 | .242 | .213 | .173 | .209 | .225 | .224 | .208 |
|  | [-.443, .545] | [-.172, .264] | [-.488, .460] | [-.345, .489] | [-.329, .350] | [-.535, .284] | [-.291, .592] | [-.415, .465] | [-.397, .417] |
|  | (.840) | (.678) | (.954) | (.734) | (.952) | (.547) | (.505) | (.912) | (.962) |
| South Dakota | .219 | .058 | .255 | -.703 | .020 | -.740 | -.094 | -.043 | -.383 |
|  | .319 | .178 | .336 | .273 | .270 | .317 | .349 | .387 | .310 |
|  | [-.405, .844] | [-.291, .406] | [-.403, .913] | [-1.237, -.168] | [-.510, .550] | [-1.361, -.119] | [-.778, .589] | [-.803, .716] | [-.990, .225] |
|  | (.491) | (.746) | (.447) | (.010) | (.941) | (.020) | (.787) | (.911) | (.217) |
| Tennessee | .061 | .115 | .157 | .354 | -.109 | .153 | .167 | -.176 | .082 |
|  | .252 | .108 | .217 | .216 | .173 | .199 | .216 | .216 | .210 |
|  | [-.433, .555] | [-.097, .327] | [-.269, .583] | [-.070, .777] | [-.449, .231] | [-.238, .543] | [-.256, .590] | [-.599, .247] | [-.330, .495] |
|  | (.808) | (.287) | (.469) | (.102) | (.528) | (.443) | (.440) | (.415) | (.695) |
| Texas | -.200 | .084 | .063 | .077 | -.176 | -.261 | -.024 | .014 | .091 |
|  | .210 | .092 | .205 | .182 | .149 | .173 | .190 | .180 | .172 |
|  | [-.611, .211] | [-.097, .266] | [-.338, .464] | [-.279, .434] | [-.468, .116] | [-.600, .079] | [-.397, .348] | [-.339, .366] | [-.246, .429] |
|  | (.340) | (.361) | (.758) | (.670) | (.237) | (.133) | (.899) | (.938) | (.596) |
| Utah | -.090 | .065 | .423 | .316 | -.138 | -.094 | -.193 | .165 | .106 |
|  | .283 | .139 | .244 | .256 | .204 | .242 | .261 | .286 | .246 |
|  | [-.645, .465] | [-.206, .337] | [-.056, .902] | [-.187, .819] | [-.539, .263] | [-.569, .380] | [-.704, .319] | [-.396, .725] | [-.375, .588] |
|  | (.751) | (.637) | (.083) | (.218) | (.499) | (.696) | (.460) | (.565) | (.665) |
| Vermont | .606 | .093 | -.007 | -.047 | -.228 | -.416 | -.134 | .156 | -.627 |
|  | .334 | .177 | .376 | .302 | .281 | .325 | .291 | .320 | .260 |
|  | [-.049, 1.262] | [-.253, .439] | [-.744, .730] | [-.638, .545] | [-.779, .324] | [-1.052, .221] | [-.705, .436] | [-.471, .783] | [-1.137, -.117] |
|  | (.070) | (.599) | (.985) | (.877) | (.418) | (.200) | (.644) | (.625) | (.016) |
| Virginia | .120 | .124 | .161 | .107 | -.162 | -.163 | -.134 | -.243 | .045 |
|  | .215 | .094 | .211 | .192 | .157 | .180 | .201 | .195 | .186 |
|  | [-.301, .542] | [-.060, .309] | [-.252, .574] | [-.270, .483] | [-.471, .146] | [-.516, .190] | [-.529, .261] | [-.625, .140] | [-.320, .409] |
|  | (.576) | (.186) | (.445) | (.578) | (.303) | (.365) | (.506) | (.214) | (.811) |
| Washington | .375 | .095 | .322 | .293 | -.226 | .022 | .057 | .007 | -.019 |
|  | .227 | .107 | .212 | .202 | .168 | .191 | .205 | .199 | .190 |
|  | [-.071, .821] | [-.114, .305] | [-.093, .737] | [-.103, .689] | [-.554, .103] | [-.352, .395] | [-.345, .459] | [-.383, .397] | [-.391, .353] |
|  | (.099) | (.372) | (.128) | (.147) | (.178) | (.910) | (.782) | (.972) | (.922) |
| West Virginia | -.202 | .148 | .240 | .238 | -.189 | .053 | .048 | -.031 | .163 |
|  | .299 | .134 | .272 | .260 | .219 | .259 | .234 | .280 | .235 |
|  | [-.788, .383] | [-.115, .412] | [-.293, .774] | [-.272, .748] | [-.619, .241] | [-.456, .561] | [-.411, .507] | [-.580, .518] | [-.298, .623] |
|  | (.498) | (.268) | (.377) | (.361) | (.389) | (.839) | (.838) | (.911) | (.489) |
| Wisconsin | .060 | .080 | -.009 | .078 | -.323 | -.378 | -.181 | -.267 | -.170 |
|  | .243 | .105 | .221 | .207 | .184 | .199 | .216 | .203 | .194 |
|  | [-.416, .537] | [-.126, .287] | [-.443, .424] | [-.329, .484] | [-.684, .037] | [-.767, .012] | [-.605, .243] | [-.665, .131] | [-.551, .211] |
|  | (.805) | (.446) | (.967) | (.707) | (.078) | (.058) | (.403) | (.189) | (.381) |
| Wyoming | .607 | .136 | -.648 | -.375 | .132 | -.275 | .037 | -.019 | -.019 |
|  | .323 | .260 | .827 | .857 | .312 | .802 | .589 | .786 | .789 |
|  | [-.026, 1.241] | [-.373, .646] | [-2.270, .974] | [-2.055, 1.305] | [-.479, .743] | [-1.847, 1.298] | [-1.117, 1.192] | [-1.560, 1.521] | [-1.566, 1.528] |
|  | (.060) | (.600) | (.434) | (.662) | (.672) | (.732) | (.949) | (.980) | (.981) |
| N | 2982 | 2980 | 2968 | 2980 | 2980 | 2981 | 2981 | 2981 | 2979 |
| Pseudo-R^2^ |  |  |  |  |  |  |  |  |  |
| Model χ^2^ |  |  |  |  |  |  |  |  |  |
| d.f. | 90 | 90 | 90 | 90 | 90 | 90 | 90 | 90 | 90 |
| p-value | <.001 | <.001 | <.001 | <.001 | <.001 | <.001 | <.001 | <.001 | <.001 |

**S10 Fig. Health Attitudes, by News Source.** Estimates are the predicted effects comparing Republicans and Others (unaffiliated or identifying with a third party) to self-identified Democrats for respondents who report watching right-wing news versus those who do not. Confidence intervals are not adjusted for multiple comparisons in this exploratory analysis.

S29 Table. Regression Results, Health Policies, Interacting with News Source.

|  | (1) | (2) | (3) | (4) | (5) | (6) | (7) | (8) | (9) |
| --- | --- | --- | --- | --- | --- | --- | --- | --- | --- |
|  | freetesting | waivecosts | closeschools | canceleverything | paidleave | compensatefirms | nationalguard | stopevents | stateslead |
| Republican | -.557 | -.675 | -.445 | -.355 | -.595 | .148 | -.134 | -.532 | .063 |
|  | .061 | .064 | .068 | .064 | .064 | .069 | .076 | .080 | .062 |
|  | [-.678, -.437] | [-.800, -.549] | [-.578, -.312] | [-.482, -.229] | [-.721, -.469] | [.013, .283] | [-.284, .015] | [-.688, -.375] | [-.060, .185] |
|  | (<.001) | (<.001) | (<.001) | (<.001) | (<.001) | (.032) | (.077) | (<.001) | (.315) |
| Other | -.239 | -.327 | -.278 | -.317 | -.297 | -.198 | -.363 | -.309 | -.143 |
|  | .040 | .045 | .048 | .049 | .045 | .058 | .058 | .057 | .050 |
|  | [-.318, -.160] | [-.415, -.238] | [-.373, -.183] | [-.413, -.221] | [-.385, -.209] | [-.311, -.085] | [-.476, -.250] | [-.421, -.196] | [-.241, -.045] |
|  | (<.001) | (<.001) | (<.001) | (<.001) | (<.001) | (.001) | (<.001) | (<.001) | (.004) |
| rightnews=1 | -.336 | -.351 | -.294 | -.110 | -.286 | .421 | .191 | -.560 | .295 |
|  | .173 | .168 | .184 | .160 | .153 | .180 | .213 | .220 | .150 |
|  | [-.675, .003] | [-.681, -.022] | [-.655, .067] | [-.424, .204] | [-.586, .014] | [.068, .773] | [-.227, .610] | [-.992, -.129] | [.000, .590] |
|  | (.052) | (.037) | (.110) | (.493) | (.062) | (.019) | (.370) | (.011) | (.050) |
| Republican # rightnews=1 | .295 | .183 | .223 | -.001 | .244 | -.332 | -.351 | .568 | -.356 |
|  | .190 | .189 | .202 | .180 | .174 | .195 | .234 | .240 | .169 |
|  | [-.076, .667] | [-.186, .553] | [-.173, .620] | [-.353, .351] | [-.097, .585] | [-.715, .051] | [-.809, .107] | [.097, 1.039] | [-.687, -.026] |
|  | (.119) | (.331) | (.269) | (.996) | (.160) | (.090) | (.133) | (.018) | (.035) |
| Other # rightnews=1 | -.059 | .017 | -.079 | -.244 | -.305 | -.263 | -.300 | .084 | -.228 |
|  | .191 | .190 | .207 | .189 | .179 | .201 | .239 | .246 | .175 |
|  | [-.434, .316] | [-.356, .389] | [-.486, .327] | [-.614, .126] | [-.656, .046] | [-.657, .132] | [-.769, .169] | [-.398, .566] | [-.572, .115] |
|  | (.758) | (.930) | (.702) | (.195) | (.088) | (.192) | (.210) | (.732) | (.192) |
| 35- | .157 | .177 | .127 | .158 | .162 | .084 | .025 | .047 | .185 |
|  | .049 | .055 | .061 | .059 | .057 | .068 | .069 | .070 | .059 |
|  | [.061, .253] | [.068, .286] | [.008, .247] | [.042, .274] | [.050, .273] | [-.049, .216] | [-.110, .160] | [-.090, .184] | [.069, .301] |
|  | (.001) | (.001) | (.037) | (.008) | (.005) | (.217) | (.714) | (.504) | (.002) |
| 50- | .181 | .148 | .133 | .171 | .116 | .016 | -.063 | .209 | .176 |
|  | .049 | .053 | .057 | .058 | .054 | .064 | .065 | .066 | .056 |
|  | [.085, .278] | [.044, .251] | [.021, .244] | [.058, .284] | [.009, .222] | [-.109, .141] | [-.191, .064] | [.081, .338] | [.066, .286] |
|  | (<.001) | (.005) | (.019) | (.003) | (.033) | (.798) | (.330) | (.001) | (.002) |
| 70- | .241 | .051 | .164 | .371 | .050 | -.060 | .244 | .278 | .309 |
|  | .060 | .068 | .071 | .068 | .066 | .078 | .082 | .083 | .069 |
|  | [.124, .357] | [-.083, .184] | [.025, .302] | [.238, .504] | [-.080, .180] | [-.212, .092] | [.083, .405] | [.116, .440] | [.175, .444] |
|  | (<.001) | (.456) | (.020) | (<.001) | (.455) | (.439) | (.003) | (.001) | (<.001) |
| gender | .091 | .038 | .166 | .218 | .118 | .328 | .185 | .193 | .099 |
|  | .034 | .038 | .041 | .040 | .038 | .045 | .049 | .048 | .041 |
|  | [.024, .157] | [-.037, .113] | [.087, .246] | [.138, .297] | [.043, .192] | [.239, .417] | [.090, .280] | [.098, .287] | [.018, .179] |
|  | (.008) | (.324) | (<.001) | (<.001) | (.002) | (<.001) | (<.001) | (<.001) | (.016) |
| white | .121 | .158 | .019 | .017 | .146 | -.056 | -.178 | .257 | -.028 |
|  | .042 | .046 | .049 | .048 | .046 | .055 | .057 | .059 | .050 |
|  | [.039, .203] | [.068, .249] | [-.077, .115] | [-.078, .112] | [.057, .235] | [-.165, .052] | [-.291, -.066] | [.141, .373] | [-.126, .070] |
|  | (.004) | (.001) | (.700) | (.725) | (.001) | (.309) | (.002) | (<.001) | (.574) |
| married | -.027 | -.008 | .002 | .052 | .003 | -.015 | .132 | -.037 | -.006 |
|  | .038 | .042 | .043 | .044 | .041 | .050 | .052 | .053 | .044 |
|  | [-.100, .047] | [-.089, .074] | [-.083, .086] | [-.033, .138] | [-.077, .084] | [-.113, .084] | [.031, .234] | [-.141, .066] | [-.092, .081] |
|  | (.480) | (.855) | (.967) | (.233) | (.936) | (.770) | (.011) | (.480) | (.894) |
| High school graduate | -.184 | -.175 | -.125 | -.082 | -.222 | -.258 | -.138 | .109 | -.236 |
|  | .088 | .101 | .119 | .129 | .116 | .124 | .141 | .158 | .108 |
|  | [-.355, -.012] | [-.373, .024] | [-.358, .109] | [-.335, .171] | [-.448, .005] | [-.501, -.014] | [-.414, .137] | [-.201, .419] | [-.448, -.025] |
|  | (.036) | (.084) | (.295) | (.523) | (.056) | (.038) | (.325) | (.490) | (.029) |
| Some college | -.109 | -.097 | -.167 | -.086 | -.135 | -.412 | -.397 | .314 | -.243 |
|  | .090 | .104 | .122 | .132 | .119 | .129 | .144 | .159 | .112 |
|  | [-.286, .068] | [-.302, .107] | [-.406, .073] | [-.345, .173] | [-.369, .099] | [-.664, -.160] | [-.679, -.115] | [.002, .626] | [-.462, -.024] |
|  | (.226) | (.351) | (.173) | (.516) | (.258) | (.001) | (.006) | (.049) | (.030) |
| 2-year | -.086 | -.110 | -.178 | -.080 | -.157 | -.247 | -.333 | .286 | -.249 |
|  | .093 | .109 | .128 | .137 | .123 | .135 | .151 | .166 | .119 |
|  | [-.269, .097] | [-.324, .104] | [-.430, .073] | [-.349, .189] | [-.398, .084] | [-.512, .017] | [-.629, -.036] | [-.039, .611] | [-.482, -.016] |
|  | (.359) | (.313) | (.164) | (.561) | (.203) | (.066) | (.028) | (.085) | (.036) |
| 4-year | -.248 | -.260 | -.246 | -.142 | -.254 | -.404 | -.401 | .283 | -.278 |
|  | .093 | .106 | .123 | .134 | .120 | .130 | .147 | .162 | .114 |
|  | [-.430, -.066] | [-.468, -.051] | [-.487, -.004] | [-.406, .121] | [-.489, -.019] | [-.660, -.148] | [-.690, -.112] | [-.035, .601] | [-.502, -.054] |
|  | (.007) | (.015) | (.046) | (.288) | (.034) | (.002) | (.007) | (.081) | (.015) |
| Post-grad | -.232 | -.258 | -.332 | -.116 | -.276 | -.504 | -.364 | .359 | -.419 |
|  | .098 | .114 | .131 | .139 | .126 | .139 | .155 | .170 | .123 |
|  | [-.424, -.041] | [-.481, -.036] | [-.590, -.075] | [-.388, .156] | [-.523, -.029] | [-.776, -.231] | [-.668, -.060] | [.026, .692] | [-.660, -.178] |
|  | (.018) | (.023) | (.011) | (.404) | (.029) | (<.001) | (.019) | (.035) | (.001) |
| income=2 | .119 | .063 | .069 | .051 | -.004 | -.090 | .069 | .002 | .062 |
|  | .106 | .115 | .115 | .122 | .113 | .131 | .131 | .141 | .116 |
|  | [-.089, .327] | [-.161, .288] | [-.156, .294] | [-.188, .290] | [-.226, .218] | [-.347, .168] | [-.188, .325] | [-.273, .278] | [-.165, .289] |
|  | (.263) | (.580) | (.548) | (.677) | (.972) | (.495) | (.600) | (.987) | (.592) |
| income=3 | .193 | .121 | .122 | .056 | .141 | -.092 | -.030 | -.083 | .221 |
|  | .101 | .110 | .111 | .116 | .105 | .126 | .130 | .136 | .112 |
|  | [-.006, .391] | [-.095, .337] | [-.096, .340] | [-.173, .284] | [-.064, .346] | [-.340, .156] | [-.284, .225] | [-.351, .184] | [.001, .442] |
|  | (.058) | (.273) | (.272) | (.634) | (.176) | (.468) | (.819) | (.541) | (.049) |
| income=4 | .212 | .193 | .117 | .142 | .089 | -.165 | .094 | .190 | .142 |
|  | .104 | .112 | .116 | .119 | .108 | .130 | .128 | .140 | .117 |
|  | [.008, .415] | [-.026, .412] | [-.110, .344] | [-.091, .375] | [-.123, .302] | [-.421, .091] | [-.158, .345] | [-.085, .465] | [-.087, .371] |
|  | (.041) | (.084) | (.313) | (.232) | (.409) | (.206) | (.464) | (.175) | (.226) |
| income=5 | .184 | .181 | -.031 | .034 | .090 | -.319 | -.150 | .026 | .091 |
|  | .107 | .112 | .121 | .123 | .111 | .133 | .133 | .141 | .118 |
|  | [-.027, .394] | [-.038, .400] | [-.268, .206] | [-.207, .274] | [-.127, .308] | [-.580, -.058] | [-.411, .111] | [-.250, .302] | [-.141, .323] |
|  | (.087) | (.105) | (.798) | (.783) | (.415) | (.017) | (.261) | (.853) | (.441) |
| income=6 | .175 | .113 | .146 | .140 | .124 | -.150 | .099 | .124 | .147 |
|  | .107 | .116 | .119 | .124 | .115 | .137 | .134 | .144 | .119 |
|  | [-.034, .384] | [-.115, .340] | [-.088, .379] | [-.103, .382] | [-.103, .350] | [-.419, .118] | [-.165, .362] | [-.159, .406] | [-.086, .380] |
|  | (.101) | (.331) | (.222) | (.259) | (.284) | (.272) | (.462) | (.391) | (.217) |
| income=7 | .236 | .024 | .237 | .214 | .117 | .016 | .035 | .270 | .025 |
|  | .109 | .122 | .119 | .124 | .115 | .138 | .138 | .146 | .126 |
|  | [.022, .450] | [-.216, .263] | [.004, .470] | [-.028, .457] | [-.109, .344] | [-.256, .287] | [-.235, .305] | [-.017, .557] | [-.222, .272] |
|  | (.030) | (.847) | (.047) | (.083) | (.308) | (.910) | (.800) | (.065) | (.843) |
| income=8 | .209 | .113 | .104 | .072 | .229 | -.069 | -.126 | .070 | .088 |
|  | .106 | .119 | .121 | .127 | .112 | .138 | .144 | .146 | .123 |
|  | [.002, .416] | [-.119, .346] | [-.133, .341] | [-.176, .320] | [.010, .448] | [-.340, .203] | [-.408, .157] | [-.216, .355] | [-.153, .328] |
|  | (.047) | (.340) | (.389) | (.570) | (.040) | (.619) | (.384) | (.631) | (.475) |
| income=9 | .017 | -.126 | .080 | -.008 | .037 | -.170 | -.234 | .206 | .053 |
|  | .113 | .124 | .122 | .126 | .116 | .135 | .136 | .141 | .127 |
|  | [-.204, .238] | [-.368, .117] | [-.160, .320] | [-.255, .239] | [-.190, .265] | [-.434, .095] | [-.500, .032] | [-.071, .483] | [-.196, .301] |
|  | (.879) | (.309) | (.513) | (.947) | (.747) | (.208) | (.084) | (.146) | (.678) |
| income=10 | .185 | .048 | .024 | .156 | .119 | -.077 | -.078 | .198 | .081 |
|  | .112 | .120 | .126 | .129 | .118 | .143 | .143 | .153 | .129 |
|  | [-.035, .404] | [-.188, .284] | [-.223, .271] | [-.096, .408] | [-.113, .351] | [-.357, .203] | [-.359, .203] | [-.101, .497] | [-.172, .334] |
|  | (.099) | (.690) | (.848) | (.225) | (.313) | (.589) | (.587) | (.195) | (.532) |
| income=11 | .214 | -.044 | .189 | .229 | .010 | -.093 | -.120 | .272 | .112 |
|  | .113 | .132 | .131 | .131 | .124 | .151 | .150 | .151 | .133 |
|  | [-.009, .436] | [-.302, .214] | [-.067, .446] | [-.027, .486] | [-.233, .254] | [-.389, .203] | [-.414, .174] | [-.024, .567] | [-.148, .373] |
|  | (.060) | (.740) | (.147) | (.080) | (.934) | (.536) | (.424) | (.071) | (.397) |
| income=12 | .063 | -.125 | .066 | .102 | -.011 | -.109 | -.270 | .341 | .090 |
|  | .127 | .140 | .145 | .149 | .134 | .167 | .170 | .170 | .145 |
|  | [-.185, .312] | [-.400, .150] | [-.218, .350] | [-.191, .394] | [-.274, .252] | [-.436, .219] | [-.604, .063] | [.008, .674] | [-.196, .375] |
|  | (.618) | (.372) | (.649) | (.495) | (.932) | (.515) | (.112) | (.045) | (.538) |
| income=13 | .390 | .190 | .124 | .341 | .162 | -.386 | -.074 | .365 | .050 |
|  | .136 | .166 | .193 | .194 | .164 | .232 | .245 | .218 | .193 |
|  | [.123, .657] | [-.136, .515] | [-.254, .502] | [-.039, .721] | [-.159, .483] | [-.841, .068] | [-.555, .406] | [-.063, .793] | [-.328, .429] |
|  | (.004) | (.253) | (.519) | (.078) | (.322) | (.096) | (.762) | (.094) | (.795) |
| income=14 | -.111 | -.170 | -.253 | -.002 | -.083 | -.249 | -.508 | .300 | -.266 |
|  | .213 | .212 | .227 | .210 | .210 | .224 | .269 | .226 | .225 |
|  | [-.528, .307] | [-.586, .245] | [-.697, .192] | [-.413, .410] | [-.494, .329] | [-.688, .189] | [-1.034, .019] | [-.142, .742] | [-.707, .175] |
|  | (.604) | (.421) | (.265) | (.993) | (.693) | (.265) | (.059) | (.184) | (.236) |
| income=15 | .685 | .732 | .003 | .277 | .480 | .192 | 1.392 | .836 | 1.245 |
|  | .222 | .294 | .583 | .682 | .112 | .405 | .333 | .151 | .156 |
|  | [.250, 1.121] | [.156, 1.309] | [-1.141, 1.147] | [-1.060, 1.614] | [.261, .699] | [-.601, .985] | [.740, 2.044] | [.541, 1.132] | [.940, 1.550] |
|  | (.002) | (.013) | (.996) | (.685) | (<.001) | (.635) | (<.001) | (<.001) | (<.001) |
| income=16 | .404 | .470 | .414 | .481 | .553 | -.031 | .116 | -.044 | .178 |
|  | .211 | .222 | .274 | .256 | .179 | .339 | .383 | .398 | .349 |
|  | [-.010, .818] | [.034, .905] | [-.123, .951] | [-.021, .983] | [.202, .905] | [-.695, .633] | [-.635, .867] | [-.825, .738] | [-.506, .862] |
|  | (.056) | (.035) | (.131) | (.061) | (.002) | (.927) | (.762) | (.913) | (.610) |
| income=17 | .079 | .015 | .002 | .014 | -.028 | -.189 | -.325 | .195 | .108 |
|  | .101 | .109 | .110 | .114 | .106 | .124 | .124 | .128 | .109 |
|  | [-.120, .277] | [-.200, .229] | [-.213, .217] | [-.209, .237] | [-.237, .180] | [-.433, .054] | [-.569, -.082] | [-.057, .447] | [-.107, .323] |
|  | (.437) | (.894) | (.984) | (.901) | (.792) | (.128) | (.009) | (.129) | (.324) |
| Rural Code=2 | -.049 | -.052 | -.110 | -.080 | .006 | -.113 | -.120 | .023 | -.038 |
|  | .045 | .050 | .054 | .054 | .050 | .060 | .063 | .062 | .054 |
|  | [-.138, .040] | [-.150, .046] | [-.217, -.003] | [-.186, .026] | [-.091, .104] | [-.232, .005] | [-.244, .004] | [-.100, .145] | [-.144, .069] |
|  | (.280) | (.297) | (.044) | (.138) | (.900) | (.060) | (.058) | (.713) | (.488) |
| Rural Code=3 | -.098 | -.201 | -.162 | -.042 | -.030 | -.026 | -.042 | .039 | -.062 |
|  | .064 | .071 | .074 | .072 | .070 | .082 | .084 | .084 | .071 |
|  | [-.224, .028] | [-.340, -.061] | [-.308, -.016] | [-.183, .098] | [-.167, .108] | [-.188, .135] | [-.207, .123] | [-.126, .204] | [-.201, .077] |
|  | (.126) | (.005) | (.030) | (.556) | (.672) | (.749) | (.619) | (.642) | (.381) |
| Rural Code=4 | -.042 | .048 | -.239 | -.062 | .093 | -.063 | -.151 | -.128 | .046 |
|  | .094 | .089 | .112 | .109 | .093 | .117 | .118 | .124 | .098 |
|  | [-.227, .143] | [-.127, .222] | [-.459, -.019] | [-.275, .152] | [-.090, .275] | [-.292, .166] | [-.382, .079] | [-.372, .115] | [-.146, .237] |
|  | (.655) | (.593) | (.033) | (.571) | (.321) | (.592) | (.199) | (.301) | (.640) |
| Rural Code=5 | -.027 | -.125 | .061 | .078 | .107 | -.083 | .011 | .305 | -.120 |
|  | .150 | .159 | .151 | .146 | .143 | .171 | .186 | .169 | .152 |
|  | [-.321, .267] | [-.437, .186] | [-.236, .357] | [-.208, .364] | [-.173, .387] | [-.419, .252] | [-.353, .375] | [-.027, .637] | [-.419, .179] |
|  | (.856) | (.430) | (.688) | (.592) | (.452) | (.626) | (.953) | (.071) | (.431) |
| Rural Code=6 | .001 | -.031 | -.100 | -.132 | .047 | -.004 | .022 | -.071 | -.230 |
|  | .084 | .093 | .099 | .106 | .089 | .102 | .126 | .119 | .111 |
|  | [-.165, .166] | [-.213, .151] | [-.293, .094] | [-.339, .075] | [-.126, .221] | [-.204, .197] | [-.226, .270] | [-.304, .162] | [-.448, -.012] |
|  | (.992) | (.740) | (.311) | (.212) | (.593) | (.971) | (.861) | (.549) | (.039) |
| Rural Code=7 | -.191 | -.095 | -.321 | -.170 | -.191 | -.167 | -.110 | -.197 | -.225 |
|  | .125 | .131 | .148 | .142 | .140 | .146 | .158 | .176 | .153 |
|  | [-.436, .055] | [-.352, .163] | [-.611, -.031] | [-.449, .109] | [-.466, .084] | [-.454, .120] | [-.419, .199] | [-.541, .148] | [-.524, .074] |
|  | (.128) | (.470) | (.030) | (.232) | (.173) | (.253) | (.486) | (.264) | (.140) |
| Rural Code=8 | -.031 | -.087 | -.213 | -.377 | .034 | .091 | -.703 | -<.001 | .434 |
|  | .282 | .272 | .325 | .286 | .292 | .274 | .291 | .313 | .200 |
|  | [-.585, .522] | [-.620, .445] | [-.851, .425] | [-.938, .184] | [-.537, .606] | [-.447, .628] | [-1.274, -.132] | [-.613, .613] | [.042, .826] |
|  | (.912) | (.747) | (.512) | (.187) | (.906) | (.741) | (.016) | (.999) | (.030) |
| Rural Code=9 | -.044 | .037 | .047 | -.081 | .012 | -.425 | .042 | .022 | -.019 |
|  | .192 | .183 | .209 | .241 | .221 | .251 | .246 | .249 | .160 |
|  | [-.421, .332] | [-.322, .396] | [-.362, .456] | [-.553, .392] | [-.421, .445] | [-.917, .067] | [-.441, .525] | [-.465, .510] | [-.332, .295] |
|  | (.818) | (.841) | (.822) | (.738) | (.957) | (.090) | (.865) | (.929) | (.908) |
| Alaska | -.006 | -.046 | .289 | .248 | .009 | .398 | .138 | .344 | -.018 |
|  | .222 | .450 | .366 | .303 | .305 | .351 | .524 | .406 | .423 |
|  | [-.440, .429] | [-.928, .836] | [-.429, 1.007] | [-.346, .842] | [-.590, .608] | [-.291, 1.087] | [-.889, 1.166] | [-.452, 1.139] | [-.846, .811] |
|  | (.979) | (.918) | (.431) | (.414) | (.977) | (.257) | (.792) | (.397) | (.967) |
| Arizona | -.198 | -.068 | .002 | -.110 | -.039 | -.288 | -.323 | .115 | -.285 |
|  | .157 | .189 | .222 | .200 | .187 | .221 | .222 | .297 | .228 |
|  | [-.506, .110] | [-.439, .303] | [-.433, .437] | [-.502, .283] | [-.406, .329] | [-.721, .145] | [-.759, .113] | [-.466, .697] | [-.732, .161] |
|  | (.208) | (.719) | (.993) | (.584) | (.837) | (.193) | (.146) | (.697) | (.211) |
| Arkansas | -.176 | -.162 | -.078 | -.113 | -.133 | -.257 | -.070 | .134 | -.126 |
|  | .158 | .203 | .247 | .208 | .219 | .267 | .261 | .310 | .252 |
|  | [-.485, .133] | [-.560, .235] | [-.563, .408] | [-.521, .295] | [-.563, .296] | [-.780, .267] | [-.583, .443] | [-.473, .741] | [-.620, .368] |
|  | (.263) | (.424) | (.754) | (.586) | (.542) | (.337) | (.789) | (.665) | (.617) |
| California | -.077 | .083 | .072 | -.042 | .030 | -.243 | .118 | .273 | -.192 |
|  | .120 | .157 | .186 | .170 | .158 | .201 | .191 | .265 | .201 |
|  | [-.312, .158] | [-.225, .391] | [-.293, .437] | [-.376, .292] | [-.279, .339] | [-.638, .152] | [-.257, .493] | [-.248, .793] | [-.585, .202] |
|  | (.521) | (.597) | (.699) | (.804) | (.849) | (.227) | (.538) | (.305) | (.339) |
| Colorado | -.109 | -.346 | -.105 | -.069 | -.144 | -.229 | .050 | .310 | -.143 |
|  | .184 | .230 | .253 | .243 | .224 | .259 | .271 | .320 | .248 |
|  | [-.470, .251] | [-.797, .105] | [-.601, .391] | [-.546, .408] | [-.583, .295] | [-.736, .278] | [-.480, .581] | [-.317, .936] | [-.630, .344] |
|  | (.552) | (.133) | (.677) | (.777) | (.520) | (.376) | (.853) | (.333) | (.565) |
| Connecticut | .098 | .284 | .504 | .432 | .085 | .169 | .245 | .182 | .216 |
|  | .196 | .183 | .208 | .207 | .208 | .279 | .283 | .358 | .251 |
|  | [-.286, .482] | [-.076, .643] | [.096, .913] | [.027, .837] | [-.323, .494] | [-.377, .716] | [-.309, .800] | [-.519, .883] | [-.276, .708] |
|  | (.618) | (.122) | (.016) | (.037) | (.683) | (.544) | (.386) | (.611) | (.389) |
| Delaware | -.340 | .166 | .093 | .116 | -.363 | -.604 | .311 | -.056 | -.264 |
|  | .231 | .242 | .295 | .243 | .312 | .444 | .363 | .439 | .334 |
|  | [-.793, .113] | [-.308, .640] | [-.485, .672] | [-.361, .593] | [-.975, .249] | [-1.474, .267] | [-.399, 1.022] | [-.915, .804] | [-.918, .391] |
|  | (.141) | (.491) | (.752) | (.634) | (.245) | (.174) | (.390) | (.899) | (.430) |
| District of Columbia | -.522 | .009 | .201 | -.134 | -.461 | -.007 | .492 | .728 | .073 |
|  | .415 | .266 | .474 | .359 | .551 | .290 | .494 | .405 | .408 |
|  | [-1.335, .291] | [-.514, .531] | [-.728, 1.129] | [-.837, .569] | [-1.543, .620] | [-.576, .562] | [-.475, 1.460] | [-.065, 1.521] | [-.727, .873] |
|  | (.208) | (.974) | (.672) | (.709) | (.403) | (.981) | (.319) | (.072) | (.858) |
| Florida | -.134 | -.076 | .165 | -.086 | -.100 | -.151 | .239 | .292 | -.054 |
|  | .122 | .161 | .188 | .171 | .161 | .201 | .190 | .264 | .201 |
|  | [-.374, .106] | [-.392, .239] | [-.203, .533] | [-.421, .249] | [-.416, .216] | [-.544, .243] | [-.133, .611] | [-.226, .810] | [-.449, .341] |
|  | (.274) | (.636) | (.379) | (.614) | (.536) | (.453) | (.208) | (.269) | (.788) |
| Georgia | -.207 | -.315 | .021 | -.224 | -.062 | -.334 | .191 | .036 | -.140 |
|  | .144 | .184 | .205 | .193 | .175 | .212 | .210 | .281 | .213 |
|  | [-.489, .074] | [-.675, .046] | [-.380, .423] | [-.601, .154] | [-.405, .280] | [-.750, .081] | [-.221, .604] | [-.515, .586] | [-.558, .278] |
|  | (.149) | (.087) | (.917) | (.245) | (.721) | (.114) | (.363) | (.899) | (.513) |
| Hawaii | .240 | .568 | .597 | .345 | .072 | -.108 | .414 | -.034 | .053 |
|  | .161 | .171 | .238 | .269 | .262 | .421 | .370 | .526 | .317 |
|  | [-.076, .555] | [.232, .903] | [.129, 1.064] | [-.183, .873] | [-.442, .585] | [-.934, .717] | [-.311, 1.139] | [-1.066, .998] | [-.569, .675] |
|  | (.136) | (.001) | (.012) | (.200) | (.784) | (.797) | (.263) | (.949) | (.867) |
| Idaho | -.355 | -.411 | .206 | -.316 | -.482 | -.344 | -.534 | .368 | -.295 |
|  | .298 | .326 | .279 | .322 | .296 | .297 | .296 | .389 | .321 |
|  | [-.938, .229] | [-1.051, .229] | [-.342, .753] | [-.946, .315] | [-1.062, .098] | [-.926, .239] | [-1.114, .047] | [-.394, 1.130] | [-.925, .334] |
|  | (.234) | (.208) | (.461) | (.327) | (.104) | (.247) | (.071) | (.344) | (.358) |
| Illinois | -.097 | -.092 | .156 | .055 | .042 | -.223 | .188 | .314 | -.077 |
|  | .132 | .175 | .198 | .181 | .175 | .217 | .209 | .280 | .212 |
|  | [-.355, .160] | [-.435, .251] | [-.232, .545] | [-.300, .411] | [-.301, .386] | [-.648, .201] | [-.222, .598] | [-.236, .864] | [-.491, .338] |
|  | (.459) | (.599) | (.430) | (.761) | (.810) | (.302) | (.369) | (.263) | (.717) |
| Indiana | .024 | .262 | .497 | .231 | .036 | -.243 | .071 | .695 | -.201 |
|  | .145 | .176 | .207 | .193 | .196 | .242 | .238 | .287 | .238 |
|  | [-.260, .309] | [-.083, .607] | [.090, .903] | [-.148, .609] | [-.349, .421] | [-.717, .232] | [-.396, .538] | [.133, 1.258] | [-.667, .266] |
|  | (.867) | (.136) | (.017) | (.232) | (.854) | (.316) | (.766) | (.015) | (.399) |
| Iowa | -.160 | -.176 | .053 | .078 | -.057 | .117 | .330 | .611 | -.057 |
|  | .194 | .231 | .268 | .225 | .203 | .244 | .289 | .310 | .277 |
|  | [-.540, .220] | [-.630, .277] | [-.472, .577] | [-.363, .518] | [-.455, .340] | [-.362, .596] | [-.237, .897] | [.004, 1.219] | [-.599, .486] |
|  | (.409) | (.446) | (.844) | (.729) | (.778) | (.631) | (.254) | (.049) | (.837) |
| Kansas | -.038 | .133 | -.003 | .326 | .116 | -.191 | .141 | .562 | -.179 |
|  | .228 | .313 | .274 | .253 | .327 | .338 | .390 | .368 | .336 |
|  | [-.485, .410] | [-.481, .747] | [-.540, .534] | [-.170, .823] | [-.525, .757] | [-.854, .472] | [-.624, .907] | [-.159, 1.284] | [-.838, .480] |
|  | (.869) | (.672) | (.991) | (.198) | (.722) | (.573) | (.717) | (.126) | (.594) |
| Kentucky | -.362 | -.130 | .041 | -.300 | -.063 | .122 | -.190 | .247 | -.076 |
|  | .182 | .213 | .246 | .228 | .202 | .240 | .243 | .320 | .237 |
|  | [-.720, -.004] | [-.548, .287] | [-.440, .523] | [-.747, .147] | [-.460, .333] | [-.349, .594] | [-.667, .286] | [-.381, .874] | [-.541, .389] |
|  | (.047) | (.540) | (.866) | (.188) | (.754) | (.611) | (.434) | (.441) | (.749) |
| Louisiana | -.195 | .154 | .049 | -.195 | -.109 | -.191 | .105 | .113 | -.137 |
|  | .213 | .223 | .264 | .251 | .233 | .285 | .255 | .343 | .252 |
|  | [-.614, .223] | [-.284, .592] | [-.470, .567] | [-.688, .298] | [-.565, .347] | [-.751, .368] | [-.396, .605] | [-.559, .785] | [-.632, .357] |
|  | (.360) | (.489) | (.854) | (.438) | (.640) | (.502) | (.682) | (.742) | (.586) |
| Maine | -.108 | -.003 | .784 | .412 | .172 | -.118 | -.357 | .360 | .301 |
|  | .293 | .285 | .213 | .330 | .246 | .369 | .358 | .410 | .287 |
|  | [-.683, .467] | [-.561, .556] | [.366, 1.203] | [-.236, 1.059] | [-.311, .655] | [-.841, .605] | [-1.059, .346] | [-.443, 1.164] | [-.262, .865] |
|  | (.713) | (.992) | (<.001) | (.213) | (.484) | (.749) | (.320) | (.379) | (.294) |
| Maryland | -.121 | -.018 | -.037 | -.188 | .166 | -.242 | .268 | .162 | -.074 |
|  | .164 | .207 | .251 | .230 | .201 | .254 | .274 | .310 | .255 |
|  | [-.442, .200] | [-.424, .388] | [-.529, .455] | [-.639, .263] | [-.229, .560] | [-.741, .256] | [-.269, .804] | [-.446, .769] | [-.575, .426] |
|  | (.460) | (.931) | (.883) | (.413) | (.410) | (.340) | (.328) | (.602) | (.771) |
| Massachusetts | -.032 | .085 | -.016 | -.027 | .086 | -.129 | .089 | .497 | -.326 |
|  | .153 | .194 | .223 | .218 | .186 | .248 | .227 | .291 | .245 |
|  | [-.332, .269] | [-.295, .465] | [-.454, .422] | [-.454, .400] | [-.278, .451] | [-.616, .358] | [-.356, .535] | [-.073, 1.068] | [-.806, .153] |
|  | (.836) | (.660) | (.943) | (.901) | (.643) | (.604) | (.694) | (.088) | (.182) |
| Michigan | -.126 | .015 | .266 | -.165 | -.120 | -.184 | .016 | .404 | -.095 |
|  | .138 | .169 | .195 | .190 | .176 | .215 | .215 | .277 | .215 |
|  | [-.398, .145] | [-.316, .347] | [-.117, .649] | [-.537, .207] | [-.465, .225] | [-.605, .237] | [-.406, .439] | [-.140, .948] | [-.516, .327] |
|  | (.362) | (.927) | (.173) | (.384) | (.495) | (.391) | (.939) | (.146) | (.659) |
| Minnesota | -.294 | -.162 | .145 | .007 | -.185 | -.203 | .030 | .245 | .025 |
|  | .180 | .211 | .252 | .210 | .198 | .247 | .256 | .328 | .250 |
|  | [-.647, .059] | [-.577, .252] | [-.350, .639] | [-.406, .419] | [-.574, .203] | [-.687, .282] | [-.473, .532] | [-.398, .887] | [-.466, .516] |
|  | (.103) | (.443) | (.566) | (.975) | (.350) | (.412) | (.908) | (.456) | (.921) |
| Mississippi | -.369 | -.305 | .159 | -.011 | -.044 | .074 | .259 | .107 | .057 |
|  | .251 | .267 | .259 | .237 | .231 | .260 | .277 | .355 | .250 |
|  | [-.861, .124] | [-.828, .218] | [-.348, .666] | [-.476, .454] | [-.497, .408] | [-.436, .583] | [-.284, .803] | [-.590, .804] | [-.434, .548] |
|  | (.142) | (.253) | (.538) | (.963) | (.848) | (.777) | (.350) | (.764) | (.819) |
| Missouri | -.296 | -.153 | -.036 | -.270 | -.004 | -.315 | .097 | .162 | -.091 |
|  | .165 | .188 | .214 | .211 | .182 | .239 | .222 | .295 | .231 |
|  | [-.621, .028] | [-.522, .216] | [-.455, .384] | [-.683, .143] | [-.360, .352] | [-.783, .154] | [-.337, .532] | [-.417, .741] | [-.545, .362] |
|  | (.073) | (.417) | (.867) | (.200) | (.983) | (.188) | (.660) | (.583) | (.693) |
| Montana | -.749 | -.715 | -.209 | -.587 | -.161 | -.799 | -.338 | -.424 | -.508 |
|  | .361 | .385 | .343 | .367 | .327 | .320 | .431 | .427 | .386 |
|  | [-1.457, -.041] | [-1.470, .040] | [-.881, .462] | [-1.306, .132] | [-.802, .480] | [-1.425, -.172] | [-1.184, .507] | [-1.260, .413] | [-1.265, .248] |
|  | (.038) | (.064) | (.541) | (.110) | (.622) | (.013) | (.432) | (.321) | (.188) |
| Nebraska | -.114 | .182 | -.423 | -.211 | -.018 | .073 | .399 | -.101 | -.101 |
|  | .227 | .252 | .369 | .343 | .283 | .376 | .452 | .423 | .323 |
|  | [-.558, .331] | [-.312, .676] | [-1.147, .300] | [-.883, .462] | [-.574, .537] | [-.663, .809] | [-.487, 1.285] | [-.930, .729] | [-.735, .532] |
|  | (.616) | (.470) | (.251) | (.539) | (.948) | (.846) | (.378) | (.812) | (.754) |
| Nevada | -.069 | .036 | .161 | -.019 | -.181 | -.355 | -.182 | .364 | .004 |
|  | .158 | .202 | .229 | .207 | .225 | .259 | .245 | .311 | .231 |
|  | [-.378, .241] | [-.360, .432] | [-.288, .610] | [-.425, .387] | [-.622, .260] | [-.863, .152] | [-.663, .299] | [-.245, .974] | [-.448, .456] |
|  | (.664) | (.858) | (.481) | (.927) | (.420) | (.170) | (.457) | (.241) | (.985) |
| New Hampshire | -.023 | .166 | -.064 | -.185 | -.006 | -.249 | .314 | .011 | .046 |
|  | .201 | .285 | .305 | .291 | .285 | .295 | .334 | .368 | .297 |
|  | [-.416, .371] | [-.393, .726] | [-.661, .533] | [-.756, .386] | [-.564, .553] | [-.828, .330] | [-.341, .968] | [-.710, .733] | [-.535, .628] |
|  | (.910) | (.560) | (.834) | (.526) | (.984) | (.399) | (.348) | (.976) | (.876) |
| New Jersey | -.104 | .179 | .189 | -.048 | .021 | .037 | .380 | .196 | -.030 |
|  | .165 | .191 | .218 | .201 | .195 | .231 | .228 | .289 | .231 |
|  | [-.427, .219] | [-.195, .553] | [-.239, .617] | [-.441, .345] | [-.362, .403] | [-.417, .490] | [-.068, .828] | [-.371, .762] | [-.482, .422] |
|  | (.527) | (.349) | (.386) | (.810) | (.916) | (.874) | (.096) | (.498) | (.897) |
| New Mexico | .144 | -.162 | .264 | -.137 | -.132 | -.332 | -.421 | .305 | -.358 |
|  | .174 | .281 | .270 | .278 | .243 | .289 | .334 | .355 | .288 |
|  | [-.197, .485] | [-.713, .389] | [-.264, .793] | [-.682, .409] | [-.609, .344] | [-.898, .235] | [-1.075, .234] | [-.391, 1.000] | [-.921, .206] |
|  | (.407) | (.565) | (.327) | (.623) | (.586) | (.251) | (.207) | (.391) | (.214) |
| New York | -.281 | -.031 | .104 | -.106 | -.091 | -.168 | .206 | .009 | -.045 |
|  | .132 | .163 | .193 | .178 | .165 | .204 | .196 | .273 | .207 |
|  | [-.539, -.022] | [-.352, .289] | [-.275, .482] | [-.454, .242] | [-.414, .232] | [-.568, .233] | [-.178, .590] | [-.526, .543] | [-.450, .361] |
|  | (.034) | (.847) | (.592) | (.551) | (.581) | (.412) | (.293) | (.974) | (.830) |
| North Carolina | -.134 | .071 | .052 | .083 | -.064 | -.375 | .243 | .066 | -.265 |
|  | .142 | .173 | .211 | .186 | .180 | .225 | .216 | .290 | .228 |
|  | [-.413, .145] | [-.268, .410] | [-.362, .466] | [-.282, .448] | [-.416, .288] | [-.815, .066] | [-.180, .666] | [-.503, .635] | [-.711, .181] |
|  | (.345) | (.680) | (.807) | (.656) | (.721) | (.095) | (.261) | (.821) | (.245) |
| North Dakota | -.160 | .415 | -.347 | -.250 | .247 | -.160 | .509 | -.048 | -.509 |
|  | .449 | .268 | .440 | .298 | .247 | .534 | .363 | .427 | .368 |
|  | [-1.040, .719] | [-.109, .940] | [-1.210, .516] | [-.834, .333] | [-.237, .731] | [-1.207, .888] | [-.202, 1.220] | [-.885, .789] | [-1.231, .214] |
|  | (.721) | (.121) | (.430) | (.400) | (.317) | (.765) | (.160) | (.911) | (.167) |
| Ohio | -.183 | -.021 | .172 | -.010 | -.007 | -.080 | -.035 | .361 | .064 |
|  | .138 | .168 | .203 | .188 | .168 | .219 | .206 | .280 | .209 |
|  | [-.454, .089] | [-.351, .309] | [-.227, .570] | [-.378, .358] | [-.336, .322] | [-.510, .349] | [-.438, .369] | [-.188, .910] | [-.345, .474] |
|  | (.187) | (.901) | (.398) | (.957) | (.968) | (.714) | (.866) | (.198) | (.757) |
| Oklahoma | -.268 | -.014 | .200 | .171 | -.142 | .249 | .405 | .023 | -.223 |
|  | .193 | .225 | .264 | .218 | .240 | .279 | .282 | .373 | .292 |
|  | [-.646, .110] | [-.455, .427] | [-.319, .718] | [-.256, .598] | [-.612, .328] | [-.297, .796] | [-.149, .958] | [-.708, .753] | [-.795, .350] |
|  | (.164) | (.950) | (.450) | (.432) | (.553) | (.371) | (.152) | (.951) | (.445) |
| Oregon | .064 | .149 | -.257 | -.120 | .007 | -.350 | -.079 | .292 | -.403 |
|  | .138 | .186 | .229 | .204 | .194 | .245 | .236 | .298 | .236 |
|  | [-.206, .334] | [-.216, .515] | [-.705, .191] | [-.520, .281] | [-.374, .388] | [-.831, .131] | [-.542, .383] | [-.292, .876] | [-.867, .060] |
|  | (.644) | (.423) | (.260) | (.558) | (.971) | (.154) | (.737) | (.327) | (.088) |
| Pennsylvania | -.125 | -.047 | .086 | -.035 | .001 | -.171 | -.001 | .327 | -.019 |
|  | .127 | .161 | .191 | .174 | .161 | .205 | .191 | .267 | .202 |
|  | [-.373, .123] | [-.362, .268] | [-.289, .462] | [-.376, .305] | [-.315, .317] | [-.572, .230] | [-.377, .374] | [-.197, .850] | [-.415, .377] |
|  | (.324) | (.768) | (.652) | (.840) | (.995) | (.403) | (.994) | (.221) | (.925) |
| Rhode Island | .325 | .298 | .301 | -.040 | .270 | .831 | .040 | -.104 | -.033 |
|  | .172 | .334 | .364 | .420 | .306 | .284 | .500 | .571 | .378 |
|  | [-.013, .662] | [-.356, .952] | [-.412, 1.014] | [-.864, .783] | [-.331, .870] | [.274, 1.388] | [-.940, 1.020] | [-1.224, 1.017] | [-.775, .709] |
|  | (.060) | (.372) | (.408) | (.924) | (.379) | (.003) | (.937) | (.856) | (.930) |
| South Carolina | -.125 | -.126 | .271 | .167 | -.309 | -.110 | .015 | .396 | -.029 |
|  | .198 | .233 | .228 | .215 | .241 | .248 | .261 | .302 | .250 |
|  | [-.513, .263] | [-.582, .331] | [-.176, .719] | [-.254, .589] | [-.782, .163] | [-.597, .377] | [-.496, .526] | [-.195, .987] | [-.519, .460] |
|  | (.528) | (.590) | (.235) | (.437) | (.200) | (.657) | (.954) | (.189) | (.907) |
| South Dakota | -.290 | -.172 | -.525 | -.262 | -.250 | -.201 | .207 | -.395 | -.071 |
|  | .425 | .332 | .347 | .375 | .438 | .445 | .374 | .413 | .245 |
|  | [-1.123, .542] | [-.824, .479] | [-1.207, .156] | [-.998, .474] | [-1.109, .609] | [-1.074, .673] | [-.525, .940] | [-1.205, .416] | [-.552, .410] |
|  | (.494) | (.604) | (.131) | (.486) | (.569) | (.652) | (.579) | (.340) | (.773) |
| Tennessee | .043 | .190 | .033 | -.273 | -.073 | -.207 | -.095 | .328 | -.221 |
|  | .150 | .204 | .240 | .232 | .204 | .236 | .244 | .315 | .252 |
|  | [-.252, .337] | [-.210, .591] | [-.438, .504] | [-.728, .182] | [-.472, .326] | [-.670, .256] | [-.573, .383] | [-.289, .945] | [-.714, .272] |
|  | (.776) | (.351) | (.890) | (.240) | (.720) | (.381) | (.697) | (.297) | (.380) |
| Texas | -.170 | -.100 | -.011 | -.107 | -.228 | -.275 | -.014 | .101 | -.093 |
|  | .127 | .167 | .193 | .177 | .169 | .205 | .195 | .272 | .205 |
|  | [-.419, .079] | [-.427, .226] | [-.389, .367] | [-.453, .240] | [-.559, .103] | [-.678, .128] | [-.397, .369] | [-.433, .635] | [-.494, .309] |
|  | (.180) | (.547) | (.953) | (.546) | (.177) | (.180) | (.943) | (.710) | (.651) |
| Utah | -.198 | -.023 | -.062 | .026 | -.049 | -.455 | .207 | .545 | -.298 |
|  | .209 | .240 | .257 | .258 | .232 | .330 | .289 | .321 | .281 |
|  | [-.609, .213] | [-.494, .449] | [-.566, .441] | [-.480, .532] | [-.503, .405] | [-1.102, .193] | [-.360, .774] | [-.084, 1.174] | [-.849, .253] |
|  | (.344) | (.925) | (.808) | (.920) | (.833) | (.169) | (.474) | (.089) | (.290) |
| Vermont | .052 | -.043 | .351 | .343 | -.326 | -.292 | .273 | .700 | -.047 |
|  | .180 | .341 | .336 | .274 | .363 | .354 | .480 | .411 | .366 |
|  | [-.302, .406] | [-.711, .625] | [-.308, 1.010] | [-.194, .880] | [-1.037, .385] | [-.986, .402] | [-.668, 1.215] | [-.106, 1.506] | [-.764, .671] |
|  | (.772) | (.899) | (.296) | (.211) | (.369) | (.409) | (.569) | (.089) | (.898) |
| Virginia | -.081 | -.040 | .014 | -.114 | -.034 | -.372 | .082 | .340 | .001 |
|  | .138 | .177 | .204 | .189 | .176 | .221 | .211 | .283 | .215 |
|  | [-.351, .189] | [-.387, .307] | [-.386, .414] | [-.485, .258] | [-.380, .312] | [-.806, .062] | [-.331, .496] | [-.215, .895] | [-.421, .424] |
|  | (.555) | (.822) | (.944) | (.549) | (.846) | (.093) | (.696) | (.230) | (.996) |
| Washington | -.075 | .005 | -.077 | -.126 | .078 | -.338 | -.216 | .350 | -.297 |
|  | .156 | .196 | .216 | .196 | .189 | .226 | .211 | .279 | .227 |
|  | [-.380, .231] | [-.380, .389] | [-.500, .346] | [-.509, .258] | [-.292, .448] | [-.781, .106] | [-.629, .198] | [-.196, .897] | [-.742, .148] |
|  | (.632) | (.982) | (.721) | (.522) | (.679) | (.136) | (.306) | (.209) | (.191) |
| West Virginia | -.603 | -.056 | .324 | -.014 | -.142 | -.334 | .126 | .452 | -<.001 |
|  | .273 | .235 | .261 | .262 | .261 | .286 | .308 | .343 | .291 |
|  | [-1.138, -.069] | [-.517, .405] | [-.189, .836] | [-.529, .500] | [-.653, .370] | [-.895, .227] | [-.478, .730] | [-.220, 1.124] | [-.570, .570] |
|  | (.027) | (.812) | (.216) | (.956) | (.587) | (.243) | (.683) | (.187) | (.999) |
| Wisconsin | -.337 | -.086 | .076 | -.091 | -.315 | -.354 | .042 | .316 | -.121 |
|  | .170 | .187 | .226 | .210 | .198 | .229 | .232 | .290 | .224 |
|  | [-.669, -.004] | [-.453, .282] | [-.367, .520] | [-.502, .321] | [-.702, .073] | [-.803, .095] | [-.412, .497] | [-.251, .884] | [-.560, .318] |
|  | (.047) | (.648) | (.736) | (.666) | (.111) | (.122) | (.855) | (.275) | (.588) |
| Wyoming | .640 | .745 | .294 | .003 | -.027 | -.260 | 1.296 | -.489 | -.236 |
|  | .176 | .282 | .708 | .759 | .594 | .673 | .415 | .988 | .595 |
|  | [.296, .985] | [.192, 1.299] | [-1.094, 1.683] | [-1.485, 1.492] | [-1.192, 1.139] | [-1.580, 1.060] | [.481, 2.110] | [-2.425, 1.448] | [-1.403, .931] |
|  | (<.001) | (.008) | (.678) | (.996) | (.964) | (.700) | (.002) | (.621) | (.692) |
| N | 2970 | 2974 | 2967 | 2973 | 2973 | 2973 | 2974 | 2975 | 2973 |
| Pseudo-R^2^ |  |  |  |  |  |  |  |  |  |
| Model χ^2^ |  |  |  |  |  |  |  |  |  |
| d.f. | 90 | 90 | 90 | 90 | 90 | 90 | 90 | 90 | 90 |
| p-value | <.001 | <.001 | <.001 | <.001 | <.001 | <.001 | <.001 | <.001 | <.001 |

**S11 Fig. Health Policies, by News Source.** Estimates are the predicted effects comparing Republicans and Others (unaffiliated or identifying with a third party) to self-identified Democrats for respondents who report watching right-wing news versus those who do not. Confidence intervals are not adjusted for multiple comparisons in this exploratory analysis.

S30 Table. Regression Results, Public Policies, Interacting with News Source.

|  | (1) | (2) | (3) | (4) | (5) | (6) | (7) | (8) | (9) | (10) |
| --- | --- | --- | --- | --- | --- | --- | --- | --- | --- | --- |
|  | increasetrade | importtaxes | lowinterest | notreliant | noairtravel | banentry | entrymexico | quarantine | rescueUS | delayelections |
| Republican | -.590 | .680 | .162 | .553 | .337 | .571 | .926 | .559 | -.203 | .410 |
|  | .063 | .065 | .062 | .062 | .070 | .068 | .060 | .079 | .071 | .083 |
|  | [-.713, -.466] | [.551, .808] | [.040, .283] | [.432, .674] | [.201, .474] | [.437, .704] | [.808, 1.044] | [.404, .714] | [-.343, -.063] | [.247, .573] |
|  | (<.001) | (<.001) | (.009) | (<.001) | (<.001) | (<.001) | (<.001) | (<.001) | (.005) | (<.001) |
| Other | -.462 | .188 | -.104 | .234 | .063 | .079 | .322 | .031 | -.192 | .144 |
|  | .047 | .052 | .050 | .052 | .056 | .056 | .057 | .059 | .055 | .067 |
|  | [-.553, -.371] | [.086, .289] | [-.202, -.007] | [.131, .336] | [-.047, .172] | [-.030, .189] | [.209, .434] | [-.085, .146] | [-.299, -.085] | [.013, .275] |
|  | (<.001) | (<.001) | (.036) | (<.001) | (.261) | (.156) | (<.001) | (.602) | (<.001) | (.032) |
| rightnews=1 | -.413 | .397 | .167 | .084 | .475 | .336 | .507 | .804 | -.305 | .120 |
|  | .180 | .179 | .162 | .184 | .175 | .180 | .181 | .218 | .197 | .253 |
|  | [-.767, -.060] | [.045, .748] | [-.149, .484] | [-.276, .444] | [.131, .819] | [-.016, .689] | [.152, .861] | [.376, 1.231] | [-.692, .081] | [-.376, .617] |
|  | (.022) | (.027) | (.300) | (.649) | (.007) | (.062) | (.005) | (<.001) | (.122) | (.635) |
| Republican # rightnews=1 | .284 | -.236 | .058 | .280 | -.378 | -.101 | -.288 | -.834 | .491 | -.292 |
|  | .196 | .195 | .176 | .193 | .193 | .194 | .189 | .240 | .215 | .272 |
|  | [-.101, .669] | [-.619, .148] | [-.287, .402] | [-.099, .659] | [-.757, .001] | [-.482, .279] | [-.659, .083] | [-1.304, -.365] | [.069, .912] | [-.825, .242] |
|  | (.148) | (.228) | (.743) | (.147) | (.050) | (.601) | (.128) | (.001) | (.022) | (.284) |
| Other # rightnews=1 | .052 | .263 | .239 | .663 | -.136 | .290 | .333 | -.506 | .348 | -.223 |
|  | .203 | .198 | .181 | .194 | .199 | .199 | .196 | .239 | .220 | .277 |
|  | [-.346, .450] | [-.126, .652] | [-.115, .593] | [.282, 1.043] | [-.527, .255] | [-.099, .679] | [-.051, .717] | [-.975, -.037] | [-.084, .780] | [-.767, .320] |
|  | (.797) | (.185) | (.186) | (.001) | (.495) | (.144) | (.089) | (.035) | (.114) | (.420) |
| 35- | -.078 | .149 | .162 | .284 | .196 | .128 | .182 | .116 | .039 | -.117 |
|  | .055 | .061 | .058 | .059 | .064 | .064 | .064 | .072 | .064 | .076 |
|  | [-.186, .031] | [.030, .268] | [.048, .275] | [.170, .399] | [.070, .322] | [.003, .252] | [.057, .307] | [-.025, .257] | [-.086, .165] | [-.266, .032] |
|  | (.160) | (.014) | (.005) | (<.001) | (.002) | (.045) | (.004) | (.106) | (.540) | (.124) |
| 50- | .019 | .075 | .173 | .382 | .150 | .233 | .332 | -.037 | .108 | -.371 |
|  | .053 | .057 | .055 | .056 | .062 | .060 | .061 | .069 | .060 | .073 |
|  | [-.085, .123] | [-.037, .188] | [.065, .280] | [.272, .491] | [.027, .272] | [.114, .351] | [.214, .451] | [-.172, .099] | [-.010, .227] | [-.513, -.229] |
|  | (.718) | (.190) | (.002) | (<.001) | (.016) | (<.001) | (<.001) | (.595) | (.073) | (<.001) |
| 70- | .097 | -.038 | .183 | .339 | .081 | .147 | .335 | -.223 | .263 | -.249 |
|  | .068 | .069 | .068 | .071 | .077 | .077 | .073 | .083 | .076 | .091 |
|  | [-.036, .229] | [-.174, .098] | [.050, .315] | [.200, .478] | [-.069, .231] | [-.003, .297] | [.192, .478] | [-.386, -.060] | [.115, .411] | [-.426, -.071] |
|  | (.155) | (.582) | (.007) | (<.001) | (.292) | (.055) | (<.001) | (.007) | (.001) | (.006) |
| gender | -.152 | .188 | .117 | -.029 | .271 | .142 | .047 | .112 | -.056 | .425 |
|  | .039 | .042 | .040 | .040 | .044 | .043 | .042 | .049 | .044 | .054 |
|  | [-.228, -.075] | [.105, .271] | [.039, .194] | [-.107, .049] | [.184, .358] | [.057, .227] | [-.036, .131] | [.016, .209] | [-.143, .031] | [.320, .530] |
|  | (<.001) | (<.001) | (.003) | (.460) | (<.001) | (.001) | (.264) | (.022) | (.206) | (<.001) |
| white | -.026 | -.103 | -.076 | -.106 | -.115 | -.038 | -.094 | -.279 | .046 | -.103 |
|  | .048 | .052 | .048 | .048 | .052 | .053 | .051 | .061 | .054 | .064 |
|  | [-.119, .067] | [-.204, -.001] | [-.170, .018] | [-.199, -.012] | [-.217, -.013] | [-.141, .066] | [-.195, .007] | [-.397, -.160] | [-.060, .152] | [-.228, .022] |
|  | (.585) | (.048) | (.113) | (.027) | (.028) | (.475) | (.068) | (<.001) | (.396) | (.108) |
| married | -.018 | .094 | -.033 | -.034 | .057 | -.011 | -.033 | .053 | -.063 | -.056 |
|  | .044 | .046 | .043 | .044 | .047 | .048 | .047 | .054 | .049 | .059 |
|  | [-.104, .067] | [.004, .184] | [-.117, .050] | [-.119, .051] | [-.035, .149] | [-.104, .082] | [-.125, .059] | [-.053, .160] | [-.159, .034] | [-.171, .059] |
|  | (.674) | (.042) | (.434) | (.436) | (.224) | (.817) | (.480) | (.327) | (.205) | (.342) |
| High school graduate | -.345 | -.088 | -.021 | -.072 | .024 | -.299 | -.160 | -.450 | .061 | -.006 |
|  | .111 | .121 | .123 | .113 | .118 | .110 | .109 | .145 | .134 | .151 |
|  | [-.563, -.126] | [-.326, .149] | [-.263, .220] | [-.294, .151] | [-.208, .256] | [-.514, -.084] | [-.373, .053] | [-.735, -.165] | [-.201, .323] | [-.303, .291] |
|  | (.002) | (.466) | (.863) | (.528) | (.838) | (.006) | (.142) | (.002) | (.650) | (.969) |
| Some college | -.318 | -.247 | -.059 | -.084 | -.212 | -.466 | -.307 | -.889 | .118 | -.215 |
|  | .114 | .124 | .126 | .117 | .122 | .113 | .114 | .147 | .136 | .155 |
|  | [-.543, -.094] | [-.491, -.003] | [-.306, .189] | [-.314, .146] | [-.452, .027] | [-.688, -.244] | [-.530, -.084] | [-1.178, -.600] | [-.149, .386] | [-.519, .089] |
|  | (.005) | (.047) | (.642) | (.475) | (.082) | (<.001) | (.007) | (<.001) | (.387) | (.166) |
| 2-year | -.281 | -.084 | -.029 | -.008 | -.108 | -.297 | -.203 | -.822 | .023 | -.105 |
|  | .118 | .129 | .131 | .121 | .128 | .119 | .118 | .155 | .142 | .163 |
|  | [-.514, -.049] | [-.337, .169] | [-.286, .229] | [-.246, .229] | [-.359, .144] | [-.530, -.064] | [-.434, .029] | [-1.126, -.517] | [-.255, .300] | [-.425, .214] |
|  | (.018) | (.514) | (.827) | (.946) | (.402) | (.012) | (.086) | (<.001) | (.873) | (.518) |
| 4-year | -.139 | -.365 | -.152 | -.239 | -.367 | -.639 | -.396 | -.947 | .069 | -.229 |
|  | .117 | .126 | .127 | .119 | .127 | .118 | .117 | .152 | .138 | .159 |
|  | [-.367, .090] | [-.613, -.117] | [-.401, .097] | [-.473, -.005] | [-.616, -.119] | [-.871, -.408] | [-.625, -.167] | [-1.246, -.648] | [-.201, .339] | [-.540, .082] |
|  | (.233) | (.004) | (.232) | (.045) | (.004) | (<.001) | (.001) | (<.001) | (.615) | (.149) |
| Post-grad | -.086 | -.450 | -.177 | -.305 | -.557 | -.619 | -.519 | -1.113 | .016 | -.437 |
|  | .123 | .135 | .133 | .128 | .136 | .126 | .126 | .159 | .146 | .170 |
|  | [-.326, .155] | [-.715, -.186] | [-.439, .084] | [-.556, -.055] | [-.823, -.290] | [-.865, -.372] | [-.767, -.271] | [-1.425, -.800] | [-.269, .302] | [-.770, -.104] |
|  | (.486) | (.001) | (.184) | (.017) | (<.001) | (<.001) | (<.001) | (<.001) | (.911) | (.010) |
| income=2 | -.188 | .124 | .132 | -.010 | .147 | .148 | .162 | -.015 | .017 | .167 |
|  | .111 | .118 | .117 | .116 | .119 | .117 | .124 | .136 | .131 | .155 |
|  | [-.405, .030] | [-.109, .356] | [-.097, .360] | [-.239, .218] | [-.086, .380] | [-.081, .378] | [-.081, .405] | [-.281, .251] | [-.240, .274] | [-.136, .471] |
|  | (.090) | (.296) | (.260) | (.929) | (.217) | (.205) | (.191) | (.912) | (.896) | (.279) |
| income=3 | -.077 | .087 | .139 | .125 | .082 | .147 | .226 | -.245 | .149 | .205 |
|  | .105 | .114 | .116 | .110 | .115 | .115 | .119 | .131 | .125 | .152 |
|  | [-.283, .128] | [-.135, .310] | [-.087, .366] | [-.090, .340] | [-.144, .307] | [-.079, .373] | [-.008, .460] | [-.502, .013] | [-.097, .395] | [-.093, .503] |
|  | (.461) | (.441) | (.228) | (.256) | (.477) | (.201) | (.059) | (.063) | (.235) | (.178) |
| income=4 | -.070 | .061 | .226 | .066 | .052 | -.022 | .128 | -.260 | .016 | .118 |
|  | .108 | .118 | .117 | .113 | .118 | .123 | .123 | .137 | .129 | .154 |
|  | [-.283, .142] | [-.171, .293] | [-.002, .455] | [-.156, .288] | [-.180, .285] | [-.262, .219] | [-.113, .368] | [-.528, .009] | [-.236, .268] | [-.183, .420] |
|  | (.515) | (.605) | (.052) | (.560) | (.658) | (.860) | (.299) | (.058) | (.901) | (.442) |
| income=5 | -.069 | -.004 | .097 | .181 | .042 | .079 | .226 | -.435 | .132 | .029 |
|  | .108 | .116 | .120 | .112 | .121 | .122 | .125 | .134 | .129 | .155 |
|  | [-.281, .144] | [-.231, .223] | [-.137, .332] | [-.038, .401] | [-.195, .278] | [-.160, .318] | [-.018, .471] | [-.697, -.173] | [-.122, .386] | [-.275, .334] |
|  | (.527) | (.973) | (.416) | (.105) | (.730) | (.518) | (.070) | (.001) | (.308) | (.850) |
| income=6 | -.079 | -.033 | .128 | .252 | .096 | .131 | .242 | -.321 | .088 | .189 |
|  | .108 | .122 | .121 | .114 | .121 | .122 | .125 | .140 | .130 | .161 |
|  | [-.291, .134] | [-.272, .207] | [-.108, .365] | [.028, .476] | [-.142, .334] | [-.108, .370] | [-.003, .486] | [-.595, -.047] | [-.167, .342] | [-.128, .505] |
|  | (.467) | (.790) | (.288) | (.028) | (.428) | (.283) | (.052) | (.021) | (.500) | (.242) |
| income=7 | -.141 | -.031 | .152 | .141 | .227 | .272 | .140 | -.312 | -.077 | .053 |
|  | .118 | .126 | .125 | .124 | .123 | .131 | .138 | .153 | .143 | .168 |
|  | [-.373, .091] | [-.277, .216] | [-.094, .398] | [-.102, .384] | [-.015, .468] | [.016, .529] | [-.130, .411] | [-.612, -.012] | [-.358, .205] | [-.276, .383] |
|  | (.235) | (.808) | (.225) | (.255) | (.066) | (.037) | (.308) | (.042) | (.593) | (.751) |
| income=8 | -.173 | -.063 | .168 | .080 | -.002 | <.001 | .148 | -.376 | -.008 | .032 |
|  | .118 | .127 | .123 | .121 | .128 | .131 | .129 | .145 | .138 | .165 |
|  | [-.404, .057] | [-.312, .186] | [-.074, .409] | [-.158, .317] | [-.253, .249] | [-.256, .257] | [-.105, .400] | [-.660, -.092] | [-.278, .262] | [-.291, .355] |
|  | (.140) | (.618) | (.174) | (.510) | (.986) | (.997) | (.251) | (.009) | (.952) | (.847) |
| income=9 | .022 | -.183 | .151 | .041 | -.191 | .076 | .044 | -.481 | <.001 | -.166 |
|  | .115 | .123 | .124 | .122 | .131 | .130 | .136 | .140 | .140 | .165 |
|  | [-.203, .248] | [-.424, .057] | [-.093, .395] | [-.198, .280] | [-.447, .065] | [-.180, .332] | [-.222, .311] | [-.755, -.206] | [-.274, .275] | [-.489, .157] |
|  | (.846) | (.136) | (.224) | (.736) | (.144) | (.562) | (.744) | (.001) | (.998) | (.314) |
| income=10 | .015 | -.183 | .161 | -.001 | -.032 | -.033 | .138 | -.515 | .019 | -.182 |
|  | .116 | .129 | .127 | .128 | .139 | .136 | .140 | .151 | .143 | .174 |
|  | [-.213, .243] | [-.436, .071] | [-.088, .410] | [-.251, .250] | [-.305, .240] | [-.300, .234] | [-.136, .412] | [-.810, -.219] | [-.261, .299] | [-.523, .159] |
|  | (.897) | (.157) | (.204) | (.996) | (.816) | (.809) | (.323) | (.001) | (.894) | (.296) |
| income=11 | -.015 | -.219 | .170 | -.098 | -.180 | -.015 | .042 | -.628 | -.038 | .050 |
|  | .121 | .139 | .133 | .132 | .148 | .147 | .143 | .152 | .144 | .178 |
|  | [-.252, .222] | [-.492, .053] | [-.091, .431] | [-.357, .162] | [-.471, .111] | [-.302, .273] | [-.238, .323] | [-.925, -.330] | [-.320, .243] | [-.299, .400] |
|  | (.903) | (.115) | (.203) | (.461) | (.225) | (.919) | (.768) | (<.001) | (.790) | (.778) |
| income=12 | -.065 | -.160 | .126 | -.106 | -.104 | .027 | .224 | -.654 | -.008 | -.187 |
|  | .138 | .160 | .147 | .153 | .162 | .159 | .164 | .168 | .160 | .194 |
|  | [-.337, .206] | [-.474, .154] | [-.162, .415] | [-.406, .193] | [-.422, .213] | [-.285, .339] | [-.096, .545] | [-.984, -.325] | [-.322, .306] | [-.568, .195] |
|  | (.637) | (.319) | (.391) | (.485) | (.519) | (.865) | (.170) | (<.001) | (.961) | (.337) |
| income=13 | .119 | -.405 | -.155 | -.199 | -.278 | .072 | .045 | -.816 | -.170 | -.257 |
|  | .155 | .189 | .213 | .188 | .238 | .204 | .208 | .210 | .210 | .272 |
|  | [-.185, .423] | [-.775, -.034] | [-.573, .263] | [-.567, .170] | [-.745, .190] | [-.328, .472] | [-.363, .453] | [-1.228, -.403] | [-.581, .241] | [-.790, .275] |
|  | (.442) | (.032) | (.468) | (.290) | (.244) | (.724) | (.830) | (<.001) | (.417) | (.344) |
| income=14 | -.098 | -.178 | .133 | .041 | -.134 | .134 | .183 | -.713 | -.324 | .044 |
|  | .201 | .221 | .166 | .218 | .254 | .215 | .226 | .211 | .235 | .299 |
|  | [-.492, .296] | [-.610, .255] | [-.193, .459] | [-.387, .468] | [-.631, .364] | [-.287, .556] | [-.260, .625] | [-1.127, -.299] | [-.785, .137] | [-.542, .629] |
|  | (.626) | (.421) | (.424) | (.852) | (.598) | (.533) | (.419) | (.001) | (.169) | (.883) |
| income=15 | .197 | -.155 | -.388 | .496 | .741 | .753 | -.348 | -.223 | 1.097 | .488 |
|  | .708 | .901 | .583 | .522 | .283 | .211 | .915 | .938 | .357 | .767 |
|  | [-1.191, 1.585] | [-1.923, 1.612] | [-1.532, .756] | [-.528, 1.520] | [.187, 1.295] | [.340, 1.167] | [-2.142, 1.445] | [-2.062, 1.617] | [.397, 1.798] | [-1.016, 1.991] |
|  | (.781) | (.863) | (.506) | (.343) | (.009) | (<.001) | (.703) | (.812) | (.002) | (.525) |
| income=16 | -.164 | .014 | .352 | -.082 | .064 | .439 | .552 | -.652 | -.166 | .090 |
|  | .303 | .318 | .280 | .313 | .297 | .228 | .214 | .375 | .423 | .358 |
|  | [-.758, .429] | [-.609, .638] | [-.196, .901] | [-.695, .531] | [-.518, .646] | [-.008, .886] | [.132, .973] | [-1.388, .083] | [-.995, .662] | [-.611, .792] |
|  | (.587) | (.964) | (.208) | (.793) | (.830) | (.054) | (.010) | (.082) | (.694) | (.800) |
| income=17 | -.234 | -.004 | -.024 | .081 | -.071 | .088 | .171 | -.345 | -.078 | -.034 |
|  | .102 | .110 | .113 | .106 | .113 | .113 | .117 | .129 | .121 | .145 |
|  | [-.434, -.033] | [-.220, .213] | [-.245, .197] | [-.127, .290] | [-.293, .150] | [-.134, .310] | [-.058, .400] | [-.599, -.092] | [-.315, .160] | [-.319, .251] |
|  | (.022) | (.974) | (.832) | (.445) | (.528) | (.435) | (.143) | (.008) | (.522) | (.816) |
| Rural Code=2 | -.106 | -.050 | -.072 | .055 | -.053 | -.049 | -.094 | -.030 | -.116 | -.151 |
|  | .052 | .055 | .052 | .050 | .060 | .056 | .056 | .063 | .059 | .072 |
|  | [-.208, -.003] | [-.158, .058] | [-.174, .029] | [-.043, .154] | [-.170, .064] | [-.159, .060] | [-.204, .016] | [-.155, .094] | [-.232, -<.001] | [-.292, -.010] |
|  | (.043) | (.362) | (.164) | (.273) | (.377) | (.377) | (.093) | (.633) | (.050) | (.036) |
| Rural Code=3 | -.088 | -.040 | -.021 | .038 | .015 | .081 | .009 | .062 | -.137 | .011 |
|  | .071 | .073 | .069 | .067 | .078 | .076 | .072 | .085 | .080 | .093 |
|  | [-.228, .052] | [-.184, .103] | [-.157, .115] | [-.094, .169] | [-.138, .168] | [-.068, .230] | [-.132, .149] | [-.105, .229] | [-.294, .019] | [-.171, .193] |
|  | (.218) | (.582) | (.763) | (.576) | (.848) | (.288) | (.902) | (.468) | (.085) | (.905) |
| Rural Code=4 | -.205 | .042 | .068 | .113 | .048 | .039 | .243 | .134 | .061 | -.196 |
|  | .098 | .101 | .099 | .098 | .108 | .104 | .095 | .116 | .113 | .127 |
|  | [-.397, -.012] | [-.157, .240] | [-.125, .262] | [-.080, .305] | [-.163, .259] | [-.164, .242] | [.058, .429] | [-.092, .361] | [-.160, .282] | [-.445, .053] |
|  | (.037) | (.682) | (.488) | (.252) | (.655) | (.703) | (.010) | (.246) | (.588) | (.122) |
| Rural Code=5 | -.096 | -.042 | -.133 | -.086 | .191 | .007 | .043 | -.031 | -.333 | .130 |
|  | .134 | .131 | .152 | .134 | .158 | .169 | .171 | .194 | .192 | .204 |
|  | [-.359, .167] | [-.298, .214] | [-.431, .165] | [-.349, .177] | [-.118, .501] | [-.324, .337] | [-.292, .379] | [-.411, .350] | [-.710, .043] | [-.271, .531] |
|  | (.475) | (.748) | (.381) | (.521) | (.226) | (.968) | (.801) | (.875) | (.083) | (.524) |
| Rural Code=6 | -.118 | .105 | .061 | .078 | .109 | .071 | .071 | .206 | -.076 | -.010 |
|  | .110 | .107 | .095 | .096 | .101 | .104 | .097 | .124 | .110 | .131 |
|  | [-.334, .098] | [-.105, .314] | [-.126, .247] | [-.111, .267] | [-.090, .307] | [-.133, .276] | [-.120, .262] | [-.038, .450] | [-.290, .139] | [-.267, .248] |
|  | (.283) | (.327) | (.525) | (.420) | (.283) | (.494) | (.468) | (.098) | (.490) | (.941) |
| Rural Code=7 | -.057 | -.032 | -.237 | .054 | -.002 | .232 | -.048 | .005 | .060 | -.133 |
|  | .117 | .147 | .135 | .138 | .148 | .143 | .151 | .170 | .141 | .176 |
|  | [-.286, .172] | [-.321, .257] | [-.502, .029] | [-.218, .325] | [-.292, .288] | [-.048, .511] | [-.345, .249] | [-.329, .338] | [-.215, .336] | [-.479, .213] |
|  | (.625) | (.827) | (.081) | (.698) | (.988) | (.104) | (.751) | (.978) | (.667) | (.451) |
| Rural Code=8 | -.514 | -.085 | -.106 | .212 | .159 | -.103 | .198 | -.069 | .355 | .062 |
|  | .274 | .203 | .206 | .226 | .236 | .310 | .202 | .347 | .267 | .337 |
|  | [-1.051, .022] | [-.484, .313] | [-.510, .299] | [-.231, .654] | [-.303, .622] | [-.711, .504] | [-.198, .593] | [-.750, .612] | [-.169, .879] | [-.599, .723] |
|  | (.060) | (.674) | (.608) | (.348) | (.499) | (.739) | (.327) | (.842) | (.184) | (.855) |
| Rural Code=9 | -.093 | -.107 | -.094 | -.090 | -.079 | -.045 | -.385 | -.228 | -.375 | .228 |
|  | .211 | .210 | .168 | .237 | .239 | .209 | .239 | .221 | .170 | .240 |
|  | [-.507, .320] | [-.518, .304] | [-.424, .236] | [-.554, .374] | [-.548, .389] | [-.454, .364] | [-.854, .085] | [-.662, .207] | [-.709, -.042] | [-.242, .698] |
|  | (.658) | (.608) | (.575) | (.704) | (.740) | (.830) | (.108) | (.304) | (.027) | (.343) |
| Alaska | -.059 | .755 | .218 | .229 | .073 | -.538 | .035 | -.028 | -.087 | -.345 |
|  | .375 | .348 | .295 | .251 | .526 | .509 | .335 | .527 | .347 | .682 |
|  | [-.793, .675] | [.072, 1.438] | [-.360, .796] | [-.263, .720] | [-.958, 1.104] | [-1.537, .460] | [-.623, .692] | [-1.062, 1.006] | [-.768, .594] | [-1.681, .991] |
|  | (.875) | (.030) | (.459) | (.362) | (.890) | (.290) | (.918) | (.957) | (.803) | (.613) |
| Arizona | .015 | -.178 | -.460 | -.316 | -.393 | -.086 | -.437 | -.330 | -.102 | -.801 |
|  | .185 | .223 | .185 | .213 | .256 | .235 | .208 | .252 | .227 | .290 |
|  | [-.348, .379] | [-.616, .260] | [-.823, -.098] | [-.734, .101] | [-.894, .109] | [-.547, .376] | [-.845, -.029] | [-.824, .163] | [-.546, .343] | [-1.369, -.232] |
|  | (.934) | (.426) | (.013) | (.138) | (.125) | (.716) | (.036) | (.189) | (.653) | (.006) |
| Arkansas | .065 | .236 | -.068 | .100 | .002 | .191 | .084 | .123 | -.006 | -.010 |
|  | .211 | .246 | .216 | .217 | .270 | .257 | .226 | .282 | .245 | .323 |
|  | [-.350, .479] | [-.247, .719] | [-.491, .355] | [-.326, .526] | [-.529, .532] | [-.314, .695] | [-.358, .527] | [-.429, .675] | [-.487, .475] | [-.644, .624] |
|  | (.760) | (.338) | (.753) | (.645) | (.995) | (.459) | (.709) | (.663) | (.981) | (.976) |
| California | -.041 | .033 | -.279 | .035 | -.167 | -.031 | -.090 | -.041 | .007 | -.482 |
|  | .159 | .204 | .156 | .187 | .227 | .203 | .175 | .230 | .197 | .260 |
|  | [-.353, .270] | [-.366, .433] | [-.584, .026] | [-.332, .401] | [-.613, .279] | [-.430, .368] | [-.433, .253] | [-.491, .409] | [-.379, .393] | [-.993, .029] |
|  | (.794) | (.870) | (.073) | (.853) | (.463) | (.878) | (.606) | (.857) | (.972) | (.064) |
| Colorado | -.395 | .180 | -.147 | -.073 | -.256 | .068 | -.083 | -.283 | -.337 | -.219 |
|  | .227 | .237 | .183 | .248 | .284 | .260 | .233 | .278 | .256 | .314 |
|  | [-.840, .051] | [-.284, .644] | [-.506, .212] | [-.559, .413] | [-.813, .301] | [-.441, .577] | [-.539, .373] | [-.827, .261] | [-.838, .164] | [-.835, .397] |
|  | (.082) | (.446) | (.422) | (.768) | (.367) | (.795) | (.721) | (.308) | (.188) | (.486) |
| Connecticut | .391 | .264 | -.233 | -.158 | -.172 | .386 | .098 | .085 | .184 | -.275 |
|  | .201 | .283 | .249 | .245 | .314 | .262 | .243 | .305 | .277 | .357 |
|  | [-.004, .786] | [-.292, .819] | [-.720, .255] | [-.638, .323] | [-.788, .443] | [-.127, .900] | [-.378, .574] | [-.513, .683] | [-.359, .727] | [-.975, .425] |
|  | (.052) | (.352) | (.350) | (.520) | (.583) | (.140) | (.687) | (.780) | (.506) | (.441) |
| Delaware | .070 | .179 | .117 | .004 | .063 | -.219 | .217 | -.112 | .229 | -.894 |
|  | .279 | .301 | .288 | .241 | .358 | .335 | .285 | .406 | .369 | .469 |
|  | [-.477, .617] | [-.412, .769] | [-.448, .683] | [-.469, .476] | [-.638, .764] | [-.876, .439] | [-.341, .775] | [-.908, .684] | [-.494, .952] | [-1.812, .025] |
|  | (.802) | (.553) | (.684) | (.988) | (.860) | (.515) | (.446) | (.782) | (.534) | (.057) |
| District of Columbia | -.134 | -.160 | .035 | -.489 | -.524 | .224 | .079 | -.376 | .026 | .483 |
|  | .250 | .364 | .274 | .373 | .460 | .470 | .522 | .344 | .386 | .516 |
|  | [-.624, .357] | [-.875, .554] | [-.503, .573] | [-1.221, .243] | [-1.425, .378] | [-.698, 1.146] | [-.944, 1.103] | [-1.050, .299] | [-.731, .783] | [-.530, 1.495] |
|  | (.593) | (.660) | (.899) | (.190) | (.255) | (.633) | (.879) | (.275) | (.946) | (.350) |
| Florida | -.082 | .089 | -.124 | -.110 | -.222 | .070 | -.157 | .057 | -.020 | -.527 |
|  | .160 | .205 | .156 | .187 | .227 | .202 | .173 | .232 | .200 | .260 |
|  | [-.396, .232] | [-.313, .492] | [-.430, .181] | [-.477, .258] | [-.668, .223] | [-.327, .467] | [-.497, .183] | [-.399, .512] | [-.413, .373] | [-1.037, -.016] |
|  | (.609) | (.663) | (.425) | (.559) | (.328) | (.730) | (.364) | (.807) | (.921) | (.043) |
| Georgia | -.160 | .119 | -.272 | -.073 | -.396 | .070 | -.095 | .019 | -.191 | -.266 |
|  | .175 | .217 | .175 | .202 | .244 | .215 | .186 | .249 | .218 | .278 |
|  | [-.503, .184] | [-.305, .544] | [-.615, .071] | [-.469, .324] | [-.874, .083] | [-.351, .491] | [-.460, .271] | [-.468, .507] | [-.618, .236] | [-.812, .279] |
|  | (.363) | (.582) | (.120) | (.720) | (.105) | (.744) | (.612) | (.938) | (.380) | (.339) |
| Hawaii | .320 | .092 | .057 | -.458 | -.116 | .518 | -.290 | .123 | -.199 | -.135 |
|  | .284 | .324 | .282 | .490 | .473 | .318 | .382 | .449 | .401 | .472 |
|  | [-.236, .876] | [-.543, .728] | [-.496, .611] | [-1.419, .504] | [-1.042, .811] | [-.105, 1.141] | [-1.038, .458] | [-.757, 1.004] | [-.986, .587] | [-1.061, .790] |
|  | (.259) | (.775) | (.839) | (.351) | (.807) | (.103) | (.447) | (.784) | (.619) | (.774) |
| Idaho | -.241 | .003 | -.087 | -.074 | -.515 | -.359 | -.429 | .078 | -.442 | -.053 |
|  | .258 | .337 | .252 | .265 | .319 | .347 | .296 | .372 | .326 | .388 |
|  | [-.746, .265] | [-.659, .664] | [-.581, .407] | [-.593, .445] | [-1.140, .111] | [-1.040, .322] | [-1.010, .152] | [-.651, .808] | [-1.081, .198] | [-.814, .709] |
|  | (.351) | (.994) | (.730) | (.780) | (.107) | (.301) | (.148) | (.834) | (.176) | (.892) |
| Illinois | -.170 | .185 | -.222 | .133 | -.094 | .146 | .009 | .218 | -.170 | -.118 |
|  | .176 | .217 | .174 | .197 | .242 | .217 | .193 | .254 | .216 | .274 |
|  | [-.515, .174] | [-.239, .610] | [-.563, .119] | [-.253, .520] | [-.570, .381] | [-.280, .571] | [-.369, .386] | [-.281, .716] | [-.594, .254] | [-.655, .420] |
|  | (.332) | (.392) | (.202) | (.499) | (.697) | (.502) | (.964) | (.392) | (.431) | (.667) |
| Indiana | -.020 | -.015 | -.066 | .002 | -.192 | .047 | -.124 | -.175 | -.006 | -.200 |
|  | .191 | .238 | .195 | .214 | .257 | .240 | .218 | .277 | .243 | .304 |
|  | [-.395, .354] | [-.482, .452] | [-.448, .316] | [-.418, .421] | [-.696, .313] | [-.424, .517] | [-.551, .304] | [-.718, .367] | [-.482, .470] | [-.795, .395] |
|  | (.915) | (.948) | (.734) | (.994) | (.457) | (.846) | (.571) | (.527) | (.980) | (.510) |
| Iowa | -.100 | .268 | -.304 | -.032 | -.518 | .293 | .041 | .088 | -.197 | -.144 |
|  | .223 | .260 | .243 | .255 | .307 | .274 | .263 | .317 | .275 | .351 |
|  | [-.538, .338] | [-.242, .778] | [-.780, .172] | [-.531, .467] | [-1.120, .083] | [-.245, .831] | [-.475, .557] | [-.533, .709] | [-.736, .342] | [-.831, .544] |
|  | (.654) | (.303) | (.211) | (.900) | (.091) | (.285) | (.876) | (.781) | (.474) | (.682) |
| Kansas | -.543 | -.248 | -.200 | .160 | -.059 | .247 | -.067 | -.469 | -.432 | -.724 |
|  | .330 | .327 | .286 | .237 | .334 | .267 | .270 | .332 | .412 | .434 |
|  | [-1.191, .105] | [-.888, .392] | [-.761, .361] | [-.306, .625] | [-.714, .595] | [-.276, .770] | [-.596, .461] | [-1.121, .183] | [-1.239, .375] | [-1.574, .127] |
|  | (.100) | (.448) | (.484) | (.502) | (.859) | (.354) | (.802) | (.158) | (.294) | (.095) |
| Kentucky | -.222 | .386 | -.055 | .169 | -.098 | .262 | .282 | .017 | .152 | -.268 |
|  | .234 | .241 | .203 | .243 | .282 | .230 | .220 | .285 | .255 | .319 |
|  | [-.681, .237] | [-.086, .858] | [-.452, .343] | [-.307, .645] | [-.652, .455] | [-.188, .712] | [-.149, .713] | [-.542, .575] | [-.348, .652] | [-.893, .357] |
|  | (.343) | (.109) | (.788) | (.486) | (.728) | (.254) | (.199) | (.953) | (.551) | (.401) |
| Louisiana | -.226 | .389 | -.164 | -.201 | -.180 | .436 | .094 | .181 | -.053 | -.106 |
|  | .242 | .258 | .236 | .249 | .284 | .228 | .226 | .284 | .261 | .348 |
|  | [-.700, .248] | [-.116, .894] | [-.627, .300] | [-.689, .287] | [-.738, .378] | [-.011, .883] | [-.349, .537] | [-.375, .737] | [-.565, .459] | [-.789, .578] |
|  | (.350) | (.131) | (.489) | (.420) | (.527) | (.056) | (.677) | (.522) | (.840) | (.761) |
| Maine | -.375 | .196 | .109 | .048 | .258 | .242 | .268 | -.005 | -.465 | -.523 |
|  | .349 | .322 | .257 | .269 | .359 | .287 | .376 | .461 | .396 | .438 |
|  | [-1.060, .309] | [-.435, .826] | [-.395, .612] | [-.479, .575] | [-.446, .962] | [-.321, .805] | [-.469, 1.004] | [-.910, .899] | [-1.241, .310] | [-1.382, .336] |
|  | (.282) | (.542) | (.672) | (.857) | (.473) | (.400) | (.476) | (.991) | (.240) | (.233) |
| Maryland | .119 | -.119 | -.537 | -.310 | -.261 | -.034 | -.178 | -.176 | -.110 | -.265 |
|  | .190 | .253 | .225 | .250 | .288 | .286 | .247 | .288 | .254 | .337 |
|  | [-.254, .491] | [-.616, .378] | [-.979, -.096] | [-.799, .180] | [-.827, .304] | [-.594, .527] | [-.663, .307] | [-.741, .389] | [-.607, .387] | [-.925, .396] |
|  | (.532) | (.639) | (.017) | (.214) | (.365) | (.906) | (.472) | (.541) | (.664) | (.432) |
| Massachusetts | -.103 | -.190 | -.078 | -.242 | -.371 | .065 | -.088 | -.415 | .005 | -.039 |
|  | .193 | .230 | .188 | .226 | .269 | .245 | .215 | .251 | .232 | .304 |
|  | [-.482, .276] | [-.640, .261] | [-.447, .291] | [-.686, .201] | [-.899, .156] | [-.414, .545] | [-.509, .333] | [-.908, .077] | [-.450, .460] | [-.635, .557] |
|  | (.594) | (.410) | (.678) | (.285) | (.168) | (.790) | (.681) | (.098) | (.984) | (.899) |
| Michigan | -.155 | .151 | -.136 | -.094 | -.284 | .082 | -.034 | -.142 | -.222 | -.458 |
|  | .182 | .218 | .168 | .203 | .244 | .215 | .192 | .242 | .212 | .278 |
|  | [-.511, .202] | [-.277, .579] | [-.466, .194] | [-.492, .303] | [-.762, .195] | [-.340, .504] | [-.410, .342] | [-.616, .332] | [-.637, .193] | [-1.004, .087] |
|  | (.395) | (.490) | (.418) | (.643) | (.245) | (.703) | (.860) | (.556) | (.295) | (.100) |
| Minnesota | -.223 | .135 | -.571 | .115 | -.269 | -.103 | -.050 | -.400 | -.359 | -.746 |
|  | .215 | .264 | .222 | .215 | .287 | .263 | .234 | .270 | .265 | .326 |
|  | [-.645, .199] | [-.383, .653] | [-1.007, -.135] | [-.306, .537] | [-.831, .293] | [-.619, .414] | [-.509, .409] | [-.929, .128] | [-.878, .160] | [-1.386, -.106] |
|  | (.301) | (.610) | (.010) | (.592) | (.348) | (.697) | (.831) | (.138) | (.175) | (.022) |
| Mississippi | -.069 | .206 | -.604 | .022 | -.330 | -.233 | .161 | -.058 | -.319 | -.093 |
|  | .220 | .257 | .255 | .246 | .301 | .287 | .228 | .328 | .270 | .357 |
|  | [-.500, .361] | [-.299, .710] | [-1.104, -.103] | [-.462, .505] | [-.920, .260] | [-.797, .330] | [-.285, .607] | [-.700, .585] | [-.848, .209] | [-.793, .607] |
|  | (.753) | (.425) | (.018) | (.930) | (.272) | (.417) | (.479) | (.860) | (.236) | (.795) |
| Missouri | -.275 | .323 | -.170 | .090 | -.221 | .296 | .001 | .008 | -.173 | -.050 |
|  | .195 | .233 | .184 | .205 | .259 | .218 | .196 | .262 | .229 | .295 |
|  | [-.658, .108] | [-.134, .779] | [-.531, .191] | [-.311, .492] | [-.729, .287] | [-.132, .724] | [-.382, .385] | [-.505, .520] | [-.623, .276] | [-.628, .527] |
|  | (.160) | (.166) | (.356) | (.659) | (.394) | (.175) | (.995) | (.977) | (.450) | (.864) |
| Montana | .161 | -.131 | -.448 | .035 | -1.127 | -.749 | -.832 | -.320 | -.572 | -1.263 |
|  | .252 | .384 | .274 | .267 | .412 | .370 | .368 | .400 | .369 | .444 |
|  | [-.334, .655] | [-.884, .621] | [-.985, .089] | [-.488, .558] | [-1.936, -.319] | [-1.475, -.023] | [-1.555, -.110] | [-1.104, .465] | [-1.296, .153] | [-2.134, -.393] |
|  | (.524) | (.732) | (.102) | (.895) | (.006) | (.043) | (.024) | (.424) | (.122) | (.004) |
| Nebraska | -.315 | .238 | .019 | .458 | -.155 | -.237 | -.127 | -.198 | -.074 | -.299 |
|  | .402 | .399 | .279 | .283 | .427 | .425 | .335 | .443 | .367 | .498 |
|  | [-1.104, .474] | [-.544, 1.019] | [-.527, .565] | [-.097, 1.014] | [-.993, .683] | [-1.069, .596] | [-.785, .530] | [-1.067, .671] | [-.793, .646] | [-1.274, .677] |
|  | (.434) | (.551) | (.945) | (.106) | (.716) | (.577) | (.704) | (.655) | (.841) | (.548) |
| Nevada | -.147 | .015 | -.064 | -.006 | -.141 | .089 | -.063 | -.231 | -.328 | -.533 |
|  | .206 | .248 | .210 | .237 | .276 | .254 | .232 | .279 | .257 | .318 |
|  | [-.551, .257] | [-.470, .501] | [-.475, .347] | [-.472, .459] | [-.683, .400] | [-.408, .587] | [-.517, .392] | [-.778, .316] | [-.831, .175] | [-1.156, .091] |
|  | (.475) | (.951) | (.761) | (.979) | (.609) | (.725) | (.787) | (.408) | (.201) | (.094) |
| New Hampshire | -.090 | -.036 | -.299 | .230 | -.140 | .154 | .180 | .085 | .470 | -.755 |
|  | .261 | .287 | .307 | .263 | .300 | .300 | .255 | .356 | .306 | .405 |
|  | [-.603, .423] | [-.598, .527] | [-.901, .302] | [-.286, .747] | [-.728, .448] | [-.434, .741] | [-.321, .681] | [-.613, .783] | [-.131, 1.070] | [-1.550, .039] |
|  | (.731) | (.901) | (.330) | (.382) | (.641) | (.608) | (.480) | (.811) | (.125) | (.062) |
| New Jersey | -.250 | -.078 | -.042 | -.217 | -.050 | .049 | -.092 | -.093 | .021 | -.352 |
|  | .211 | .252 | .175 | .225 | .262 | .248 | .211 | .275 | .225 | .310 |
|  | [-.665, .164] | [-.573, .416] | [-.385, .300] | [-.659, .225] | [-.564, .463] | [-.437, .535] | [-.505, .321] | [-.632, .445] | [-.420, .461] | [-.960, .255] |
|  | (.237) | (.756) | (.808) | (.335) | (.848) | (.843) | (.662) | (.734) | (.926) | (.255) |
| New Mexico | -.212 | .596 | -.044 | .139 | -.028 | .199 | .195 | .138 | -.127 | -.371 |
|  | .260 | .296 | .228 | .254 | .299 | .268 | .238 | .322 | .317 | .358 |
|  | [-.722, .299] | [.016, 1.175] | [-.490, .403] | [-.359, .636] | [-.613, .558] | [-.326, .724] | [-.271, .661] | [-.493, .768] | [-.748, .493] | [-1.073, .331] |
|  | (.417) | (.044) | (.847) | (.585) | (.926) | (.457) | (.411) | (.669) | (.688) | (.300) |
| New York | -.168 | .283 | -.248 | .074 | -.045 | .210 | .136 | .181 | -.013 | -.242 |
|  | .168 | .208 | .162 | .189 | .233 | .205 | .180 | .238 | .205 | .265 |
|  | [-.497, .160] | [-.125, .691] | [-.566, .070] | [-.297, .446] | [-.501, .411] | [-.193, .612] | [-.217, .488] | [-.285, .647] | [-.415, .390] | [-.762, .278] |
|  | (.316) | (.174) | (.126) | (.695) | (.847) | (.307) | (.451) | (.445) | (.951) | (.362) |
| North Carolina | -.097 | .083 | -.242 | -.117 | .193 | .230 | .071 | .068 | -.213 | -.234 |
|  | .186 | .224 | .186 | .217 | .238 | .218 | .196 | .253 | .225 | .290 |
|  | [-.461, .268] | [-.356, .523] | [-.605, .122] | [-.542, .308] | [-.274, .660] | [-.198, .658] | [-.314, .455] | [-.427, .564] | [-.654, .228] | [-.803, .335] |
|  | (.603) | (.711) | (.193) | (.590) | (.417) | (.292) | (.718) | (.787) | (.343) | (.421) |
| North Dakota | .271 | -.106 | -.578 | -.486 | -.601 | -.753 | -.558 | -.144 | -.146 | -1.509 |
|  | .272 | .416 | .373 | .497 | .518 | .358 | .451 | .371 | .400 | .490 |
|  | [-.263, .804] | [-.921, .709] | [-1.310, .154] | [-1.460, .488] | [-1.616, .414] | [-1.456, -.050] | [-1.442, .325] | [-.871, .582] | [-.930, .637] | [-2.470, -.548] |
|  | (.320) | (.798) | (.122) | (.328) | (.246) | (.036) | (.215) | (.697) | (.715) | (.002) |
| Ohio | -.090 | .236 | -.256 | .090 | -.097 | .177 | -.029 | -.272 | -.218 | .064 |
|  | .177 | .215 | .176 | .199 | .241 | .219 | .191 | .247 | .216 | .271 |
|  | [-.437, .256] | [-.186, .657] | [-.601, .089] | [-.300, .479] | [-.570, .376] | [-.253, .607] | [-.403, .345] | [-.756, .212] | [-.642, .206] | [-.468, .596] |
|  | (.609) | (.274) | (.145) | (.652) | (.689) | (.420) | (.879) | (.271) | (.314) | (.813) |
| Oklahoma | -.296 | .031 | -.642 | -.148 | .014 | -.265 | -.245 | .027 | -.240 | -.649 |
|  | .289 | .290 | .283 | .311 | .286 | .312 | .299 | .324 | .325 | .371 |
|  | [-.863, .271] | [-.538, .600] | [-1.196, -.088] | [-.758, .461] | [-.548, .575] | [-.877, .346] | [-.831, .341] | [-.609, .662] | [-.877, .396] | [-1.376, .079] |
|  | (.306) | (.914) | (.023) | (.634) | (.962) | (.395) | (.412) | (.934) | (.459) | (.081) |
| Oregon | -.240 | -.054 | -.322 | .039 | -.851 | -.107 | -.459 | -.619 | .291 | -.421 |
|  | .208 | .236 | .200 | .217 | .276 | .254 | .223 | .249 | .231 | .302 |
|  | [-.648, .169] | [-.516, .409] | [-.714, .071] | [-.386, .464] | [-1.393, -.310] | [-.606, .392] | [-.897, -.022] | [-1.107, -.131] | [-.161, .744] | [-1.013, .171] |
|  | (.250) | (.819) | (.109) | (.858) | (.002) | (.674) | (.040) | (.013) | (.207) | (.163) |
| Pennsylvania | -.145 | .094 | -.165 | .099 | -.005 | .147 | .159 | -.193 | -.119 | -.081 |
|  | .159 | .205 | .160 | .189 | .231 | .206 | .175 | .233 | .203 | .261 |
|  | [-.457, .168] | [-.309, .496] | [-.478, .147] | [-.270, .469] | [-.457, .448] | [-.257, .551] | [-.185, .502] | [-.650, .263] | [-.516, .278] | [-.592, .430] |
|  | (.364) | (.648) | (.300) | (.598) | (.984) | (.476) | (.365) | (.406) | (.557) | (.756) |
| Rhode Island | -.082 | .881 | .024 | -.044 | .125 | .228 | .267 | .495 | -.269 | -.175 |
|  | .550 | .408 | .258 | .512 | .427 | .370 | .316 | .542 | .489 | .566 |
|  | [-1.159, .996] | [.082, 1.681] | [-.482, .530] | [-1.048, .960] | [-.713, .963] | [-.498, .955] | [-.353, .887] | [-.567, 1.558] | [-1.227, .689] | [-1.284, .934] |
|  | (.882) | (.031) | (.926) | (.931) | (.770) | (.538) | (.398) | (.361) | (.582) | (.757) |
| South Carolina | -.156 | -.109 | -.147 | -.012 | -.064 | .217 | .079 | -.377 | -.109 | -.203 |
|  | .232 | .248 | .199 | .235 | .295 | .248 | .244 | .283 | .276 | .321 |
|  | [-.611, .299] | [-.595, .378] | [-.538, .244] | [-.473, .449] | [-.643, .516] | [-.269, .704] | [-.400, .558] | [-.931, .178] | [-.651, .433] | [-.833, .428] |
|  | (.502) | (.661) | (.461) | (.960) | (.830) | (.382) | (.746) | (.183) | (.693) | (.528) |
| South Dakota | -.068 | .301 | -.536 | .138 | -.105 | .151 | -.169 | -.698 | -.205 | -.203 |
|  | .317 | .361 | .374 | .319 | .392 | .435 | .394 | .408 | .367 | .364 |
|  | [-.689, .553] | [-.407, 1.009] | [-1.268, .197] | [-.487, .763] | [-.873, .664] | [-.702, 1.005] | [-.942, .605] | [-1.498, .101] | [-.925, .515] | [-.917, .511] |
|  | (.830) | (.404) | (.152) | (.665) | (.789) | (.728) | (.668) | (.087) | (.577) | (.577) |
| Tennessee | -.173 | -.178 | -.326 | -.269 | -.491 | -.151 | -.127 | .031 | -.019 | -.481 |
|  | .200 | .239 | .198 | .223 | .269 | .239 | .221 | .305 | .246 | .320 |
|  | [-.566, .220] | [-.646, .290] | [-.714, .063] | [-.706, .167] | [-1.019, .036] | [-.620, .318] | [-.561, .306] | [-.567, .629] | [-.502, .463] | [-1.108, .146] |
|  | (.388) | (.455) | (.100) | (.226) | (.068) | (.527) | (.565) | (.919) | (.938) | (.132) |
| Texas | -.129 | .073 | -.342 | -.092 | -.225 | .025 | -.015 | -.065 | -.178 | -.496 |
|  | .166 | .208 | .161 | .190 | .233 | .208 | .177 | .234 | .204 | .267 |
|  | [-.455, .197] | [-.335, .480] | [-.657, -.027] | [-.463, .280] | [-.681, .231] | [-.383, .433] | [-.362, .332] | [-.525, .395] | [-.578, .223] | [-1.019, .027] |
|  | (.438) | (.727) | (.033) | (.629) | (.333) | (.906) | (.932) | (.781) | (.384) | (.063) |
| Utah | -.017 | -.262 | -.493 | -.336 | -.326 | -.081 | -.035 | -.632 | .057 | -1.016 |
|  | .241 | .280 | .274 | .257 | .294 | .267 | .258 | .305 | .302 | .377 |
|  | [-.490, .455] | [-.811, .287] | [-1.030, .044] | [-.840, .167] | [-.902, .251] | [-.605, .443] | [-.542, .472] | [-1.230, -.034] | [-.535, .649] | [-1.756, -.276] |
|  | (.943) | (.350) | (.072) | (.191) | (.268) | (.761) | (.892) | (.038) | (.850) | (.007) |
| Vermont | .319 | -.117 | -.118 | -.098 | -.350 | .214 | -.350 | -.830 | -.441 | -1.379 |
|  | .237 | .348 | .248 | .368 | .365 | .363 | .387 | .349 | .442 | .496 |
|  | [-.146, .784] | [-.800, .565] | [-.604, .368] | [-.821, .624] | [-1.066, .366] | [-.498, .925] | [-1.110, .409] | [-1.515, -.146] | [-1.308, .427] | [-2.351, -.407] |
|  | (.179) | (.736) | (.635) | (.790) | (.337) | (.556) | (.365) | (.017) | (.319) | (.005) |
| Virginia | -.067 | .083 | -.242 | -.112 | -.310 | .102 | -.110 | -.256 | .038 | -.534 |
|  | .177 | .220 | .172 | .211 | .247 | .222 | .195 | .249 | .218 | .288 |
|  | [-.413, .280] | [-.347, .514] | [-.579, .095] | [-.526, .303] | [-.794, .174] | [-.334, .537] | [-.492, .273] | [-.744, .232] | [-.389, .466] | [-1.098, .030] |
|  | (.707) | (.704) | (.160) | (.597) | (.210) | (.647) | (.574) | (.304) | (.861) | (.063) |
| Washington | -.165 | -.207 | -.151 | -.222 | -.763 | -.218 | -.539 | -.370 | -.278 | -.894 |
|  | .198 | .228 | .183 | .214 | .260 | .231 | .201 | .249 | .236 | .292 |
|  | [-.553, .223] | [-.654, .239] | [-.511, .209] | [-.642, .197] | [-1.273, -.253] | [-.672, .235] | [-.932, -.146] | [-.859, .118] | [-.741, .185] | [-1.466, -.321] |
|  | (.404) | (.363) | (.411) | (.299) | (.003) | (.346) | (.007) | (.137) | (.239) | (.002) |
| West Virginia | -.126 | -.013 | -.255 | -.445 | .016 | .194 | .068 | .090 | .038 | -.497 |
|  | .267 | .270 | .252 | .293 | .311 | .306 | .294 | .336 | .279 | .376 |
|  | [-.650, .398] | [-.542, .515] | [-.749, .238] | [-1.019, .128] | [-.593, .625] | [-.406, .794] | [-.508, .645] | [-.570, .749] | [-.509, .585] | [-1.234, .241] |
|  | (.636) | (.961) | (.310) | (.128) | (.959) | (.526) | (.817) | (.790) | (.891) | (.187) |
| Wisconsin | .064 | -.031 | -.347 | -.116 | -.192 | .012 | -.165 | -.193 | -.426 | -.366 |
|  | .186 | .221 | .192 | .224 | .258 | .230 | .211 | .261 | .233 | .290 |
|  | [-.301, .429] | [-.465, .403] | [-.723, .028] | [-.554, .323] | [-.698, .314] | [-.438, .462] | [-.579, .249] | [-.704, .318] | [-.883, .030] | [-.935, .203] |
|  | (.730) | (.888) | (.070) | (.606) | (.457) | (.957) | (.434) | (.459) | (.067) | (.207) |
| Wyoming | -.649 | .982 | .145 | -.355 | .546 | -.472 | .704 | .967 | 1.060 | -.508 |
|  | 1.093 | .455 | .596 | .709 | .307 | 1.259 | .267 | .965 | .472 | .961 |
|  | [-2.792, 1.494] | [.090, 1.875] | [-1.025, 1.314] | [-1.745, 1.034] | [-.057, 1.148] | [-2.941, 1.997] | [.181, 1.227] | [-.925, 2.859] | [.135, 1.985] | [-2.391, 1.375] |
|  | (.553) | (.031) | (.808) | (.616) | (.076) | (.708) | (.008) | (.316) | (.025) | (.597) |
| N | 2967 | 2973 | 2969 | 2970 | 2976 | 2973 | 2972 | 2967 | 2970 | 2975 |
| Pseudo-R^2^ |  |  |  |  |  |  |  |  |  |  |
| Model χ^2^ |  |  |  |  |  |  |  |  |  |  |
| d.f. | 90 | 90 | 90 | 90 | 90 | 90 | 90 | 90 | 90 | 90 |
| p-value | <.001 | <.001 | <.001 | <.001 | <.001 | <.001 | <.001 | <.001 | <.001 | <.001 |

**S12 Fig. Public Policies, by News Source.** Estimates are the predicted effects comparing Republicans and Others (unaffiliated or identifying with a third party) to self-identified Democrats for respondents who report watching right-wing news versus those who do not. Confidence intervals are not adjusted for multiple comparisons in this exploratory analysis.

Results Adjusting for Local Policies

S31 Table. Regression Results, Health Behaviors, Adjusting for Local Policies.

|  | (1) | (2) | (3) | (4) | (5) | (6) | (7) | (8) | (9) |
| --- | --- | --- | --- | --- | --- | --- | --- | --- | --- |
|  | washhands | boughtsanitizer | visitdoctor | changedtravel | avoidcontact | avoidgatherings | soughtinfo | selfquarantine | changehealth |
| Republican | -.079 | -.096 | -.023 | -.127 | -.135 | -.101 | -.189 | -.141 | -.890 |
|  | .038 | .055 | .027 | .053 | .054 | .046 | .055 | .056 | .206 |
|  | [-.154, -.003] | [-.203, .011] | [-.077, .030] | [-.230, -.023] | [-.241, -.029] | [-.191, -.010] | [-.296, -.082] | [-.251, -.030] | [-1.293, -.486] |
|  | (.040) | (.079) | (.394) | (.017) | (.013) | (.029) | (.001) | (.013) | (<.001) |
| Other | -.112 | -.034 | -.010 | -.098 | -.073 | -.083 | -.047 | -.047 | -.503 |
|  | .036 | .052 | .025 | .049 | .048 | .042 | .052 | .052 | .196 |
|  | [-.182, -.041] | [-.137, .068] | [-.059, .039] | [-.193, -.002] | [-.168, .022] | [-.164, -.001] | [-.149, .054] | [-.149, .055] | [-.888, -.119] |
|  | (.002) | (.514) | (.696) | (.045) | (.130) | (.047) | (.360) | (.368) | (.010) |
| policies | -.022 | -.036 | -.051 | -.211 | -.054 | -.125 | -.008 | -.007 | -.514 |
|  | .061 | .105 | .054 | .100 | .093 | .074 | .102 | .106 | .361 |
|  | [-.141, .097] | [-.242, .171] | [-.156, .055] | [-.408, -.014] | [-.237, .128] | [-.271, .020] | [-.208, .191] | [-.216, .201] | [-1.223, .194] |
|  | (.722) | (.735) | (.346) | (.035) | (.560) | (.091) | (.934) | (.945) | (.155) |
| Republican # policies | -.009 | .024 | .137 | .253 | -.076 | -.060 | .033 | .121 | .423 |
|  | .110 | .154 | .081 | .151 | .153 | .129 | .156 | .161 | .589 |
|  | [-.224, .206] | [-.277, .326] | [-.021, .296] | [-.043, .549] | [-.376, .223] | [-.313, .193] | [-.273, .338] | [-.194, .436] | [-.732,1.578] |
|  | (.933) | (.873) | (.089) | (.094) | (.618) | (.641) | (.833) | (.451) | (.473) |
| Other # policies | .116 | -.051 | -.012 | .117 | -.068 | .038 | -.205 | -.087 | -.154 |
|  | .099 | .146 | .069 | .136 | .137 | .120 | .147 | .145 | .552 |
|  | [-.078, .309] | [-.338, .235] | [-.147, .122] | [-.150, .383] | [-.338, .201] | [-.199, .274] | [-.493, .084] | [-.371, .197] | [-1.236, .929] |
|  | (.241) | (.726) | (.858) | (.391) | (.619) | (.755) | (.164) | (.547) | (.781) |
| 35- | .039 | .049 | .003 | -.034 | .044 | .013 | -.012 | -.020 | .083 |
|  | .023 | .033 | .016 | .032 | .032 | .029 | .033 | .034 | .129 |
|  | [-.007, .085] | [-.016, .115] | [-.029, .036] | [-.096, .028] | [-.019, .107] | [-.044, .070] | [-.077, .054] | [-.086, .046] | [-.169, .336] |
|  | (.095) | (.138) | (.842) | (.284) | (.167) | (.658) | (.728) | (.556) | (.517) |
| 50- | .032 | .045 | -.017 | -.051 | .038 | .073 | -.055 | -.100 | -.033 |
|  | .022 | .031 | .015 | .030 | .030 | .027 | .031 | .031 | .119 |
|  | [-.012, .076] | [-.016, .107] | [-.046, .013] | [-.109, .008] | [-.021, .097] | [.021, .126] | [-.116, .007] | [-.161, -.039] | [-.266, .200] |
|  | (.157) | (.146) | (.265) | (.088) | (.206) | (.006) | (.080) | (.001) | (.782) |
| 70- | .089 | .082 | -.028 | -.022 | .154 | .165 | -.059 | .061 | .442 |
|  | .026 | .040 | .017 | .039 | .037 | .030 | .039 | .040 | .141 |
|  | [.037, .140] | [.004, .161] | [-.062, .006] | [-.097, .054] | [.082, .225] | [.106, .224] | [-.137, .018] | [-.018, .140] | [.165, .718] |
|  | (.001) | (.040) | (.108) | (.574) | (<.001) | (<.001) | (.132) | (.132) | (.002) |
| gender | .039 | .015 | .004 | -.011 | .009 | .037 | .032 | .054 | .178 |
|  | .016 | .023 | .011 | .022 | .022 | .019 | .023 | .023 | .086 |
|  | [.008, .070] | [-.031, .060] | [-.017, .025] | [-.054, .031] | [-.034, .052] | [-.001, .074] | [-.013, .077] | [.009, .099] | [.009, .346] |
|  | (.013) | (.529) | (.692) | (.601) | (.687) | (.055) | (.167) | (.020) | (.039) |
| white | -.006 | -.091 | -.024 | -.024 | .036 | .049 | .064 | -.009 | -.005 |
|  | .019 | .027 | .013 | .026 | .026 | .024 | .027 | .027 | .107 |
|  | [-.043, .031] | [-.144, -.037] | [-.050, .002] | [-.075, .026] | [-.015, .087] | [.002, .096] | [.011, .117] | [-.062, .044] | [-.215, .205] |
|  | (.745) | (.001) | (.076) | (.347) | (.167) | (.039) | (.018) | (.737) | (.962) |
| married | -.010 | .040 | .010 | .049 | .008 | -.018 | -.002 | .008 | .084 |
|  | .017 | .025 | .012 | .024 | .024 | .021 | .025 | .025 | .092 |
|  | [-.044, .024] | [-.010, .089] | [-.013, .034] | [.002, .095] | [-.039, .054] | [-.059, .022] | [-.052, .047] | [-.041, .057] | [-.098, .265] |
|  | (.557) | (.114) | (.392) | (.040) | (.742) | (.377) | (.922) | (.749) | (.366) |
| High school graduate | -.071 | -.030 | -.008 | .106 | -.154 | -.062 | -.036 | .017 | -.238 |
|  | .051 | .069 | .036 | .049 | .064 | .061 | .068 | .071 | .252 |
|  | [-.172, .030] | [-.164, .105] | [-.078, .063] | [.010, .201] | [-.280, -.029] | [-.182, .058] | [-.170, .097] | [-.124, .157] | [-.732, .256] |
|  | (.169) | (.667) | (.831) | (.030) | (.016) | (.310) | (.595) | (.817) | (.344) |
| Some college | -.021 | -.056 | -.025 | .209 | -.069 | .009 | .124 | .025 | .198 |
|  | .052 | .070 | .036 | .051 | .065 | .062 | .069 | .073 | .257 |
|  | [-.123, .082] | [-.193, .081] | [-.096, .046] | [.108, .310] | [-.197, .059] | [-.112, .131] | [-.011, .260] | [-.118, .168] | [-.305, .701] |
|  | (.692) | (.425) | (.488) | (<.001) | (.291) | (.879) | (.073) | (.732) | (.441) |
| 2-year | -.008 | -.006 | -.011 | .164 | -.106 | .001 | .126 | -.017 | .144 |
|  | .054 | .074 | .038 | .056 | .069 | .065 | .074 | .077 | .273 |
|  | [-.114, .098] | [-.152, .140] | [-.086, .063] | [.056, .273] | [-.242, .030] | [-.127, .130] | [-.019, .271] | [-.167, .134] | [-.393, .680] |
|  | (.884) | (.934) | (.764) | (.003) | (.127) | (.983) | (.088) | (.827) | (.599) |
| 4-year | .019 | -.068 | .013 | .243 | -.068 | .021 | .142 | .023 | .327 |
|  | .052 | .071 | .037 | .053 | .066 | .062 | .070 | .074 | .259 |
|  | [-.083, .122] | [-.207, .072] | [-.060, .086] | [.139, .347] | [-.197, .062] | [-.101, .143] | [.004, .280] | [-.122, .168] | [-.182, .835] |
|  | (.715) | (.344) | (.723) | (<.001) | (.306) | (.733) | (.044) | (.751) | (.208) |
| Post-grad | -.016 | -.070 | .008 | .369 | -.016 | .059 | .199 | .060 | .593 |
|  | .054 | .074 | .040 | .057 | .068 | .063 | .073 | .077 | .269 |
|  | [-.123, .090] | [-.216, .075] | [-.071, .086] | [.257, .482] | [-.149, .117] | [-.065, .184] | [.055, .343] | [-.090, .211] | [.067,1.120] |
|  | (.765) | (.344) | (.845) | (<.001) | (.809) | (.351) | (.007) | (.431) | (.027) |
| income=2 | -.034 | -.120 | -.084 | -.095 | -.090 | -.083 | .076 | -.009 | -.439 |
|  | .055 | .068 | .037 | .056 | .068 | .065 | .069 | .071 | .279 |
|  | [-.141, .073] | [-.253, .013] | [-.156, -.012] | [-.206, .016] | [-.223, .044] | [-.212, .045] | [-.060, .212] | [-.148, .130] | [-.987, .108] |
|  | (.534) | (.076) | (.022) | (.093) | (.187) | (.202) | (.272) | (.894) | (.116) |
| income=3 | .008 | -.061 | -.049 | -.036 | -.044 | .004 | .117 | -.093 | -.154 |
|  | .053 | .068 | .039 | .059 | .066 | .062 | .066 | .067 | .271 |
|  | [-.096, .111] | [-.195, .074] | [-.126, .028] | [-.151, .080] | [-.173, .085] | [-.118, .125] | [-.013, .247] | [-.225, .039] | [-.686, .378] |
|  | (.885) | (.377) | (.211) | (.546) | (.502) | (.952) | (.079) | (.169) | (.571) |
| income=4 | .013 | .036 | -.046 | .028 | -.019 | .045 | .203 | -.089 | .171 |
|  | .052 | .068 | .040 | .060 | .066 | .062 | .067 | .067 | .269 |
|  | [-.088, .115] | [-.097, .169] | [-.124, .032] | [-.091, .146] | [-.149, .111] | [-.077, .167] | [.071, .334] | [-.220, .043] | [-.356, .698] |
|  | (.797) | (.598) | (.249) | (.647) | (.778) | (.471) | (.003) | (.186) | (.524) |
| income=5 | .027 | -.035 | -.032 | -.118 | -.022 | .057 | .189 | -.082 | -.016 |
|  | .052 | .069 | .041 | .058 | .068 | .062 | .068 | .068 | .282 |
|  | [-.076, .130] | [-.170, .100] | [-.113, .049] | [-.233, -.004] | [-.154, .110] | [-.063, .178] | [.057, .322] | [-.215, .051] | [-.568, .537] |
|  | (.609) | (.611) | (.439) | (.042) | (.744) | (.353) | (.005) | (.227) | (.955) |
| income=6 | .047 | -.040 | -.049 | -.034 | .003 | .041 | .131 | -.051 | .049 |
|  | .051 | .069 | .039 | .060 | .066 | .061 | .067 | .068 | .267 |
|  | [-.053, .148] | [-.174, .095] | [-.126, .028] | [-.151, .083] | [-.126, .132] | [-.078, .161] | [-.001, .262] | [-.184, .081] | [-.475, .573] |
|  | (.356) | (.563) | (.213) | (.574) | (.963) | (.497) | (.051) | (.449) | (.855) |
| income=7 | .047 | .050 | -.008 | .139 | -.035 | .078 | .186 | -.117 | .340 |
|  | .055 | .076 | .045 | .070 | .072 | .064 | .073 | .072 | .296 |
|  | [-.060, .154] | [-.098, .198] | [-.095, .080] | [.002, .275] | [-.177, .107] | [-.049, .204] | [.044, .329] | [-.258, .024] | [-.240, .920] |
|  | (.389) | (.510) | (.867) | (.046) | (.627) | (.229) | (.011) | (.104) | (.250) |
| income=8 | .017 | -.076 | -.077 | .037 | -.049 | .030 | .056 | .018 | -.044 |
|  | .056 | .071 | .038 | .066 | .070 | .064 | .071 | .071 | .282 |
|  | [-.092, .126] | [-.214, .063] | [-.152, -.003] | [-.092, .166] | [-.187, .088] | [-.095, .156] | [-.083, .196] | [-.122, .158] | [-.597, .509] |
|  | (.758) | (.283) | (.042) | (.577) | (.480) | (.635) | (.429) | (.800) | (.876) |
| income=9 | .075 | .008 | -.058 | .008 | -.014 | .056 | .182 | -.092 | .164 |
|  | .051 | .070 | .040 | .064 | .069 | .062 | .069 | .069 | .274 |
|  | [-.025, .176] | [-.131, .146] | [-.137, .020] | [-.117, .134] | [-.150, .121] | [-.065, .177] | [.046, .317] | [-.228, .044] | [-.374, .701] |
|  | (.142) | (.915) | (.144) | (.896) | (.834) | (.365) | (.009) | (.184) | (.551) |
| income=10 | .030 | -.047 | -.080 | .066 | .025 | .048 | .105 | -.063 | .083 |
|  | .054 | .073 | .039 | .067 | .069 | .064 | .072 | .071 | .285 |
|  | [-.076, .136] | [-.190, .096] | [-.157, -.003] | [-.065, .197] | [-.110, .160] | [-.078, .173] | [-.037, .247] | [-.203, .077] | [-.475, .642] |
|  | (.583) | (.519) | (.042) | (.323) | (.718) | (.458) | (.148) | (.378) | (.770) |
| income=11 | .045 | .025 | -.061 | .093 | -.002 | .060 | .114 | -.045 | .229 |
|  | .052 | .073 | .040 | .067 | .069 | .062 | .071 | .071 | .284 |
|  | [-.058, .148] | [-.119, .169] | [-.140, .018] | [-.038, .224] | [-.136, .132] | [-.062, .182] | [-.026, .253] | [-.185, .095] | [-.328, .786] |
|  | (.391) | (.732) | (.128) | (.165) | (.976) | (.336) | (.110) | (.531) | (.421) |
| income=12 | .074 | .031 | -.050 | .076 | .034 | .057 | .199 | -.074 | .346 |
|  | .054 | .079 | .043 | .074 | .073 | .067 | .076 | .077 | .308 |
|  | [-.032, .180] | [-.123, .185] | [-.135, .035] | [-.068, .221] | [-.109, .177] | [-.075, .188] | [.049, .348] | [-.226, .077] | [-.259, .951] |
|  | (.173) | (.695) | (.251) | (.301) | (.642) | (.397) | (.009) | (.335) | (.262) |
| income=13 | .051 | -.075 | -.108 | .146 | .042 | .097 | .363 | .003 | .518 |
|  | .075 | .113 | .036 | .102 | .098 | .084 | .093 | .114 | .440 |
|  | [-.097, .199] | [-.296, .146] | [-.179, -.037] | [-.053, .345] | [-.151, .235] | [-.067, .262] | [.180, .545] | [-.220, .225] | [-.344,1.380] |
|  | (.501) | (.506) | (.003) | (.150) | (.672) | (.246) | (<.001) | (.981) | (.239) |
| income=14 | .005 | .116 | -.017 | .230 | .072 | .079 | .143 | .024 | .651 |
|  | .080 | .111 | .071 | .107 | .095 | .085 | .104 | .113 | .406 |
|  | [-.153, .162] | [-.101, .333] | [-.156, .122] | [.019, .440] | [-.115, .259] | [-.087, .245] | [-.060, .346] | [-.197, .246] | [-.145,1.447] |
|  | (.953) | (.296) | (.810) | (.032) | (.452) | (.353) | (.168) | (.829) | (.109) |
| income=15 | .145 | .164 | .190 | .243 | .311 | .202 | .535 | .303 | 2.094 |
|  | .050 | .273 | .274 | .324 | .098 | .083 | .113 | .321 | 1.120 |
|  | [.046, .244] | [-.371, .700] | [-.348, .728] | [-.392, .879] | [.119, .504] | [.040, .365] | [.314, .756] | [-.327, .934] | [-.101,4.290] |
|  | (.004) | (.548) | (.488) | (.453) | (.002) | (.015) | (<.001) | (.346) | (.062) |
| income=16 | .149 | .346 | -.008 | .124 | .093 | .087 | .198 | -.082 | .907 |
|  | .057 | .145 | .092 | .125 | .115 | .103 | .159 | .148 | .440 |
|  | [.038, .260] | [.062, .629] | [-.188, .173] | [-.120, .369] | [-.132, .319] | [-.114, .288] | [-.115, .510] | [-.372, .208] | [.044,1.769] |
|  | (.009) | (.017) | (.935) | (.319) | (.418) | (.397) | (.215) | (.579) | (.039) |
| income=17 | .013 | -.016 | -.090 | .014 | -.047 | .052 | .088 | -.080 | -.068 |
|  | .049 | .063 | .036 | .055 | .061 | .057 | .062 | .062 | .255 |
|  | [-.083, .109] | [-.140, .107] | [-.161, -.020] | [-.094, .121] | [-.167, .073] | [-.060, .164] | [-.033, .209] | [-.201, .041] | [-.567, .432] |
|  | (.792) | (.797) | (.012) | (.802) | (.440) | (.362) | (.154) | (.194) | (.791) |
| Rural Code=2 | -.003 | -.029 | -.006 | .027 | .027 | .028 | -.037 | -.007 | .000 |
|  | .022 | .031 | .012 | .029 | .029 | .026 | .030 | .031 | .116 |
|  | [-.045, .039] | [-.089, .032] | [-.030, .019] | [-.031, .084] | [-.030, .083] | [-.022, .078] | [-.096, .023] | [-.067, .053] | [-.228, .228] |
|  | (.888) | (.350) | (.651) | (.362) | (.353) | (.276) | (.229) | (.815) | (.998) |
| Rural Code=3 | -.022 | -.003 | -.016 | -.014 | -.012 | -.021 | -.035 | -.024 | -.147 |
|  | .041 | .054 | .022 | .051 | .053 | .047 | .053 | .053 | .208 |
|  | [-.102, .059] | [-.110, .104] | [-.060, .028] | [-.115, .086] | [-.117, .093] | [-.114, .072] | [-.138, .069] | [-.127, .080] | [-.555, .262] |
|  | (.598) | (.955) | (.485) | (.779) | (.820) | (.652) | (.511) | (.652) | (.481) |
| Rural Code=4 | -.103 | .115 | .058 | .078 | -.030 | .239 | .184 | -.003 | .539 |
|  | .111 | .125 | .082 | .124 | .123 | .038 | .114 | .123 | .452 |
|  | [-.320, .115] | [-.131, .360] | [-.102, .219] | [-.166, .322] | [-.272, .212] | [.166, .313] | [-.039, .407] | [-.244, .238] | [-.347,1.425] |
|  | (.355) | (.360) | (.476) | (.531) | (.809) | (<.001) | (.106) | (.982) | (.233) |
| Rural Code=6 | -.233 | .305 | -.029 | -.187 | .018 | -.123 | .771 | -.325 | .197 |
|  | .351 | .367 | .034 | .109 | .358 | .326 | .077 | .124 | 1.221 |
|  | [-.922, .455] | [-.414,1.024] | [-.095, .038] | [-.402, .028] | [-.684, .719] | [-.763, .517] | [.620, .922] | [-.567, -.082] | [-2.197,2.591] |
|  | (.506) | (.406) | (.396) | (.088) | (.960) | (.706) | (<.001) | (.009) | (.872) |
| Arizona | .157 | -.193 | -.099 | -.007 | -.018 | -.046 | -.110 | .003 | -.315 |
|  | .096 | .114 | .075 | .100 | .114 | .099 | .119 | .117 | .441 |
|  | [-.032, .345] | [-.417, .031] | [-.247, .049] | [-.202, .188] | [-.243, .206] | [-.240, .148] | [-.345, .124] | [-.227, .232] | [-1.180, .551] |
|  | (.104) | (.092) | (.189) | (.945) | (.872) | (.641) | (.356) | (.982) | (.476) |
| Arkansas | .124 | -.010 | -.079 | .057 | -.114 | -.215 | -.154 | .067 | -.325 |
|  | .123 | .160 | .098 | .143 | .155 | .146 | .155 | .162 | .645 |
|  | [-.118, .366] | [-.323, .303] | [-.272, .114] | [-.223, .338] | [-.419, .190] | [-.503, .072] | [-.459, .150] | [-.251, .384] | [-1.590, .940] |
|  | (.314) | (.950) | (.420) | (.688) | (.462) | (.142) | (.320) | (.680) | (.614) |
| California | .153 | .007 | -.079 | .068 | .007 | .062 | -.049 | .123 | .293 |
|  | .090 | .102 | .072 | .084 | .097 | .083 | .104 | .104 | .386 |
|  | [-.022, .329] | [-.192, .207] | [-.220, .063] | [-.096, .232] | [-.183, .198] | [-.100, .224] | [-.253, .155] | [-.081, .327] | [-.465,1.051] |
|  | (.087) | (.944) | (.274) | (.417) | (.942) | (.449) | (.638) | (.237) | (.449) |
| Colorado | .236 | .022 | -.091 | .178 | .111 | .145 | -.030 | .064 | .635 |
|  | .098 | .130 | .075 | .118 | .121 | .106 | .131 | .134 | .503 |
|  | [.043, .429] | [-.234, .277] | [-.238, .057] | [-.052, .409] | [-.126, .348] | [-.062, .353] | [-.288, .227] | [-.199, .326] | [-.352,1.621] |
|  | (.017) | (.867) | (.228) | (.130) | (.359) | (.169) | (.817) | (.634) | (.207) |
| Connecticut | .055 | .012 | -.136 | .042 | .139 | .064 | -.065 | .147 | .259 |
|  | .117 | .132 | .071 | .113 | .119 | .106 | .130 | .138 | .473 |
|  | [-.173, .284] | [-.247, .270] | [-.275, .003] | [-.178, .263] | [-.095, .373] | [-.145, .272] | [-.320, .191] | [-.124, .418] | [-.668,1.186] |
|  | (.635) | (.930) | (.056) | (.706) | (.243) | (.550) | (.620) | (.288) | (.584) |
| Delaware | .254 | -.043 | -.128 | .070 | .332 | -.124 | -.123 | .276 | .513 |
|  | .095 | .225 | .075 | .179 | .103 | .230 | .200 | .245 | .676 |
|  | [.067, .440] | [-.484, .397] | [-.275, .018] | [-.281, .422] | [.129, .535] | [-.576, .328] | [-.515, .268] | [-.204, .756] | [-.813,1.839] |
|  | (.008) | (.847) | (.087) | (.694) | (.001) | (.591) | (.537) | (.260) | (.448) |
| Florida | .139 | .068 | -.077 | -.035 | .045 | -.009 | -.106 | .075 | .100 |
|  | .092 | .104 | .073 | .084 | .100 | .087 | .107 | .107 | .397 |
|  | [-.041, .319] | [-.137, .272] | [-.220, .066] | [-.201, .131] | [-.151, .241] | [-.179, .162] | [-.317, .104] | [-.134, .284] | [-.679, .879] |
|  | (.130) | (.516) | (.292) | (.678) | (.651) | (.920) | (.321) | (.481) | (.801) |
| Georgia | .151 | -.038 | -.112 | .162 | .118 | .132 | -.127 | .111 | .398 |
|  | .097 | .117 | .074 | .098 | .109 | .092 | .120 | .120 | .427 |
|  | [-.040, .342] | [-.268, .192] | [-.258, .034] | [-.030, .354] | [-.097, .332] | [-.048, .313] | [-.362, .108] | [-.123, .346] | [-.440,1.236] |
|  | (.122) | (.748) | (.131) | (.099) | (.281) | (.150) | (.290) | (.352) | (.352) |
| Idaho | -.084 | -.081 | -.141 | .002 | -.150 | -.162 | -.423 | -.051 | -1.089 |
|  | .164 | .173 | .074 | .147 | .177 | .164 | .168 | .158 | .836 |
|  | [-.405, .238] | [-.420, .257] | [-.285, .004] | [-.285, .290] | [-.497, .196] | [-.483, .159] | [-.752, -.093] | [-.361, .259] | [-2.729, .550] |
|  | (.611) | (.637) | (.057) | (.987) | (.394) | (.321) | (.012) | (.747) | (.193) |
| Illinois | .130 | -.107 | -.102 | .102 | -.043 | -.021 | -.083 | .105 | -.020 |
|  | .095 | .108 | .073 | .092 | .106 | .093 | .112 | .111 | .421 |
|  | [-.057, .317] | [-.320, .105] | [-.245, .040] | [-.079, .282] | [-.251, .165] | [-.202, .161] | [-.303, .137] | [-.113, .322] | [-.846, .807] |
|  | (.172) | (.322) | (.159) | (.269) | (.683) | (.824) | (.459) | (.345) | (.963) |
| Indiana | .145 | -.107 | -.095 | .118 | .055 | .131 | -.132 | .112 | .227 |
|  | .110 | .130 | .077 | .115 | .130 | .107 | .131 | .133 | .498 |
|  | [-.071, .361] | [-.362, .149] | [-.246, .057] | [-.107, .342] | [-.199, .309] | [-.079, .341] | [-.390, .125] | [-.149, .372] | [-.750,1.203] |
|  | (.189) | (.413) | (.219) | (.304) | (.670) | (.220) | (.313) | (.399) | (.649) |
| Iowa | .304 | -.021 | -.132 | -.225 | -.176 | -.016 | -.061 | -.226 | -.554 |
|  | .092 | .173 | .071 | .129 | .177 | .160 | .173 | .124 | .501 |
|  | [.123, .485] | [-.361, .319] | [-.272, .008] | [-.478, .027] | [-.523, .171] | [-.329, .297] | [-.401, .279] | [-.469, .016] | [-1.537, .428] |
|  | (.001) | (.902) | (.064) | (.080) | (.320) | (.920) | (.725) | (.067) | (.269) |
| Kansas | .303 | -.031 | -.143 | .252 | .380 | .290 | .179 | .271 | 1.500 |
|  | .092 | .254 | .072 | .195 | .109 | .086 | .254 | .252 | .815 |
|  | [.122, .484] | [-.529, .467] | [-.284, -.002] | [-.131, .634] | [.166, .593] | [.121, .459] | [-.319, .677] | [-.224, .765] | [-.099,3.100] |
|  | (.001) | (.903) | (.048) | (.197) | (<.001) | (.001) | (.481) | (.283) | (.066) |
| Kentucky | -.091 | -.306 | -.153 | -.261 | -.382 | -.721 | -.100 | -.048 | -2.061 |
|  | .219 | .149 | .073 | .102 | .232 | .092 | .193 | .178 | .495 |
|  | [-.521, .339] | [-.597, -.014] | [-.296, -.010] | [-.462, -.060] | [-.838, .074] | [-.901, -.540] | [-.479, .279] | [-.397, .302] | [-3.032, -1.091] |
|  | (.678) | (.040) | (.036) | (.011) | (.101) | (<.001) | (.605) | (.789) | (<.001) |
| Louisiana | .214 | -.144 | -.100 | -.101 | .153 | .217 | .032 | .059 | .330 |
|  | .105 | .133 | .079 | .100 | .132 | .109 | .144 | .143 | .454 |
|  | [.009, .420] | [-.405, .117] | [-.255, .056] | [-.298, .096] | [-.106, .411] | [.004, .430] | [-.250, .314] | [-.223, .340] | [-.561,1.220] |
|  | (.041) | (.279) | (.210) | (.314) | (.246) | (.046) | (.826) | (.682) | (.468) |
| Maine | .350 | -.210 | -.120 | -.028 | .382 | .243 | .490 | .452 | 1.559 |
|  | .099 | .239 | .074 | .246 | .102 | .093 | .115 | .205 | .631 |
|  | [.156, .543] | [-.680, .260] | [-.265, .026] | [-.511, .454] | [.183, .581] | [.061, .424] | [.265, .715] | [.051, .854] | [.321,2.796] |
|  | (<.001) | (.381) | (.107) | (.908) | (<.001) | (.009) | (<.001) | (.027) | (.014) |
| Maryland | .193 | -.061 | -.131 | .061 | .027 | .129 | -.040 | -.169 | .010 |
|  | .099 | .133 | .070 | .115 | .125 | .107 | .130 | .127 | .486 |
|  | [-.002, .388] | [-.321, .199] | [-.269, .007] | [-.165, .287] | [-.217, .272] | [-.082, .339] | [-.295, .216] | [-.418, .080] | [-.944, .963] |
|  | (.052) | (.646) | (.063) | (.597) | (.827) | (.230) | (.761) | (.183) | (.984) |
| Massachusetts | .143 | -.058 | -.120 | .123 | .106 | .144 | -.200 | .108 | .244 |
|  | .101 | .118 | .071 | .102 | .110 | .092 | .119 | .120 | .438 |
|  | [-.056, .341] | [-.289, .173] | [-.259, .018] | [-.077, .323] | [-.110, .321] | [-.036, .325] | [-.433, .033] | [-.129, .344] | [-.614,1.103] |
|  | (.159) | (.622) | (.089) | (.230) | (.335) | (.116) | (.092) | (.372) | (.577) |
| Michigan | .175 | -.043 | -.062 | -.064 | .062 | .026 | -.104 | -.018 | -.029 |
|  | .094 | .109 | .076 | .088 | .104 | .090 | .110 | .110 | .410 |
|  | [-.009, .359] | [-.258, .171] | [-.211, .087] | [-.237, .109] | [-.141, .265] | [-.151, .203] | [-.320, .112] | [-.234, .198] | [-.833, .775] |
|  | (.062) | (.691) | (.415) | (.468) | (.551) | (.774) | (.345) | (.868) | (.944) |
| Minnesota | .251 | -.064 | -.110 | .045 | .084 | -.000 | -.020 | .085 | .271 |
|  | .092 | .126 | .076 | .111 | .120 | .109 | .129 | .129 | .463 |
|  | [.071, .431] | [-.311, .183] | [-.259, .038] | [-.173, .263] | [-.151, .319] | [-.215, .214] | [-.272, .233] | [-.168, .337] | [-.638,1.180] |
|  | (.006) | (.613) | (.146) | (.687) | (.483) | (.999) | (.879) | (.511) | (.559) |
| Mississippi | .219 | -.027 | -.013 | -.109 | .470 | .096 | .236 | .376 | 1.249 |
|  | .151 | .205 | .148 | .139 | .103 | .184 | .170 | .203 | .637 |
|  | [-.077, .516] | [-.428, .374] | [-.303, .278] | [-.382, .164] | [.269, .672] | [-.266, .458] | [-.098, .570] | [-.023, .774] | [-.001,2.499] |
|  | (.147) | (.895) | (.932) | (.435) | (<.001) | (.602) | (.167) | (.065) | (.050) |
| Missouri | .107 | -.152 | -.077 | .006 | -.180 | .070 | -.113 | -.070 | -.407 |
|  | .107 | .123 | .081 | .113 | .123 | .100 | .127 | .126 | .435 |
|  | [-.102, .317] | [-.393, .089] | [-.235, .082] | [-.214, .227] | [-.420, .061] | [-.127, .267] | [-.362, .136] | [-.316, .177] | [-1.260, .446] |
|  | (.315) | (.217) | (.344) | (.955) | (.143) | (.485) | (.375) | (.578) | (.349) |
| Montana | .342 | .102 | -.075 | .243 | .434 | .288 | .482 | .197 | 2.012 |
|  | .107 | .403 | .072 | .342 | .105 | .090 | .107 | .336 | .610 |
|  | [.132, .551] | [-.688, .892] | [-.216, .066] | [-.428, .915] | [.229, .640] | [.111, .465] | [.271, .692] | [-.463, .856] | [.816,3.208] |
|  | (.001) | (.800) | (.294) | (.478) | (<.001) | (.001) | (<.001) | (.559) | (.001) |
| Nebraska | .125 | -.000 | -.148 | -.303 | -.113 | -.022 | -.047 | -.046 | -.554 |
|  | .154 | .202 | .073 | .101 | .217 | .190 | .212 | .186 | .830 |
|  | [-.176, .426] | [-.395, .395] | [-.291, -.005] | [-.500, -.106] | [-.539, .313] | [-.395, .350] | [-.463, .369] | [-.411, .319] | [-2.182,1.073] |
|  | (.415) | (.999) | (.042) | (.003) | (.602) | (.906) | (.826) | (.805) | (.504) |
| Nevada | .106 | -.188 | -.078 | -.061 | .149 | .079 | -.055 | .123 | .075 |
|  | .108 | .119 | .078 | .099 | .116 | .101 | .126 | .127 | .478 |
|  | [-.105, .317] | [-.422, .045] | [-.230, .075] | [-.255, .133] | [-.080, .377] | [-.119, .277] | [-.302, .193] | [-.127, .373] | [-.862,1.012] |
|  | (.323) | (.113) | (.319) | (.537) | (.202) | (.435) | (.665) | (.335) | (.875) |
| New Hampshire | .253 | -.151 | -.074 | -.095 | .042 | .024 | -.029 | -.145 | -.175 |
|  | .104 | .147 | .090 | .128 | .140 | .125 | .154 | .138 | .484 |
|  | [.049, .456] | [-.439, .137] | [-.251, .103] | [-.346, .157] | [-.233, .316] | [-.221, .269] | [-.331, .273] | [-.417, .127] | [-1.125, .774] |
|  | (.015) | (.305) | (.411) | (.460) | (.766) | (.846) | (.850) | (.295) | (.717) |
| New Jersey | .196 | .053 | -.093 | .075 | .065 | .093 | -.060 | -.011 | .317 |
|  | .094 | .115 | .076 | .095 | .108 | .092 | .114 | .114 | .422 |
|  | [.012, .381] | [-.173, .279] | [-.242, .055] | [-.112, .261] | [-.148, .277] | [-.088, .274] | [-.284, .163] | [-.234, .212] | [-.510,1.144] |
|  | (.037) | (.646) | (.219) | (.433) | (.550) | (.316) | (.597) | (.925) | (.452) |
| New Mexico | .183 | -.119 | -.128 | .108 | .134 | -.126 | -.013 | .028 | .068 |
|  | .120 | .149 | .069 | .137 | .149 | .144 | .151 | .156 | .581 |
|  | [-.053, .419] | [-.411, .174] | [-.264, .007] | [-.161, .378] | [-.159, .427] | [-.409, .157] | [-.309, .283] | [-.277, .334] | [-1.071,1.207] |
|  | (.128) | (.425) | (.064) | (.431) | (.369) | (.383) | (.930) | (.855) | (.907) |
| New York | .078 | .079 | -.048 | .061 | .003 | .073 | -.134 | -.000 | .113 |
|  | .094 | .107 | .075 | .090 | .103 | .087 | .110 | .109 | .411 |
|  | [-.106, .263] | [-.131, .289] | [-.196, .099] | [-.115, .237] | [-.199, .205] | [-.098, .244] | [-.349, .082] | [-.214, .214] | [-.692, .918] |
|  | (.403) | (.461) | (.522) | (.494) | (.974) | (.401) | (.223) | (.999) | (.783) |
| North Carolina | .150 | .073 | -.053 | .083 | .073 | .040 | -.100 | -.028 | .240 |
|  | .102 | .119 | .081 | .102 | .113 | .099 | .121 | .120 | .468 |
|  | [-.049, .350] | [-.159, .306] | [-.211, .106] | [-.117, .283] | [-.148, .294] | [-.154, .234] | [-.337, .137] | [-.263, .208] | [-.677,1.158] |
|  | (.139) | (.536) | (.515) | (.414) | (.516) | (.685) | (.408) | (.819) | (.608) |
| North Dakota | .268 | .430 | -.109 | -.130 | -.021 | .259 | .025 | .134 | .855 |
|  | .099 | .200 | .074 | .171 | .201 | .101 | .194 | .204 | .606 |
|  | [.074, .463] | [.038, .821] | [-.254, .036] | [-.465, .205] | [-.415, .373] | [.060, .457] | [-.356, .406] | [-.267, .535] | [-.333,2.044] |
|  | (.007) | (.031) | (.140) | (.446) | (.918) | (.011) | (.898) | (.513) | (.158) |
| Ohio | .163 | -.037 | -.092 | .086 | .046 | .051 | -.037 | .094 | .274 |
|  | .096 | .112 | .074 | .096 | .106 | .091 | .115 | .114 | .433 |
|  | [-.026, .351] | [-.257, .182] | [-.237, .054] | [-.103, .274] | [-.162, .255] | [-.128, .230] | [-.262, .189] | [-.130, .318] | [-.575,1.123] |
|  | (.091) | (.739) | (.215) | (.375) | (.663) | (.577) | (.749) | (.409) | (.527) |
| Oklahoma | .080 | -.073 | -.049 | -.069 | .041 | .069 | -.123 | .121 | -.004 |
|  | .163 | .190 | .117 | .141 | .178 | .157 | .167 | .180 | .656 |
|  | [-.240, .399] | [-.445, .300] | [-.279, .180] | [-.346, .208] | [-.309, .391] | [-.239, .377] | [-.450, .204] | [-.231, .473] | [-1.292,1.283] |
|  | (.625) | (.702) | (.674) | (.625) | (.820) | (.662) | (.461) | (.501) | (.995) |
| Oregon | .096 | -.146 | -.141 | .042 | -.021 | .040 | -.028 | .096 | -.061 |
|  | .106 | .115 | .071 | .098 | .112 | .099 | .123 | .123 | .457 |
|  | [-.111, .303] | [-.372, .081] | [-.279, -.002] | [-.151, .235] | [-.240, .199] | [-.153, .234] | [-.270, .213] | [-.145, .336] | [-.957, .835] |
|  | (.362) | (.207) | (.046) | (.670) | (.855) | (.682) | (.818) | (.436) | (.894) |
| Pennsylvania | .206 | -.095 | -.080 | .051 | .044 | .037 | -.092 | .060 | .131 |
|  | .092 | .106 | .074 | .088 | .101 | .087 | .109 | .109 | .401 |
|  | [.026, .385] | [-.302, .112] | [-.225, .065] | [-.123, .224] | [-.154, .243] | [-.133, .207] | [-.306, .122] | [-.155, .274] | [-.657, .918] |
|  | (.025) | (.370) | (.280) | (.567) | (.663) | (.670) | (.399) | (.583) | (.745) |
| Rhode Island | .153 | .297 | .017 | .248 | .098 | .012 | -.174 | .126 | .777 |
|  | .153 | .201 | .155 | .194 | .196 | .186 | .223 | .196 | 1.069 |
|  | [-.147, .453] | [-.097, .690] | [-.287, .322] | [-.132, .627] | [-.287, .482] | [-.352, .376] | [-.611, .264] | [-.258, .510] | [-1.320,2.874] |
|  | (.316) | (.139) | (.911) | (.201) | (.619) | (.948) | (.436) | (.520) | (.467) |
| South Carolina | .157 | -.173 | -.146 | .074 | -.027 | .126 | .039 | .007 | .057 |
|  | .123 | .147 | .072 | .146 | .149 | .105 | .147 | .155 | .559 |
|  | [-.083, .397] | [-.461, .115] | [-.287, -.004] | [-.212, .361] | [-.319, .265] | [-.080, .333] | [-.249, .327] | [-.298, .311] | [-1.040,1.154] |
|  | (.201) | (.238) | (.043) | (.610) | (.854) | (.231) | (.790) | (.965) | (.919) |
| South Dakota | -.005 | -.410 | -.156 | -.230 | -.240 | -.324 | -.335 | -.297 | -1.996 |
|  | .263 | .112 | .073 | .117 | .295 | .284 | .288 | .114 | .967 |
|  | [-.520, .511] | [-.630, -.191] | [-.300, -.012] | [-.458, -.001] | [-.819, .339] | [-.880, .233] | [-.901, .230] | [-.520, -.073] | [-3.892, -.100] |
|  | (.986) | (<.001) | (.034) | (.049) | (.416) | (.254) | (.245) | (.009) | (.039) |
| Tennessee | .182 | .122 | -.107 | -.004 | .083 | .107 | .130 | .172 | .684 |
|  | .105 | .133 | .078 | .113 | .122 | .101 | .126 | .135 | .508 |
|  | [-.024, .388] | [-.140, .384] | [-.260, .046] | [-.225, .217] | [-.155, .322] | [-.091, .305] | [-.118, .377] | [-.093, .437] | [-.313,1.681] |
|  | (.083) | (.361) | (.170) | (.970) | (.495) | (.289) | (.305) | (.202) | (.178) |
| Texas | .151 | .045 | -.062 | .047 | -.042 | .019 | -.113 | -.099 | -.054 |
|  | .092 | .106 | .073 | .088 | .102 | .088 | .108 | .106 | .406 |
|  | [-.030, .332] | [-.162, .252] | [-.206, .082] | [-.125, .220] | [-.241, .157] | [-.154, .192] | [-.325, .100] | [-.306, .108] | [-.850, .742] |
|  | (.102) | (.671) | (.399) | (.591) | (.677) | (.829) | (.298) | (.349) | (.895) |
| Utah | .149 | -.134 | -.123 | .155 | -.060 | .010 | -.011 | .128 | .113 |
|  | .117 | .140 | .072 | .131 | .141 | .125 | .142 | .151 | .596 |
|  | [-.081, .380] | [-.408, .140] | [-.264, .017] | [-.103, .412] | [-.336, .215] | [-.235, .255] | [-.289, .267] | [-.167, .424] | [-1.057,1.282] |
|  | (.203) | (.336) | (.085) | (.239) | (.668) | (.937) | (.938) | (.395) | (.850) |
| Vermont | .315 | -.402 | -.153 | .967 | .256 | .271 | -.586 | -.322 | .344 |
|  | .108 | .132 | .086 | .105 | .124 | .109 | .134 | .134 | .503 |
|  | [.103, .528] | [-.662, -.142] | [-.322, .015] | [.761,1.172] | [.013, .498] | [.056, .486] | [-.849, -.324] | [-.585, -.060] | [-.642,1.331] |
|  | (.004) | (.002) | (.074) | (<.001) | (.039) | (.013) | (<.001) | (.016) | (.494) |
| Virginia | .280 | .068 | -.124 | .121 | -.173 | .236 | .280 | -.115 | .572 |
|  | .095 | .266 | .074 | .265 | .249 | .084 | .111 | .247 | .427 |
|  | [.094, .466] | [-.454, .589] | [-.269, .021] | [-.399, .640] | [-.661, .316] | [.071, .401] | [.062, .497] | [-.600, .370] | [-.264,1.409] |
|  | (.003) | (.799) | (.093) | (.649) | (.488) | (.005) | (.012) | (.643) | (.180) |
| Washington | .184 | -.008 | -.036 | .033 | .105 | .146 | .008 | .039 | .471 |
|  | .099 | .120 | .081 | .105 | .111 | .093 | .121 | .123 | .451 |
|  | [-.010, .378] | [-.244, .228] | [-.195, .124] | [-.173, .239] | [-.114, .323] | [-.035, .328] | [-.230, .246] | [-.202, .280] | [-.413,1.356] |
|  | (.063) | (.949) | (.662) | (.755) | (.347) | (.115) | (.946) | (.750) | (.296) |
| West Virginia | -.073 | -.043 | -.109 | -.237 | -.016 | -.069 | -.162 | -.069 | -.778 |
|  | .250 | .253 | .071 | .109 | .260 | .240 | .198 | .233 | .898 |
|  | [-.563, .417] | [-.538, .453] | [-.247, .030] | [-.450, -.024] | [-.527, .494] | [-.539, .401] | [-.552, .227] | [-.526, .388] | [-2.540, .984] |
|  | (.770) | (.865) | (.124) | (.029) | (.951) | (.774) | (.413) | (.768) | (.387) |
| Wisconsin | .182 | -.025 | -.078 | .006 | .062 | .082 | -.154 | -.112 | -.037 |
|  | .100 | .123 | .079 | .108 | .118 | .098 | .125 | .119 | .432 |
|  | [-.015, .379] | [-.266, .216] | [-.233, .077] | [-.205, .217] | [-.170, .293] | [-.110, .274] | [-.399, .092] | [-.345, .122] | [-.884, .810] |
|  | (.070) | (.838) | (.323) | (.956) | (.602) | (.401) | (.220) | (.349) | (.932) |
| Wyoming | .333 | .060 | .349 | .202 | .444 | -.145 | .479 | .753 | 2.476 |
|  | .103 | .382 | .340 | .274 | .109 | .335 | .134 | .125 | 1.289 |
|  | [.131, .535] | [-.689, .809] | [-.317,1.016] | [-.336, .740] | [.229, .659] | [-.801, .511] | [.217, .741] | [.509, .998] | [-.053,5.005] |
|  | (.001) | (.875) | (.304) | (.461) | (<.001) | (.665) | (<.001) | (<.001) | (.055) |
| Observations | 1902 | 1902 | 1902 | 1902 | 1902 | 1902 | 1902 | 1902 | 1902 |
| r2_p |  |  |  |  |  |  |  |  |  |
| chi2 |  |  |  |  |  |  |  |  |  |
| df_m | 82 | 82 | 82 | 82 | 82 | 82 | 82 | 82 | 82 |
| p | 82 | 82 | 82 | 82 | 82 | 82 | 82 | 82 | 82 |

S32 Table. Regression Results, Health Attitudes, Adjusting for Local Policies.

|  | (1) | (2) | (3) | (4) | (5) | (6) | (7) | (8) | (9) |
| --- | --- | --- | --- | --- | --- | --- | --- | --- | --- |
|  | numberdeaths | spendingincrease | notenoughtests | getsick | negativeeconomy | friendsick | cannotreturn | schoolsclosed | hardgetitems |
| Republican | -.312 | -.339 | -.706 | -.535 | -.201 | -.577 | -.332 | -.322 | -.348 |
|  | .124 | .058 | .126 | .115 | .104 | .110 | .117 | .119 | .118 |
|  | [-.554, -.069] | [-.454, -.225] | [-.952, -.459] | [-.760, -.310] | [-.406, .004] | [-.792, -.362] | [-.560, -.103] | [-.555, -.089] | [-.578, -.117] |
|  | (.012) | (<.001) | (<.001) | (<.001) | (.055) | (<.001) | (.004) | (.007) | (.003) |
| Other | -.240 | -.183 | -.230 | -.329 | -.242 | -.299 | -.413 | -.314 | -.241 |
|  | .113 | .049 | .117 | .116 | .097 | .108 | .109 | .113 | .108 |
|  | [-.461, -.018] | [-.280, -.086] | [-.460, .001] | [-.555, -.102] | [-.432, -.051] | [-.511, -.087] | [-.626, -.200] | [-.536, -.092] | [-.452, -.030] |
|  | (.034) | (<.001) | (.051) | (.005) | (.013) | (.006) | (<.001) | (.006) | (.025) |
| policies | -.129 | -.107 | .197 | .091 | .299 | -.177 | .227 | .031 | -.154 |
|  | .247 | .096 | .237 | .218 | .187 | .215 | .210 | .231 | .206 |
|  | [-.614, .355] | [-.294, .081] | [-.268, .661] | [-.337, .519] | [-.068, .667] | [-.599, .245] | [-.184, .639] | [-.422, .484] | [-.559, .250] |
|  | (.601) | (.264) | (.406) | (.678) | (.110) | (.411) | (.279) | (.894) | (.455) |
| Republican # policies | -.210 | -.013 | -.698 | .110 | -.504 | .308 | -.231 | .213 | .206 |
|  | .356 | .167 | .361 | .333 | .294 | .321 | .329 | .328 | .335 |
|  | [-.908, .488] | [-.341, .315] | [-1.407, .011] | [-.544, .764] | [-1.081, .073] | [-.322, .939] | [-.877, .415] | [-.431, .856] | [-.451, .863] |
|  | (.555) | (.938) | (.054) | (.742) | (.087) | (.338) | (.483) | (.517) | (.539) |
| Other # policies | .015 | .008 | -.252 | .100 | -.060 | -.055 | .144 | .466 | .014 |
|  | .323 | .146 | .335 | .328 | .273 | .302 | .301 | .316 | .306 |
|  | [-.618, .649] | [-.278, .295] | [-.909, .405] | [-.543, .743] | [-.595, .475] | [-.648, .538] | [-.445, .734] | [-.154,1.087] | [-.585, .614] |
|  | (.962) | (.954) | (.452) | (.761) | (.826) | (.856) | (.631) | (.141) | (.963) |
| 35- | .004 | .055 | .156 | .101 | .058 | .105 | -.036 | .086 | .032 |
|  | .076 | .035 | .079 | .070 | .064 | .069 | .070 | .074 | .069 |
|  | [-.145, .153] | [-.014, .124] | [.000, .312] | [-.035, .237] | [-.068, .184] | [-.030, .239] | [-.172, .101] | [-.058, .231] | [-.104, .169] |
|  | (.957) | (.120) | (.050) | (.146) | (.363) | (.128) | (.608) | (.241) | (.641) |
| 50- | .191 | .003 | .149 | -.054 | .083 | -.033 | -.180 | -.105 | -.211 |
|  | .074 | .034 | .072 | .066 | .060 | .064 | .065 | .070 | .065 |
|  | [.047, .336] | [-.063, .069] | [.008, .289] | [-.184, .075] | [-.034, .201] | [-.158, .092] | [-.308, -.052] | [-.243, .033] | [-.339, -.083] |
|  | (.009) | (.927) | (.038) | (.413) | (.164) | (.602) | (.006) | (.136) | (.001) |
| 70- | .485 | .105 | .213 | -.002 | -.041 | -.019 | -.239 | -.536 | -.434 |
|  | .091 | .039 | .085 | .081 | .076 | .078 | .083 | .085 | .081 |
|  | [.308, .663] | [.028, .182] | [.047, .380] | [-.161, .157] | [-.190, .108] | [-.172, .134] | [-.401, -.077] | [-.703, -.369] | [-.593, -.274] |
|  | (<.001) | (.007) | (.012) | (.978) | (.593) | (.806) | (.004) | (<.001) | (<.001) |
| gender | .018 | -.050 | -.011 | .095 | -.032 | .063 | .022 | .078 | .111 |
|  | .051 | .023 | .053 | .048 | .043 | .047 | .049 | .051 | .048 |
|  | [-.083, .119] | [-.096, -.004] | [-.115, .093] | [.001, .190] | [-.117, .053] | [-.029, .154] | [-.074, .118] | [-.022, .178] | [.017, .205] |
|  | (.723) | (.035) | (.835) | (.049) | (.465) | (.181) | (.655) | (.126) | (.021) |
| white | .018 | .103 | .339 | .034 | .146 | .030 | .173 | .004 | .080 |
|  | .061 | .029 | .064 | .058 | .052 | .055 | .057 | .059 | .057 |
|  | [-.100, .137] | [.047, .160] | [.213, .466] | [-.081, .148] | [.045, .247] | [-.079, .138] | [.062, .284] | [-.112, .120] | [-.031, .191] |
|  | (.760) | (<.001) | (<.001) | (.565) | (.005) | (.590) | (.002) | (.943) | (.159) |
| married | .012 | -.021 | -.168 | .065 | .063 | .064 | -.054 | .036 | .055 |
|  | .057 | .026 | .059 | .052 | .047 | .051 | .053 | .056 | .053 |
|  | [-.100, .124] | [-.073, .030] | [-.284, -.051] | [-.037, .167] | [-.030, .155] | [-.035, .164] | [-.158, .050] | [-.073, .146] | [-.049, .158] |
|  | (.831) | (.417) | (.005) | (.210) | (.184) | (.206) | (.306) | (.515) | (.300) |
| High school graduate | -.080 | -.068 | .006 | -.258 | -.308 | -.242 | -.380 | -.074 | -.340 |
|  | .174 | .091 | .169 | .148 | .115 | .133 | .140 | .155 | .144 |
|  | [-.421, .262] | [-.245, .110] | [-.326, .337] | [-.548, .032] | [-.533, -.083] | [-.504, .019] | [-.655, -.105] | [-.378, .230] | [-.622, -.058] |
|  | (.648) | (.457) | (.973) | (.081) | (.007) | (.069) | (.007) | (.632) | (.018) |
| Some college | -.116 | .026 | .216 | -.219 | -.213 | -.236 | -.304 | -.058 | -.471 |
|  | .176 | .093 | .172 | .151 | .118 | .136 | .144 | .159 | .147 |
|  | [-.461, .229] | [-.157, .209] | [-.121, .553] | [-.514, .076] | [-.445, .019] | [-.503, .032] | [-.587, -.022] | [-.369, .253] | [-.759, -.184] |
|  | (.510) | (.781) | (.209) | (.146) | (.072) | (.084) | (.035) | (.714) | (.001) |
| 2-year | -.080 | .023 | .158 | -.173 | -.174 | -.197 | -.345 | -.112 | -.444 |
|  | .186 | .095 | .183 | .156 | .125 | .144 | .152 | .166 | .156 |
|  | [-.445, .284] | [-.164, .210] | [-.200, .517] | [-.478, .133] | [-.419, .070] | [-.479, .086] | [-.644, -.047] | [-.437, .214] | [-.750, -.137] |
|  | (.666) | (.809) | (.386) | (.267) | (.162) | (.172) | (.023) | (.502) | (.005) |
| 4-year | -.039 | .066 | .112 | -.253 | -.179 | -.189 | -.321 | -.139 | -.457 |
|  | .177 | .093 | .175 | .151 | .120 | .137 | .145 | .160 | .148 |
|  | [-.385, .307] | [-.116, .248] | [-.232, .455] | [-.550, .044] | [-.414, .056] | [-.458, .079] | [-.605, -.036] | [-.452, .174] | [-.748, -.166] |
|  | (.825) | (.475) | (.523) | (.095) | (.135) | (.167) | (.027) | (.384) | (.002) |
| Post-grad | -.017 | .054 | .151 | -.149 | -.119 | -.120 | -.282 | -.078 | -.521 |
|  | .184 | .094 | .183 | .158 | .124 | .142 | .149 | .166 | .154 |
|  | [-.377, .343] | [-.129, .238] | [-.207, .509] | [-.459, .162] | [-.362, .125] | [-.399, .159] | [-.575, .011] | [-.404, .249] | [-.823, -.220] |
|  | (.926) | (.561) | (.407) | (.348) | (.339) | (.398) | (.059) | (.641) | (.001) |
| income=2 | .047 | -.064 | .291 | -.062 | .028 | -.070 | -.180 | -.208 | .024 |
|  | .176 | .078 | .171 | .151 | .137 | .145 | .147 | .154 | .161 |
|  | [-.298, .393] | [-.216, .089] | [-.045, .627] | [-.357, .234] | [-.241, .296] | [-.354, .214] | [-.469, .109] | [-.509, .093] | [-.292, .339] |
|  | (.787) | (.411) | (.090) | (.681) | (.840) | (.629) | (.223) | (.176) | (.884) |
| income=3 | .136 | .018 | .330 | -.084 | .143 | -.051 | -.131 | -.090 | -.013 |
|  | .171 | .076 | .168 | .147 | .134 | .144 | .149 | .160 | .158 |
|  | [-.199, .471] | [-.131, .167] | [.001, .660] | [-.372, .204] | [-.121, .406] | [-.334, .232] | [-.424, .161] | [-.403, .223] | [-.323, .296] |
|  | (.426) | (.812) | (.049) | (.569) | (.288) | (.725) | (.379) | (.575) | (.933) |
| income=4 | -.027 | -.018 | .379 | .042 | .228 | .054 | -.081 | -.105 | -.038 |
|  | .172 | .077 | .173 | .145 | .131 | .141 | .146 | .155 | .156 |
|  | [-.366, .311] | [-.169, .134] | [.040, .719] | [-.241, .326] | [-.030, .486] | [-.223, .331] | [-.367, .205] | [-.410, .200] | [-.344, .267] |
|  | (.874) | (.820) | (.028) | (.770) | (.083) | (.703) | (.579) | (.499) | (.806) |
| income=5 | .066 | .007 | .416 | -.023 | .189 | .023 | -.230 | .007 | -.026 |
|  | .172 | .076 | .172 | .148 | .136 | .141 | .144 | .149 | .155 |
|  | [-.272, .404] | [-.143, .156] | [.078, .753] | [-.314, .268] | [-.077, .456] | [-.253, .299] | [-.512, .052] | [-.286, .300] | [-.331, .278] |
|  | (.701) | (.928) | (.016) | (.876) | (.164) | (.871) | (.109) | (.963) | (.866) |
| income=6 | .140 | .018 | .404 | .055 | .179 | -.044 | -.036 | .076 | .027 |
|  | .171 | .078 | .169 | .146 | .134 | .140 | .143 | .153 | .157 |
|  | [-.195, .475] | [-.135, .171] | [.073, .735] | [-.232, .341] | [-.083, .442] | [-.319, .231] | [-.317, .245] | [-.224, .377] | [-.280, .335] |
|  | (.414) | (.814) | (.017) | (.708) | (.180) | (.752) | (.802) | (.618) | (.861) |
| income=7 | -.006 | -.033 | .397 | .010 | .105 | .018 | -.221 | -.222 | -.113 |
|  | .180 | .079 | .184 | .159 | .145 | .151 | .156 | .173 | .166 |
|  | [-.360, .348] | [-.188, .123] | [.036, .758] | [-.302, .321] | [-.179, .389] | [-.277, .314] | [-.526, .085] | [-.561, .117] | [-.439, .213] |
|  | (.974) | (.679) | (.031) | (.950) | (.468) | (.904) | (.156) | (.199) | (.496) |
| income=8 | .071 | -.018 | .350 | -.278 | .184 | -.228 | -.214 | -.129 | -.238 |
|  | .174 | .079 | .178 | .155 | .136 | .147 | .153 | .162 | .164 |
|  | [-.271, .413] | [-.174, .138] | [.002, .699] | [-.582, .025] | [-.083, .451] | [-.516, .059] | [-.515, .087] | [-.446, .188] | [-.559, .083] |
|  | (.683) | (.823) | (.049) | (.072) | (.177) | (.120) | (.163) | (.426) | (.146) |
| income=9 | .191 | .021 | .427 | -.240 | .141 | -.199 | -.070 | -.149 | -.158 |
|  | .171 | .076 | .180 | .149 | .136 | .143 | .148 | .159 | .157 |
|  | [-.144, .525] | [-.129, .170] | [.075, .780] | [-.532, .051] | [-.125, .407] | [-.479, .080] | [-.359, .220] | [-.461, .163] | [-.465, .149] |
|  | (.264) | (.785) | (.018) | (.106) | (.299) | (.163) | (.638) | (.350) | (.314) |
| income=10 | .101 | -.022 | .583 | -.193 | .178 | -.145 | -.180 | -.246 | -.325 |
|  | .170 | .078 | .182 | .152 | .142 | .147 | .150 | .157 | .164 |
|  | [-.231, .434] | [-.175, .131] | [.227, .939] | [-.490, .105] | [-.100, .456] | [-.434, .144] | [-.475, .115] | [-.553, .061] | [-.646, -.004] |
|  | (.550) | (.779) | (.001) | (.204) | (.210) | (.326) | (.232) | (.117) | (.047) |
| income=11 | .257 | -.021 | .431 | .019 | .374 | .019 | .016 | -.174 | -.190 |
|  | .174 | .077 | .178 | .155 | .137 | .151 | .151 | .164 | .163 |
|  | [-.084, .599] | [-.171, .130] | [.081, .781] | [-.285, .323] | [.106, .642] | [-.278, .315] | [-.279, .311] | [-.495, .148] | [-.510, .131] |
|  | (.140) | (.788) | (.016) | (.901) | (.006) | (.902) | (.915) | (.289) | (.246) |
| income=12 | .054 | .014 | .344 | -.135 | .246 | -.050 | -.021 | -.268 | -.309 |
|  | .183 | .078 | .206 | .165 | .145 | .165 | .162 | .179 | .175 |
|  | [-.306, .413] | [-.139, .167] | [-.060, .748] | [-.458, .188] | [-.038, .530] | [-.374, .274] | [-.339, .297] | [-.620, .083] | [-.653, .035] |
|  | (.770) | (.859) | (.095) | (.414) | (.089) | (.762) | (.898) | (.135) | (.078) |
| income=13 | .114 | .083 | .698 | -.231 | .113 | -.175 | -.101 | -.600 | -.613 |
|  | .271 | .095 | .237 | .207 | .189 | .202 | .214 | .264 | .230 |
|  | [-.417, .645] | [-.104, .270] | [.234,1.162] | [-.637, .176] | [-.259, .484] | [-.572, .222] | [-.521, .319] | [-1.118, -.081] | [-1.065, -.162] |
|  | (.674) | (.383) | (.003) | (.266) | (.552) | (.387) | (.638) | (.023) | (.008) |
| income=14 | .175 | -.046 | .570 | -.202 | .114 | -.078 | -.381 | -.209 | -.528 |
|  | .269 | .104 | .215 | .245 | .219 | .245 | .265 | .208 | .220 |
|  | [-.353, .703] | [-.250, .159] | [.148, .991] | [-.682, .277] | [-.315, .542] | [-.559, .403] | [-.901, .140] | [-.616, .198] | [-.960, -.095] |
|  | (.515) | (.660) | (.008) | (.408) | (.603) | (.750) | (.152) | (.315) | (.017) |
| income=15 | .079 | .212 | 1.483 | .514 | .682 | .234 | .329 | -.067 | -1.038 |
|  | .797 | .119 | .306 | .554 | .204 | .715 | .454 | .679 | .440 |
|  | [-1.485,1.643] | [-.022, .445] | [.883,2.084] | [-.573,1.601] | [.282,1.083] | [-1.169,1.637] | [-.562,1.219] | [-1.398,1.264] | [-1.901, -.175] |
|  | (.921) | (.076) | (<.001) | (.354) | (.001) | (.744) | (.469) | (.922) | (.018) |
| income=16 | .526 | -.098 | .618 | -.303 | .034 | -.180 | .192 | .170 | -.242 |
|  | .327 | .147 | .367 | .354 | .302 | .348 | .275 | .333 | .325 |
|  | [-.114,1.167] | [-.387, .190] | [-.101,1.337] | [-.998, .391] | [-.559, .628] | [-.863, .502] | [-.348, .732] | [-.484, .824] | [-.880, .397] |
|  | (.107) | (.505) | (.092) | (.392) | (.910) | (.604) | (.485) | (.610) | (.458) |
| income=17 | .126 | -.044 | .403 | -.203 | .059 | -.191 | -.268 | -.343 | -.327 |
|  | .160 | .073 | .159 | .137 | .127 | .131 | .134 | .144 | .145 |
|  | [-.188, .441] | [-.186, .098] | [.091, .715] | [-.471, .065] | [-.191, .308] | [-.449, .066] | [-.530, -.006] | [-.627, -.060] | [-.612, -.043] |
|  | (.432) | (.544) | (.011) | (.138) | (.645) | (.145) | (.045) | (.018) | (.024) |
| Rural Code=2 | .031 | -.014 | -.052 | .058 | -.099 | .003 | -.071 | -.165 | -.043 |
|  | .069 | .031 | .069 | .067 | .059 | .064 | .066 | .069 | .066 |
|  | [-.104, .165] | [-.075, .047] | [-.187, .083] | [-.072, .189] | [-.215, .016] | [-.122, .128] | [-.201, .059] | [-.299, -.030] | [-.172, .086] |
|  | (.657) | (.643) | (.451) | (.382) | (.091) | (.957) | (.285) | (.016) | (.512) |
| Rural Code=3 | -.138 | -.059 | -.198 | -.039 | -.091 | .043 | -.116 | -.013 | .048 |
|  | .128 | .060 | .115 | .111 | .102 | .105 | .111 | .111 | .105 |
|  | [-.389, .112] | [-.177, .059] | [-.423, .028] | [-.257, .179] | [-.290, .108] | [-.163, .250] | [-.333, .102] | [-.232, .205] | [-.158, .254] |
|  | (.278) | (.328) | (.086) | (.726) | (.372) | (.679) | (.297) | (.905) | (.647) |
| Rural Code=4 | .128 | .097 | -.085 | -.013 | -.391 | -.341 | -.328 | -.402 | -.517 |
|  | .276 | .100 | .279 | .242 | .239 | .231 | .259 | .293 | .275 |
|  | [-.413, .670] | [-.100, .294] | [-.632, .461] | [-.487, .461] | [-.860, .078] | [-.795, .113] | [-.835, .179] | [-.976, .173] | [-1.056, .023] |
|  | (.643) | (.334) | (.759) | (.957) | (.102) | (.141) | (.205) | (.171) | (.060) |
| Rural Code=6 | .604 | .560 | .550 | .755 | 1.158 | .973 | .983 | 1.688 | .555 |
|  | .597 | .085 | .642 | .736 | .171 | .655 | .461 | .211 | .185 |
|  | [-.567,1.775] | [.393, .728] | [-.709,1.810] | [-.688,2.199] | [.823,1.494] | [-.312,2.257] | [.079,1.886] | [1.274,2.101] | [.192, .918] |
|  | (.312) | (<.001) | (.392) | (.305) | (<.001) | (.138) | (.033) | (<.001) | (.003) |
| Arizona | .060 | -.049 | .051 | -.147 | -.271 | -.106 | -.348 | -.557 | -.035 |
|  | .273 | .112 | .275 | .241 | .211 | .229 | .268 | .230 | .228 |
|  | [-.474, .595] | [-.270, .171] | [-.489, .591] | [-.619, .325] | [-.684, .142] | [-.556, .344] | [-.873, .177] | [-1.009, -.106] | [-.482, .413] |
|  | (.825) | (.660) | (.852) | (.542) | (.199) | (.644) | (.194) | (.016) | (.880) |
| Arkansas | .231 | -.053 | -.051 | .039 | -.336 | .158 | .093 | -.212 | .076 |
|  | .357 | .206 | .423 | .320 | .351 | .323 | .354 | .326 | .280 |
|  | [-.469, .930] | [-.456, .350] | [-.880, .777] | [-.590, .667] | [-1.025, .353] | [-.475, .791] | [-.602, .788] | [-.851, .427] | [-.473, .626] |
|  | (.518) | (.797) | (.903) | (.904) | (.339) | (.624) | (.792) | (.516) | (.785) |
| California | .268 | .032 | -.063 | .066 | -.162 | .073 | -.085 | -.259 | .190 |
|  | .241 | .097 | .250 | .213 | .175 | .209 | .243 | .202 | .206 |
|  | [-.206, .741] | [-.159, .223] | [-.553, .426] | [-.351, .484] | [-.506, .182] | [-.336, .483] | [-.561, .392] | [-.656, .137] | [-.214, .595] |
|  | (.268) | (.740) | (.800) | (.756) | (.356) | (.725) | (.728) | (.200) | (.356) |
| Colorado | .537 | -.076 | -.162 | .049 | -.412 | .014 | -.204 | -.338 | -.108 |
|  | .285 | .134 | .309 | .261 | .230 | .254 | .288 | .248 | .250 |
|  | [-.023,1.097] | [-.339, .186] | [-.768, .445] | [-.464, .561] | [-.863, .040] | [-.484, .513] | [-.769, .361] | [-.824, .149] | [-.598, .382] |
|  | (.060) | (.569) | (.601) | (.852) | (.074) | (.956) | (.479) | (.174) | (.666) |
| Connecticut | .240 | .198 | .213 | .102 | -.021 | .114 | .115 | -.162 | .131 |
|  | .294 | .123 | .296 | .267 | .238 | .242 | .309 | .268 | .272 |
|  | [-.337, .816] | [-.044, .440] | [-.369, .794] | [-.422, .626] | [-.489, .446] | [-.361, .589] | [-.491, .720] | [-.688, .363] | [-.403, .665] |
|  | (.415) | (.109) | (.473) | (.703) | (.928) | (.639) | (.710) | (.545) | (.631) |
| Delaware | .712 | .091 | -.629 | -.314 | -.214 | -.951 | -.629 | -.860 | -.829 |
|  | .402 | .202 | .403 | .416 | .311 | .250 | .303 | .316 | .261 |
|  | [-.076,1.499] | [-.305, .487] | [-1.420, .162] | [-1.130, .501] | [-.824, .396] | [-1.441, -.461] | [-1.224, -.034] | [-1.481, -.239] | [-1.340, -.317] |
|  | (.077) | (.652) | (.119) | (.450) | (.492) | (<.001) | (.038) | (.007) | (.002) |
| Florida | .520 | .031 | .047 | .004 | -.207 | -.026 | -.006 | -.200 | .163 |
|  | .246 | .100 | .258 | .217 | .184 | .211 | .248 | .213 | .213 |
|  | [.037,1.002] | [-.164, .227] | [-.458, .552] | [-.421, .429] | [-.567, .153] | [-.440, .388] | [-.492, .480] | [-.617, .218] | [-.255, .581] |
|  | (.035) | (.752) | (.855) | (.985) | (.260) | (.902) | (.981) | (.348) | (.445) |
| Georgia | .184 | .034 | .178 | -.008 | -.303 | .071 | .064 | -.368 | .098 |
|  | .287 | .115 | .272 | .243 | .207 | .237 | .270 | .238 | .237 |
|  | [-.378, .746] | [-.191, .259] | [-.355, .712] | [-.484, .468] | [-.710, .103] | [-.393, .534] | [-.465, .593] | [-.834, .099] | [-.366, .563] |
|  | (.521) | (.767) | (.512) | (.974) | (.144) | (.765) | (.811) | (.122) | (.678) |
| Idaho | .177 | -.279 | -.552 | -.733 | -.285 | -.794 | -.147 | -.476 | .008 |
|  | .356 | .235 | .488 | .348 | .292 | .328 | .347 | .348 | .335 |
|  | [-.520, .875] | [-.740, .182] | [-1.510, .405] | [-1.417, -.050] | [-.858, .289] | [-1.437, -.150] | [-.827, .534] | [-1.158, .206] | [-.648, .665] |
|  | (.618) | (.235) | (.258) | (.035) | (.331) | (.016) | (.672) | (.171) | (.980) |
| Illinois | .358 | .021 | -.086 | .304 | -.270 | .043 | -.072 | -.226 | .098 |
|  | .254 | .104 | .263 | .225 | .199 | .226 | .261 | .225 | .219 |
|  | [-.141, .856] | [-.183, .225] | [-.602, .430] | [-.138, .746] | [-.660, .119] | [-.400, .486] | [-.584, .440] | [-.667, .216] | [-.331, .527] |
|  | (.159) | (.839) | (.745) | (.177) | (.174) | (.848) | (.782) | (.316) | (.654) |
| Indiana | .059 | -.001 | .149 | .076 | -.267 | -.056 | -.079 | -.386 | -.095 |
|  | .279 | .116 | .302 | .268 | .233 | .261 | .308 | .279 | .272 |
|  | [-.487, .605] | [-.229, .228] | [-.442, .741] | [-.450, .602] | [-.725, .190] | [-.568, .456] | [-.682, .525] | [-.934, .161] | [-.629, .440] |
|  | (.832) | (.995) | (.621) | (.777) | (.252) | (.830) | (.798) | (.166) | (.728) |
| Iowa | .658 | -.213 | -.372 | .102 | -.155 | -.109 | -.064 | -.298 | .004 |
|  | .544 | .158 | .324 | .324 | .286 | .298 | .350 | .391 | .380 |
|  | [-.410,1.725] | [-.523, .097] | [-1.007, .264] | [-.533, .738] | [-.716, .407] | [-.693, .475] | [-.750, .622] | [-1.065, .469] | [-.741, .749] |
|  | (.227) | (.178) | (.251) | (.752) | (.589) | (.714) | (.854) | (.446) | (.992) |
| Kansas | .709 | .318 | .123 | .625 | .440 | .601 | .402 | .308 | .163 |
|  | .297 | .127 | .509 | .563 | .189 | .445 | .360 | .375 | .269 |
|  | [.126,1.291] | [.069, .567] | [-.875,1.121] | [-.478,1.729] | [.069, .811] | [-.272,1.475] | [-.303,1.107] | [-.428,1.043] | [-.364, .691] |
|  | (.017) | (.012) | (.809) | (.267) | (.020) | (.177) | (.264) | (.412) | (.544) |
| Kentucky | -.117 | -.113 | -1.063 | -.578 | .106 | -.820 | -.670 | -.760 | -.792 |
|  | .651 | .223 | .448 | .543 | .420 | .319 | .629 | .486 | .296 |
|  | [-1.394,1.160] | [-.550, .324] | [-1.942, -.185] | [-1.642, .486] | [-.718, .930] | [-1.447, -.194] | [-1.903, .563] | [-1.713, .193] | [-1.373, -.212] |
|  | (.857) | (.613) | (.018) | (.287) | (.800) | (.010) | (.287) | (.118) | (.007) |
| Louisiana | .460 | .038 | -.004 | -.276 | -.319 | -.219 | -.160 | -.397 | .128 |
|  | .332 | .148 | .348 | .313 | .296 | .294 | .331 | .311 | .303 |
|  | [-.192,1.111] | [-.252, .327] | [-.687, .679] | [-.890, .338] | [-.899, .261] | [-.796, .357] | [-.809, .488] | [-1.007, .213] | [-.466, .723] |
|  | (.167) | (.799) | (.991) | (.378) | (.281) | (.456) | (.628) | (.202) | (.672) |
| Maine | -.357 | .402 | -.623 | -.010 | -.452 | -.263 | .180 | .360 | -.066 |
|  | .467 | .108 | .657 | .545 | .474 | .236 | .500 | .260 | .390 |
|  | [-1.274, .560] | [.191, .614] | [-1.911, .665] | [-1.079,1.059] | [-1.382, .479] | [-.727, .200] | [-.800,1.160] | [-.151, .871] | [-.832, .699] |
|  | (.445) | (<.001) | (.343) | (.985) | (.341) | (.265) | (.719) | (.167) | (.865) |
| Maryland | .340 | .115 | -.318 | -.045 | -.079 | .207 | -.114 | -.160 | .012 |
|  | .298 | .127 | .343 | .280 | .225 | .275 | .299 | .271 | .277 |
|  | [-.244, .924] | [-.133, .364] | [-.990, .355] | [-.593, .503] | [-.520, .363] | [-.332, .746] | [-.701, .473] | [-.692, .372] | [-.532, .555] |
|  | (.253) | (.363) | (.354) | (.872) | (.726) | (.451) | (.703) | (.555) | (.967) |
| Massachusetts | .304 | -.003 | -.023 | .254 | -.102 | .309 | .112 | -.137 | .303 |
|  | .280 | .112 | .276 | .250 | .208 | .236 | .272 | .249 | .232 |
|  | [-.246, .854] | [-.221, .216] | [-.564, .519] | [-.236, .744] | [-.509, .306] | [-.154, .771] | [-.420, .645] | [-.624, .351] | [-.152, .759] |
|  | (.278) | (.981) | (.934) | (.309) | (.625) | (.190) | (.679) | (.583) | (.191) |
| Michigan | .258 | .060 | .010 | .136 | -.114 | -.034 | .033 | -.381 | .007 |
|  | .250 | .104 | .266 | .230 | .189 | .225 | .256 | .224 | .224 |
|  | [-.232, .748] | [-.145, .264] | [-.512, .533] | [-.315, .587] | [-.485, .257] | [-.475, .407] | [-.469, .534] | [-.819, .058] | [-.433, .447] |
|  | (.302) | (.567) | (.969) | (.555) | (.547) | (.879) | (.898) | (.089) | (.976) |
| Minnesota | .371 | -.033 | .439 | .121 | -.134 | -.096 | -.028 | -.265 | -.095 |
|  | .299 | .120 | .285 | .266 | .216 | .258 | .294 | .257 | .249 |
|  | [-.215, .958] | [-.268, .202] | [-.121, .998] | [-.401, .643] | [-.557, .289] | [-.602, .410] | [-.605, .549] | [-.770, .240] | [-.583, .393] |
|  | (.215) | (.784) | (.124) | (.650) | (.535) | (.709) | (.924) | (.303) | (.703) |
| Mississippi | .518 | .124 | -.255 | .187 | -.333 | -.071 | -.266 | -.744 | .035 |
|  | .413 | .187 | .353 | .416 | .466 | .428 | .438 | .468 | .377 |
|  | [-.292,1.327] | [-.242, .490] | [-.948, .438] | [-.630,1.003] | [-1.246, .580] | [-.911, .769] | [-1.125, .594] | [-1.663, .174] | [-.705, .774] |
|  | (.210) | (.506) | (.471) | (.654) | (.475) | (.868) | (.544) | (.112) | (.927) |
| Missouri | .200 | -.033 | -.213 | .034 | -.322 | .034 | -.135 | -.284 | .038 |
|  | .317 | .122 | .285 | .259 | .212 | .261 | .279 | .252 | .245 |
|  | [-.422, .822] | [-.272, .207] | [-.772, .346] | [-.473, .541] | [-.737, .094] | [-.477, .546] | [-.682, .412] | [-.778, .211] | [-.444, .519] |
|  | (.529) | (.788) | (.455) | (.896) | (.129) | (.895) | (.628) | (.261) | (.878) |
| Montana | .063 | -.530 | .565 | 1.396 | .692 | .007 | 1.278 | -.240 | 1.130 |
|  | .422 | .732 | .429 | .250 | .314 | 1.053 | .275 | .909 | .465 |
|  | [-.764, .890] | [-1.966, .905] | [-.276,1.406] | [.907,1.886] | [.076,1.309] | [-2.059,2.072] | [.738,1.817] | [-2.023,1.542] | [.217,2.043] |
|  | (.881) | (.469) | (.188) | (<.001) | (.028) | (.995) | (<.001) | (.791) | (.015) |
| Nebraska | .207 | -.390 | .164 | -.059 | -.094 | -.119 | -.218 | -.418 | -.007 |
|  | .348 | .298 | .392 | .538 | .361 | .478 | .539 | .341 | .505 |
|  | [-.476, .890] | [-.975, .194] | [-.604, .932] | [-1.114, .997] | [-.802, .614] | [-1.057, .818] | [-1.276, .840] | [-1.087, .251] | [-.999, .984] |
|  | (.552) | (.190) | (.675) | (.913) | (.794) | (.803) | (.686) | (.221) | (.988) |
| Nevada | .435 | -.052 | .066 | .060 | -.172 | .141 | -.185 | -.377 | .111 |
|  | .277 | .117 | .293 | .261 | .234 | .250 | .294 | .270 | .260 |
|  | [-.107, .977] | [-.281, .178] | [-.509, .641] | [-.453, .572] | [-.630, .287] | [-.349, .631] | [-.762, .392] | [-.907, .153] | [-.399, .620] |
|  | (.116) | (.660) | (.822) | (.820) | (.462) | (.572) | (.529) | (.163) | (.671) |
| New Hampshire | .770 | -.185 | -.027 | .141 | -.248 | .122 | -.183 | -.146 | .242 |
|  | .353 | .185 | .341 | .330 | .273 | .291 | .337 | .358 | .315 |
|  | [.078,1.463] | [-.547, .177] | [-.696, .642] | [-.506, .788] | [-.783, .287] | [-.450, .693] | [-.844, .478] | [-.847, .556] | [-.375, .859] |
|  | (.029) | (.316) | (.937) | (.669) | (.363) | (.677) | (.587) | (.684) | (.441) |
| New Jersey | .581 | .031 | -.226 | .228 | -.240 | .205 | .144 | -.185 | .329 |
|  | .269 | .107 | .275 | .239 | .209 | .234 | .266 | .226 | .240 |
|  | [.054,1.108] | [-.179, .242] | [-.765, .313] | [-.241, .698] | [-.651, .171] | [-.255, .664] | [-.378, .665] | [-.629, .259] | [-.142, .799] |
|  | (.031) | (.771) | (.411) | (.340) | (.252) | (.383) | (.590) | (.414) | (.171) |
| New Mexico | .385 | .137 | -.169 | .347 | -.141 | .122 | .230 | -.009 | .032 |
|  | .324 | .137 | .394 | .322 | .271 | .320 | .312 | .327 | .295 |
|  | [-.250,1.021] | [-.132, .407] | [-.942, .605] | [-.285, .979] | [-.672, .390] | [-.505, .749] | [-.381, .841] | [-.650, .633] | [-.546, .611] |
|  | (.235) | (.318) | (.669) | (.281) | (.603) | (.703) | (.460) | (.978) | (.913) |
| New York | .581 | .013 | -.301 | .240 | -.140 | .172 | .034 | -.248 | .253 |
|  | .258 | .102 | .265 | .223 | .186 | .220 | .250 | .219 | .215 |
|  | [.075,1.086] | [-.187, .213] | [-.821, .219] | [-.197, .677] | [-.504, .224] | [-.260, .604] | [-.456, .525] | [-.676, .181] | [-.168, .674] |
|  | (.024) | (.899) | (.256) | (.281) | (.452) | (.435) | (.891) | (.257) | (.240) |
| North Carolina | .165 | .061 | .108 | .105 | -.070 | .033 | .025 | -.115 | .231 |
|  | .292 | .114 | .272 | .256 | .206 | .249 | .276 | .254 | .248 |
|  | [-.408, .739] | [-.163, .284] | [-.426, .642] | [-.397, .608] | [-.474, .334] | [-.454, .521] | [-.516, .566] | [-.613, .383] | [-.257, .718] |
|  | (.572) | (.594) | (.692) | (.681) | (.734) | (.893) | (.928) | (.651) | (.353) |
| North Dakota | .689 | -.032 | -.104 | -.220 | -.407 | -.529 | -.047 | -.824 | -.436 |
|  | .504 | .194 | .568 | .369 | .307 | .260 | .366 | .511 | .389 |
|  | [-.298,1.677] | [-.413, .348] | [-1.219,1.010] | [-.944, .503] | [-1.009, .195] | [-1.039, -.018] | [-.766, .671] | [-1.826, .178] | [-1.200, .327] |
|  | (.171) | (.868) | (.855) | (.551) | (.185) | (.042) | (.897) | (.107) | (.262) |
| Ohio | .276 | -.021 | -.095 | -.027 | -.130 | -.020 | -.152 | -.632 | -.093 |
|  | .257 | .106 | .269 | .244 | .195 | .235 | .266 | .226 | .228 |
|  | [-.228, .781] | [-.229, .187] | [-.623, .434] | [-.505, .451] | [-.512, .252] | [-.481, .441] | [-.674, .370] | [-1.075, -.190] | [-.540, .353] |
|  | (.283) | (.843) | (.725) | (.912) | (.506) | (.933) | (.568) | (.005) | (.682) |
| Oklahoma | -.163 | .101 | .001 | -.181 | -.093 | .295 | -.117 | -.454 | .037 |
|  | .505 | .211 | .473 | .367 | .332 | .310 | .451 | .398 | .447 |
|  | [-1.152, .827] | [-.312, .514] | [-.927, .928] | [-.901, .539] | [-.745, .559] | [-.313, .902] | [-1.002, .768] | [-1.234, .327] | [-.840, .914] |
|  | (.747) | (.631) | (.998) | (.622) | (.779) | (.342) | (.796) | (.254) | (.934) |
| Oregon | .026 | .009 | .290 | -.037 | .072 | -.093 | .109 | -.347 | .142 |
|  | .264 | .110 | .263 | .250 | .195 | .237 | .271 | .237 | .246 |
|  | [-.492, .544] | [-.205, .224] | [-.226, .807] | [-.527, .453] | [-.311, .455] | [-.559, .372] | [-.422, .640] | [-.812, .117] | [-.341, .624] |
|  | (.922) | (.932) | (.270) | (.883) | (.711) | (.694) | (.686) | (.143) | (.565) |
| Pennsylvania | .287 | -.005 | .083 | .087 | -.129 | -.026 | .055 | -.126 | .139 |
|  | .248 | .104 | .248 | .223 | .187 | .214 | .251 | .219 | .217 |
|  | [-.199, .772] | [-.209, .199] | [-.404, .570] | [-.349, .524] | [-.496, .238] | [-.446, .393] | [-.438, .548] | [-.555, .302] | [-.286, .564] |
|  | (.247) | (.964) | (.739) | (.695) | (.491) | (.902) | (.827) | (.563) | (.521) |
| Rhode Island | .471 | .036 | -.136 | .558 | -.093 | .298 | .218 | -.265 | .047 |
|  | .432 | .194 | .392 | .391 | .335 | .438 | .390 | .431 | .415 |
|  | [-.375,1.317] | [-.345, .416] | [-.904, .632] | [-.209,1.325] | [-.750, .564] | [-.562,1.157] | [-.547, .983] | [-1.111, .581] | [-.767, .860] |
|  | (.275) | (.854) | (.729) | (.154) | (.781) | (.497) | (.577) | (.539) | (.911) |
| South Carolina | -.320 | .097 | -.315 | -.101 | -.006 | -.037 | .301 | .118 | -.271 |
|  | .330 | .136 | .346 | .293 | .242 | .312 | .317 | .359 | .310 |
|  | [-.967, .326] | [-.170, .365] | [-.993, .364] | [-.676, .474] | [-.482, .469] | [-.649, .575] | [-.321, .923] | [-.587, .822] | [-.879, .338] |
|  | (.331) | (.475) | (.363) | (.730) | (.979) | (.906) | (.343) | (.743) | (.383) |
| South Dakota | .513 | -.261 | 1.043 | -.534 | -.266 | -.650 | .654 | -.924 | -.360 |
|  | .383 | .290 | .529 | .593 | .521 | .606 | .630 | .821 | .346 |
|  | [-.239,1.265] | [-.831, .308] | [.004,2.081] | [-1.696, .629] | [-1.287, .755] | [-1.839, .539] | [-.581,1.889] | [-2.534, .686] | [-1.040, .319] |
|  | (.181) | (.368) | (.049) | (.368) | (.609) | (.284) | (.299) | (.260) | (.298) |
| Tennessee | .136 | .046 | .071 | .293 | .016 | .102 | .220 | -.351 | .138 |
|  | .304 | .114 | .274 | .278 | .223 | .264 | .282 | .272 | .278 |
|  | [-.461, .733] | [-.177, .269] | [-.468, .609] | [-.253, .838] | [-.422, .454] | [-.416, .620] | [-.333, .772] | [-.884, .181] | [-.408, .684] |
|  | (.655) | (.686) | (.797) | (.293) | (.943) | (.700) | (.436) | (.196) | (.620) |
| Texas | .203 | .064 | .019 | -.033 | -.124 | -.133 | -.006 | -.226 | .177 |
|  | .254 | .101 | .256 | .223 | .183 | .218 | .251 | .214 | .217 |
|  | [-.296, .702] | [-.134, .261] | [-.484, .522] | [-.471, .405] | [-.483, .235] | [-.560, .293] | [-.499, .487] | [-.645, .194] | [-.248, .601] |
|  | (.425) | (.526) | (.941) | (.884) | (.498) | (.540) | (.981) | (.292) | (.415) |
| Utah | .390 | -.130 | .499 | .217 | -.275 | -.064 | -.342 | -.388 | .096 |
|  | .329 | .168 | .262 | .312 | .262 | .301 | .351 | .345 | .317 |
|  | [-.255,1.035] | [-.460, .200] | [-.016,1.014] | [-.394, .829] | [-.789, .239] | [-.655, .527] | [-1.031, .348] | [-1.063, .288] | [-.526, .718] |
|  | (.236) | (.440) | (.057) | (.486) | (.294) | (.832) | (.331) | (.261) | (.763) |
| Vermont | -.508 | -.724 | -.996 | -.923 | -.386 | -.924 | -.024 | -.834 | -.138 |
|  | .307 | .140 | .316 | .286 | .236 | .271 | .305 | .280 | .271 |
|  | [-1.110, .093] | [-.999, -.450] | [-1.617, -.376] | [-1.485, -.361] | [-.848, .077] | [-1.455, -.393] | [-.622, .575] | [-1.384, -.284] | [-.669, .392] |
|  | (.098) | (<.001) | (.002) | (.001) | (.102) | (.001) | (.939) | (.003) | (.609) |
| Virginia | .539 | .321 | .763 | .801 | .151 | .574 | -.261 | -.877 | -.497 |
|  | .237 | .110 | .317 | .457 | .324 | .472 | .356 | .434 | .347 |
|  | [.074,1.005] | [.106, .536] | [.141,1.385] | [-.094,1.697] | [-.484, .787] | [-.352,1.501] | [-.958, .437] | [-1.728, -.025] | [-1.178, .184] |
|  | (.023) | (.003) | (.016) | (.080) | (.640) | (.224) | (.464) | (.044) | (.153) |
| Washington | .621 | -.007 | .126 | .211 | -.317 | .158 | .028 | -.209 | .011 |
|  | .273 | .126 | .267 | .252 | .212 | .244 | .275 | .245 | .248 |
|  | [.085,1.157] | [-.255, .240] | [-.397, .649] | [-.284, .705] | [-.733, .098] | [-.320, .636] | [-.512, .567] | [-.688, .271] | [-.475, .496] |
|  | (.023) | (.954) | (.638) | (.404) | (.135) | (.518) | (.920) | (.393) | (.966) |
| West Virginia | .867 | -.001 | -.319 | -.027 | .029 | .167 | .070 | -.343 | .211 |
|  | .625 | .369 | .419 | .518 | .355 | .583 | .438 | .580 | .402 |
|  | [-.360,2.093] | [-.724, .722] | [-1.140, .502] | [-1.042, .989] | [-.666, .725] | [-.977,1.311] | [-.788, .928] | [-1.481, .796] | [-.578,1.000] |
|  | (.166) | (.998) | (.446) | (.959) | (.934) | (.775) | (.873) | (.555) | (.600) |
| Wisconsin | .388 | -.036 | -.086 | .010 | -.348 | -.307 | -.338 | -.502 | -.059 |
|  | .293 | .121 | .277 | .268 | .220 | .255 | .280 | .238 | .243 |
|  | [-.186, .962] | [-.273, .202] | [-.628, .457] | [-.516, .536] | [-.780, .083] | [-.807, .192] | [-.887, .212] | [-.969, -.036] | [-.535, .417] |
|  | (.185) | (.768) | (.757) | (.969) | (.114) | (.228) | (.228) | (.035) | (.808) |
| Wyoming | 1.179 | .412 | -.554 | -.032 | .281 | -.169 | .401 | -.249 | -.121 |
|  | .327 | .112 | 1.358 | 1.182 | .355 | 1.181 | .785 | 1.168 | 1.187 |
|  | [.539,1.820] | [.193, .632] | [-3.218,2.110] | [-2.350,2.286] | [-.415, .978] | [-2.486,2.147] | [-1.139,1.941] | [-2.539,2.042] | [-2.450,2.208] |
|  | (<.001) | (<.001) | (.684) | (.979) | (.428) | (.886) | (.610) | (.831) | (.919) |
| Observations | 1902 | 1900 | 1891 | 1901 | 1900 | 1901 | 1901 | 1901 | 1900 |
| r2_p |  |  |  |  |  |  |  |  |  |
| chi2 |  |  |  |  |  |  |  |  |  |
| df_m | 82 | 82 | 82 | 82 | 82 | 82 | 82 | 82 | 82 |
| p | 82 | 82 | 82 | 82 | 82 | 82 | 82 | 82 | 82 |

S33 Table. Regression Results, Health Policies, Adjusting for Local Policies.

|  | (1) | (2) | (3) | (4) | (5) | (6) | (7) | (8) | (9) |
| --- | --- | --- | --- | --- | --- | --- | --- | --- | --- |
|  | freetesting | waivecosts | closeschools | canceleverything | paidleave | compensatefirms | nationalguard | stopevents | stateslead |
| Republican | -.653 | -.807 | -.495 | -.547 | -.693 | -.016 | -.263 | -.556 | .097 |
|  | .117 | .120 | .128 | .127 | .119 | .134 | .148 | .149 | .118 |
|  | [-.884, -.423] | [-1.042, -.572] | [-.747, -.243] | [-.797, -.298] | [-.927, -.460] | [-.279, .247] | [-.553, .026] | [-.848, -.265] | [-.135, .329] |
|  | (<.001) | (<.001) | (<.001) | (<.001) | (<.001) | (.905) | (.074) | (<.001) | (.412) |
| Other | -.333 | -.420 | -.337 | -.397 | -.348 | -.217 | -.352 | -.274 | -.219 |
|  | .093 | .102 | .116 | .115 | .107 | .133 | .138 | .135 | .119 |
|  | [-.515, -.151] | [-.621, -.219] | [-.564, -.110] | [-.622, -.171] | [-.557, -.139] | [-.479, .044] | [-.622, -.081] | [-.538, -.010] | [-.451, .014] |
|  | (<.001) | (<.001) | (.004) | (.001) | (.001) | (.103) | (.011) | (.042) | (.065) |
| policies | .032 | .022 | -.049 | -.117 | .081 | .123 | .167 | -.093 | -.022 |
|  | .177 | .202 | .233 | .207 | .191 | .297 | .267 | .268 | .248 |
|  | [-.315, .378] | [-.374, .419] | [-.505, .408] | [-.523, .290] | [-.295, .456] | [-.460, .707] | [-.356, .690] | [-.617, .432] | [-.509, .464] |
|  | (.857) | (.912) | (.834) | (.573) | (.674) | (.678) | (.531) | (.729) | (.928) |
| Republican # policies | .081 | .103 | -.083 | .338 | .187 | .489 | .128 | -.022 | -.046 |
|  | .339 | .347 | .379 | .352 | .343 | .376 | .412 | .420 | .334 |
|  | [-.584, .746] | [-.576, .783] | [-.826, .661] | [-.352,1.029] | [-.487, .861] | [-.249,1.226] | [-.681, .936] | [-.845, .801] | [-.700, .608] |
|  | (.812) | (.765) | (.828) | (.336) | (.587) | (.194) | (.757) | (.958) | (.891) |
| Other # policies | .148 | .246 | .024 | -.013 | -.118 | -.100 | -.237 | -.043 | .177 |
|  | .244 | .283 | .331 | .313 | .298 | .385 | .379 | .387 | .321 |
|  | [-.330, .626] | [-.309, .801] | [-.624, .672] | [-.627, .600] | [-.703, .466] | [-.854, .655] | [-.982, .507] | [-.803, .717] | [-.453, .808] |
|  | (.543) | (.384) | (.942) | (.966) | (.691) | (.795) | (.532) | (.912) | (.581) |
| 35- | .192 | .254 | .118 | .167 | .199 | .223 | .042 | .006 | .203 |
|  | .062 | .068 | .074 | .073 | .070 | .086 | .087 | .086 | .074 |
|  | [.070, .313] | [.121, .386] | [-.028, .264] | [.025, .310] | [.061, .337] | [.054, .391] | [-.128, .212] | [-.162, .174] | [.057, .349] |
|  | (.002) | (<.001) | (.112) | (.021) | (.005) | (.010) | (.627) | (.945) | (.006) |
| 50- | .186 | .139 | .087 | .101 | .089 | .155 | -.098 | .122 | .219 |
|  | .063 | .064 | .069 | .071 | .068 | .080 | .080 | .081 | .069 |
|  | [.063, .309] | [.014, .265] | [-.048, .221] | [-.037, .240] | [-.045, .223] | [-.002, .313] | [-.254, .059] | [-.036, .280] | [.083, .354] |
|  | (.003) | (.030) | (.206) | (.151) | (.193) | (.053) | (.221) | (.131) | (.002) |
| 70- | .253 | -.026 | .124 | .312 | .048 | -.068 | .145 | .252 | .271 |
|  | .074 | .086 | .088 | .083 | .083 | .098 | .103 | .102 | .086 |
|  | [.108, .399] | [-.195, .143] | [-.049, .297] | [.149, .476] | [-.114, .211] | [-.261, .124] | [-.057, .347] | [.052, .451] | [.103, .439] |
|  | (.001) | (.765) | (.161) | (<.001) | (.561) | (.488) | (.159) | (.013) | (.002) |
| gender | .036 | -.006 | .154 | .190 | .091 | .356 | .130 | .170 | .087 |
|  | .043 | .048 | .051 | .051 | .048 | .058 | .061 | .061 | .051 |
|  | [-.048, .120] | [-.099, .088] | [.055, .253] | [.089, .290] | [-.002, .185] | [.243, .470] | [.011, .250] | [.051, .289] | [-.014, .188] |
|  | (.397) | (.903) | (.002) | (<.001) | (.055) | (<.001) | (.032) | (.005) | (.091) |
| white | .146 | .185 | .056 | .028 | .194 | -.070 | -.223 | .272 | -.006 |
|  | .051 | .055 | .059 | .059 | .057 | .068 | .071 | .073 | .061 |
|  | [.047, .246] | [.077, .292] | [-.060, .171] | [-.087, .143] | [.082, .307] | [-.202, .063] | [-.361, -.084] | [.128, .415] | [-.126, .113] |
|  | (.004) | (.001) | (.346) | (.632) | (.001) | (.305) | (.002) | (<.001) | (.916) |
| married | -.023 | -.069 | -.055 | .020 | -.034 | -.046 | .108 | -.125 | -.069 |
|  | .048 | .052 | .053 | .055 | .052 | .063 | .065 | .066 | .055 |
|  | [-.117, .070] | [-.171, .032] | [-.158, .049] | [-.087, .127] | [-.136, .068] | [-.171, .078] | [-.020, .236] | [-.256, .005] | [-.177, .039] |
|  | (.624) | (.181) | (.301) | (.714) | (.514) | (.465) | (.099) | (.059) | (.208) |
| High school graduate | -.185 | -.352 | -.218 | -.027 | -.304 | -.278 | -.246 | .019 | -.368 |
|  | .113 | .120 | .142 | .168 | .138 | .160 | .178 | .200 | .126 |
|  | [-.406, .036] | [-.587, -.117] | [-.497, .061] | [-.357, .303] | [-.574, -.034] | [-.592, .035] | [-.595, .104] | [-.374, .412] | [-.615, -.121] |
|  | (.101) | (.003) | (.125) | (.871) | (.027) | (.082) | (.168) | (.923) | (.003) |
| Some college | -.156 | -.257 | -.284 | -.122 | -.250 | -.450 | -.529 | .234 | -.370 |
|  | .118 | .125 | .147 | .173 | .145 | .168 | .183 | .202 | .131 |
|  | [-.388, .076] | [-.502, -.012] | [-.572, .005] | [-.462, .218] | [-.535, .035] | [-.779, -.121] | [-.889, -.170] | [-.163, .630] | [-.628, -.113] |
|  | (.187) | (.040) | (.054) | (.481) | (.086) | (.007) | (.004) | (.248) | (.005) |
| 2-year | -.101 | -.234 | -.298 | -.084 | -.244 | -.218 | -.408 | .166 | -.304 |
|  | .123 | .130 | .156 | .179 | .149 | .175 | .192 | .213 | .139 |
|  | [-.342, .140] | [-.489, .021] | [-.603, .007] | [-.435, .268] | [-.537, .048] | [-.560, .125] | [-.784, -.032] | [-.252, .583] | [-.576, -.032] |
|  | (.411) | (.072) | (.055) | (.641) | (.102) | (.213) | (.034) | (.437) | (.029) |
| 4-year | -.250 | -.335 | -.338 | -.142 | -.355 | -.406 | -.493 | .192 | -.437 |
|  | .119 | .126 | .148 | .174 | .144 | .168 | .186 | .206 | .135 |
|  | [-.483, -.016] | [-.581, -.089] | [-.627, -.049] | [-.484, .199] | [-.638, -.072] | [-.735, -.077] | [-.857, -.129] | [-.212, .597] | [-.701, -.172] |
|  | (.036) | (.008) | (.022) | (.414) | (.014) | (.016) | (.008) | (.351) | (.001) |
| Post-grad | -.237 | -.318 | -.485 | -.187 | -.426 | -.570 | -.513 | .189 | -.513 |
|  | .125 | .135 | .159 | .180 | .153 | .177 | .196 | .216 | .144 |
|  | [-.483, .009] | [-.582, -.054] | [-.797, -.173] | [-.540, .166] | [-.726, -.125] | [-.917, -.223] | [-.898, -.128] | [-.234, .613] | [-.795, -.230] |
|  | (.059) | (.018) | (.002) | (.299) | (.006) | (.001) | (.009) | (.381) | (<.001) |
| income=2 | .005 | .027 | .126 | -.079 | -.017 | -.188 | .045 | -.066 | .037 |
|  | .148 | .152 | .153 | .165 | .155 | .188 | .174 | .187 | .150 |
|  | [-.285, .294] | [-.272, .325] | [-.174, .425] | [-.403, .245] | [-.321, .287] | [-.556, .180] | [-.297, .387] | [-.432, .300] | [-.257, .331] |
|  | (.975) | (.860) | (.410) | (.631) | (.913) | (.317) | (.798) | (.724) | (.805) |
| income=3 | .136 | .164 | .234 | .103 | .027 | -.111 | -.074 | .021 | .278 |
|  | .140 | .145 | .153 | .157 | .151 | .178 | .179 | .188 | .146 |
|  | [-.139, .411] | [-.121, .449] | [-.066, .535] | [-.204, .411] | [-.270, .325] | [-.460, .238] | [-.426, .277] | [-.348, .390] | [-.009, .566] |
|  | (.332) | (.259) | (.126) | (.510) | (.856) | (.533) | (.678) | (.910) | (.058) |
| income=4 | .169 | .242 | .115 | .112 | .043 | -.178 | .147 | .166 | .010 |
|  | .139 | .145 | .161 | .155 | .150 | .180 | .168 | .190 | .154 |
|  | [-.103, .441] | [-.042, .526] | [-.200, .431] | [-.193, .417] | [-.250, .337] | [-.531, .174] | [-.183, .477] | [-.207, .539] | [-.292, .312] |
|  | (.224) | (.095) | (.473) | (.473) | (.772) | (.322) | (.382) | (.383) | (.949) |
| income=5 | .176 | .207 | .050 | .057 | .065 | -.265 | -.172 | .121 | .016 |
|  | .145 | .142 | .161 | .161 | .150 | .180 | .172 | .185 | .154 |
|  | [-.107, .460] | [-.071, .484] | [-.265, .365] | [-.258, .373] | [-.229, .358] | [-.618, .089] | [-.510, .166] | [-.242, .484] | [-.285, .317] |
|  | (.223) | (.145) | (.754) | (.722) | (.665) | (.143) | (.318) | (.513) | (.917) |
| income=6 | .188 | .099 | .210 | .210 | .105 | -.120 | .182 | .261 | .178 |
|  | .141 | .146 | .152 | .155 | .151 | .181 | .168 | .180 | .150 |
|  | [-.088, .464] | [-.187, .385] | [-.089, .508] | [-.094, .514] | [-.191, .401] | [-.476, .235] | [-.148, .512] | [-.091, .613] | [-.117, .473] |
|  | (.183) | (.498) | (.169) | (.175) | (.488) | (.506) | (.280) | (.146) | (.237) |
| income=7 | .197 | -.063 | .388 | .302 | .095 | .032 | .132 | .346 | .078 |
|  | .148 | .160 | .155 | .162 | .155 | .185 | .178 | .196 | .164 |
|  | [-.094, .488] | [-.377, .252] | [.084, .691] | [-.016, .621] | [-.210, .399] | [-.331, .394] | [-.217, .481] | [-.038, .731] | [-.244, .399] |
|  | (.184) | (.696) | (.012) | (.063) | (.542) | (.864) | (.457) | (.077) | (.636) |
| income=8 | .156 | .186 | .170 | .073 | .207 | -.009 | -.034 | .079 | .115 |
|  | .144 | .153 | .159 | .167 | .151 | .187 | .185 | .193 | .156 |
|  | [-.127, .438] | [-.113, .486] | [-.143, .482] | [-.254, .400] | [-.089, .502] | [-.375, .356] | [-.397, .328] | [-.299, .457] | [-.191, .421] |
|  | (.280) | (.222) | (.287) | (.660) | (.170) | (.960) | (.853) | (.683) | (.463) |
| income=9 | .094 | .004 | .256 | .138 | .155 | -.103 | -.108 | .359 | -.031 |
|  | .145 | .154 | .161 | .163 | .147 | .179 | .175 | .184 | .158 |
|  | [-.192, .379] | [-.297, .306] | [-.060, .572] | [-.181, .457] | [-.134, .444] | [-.455, .248] | [-.450, .235] | [-.002, .719] | [-.342, .279] |
|  | (.520) | (.977) | (.113) | (.397) | (.293) | (.564) | (.538) | (.052) | (.843) |
| income=10 | .161 | .061 | .221 | .267 | .030 | -.109 | -.130 | .396 | .032 |
|  | .147 | .152 | .160 | .163 | .157 | .189 | .182 | .193 | .165 |
|  | [-.128, .450] | [-.237, .358] | [-.093, .534] | [-.053, .587] | [-.277, .337] | [-.480, .262] | [-.487, .228] | [.016, .775] | [-.291, .356] |
|  | (.275) | (.689) | (.167) | (.102) | (.849) | (.565) | (.477) | (.041) | (.844) |
| income=11 | .217 | -.049 | .290 | .349 | .089 | -.035 | -.054 | .296 | -.001 |
|  | .143 | .162 | .165 | .163 | .159 | .191 | .188 | .192 | .167 |
|  | [-.063, .497] | [-.367, .269] | [-.034, .614] | [.028, .669] | [-.223, .400] | [-.410, .340] | [-.423, .314] | [-.081, .673] | [-.328, .327] |
|  | (.129) | (.762) | (.079) | (.033) | (.577) | (.855) | (.772) | (.124) | (.998) |
| income=12 | .049 | -.110 | .141 | .120 | .031 | -.020 | -.221 | .461 | .066 |
|  | .155 | .167 | .179 | .183 | .164 | .206 | .202 | .211 | .176 |
|  | [-.255, .354] | [-.438, .217] | [-.211, .492] | [-.238, .478] | [-.290, .353] | [-.423, .383] | [-.616, .174] | [.048, .875] | [-.279, .412] |
|  | (.750) | (.508) | (.433) | (.511) | (.848) | (.922) | (.273) | (.029) | (.706) |
| income=13 | .393 | .124 | .306 | .558 | .113 | -.182 | .097 | .489 | .155 |
|  | .172 | .207 | .245 | .218 | .227 | .290 | .293 | .272 | .230 |
|  | [.056, .729] | [-.283, .531] | [-.174, .786] | [.130, .986] | [-.333, .559] | [-.751, .387] | [-.478, .672] | [-.045,1.022] | [-.296, .606] |
|  | (.022) | (.550) | (.211) | (.011) | (.619) | (.530) | (.740) | (.073) | (.502) |
| income=14 | .068 | .058 | -.021 | .088 | .072 | -.152 | -.242 | .526 | -.160 |
|  | .235 | .247 | .279 | .270 | .243 | .277 | .313 | .272 | .277 |
|  | [-.393, .529] | [-.427, .543] | [-.568, .526] | [-.441, .617] | [-.404, .548] | [-.694, .391] | [-.856, .371] | [-.007,1.060] | [-.703, .382] |
|  | (.773) | (.815) | (.939) | (.743) | (.766) | (.584) | (.439) | (.053) | (.562) |
| income=15 | .711 | .788 | .125 | .422 | .581 | .197 | 1.427 | 1.046 | 1.182 |
|  | .262 | .333 | .600 | .706 | .149 | .464 | .337 | .200 | .180 |
|  | [.196,1.225] | [.136,1.441] | [-1.051,1.301] | [-.964,1.807] | [.289, .873] | [-.714,1.108] | [.767,2.087] | [.654,1.438] | [.829,1.534] |
|  | (.007) | (.018) | (.835) | (.550) | (<.001) | (.671) | (<.001) | (<.001) | (<.001) |
| income=16 | .238 | .350 | .406 | .510 | .495 | .099 | -.025 | .204 | .228 |
|  | .216 | .262 | .336 | .285 | .211 | .404 | .442 | .432 | .343 |
|  | [-.186, .663] | [-.164, .863] | [-.254,1.065] | [-.049,1.069] | [.081, .910] | [-.693, .892] | [-.892, .842] | [-.643,1.051] | [-.444, .900] |
|  | (.270) | (.182) | (.228) | (.074) | (.019) | (.805) | (.956) | (.636) | (.506) |
| income=17 | .090 | .044 | .073 | .070 | -.008 | -.083 | -.349 | .187 | .095 |
|  | .132 | .139 | .143 | .146 | .139 | .165 | .159 | .167 | .140 |
|  | [-.170, .350] | [-.228, .316] | [-.208, .354] | [-.216, .356] | [-.281, .265] | [-.406, .240] | [-.661, -.037] | [-.140, .515] | [-.180, .370] |
|  | (.496) | (.752) | (.612) | (.633) | (.955) | (.614) | (.028) | (.263) | (.499) |
| Rural Code=2 | -.045 | -.020 | -.052 | .025 | .038 | -.030 | -.102 | .078 | -.014 |
|  | .060 | .063 | .068 | .068 | .063 | .080 | .081 | .080 | .069 |
|  | [-.163, .073] | [-.143, .103] | [-.186, .081] | [-.108, .158] | [-.085, .161] | [-.187, .126] | [-.260, .057] | [-.078, .234] | [-.149, .122] |
|  | (.452) | (.750) | (.441) | (.714) | (.544) | (.702) | (.209) | (.324) | (.842) |
| Rural Code=3 | -.178 | -.494 | -.378 | -.153 | -.207 | -.032 | -.240 | .007 | -.357 |
|  | .121 | .129 | .132 | .131 | .132 | .131 | .145 | .140 | .116 |
|  | [-.415, .059] | [-.746, -.241] | [-.636, -.120] | [-.410, .104] | [-.465, .051] | [-.290, .226] | [-.524, .044] | [-.268, .282] | [-.585, -.129] |
|  | (.141) | (<.001) | (.004) | (.243) | (.115) | (.808) | (.097) | (.960) | (.002) |
| Rural Code=4 | -.599 | .010 | -.408 | -.602 | -.033 | -.206 | .332 | .026 | -.473 |
|  | .377 | .240 | .374 | .288 | .252 | .303 | .298 | .310 | .287 |
|  | [-1.338, .139] | [-.461, .481] | [-1.141, .326] | [-1.166, -.037] | [-.528, .461] | [-.801, .389] | [-.252, .917] | [-.583, .635] | [-1.037, .090] |
|  | (.112) | (.966) | (.276) | (.037) | (.895) | (.497) | (.265) | (.933) | (.100) |
| Rural Code=6 | 1.016 | .041 | .966 | -.872 | 1.158 | 1.147 | -2.229 | -.533 | -1.062 |
|  | .277 | .971 | .267 | 1.088 | .304 | .639 | .230 | 1.259 | .631 |
|  | [.473,1.558] | [-1.864,1.945] | [.443,1.489] | [-3.006,1.263] | [.563,1.753] | [-.106,2.400] | [-2.681, -1.778] | [-3.003,1.936] | [-2.299, .176] |
|  | (<.001) | (.967) | (<.001) | (.423) | (<.001) | (.073) | (<.001) | (.672) | (.093) |
| Arizona | -.286 | -.133 | .138 | -.324 | -.063 | -.265 | -.332 | -.013 | -.158 |
|  | .190 | .250 | .268 | .230 | .250 | .282 | .267 | .367 | .298 |
|  | [-.658, .086] | [-.624, .358] | [-.388, .664] | [-.776, .127] | [-.553, .428] | [-.818, .289] | [-.855, .191] | [-.732, .706] | [-.742, .427] |
|  | (.132) | (.595) | (.607) | (.159) | (.802) | (.349) | (.214) | (.971) | (.597) |
| Arkansas | -.390 | -.133 | -.479 | -.364 | -.339 | -.474 | .481 | .059 | -.390 |
|  | .237 | .327 | .369 | .285 | .336 | .386 | .404 | .468 | .439 |
|  | [-.855, .076] | [-.775, .509] | [-1.204, .245] | [-.923, .194] | [-.999, .320] | [-1.231, .283] | [-.312,1.274] | [-.859, .978] | [-1.252, .471] |
|  | (.101) | (.685) | (.194) | (.201) | (.313) | (.220) | (.234) | (.899) | (.375) |
| California | -.142 | .135 | .202 | -.173 | .084 | -.139 | .154 | .127 | .010 |
|  | .150 | .217 | .243 | .197 | .218 | .253 | .237 | .323 | .261 |
|  | [-.436, .152] | [-.290, .560] | [-.274, .677] | [-.559, .212] | [-.342, .511] | [-.634, .356] | [-.311, .620] | [-.507, .760] | [-.503, .523] |
|  | (.343) | (.534) | (.406) | (.379) | (.698) | (.582) | (.516) | (.695) | (.970) |
| Colorado | -.031 | -.196 | .225 | .049 | .084 | -.210 | .194 | .256 | .120 |
|  | .201 | .288 | .304 | .260 | .282 | .318 | .319 | .378 | .299 |
|  | [-.426, .364] | [-.761, .369] | [-.372, .822] | [-.462, .559] | [-.469, .637] | [-.833, .414] | [-.431, .818] | [-.486, .998] | [-.467, .708] |
|  | (.878) | (.496) | (.459) | (.852) | (.766) | (.510) | (.543) | (.499) | (.688) |
| Connecticut | .088 | .302 | .595 | .333 | .130 | .291 | .211 | .043 | .410 |
|  | .222 | .235 | .261 | .233 | .255 | .319 | .319 | .403 | .304 |
|  | [-.348, .524] | [-.160, .763] | [.084,1.106] | [-.125, .791] | [-.370, .630] | [-.334, .917] | [-.415, .837] | [-.746, .833] | [-.186,1.005] |
|  | (.691) | (.200) | (.023) | (.154) | (.611) | (.361) | (.508) | (.914) | (.178) |
| Delaware | .102 | .261 | -.144 | .276 | .130 | -.315 | .163 | -.375 | -.314 |
|  | .160 | .282 | .432 | .313 | .266 | .723 | .455 | .757 | .470 |
|  | [-.213, .416] | [-.291, .813] | [-.991, .703] | [-.337, .889] | [-.391, .651] | [-1.733,1.102] | [-.730,1.056] | [-1.860,1.110] | [-1.235, .607] |
|  | (.525) | (.354) | (.739) | (.377) | (.625) | (.662) | (.721) | (.620) | (.504) |
| Florida | -.176 | -.106 | .259 | -.228 | -.057 | -.054 | .257 | .178 | .202 |
|  | .155 | .225 | .247 | .202 | .225 | .257 | .241 | .325 | .263 |
|  | [-.479, .128] | [-.547, .335] | [-.225, .743] | [-.624, .167] | [-.499, .384] | [-.559, .451] | [-.214, .729] | [-.461, .816] | [-.313, .717] |
|  | (.257) | (.638) | (.295) | (.258) | (.799) | (.833) | (.285) | (.585) | (.442) |
| Georgia | -.169 | -.211 | .074 | -.410 | .138 | -.145 | .078 | .004 | .056 |
|  | .180 | .256 | .270 | .234 | .241 | .273 | .273 | .349 | .279 |
|  | [-.522, .185] | [-.713, .291] | [-.456, .604] | [-.869, .049] | [-.335, .612] | [-.680, .391] | [-.456, .613] | [-.680, .687] | [-.490, .603] |
|  | (.349) | (.410) | (.784) | (.080) | (.567) | (.596) | (.774) | (.991) | (.840) |
| Idaho | -.389 | -.182 | .386 | -.588 | -.410 | -.319 | -.486 | .047 | -.091 |
|  | .415 | .447 | .359 | .449 | .447 | .421 | .425 | .527 | .450 |
|  | [-1.203, .424] | [-1.058, .694] | [-.318,1.089] | [-1.470, .293] | [-1.287, .467] | [-1.144, .506] | [-1.320, .348] | [-.986,1.080] | [-.973, .791] |
|  | (.348) | (.684) | (.282) | (.191) | (.359) | (.448) | (.253) | (.928) | (.840) |
| Illinois | -.091 | -.031 | .246 | .001 | .203 | -.075 | .257 | .197 | .219 |
|  | .159 | .236 | .255 | .209 | .234 | .269 | .259 | .339 | .269 |
|  | [-.403, .221] | [-.495, .432] | [-.255, .747] | [-.409, .411] | [-.256, .661] | [-.602, .452] | [-.251, .766] | [-.469, .862] | [-.309, .747] |
|  | (.569) | (.895) | (.335) | (.996) | (.386) | (.781) | (.322) | (.563) | (.417) |
| Indiana | -.090 | .210 | .631 | .364 | .013 | -.147 | .284 | .673 | .242 |
|  | .222 | .268 | .270 | .230 | .299 | .336 | .316 | .369 | .300 |
|  | [-.525, .345] | [-.317, .736] | [.100,1.161] | [-.086, .814] | [-.573, .598] | [-.806, .513] | [-.335, .904] | [-.051,1.397] | [-.346, .830] |
|  | (.685) | (.435) | (.020) | (.113) | (.966) | (.663) | (.368) | (.068) | (.419) |
| Iowa | -.273 | -.147 | .172 | -.196 | -.105 | .341 | .249 | .457 | -.141 |
|  | .280 | .351 | .390 | .324 | .341 | .359 | .412 | .397 | .398 |
|  | [-.821, .275] | [-.837, .542] | [-.593, .937] | [-.832, .439] | [-.773, .564] | [-.363,1.044] | [-.558,1.057] | [-.321,1.235] | [-.921, .640] |
|  | (.329) | (.675) | (.659) | (.545) | (.759) | (.343) | (.545) | (.249) | (.724) |
| Kansas | .169 | .561 | .614 | .396 | .782 | .507 | .786 | 1.224 | .067 |
|  | .227 | .269 | .355 | .353 | .236 | .408 | .541 | .369 | .591 |
|  | [-.275, .614] | [.034,1.087] | [-.082,1.309] | [-.296,1.089] | [.319,1.244] | [-.294,1.307] | [-.275,1.848] | [.500,1.949] | [-1.092,1.227] |
|  | (.455) | (.037) | (.084) | (.262) | (.001) | (.215) | (.147) | (.001) | (.909) |
| Kentucky | -.701 | -.082 | -.247 | -1.481 | -.119 | .139 | -.697 | -.743 | -.288 |
|  | .446 | .456 | .438 | .609 | .377 | .479 | .445 | .615 | .319 |
|  | [-1.576, .174] | [-.977, .812] | [-1.107, .613] | [-2.675, -.287] | [-.858, .620] | [-.800,1.078] | [-1.570, .175] | [-1.949, .462] | [-.914, .338] |
|  | (.116) | (.857) | (.573) | (.015) | (.752) | (.772) | (.117) | (.227) | (.367) |
| Louisiana | -.276 | .179 | .237 | -.214 | .037 | -.184 | .236 | .204 | .125 |
|  | .274 | .285 | .348 | .295 | .316 | .390 | .331 | .413 | .335 |
|  | [-.813, .262] | [-.380, .738] | [-.446, .920] | [-.793, .365] | [-.582, .657] | [-.949, .580] | [-.414, .886] | [-.606,1.014] | [-.532, .781] |
|  | (.315) | (.531) | (.496) | (.468) | (.906) | (.636) | (.477) | (.621) | (.710) |
| Maine | -.054 | .289 | .608 | -.058 | .589 | -.229 | .367 | -.334 | .513 |
|  | .384 | .384 | .294 | .620 | .278 | .673 | .616 | .909 | .635 |
|  | [-.807, .699] | [-.464,1.042] | [.032,1.183] | [-1.275,1.158] | [.044,1.133] | [-1.549,1.090] | [-.842,1.576] | [-2.118,1.449] | [-.732,1.758] |
|  | (.888) | (.452) | (.039) | (.925) | (.034) | (.733) | (.552) | (.713) | (.419) |
| Maryland | -.113 | .000 | .034 | -.298 | .204 | .007 | .196 | .024 | .127 |
|  | .194 | .263 | .331 | .279 | .270 | .324 | .347 | .384 | .341 |
|  | [-.493, .267] | [-.515, .515] | [-.615, .683] | [-.845, .250] | [-.325, .733] | [-.627, .642] | [-.484, .876] | [-.730, .777] | [-.541, .796] |
|  | (.561) | (.999) | (.918) | (.287) | (.450) | (.982) | (.572) | (.951) | (.709) |
| Massachusetts | -.077 | .091 | .067 | -.217 | .124 | .100 | .093 | .384 | -.170 |
|  | .177 | .248 | .277 | .245 | .238 | .288 | .268 | .339 | .303 |
|  | [-.425, .271] | [-.394, .577] | [-.477, .610] | [-.698, .264] | [-.343, .592] | [-.466, .665] | [-.433, .620] | [-.281,1.049] | [-.764, .425] |
|  | (.664) | (.712) | (.810) | (.376) | (.602) | (.730) | (.728) | (.258) | (.576) |
| Michigan | -.145 | .111 | .371 | -.271 | .007 | -.108 | .189 | .261 | .077 |
|  | .177 | .232 | .255 | .225 | .242 | .270 | .265 | .336 | .277 |
|  | [-.493, .203] | [-.343, .566] | [-.128, .871] | [-.712, .170] | [-.468, .482] | [-.638, .422] | [-.331, .710] | [-.397, .919] | [-.467, .620] |
|  | (.413) | (.631) | (.145) | (.228) | (.977) | (.689) | (.476) | (.437) | (.782) |
| Minnesota | -.355 | -.158 | .391 | -.134 | -.173 | -.102 | .083 | .304 | .220 |
|  | .207 | .281 | .296 | .238 | .262 | .309 | .300 | .375 | .309 |
|  | [-.761, .051] | [-.708, .393] | [-.191, .972] | [-.600, .332] | [-.688, .342] | [-.709, .504] | [-.506, .672] | [-.431,1.038] | [-.387, .827] |
|  | (.086) | (.574) | (.188) | (.572) | (.510) | (.740) | (.782) | (.418) | (.478) |
| Mississippi | -.058 | .066 | -.013 | -.078 | -.021 | -.106 | .230 | .153 | .390 |
|  | .346 | .344 | .486 | .313 | .380 | .422 | .468 | .614 | .340 |
|  | [-.736, .619] | [-.609, .741] | [-.966, .939] | [-.692, .536] | [-.766, .724] | [-.933, .722] | [-.687,1.148] | [-1.051,1.356] | [-.277,1.058] |
|  | (.866) | (.849) | (.978) | (.804) | (.956) | (.802) | (.623) | (.804) | (.252) |
| Missouri | -.171 | .043 | .197 | -.195 | .095 | .097 | .028 | .270 | .021 |
|  | .204 | .254 | .287 | .260 | .257 | .309 | .297 | .365 | .310 |
|  | [-.572, .229] | [-.456, .541] | [-.366, .761] | [-.706, .316] | [-.409, .600] | [-.509, .703] | [-.554, .609] | [-.446, .986] | [-.587, .630] |
|  | (.402) | (.867) | (.492) | (.453) | (.712) | (.754) | (.926) | (.460) | (.945) |
| Montana | .439 | 1.103 | 1.294 | .845 | .889 | -.968 | 2.003 | 1.118 | 1.056 |
|  | .197 | .246 | .259 | .204 | .238 | 1.115 | .391 | .336 | .394 |
|  | [.054, .825] | [.620,1.586] | [.787,1.801] | [.444,1.245] | [.423,1.355] | [-3.155,1.219] | [1.237,2.770] | [.459,1.777] | [.283,1.830] |
|  | (.026) | (<.001) | (<.001) | (<.001) | (<.001) | (.385) | (<.001) | (.001) | (.007) |
| Nebraska | -.150 | .192 | -.737 | -.690 | .098 | .247 | .825 | -.832 | -.405 |
|  | .347 | .344 | .544 | .494 | .366 | .481 | .608 | .556 | .431 |
|  | [-.830, .530] | [-.482, .866] | [-1.804, .329] | [-1.660, .280] | [-.619, .815] | [-.696,1.190] | [-.367,2.017] | [-1.923, .259] | [-1.251, .440] |
|  | (.666) | (.577) | (.175) | (.163) | (.789) | (.608) | (.175) | (.135) | (.347) |
| Nevada | -.088 | .070 | .240 | -.130 | -.071 | -.252 | -.129 | .218 | .172 |
|  | .184 | .254 | .281 | .233 | .282 | .312 | .287 | .368 | .287 |
|  | [-.449, .272] | [-.428, .568] | [-.310, .791] | [-.587, .326] | [-.624, .483] | [-.865, .360] | [-.692, .434] | [-.503, .939] | [-.390, .734] |
|  | (.631) | (.783) | (.392) | (.575) | (.802) | (.419) | (.652) | (.553) | (.548) |
| New Hampshire | -.051 | .070 | -.081 | -.368 | .134 | -.275 | .178 | -.316 | .507 |
|  | .273 | .371 | .385 | .364 | .310 | .378 | .423 | .450 | .328 |
|  | [-.587, .485] | [-.657, .797] | [-.837, .675] | [-1.082, .345] | [-.475, .742] | [-1.016, .466] | [-.651,1.007] | [-1.199, .567] | [-.136,1.151] |
|  | (.852) | (.851) | (.833) | (.311) | (.667) | (.467) | (.673) | (.483) | (.122) |
| New Jersey | -.147 | .231 | .301 | -.153 | .120 | .178 | .425 | .102 | .162 |
|  | .185 | .241 | .269 | .224 | .248 | .277 | .267 | .345 | .285 |
|  | [-.509, .216] | [-.241, .704] | [-.228, .829] | [-.593, .286] | [-.365, .606] | [-.365, .722] | [-.099, .950] | [-.574, .778] | [-.397, .721] |
|  | (.428) | (.337) | (.265) | (.494) | (.627) | (.520) | (.112) | (.767) | (.569) |
| New Mexico | .125 | -.121 | .390 | -.262 | -.227 | -.133 | .191 | .361 | .106 |
|  | .237 | .374 | .360 | .374 | .353 | .389 | .429 | .421 | .370 |
|  | [-.339, .589] | [-.856, .613] | [-.317,1.097] | [-.995, .470] | [-.920, .466] | [-.895, .629] | [-.650,1.031] | [-.465,1.186] | [-.620, .831] |
|  | (.597) | (.746) | (.279) | (.483) | (.521) | (.733) | (.657) | (.392) | (.775) |
| New York | -.333 | -.002 | .216 | -.209 | -.018 | .013 | .242 | -.118 | .150 |
|  | .164 | .224 | .253 | .211 | .227 | .264 | .250 | .338 | .273 |
|  | [-.654, -.011] | [-.440, .437] | [-.280, .711] | [-.624, .205] | [-.464, .428] | [-.505, .531] | [-.248, .731] | [-.781, .545] | [-.386, .686] |
|  | (.042) | (.994) | (.394) | (.322) | (.936) | (.961) | (.333) | (.726) | (.584) |
| North Carolina | -.233 | .127 | .207 | .051 | .082 | -.202 | .286 | .020 | -.023 |
|  | .196 | .245 | .286 | .227 | .255 | .298 | .273 | .372 | .300 |
|  | [-.616, .151] | [-.353, .607] | [-.353, .767] | [-.395, .496] | [-.418, .582] | [-.787, .382] | [-.249, .822] | [-.710, .749] | [-.611, .565] |
|  | (.235) | (.603) | (.468) | (.823) | (.748) | (.498) | (.294) | (.958) | (.940) |
| North Dakota | -.386 | .684 | .470 | -.292 | .270 | .224 | 1.041 | -.266 | -.301 |
|  | .512 | .334 | .428 | .370 | .346 | .572 | .437 | .524 | .447 |
|  | [-1.390, .617] | [.029,1.339] | [-.370,1.311] | [-1.017, .434] | [-.409, .949] | [-.899,1.346] | [.184,1.899] | [-1.294, .763] | [-1.177, .575] |
|  | (.450) | (.041) | (.273) | (.430) | (.436) | (.696) | (.017) | (.612) | (.501) |
| Ohio | -.159 | .078 | .215 | -.160 | .093 | -.105 | -.099 | .253 | .285 |
|  | .172 | .234 | .274 | .231 | .235 | .285 | .269 | .349 | .280 |
|  | [-.497, .179] | [-.380, .537] | [-.322, .752] | [-.614, .293] | [-.368, .555] | [-.664, .455] | [-.626, .429] | [-.430, .937] | [-.263, .834] |
|  | (.357) | (.738) | (.433) | (.488) | (.691) | (.713) | (.714) | (.468) | (.308) |
| Oklahoma | -.261 | .039 | .382 | .218 | .025 | .544 | .754 | .115 | -.454 |
|  | .284 | .336 | .386 | .276 | .311 | .383 | .403 | .553 | .532 |
|  | [-.818, .296] | [-.620, .697] | [-.375,1.139] | [-.324, .760] | [-.584, .635] | [-.208,1.295] | [-.037,1.544] | [-.970,1.200] | [-1.498, .590] |
|  | (.358) | (.908) | (.322) | (.429) | (.935) | (.156) | (.062) | (.835) | (.394) |
| Oregon | -.004 | .211 | -.216 | -.316 | .024 | -.150 | -.145 | .206 | -.278 |
|  | .172 | .241 | .278 | .231 | .254 | .289 | .281 | .353 | .296 |
|  | [-.341, .334] | [-.261, .683] | [-.762, .330] | [-.769, .136] | [-.475, .522] | [-.717, .416] | [-.696, .407] | [-.486, .898] | [-.860, .304] |
|  | (.983) | (.381) | (.437) | (.170) | (.926) | (.602) | (.607) | (.560) | (.349) |
| Pennsylvania | -.187 | .026 | .169 | -.115 | .100 | .022 | .149 | .208 | .252 |
|  | .168 | .228 | .256 | .211 | .231 | .264 | .243 | .327 | .263 |
|  | [-.516, .143] | [-.420, .472] | [-.333, .672] | [-.528, .299] | [-.352, .552] | [-.495, .538] | [-.328, .627] | [-.432, .849] | [-.264, .769] |
|  | (.266) | (.909) | (.509) | (.586) | (.665) | (.935) | (.539) | (.524) | (.338) |
| Rhode Island | .332 | .404 | .496 | -.023 | .303 | 1.095 | .244 | -.236 | .179 |
|  | .161 | .355 | .452 | .523 | .343 | .322 | .579 | .706 | .449 |
|  | [.016, .648] | [-.292,1.100] | [-.390,1.382] | [-1.049,1.004] | [-.370, .976] | [.463,1.727] | [-.892,1.380] | [-1.621,1.149] | [-.702,1.059] |
|  | (.040) | (.255) | (.272) | (.966) | (.377) | (.001) | (.673) | (.738) | (.691) |
| South Carolina | -.417 | -.576 | .330 | .002 | -.339 | -.303 | -.458 | .279 | -.014 |
|  | .383 | .427 | .291 | .296 | .384 | .339 | .424 | .399 | .393 |
|  | [-1.167, .334] | [-1.414, .262] | [-.240, .901] | [-.579, .583] | [-1.092, .415] | [-.967, .362] | [-1.291, .374] | [-.503,1.062] | [-.784, .756] |
|  | (.276) | (.178) | (.256) | (.995) | (.378) | (.372) | (.280) | (.484) | (.971) |
| South Dakota | .360 | .318 | -.703 | .060 | .715 | -.419 | .972 | -.956 | .779 |
|  | .392 | .555 | .707 | .910 | .427 | .916 | .398 | .919 | .298 |
|  | [-.409,1.129] | [-.771,1.408] | [-2.088, .683] | [-1.724,1.845] | [-.123,1.554] | [-2.215,1.378] | [.192,1.753] | [-2.758, .847] | [.195,1.364] |
|  | (.359) | (.567) | (.320) | (.947) | (.094) | (.648) | (.015) | (.299) | (.009) |
| Tennessee | .084 | .415 | .329 | -.282 | .327 | -.230 | -.061 | .614 | -.052 |
|  | .190 | .270 | .298 | .287 | .249 | .317 | .325 | .374 | .331 |
|  | [-.290, .457] | [-.115, .944] | [-.255, .913] | [-.844, .280] | [-.161, .814] | [-.852, .392] | [-.699, .578] | [-.120,1.348] | [-.701, .597] |
|  | (.660) | (.125) | (.270) | (.325) | (.189) | (.469) | (.852) | (.101) | (.875) |
| Texas | -.223 | -.086 | .174 | -.162 | -.210 | -.228 | .135 | .030 | .152 |
|  | .160 | .229 | .251 | .206 | .230 | .262 | .243 | .334 | .265 |
|  | [-.537, .091] | [-.535, .363] | [-.319, .667] | [-.567, .242] | [-.661, .241] | [-.741, .286] | [-.343, .612] | [-.625, .685] | [-.369, .672] |
|  | (.164) | (.707) | (.489) | (.431) | (.362) | (.385) | (.580) | (.928) | (.568) |
| Utah | -.292 | -.191 | .021 | -.136 | -.162 | -.562 | .152 | .252 | -.280 |
|  | .255 | .317 | .321 | .317 | .315 | .449 | .371 | .385 | .351 |
|  | [-.791, .208] | [-.812, .430] | [-.608, .651] | [-.758, .486] | [-.780, .456] | [-1.442, .318] | [-.575, .880] | [-.504,1.008] | [-.969, .410] |
|  | (.252) | (.547) | (.948) | (.668) | (.608) | (.210) | (.682) | (.513) | (.426) |
| Vermont | .177 | -1.723 | -1.284 | -1.368 | -1.683 | -.940 | -.144 | -1.629 | -1.286 |
|  | .208 | .269 | .304 | .278 | .280 | .321 | .329 | .401 | .312 |
|  | [-.231, .585] | [-2.250, -1.196] | [-1.880, -.688] | [-1.913, -.824] | [-2.231, -1.135] | [-1.570, -.311] | [-.790, .502] | [-2.416, -.843] | [-1.897, -.674] |
|  | (.394) | (<.001) | (<.001) | (<.001) | (<.001) | (.003) | (.662) | (<.001) | (<.001) |
| Virginia | -.287 | .474 | .861 | .599 | .343 | -.190 | .561 | .842 | .805 |
|  | .654 | .413 | .329 | .253 | .428 | .598 | .312 | .395 | .495 |
|  | [-1.569, .995] | [-.337,1.285] | [.217,1.506] | [.102,1.096] | [-.496,1.181] | [-1.363, .983] | [-.051,1.173] | [.068,1.616] | [-.166,1.776] |
|  | (.660) | (.252) | (.009) | (.018) | (.423) | (.751) | (.072) | (.033) | (.104) |
| Washington | -.279 | -.202 | -.113 | -.373 | .133 | -.262 | -.160 | .274 | -.078 |
|  | .206 | .272 | .290 | .239 | .256 | .294 | .265 | .348 | .294 |
|  | [-.684, .126] | [-.737, .332] | [-.682, .456] | [-.843, .096] | [-.369, .636] | [-.839, .315] | [-.681, .360] | [-.409, .957] | [-.655, .499] |
|  | (.176) | (.458) | (.697) | (.119) | (.603) | (.373) | (.546) | (.431) | (.791) |
| West Virginia | -.898 | -.042 | 1.113 | .382 | .537 | -.064 | .500 | .003 | .719 |
|  | .505 | .439 | .416 | .389 | .412 | .479 | .757 | .855 | .406 |
|  | [-1.889, .092] | [-.902, .819] | [.297,1.929] | [-.382,1.146] | [-.272,1.345] | [-1.003, .874] | [-.985,1.985] | [-1.674,1.680] | [-.077,1.515] |
|  | (.075) | (.925) | (.008) | (.327) | (.193) | (.893) | (.509) | (.997) | (.077) |
| Wisconsin | -.329 | .016 | .162 | -.267 | -.162 | -.229 | .043 | .232 | .191 |
|  | .212 | .256 | .294 | .254 | .266 | .290 | .289 | .337 | .292 |
|  | [-.745, .087] | [-.486, .518] | [-.416, .739] | [-.765, .232] | [-.683, .359] | [-.797, .340] | [-.523, .609] | [-.429, .894] | [-.381, .763] |
|  | (.121) | (.950) | (.582) | (.294) | (.542) | (.430) | (.882) | (.491) | (.512) |
| Wyoming | .680 | 1.438 | 1.433 | .990 | .139 | .539 | 2.015 | -.468 | .524 |
|  | .232 | .340 | .287 | .230 | .893 | .762 | .285 | 1.505 | .777 |
|  | [.225,1.135] | [.771,2.104] | [.870,1.996] | [.539,1.440] | [-1.611,1.890] | [-.956,2.034] | [1.457,2.574] | [-3.420,2.484] | [-1.000,2.047] |
|  | (.003) | (<.001) | (<.001) | (<.001) | (.876) | (.479) | (<.001) | (.756) | (.500) |
| Observations | 1892 | 1895 | 1890 | 1895 | 1896 | 1895 | 1896 | 1895 | 1898 |
| r2_p |  |  |  |  |  |  |  |  |  |
| chi2 |  |  |  |  |  |  |  |  |  |
| df_m | 82 | 82 | 82 | 82 | 82 | 82 | 82 | 82 | 82 |
| p | 82 | 82 | 82 | 82 | 82 | 82 | 82 | 82 | 82 |

S34 Table. Regression Results, Public Policies, Adjusting for Local Policies.

|  | (1) | (2) | (3) | (4) | (5) | (6) | (7) | (8) | (9) | (10) |
| --- | --- | --- | --- | --- | --- | --- | --- | --- | --- | --- |
|  | increasetrade | importtaxes | lowinterest | notreliant | noairtravel | banentry | entrymexico | quarantine | rescueUS | delayelections |
| Republican | -.517 | .572 | .218 | .700 | .292 | .655 | .868 | .337 | .037 | .239 |
|  | .125 | .129 | .113 | .109 | .132 | .120 | .115 | .160 | .129 | .166 |
|  | [-.763, -.271] | [.320, .825] | [-.004, .441] | [.487, .913] | [.033, .551] | [.421, .890] | [.642,1.094] | [.024, .650] | [-.215, .290] | [-.087, .565] |
|  | (<.001) | (<.001) | (.054) | (<.001) | (.027) | (<.001) | (<.001) | (.035) | (.771) | (.151) |
| Other | -.408 | .285 | -.094 | .358 | .004 | .056 | .277 | -.023 | -.053 | .091 |
|  | .108 | .123 | .114 | .115 | .133 | .131 | .131 | .135 | .127 | .162 |
|  | [-.620, -.196] | [.043, .527] | [-.318, .129] | [.132, .583] | [-.258, .265] | [-.201, .312] | [.020, .533] | [-.287, .241] | [-.301, .196] | [-.228, .409] |
|  | (<.001) | (.021) | (.408) | (.002) | (.977) | (.671) | (.034) | (.863) | (.678) | (.577) |
| policies | .059 | .248 | -.085 | -.217 | -.027 | .003 | -.412 | -.224 | -.018 | -.113 |
|  | .217 | .251 | .232 | .240 | .263 | .254 | .260 | .268 | .248 | .331 |
|  | [-.367, .485] | [-.245, .740] | [-.540, .371] | [-.688, .253] | [-.543, .488] | [-.496, .502] | [-.922, .097] | [-.751, .302] | [-.503, .468] | [-.763, .537] |
|  | (.786) | (.324) | (.715) | (.365) | (.917) | (.991) | (.113) | (.404) | (.943) | (.733) |
| Republican # policies | -.141 | .403 | .045 | .070 | .051 | .055 | .371 | .720 | -.196 | .294 |
|  | .370 | .371 | .321 | .301 | .369 | .334 | .313 | .456 | .367 | .480 |
|  | [-.868, .585] | [-.324,1.130] | [-.585, .676] | [-.520, .659] | [-.674, .775] | [-.599, .710] | [-.242, .985] | [-.174,1.615] | [-.916, .524] | [-.646,1.235] |
|  | (.702) | (.277) | (.888) | (.817) | (.891) | (.868) | (.235) | (.115) | (.593) | (.539) |
| Other # policies | -.238 | -.220 | .036 | -.144 | .128 | .244 | .448 | .242 | -.377 | -.102 |
|  | .301 | .347 | .324 | .327 | .368 | .360 | .366 | .377 | .352 | .465 |
|  | [-.829, .353] | [-.902, .461] | [-.600, .671] | [-.784, .497] | [-.593, .849] | [-.462, .950] | [-.271,1.166] | [-.497, .982] | [-1.067, .312] | [-1.015, .811] |
|  | (.430) | (.526) | (.913) | (.660) | (.728) | (.498) | (.222) | (.520) | (.283) | (.827) |
| 35- | .018 | .158 | .264 | .269 | .244 | .154 | .179 | .094 | .121 | -.069 |
|  | .068 | .078 | .074 | .075 | .083 | .082 | .082 | .090 | .080 | .097 |
|  | [-.115, .151] | [.006, .311] | [.120, .409] | [.123, .415] | [.081, .406] | [-.006, .314] | [.018, .341] | [-.082, .270] | [-.036, .279] | [-.260, .121] |
|  | (.790) | (.042) | (<.001) | (<.001) | (.003) | (.059) | (.029) | (.296) | (.131) | (.473) |
| 50- | .017 | .229 | .274 | .489 | .215 | .365 | .465 | -.079 | .138 | -.404 |
|  | .066 | .072 | .068 | .069 | .078 | .074 | .076 | .084 | .073 | .092 |
|  | [-.113, .148] | [.089, .370] | [.141, .407] | [.354, .624] | [.063, .367] | [.219, .511] | [.316, .613] | [-.244, .086] | [-.005, .282] | [-.584, -.224] |
|  | (.794) | (.001) | (<.001) | (<.001) | (.006) | (<.001) | (<.001) | (.346) | (.059) | (<.001) |
| 70- | .112 | .066 | .291 | .472 | .105 | .239 | .420 | -.278 | .334 | -.279 |
|  | .085 | .090 | .084 | .089 | .098 | .099 | .094 | .101 | .094 | .117 |
|  | [-.054, .278] | [-.111, .243] | [.127, .456] | [.297, .648] | [-.087, .298] | [.045, .433] | [.236, .604] | [-.477, -.079] | [.150, .518] | [-.508, -.050] |
|  | (.185) | (.466) | (.001) | (<.001) | (.283) | (.016) | (<.001) | (.006) | (<.001) | (.017) |
| gender | -.159 | .122 | .030 | -.109 | .206 | .118 | -.012 | .038 | -.082 | .397 |
|  | .049 | .054 | .050 | .051 | .058 | .056 | .055 | .063 | .055 | .069 |
|  | [-.255, -.064] | [.015, .229] | [-.068, .128] | [-.208, -.010] | [.094, .319] | [.007, .229] | [-.120, .096] | [-.085, .161] | [-.191, .027] | [.263, .532] |
|  | (.001) | (.026) | (.544) | (.032) | (<.001) | (.037) | (.823) | (.543) | (.139) | (<.001) |
| white | -.019 | -.142 | -.049 | -.040 | -.051 | -.022 | -.060 | -.359 | .081 | -.090 |
|  | .057 | .065 | .059 | .060 | .066 | .067 | .066 | .075 | .065 | .079 |
|  | [-.131, .094] | [-.268, -.015] | [-.165, .066] | [-.157, .077] | [-.180, .078] | [-.154, .110] | [-.189, .069] | [-.506, -.212] | [-.047, .208] | [-.245, .066] |
|  | (.745) | (.028) | (.402) | (.500) | (.437) | (.741) | (.362) | (<.001) | (.214) | (.257) |
| married | -.044 | .084 | -.052 | -.008 | .042 | -.017 | .019 | .057 | -.079 | -.079 |
|  | .054 | .059 | .054 | .055 | .060 | .062 | .061 | .067 | .061 | .075 |
|  | [-.151, .062] | [-.032, .199] | [-.158, .054] | [-.117, .100] | [-.076, .161] | [-.138, .104] | [-.100, .138] | [-.074, .189] | [-.198, .040] | [-.225, .067] |
|  | (.418) | (.154) | (.335) | (.878) | (.484) | (.780) | (.750) | (.393) | (.195) | (.291) |
| High school graduate | -.478 | -.051 | -.062 | -.024 | .019 | -.264 | -.142 | -.602 | .116 | .070 |
|  | .143 | .148 | .158 | .158 | .160 | .150 | .149 | .187 | .160 | .191 |
|  | [-.758, -.198] | [-.341, .239] | [-.372, .248] | [-.334, .285] | [-.295, .333] | [-.558, .031] | [-.434, .150] | [-.968, -.236] | [-.199, .430] | [-.304, .445] |
|  | (.001) | (.730) | (.695) | (.877) | (.904) | (.079) | (.342) | (.001) | (.470) | (.712) |
| Some college | -.384 | -.193 | -.132 | -.073 | -.342 | -.458 | -.384 | -.894 | .162 | -.161 |
|  | .146 | .152 | .164 | .164 | .166 | .156 | .156 | .190 | .163 | .195 |
|  | [-.670, -.097] | [-.491, .105] | [-.454, .189] | [-.395, .248] | [-.667, -.017] | [-.763, -.152] | [-.690, -.077] | [-1.266, -.522] | [-.159, .482] | [-.544, .222] |
|  | (.009) | (.205) | (.419) | (.655) | (.039) | (.003) | (.014) | (<.001) | (.322) | (.410) |
| 2-year | -.373 | -.075 | -.064 | -.024 | -.205 | -.277 | -.250 | -.882 | .141 | .002 |
|  | .153 | .159 | .172 | .169 | .175 | .164 | .163 | .199 | .172 | .205 |
|  | [-.674, -.073] | [-.387, .236] | [-.401, .273] | [-.356, .307] | [-.547, .138] | [-.598, .043] | [-.569, .069] | [-1.273, -.491] | [-.195, .478] | [-.401, .405] |
|  | (.015) | (.636) | (.711) | (.885) | (.242) | (.090) | (.124) | (<.001) | (.410) | (.990) |
| 4-year | -.182 | -.293 | -.241 | -.270 | -.475 | -.665 | -.478 | -.986 | .134 | -.188 |
|  | .148 | .154 | .165 | .167 | .168 | .160 | .158 | .194 | .165 | .198 |
|  | [-.472, .109] | [-.595, .009] | [-.565, .082] | [-.597, .057] | [-.804, -.145] | [-.979, -.351] | [-.788, -.169] | [-1.365, -.606] | [-.188, .457] | [-.577, .201] |
|  | (.221) | (.057) | (.144) | (.105) | (.005) | (<.001) | (.002) | (<.001) | (.414) | (.343) |
| Post-grad | -.110 | -.423 | -.176 | -.439 | -.685 | -.583 | -.599 | -1.080 | .082 | -.345 |
|  | .153 | .163 | .170 | .177 | .179 | .169 | .171 | .204 | .175 | .211 |
|  | [-.410, .191] | [-.743, -.102] | [-.511, .158] | [-.786, -.093] | [-1.035, -.335] | [-.915, -.251] | [-.935, -.264] | [-1.481, -.679] | [-.262, .426] | [-.758, .069] |
|  | (.475) | (.010) | (.301) | (.013) | (<.001) | (.001) | (<.001) | (<.001) | (.640) | (.102) |
| income=2 | -.231 | -.065 | .070 | -.104 | .099 | .150 | .239 | -.089 | -.127 | .018 |
|  | .150 | .159 | .157 | .161 | .165 | .159 | .166 | .183 | .181 | .207 |
|  | [-.524, .062] | [-.378, .247] | [-.238, .378] | [-.421, .212] | [-.225, .423] | [-.162, .462] | [-.086, .564] | [-.449, .270] | [-.482, .227] | [-.388, .423] |
|  | (.123) | (.682) | (.654) | (.518) | (.549) | (.347) | (.149) | (.626) | (.481) | (.932) |
| income=3 | -.059 | -.149 | .054 | -.021 | .028 | .067 | .121 | -.343 | .036 | .106 |
|  | .146 | .158 | .158 | .155 | .159 | .163 | .163 | .181 | .180 | .214 |
|  | [-.345, .227] | [-.459, .162] | [-.255, .364] | [-.324, .282] | [-.283, .339] | [-.252, .386] | [-.200, .441] | [-.697, .012] | [-.317, .389] | [-.313, .526] |
|  | (.685) | (.348) | (.731) | (.892) | (.859) | (.681) | (.460) | (.058) | (.843) | (.619) |
| income=4 | -.018 | -.125 | .136 | -.047 | .073 | -.123 | .053 | -.256 | -.003 | .233 |
|  | .145 | .160 | .155 | .156 | .160 | .168 | .170 | .187 | .176 | .205 |
|  | [-.303, .267] | [-.440, .190] | [-.167, .440] | [-.353, .258] | [-.241, .388] | [-.452, .206] | [-.280, .386] | [-.622, .110] | [-.348, .343] | [-.169, .635] |
|  | (.901) | (.436) | (.378) | (.761) | (.647) | (.463) | (.756) | (.170) | (.988) | (.256) |
| income=5 | -.132 | -.173 | .079 | .147 | -.018 | .145 | .192 | -.494 | .085 | .114 |
|  | .142 | .148 | .156 | .153 | .161 | .163 | .167 | .172 | .176 | .202 |
|  | [-.411, .146] | [-.463, .117] | [-.226, .385] | [-.153, .448] | [-.333, .297] | [-.174, .465] | [-.136, .520] | [-.832, -.156] | [-.260, .430] | [-.281, .510] |
|  | (.351) | (.241) | (.612) | (.337) | (.912) | (.373) | (.251) | (.004) | (.631) | (.571) |
| income=6 | -.150 | -.106 | .068 | .248 | .038 | .135 | .209 | -.282 | .068 | .270 |
|  | .135 | .158 | .158 | .154 | .158 | .162 | .163 | .181 | .174 | .207 |
|  | [-.415, .116] | [-.415, .203] | [-.242, .378] | [-.053, .550] | [-.271, .347] | [-.183, .454] | [-.110, .529] | [-.638, .073] | [-.273, .410] | [-.137, .677] |
|  | (.269) | (.502) | (.666) | (.107) | (.809) | (.405) | (.199) | (.120) | (.694) | (.193) |
| income=7 | -.128 | -.165 | .134 | .121 | .218 | .273 | .112 | -.478 | .043 | .009 |
|  | .157 | .166 | .162 | .171 | .159 | .174 | .186 | .202 | .195 | .222 |
|  | [-.436, .181] | [-.491, .161] | [-.184, .451] | [-.216, .457] | [-.094, .530] | [-.068, .614] | [-.253, .478] | [-.874, -.081] | [-.338, .425] | [-.426, .443] |
|  | (.418) | (.321) | (.409) | (.482) | (.171) | (.116) | (.547) | (.018) | (.823) | (.969) |
| income=8 | -.138 | -.112 | .084 | -.046 | -.066 | -.032 | .057 | -.349 | -.083 | .024 |
|  | .150 | .168 | .163 | .166 | .170 | .178 | .174 | .189 | .185 | .215 |
|  | [-.433, .157] | [-.442, .218] | [-.236, .404] | [-.372, .279] | [-.400, .268] | [-.381, .316] | [-.285, .398] | [-.719, .021] | [-.447, .281] | [-.399, .446] |
|  | (.359) | (.505) | (.606) | (.780) | (.699) | (.855) | (.745) | (.065) | (.655) | (.912) |
| income=9 | -.028 | -.257 | .062 | -.029 | -.204 | .056 | -.015 | -.567 | -.022 | .020 |
|  | .146 | .158 | .162 | .163 | .171 | .171 | .176 | .180 | .188 | .213 |
|  | [-.315, .260] | [-.567, .054] | [-.255, .380] | [-.349, .290] | [-.538, .131] | [-.280, .391] | [-.361, .331] | [-.921, -.213] | [-.391, .346] | [-.397, .437] |
|  | (.851) | (.105) | (.699) | (.857) | (.232) | (.745) | (.931) | (.002) | (.905) | (.925) |
| income=10 | -.090 | -.327 | .062 | -.091 | -.043 | -.113 | .053 | -.610 | -.073 | -.121 |
|  | .148 | .165 | .168 | .168 | .177 | .181 | .186 | .193 | .191 | .224 |
|  | [-.381, .200] | [-.650, -.004] | [-.266, .391] | [-.421, .238] | [-.391, .305] | [-.468, .242] | [-.312, .417] | [-.988, -.232] | [-.448, .301] | [-.559, .318] |
|  | (.542) | (.047) | (.710) | (.587) | (.809) | (.533) | (.777) | (.002) | (.701) | (.589) |
| income=11 | -.056 | -.307 | .102 | -.150 | -.149 | .004 | -.031 | -.609 | -.060 | .068 |
|  | .148 | .175 | .170 | .173 | .184 | .186 | .185 | .192 | .189 | .224 |
|  | [-.347, .235] | [-.650, .037] | [-.232, .436] | [-.488, .189] | [-.510, .211] | [-.362, .369] | [-.394, .333] | [-.986, -.232] | [-.430, .310] | [-.370, .506] |
|  | (.704) | (.080) | (.549) | (.386) | (.416) | (.985) | (.869) | (.002) | (.750) | (.761) |
| income=12 | -.184 | -.254 | .042 | -.143 | -.188 | .038 | .221 | -.653 | .030 | -.198 |
|  | .168 | .196 | .181 | .187 | .199 | .194 | .202 | .206 | .203 | .234 |
|  | [-.514, .146] | [-.640, .131] | [-.313, .398] | [-.511, .224] | [-.577, .201] | [-.343, .420] | [-.174, .616] | [-1.057, -.249] | [-.367, .428] | [-.656, .260] |
|  | (.275) | (.196) | (.817) | (.445) | (.344) | (.843) | (.273) | (.002) | (.881) | (.396) |
| income=13 | .108 | -.446 | -.280 | -.223 | -.323 | .133 | .117 | -.923 | -.339 | -.132 |
|  | .195 | .234 | .268 | .234 | .291 | .241 | .232 | .259 | .261 | .327 |
|  | [-.273, .490] | [-.905, .013] | [-.806, .246] | [-.681, .236] | [-.894, .248] | [-.341, .606] | [-.338, .572] | [-1.430, -.415] | [-.851, .174] | [-.773, .509] |
|  | (.577) | (.057) | (.297) | (.341) | (.267) | (.583) | (.614) | (<.001) | (.195) | (.686) |
| income=14 | -.111 | -.457 | .147 | .107 | .059 | .346 | .120 | -.727 | -.225 | .042 |
|  | .221 | .262 | .211 | .261 | .319 | .255 | .303 | .280 | .291 | .348 |
|  | [-.544, .323] | [-.970, .056] | [-.266, .560] | [-.404, .618] | [-.566, .683] | [-.155, .846] | [-.475, .715] | [-1.277, -.178] | [-.796, .346] | [-.639, .724] |
|  | (.616) | (.081) | (.486) | (.682) | (.854) | (.175) | (.692) | (.010) | (.439) | (.903) |
| income=15 | .174 | -.416 | -.549 | .320 | .704 | .609 | -.501 | -.330 | 1.003 | .510 |
|  | .712 | .925 | .570 | .555 | .307 | .242 | .959 | .943 | .318 | .813 |
|  | [-1.221,1.570] | [-2.230,1.398] | [-1.667, .568] | [-.768,1.407] | [.103,1.306] | [.135,1.083] | [-2.381,1.379] | [-2.180,1.520] | [.378,1.627] | [-1.086,2.105] |
|  | (.806) | (.653) | (.335) | (.564) | (.022) | (.012) | (.601) | (.727) | (.002) | (.531) |
| income=16 | -.031 | -.179 | .276 | -.154 | .118 | .510 | .537 | -.744 | -.022 | .305 |
|  | .315 | .371 | .314 | .377 | .299 | .245 | .267 | .425 | .476 | .424 |
|  | [-.649, .587] | [-.907, .549] | [-.340, .892] | [-.892, .585] | [-.468, .704] | [.028, .991] | [.014,1.061] | [-1.577, .089] | [-.956, .912] | [-.527,1.136] |
|  | (.922) | (.630) | (.380) | (.683) | (.694) | (.038) | (.044) | (.080) | (.963) | (.473) |
| income=17 | -.281 | -.105 | -.101 | .051 | -.122 | .075 | .111 | -.453 | .005 | -.022 |
|  | .132 | .145 | .146 | .142 | .148 | .149 | .155 | .165 | .164 | .188 |
|  | [-.539, -.022] | [-.390, .179] | [-.388, .185] | [-.228, .330] | [-.411, .168] | [-.218, .368] | [-.192, .415] | [-.777, -.128] | [-.316, .326] | [-.390, .346] |
|  | (.034) | (.468) | (.488) | (.719) | (.410) | (.616) | (.472) | (.006) | (.976) | (.907) |
| Rural Code=2 | -.142 | -.033 | -.079 | .030 | .011 | .035 | -.071 | .019 | -.163 | -.063 |
|  | .068 | .072 | .066 | .065 | .078 | .073 | .073 | .082 | .074 | .092 |
|  | [-.275, -.010] | [-.174, .108] | [-.209, .050] | [-.099, .158] | [-.142, .165] | [-.109, .179] | [-.214, .073] | [-.142, .179] | [-.308, -.019] | [-.245, .118] |
|  | (.036) | (.647) | (.230) | (.650) | (.887) | (.633) | (.333) | (.820) | (.027) | (.493) |
| Rural Code=3 | -.266 | -.140 | -.082 | -.018 | -.174 | -.116 | -.087 | .176 | -.158 | .102 |
|  | .116 | .128 | .111 | .111 | .136 | .120 | .128 | .138 | .129 | .152 |
|  | [-.494, -.039] | [-.390, .111] | [-.299, .136] | [-.236, .199] | [-.442, .093] | [-.350, .119] | [-.337, .164] | [-.095, .446] | [-.411, .095] | [-.196, .399] |
|  | (.022) | (.274) | (.461) | (.870) | (.201) | (.334) | (.499) | (.202) | (.221) | (.503) |
| Rural Code=4 | -.220 | .243 | .069 | .074 | -.151 | .342 | -.205 | .313 | .191 | .260 |
|  | .262 | .297 | .246 | .212 | .332 | .204 | .305 | .262 | .329 | .373 |
|  | [-.734, .293] | [-.339, .826] | [-.413, .552] | [-.343, .491] | [-.803, .500] | [-.058, .741] | [-.803, .392] | [-.202, .828] | [-.454, .837] | [-.472, .992] |
|  | (.400) | (.413) | (.778) | (.727) | (.649) | (.094) | (.500) | (.233) | (.561) | (.486) |
| Rural Code=6 | 1.055 | .263 | .834 | .573 | -1.237 | -.765 | .449 | -1.817 | -.402 | -.601 |
|  | .392 | .352 | .222 | .202 | .194 | .399 | .182 | .197 | 1.254 | .637 |
|  | [.286,1.825] | [-.428, .954] | [.399,1.269] | [.176, .970] | [-1.616, -.857] | [-1.548, .018] | [.092, .805] | [-2.203, -1.430] | [-2.862,2.059] | [-1.849, .648] |
|  | (.007) | (.455) | (<.001) | (.005) | (<.001) | (.055) | (.014) | (<.001) | (.749) | (.346) |
| Arizona | .114 | -.064 | -.481 | -.152 | -.358 | -.179 | -.192 | .112 | -.071 | -.473 |
|  | .225 | .290 | .242 | .289 | .341 | .278 | .280 | .269 | .304 | .389 |
|  | [-.327, .555] | [-.632, .504] | [-.956, -.007] | [-.719, .415] | [-1.027, .310] | [-.724, .367] | [-.740, .357] | [-.416, .640] | [-.668, .526] | [-1.235, .290] |
|  | (.613) | (.825) | (.047) | (.598) | (.293) | (.521) | (.493) | (.677) | (.816) | (.224) |
| Arkansas | .161 | .310 | -.277 | .315 | .014 | -.162 | .096 | -.214 | -.134 | -.224 |
|  | .335 | .369 | .366 | .335 | .414 | .412 | .446 | .359 | .350 | .528 |
|  | [-.495, .818] | [-.413,1.033] | [-.996, .441] | [-.341, .971] | [-.798, .825] | [-.969, .646] | [-.780, .972] | [-.917, .490] | [-.819, .552] | [-1.260, .811] |
|  | (.629) | (.401) | (.449) | (.346) | (.973) | (.694) | (.830) | (.551) | (.702) | (.671) |
| California | .053 | .147 | -.199 | .216 | -.144 | -.083 | .081 | .351 | .151 | -.190 |
|  | .187 | .263 | .198 | .258 | .313 | .232 | .245 | .237 | .266 | .354 |
|  | [-.314, .420] | [-.368, .663] | [-.588, .190] | [-.289, .722] | [-.758, .469] | [-.539, .373] | [-.399, .561] | [-.115, .816] | [-.370, .672] | [-.885, .505] |
|  | (.776) | (.576) | (.317) | (.401) | (.645) | (.721) | (.741) | (.140) | (.570) | (.593) |
| Colorado | -.395 | .338 | -.124 | .217 | .011 | .209 | .171 | .228 | .041 | .142 |
|  | .262 | .292 | .237 | .327 | .368 | .282 | .305 | .307 | .305 | .409 |
|  | [-.908, .119] | [-.235, .911] | [-.588, .340] | [-.424, .858] | [-.711, .733] | [-.344, .762] | [-.427, .770] | [-.374, .831] | [-.556, .639] | [-.660, .944] |
|  | (.132) | (.248) | (.600) | (.507) | (.976) | (.459) | (.575) | (.458) | (.892) | (.728) |
| Connecticut | .499 | .328 | -.157 | .108 | -.117 | .282 | .340 | .538 | .434 | -.106 |
|  | .225 | .329 | .285 | .295 | .382 | .292 | .296 | .315 | .327 | .427 |
|  | [.058, .939] | [-.318, .973] | [-.716, .402] | [-.470, .686] | [-.866, .633] | [-.292, .855] | [-.240, .921] | [-.079,1.155] | [-.208,1.077] | [-.944, .732] |
|  | (.027) | (.319) | (.582) | (.715) | (.760) | (.336) | (.250) | (.087) | (.185) | (.804) |
| Delaware | .385 | .241 | .075 | -.039 | .009 | -.317 | .160 | .831 | .510 | -.556 |
|  | .403 | .332 | .438 | .371 | .551 | .347 | .510 | .661 | .524 | .696 |
|  | [-.405,1.175] | [-.411, .892] | [-.784, .935] | [-.766, .687] | [-1.072,1.089] | [-.997, .364] | [-.840,1.160] | [-.464,2.127] | [-.518,1.538] | [-1.921, .810] |
|  | (.339) | (.469) | (.864) | (.915) | (.988) | (.361) | (.754) | (.208) | (.330) | (.425) |
| Florida | .018 | .166 | -.069 | .041 | -.193 | -.019 | -.021 | .540 | .216 | -.183 |
|  | .192 | .269 | .203 | .263 | .315 | .236 | .247 | .249 | .273 | .358 |
|  | [-.358, .394] | [-.360, .693] | [-.468, .329] | [-.475, .557] | [-.810, .425] | [-.482, .444] | [-.507, .464] | [.051,1.029] | [-.319, .751] | [-.885, .520] |
|  | (.926) | (.535) | (.733) | (.877) | (.541) | (.937) | (.931) | (.030) | (.429) | (.610) |
| Georgia | -.048 | .226 | -.080 | .236 | -.169 | .004 | .040 | .478 | .001 | -.033 |
|  | .217 | .290 | .228 | .285 | .342 | .261 | .270 | .285 | .298 | .390 |
|  | [-.474, .377] | [-.342, .794] | [-.526, .367] | [-.322, .795] | [-.840, .502] | [-.507, .516] | [-.490, .571] | [-.082,1.037] | [-.584, .585] | [-.799, .733] |
|  | (.824) | (.436) | (.726) | (.407) | (.621) | (.987) | (.881) | (.094) | (.998) | (.933) |
| Idaho | .100 | .297 | .180 | .099 | -.308 | -.215 | -.427 | .631 | -.240 | -.083 |
|  | .343 | .486 | .284 | .326 | .448 | .388 | .440 | .426 | .428 | .552 |
|  | [-.573, .773] | [-.656,1.249] | [-.377, .738] | [-.540, .738] | [-1.188, .572] | [-.977, .546] | [-1.291, .437] | [-.204,1.466] | [-1.080, .600] | [-1.166,1.001] |
|  | (.771) | (.542) | (.526) | (.762) | (.492) | (.579) | (.332) | (.138) | (.575) | (.881) |
| Illinois | -.036 | .304 | -.138 | .355 | .060 | .190 | .235 | .561 | .075 | .238 |
|  | .207 | .279 | .220 | .267 | .325 | .250 | .262 | .269 | .284 | .369 |
|  | [-.442, .369] | [-.243, .850] | [-.570, .294] | [-.170, .879] | [-.577, .698] | [-.300, .680] | [-.279, .749] | [.034,1.088] | [-.482, .633] | [-.485, .961] |
|  | (.860) | (.276) | (.531) | (.185) | (.853) | (.446) | (.369) | (.037) | (.791) | (.519) |
| Indiana | .049 | -.007 | -.110 | .271 | -.067 | .065 | .249 | .195 | .232 | .113 |
|  | .247 | .308 | .284 | .292 | .382 | .310 | .303 | .329 | .350 | .431 |
|  | [-.434, .533] | [-.611, .597] | [-.668, .447] | [-.302, .844] | [-.816, .681] | [-.544, .673] | [-.345, .843] | [-.449, .839] | [-.454, .918] | [-.732, .958] |
|  | (.842) | (.982) | (.698) | (.354) | (.861) | (.835) | (.411) | (.553) | (.507) | (.793) |
| Iowa | -.200 | .710 | .081 | .200 | -.324 | .528 | .376 | .527 | .313 | -.297 |
|  | .356 | .314 | .333 | .373 | .444 | .386 | .434 | .371 | .395 | .532 |
|  | [-.897, .498] | [.093,1.326] | [-.571, .734] | [-.532, .932] | [-1.196, .548] | [-.229,1.285] | [-.475,1.227] | [-.200,1.254] | [-.461,1.087] | [-1.341, .747] |
|  | (.574) | (.024) | (.807) | (.593) | (.466) | (.172) | (.387) | (.155) | (.428) | (.576) |
| Kansas | -.568 | .019 | .023 | .307 | .514 | .441 | .375 | .261 | -.980 | -.209 |
|  | .590 | .552 | .343 | .437 | .461 | .447 | .334 | .603 | .737 | .649 |
|  | [-1.725, .589] | [-1.064,1.101] | [-.649, .696] | [-.550,1.163] | [-.391,1.419] | [-.435,1.318] | [-.280,1.031] | [-.921,1.443] | [-2.426, .467] | [-1.483,1.064] |
|  | (.336) | (.973) | (.946) | (.482) | (.266) | (.324) | (.261) | (.665) | (.184) | (.747) |
| Kentucky | -.231 | .512 | -.042 | -.025 | -.422 | -.242 | .273 | .031 | .472 | -.265 |
|  | .323 | .447 | .416 | .330 | .735 | .370 | .252 | .447 | .642 | .494 |
|  | [-.864, .402] | [-.366,1.390] | [-.857, .773] | [-.673, .623] | [-1.863,1.019] | [-.968, .485] | [-.221, .766] | [-.845, .907] | [-.786,1.730] | [-1.233, .703] |
|  | (.474) | (.253) | (.919) | (.941) | (.565) | (.514) | (.278) | (.944) | (.462) | (.592) |
| Louisiana | .019 | .051 | -.046 | -.077 | -.078 | .173 | .272 | .389 | .360 | .390 |
|  | .285 | .348 | .316 | .344 | .401 | .283 | .341 | .322 | .355 | .481 |
|  | [-.540, .579] | [-.632, .733] | [-.664, .573] | [-.752, .598] | [-.864, .708] | [-.382, .727] | [-.396, .939] | [-.242,1.021] | [-.336,1.056] | [-.553,1.333] |
|  | (.946) | (.884) | (.885) | (.822) | (.846) | (.542) | (.425) | (.227) | (.311) | (.418) |
| Maine | -.079 | 1.153 | .711 | .344 | .568 | .720 | .933 | 2.321 | -1.384 | -.520 |
|  | .760 | .518 | .379 | .363 | .467 | .384 | .336 | .458 | .552 | .839 |
|  | [-1.569,1.411] | [.137,2.169] | [-.032,1.453] | [-.368,1.056] | [-.349,1.484] | [-.033,1.472] | [.275,1.591] | [1.422,3.220] | [-2.466, -.302] | [-2.165,1.125] |
|  | (.917) | (.026) | (.061) | (.343) | (.224) | (.061) | (.005) | (<.001) | (.012) | (.536) |
| Maryland | .418 | -.216 | -.553 | -.228 | -.272 | -.117 | .023 | .162 | .105 | .165 |
|  | .214 | .322 | .300 | .342 | .393 | .351 | .343 | .344 | .339 | .436 |
|  | [-.000, .837] | [-.847, .416] | [-1.142, .036] | [-.898, .442] | [-1.043, .498] | [-.806, .572] | [-.650, .697] | [-.512, .837] | [-.560, .770] | [-.691,1.021] |
|  | (.050) | (.503) | (.066) | (.504) | (.488) | (.739) | (.946) | (.637) | (.757) | (.706) |
| Massachusetts | .016 | -.072 | .045 | .022 | -.271 | .095 | .188 | .021 | .276 | .250 |
|  | .227 | .289 | .230 | .293 | .344 | .270 | .280 | .266 | .295 | .389 |
|  | [-.430, .462] | [-.638, .494] | [-.405, .496] | [-.552, .597] | [-.946, .404] | [-.434, .624] | [-.361, .736] | [-.500, .542] | [-.303, .855] | [-.513,1.014] |
|  | (.944) | (.804) | (.843) | (.939) | (.431) | (.724) | (.502) | (.938) | (.350) | (.520) |
| Michigan | -.018 | .384 | -.033 | .116 | -.161 | .047 | .171 | .326 | -.050 | -.176 |
|  | .217 | .279 | .212 | .272 | .329 | .246 | .262 | .257 | .281 | .375 |
|  | [-.445, .408] | [-.162, .930] | [-.449, .384] | [-.418, .649] | [-.806, .484] | [-.434, .529] | [-.343, .686] | [-.178, .829] | [-.603, .502] | [-.912, .559] |
|  | (.933) | (.168) | (.878) | (.671) | (.624) | (.847) | (.513) | (.205) | (.858) | (.638) |
| Minnesota | -.042 | .087 | -.659 | .155 | -.306 | -.168 | .018 | .164 | -.050 | -.419 |
|  | .242 | .317 | .265 | .286 | .369 | .303 | .308 | .290 | .318 | .411 |
|  | [-.517, .433] | [-.534, .708] | [-1.178, -.140] | [-.406, .717] | [-1.030, .419] | [-.762, .427] | [-.586, .622] | [-.404, .732] | [-.674, .574] | [-1.226, .388] |
|  | (.863) | (.784) | (.013) | (.588) | (.408) | (.581) | (.953) | (.571) | (.875) | (.309) |
| Mississippi | .202 | .314 | -.327 | .439 | -.331 | -.119 | .698 | -.131 | .372 | -.217 |
|  | .290 | .449 | .343 | .319 | .497 | .500 | .323 | .605 | .464 | .733 |
|  | [-.366, .770] | [-.566,1.194] | [-.999, .345] | [-.186,1.065] | [-1.306, .644] | [-1.099, .862] | [.064,1.332] | [-1.318,1.056] | [-.538,1.282] | [-1.654,1.220] |
|  | (.486) | (.485) | (.340) | (.168) | (.505) | (.813) | (.031) | (.829) | (.422) | (.767) |
| Missouri | -.099 | .548 | -.003 | .215 | -.132 | .304 | .327 | .529 | -.009 | .305 |
|  | .262 | .315 | .248 | .300 | .362 | .274 | .283 | .303 | .313 | .406 |
|  | [-.613, .414] | [-.069,1.165] | [-.489, .484] | [-.374, .805] | [-.843, .578] | [-.234, .842] | [-.229, .883] | [-.066,1.123] | [-.624, .606] | [-.492,1.102] |
|  | (.704) | (.082) | (.991) | (.473) | (.715) | (.268) | (.249) | (.081) | (.977) | (.453) |
| Montana | .914 | .316 | -.330 | .743 | -.864 | -.251 | -.165 | 1.157 | .467 | -.502 |
|  | .773 | 1.428 | 1.221 | .439 | 1.390 | 1.139 | 1.151 | 1.362 | .405 | 1.289 |
|  | [-.602,2.430] | [-2.486,3.118] | [-2.724,2.064] | [-.119,1.605] | [-3.591,1.863] | [-2.484,1.982] | [-2.422,2.092] | [-1.514,3.828] | [-.327,1.262] | [-3.030,2.026] |
|  | (.237) | (.825) | (.787) | (.091) | (.534) | (.826) | (.886) | (.396) | (.249) | (.697) |
| Nebraska | -.058 | .260 | -.074 | .506 | -.401 | -.987 | -.244 | .451 | .023 | -.149 |
|  | .397 | .504 | .364 | .377 | .599 | .499 | .515 | .456 | .450 | .617 |
|  | [-.838, .721] | [-.727,1.248] | [-.788, .641] | [-.233,1.246] | [-1.575, .773] | [-1.966, -.007] | [-1.254, .766] | [-.443,1.344] | [-.859, .905] | [-1.359,1.060] |
|  | (.884) | (.605) | (.840) | (.180) | (.503) | (.048) | (.635) | (.323) | (.959) | (.808) |
| Nevada | -.015 | .080 | -.004 | .160 | -.113 | -.064 | .168 | .251 | -.124 | -.168 |
|  | .237 | .296 | .257 | .305 | .357 | .284 | .300 | .294 | .323 | .410 |
|  | [-.479, .449] | [-.501, .662] | [-.507, .499] | [-.438, .759] | [-.814, .587] | [-.620, .493] | [-.421, .757] | [-.326, .828] | [-.759, .510] | [-.972, .636] |
|  | (.951) | (.786) | (.987) | (.599) | (.751) | (.823) | (.577) | (.394) | (.700) | (.682) |
| New Hampshire | .039 | .243 | -.232 | .620 | -.121 | .058 | .446 | .368 | .853 | -.792 |
|  | .320 | .349 | .373 | .293 | .400 | .343 | .337 | .377 | .352 | .506 |
|  | [-.589, .667] | [-.441, .926] | [-.963, .499] | [.046,1.195] | [-.906, .664] | [-.614, .730] | [-.215,1.106] | [-.373,1.108] | [.162,1.545] | [-1.786, .201] |
|  | (.903) | (.486) | (.534) | (.034) | (.763) | (.866) | (.186) | (.330) | (.016) | (.118) |
| New Jersey | -.109 | -.025 | .023 | -.103 | -.013 | -.026 | .091 | .351 | .238 | -.084 |
|  | .236 | .300 | .214 | .287 | .338 | .275 | .271 | .282 | .287 | .392 |
|  | [-.572, .353] | [-.614, .565] | [-.397, .443] | [-.666, .459] | [-.676, .651] | [-.564, .513] | [-.439, .622] | [-.203, .905] | [-.324, .800] | [-.853, .684] |
|  | (.643) | (.935) | (.913) | (.719) | (.970) | (.925) | (.735) | (.214) | (.407) | (.830) |
| New Mexico | -.147 | .630 | -.244 | .361 | .011 | .278 | .264 | .481 | .446 | .039 |
|  | .304 | .376 | .321 | .357 | .426 | .318 | .373 | .382 | .386 | .504 |
|  | [-.743, .449] | [-.109,1.368] | [-.873, .385] | [-.340,1.062] | [-.824, .847] | [-.346, .902] | [-.468, .995] | [-.267,1.229] | [-.311,1.202] | [-.949,1.027] |
|  | (.628) | (.095) | (.447) | (.313) | (.979) | (.382) | (.480) | (.208) | (.248) | (.938) |
| New York | -.016 | .430 | -.113 | .300 | -.002 | .306 | .420 | .714 | .339 | .186 |
|  | .202 | .272 | .212 | .268 | .326 | .239 | .256 | .256 | .274 | .368 |
|  | [-.413, .380] | [-.104, .964] | [-.529, .304] | [-.226, .827] | [-.642, .637] | [-.162, .774] | [-.083, .922] | [.211,1.216] | [-.198, .877] | [-.535, .907] |
|  | (.935) | (.114) | (.596) | (.263) | (.994) | (.200) | (.102) | (.005) | (.216) | (.613) |
| North Carolina | .136 | -.038 | -.153 | .021 | .285 | .145 | .281 | .389 | .009 | -.141 |
|  | .220 | .294 | .247 | .298 | .339 | .269 | .274 | .284 | .313 | .407 |
|  | [-.296, .567] | [-.614, .538] | [-.637, .332] | [-.563, .605] | [-.380, .949] | [-.384, .673] | [-.257, .818] | [-.169, .947] | [-.603, .622] | [-.940, .658] |
|  | (.538) | (.897) | (.537) | (.944) | (.401) | (.591) | (.306) | (.171) | (.976) | (.730) |
| North Dakota | .420 | .321 | -.346 | .012 | .239 | -.242 | .300 | .579 | .050 | -1.121 |
|  | .291 | .425 | .366 | .628 | .573 | .391 | .436 | .354 | .444 | .644 |
|  | [-.151, .991] | [-.512,1.155] | [-1.064, .372] | [-1.220,1.244] | [-.884,1.362] | [-1.009, .525] | [-.555,1.155] | [-.115,1.273] | [-.822, .922] | [-2.383, .142] |
|  | (.149) | (.450) | (.345) | (.985) | (.677) | (.536) | (.492) | (.102) | (.911) | (.082) |
| Ohio | .041 | .288 | -.127 | .326 | -.114 | .054 | .166 | .091 | -.001 | .150 |
|  | .220 | .280 | .226 | .273 | .333 | .268 | .270 | .267 | .292 | .378 |
|  | [-.391, .474] | [-.262, .838] | [-.570, .317] | [-.210, .862] | [-.768, .540] | [-.471, .579] | [-.364, .696] | [-.431, .614] | [-.574, .571] | [-.591, .892] |
|  | (.851) | (.304) | (.576) | (.233) | (.732) | (.840) | (.539) | (.732) | (.996) | (.691) |
| Oklahoma | -.849 | .341 | -.998 | .421 | .160 | -.578 | .079 | .371 | .063 | -.408 |
|  | .488 | .530 | .482 | .388 | .460 | .519 | .471 | .545 | .490 | .620 |
|  | [-1.805, .108] | [-.697,1.380] | [-1.942, -.054] | [-.339,1.182] | [-.741,1.062] | [-1.595, .439] | [-.844,1.002] | [-.698,1.440] | [-.898,1.024] | [-1.624, .808] |
|  | (.082) | (.519) | (.038) | (.277) | (.727) | (.265) | (.867) | (.496) | (.898) | (.510) |
| Oregon | -.087 | .090 | -.208 | .188 | -.953 | -.229 | -.361 | -.219 | .318 | -.160 |
|  | .233 | .299 | .237 | .284 | .354 | .292 | .290 | .260 | .296 | .396 |
|  | [-.545, .370] | [-.496, .677] | [-.673, .257] | [-.368, .745] | [-1.648, -.259] | [-.802, .344] | [-.929, .206] | [-.730, .292] | [-.263, .899] | [-.937, .617] |
|  | (.708) | (.763) | (.381) | (.507) | (.007) | (.433) | (.212) | (.401) | (.283) | (.687) |
| Pennsylvania | .041 | .207 | -.097 | .181 | -.104 | .047 | .364 | .163 | .044 | .139 |
|  | .191 | .269 | .209 | .264 | .325 | .242 | .249 | .248 | .276 | .358 |
|  | [-.335, .416] | [-.321, .735] | [-.507, .313] | [-.336, .698] | [-.741, .532] | [-.428, .522] | [-.125, .853] | [-.323, .649] | [-.497, .585] | [-.564, .841] |
|  | (.832) | (.443) | (.642) | (.492) | (.748) | (.847) | (.144) | (.510) | (.872) | (.699) |
| Rhode Island | -.045 | 1.151 | .175 | .297 | .563 | .418 | .644 | .982 | .044 | .106 |
|  | .685 | .486 | .310 | .613 | .345 | .387 | .389 | .606 | .574 | .689 |
|  | [-1.388,1.298] | [.199,2.104] | [-.434, .783] | [-.905,1.500] | [-.113,1.240] | [-.340,1.177] | [-.120,1.408] | [-.206,2.171] | [-1.082,1.170] | [-1.246,1.457] |
|  | (.948) | (.018) | (.573) | (.628) | (.103) | (.280) | (.098) | (.105) | (.939) | (.878) |
| South Carolina | -.544 | -.223 | -.378 | -.126 | .104 | .186 | .164 | -.148 | -.365 | .168 |
|  | .384 | .352 | .342 | .426 | .435 | .350 | .440 | .371 | .467 | .443 |
|  | [-1.297, .209] | [-.914, .468] | [-1.049, .294] | [-.961, .710] | [-.749, .957] | [-.500, .872] | [-.700,1.027] | [-.877, .580] | [-1.282, .551] | [-.700,1.037] |
|  | (.157) | (.527) | (.270) | (.768) | (.811) | (.594) | (.710) | (.690) | (.434) | (.704) |
| South Dakota | .666 | .980 | -.970 | .505 | .892 | 1.156 | .332 | .160 | .458 | .570 |
|  | .603 | .444 | 1.018 | .285 | .487 | .375 | .822 | .918 | .441 | .508 |
|  | [-.517,1.848] | [.110,1.850] | [-2.967,1.026] | [-.055,1.064] | [-.063,1.848] | [.421,1.891] | [-1.281,1.945] | [-1.639,1.960] | [-.406,1.323] | [-.426,1.566] |
|  | (.270) | (.027) | (.340) | (.077) | (.067) | (.002) | (.686) | (.861) | (.299) | (.262) |
| Tennessee | -.158 | -.128 | -.205 | .021 | -.240 | -.228 | .143 | .568 | .178 | -.409 |
|  | .264 | .327 | .268 | .302 | .370 | .305 | .325 | .373 | .329 | .436 |
|  | [-.675, .359] | [-.769, .514] | [-.731, .321] | [-.571, .613] | [-.965, .485] | [-.826, .370] | [-.493, .780] | [-.164,1.300] | [-.467, .823] | [-1.265, .446] |
|  | (.548) | (.696) | (.445) | (.944) | (.516) | (.454) | (.659) | (.128) | (.588) | (.348) |
| Texas | -.035 | .115 | -.265 | .124 | -.193 | -.023 | .183 | .402 | .066 | -.128 |
|  | .198 | .268 | .209 | .265 | .323 | .242 | .251 | .249 | .276 | .367 |
|  | [-.424, .355] | [-.411, .640] | [-.675, .144] | [-.396, .643] | [-.827, .441] | [-.498, .452] | [-.310, .675] | [-.086, .891] | [-.476, .607] | [-.847, .591] |
|  | (.862) | (.668) | (.204) | (.641) | (.551) | (.925) | (.467) | (.107) | (.812) | (.727) |
| Utah | .151 | -.135 | -.739 | -.284 | -.446 | -.202 | .252 | -.394 | .593 | -.851 |
|  | .300 | .370 | .351 | .359 | .396 | .348 | .365 | .310 | .348 | .497 |
|  | [-.437, .739] | [-.861, .592] | [-1.427, -.051] | [-.987, .419] | [-1.222, .330] | [-.884, .480] | [-.465, .968] | [-1.002, .214] | [-.089,1.276] | [-1.825, .124] |
|  | (.615) | (.716) | (.035) | (.428) | (.260) | (.561) | (.491) | (.204) | (.088) | (.087) |
| Vermont | .213 | .092 | -.155 | .978 | -1.359 | .898 | 1.060 | -.798 | -1.194 | -.800 |
|  | .259 | .330 | .278 | .321 | .371 | .305 | .309 | .329 | .352 | .430 |
|  | [-.295, .721] | [-.555, .738] | [-.699, .390] | [.349,1.607] | [-2.087, -.632] | [.300,1.497] | [.454,1.665] | [-1.445, -.152] | [-1.883, -.504] | [-1.643, .043] |
|  | (.410) | (.781) | (.578) | (.002) | (<.001) | (.003) | (.001) | (.015) | (.001) | (.063) |
| Virginia | -.485 | .684 | .412 | 1.135 | -.075 | .505 | .624 | -.572 | .823 | -1.427 |
|  | .387 | .489 | .297 | .309 | .703 | .401 | .448 | .326 | .656 | .687 |
|  | [-1.244, .273] | [-.275,1.644] | [-.171, .995] | [.528,1.742] | [-1.455,1.304] | [-.282,1.292] | [-.255,1.502] | [-1.212, .067] | [-.464,2.111] | [-2.774, -.079] |
|  | (.210) | (.162) | (.166) | (<.001) | (.915) | (.208) | (.164) | (.079) | (.210) | (.038) |
| Washington | -.033 | -.227 | -.074 | -.101 | -.753 | -.436 | -.572 | .084 | -.083 | -.781 |
|  | .236 | .298 | .239 | .300 | .350 | .280 | .294 | .285 | .317 | .388 |
|  | [-.497, .430] | [-.812, .358] | [-.541, .394] | [-.690, .488] | [-1.440, -.067] | [-.985, .113] | [-1.148, .003] | [-.475, .642] | [-.705, .539] | [-1.543, -.020] |
|  | (.888) | (.448) | (.758) | (.736) | (.031) | (.120) | (.051) | (.769) | (.794) | (.044) |
| West Virginia | -.169 | .233 | .200 | .214 | 1.007 | .423 | .998 | .280 | .019 | -1.151 |
|  | .611 | .462 | .385 | .462 | .358 | .709 | .335 | .720 | .574 | .778 |
|  | [-1.366,1.029] | [-.673,1.139] | [-.555, .954] | [-.692,1.120] | [.304,1.710] | [-.967,1.814] | [.340,1.655] | [-1.132,1.691] | [-1.106,1.144] | [-2.677, .376] |
|  | (.782) | (.614) | (.604) | (.644) | (.005) | (.550) | (.003) | (.698) | (.974) | (.140) |
| Wisconsin | .134 | .190 | -.235 | .172 | -.099 | .048 | .046 | .120 | -.245 | -.241 |
|  | .218 | .280 | .248 | .284 | .350 | .266 | .287 | .270 | .313 | .395 |
|  | [-.294, .562] | [-.360, .740] | [-.720, .251] | [-.385, .729] | [-.786, .588] | [-.474, .569] | [-.517, .609] | [-.410, .649] | [-.859, .369] | [-1.015, .534] |
|  | (.539) | (.498) | (.343) | (.544) | (.778) | (.858) | (.873) | (.658) | (.434) | (.542) |
| Wyoming | -.508 | 1.309 | -.225 | -.583 | .955 | 1.107 | .979 | 2.504 | 1.671 | -.283 |
|  | 1.527 | .739 | .758 | .976 | .324 | .247 | .264 | .329 | .289 | 1.443 |
|  | [-3.504,2.487] | [-.141,2.759] | [-1.712,1.262] | [-2.497,1.331] | [.320,1.591] | [.622,1.591] | [.461,1.496] | [1.858,3.149] | [1.105,2.237] | [-3.114,2.547] |
|  | (.739) | (.077) | (.767) | (.551) | (.003) | (<.001) | (<.001) | (<.001) | (<.001) | (.844) |
| Observations | 1891 | 1894 | 1892 | 1894 | 1898 | 1895 | 1895 | 1890 | 1894 | 1895 |
| r2_p |  |  |  |  |  |  |  |  |  |  |
| chi2 |  |  |  |  |  |  |  |  |  |  |
| df_m | 82 | 82 | 82 | 82 | 82 | 82 | 82 | 82 | 82 | 82 |
| p | 82 | 82 | 82 | 82 | 82 | 82 | 82 | 82 | 82 | 82 |

**S13 Fig. Partisanship and Health Behaviors and Attitudes.** Estimates are odds-ratios comparing Democrats and Others (unaffiliated or identifying with a third party) to self-identified Republicans. For each comparison and each dependent variable, we display estimates for four separate models: the baseline with all control variables reported in the main text, a model with no controls, a model with demographic controls only, and a model with geographic controls only. Odds ratios greater than 1 imply the respondent is more likely report a behavior or to express a view. 95% confidence intervals are adjusted for nine comparisons using a Bonferroni correction.

**S14 Fig. Partisanship and Policy Preferences.** Odds ratios greater than 1 imply the respondent is more likely to support a policy. For each comparison and each dependent variable, we display estimates for four separate models: the baseline with all control variables reported in the main text, a model with no controls, a model with demographic controls only, and a model with geographic controls only. 95% confidence intervals are adjusted for twenty comparisons using a Bonferroni correction.
